# Supplementary figures and images for: Two-way Dispatched function in Sonic hedgehog shedding and transfer to high-density lipoproteins (part 1 of 3)
Source: eLife. 2024 Sep 19;12:RP86920. doi: 10.7554/eLife.86920 (PMC11412720; doi:10.7554/eLife.86920)

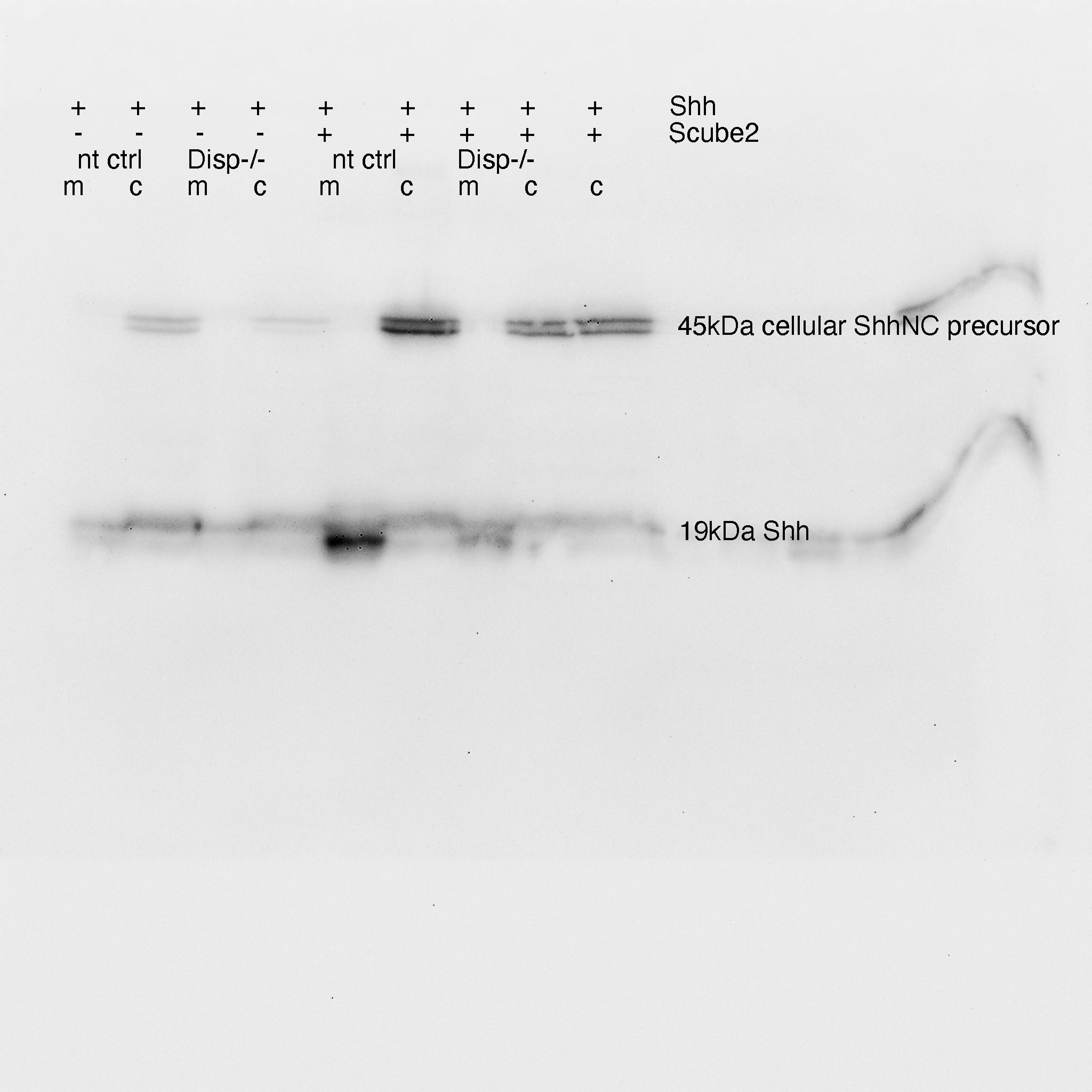

Supplement: Figure 1—source data 1. — A–D contain uncropped western blots shown in Figure 1A–D. Folders A’–D’ contain biological replicates of the respective experiments. Prizm files A’–D’ quantify relative Shh release rates based on the data shown in folders A’–D’. [file elife-86920-fig1-data1.zip › Figure_1_Source_Data_1 /A_V744_1_antiShh_1min labelled.jpg]

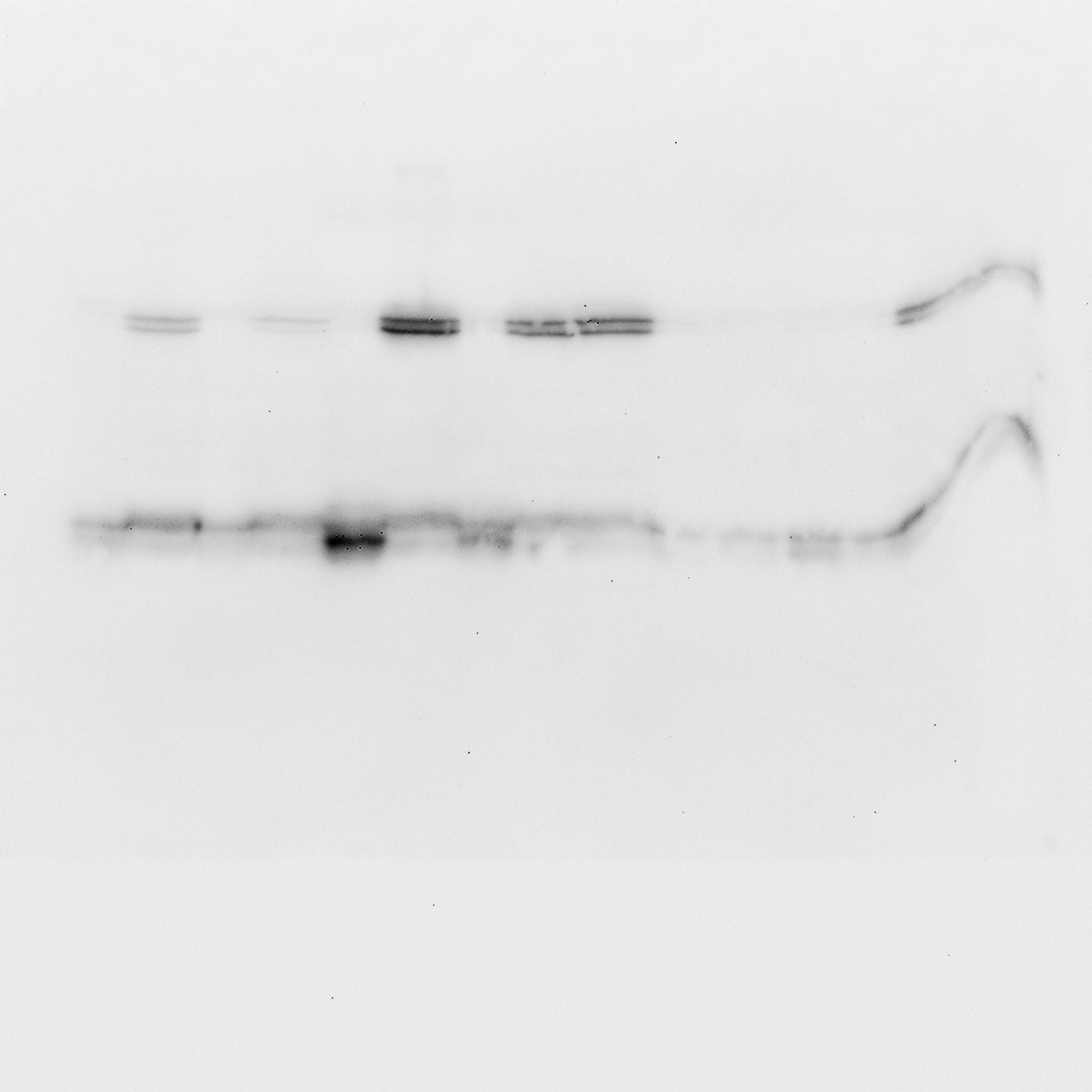

Supplement: Figure 1—source data 1. — A–D contain uncropped western blots shown in Figure 1A–D. Folders A’–D’ contain biological replicates of the respective experiments. Prizm files A’–D’ quantify relative Shh release rates based on the data shown in folders A’–D’. [file elife-86920-fig1-data1.zip › Figure_1_Source_Data_1 /A_V744_1_antiShh_1min.jpg]

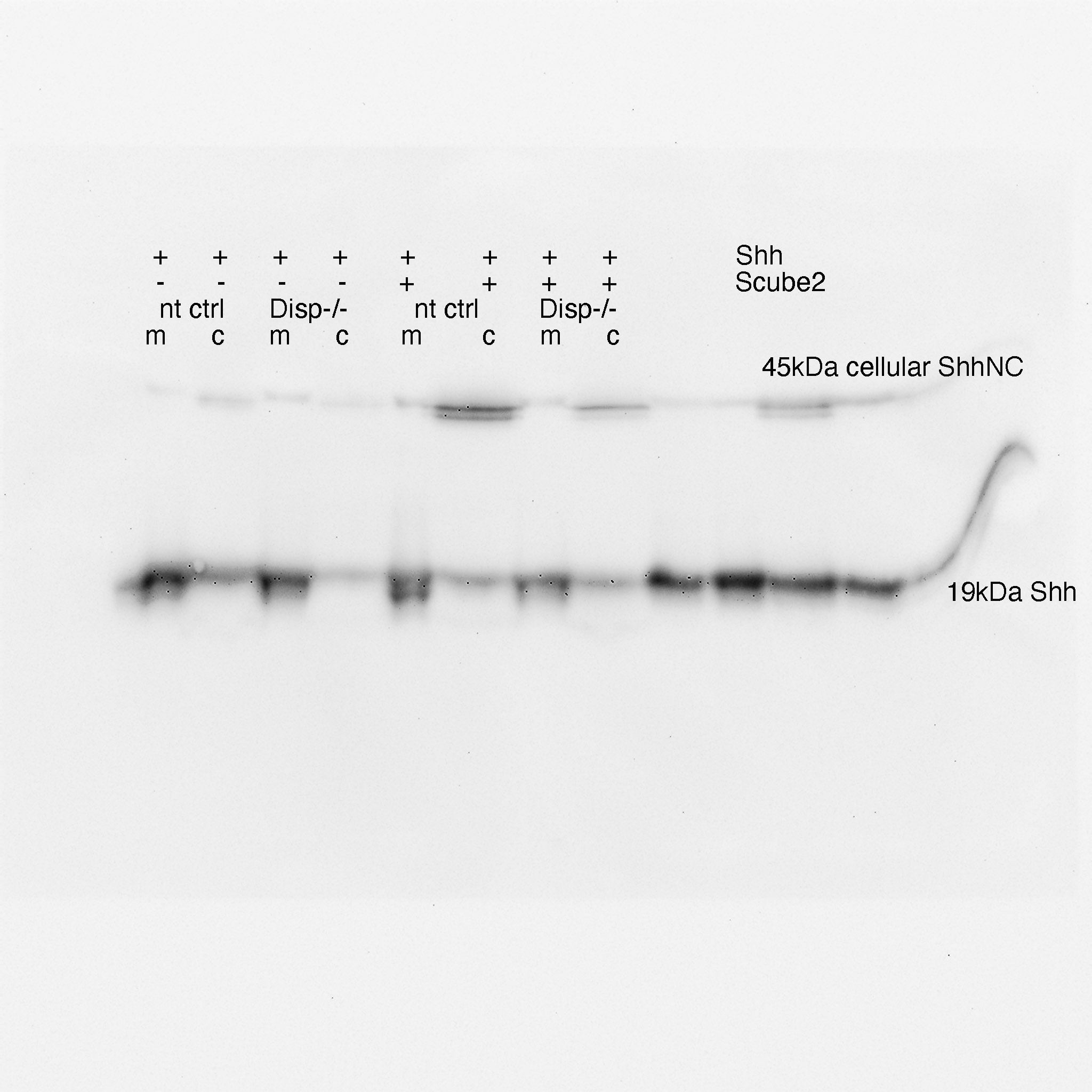

Supplement: Figure 1—source data 1. — A–D contain uncropped western blots shown in Figure 1A–D. Folders A’–D’ contain biological replicates of the respective experiments. Prizm files A’–D’ quantify relative Shh release rates based on the data shown in folders A’–D’. [file elife-86920-fig1-data1.zip › Figure_1_Source_Data_1 /B_V749_2_antiShh_2min labelled.jpg]

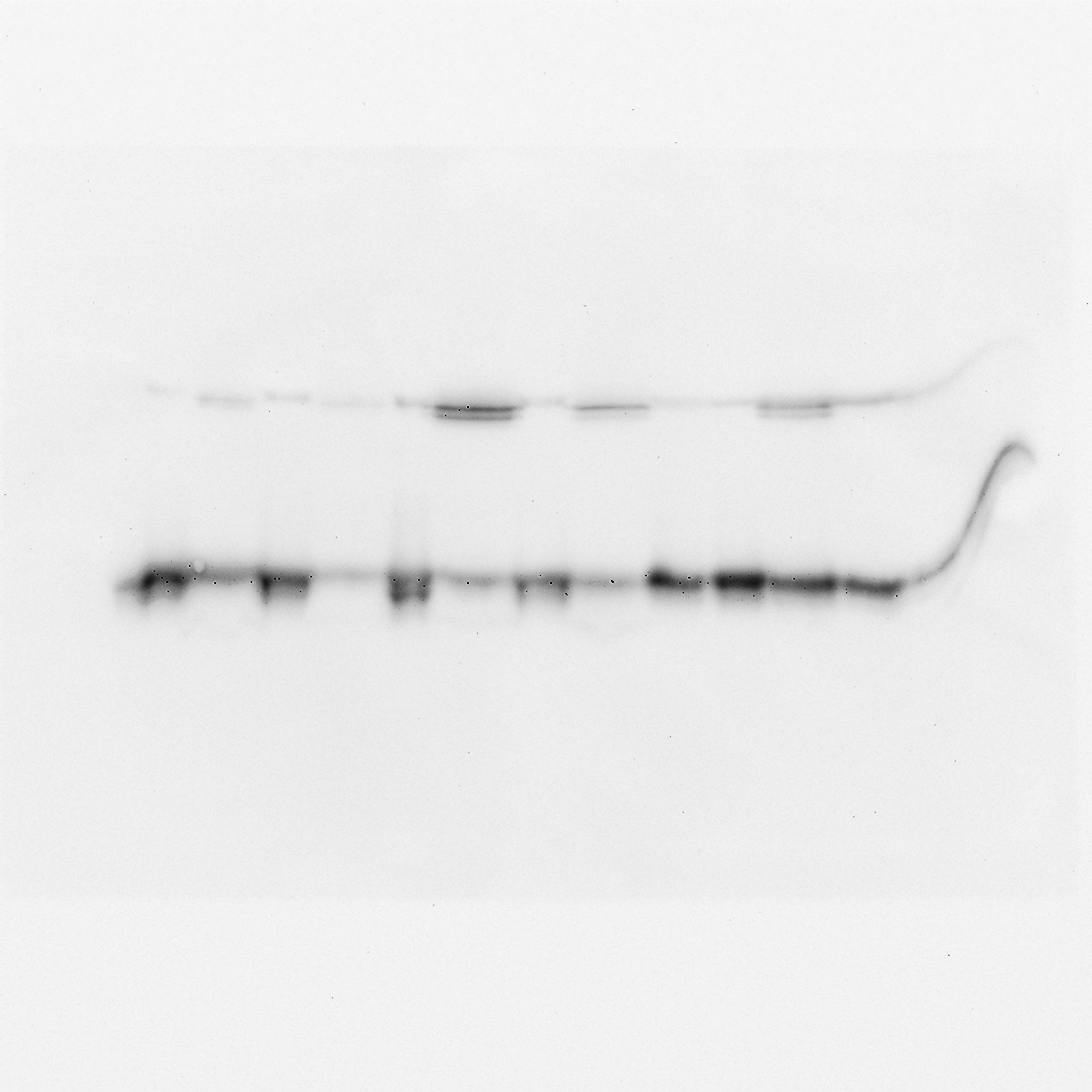

Supplement: Figure 1—source data 1. — A–D contain uncropped western blots shown in Figure 1A–D. Folders A’–D’ contain biological replicates of the respective experiments. Prizm files A’–D’ quantify relative Shh release rates based on the data shown in folders A’–D’. [file elife-86920-fig1-data1.zip › Figure_1_Source_Data_1 /B_V749_2_antiShh_2min.jpg]

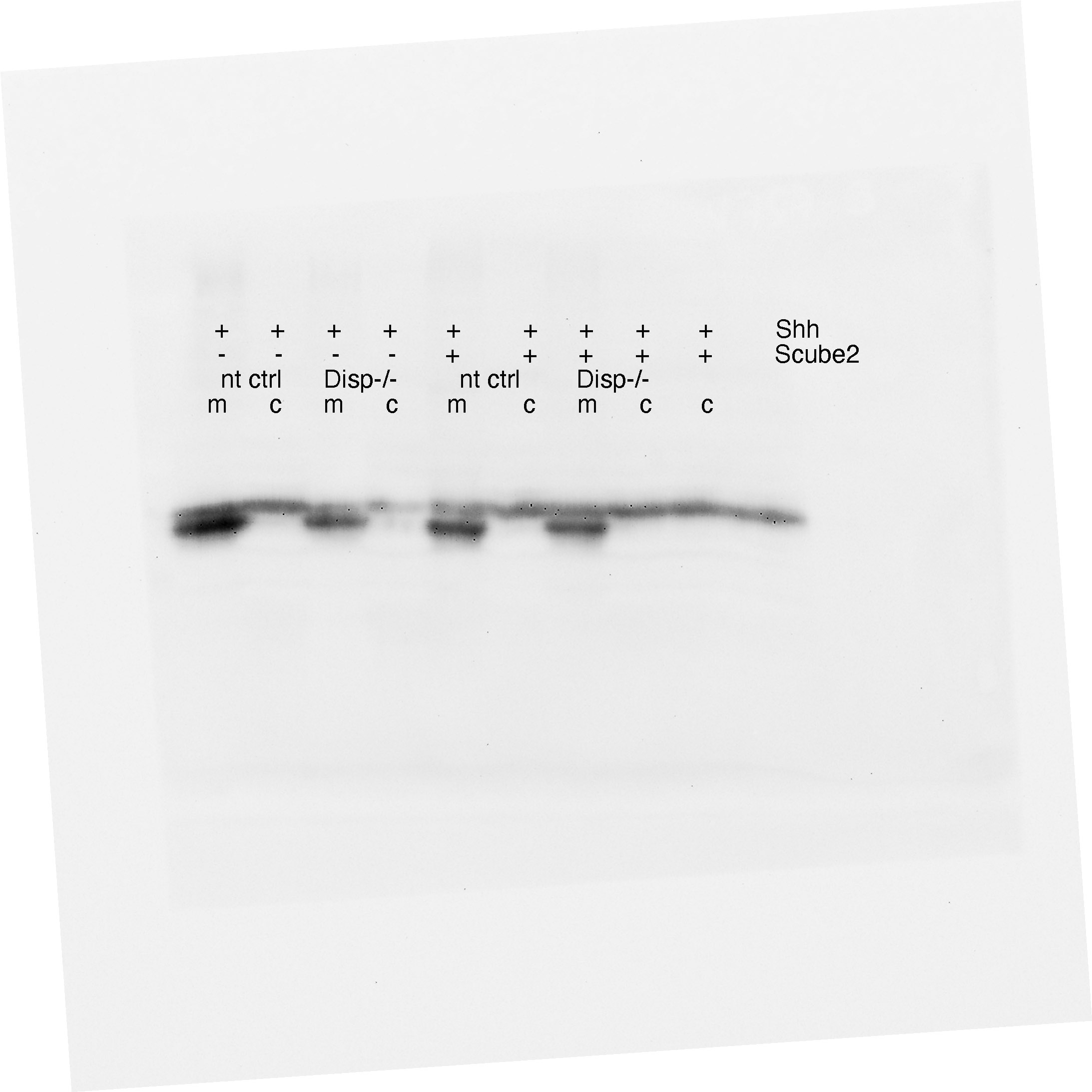

Supplement: Figure 1—source data 1. — A–D contain uncropped western blots shown in Figure 1A–D. Folders A’–D’ contain biological replicates of the respective experiments. Prizm files A’–D’ quantify relative Shh release rates based on the data shown in folders A’–D’. [file elife-86920-fig1-data1.zip › Figure_1_Source_Data_1 /C_V757_3_0.5min_antiShh labelled.jpg]

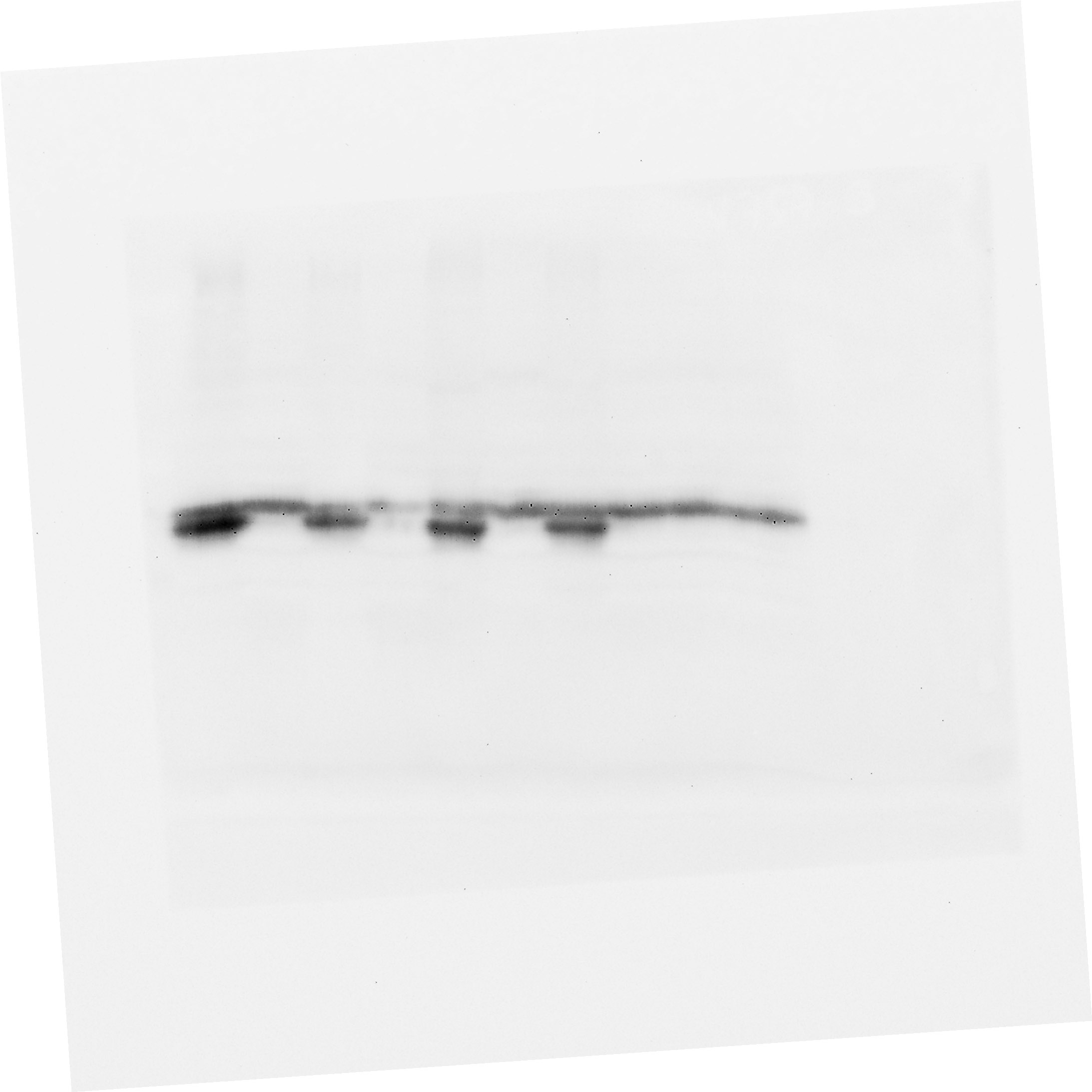

Supplement: Figure 1—source data 1. — A–D contain uncropped western blots shown in Figure 1A–D. Folders A’–D’ contain biological replicates of the respective experiments. Prizm files A’–D’ quantify relative Shh release rates based on the data shown in folders A’–D’. [file elife-86920-fig1-data1.zip › Figure_1_Source_Data_1 /C_V757_3_0.5min_antiShh.jpg]

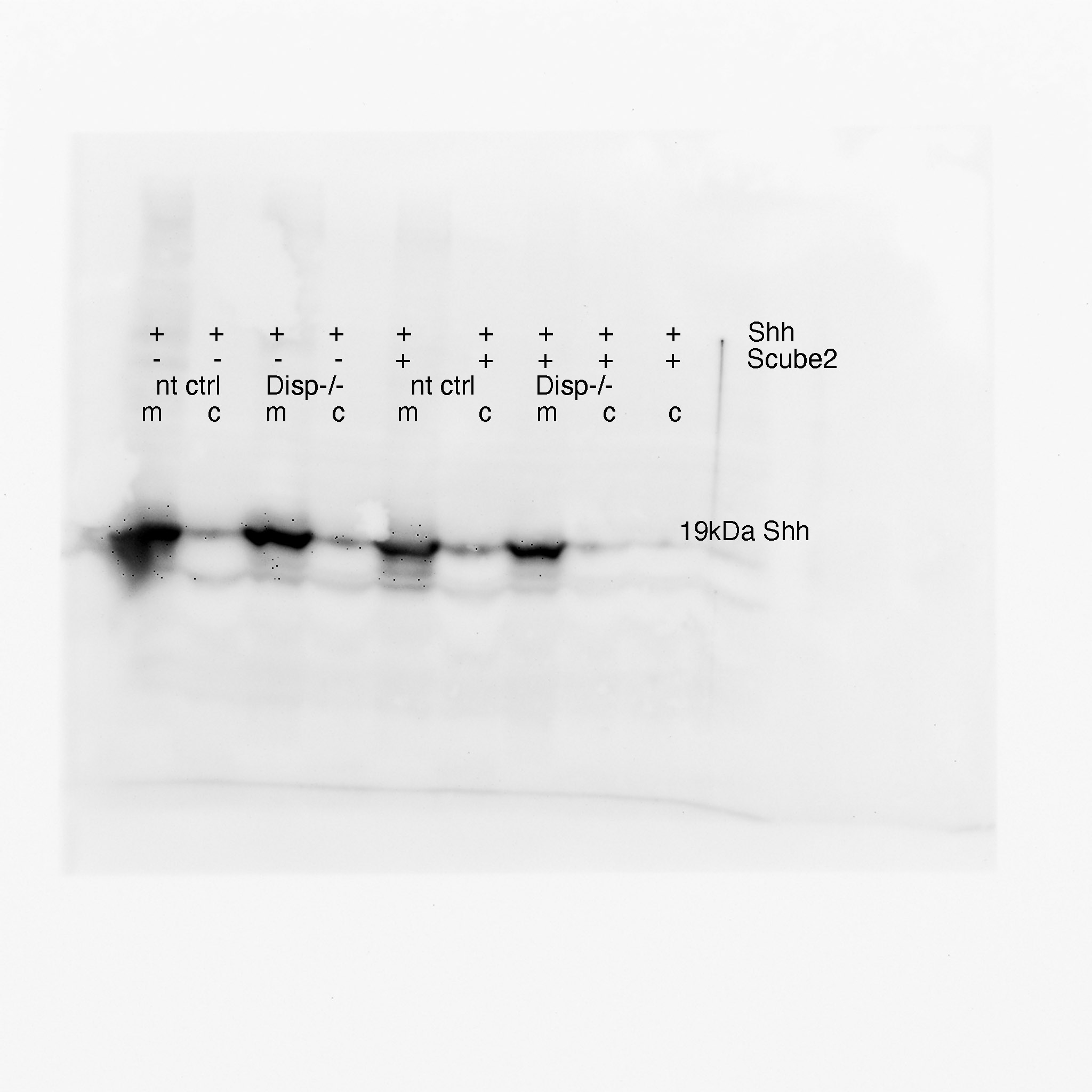

Supplement: Figure 1—source data 1. — A–D contain uncropped western blots shown in Figure 1A–D. Folders A’–D’ contain biological replicates of the respective experiments. Prizm files A’–D’ quantify relative Shh release rates based on the data shown in folders A’–D’. [file elife-86920-fig1-data1.zip › Figure_1_Source_Data_1 /D_V757_6_0.5min_antiShh labelled.jpg]

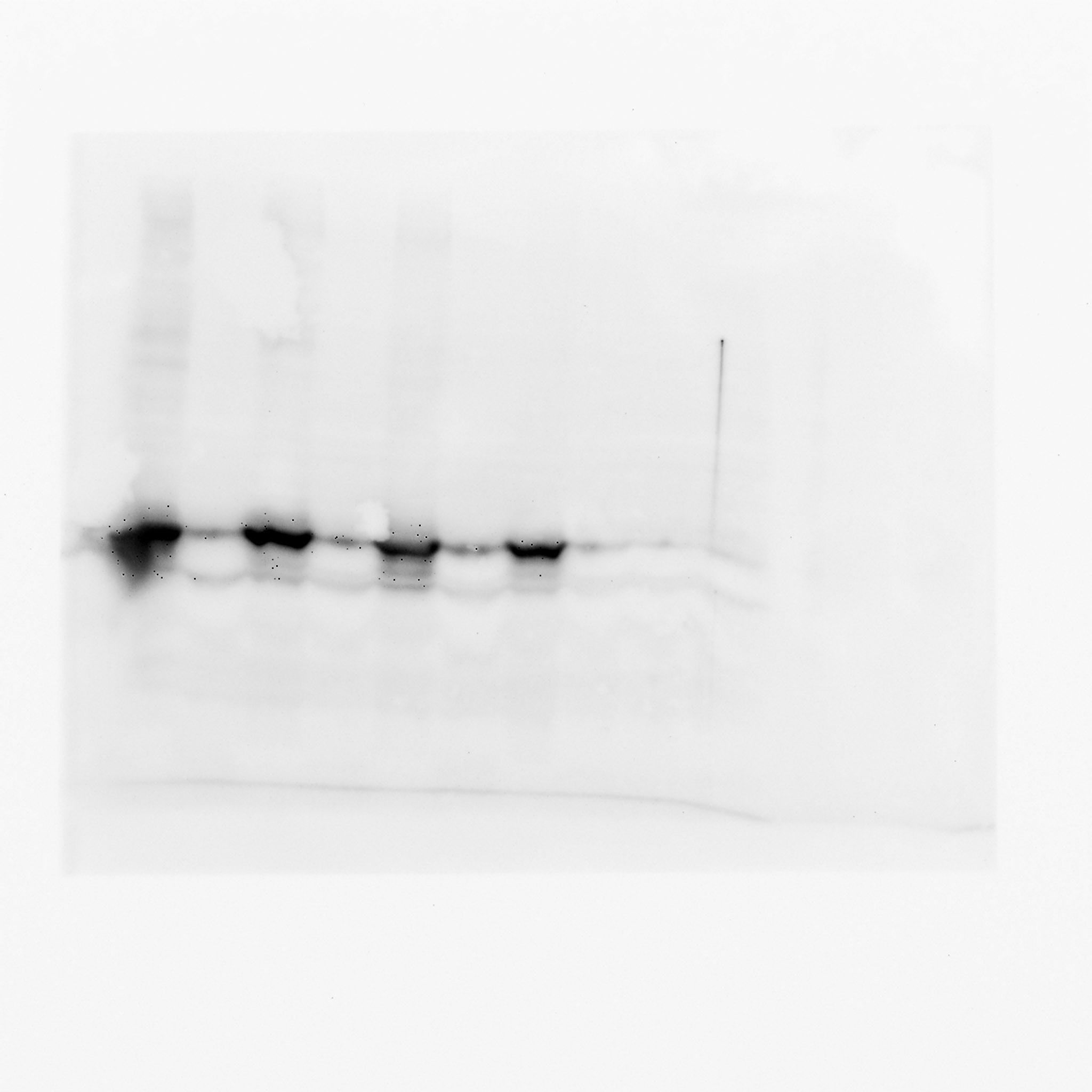

Supplement: Figure 1—source data 1. — A–D contain uncropped western blots shown in Figure 1A–D. Folders A’–D’ contain biological replicates of the respective experiments. Prizm files A’–D’ quantify relative Shh release rates based on the data shown in folders A’–D’. [file elife-86920-fig1-data1.zip › Figure_1_Source_Data_1 /D_V757_6_0.5min_antiShh.jpg]

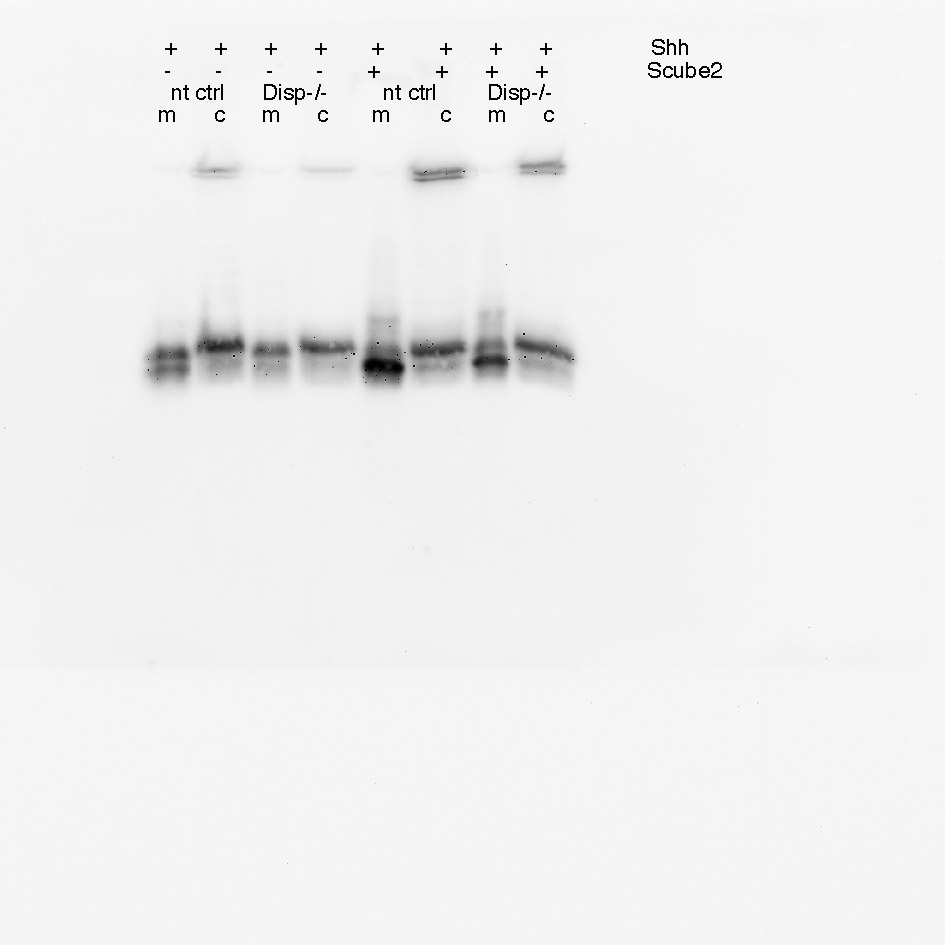

Supplement: Figure 1—source data 1. — A–D contain uncropped western blots shown in Figure 1A–D. Folders A’–D’ contain biological replicates of the respective experiments. Prizm files A’–D’ quantify relative Shh release rates based on the data shown in folders A’–D’. [file elife-86920-fig1-data1.zip › Figure_1_Source_Data_1 /A'_Fig_1_Quant_H7/14-11-23_Gel 2_antiShh Rabbit_1min labelled.jpg]

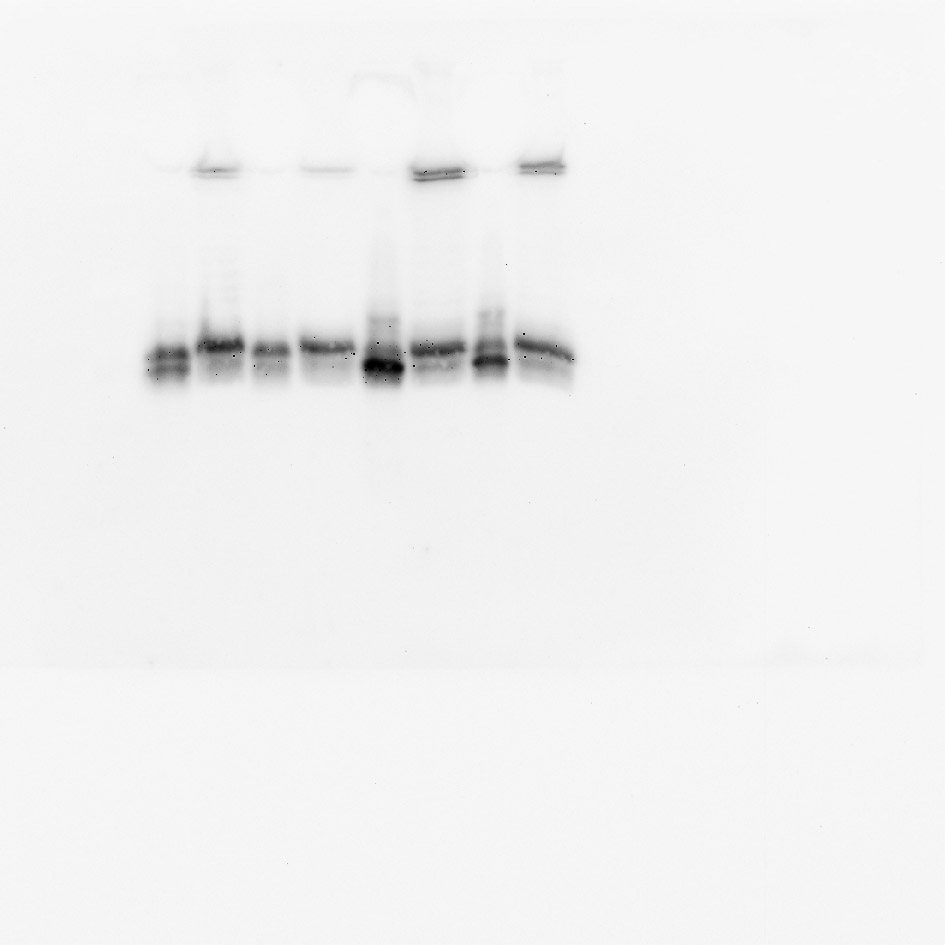

Supplement: Figure 1—source data 1. — A–D contain uncropped western blots shown in Figure 1A–D. Folders A’–D’ contain biological replicates of the respective experiments. Prizm files A’–D’ quantify relative Shh release rates based on the data shown in folders A’–D’. [file elife-86920-fig1-data1.zip › Figure_1_Source_Data_1 /A'_Fig_1_Quant_H7/14-11-23_Gel 2_antiShh Rabbit_1min.jpg]

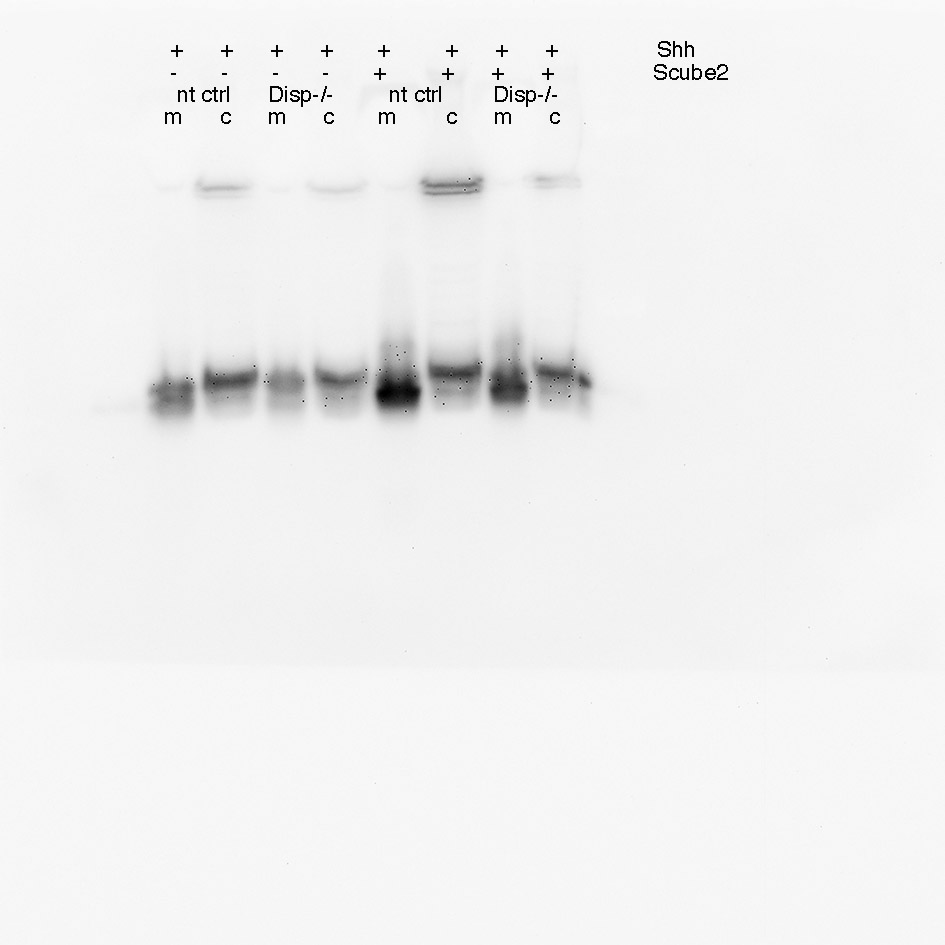

Supplement: Figure 1—source data 1. — A–D contain uncropped western blots shown in Figure 1A–D. Folders A’–D’ contain biological replicates of the respective experiments. Prizm files A’–D’ quantify relative Shh release rates based on the data shown in folders A’–D’. [file elife-86920-fig1-data1.zip › Figure_1_Source_Data_1 /A'_Fig_1_Quant_H7/14-11-23_Gel 3_antiShh Rabbit_1min labelled.jpg]

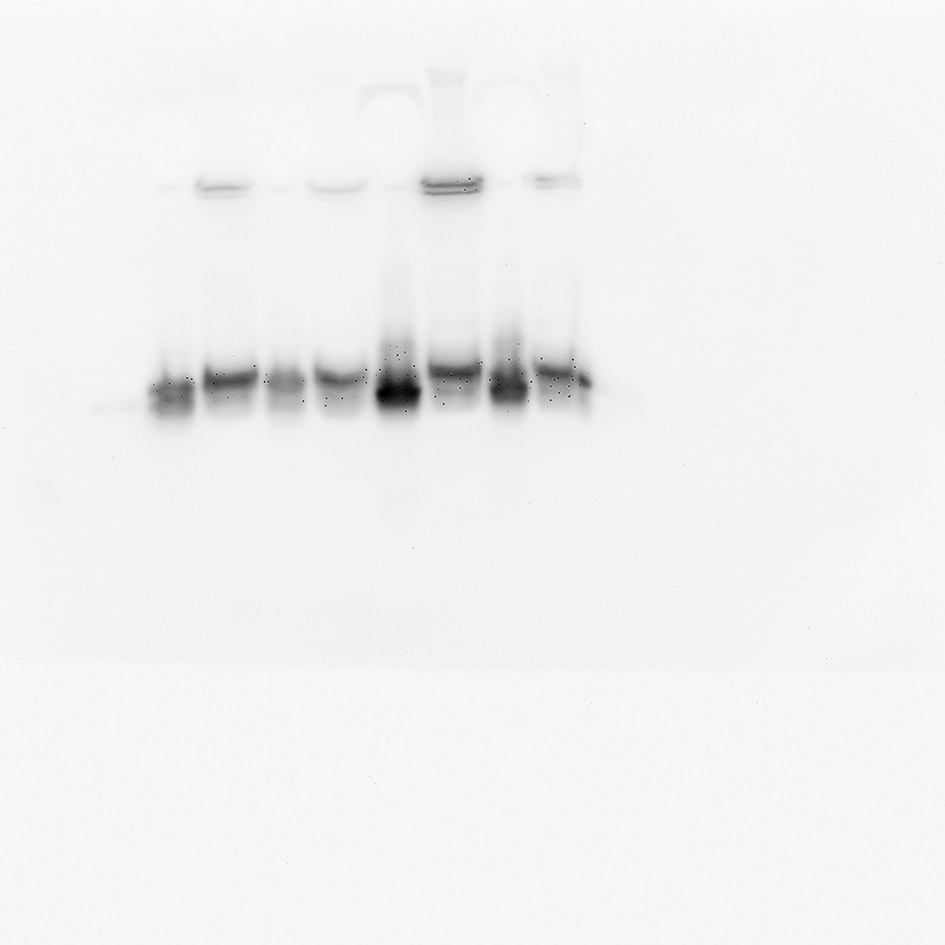

Supplement: Figure 1—source data 1. — A–D contain uncropped western blots shown in Figure 1A–D. Folders A’–D’ contain biological replicates of the respective experiments. Prizm files A’–D’ quantify relative Shh release rates based on the data shown in folders A’–D’. [file elife-86920-fig1-data1.zip › Figure_1_Source_Data_1 /A'_Fig_1_Quant_H7/14-11-23_Gel 3_antiShh Rabbit_1min.jpg]

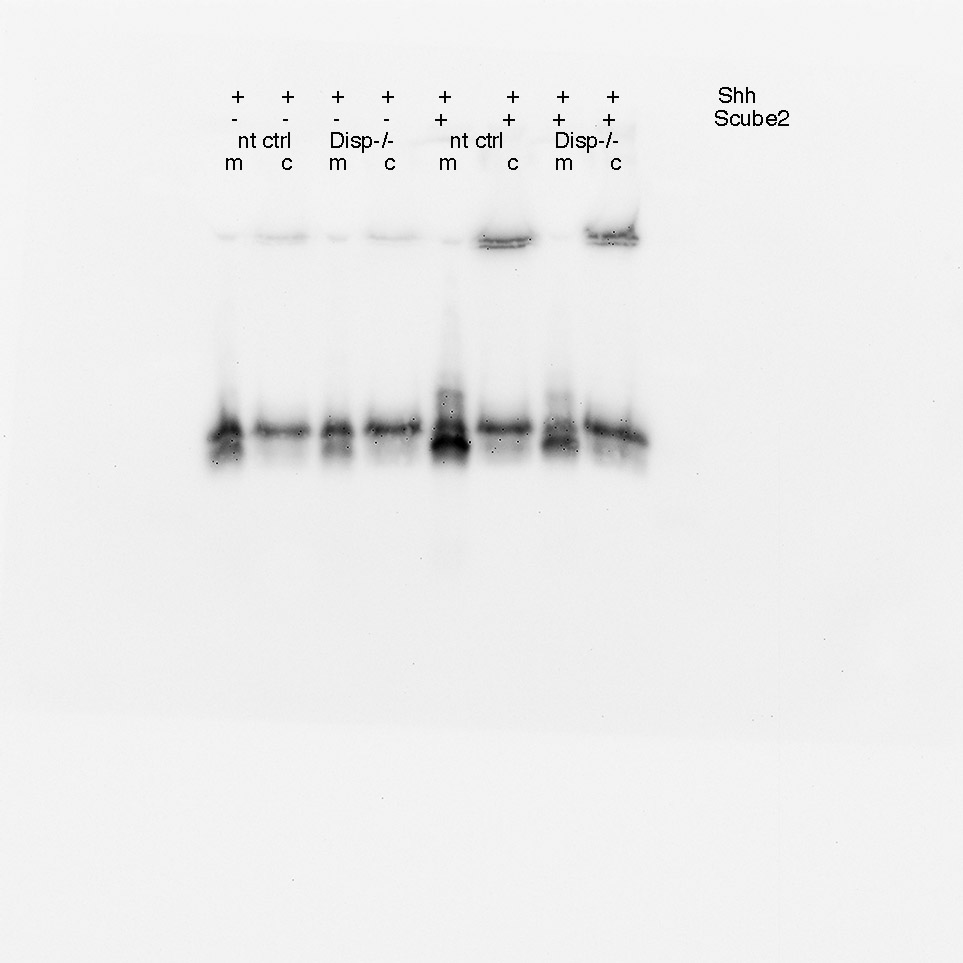

Supplement: Figure 1—source data 1. — A–D contain uncropped western blots shown in Figure 1A–D. Folders A’–D’ contain biological replicates of the respective experiments. Prizm files A’–D’ quantify relative Shh release rates based on the data shown in folders A’–D’. [file elife-86920-fig1-data1.zip › Figure_1_Source_Data_1 /A'_Fig_1_Quant_H7/14-11-23_Gel 4_antiShh Rabbit_1min labelled.jpg]

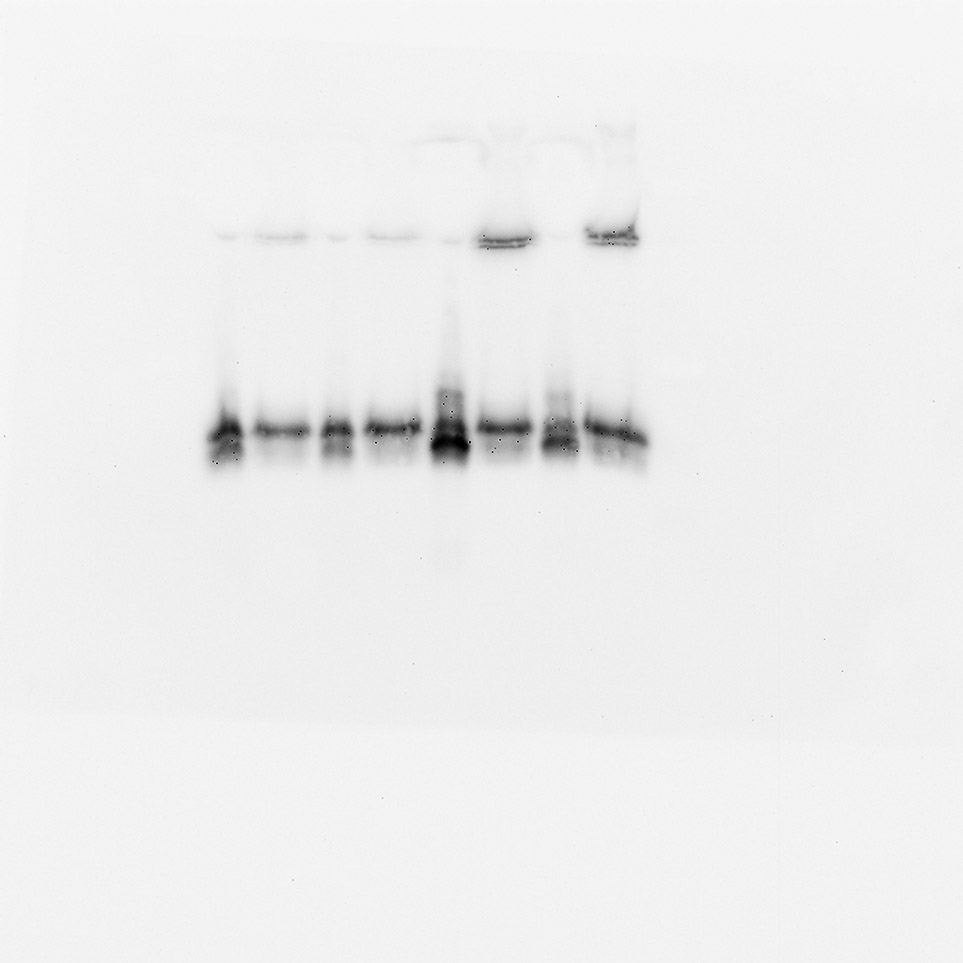

Supplement: Figure 1—source data 1. — A–D contain uncropped western blots shown in Figure 1A–D. Folders A’–D’ contain biological replicates of the respective experiments. Prizm files A’–D’ quantify relative Shh release rates based on the data shown in folders A’–D’. [file elife-86920-fig1-data1.zip › Figure_1_Source_Data_1 /A'_Fig_1_Quant_H7/14-11-23_Gel 4_antiShh Rabbit_1min.jpg]

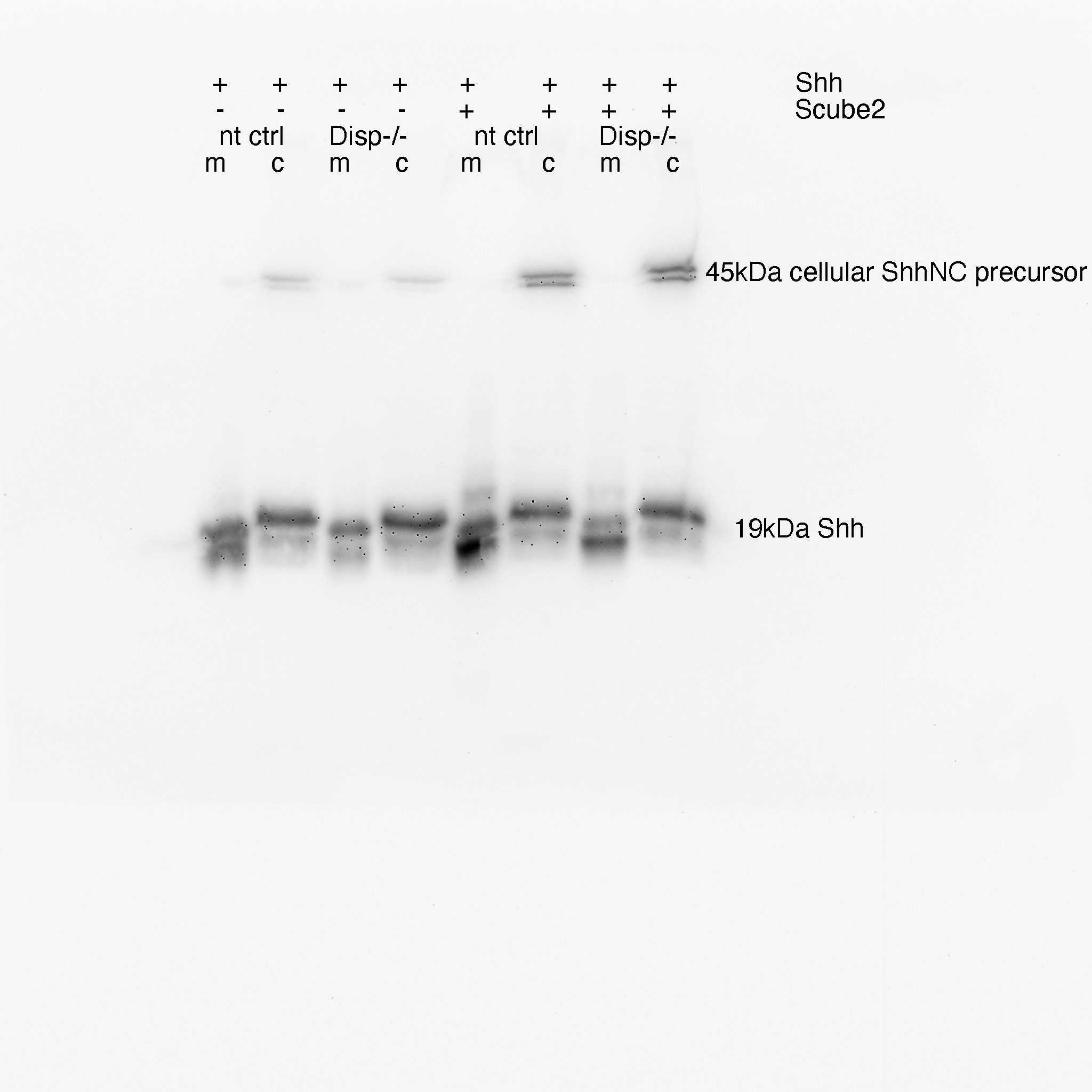

Supplement: Figure 1—source data 1. — A–D contain uncropped western blots shown in Figure 1A–D. Folders A’–D’ contain biological replicates of the respective experiments. Prizm files A’–D’ quantify relative Shh release rates based on the data shown in folders A’–D’. [file elife-86920-fig1-data1.zip › Figure_1_Source_Data_1 /A'_Fig_1_Quant_H7/16-11-23_Gel6_antiShh Rabbit_1min labelled.jpg]

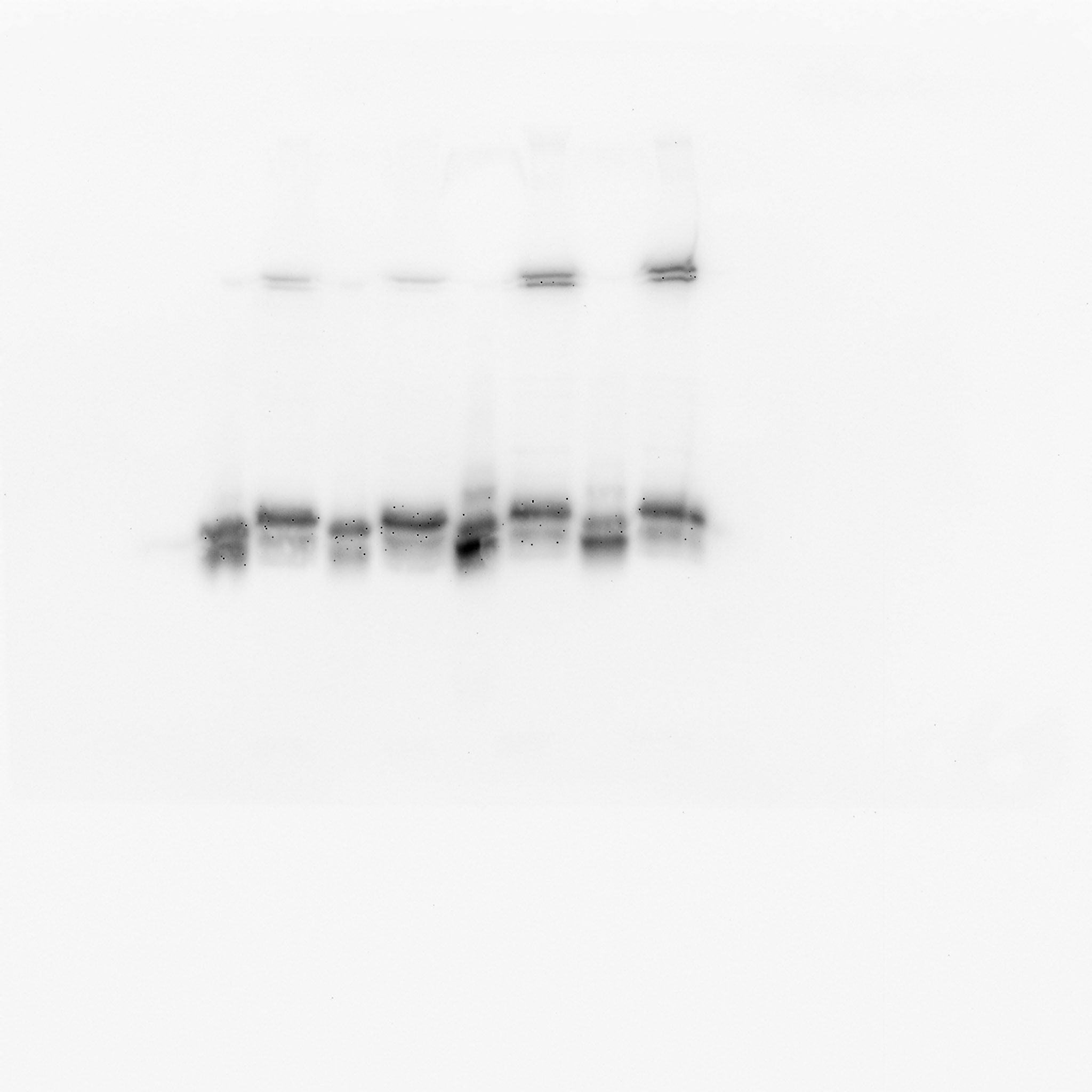

Supplement: Figure 1—source data 1. — A–D contain uncropped western blots shown in Figure 1A–D. Folders A’–D’ contain biological replicates of the respective experiments. Prizm files A’–D’ quantify relative Shh release rates based on the data shown in folders A’–D’. [file elife-86920-fig1-data1.zip › Figure_1_Source_Data_1 /A'_Fig_1_Quant_H7/16-11-23_Gel6_antiShh Rabbit_1min.jpg]

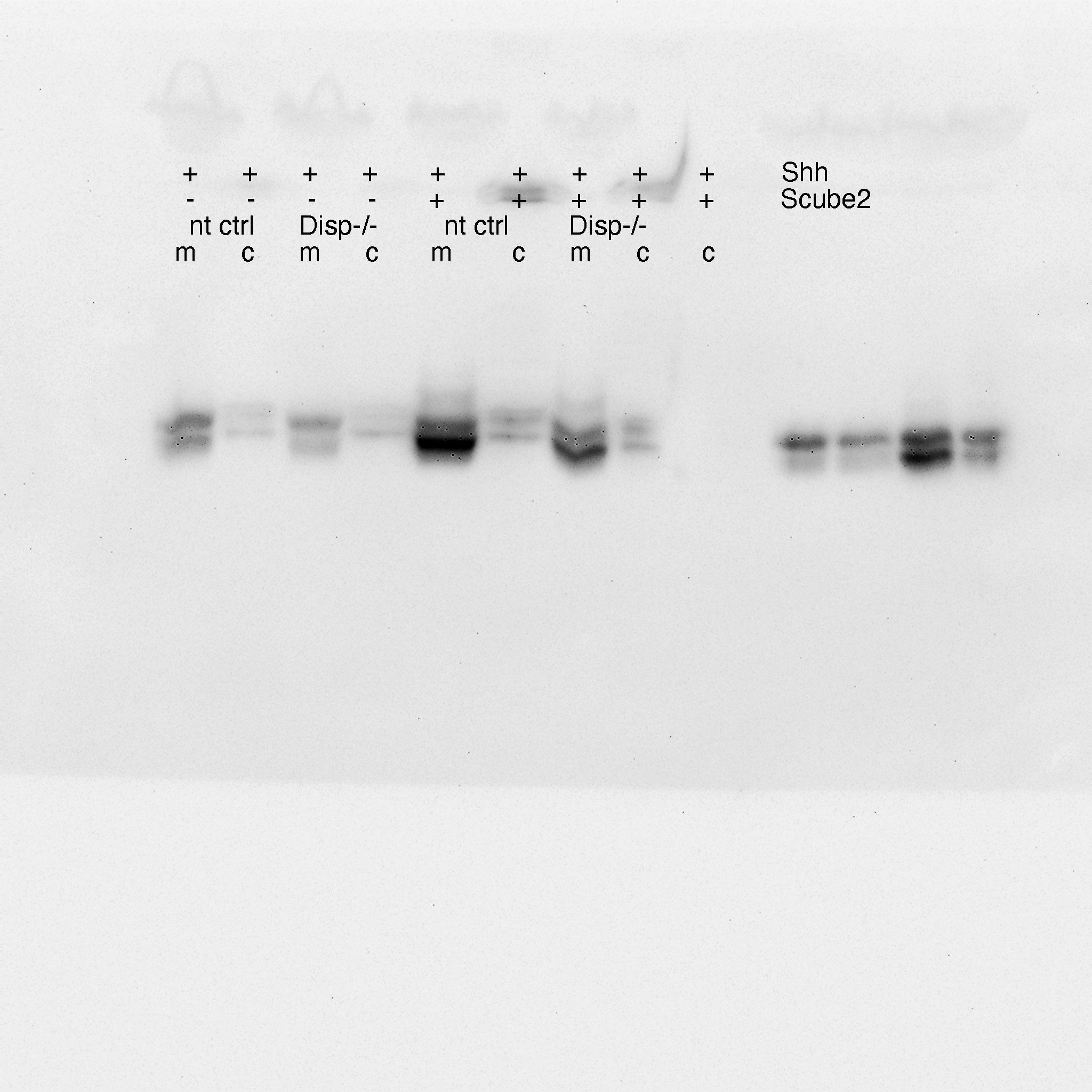

Supplement: Figure 1—source data 1. — A–D contain uncropped western blots shown in Figure 1A–D. Folders A’–D’ contain biological replicates of the respective experiments. Prizm files A’–D’ quantify relative Shh release rates based on the data shown in folders A’–D’. [file elife-86920-fig1-data1.zip › Figure_1_Source_Data_1 /A'_Fig_1_Quant_H7/20-01-22_Gel 2_antiShh Rabbit_3min labelled.jpg]

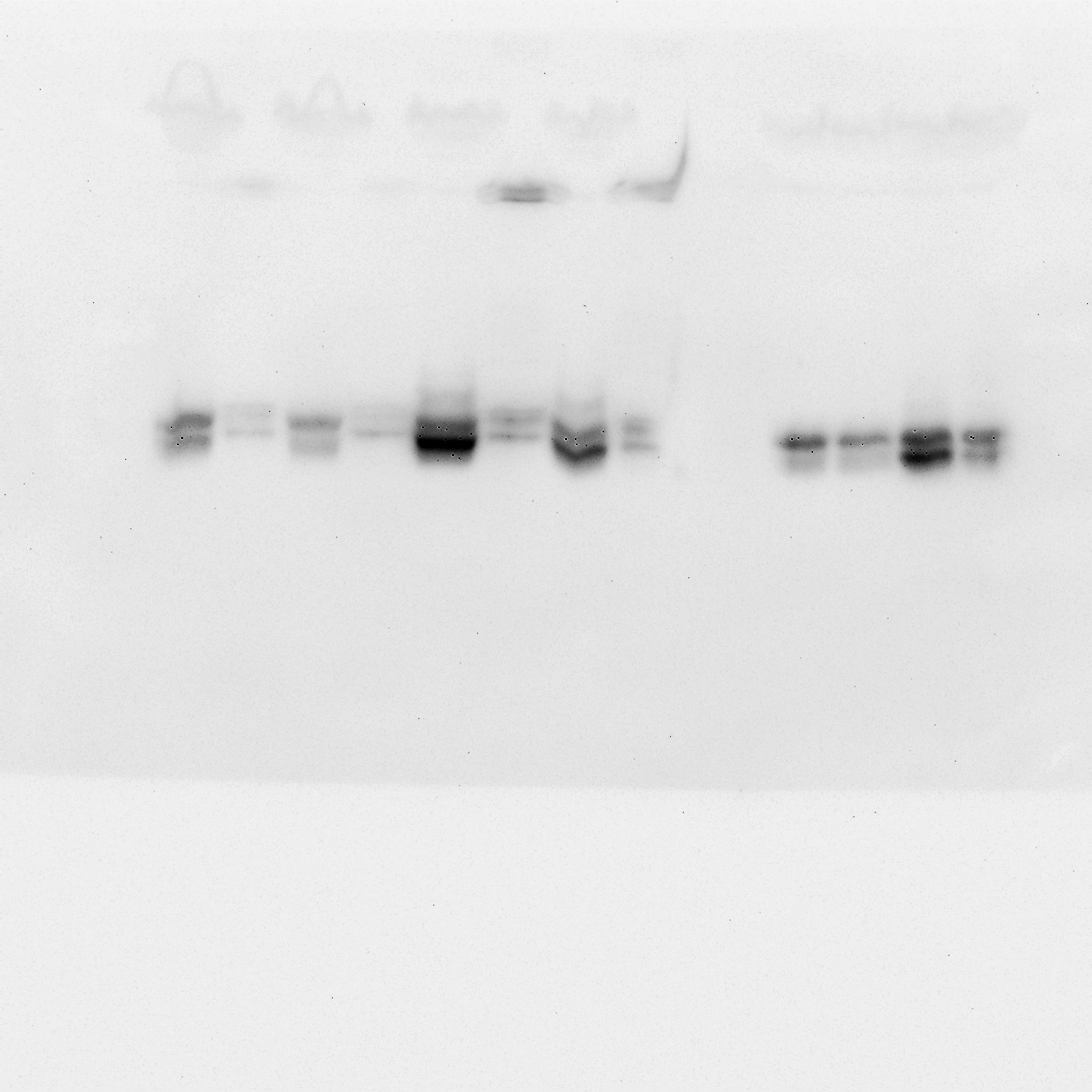

Supplement: Figure 1—source data 1. — A–D contain uncropped western blots shown in Figure 1A–D. Folders A’–D’ contain biological replicates of the respective experiments. Prizm files A’–D’ quantify relative Shh release rates based on the data shown in folders A’–D’. [file elife-86920-fig1-data1.zip › Figure_1_Source_Data_1 /A'_Fig_1_Quant_H7/20-01-22_Gel 2_antiShh Rabbit_3min.jpg]

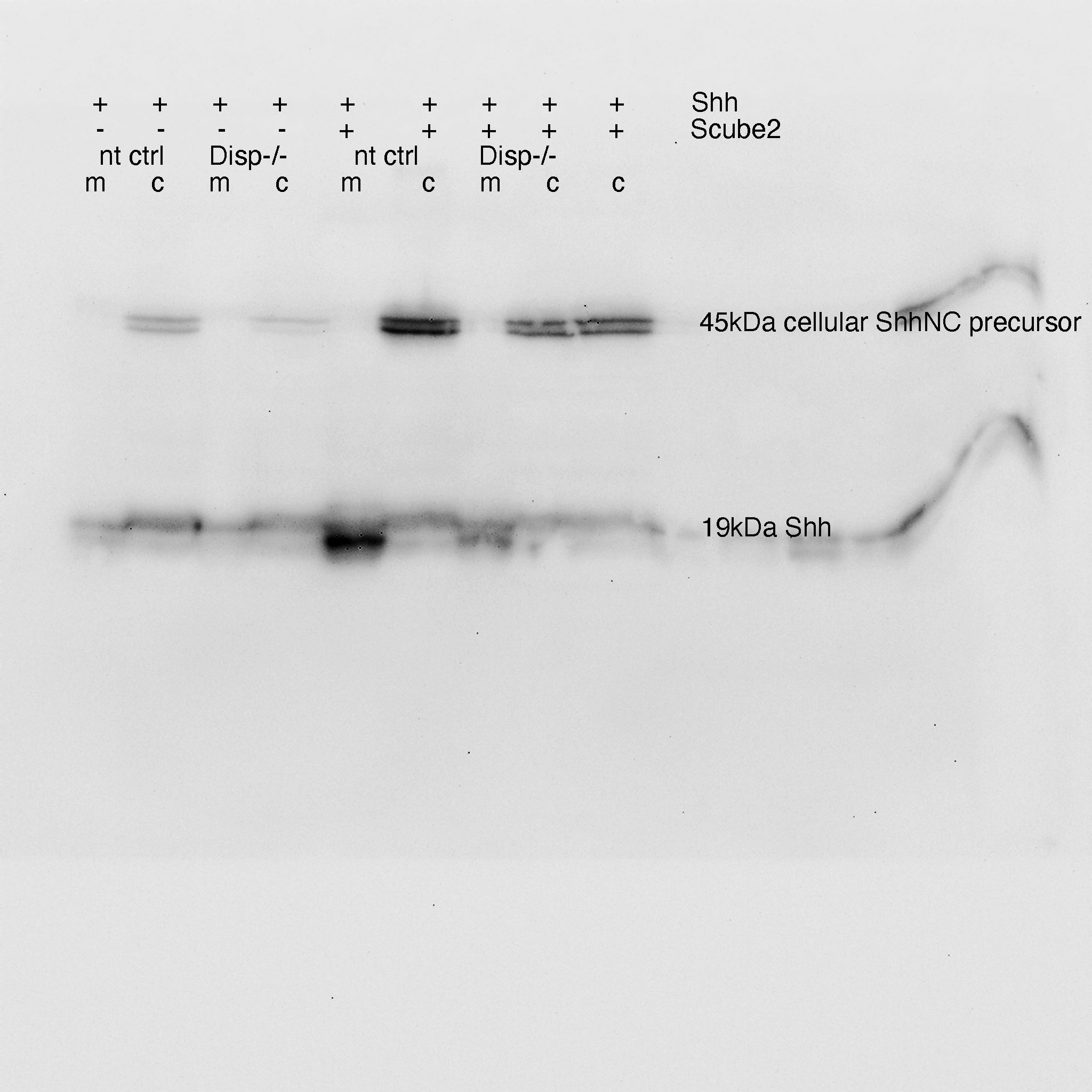

Supplement: Figure 1—source data 1. — A–D contain uncropped western blots shown in Figure 1A–D. Folders A’–D’ contain biological replicates of the respective experiments. Prizm files A’–D’ quantify relative Shh release rates based on the data shown in folders A’–D’. [file elife-86920-fig1-data1.zip › Figure_1_Source_Data_1 /A'_Fig_1_Quant_H7/V744_Gel1_antiShhrabbit_1min labelled.jpg]

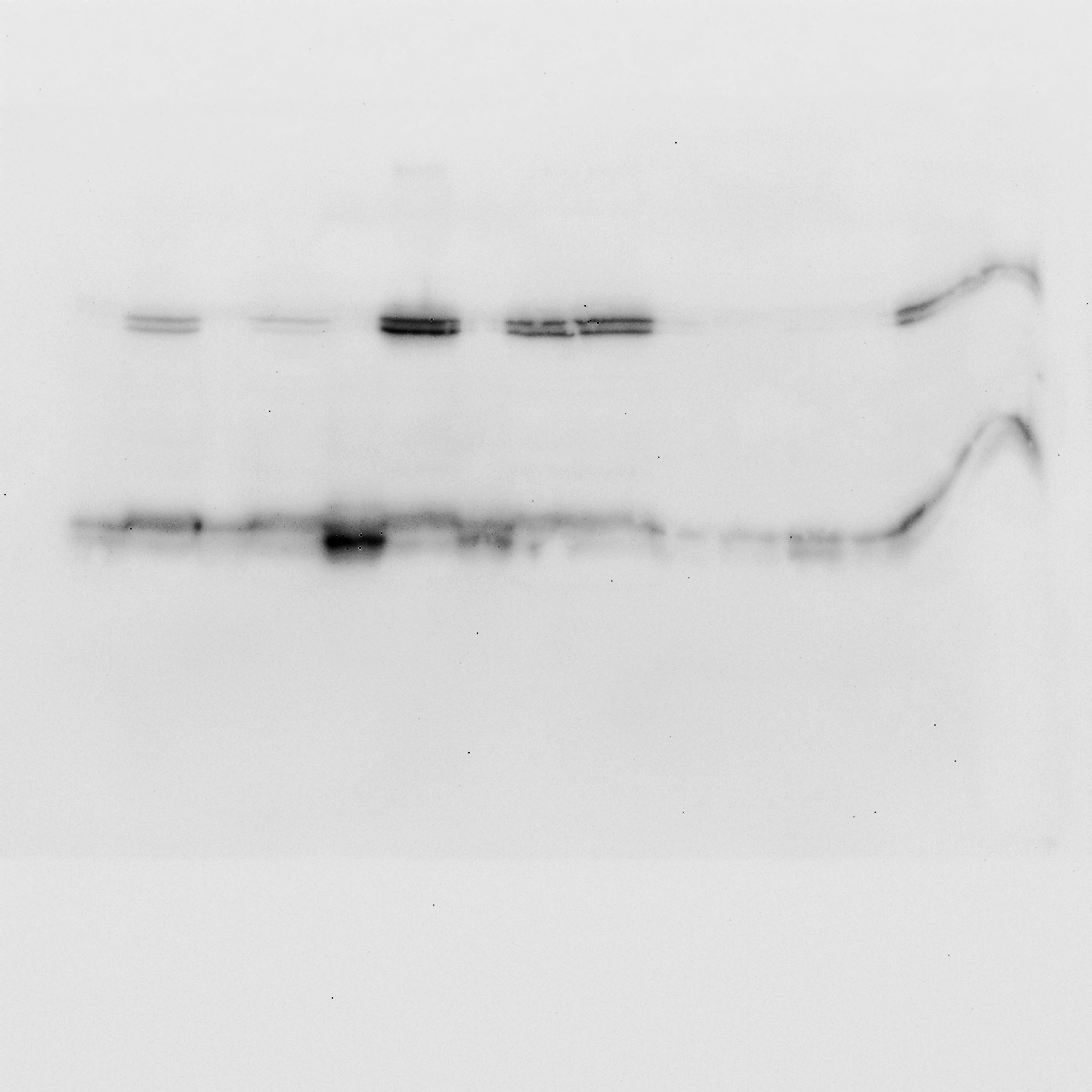

Supplement: Figure 1—source data 1. — A–D contain uncropped western blots shown in Figure 1A–D. Folders A’–D’ contain biological replicates of the respective experiments. Prizm files A’–D’ quantify relative Shh release rates based on the data shown in folders A’–D’. [file elife-86920-fig1-data1.zip › Figure_1_Source_Data_1 /A'_Fig_1_Quant_H7/V744_Gel1_antiShhrabbit_1min.jpg]

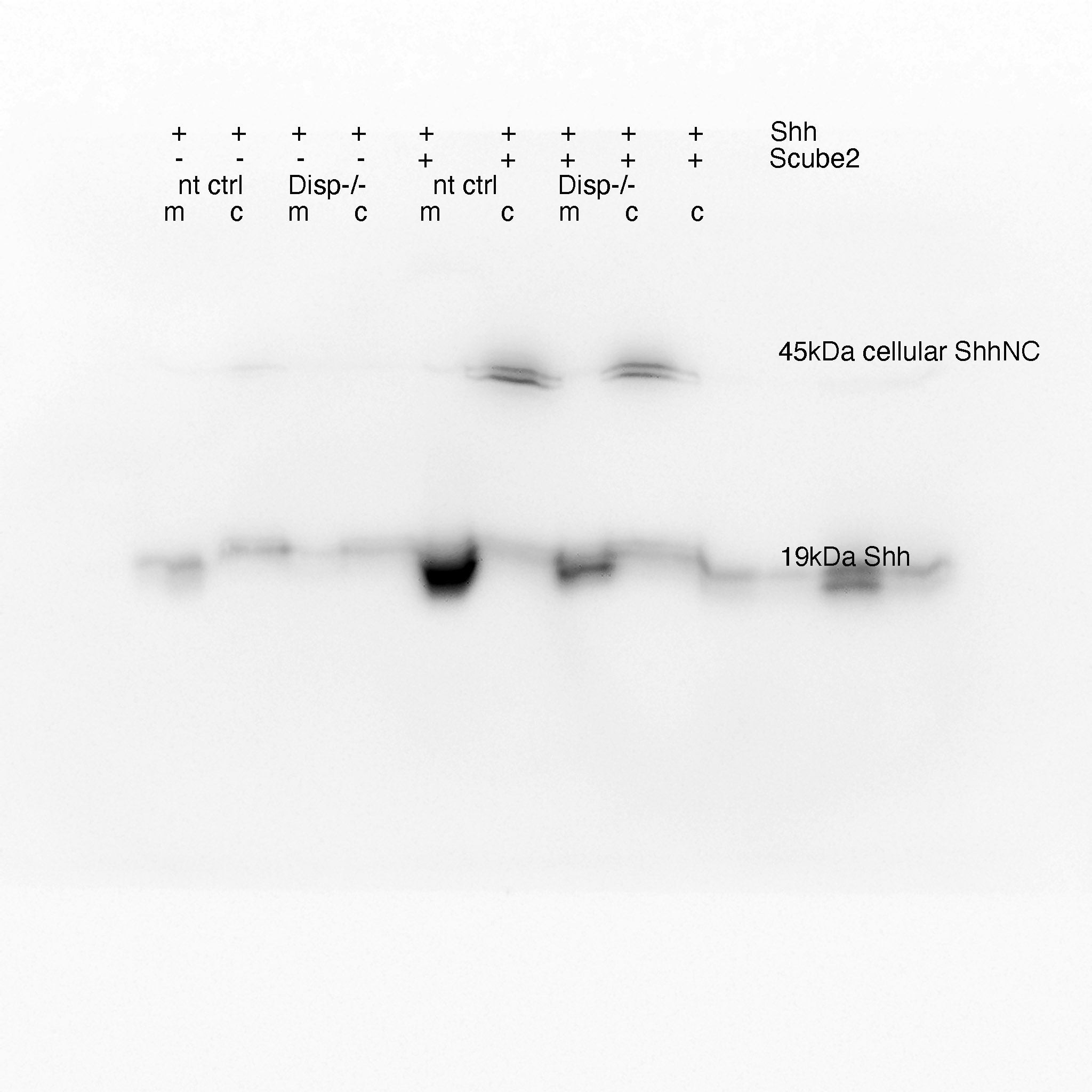

Supplement: Figure 1—source data 1. — A–D contain uncropped western blots shown in Figure 1A–D. Folders A’–D’ contain biological replicates of the respective experiments. Prizm files A’–D’ quantify relative Shh release rates based on the data shown in folders A’–D’. [file elife-86920-fig1-data1.zip › Figure_1_Source_Data_1 /A'_Fig_1_Quant_H7/VK193_20220209_Gel2_anti-Shh Rabitt_3min labelled.jpg]

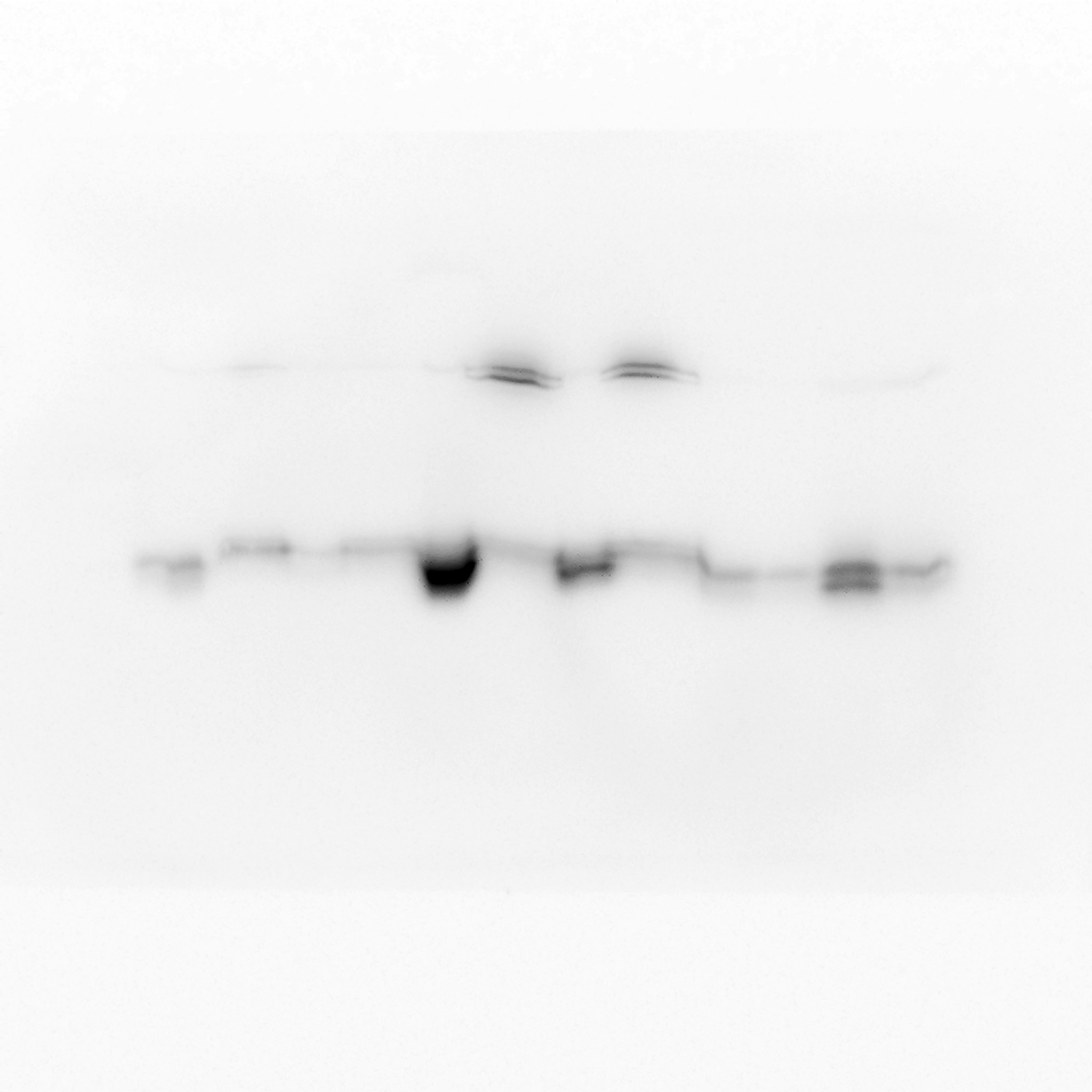

Supplement: Figure 1—source data 1. — A–D contain uncropped western blots shown in Figure 1A–D. Folders A’–D’ contain biological replicates of the respective experiments. Prizm files A’–D’ quantify relative Shh release rates based on the data shown in folders A’–D’. [file elife-86920-fig1-data1.zip › Figure_1_Source_Data_1 /A'_Fig_1_Quant_H7/VK193_20220209_Gel2_anti-Shh Rabitt_3min.jpg]

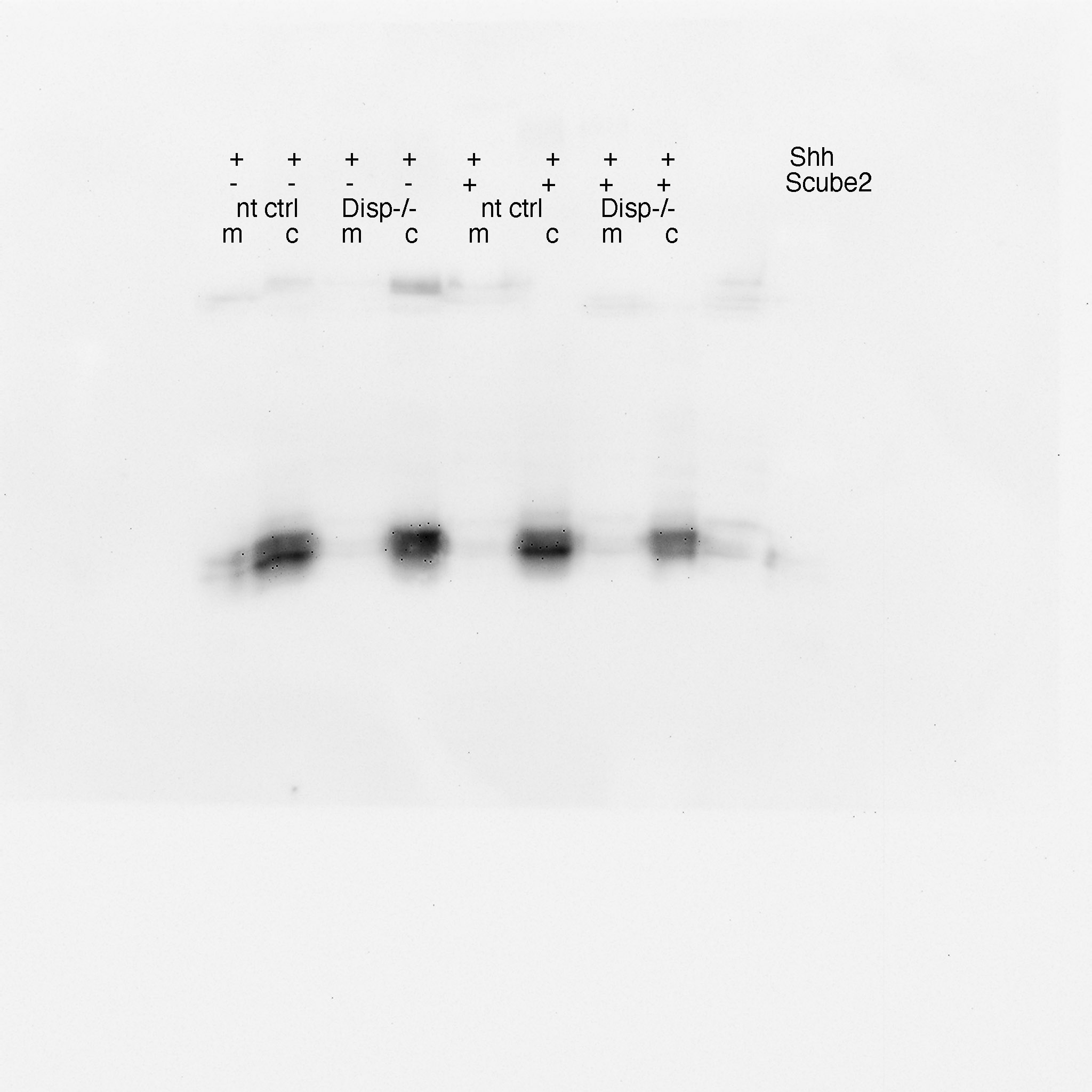

Supplement: Figure 1—source data 1. — A–D contain uncropped western blots shown in Figure 1A–D. Folders A’–D’ contain biological replicates of the respective experiments. Prizm files A’–D’ quantify relative Shh release rates based on the data shown in folders A’–D’. [file elife-86920-fig1-data1.zip › Figure_1_Source_Data_1 /B'_Fig_1_quant_268/24-02-15_16 Bit_V790 Gel 2_antiShh_1min49sec labelled.jpg]

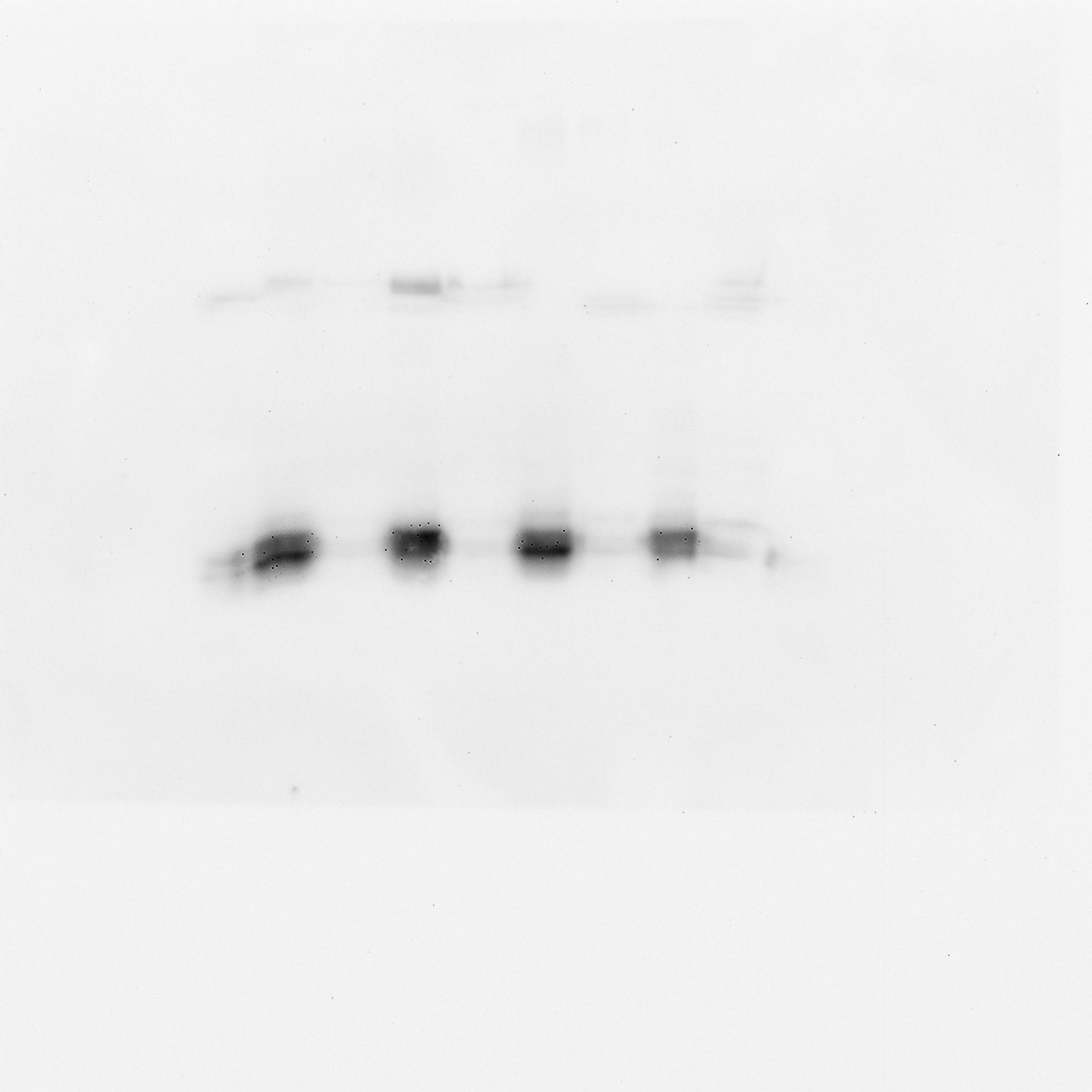

Supplement: Figure 1—source data 1. — A–D contain uncropped western blots shown in Figure 1A–D. Folders A’–D’ contain biological replicates of the respective experiments. Prizm files A’–D’ quantify relative Shh release rates based on the data shown in folders A’–D’. [file elife-86920-fig1-data1.zip › Figure_1_Source_Data_1 /B'_Fig_1_quant_268/24-02-15_16 Bit_V790 Gel 2_antiShh_1min49sec.jpg]

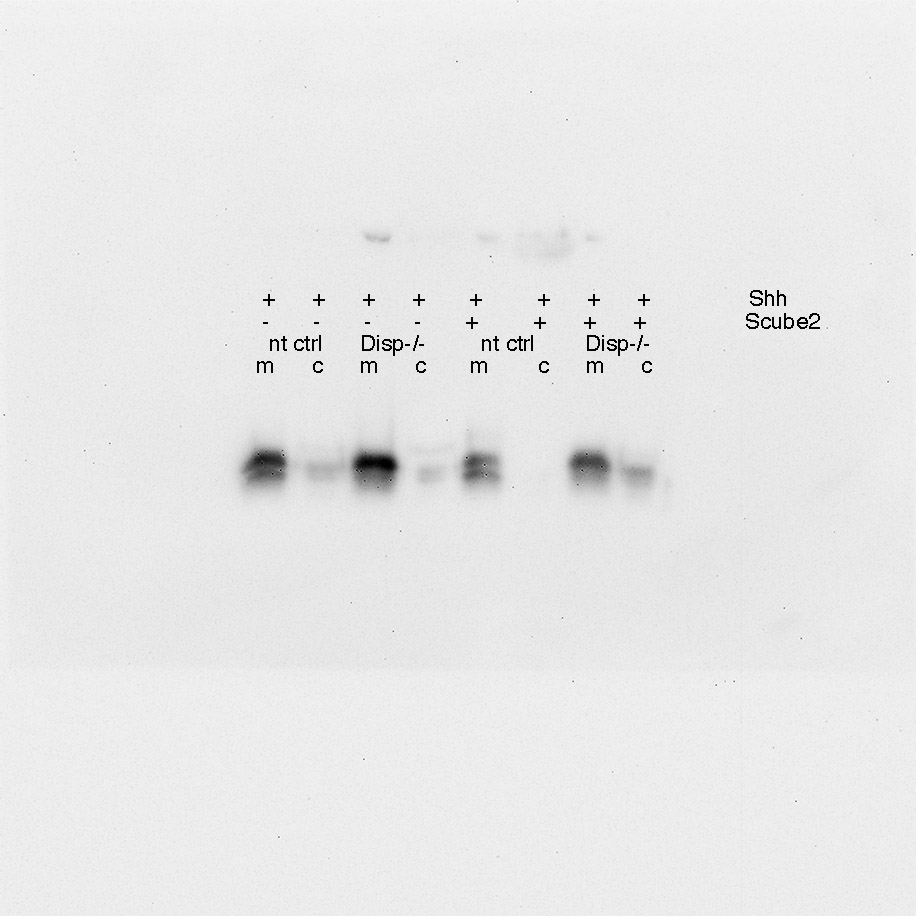

Supplement: Figure 1—source data 1. — A–D contain uncropped western blots shown in Figure 1A–D. Folders A’–D’ contain biological replicates of the respective experiments. Prizm files A’–D’ quantify relative Shh release rates based on the data shown in folders A’–D’. [file elife-86920-fig1-data1.zip › Figure_1_Source_Data_1 /B'_Fig_1_quant_268/24-02-21_16Bit_V793 Gel 2_antiShh_2min51sec labelled.jpg]

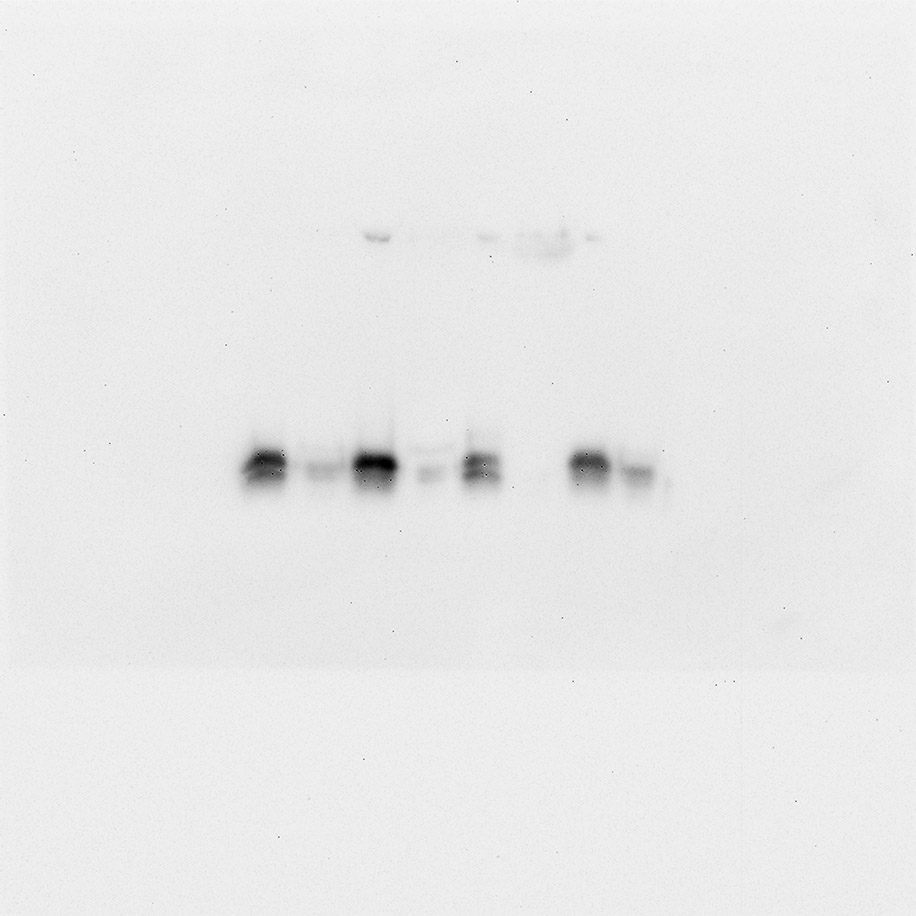

Supplement: Figure 1—source data 1. — A–D contain uncropped western blots shown in Figure 1A–D. Folders A’–D’ contain biological replicates of the respective experiments. Prizm files A’–D’ quantify relative Shh release rates based on the data shown in folders A’–D’. [file elife-86920-fig1-data1.zip › Figure_1_Source_Data_1 /B'_Fig_1_quant_268/24-02-21_16Bit_V793 Gel 2_antiShh_2min51sec.jpg]

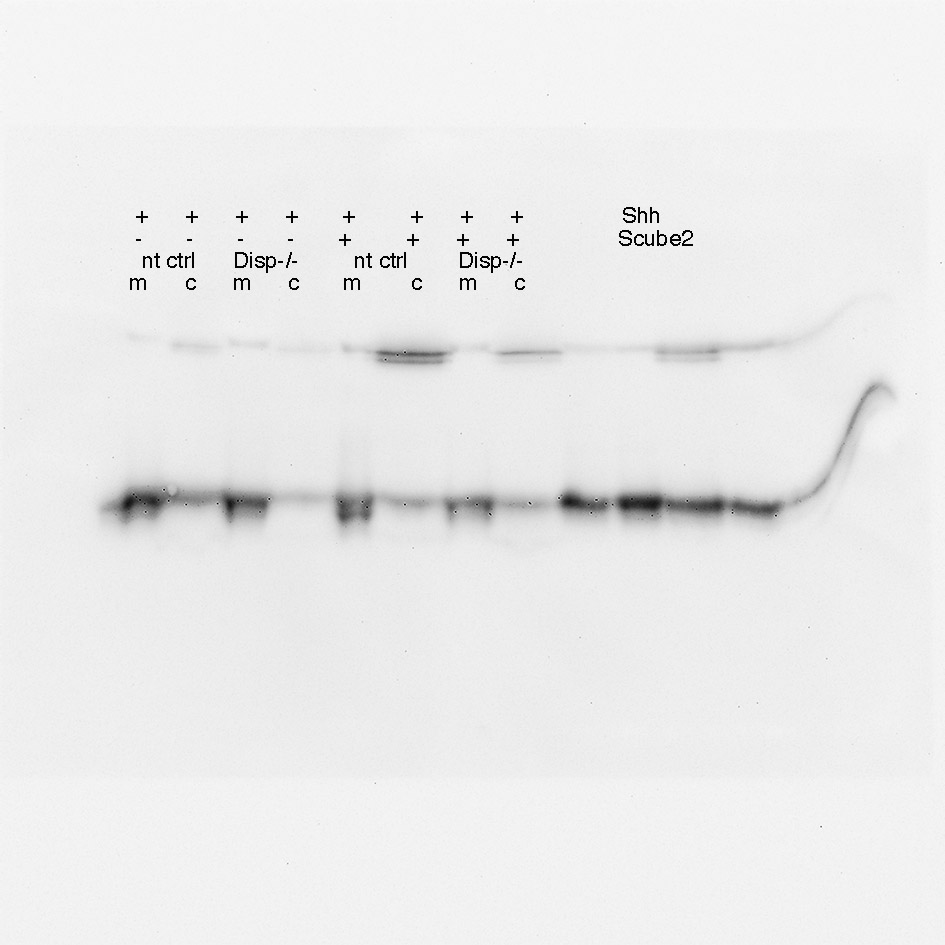

Supplement: Figure 1—source data 1. — A–D contain uncropped western blots shown in Figure 1A–D. Folders A’–D’ contain biological replicates of the respective experiments. Prizm files A’–D’ quantify relative Shh release rates based on the data shown in folders A’–D’. [file elife-86920-fig1-data1.zip › Figure_1_Source_Data_1 /B'_Fig_1_quant_268/V749_2_antiShhrabbit_2min labelled.jpg]

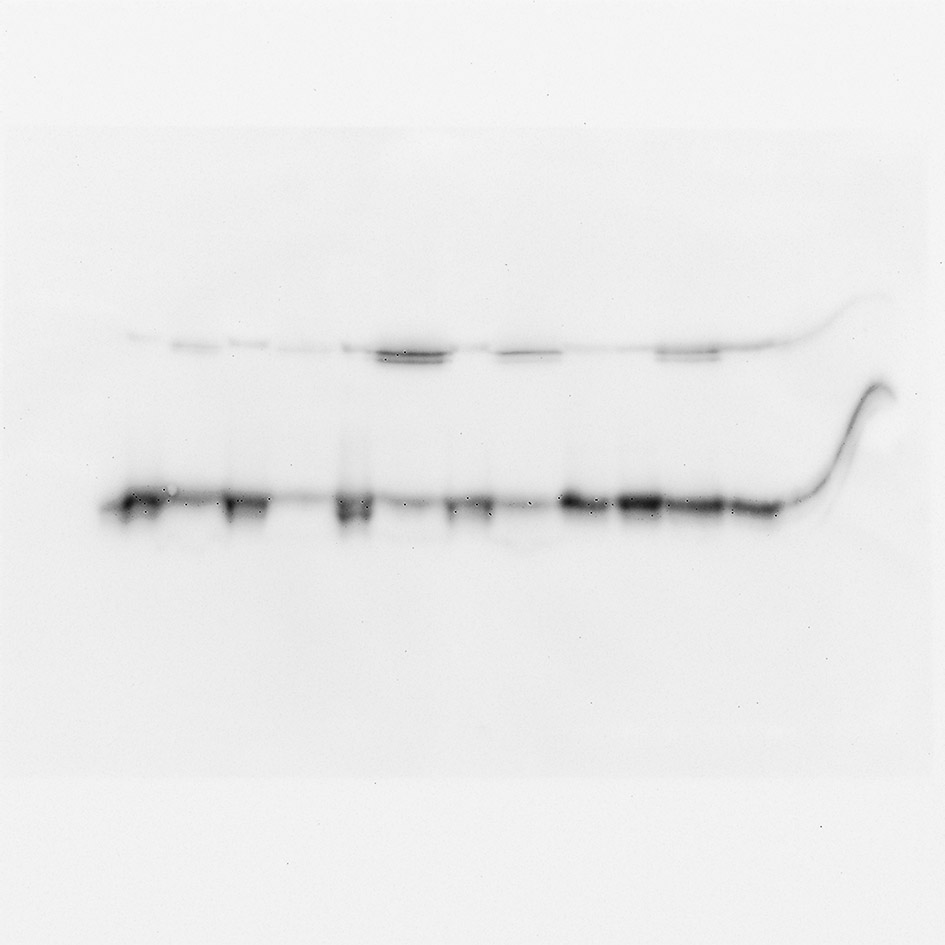

Supplement: Figure 1—source data 1. — A–D contain uncropped western blots shown in Figure 1A–D. Folders A’–D’ contain biological replicates of the respective experiments. Prizm files A’–D’ quantify relative Shh release rates based on the data shown in folders A’–D’. [file elife-86920-fig1-data1.zip › Figure_1_Source_Data_1 /B'_Fig_1_quant_268/V749_2_antiShhrabbit_2min.jpg]

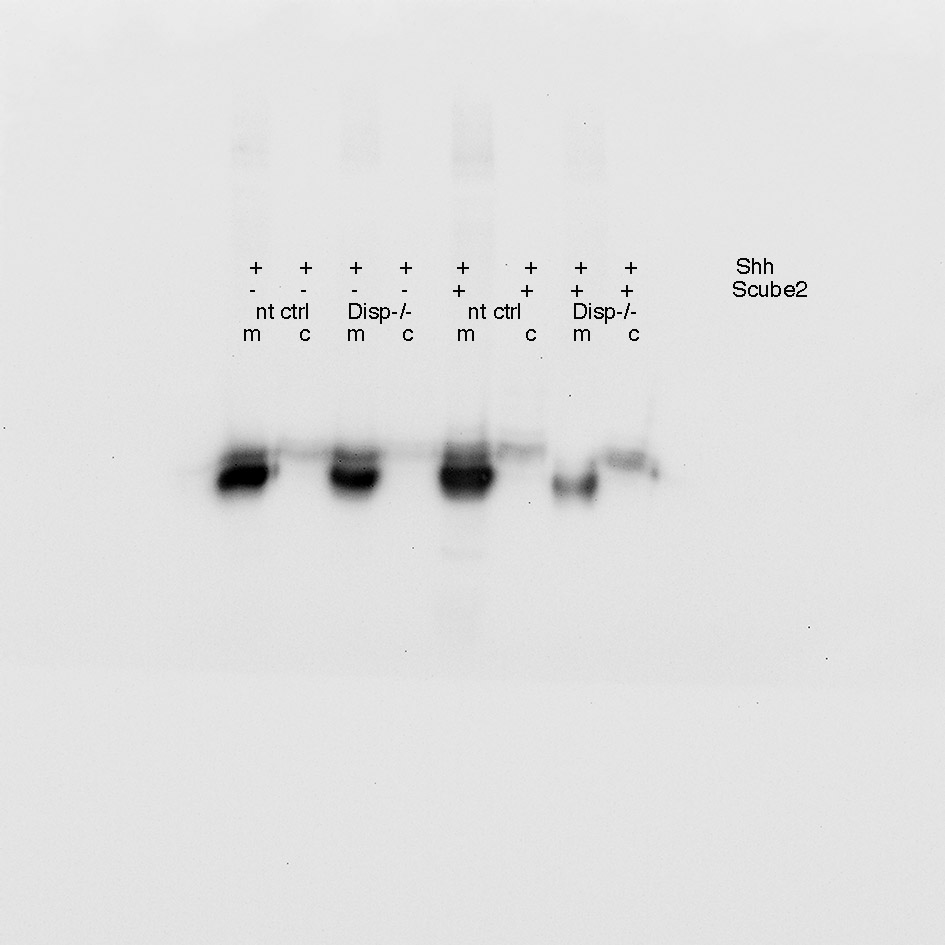

Supplement: Figure 1—source data 1. — A–D contain uncropped western blots shown in Figure 1A–D. Folders A’–D’ contain biological replicates of the respective experiments. Prizm files A’–D’ quantify relative Shh release rates based on the data shown in folders A’–D’. [file elife-86920-fig1-data1.zip › Figure_1_Source_Data_1 /C'_Fig_1_quant_H18/24-02-15_V790 Gel 1_antiShh_20sec labelled.jpg]

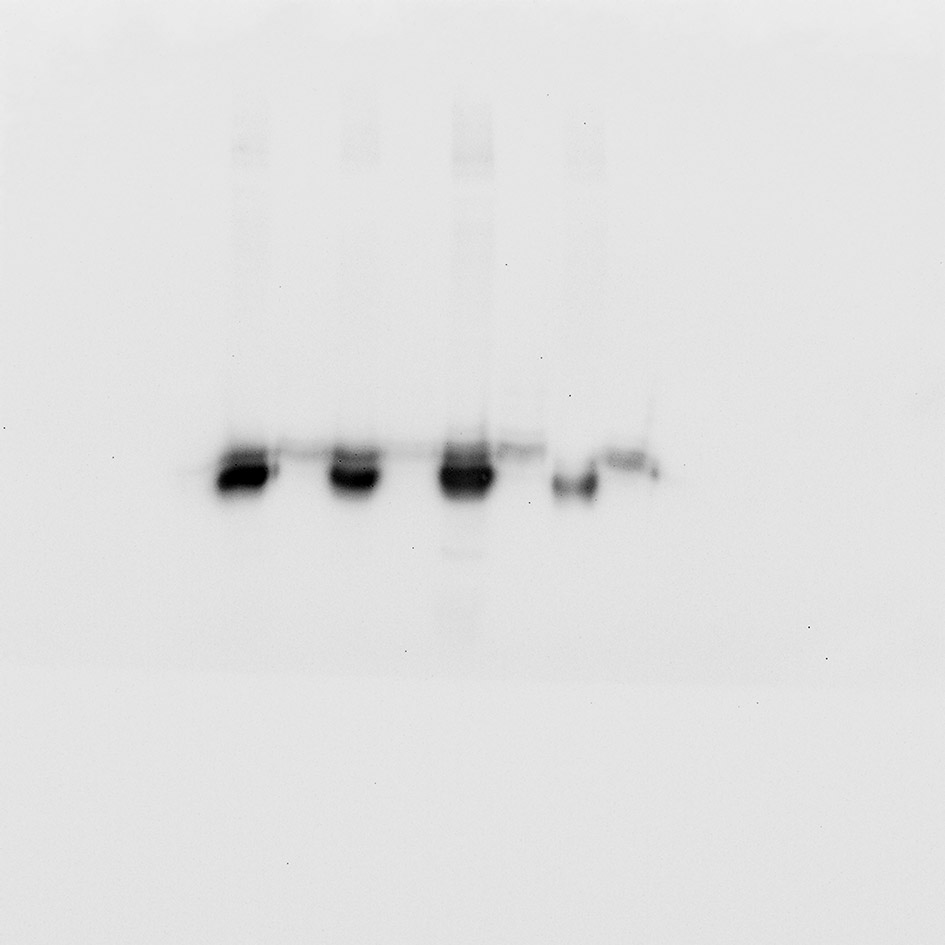

Supplement: Figure 1—source data 1. — A–D contain uncropped western blots shown in Figure 1A–D. Folders A’–D’ contain biological replicates of the respective experiments. Prizm files A’–D’ quantify relative Shh release rates based on the data shown in folders A’–D’. [file elife-86920-fig1-data1.zip › Figure_1_Source_Data_1 /C'_Fig_1_quant_H18/24-02-15_V790 Gel 1_antiShh_20sec.jpg]

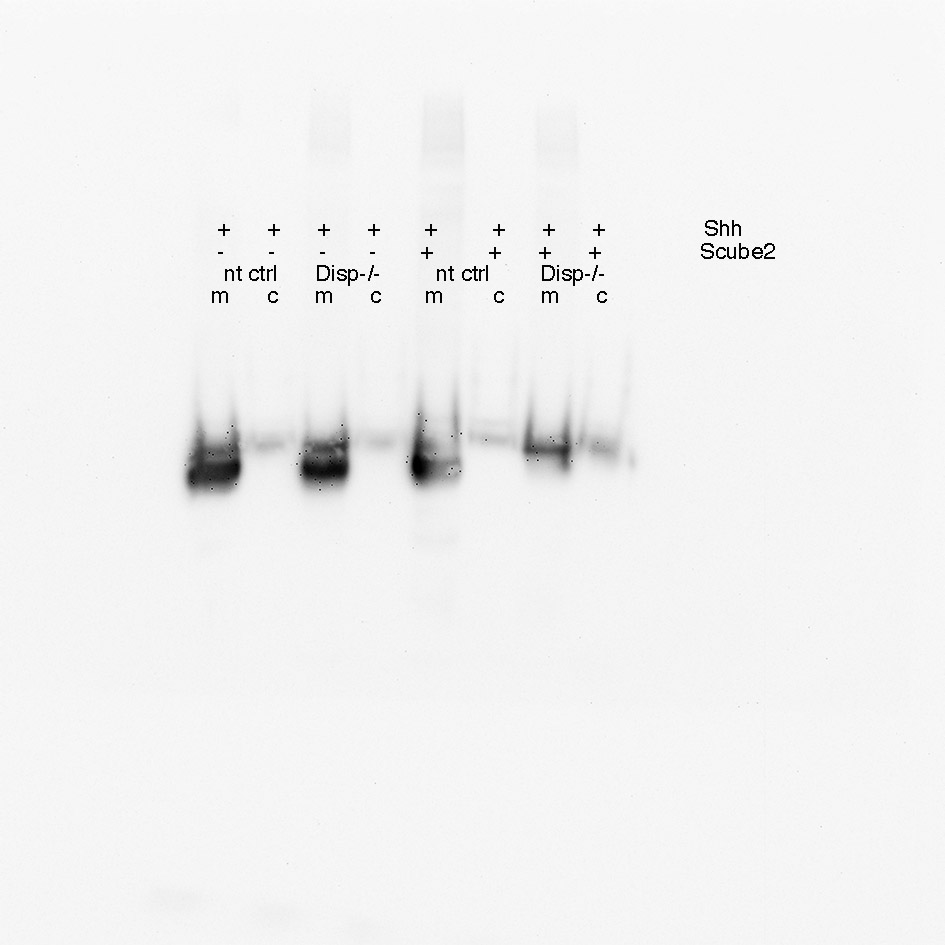

Supplement: Figure 1—source data 1. — A–D contain uncropped western blots shown in Figure 1A–D. Folders A’–D’ contain biological replicates of the respective experiments. Prizm files A’–D’ quantify relative Shh release rates based on the data shown in folders A’–D’. [file elife-86920-fig1-data1.zip › Figure_1_Source_Data_1 /C'_Fig_1_quant_H18/24-02-15_V790 Gel 4_antiShh_2min labelled.jpg]

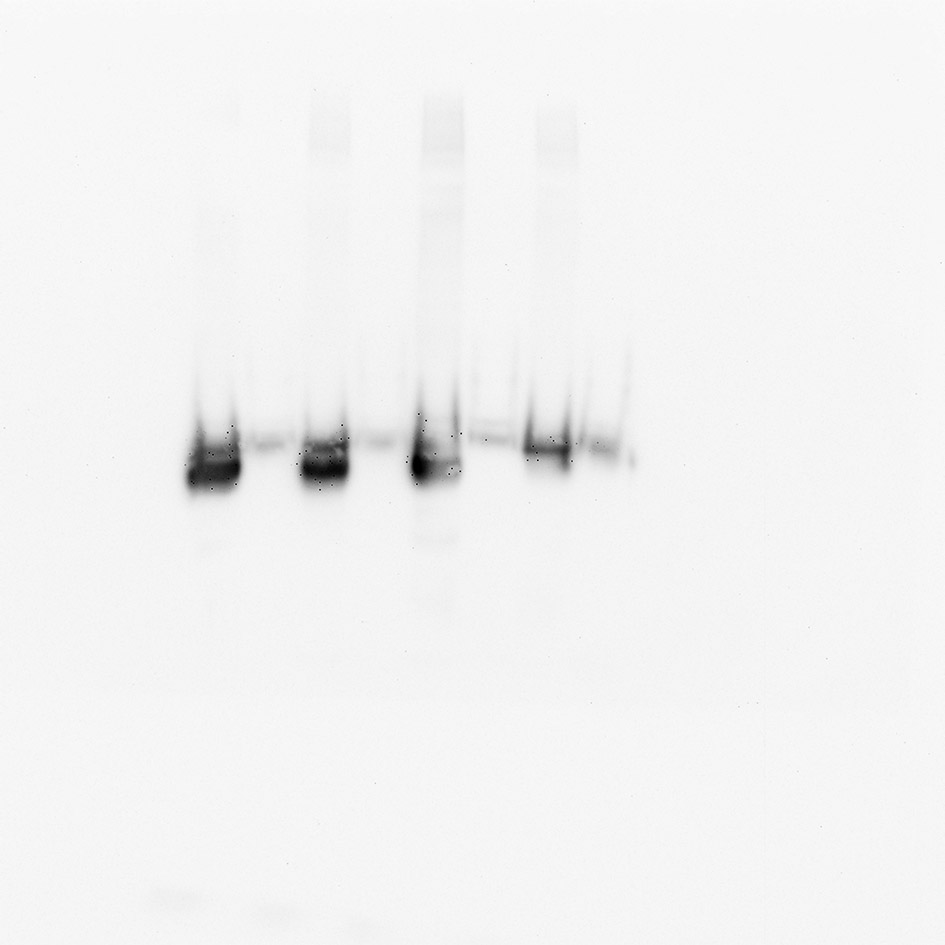

Supplement: Figure 1—source data 1. — A–D contain uncropped western blots shown in Figure 1A–D. Folders A’–D’ contain biological replicates of the respective experiments. Prizm files A’–D’ quantify relative Shh release rates based on the data shown in folders A’–D’. [file elife-86920-fig1-data1.zip › Figure_1_Source_Data_1 /C'_Fig_1_quant_H18/24-02-15_V790 Gel 4_antiShh_2min.jpg]

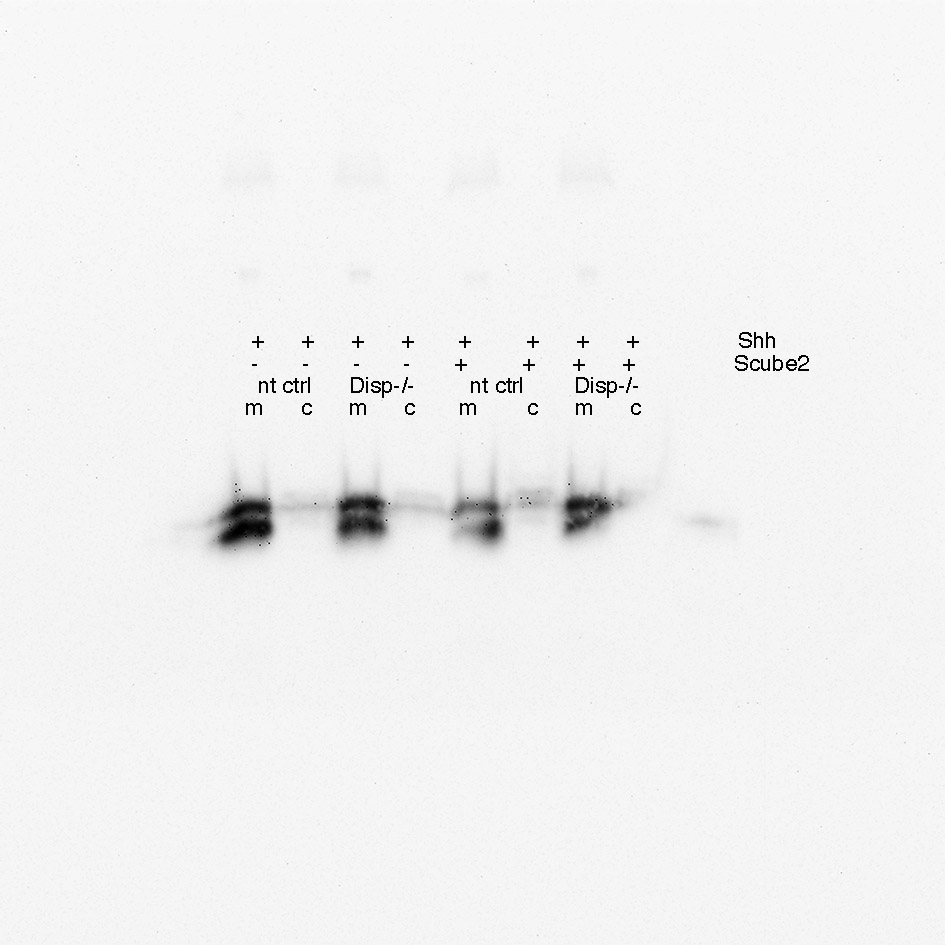

Supplement: Figure 1—source data 1. — A–D contain uncropped western blots shown in Figure 1A–D. Folders A’–D’ contain biological replicates of the respective experiments. Prizm files A’–D’ quantify relative Shh release rates based on the data shown in folders A’–D’. [file elife-86920-fig1-data1.zip › Figure_1_Source_Data_1 /C'_Fig_1_quant_H18/24-02-21_V793 Gel 5_antiShh_5min labelled.jpg]

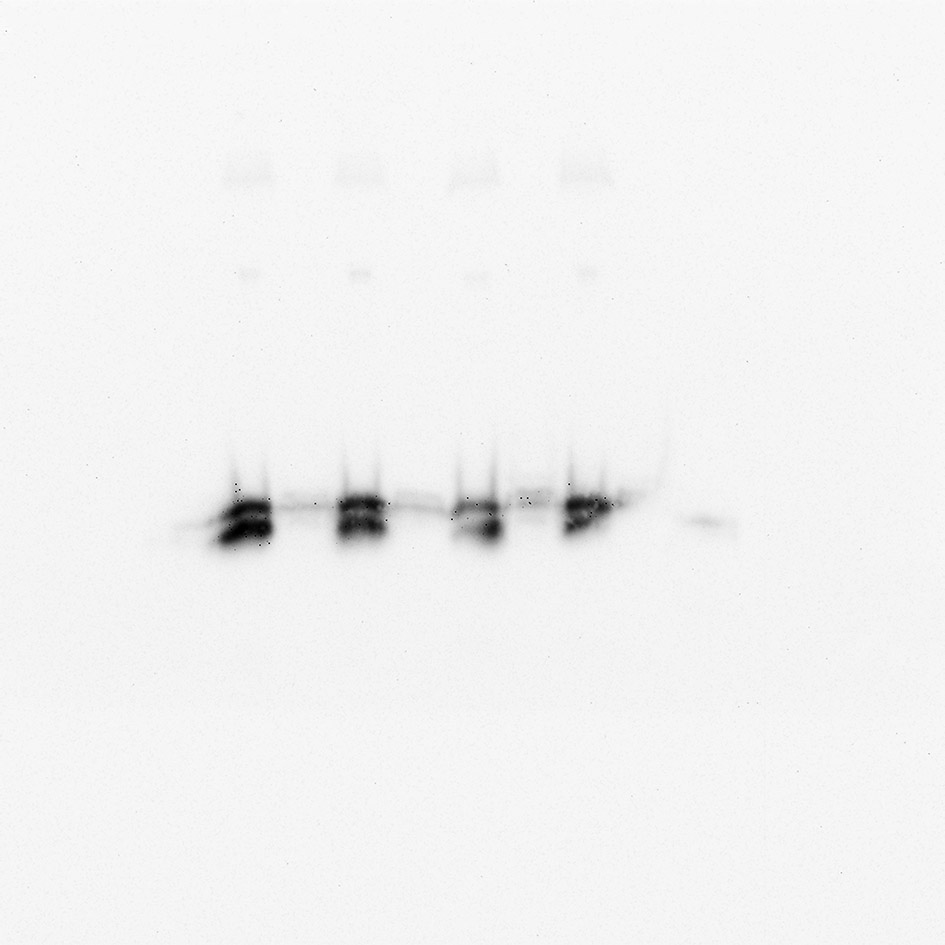

Supplement: Figure 1—source data 1. — A–D contain uncropped western blots shown in Figure 1A–D. Folders A’–D’ contain biological replicates of the respective experiments. Prizm files A’–D’ quantify relative Shh release rates based on the data shown in folders A’–D’. [file elife-86920-fig1-data1.zip › Figure_1_Source_Data_1 /C'_Fig_1_quant_H18/24-02-21_V793 Gel 5_antiShh_5min.jpg]

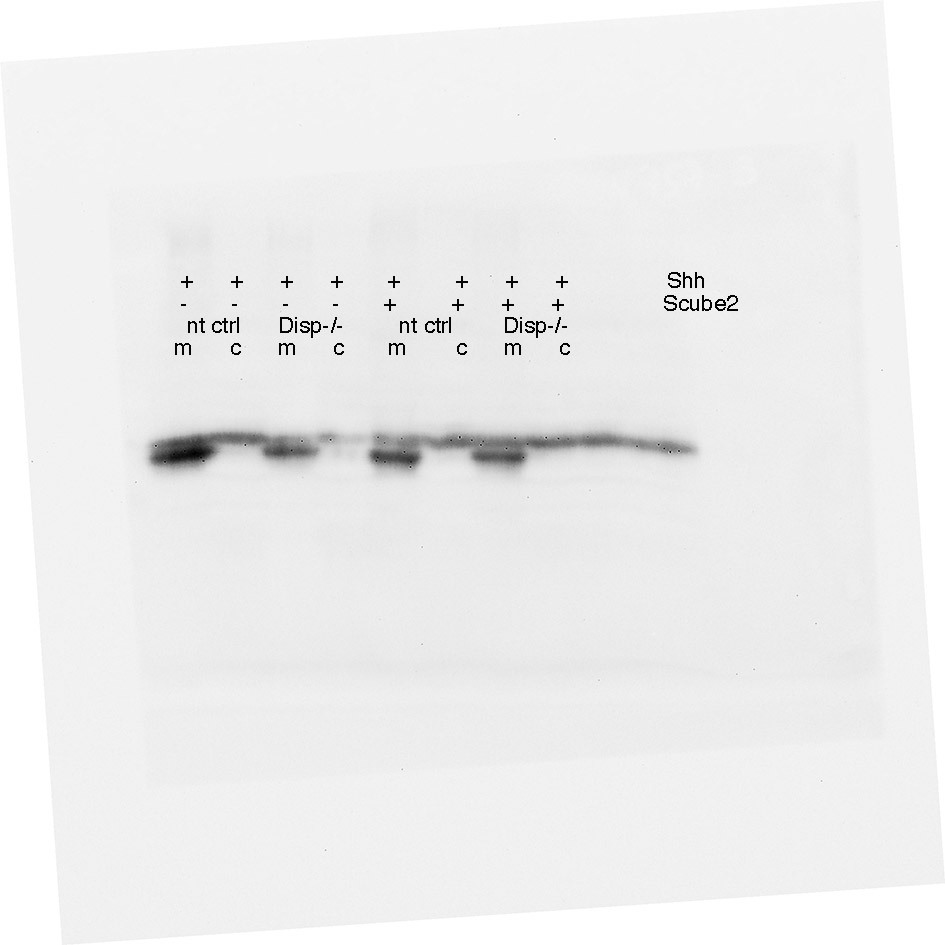

Supplement: Figure 1—source data 1. — A–D contain uncropped western blots shown in Figure 1A–D. Folders A’–D’ contain biological replicates of the respective experiments. Prizm files A’–D’ quantify relative Shh release rates based on the data shown in folders A’–D’. [file elife-86920-fig1-data1.zip › Figure_1_Source_Data_1 /C'_Fig_1_quant_H18/V757_3_0.5min_antiShh labelled.jpg]

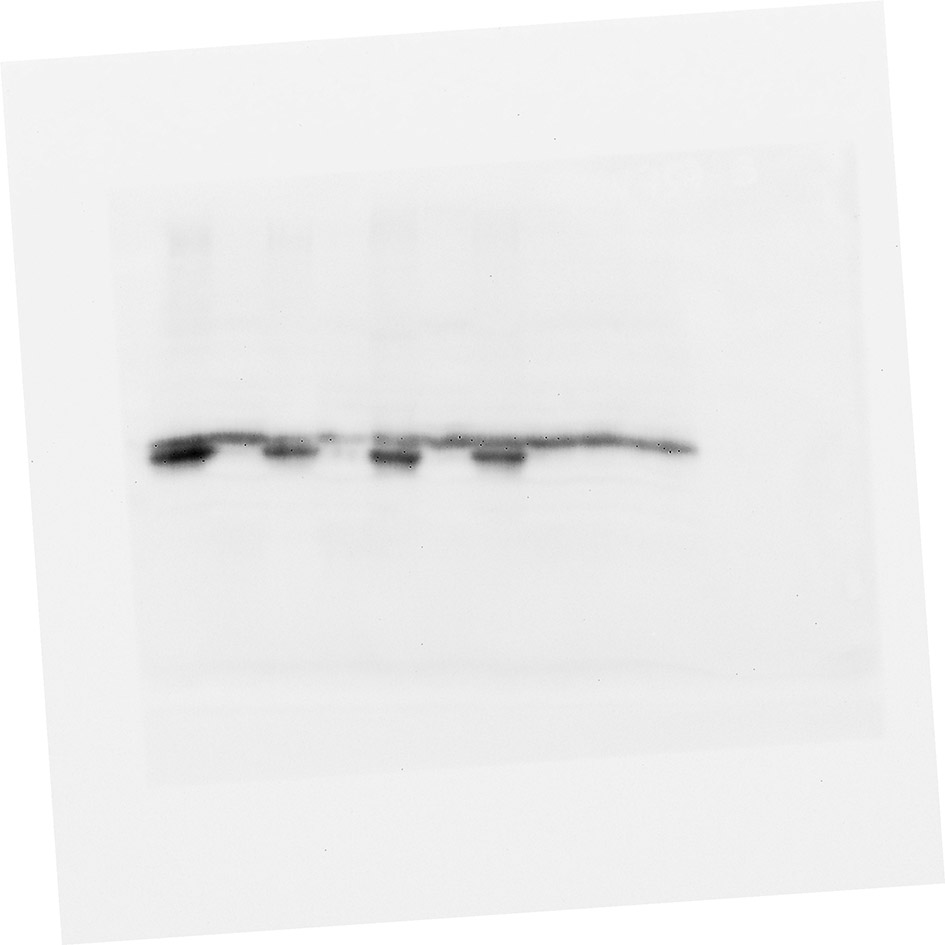

Supplement: Figure 1—source data 1. — A–D contain uncropped western blots shown in Figure 1A–D. Folders A’–D’ contain biological replicates of the respective experiments. Prizm files A’–D’ quantify relative Shh release rates based on the data shown in folders A’–D’. [file elife-86920-fig1-data1.zip › Figure_1_Source_Data_1 /C'_Fig_1_quant_H18/V757_3_0.5min_antiShh.jpg]

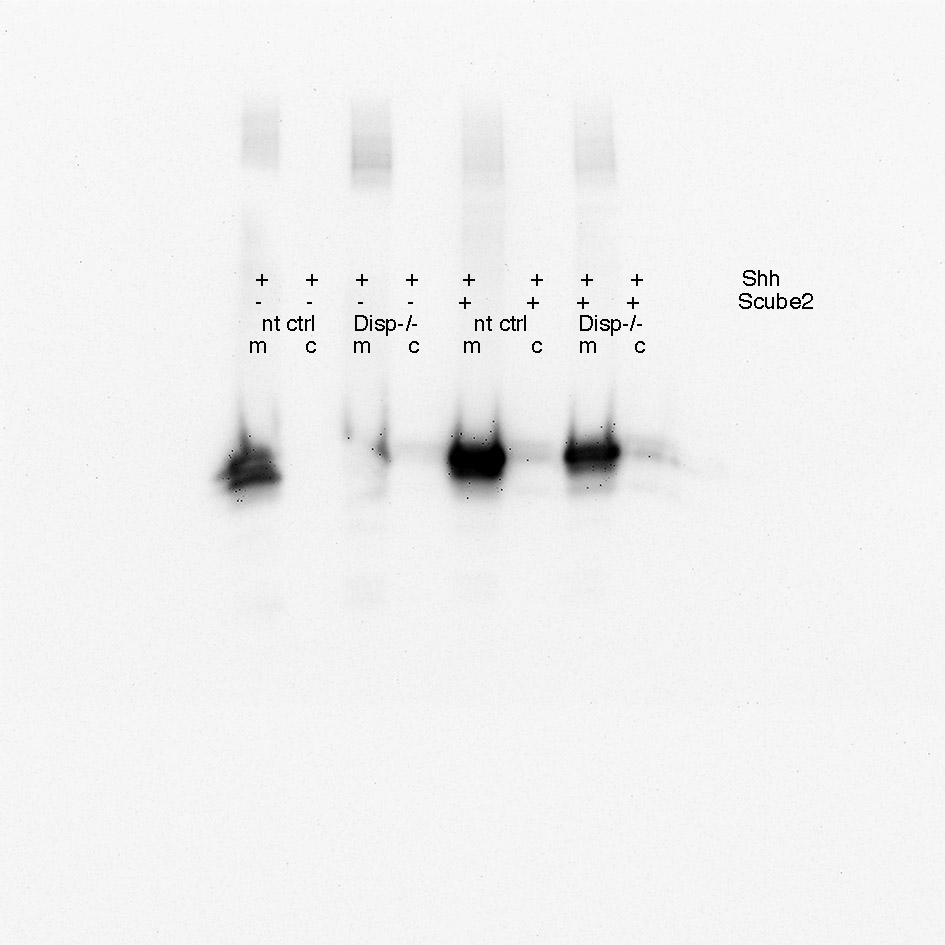

Supplement: Figure 1—source data 1. — A–D contain uncropped western blots shown in Figure 1A–D. Folders A’–D’ contain biological replicates of the respective experiments. Prizm files A’–D’ quantify relative Shh release rates based on the data shown in folders A’–D’. [file elife-86920-fig1-data1.zip › Figure_1_Source_Data_1 /D'_Fig_1_quant_242/24-02-15_16 Bit_Kay V 790 Gel 6_antiShh_5min labelled.jpg]

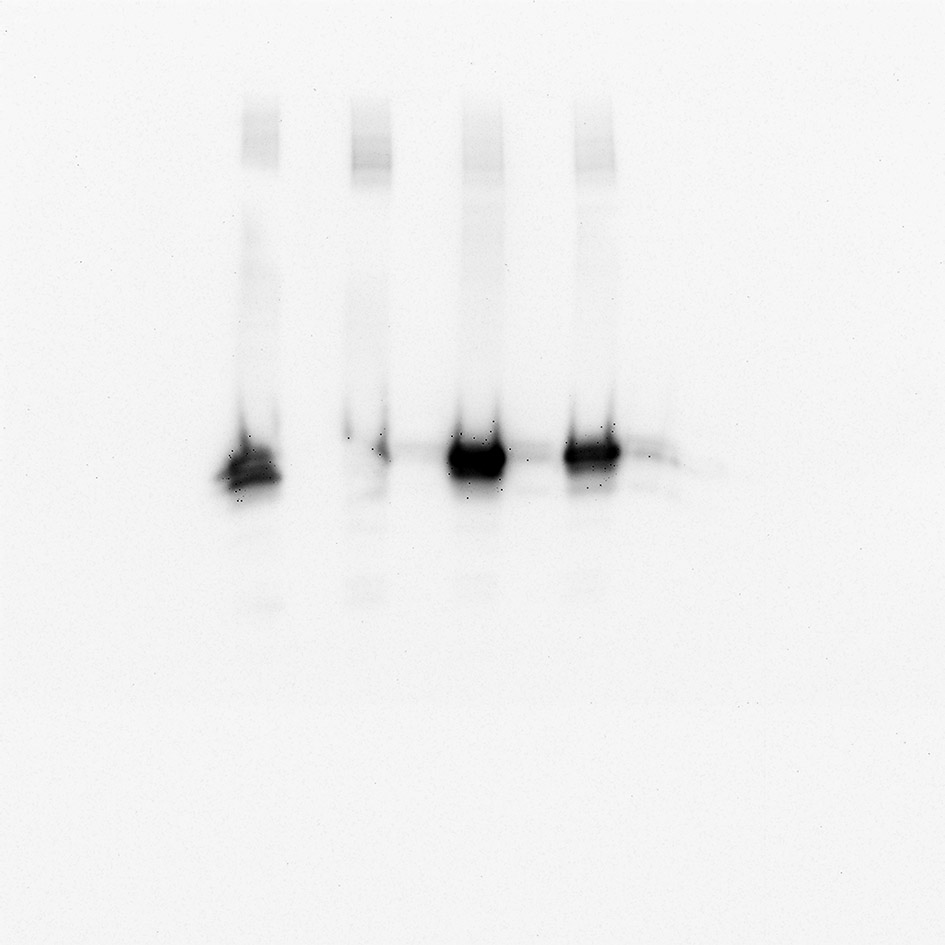

Supplement: Figure 1—source data 1. — A–D contain uncropped western blots shown in Figure 1A–D. Folders A’–D’ contain biological replicates of the respective experiments. Prizm files A’–D’ quantify relative Shh release rates based on the data shown in folders A’–D’. [file elife-86920-fig1-data1.zip › Figure_1_Source_Data_1 /D'_Fig_1_quant_242/24-02-15_16 Bit_Kay V 790 Gel 6_antiShh_5min.jpg]

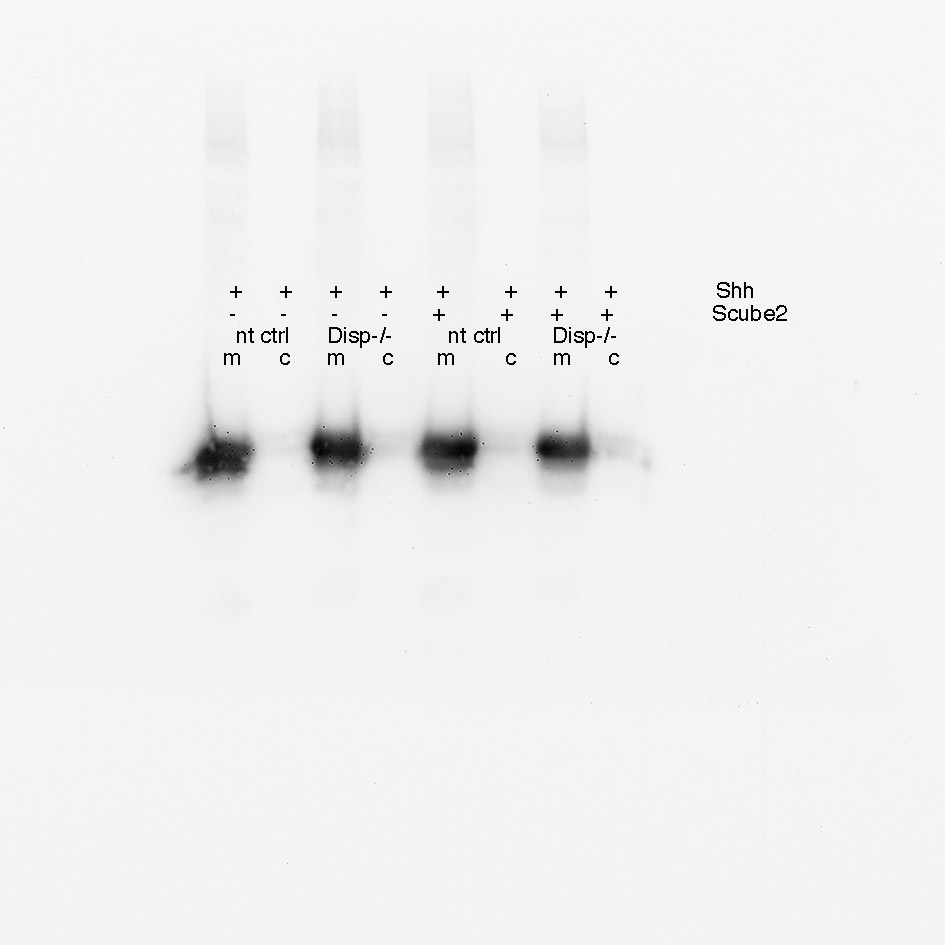

Supplement: Figure 1—source data 1. — A–D contain uncropped western blots shown in Figure 1A–D. Folders A’–D’ contain biological replicates of the respective experiments. Prizm files A’–D’ quantify relative Shh release rates based on the data shown in folders A’–D’. [file elife-86920-fig1-data1.zip › Figure_1_Source_Data_1 /D'_Fig_1_quant_242/24-02-15_V790_Gel 3_antiShh_1min labelled.jpg]

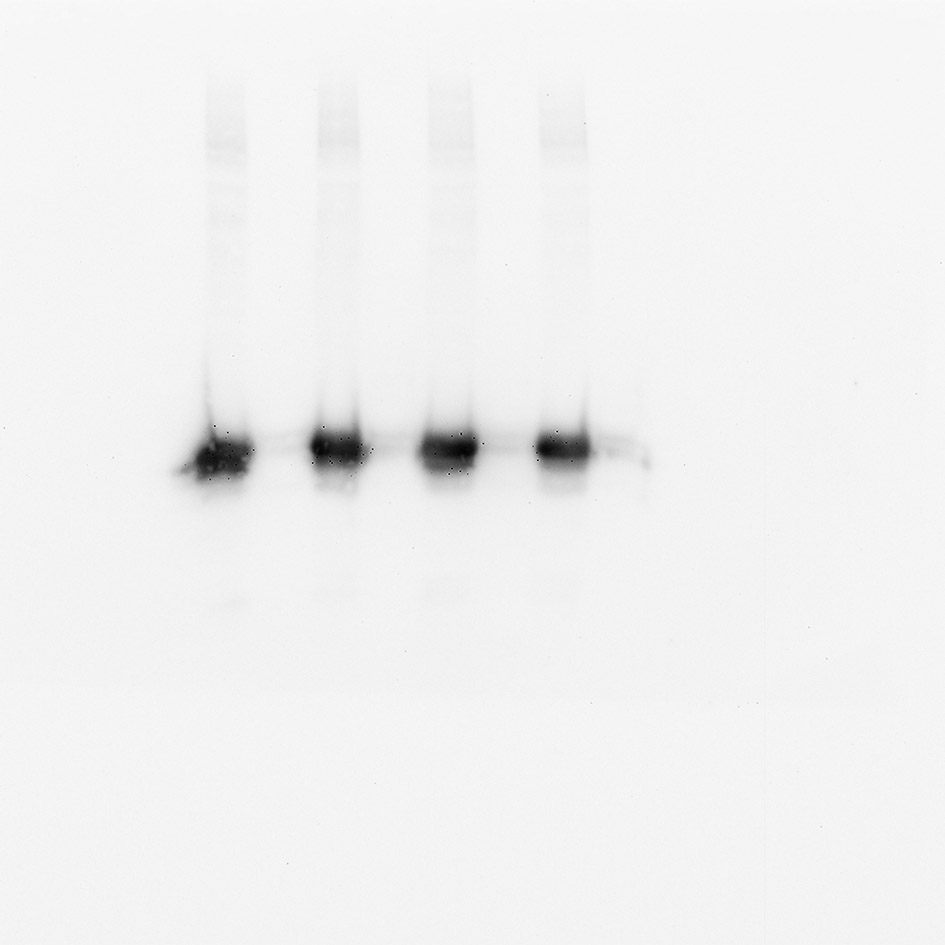

Supplement: Figure 1—source data 1. — A–D contain uncropped western blots shown in Figure 1A–D. Folders A’–D’ contain biological replicates of the respective experiments. Prizm files A’–D’ quantify relative Shh release rates based on the data shown in folders A’–D’. [file elife-86920-fig1-data1.zip › Figure_1_Source_Data_1 /D'_Fig_1_quant_242/24-02-15_V790_Gel 3_antiShh_1min.jpg]

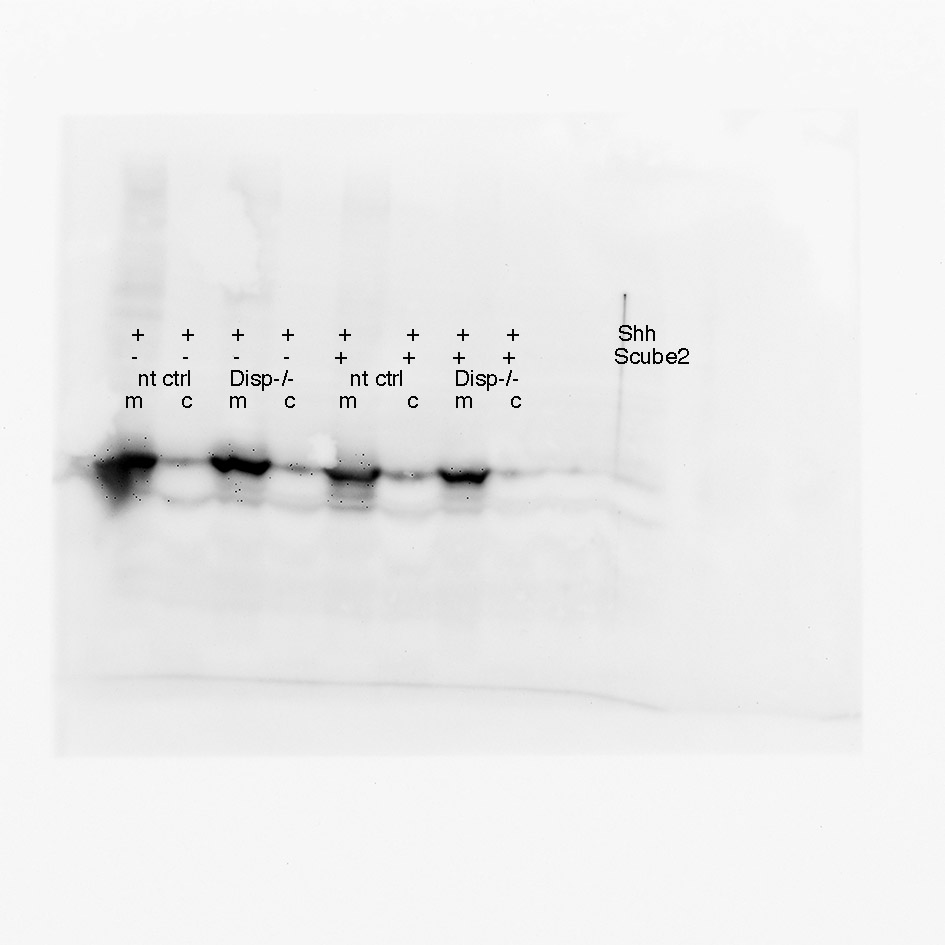

Supplement: Figure 1—source data 1. — A–D contain uncropped western blots shown in Figure 1A–D. Folders A’–D’ contain biological replicates of the respective experiments. Prizm files A’–D’ quantify relative Shh release rates based on the data shown in folders A’–D’. [file elife-86920-fig1-data1.zip › Figure_1_Source_Data_1 /D'_Fig_1_quant_242/V757_6_0.5min_aShh labelled.jpg]

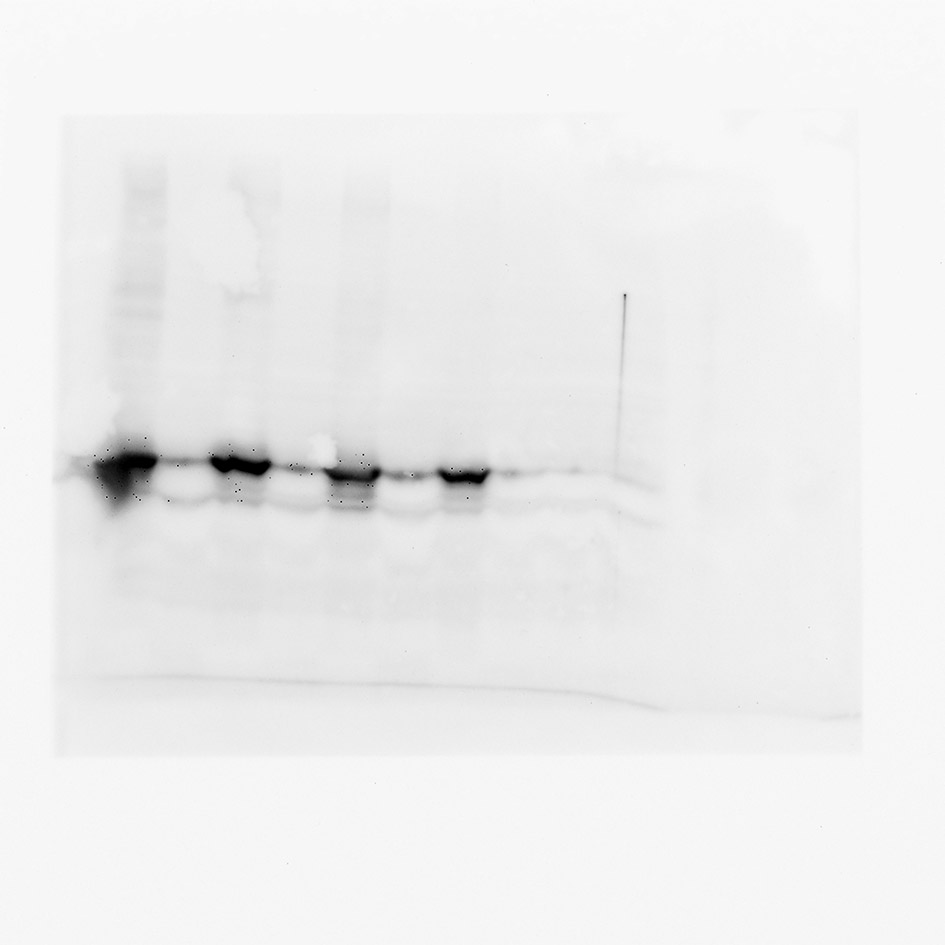

Supplement: Figure 1—source data 1. — A–D contain uncropped western blots shown in Figure 1A–D. Folders A’–D’ contain biological replicates of the respective experiments. Prizm files A’–D’ quantify relative Shh release rates based on the data shown in folders A’–D’. [file elife-86920-fig1-data1.zip › Figure_1_Source_Data_1 /D'_Fig_1_quant_242/V757_6_0.5min_aShh.jpg]

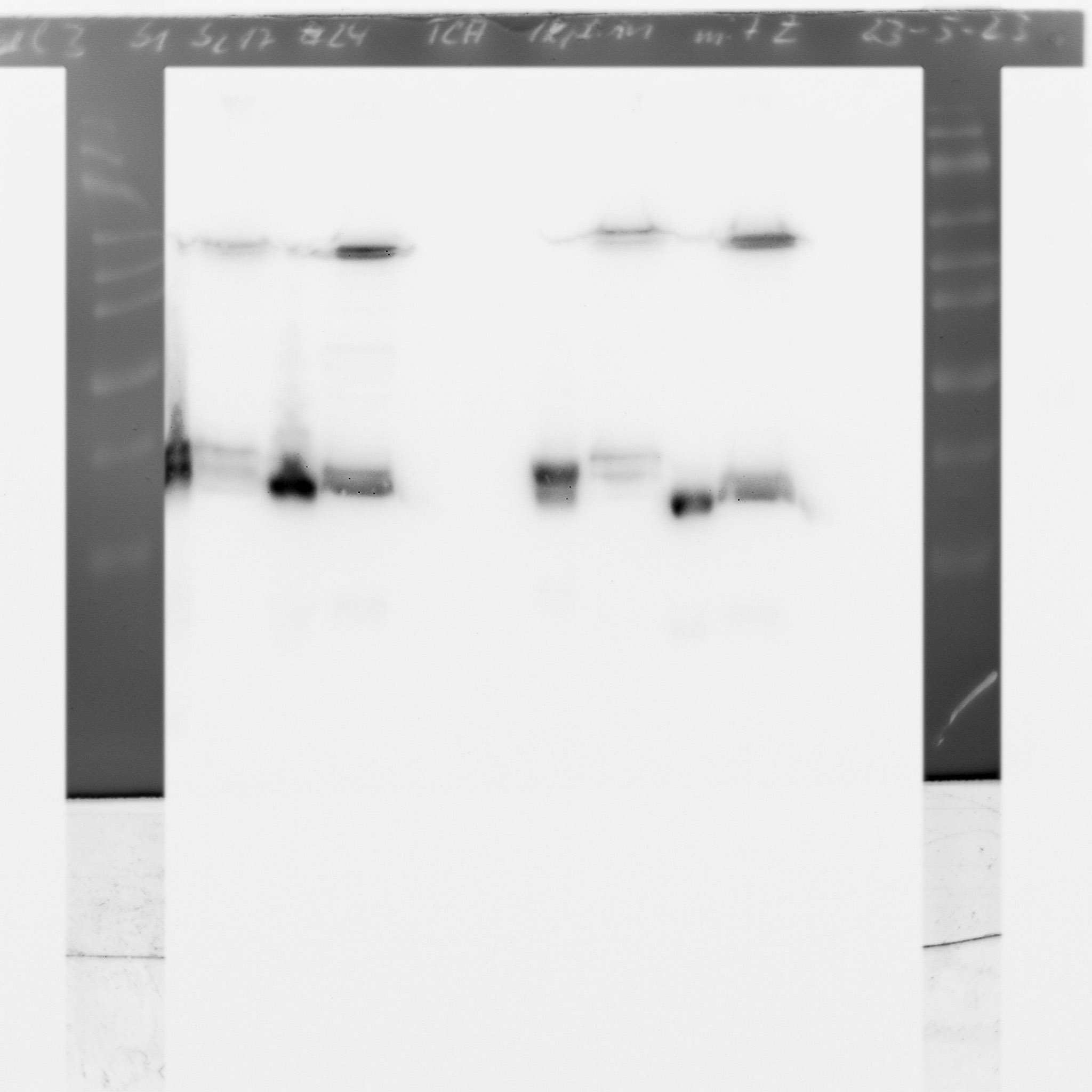

Supplement: Figure 1—figure supplement 1—source data 1. [file elife-86920-fig1-figsupp1-data1.zip › Figure Supplement 1 - Source Data 1/C_24-05-23_16Bit_Gel 3_S1 S217 #24 TCA Heparin mit Zellen_antiShh Rabbit(neu)_5sec.jpg]

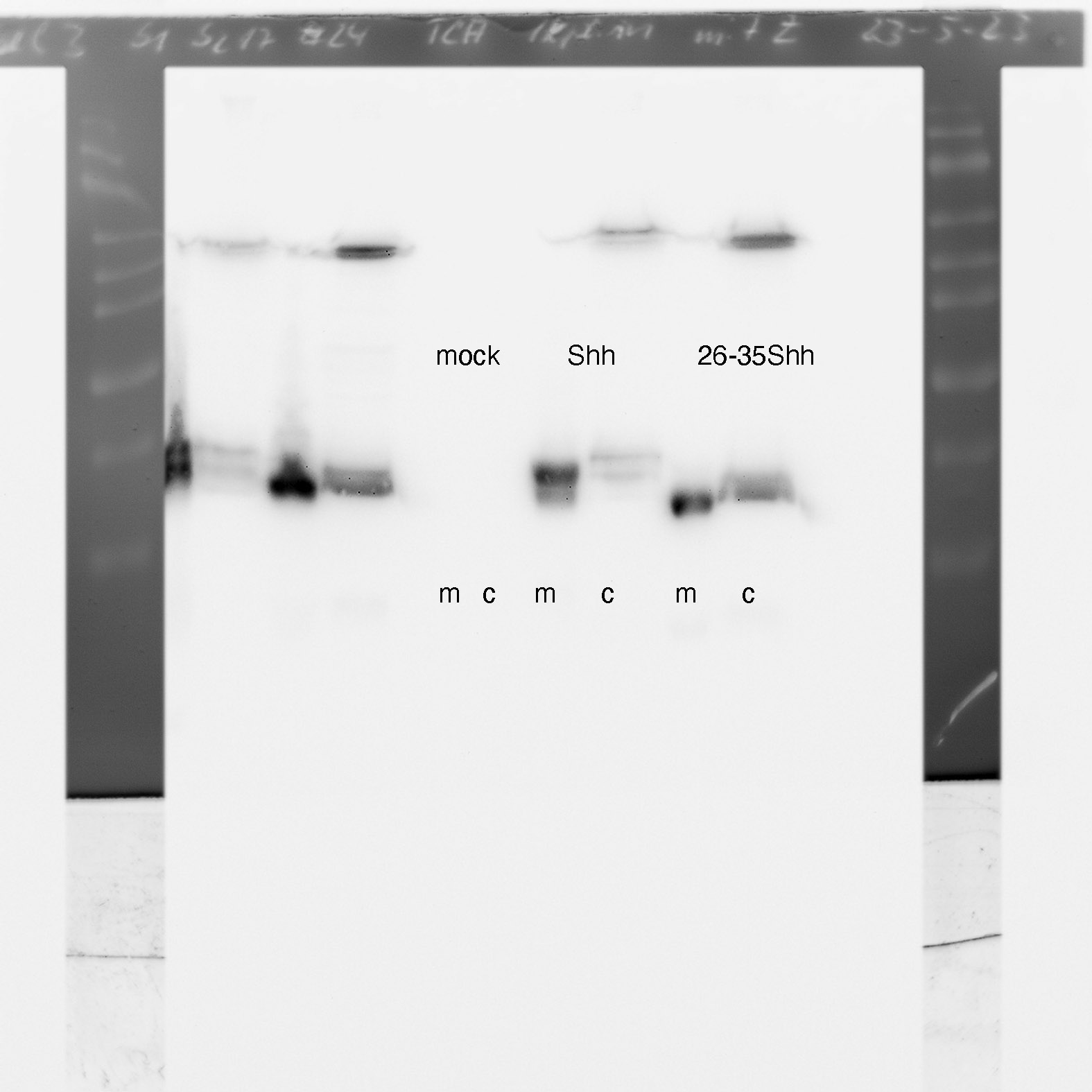

Supplement: Figure 1—figure supplement 1—source data 1. [file elife-86920-fig1-figsupp1-data1.zip › Figure Supplement 1 - Source Data 1/C_24-05-23_16Bit_Gel 3_S1 S217 #24 TCA Heparin mit Zellen_antiShh Rabbit(neu)_5sec_labelled.jpg]

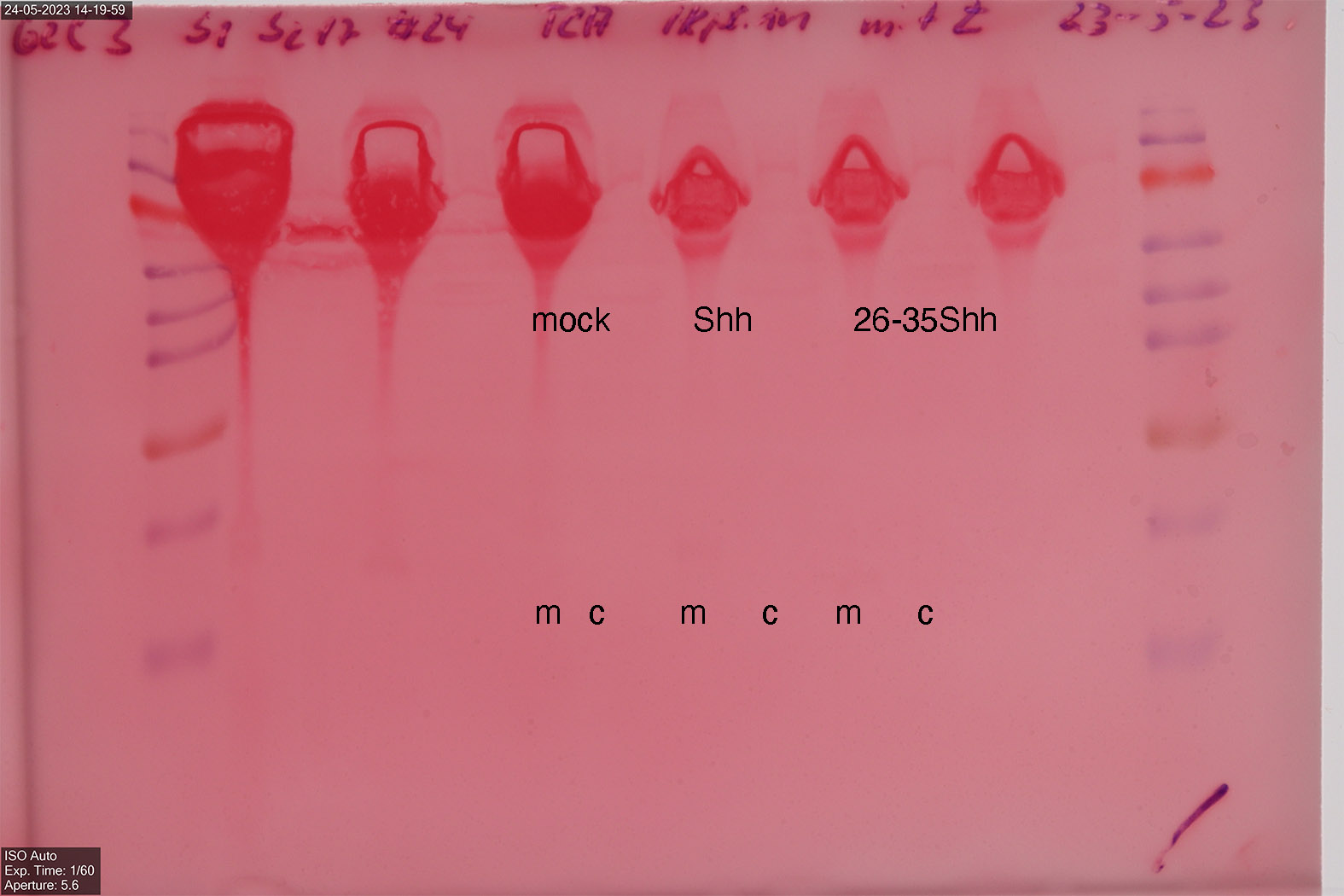

Supplement: Figure 1—figure supplement 1—source data 1. [file elife-86920-fig1-figsupp1-data1.zip › Figure Supplement 1 - Source Data 1/C_Gel 3 Ladungskontrolle 24-05-23labelled.jpg]

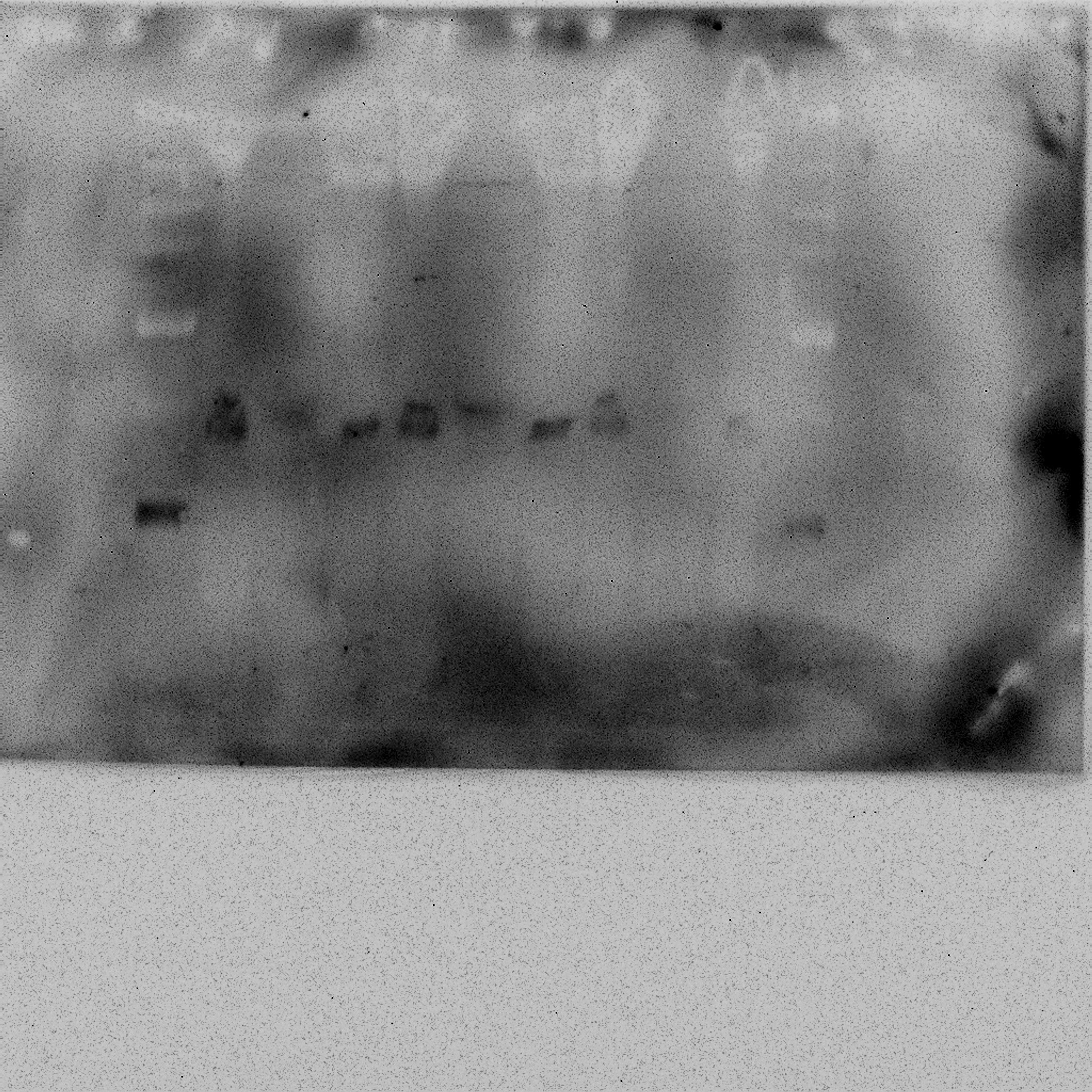

Supplement: Figure 1—figure supplement 1—source data 1. [file elife-86920-fig1-figsupp1-data1.zip › Figure Supplement 1 - Source Data 1/D_02-06-23_16Bit_Gel 2_ Panc 4-6_anti Shh Rabbit_3min4sec.jpg]

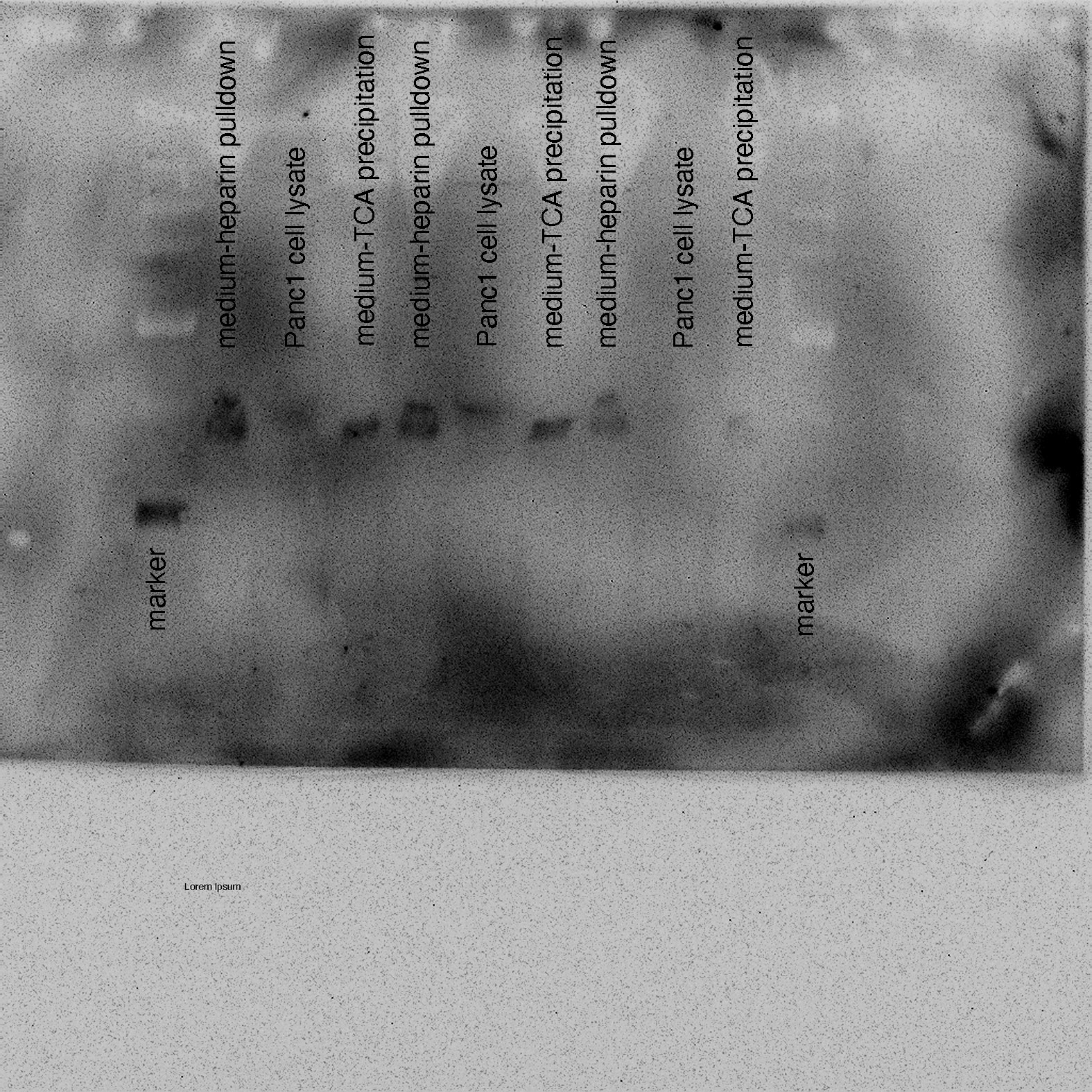

Supplement: Figure 1—figure supplement 1—source data 1. [file elife-86920-fig1-figsupp1-data1.zip › Figure Supplement 1 - Source Data 1/D_02-06-23_16Bit_Gel 2_ Panc 4-6_anti Shh Rabbit_3min4sec_labelled.jpg]

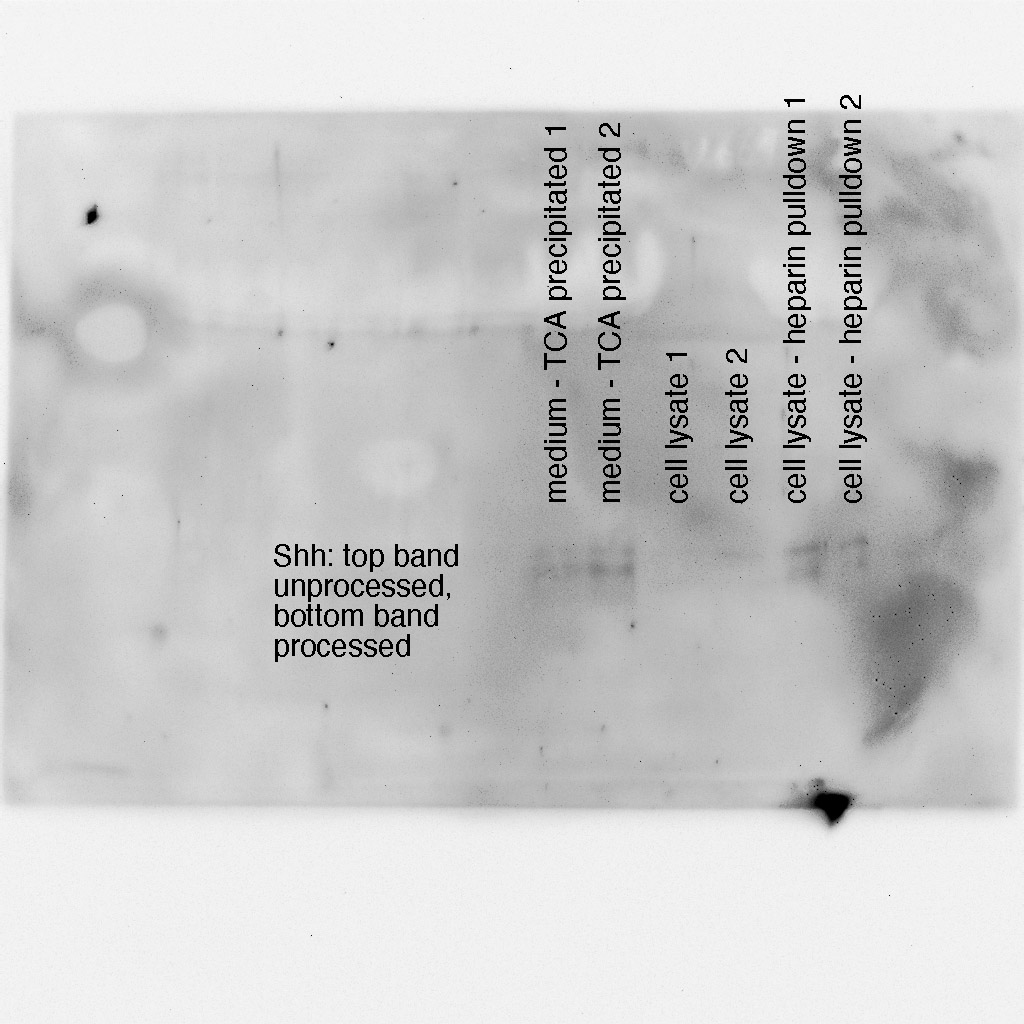

Supplement: Figure 1—figure supplement 1—source data 1. [file elife-86920-fig1-figsupp1-data1.zip › Figure Supplement 1 - Source Data 1/D_V661_6min labelled.jpg]

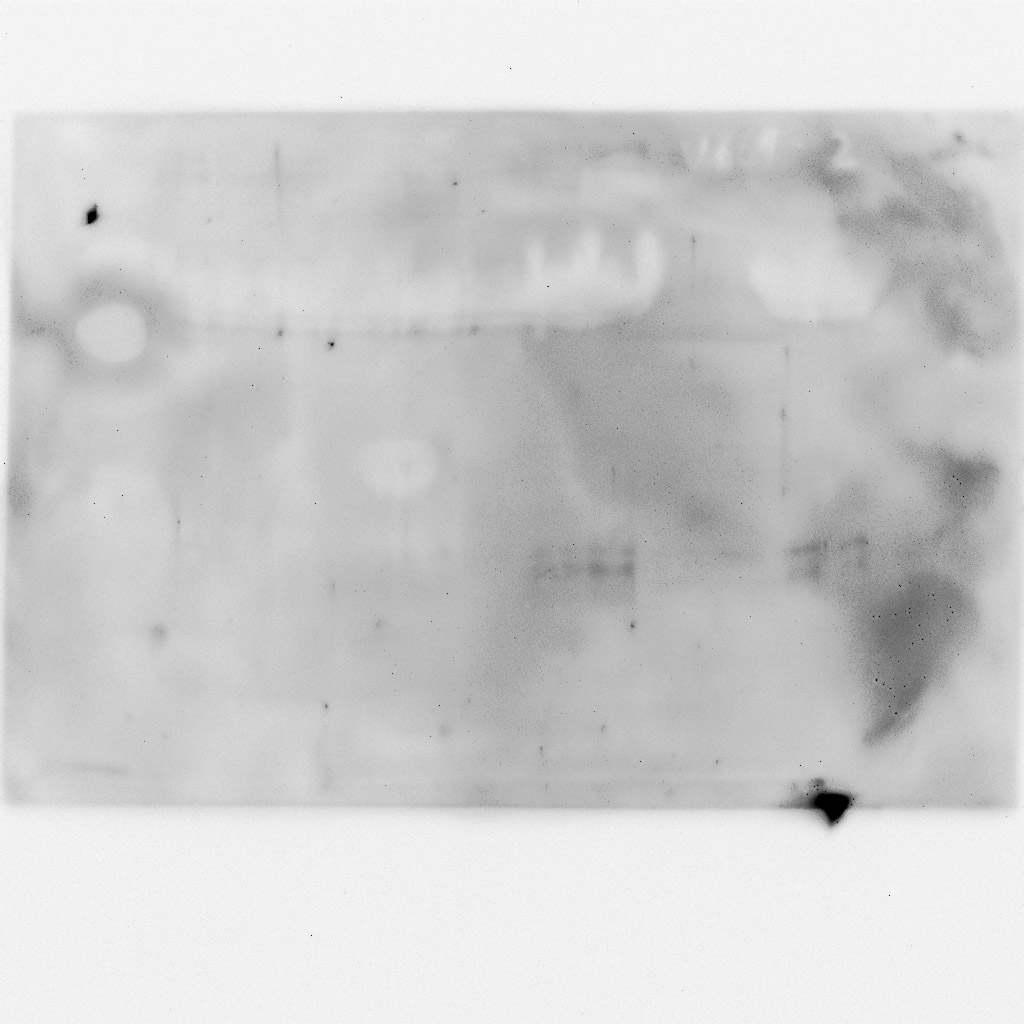

Supplement: Figure 1—figure supplement 1—source data 1. [file elife-86920-fig1-figsupp1-data1.zip › Figure Supplement 1 - Source Data 1/D_V661_6min.jpg]

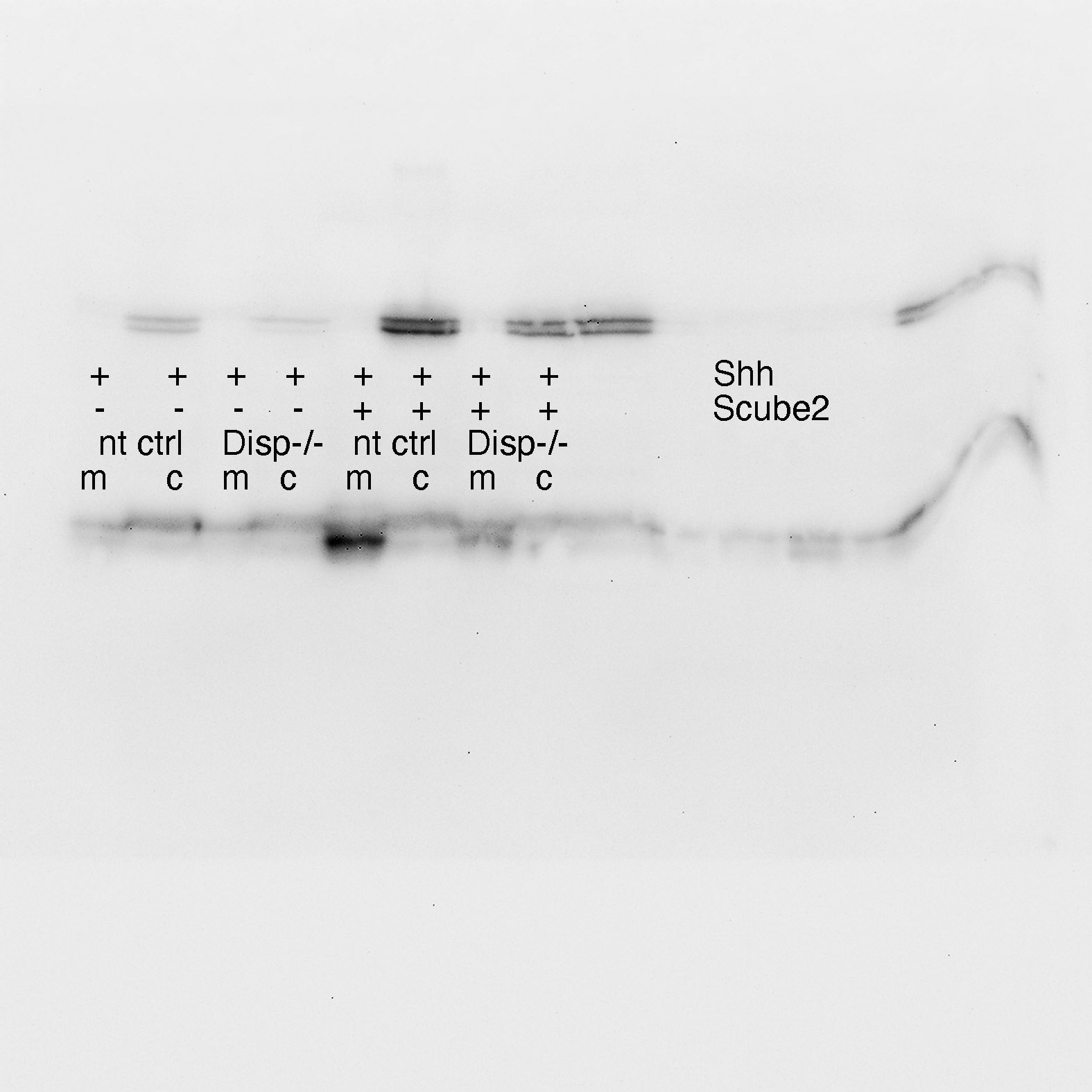

Supplement: Figure 1—figure supplement 2—source data 1. [file elife-86920-fig1-figsupp2-data1.zip › Figure 1-Figure Supplement 1 - Source Data 1/A_V744_1_1min labelled.jpg]

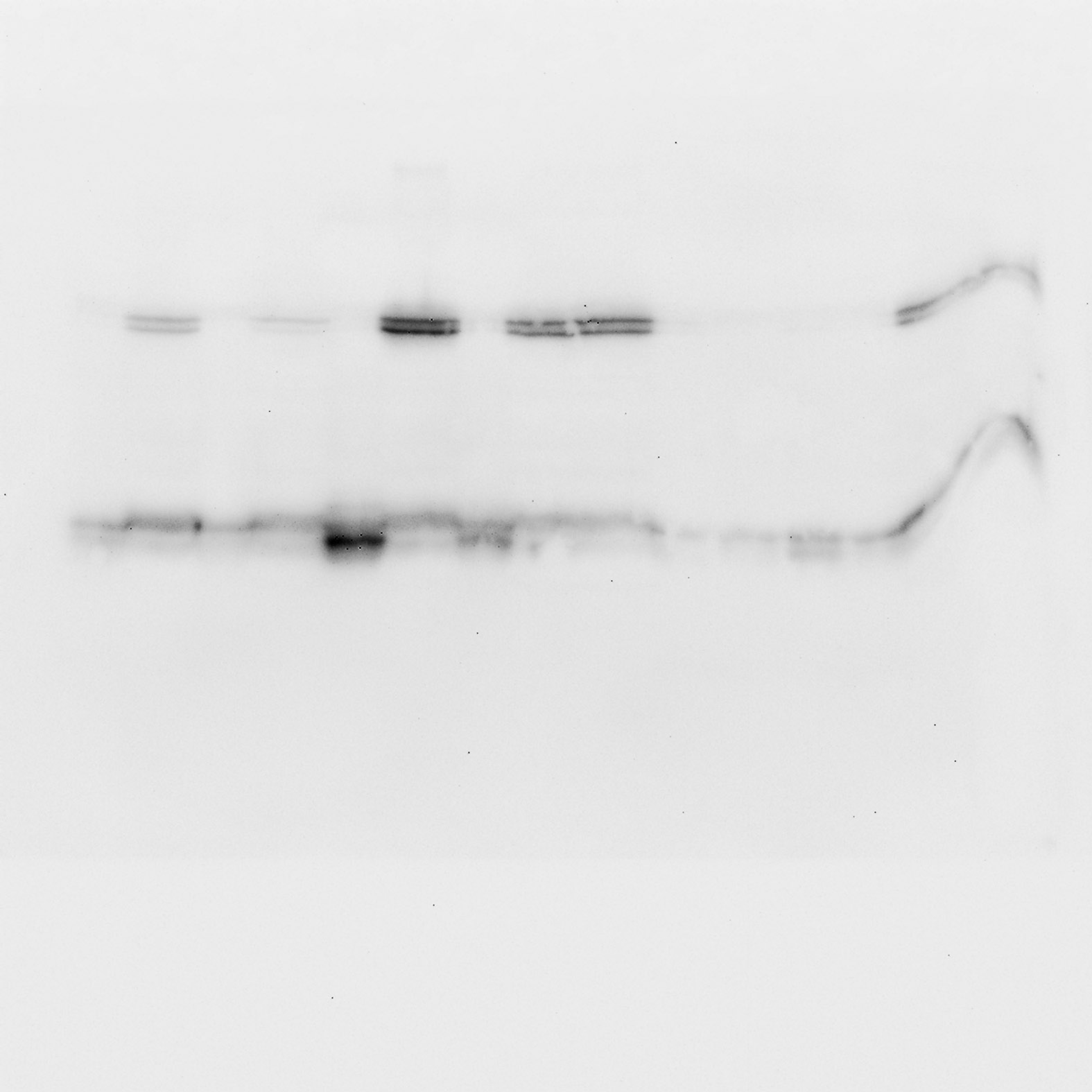

Supplement: Figure 1—figure supplement 2—source data 1. [file elife-86920-fig1-figsupp2-data1.zip › Figure 1-Figure Supplement 1 - Source Data 1/A_V744_1_1min.jpg]

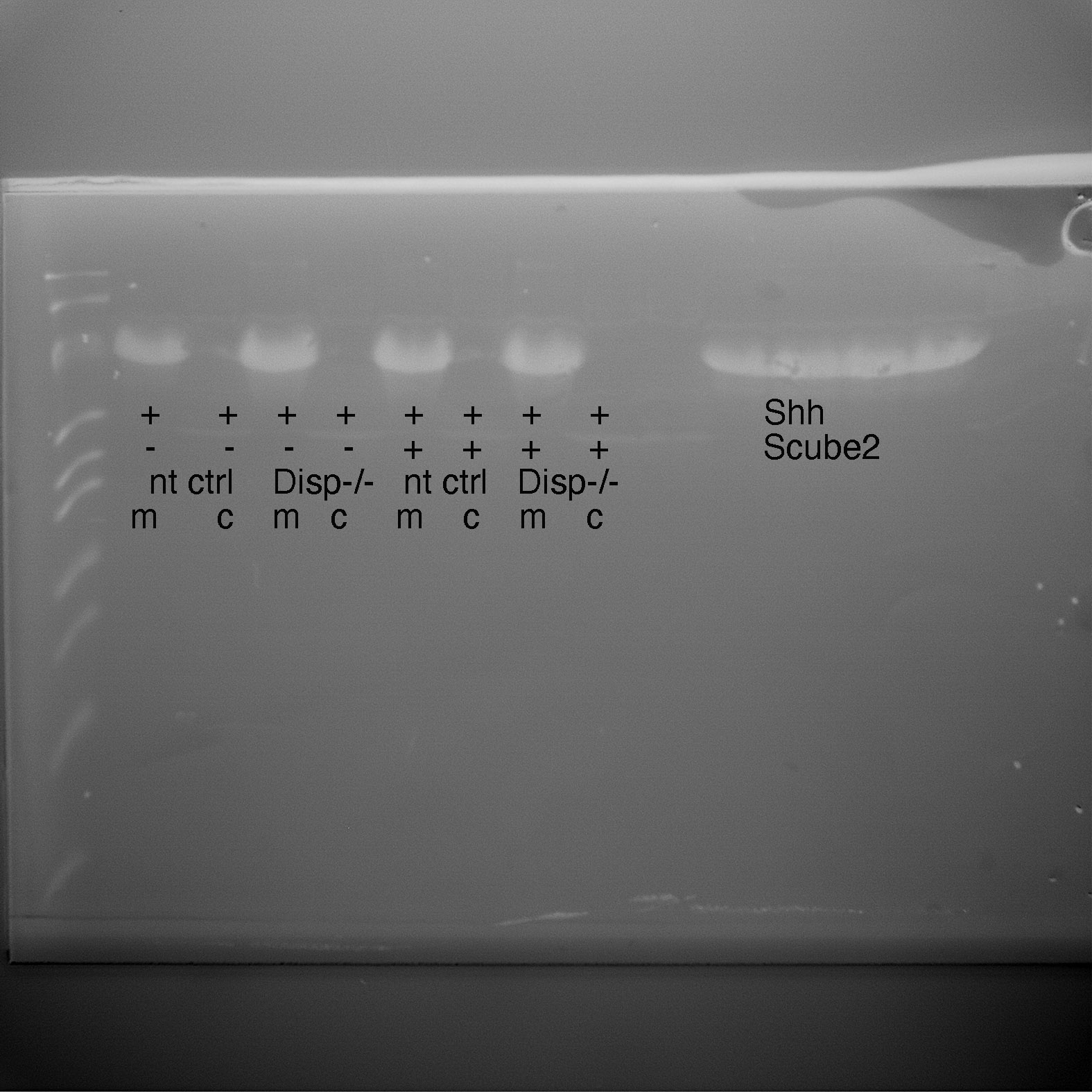

Supplement: Figure 1—figure supplement 2—source data 1. [file elife-86920-fig1-figsupp2-data1.zip › Figure 1-Figure Supplement 1 - Source Data 1/A_V744_1_Pon labelled.jpg]

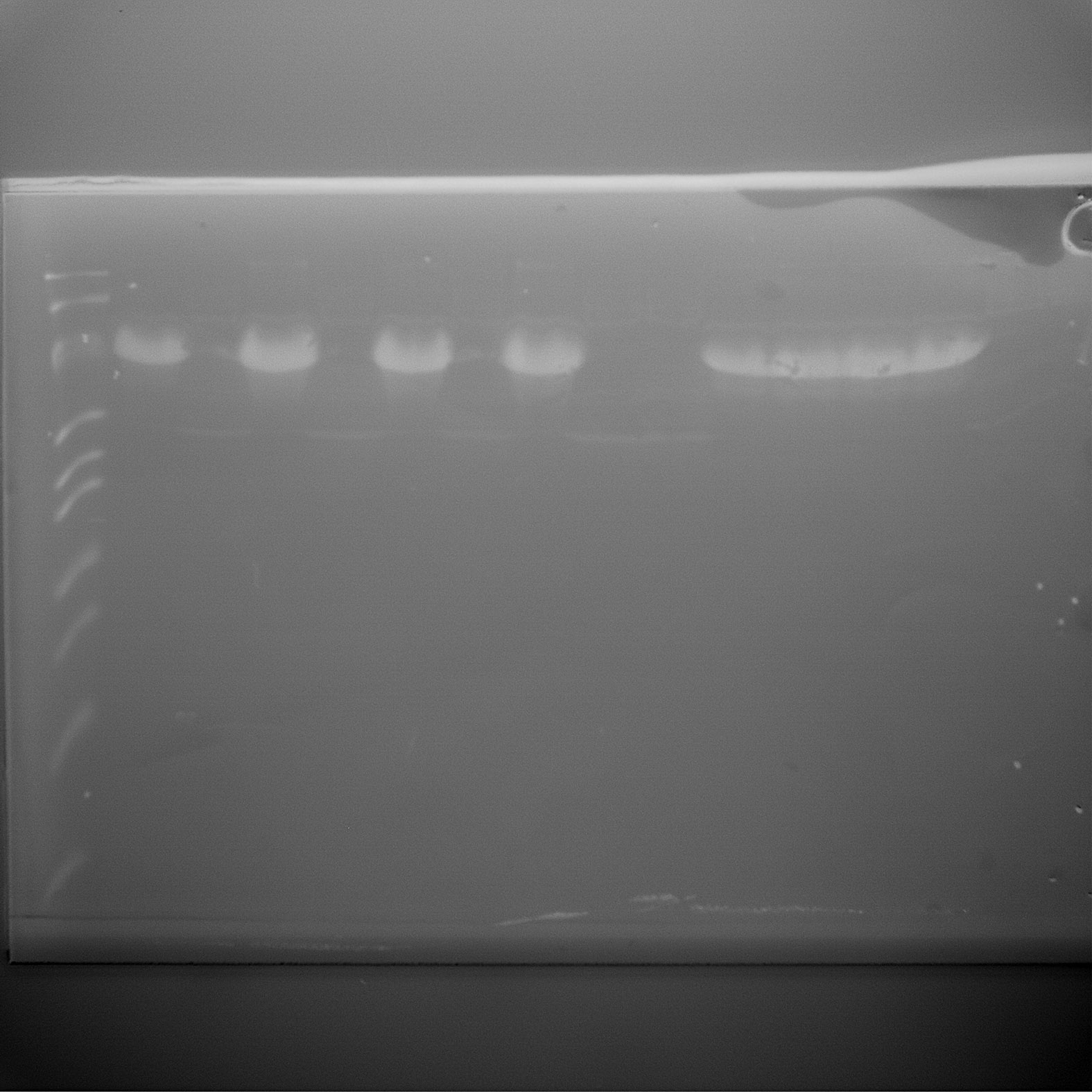

Supplement: Figure 1—figure supplement 2—source data 1. [file elife-86920-fig1-figsupp2-data1.zip › Figure 1-Figure Supplement 1 - Source Data 1/A_V744_1_Pon.jpg]

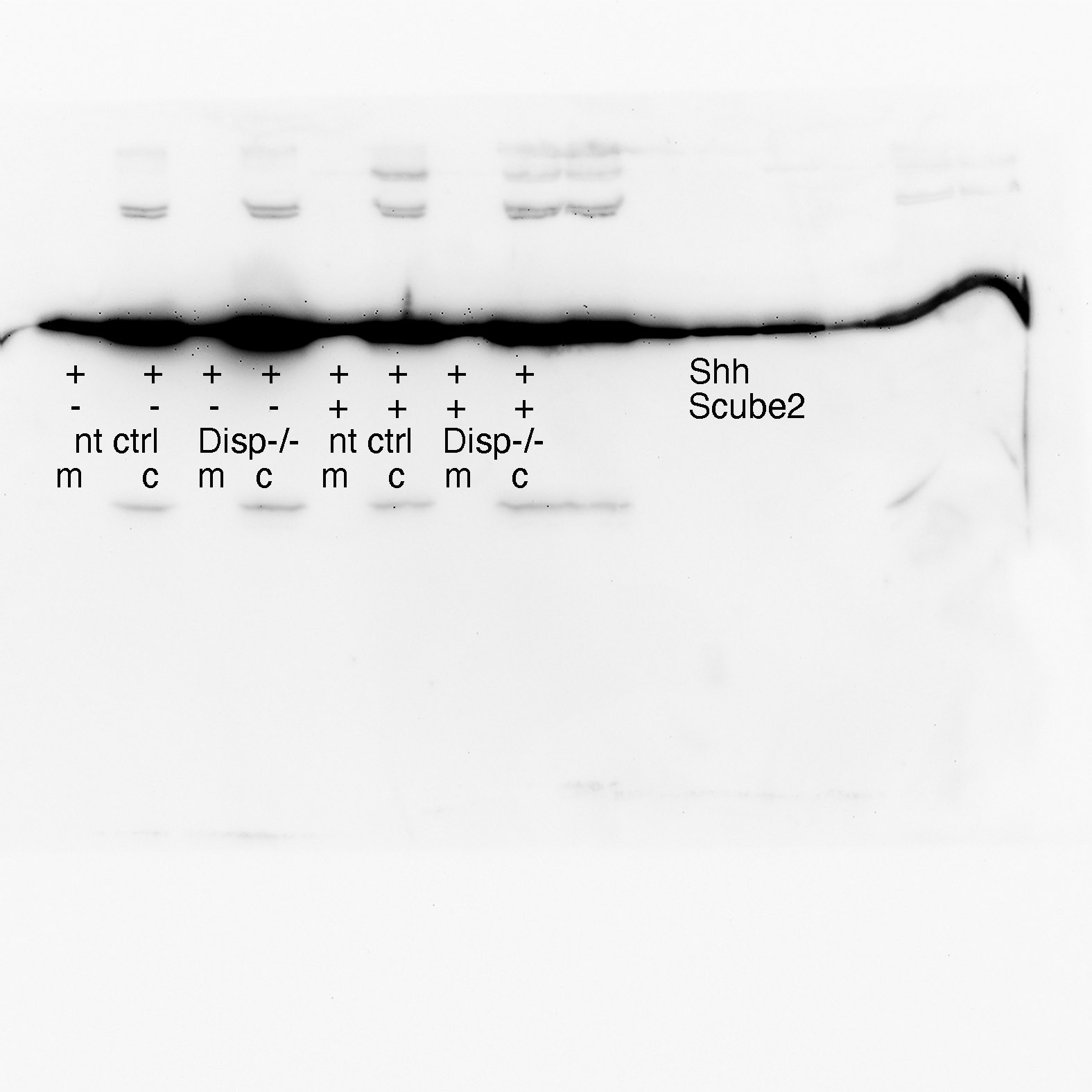

Supplement: Figure 1—figure supplement 2—source data 1. [file elife-86920-fig1-figsupp2-data1.zip › Figure 1-Figure Supplement 1 - Source Data 1/A_V744actin_1_80sec labelled.jpg]

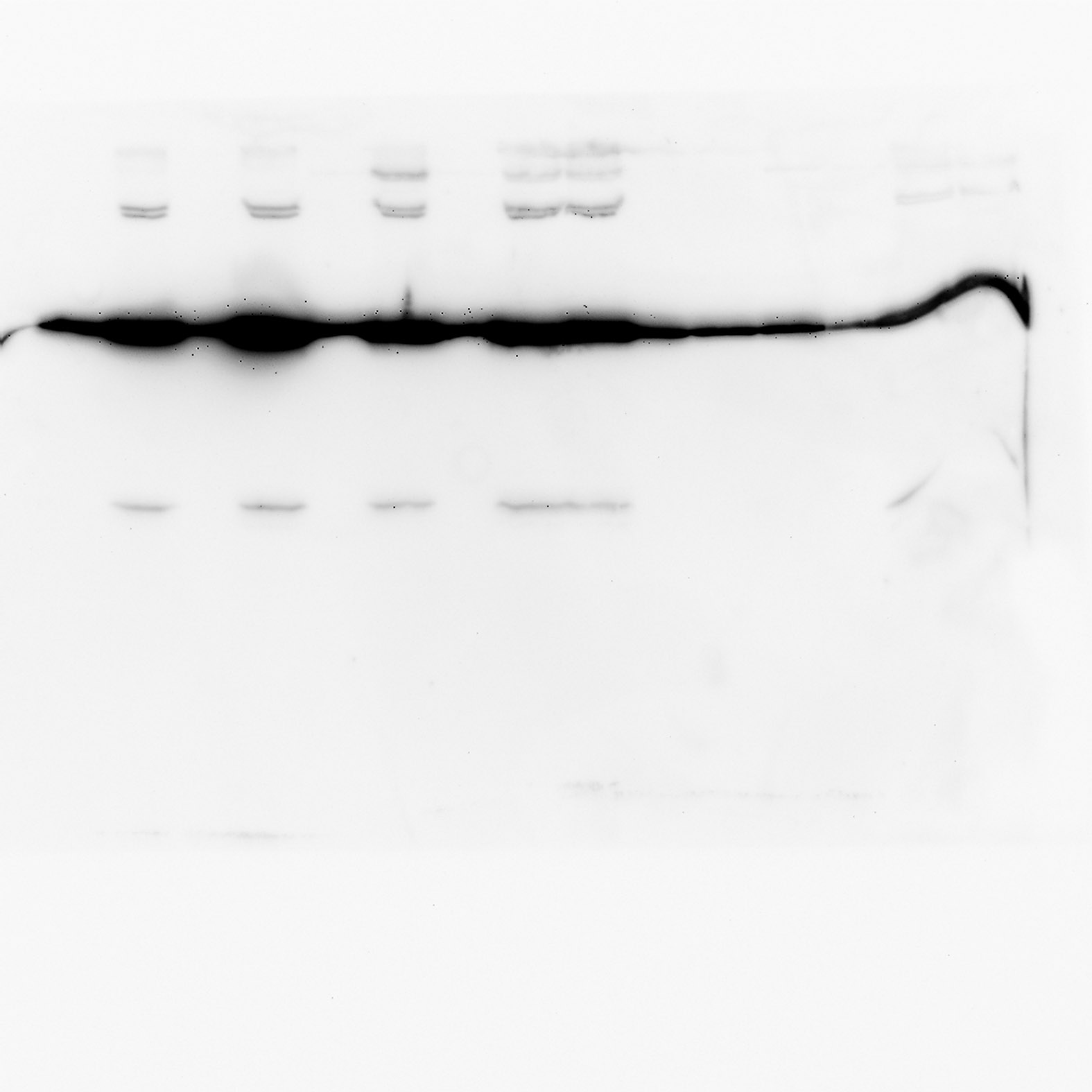

Supplement: Figure 1—figure supplement 2—source data 1. [file elife-86920-fig1-figsupp2-data1.zip › Figure 1-Figure Supplement 1 - Source Data 1/A_V744actin_1_80sec.jpg]

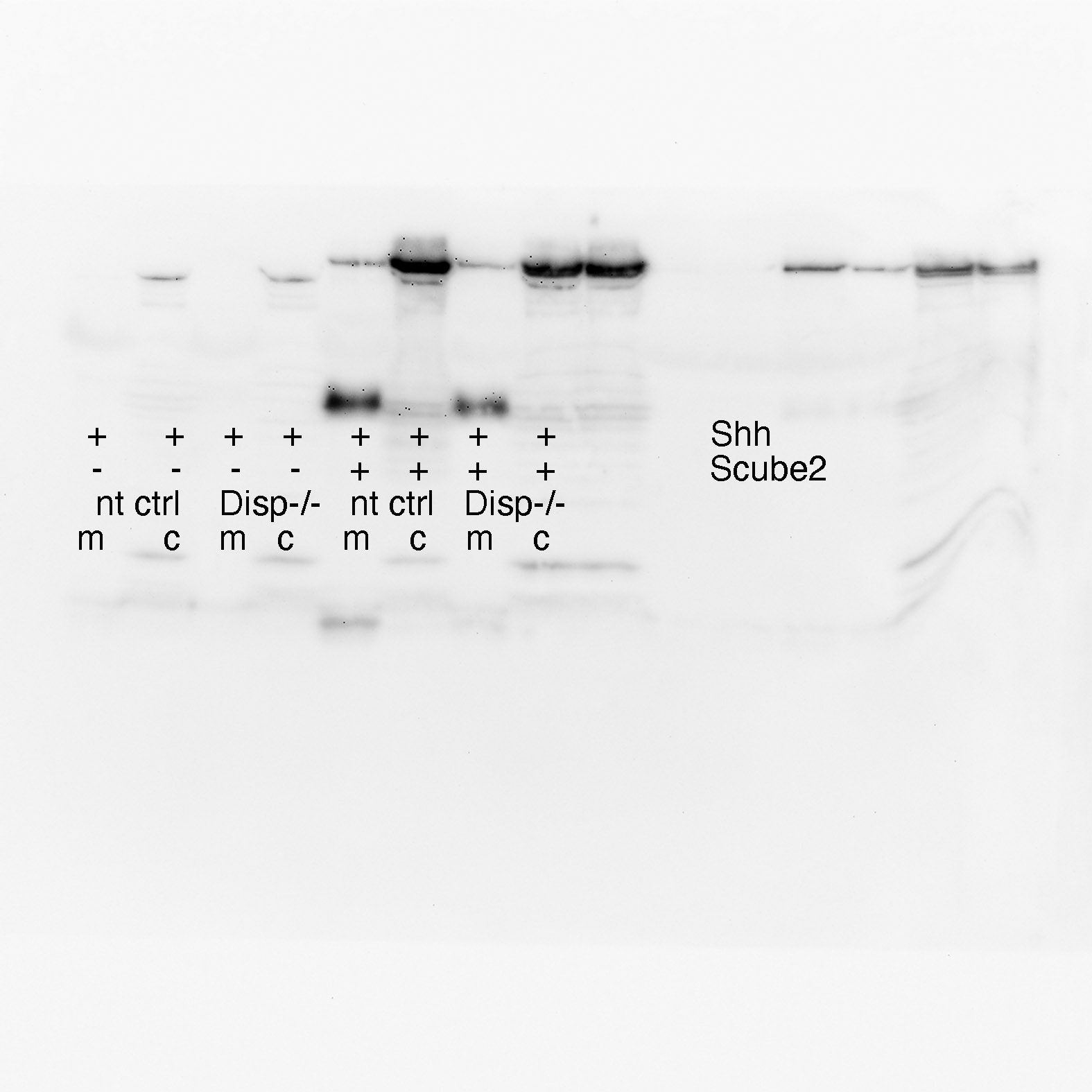

Supplement: Figure 1—figure supplement 2—source data 1. [file elife-86920-fig1-figsupp2-data1.zip › Figure 1-Figure Supplement 1 - Source Data 1/A_V744Flag_1_1min labelled.jpg]

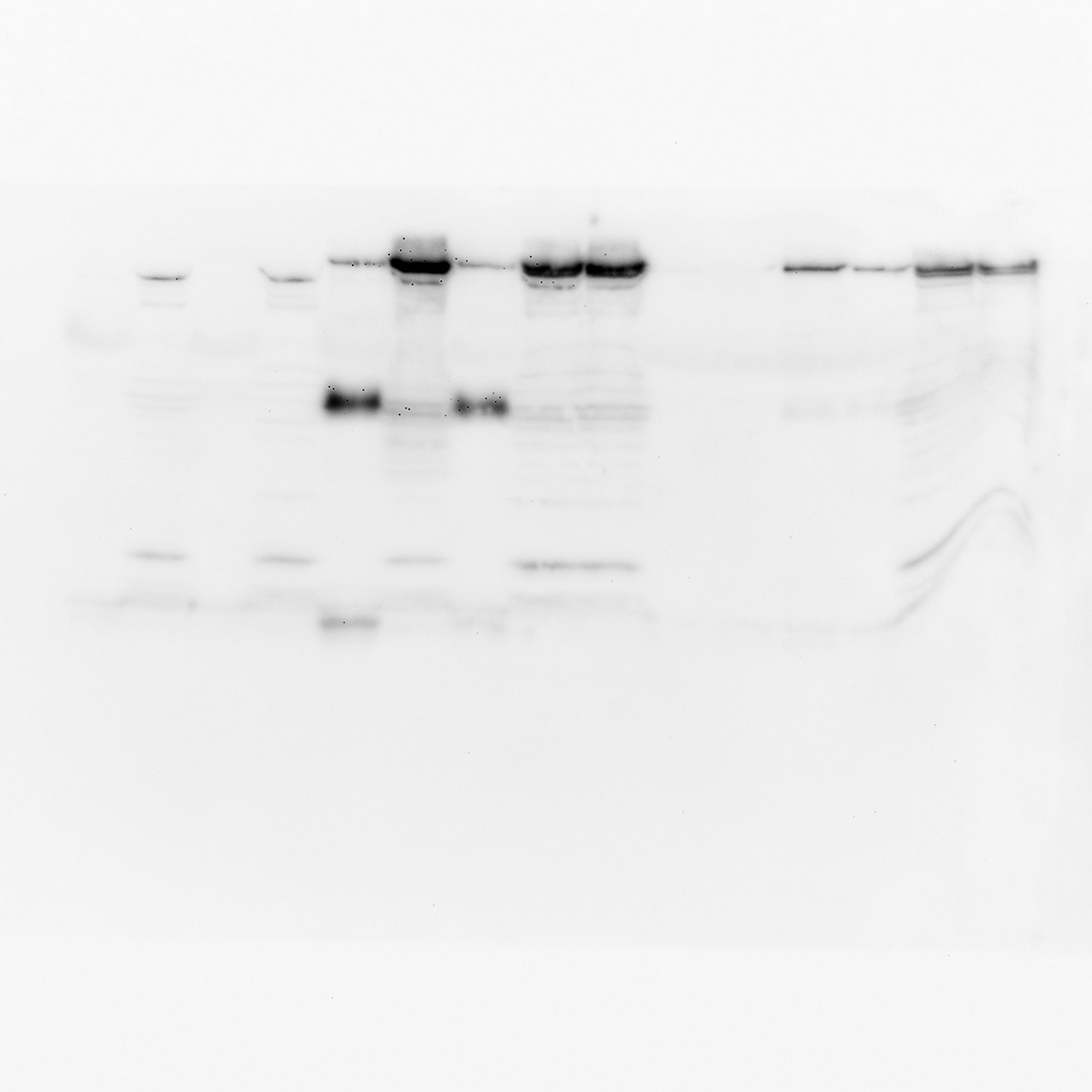

Supplement: Figure 1—figure supplement 2—source data 1. [file elife-86920-fig1-figsupp2-data1.zip › Figure 1-Figure Supplement 1 - Source Data 1/A_V744Flag_1_1min.jpg]

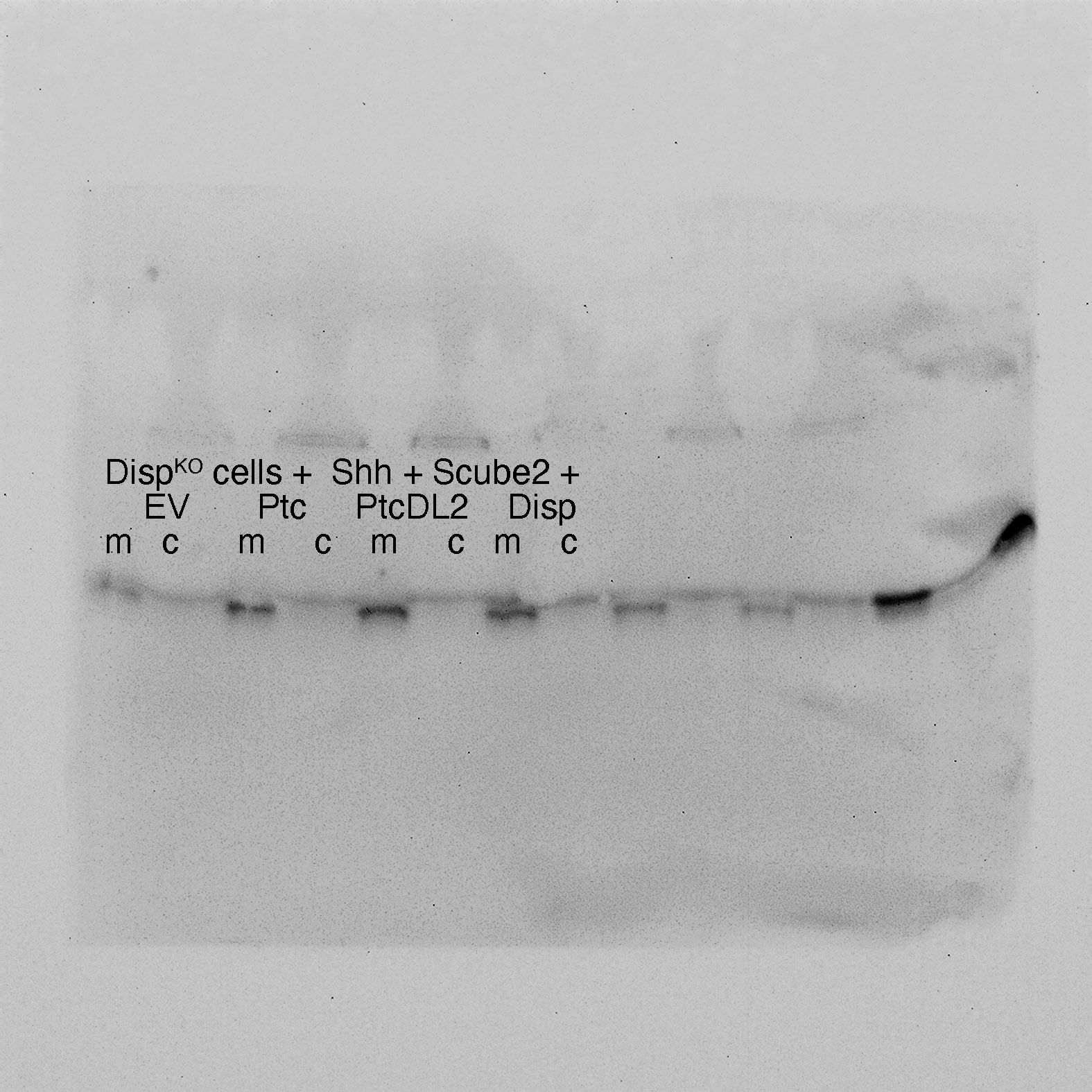

Supplement: Figure 1—figure supplement 2—source data 1. [file elife-86920-fig1-figsupp2-data1.zip › Figure 1-Figure Supplement 1 - Source Data 1/H_V725_1 C3 assay blot 3 1min labelled.jpg]

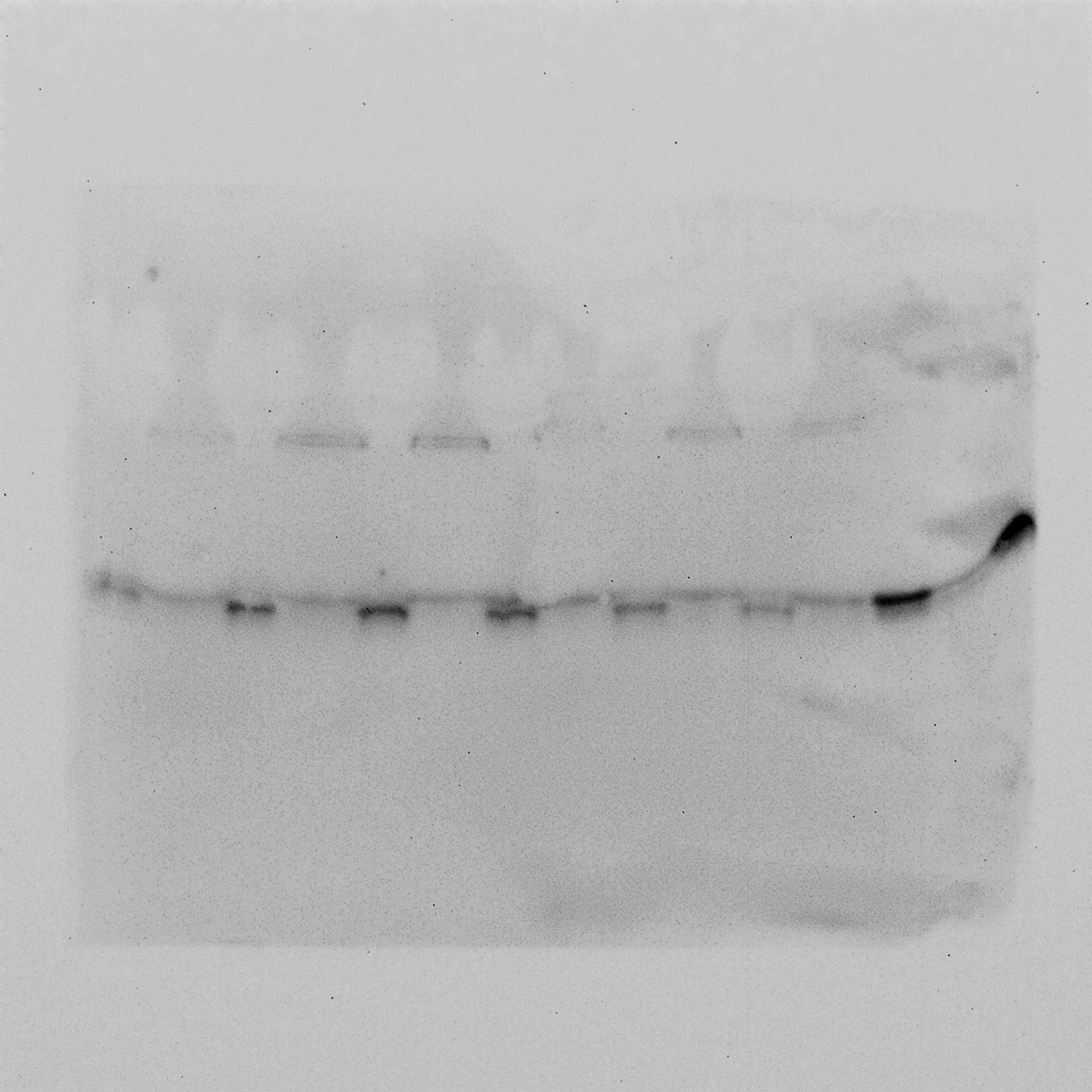

Supplement: Figure 1—figure supplement 2—source data 1. [file elife-86920-fig1-figsupp2-data1.zip › Figure 1-Figure Supplement 1 - Source Data 1/H_V725_1 C3 assay blot 3 1min.jpg]

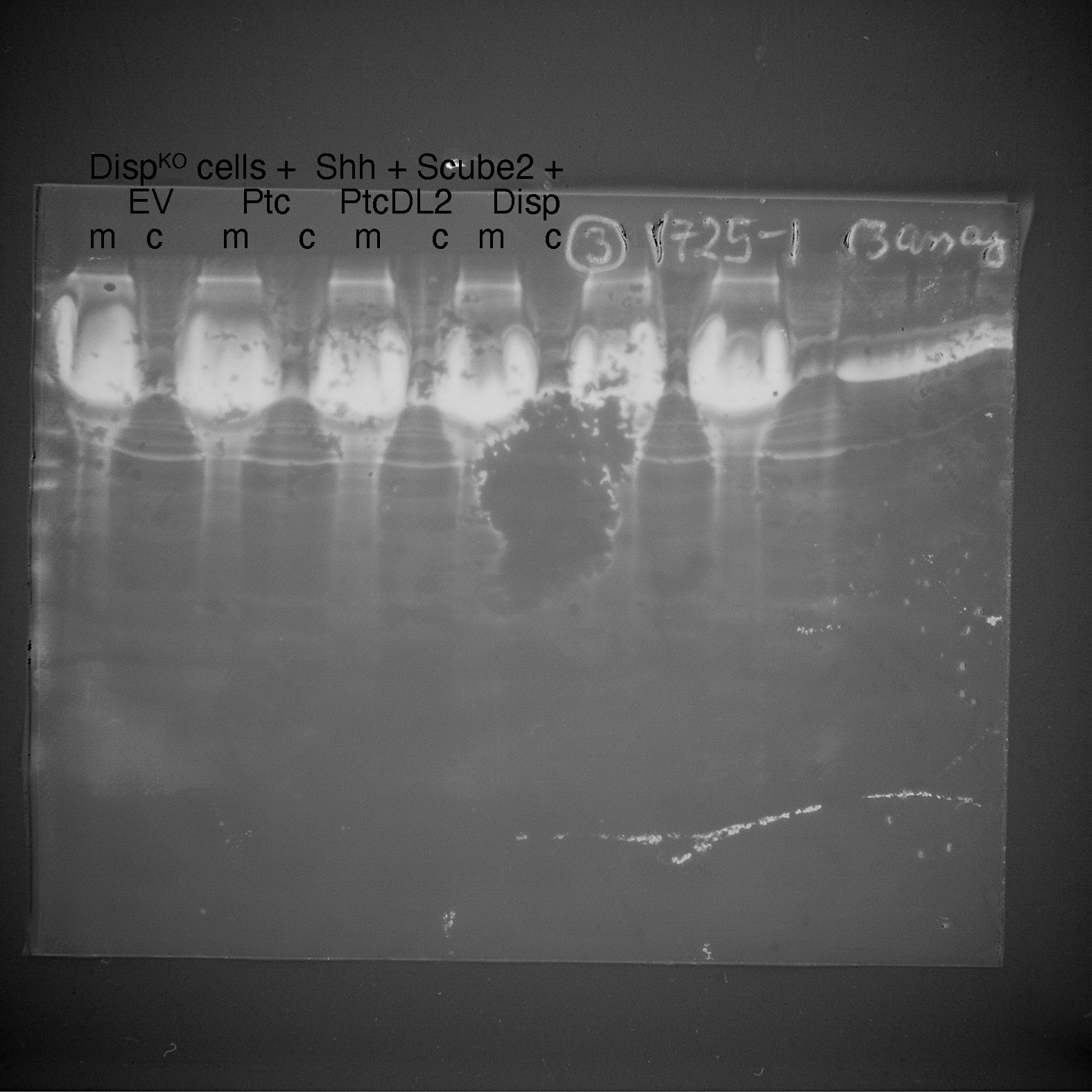

Supplement: Figure 1—figure supplement 2—source data 1. [file elife-86920-fig1-figsupp2-data1.zip › Figure 1-Figure Supplement 1 - Source Data 1/H_V725_1 Ponceau labelled.jpg]

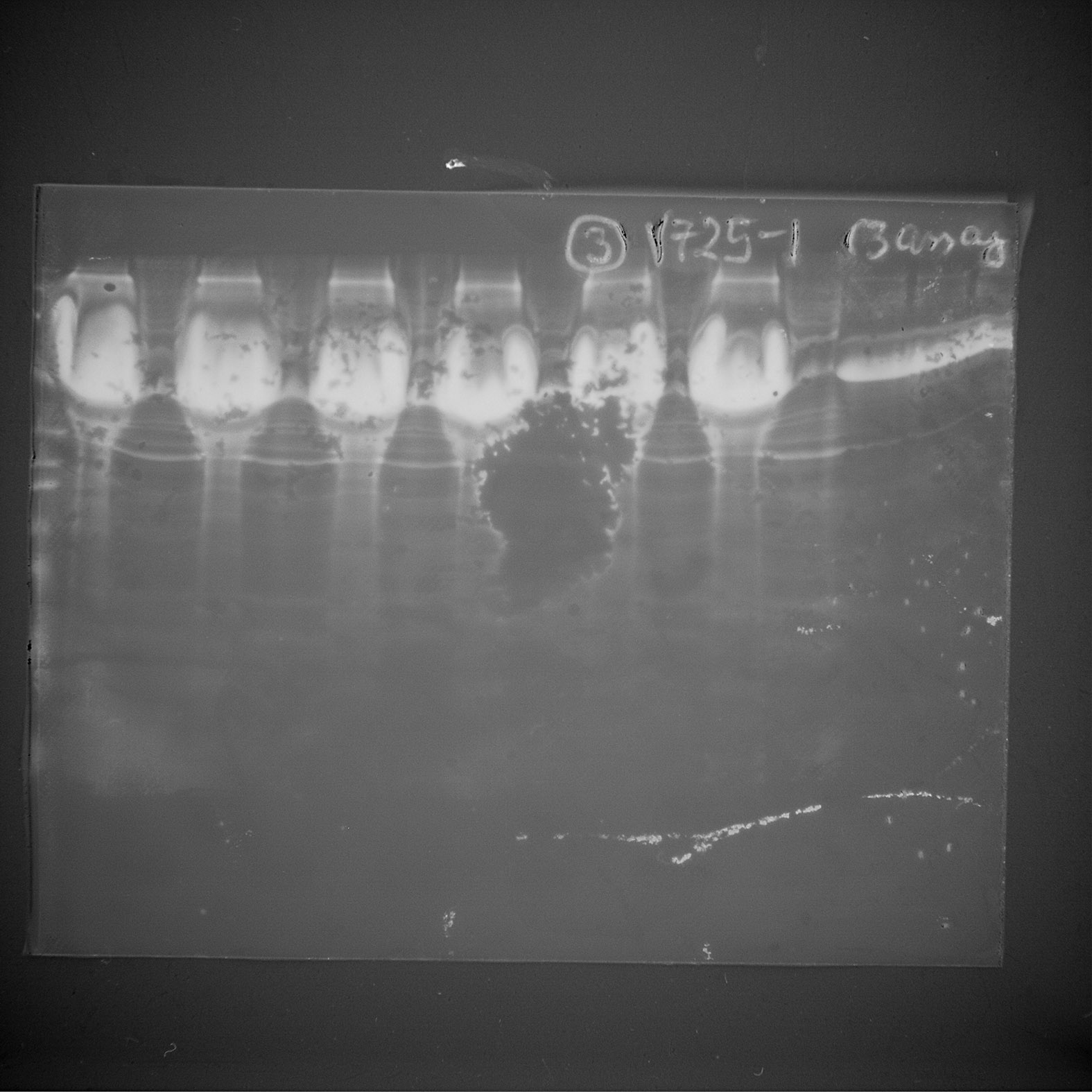

Supplement: Figure 1—figure supplement 2—source data 1. [file elife-86920-fig1-figsupp2-data1.zip › Figure 1-Figure Supplement 1 - Source Data 1/H_V725_1 Ponceau.jpg]

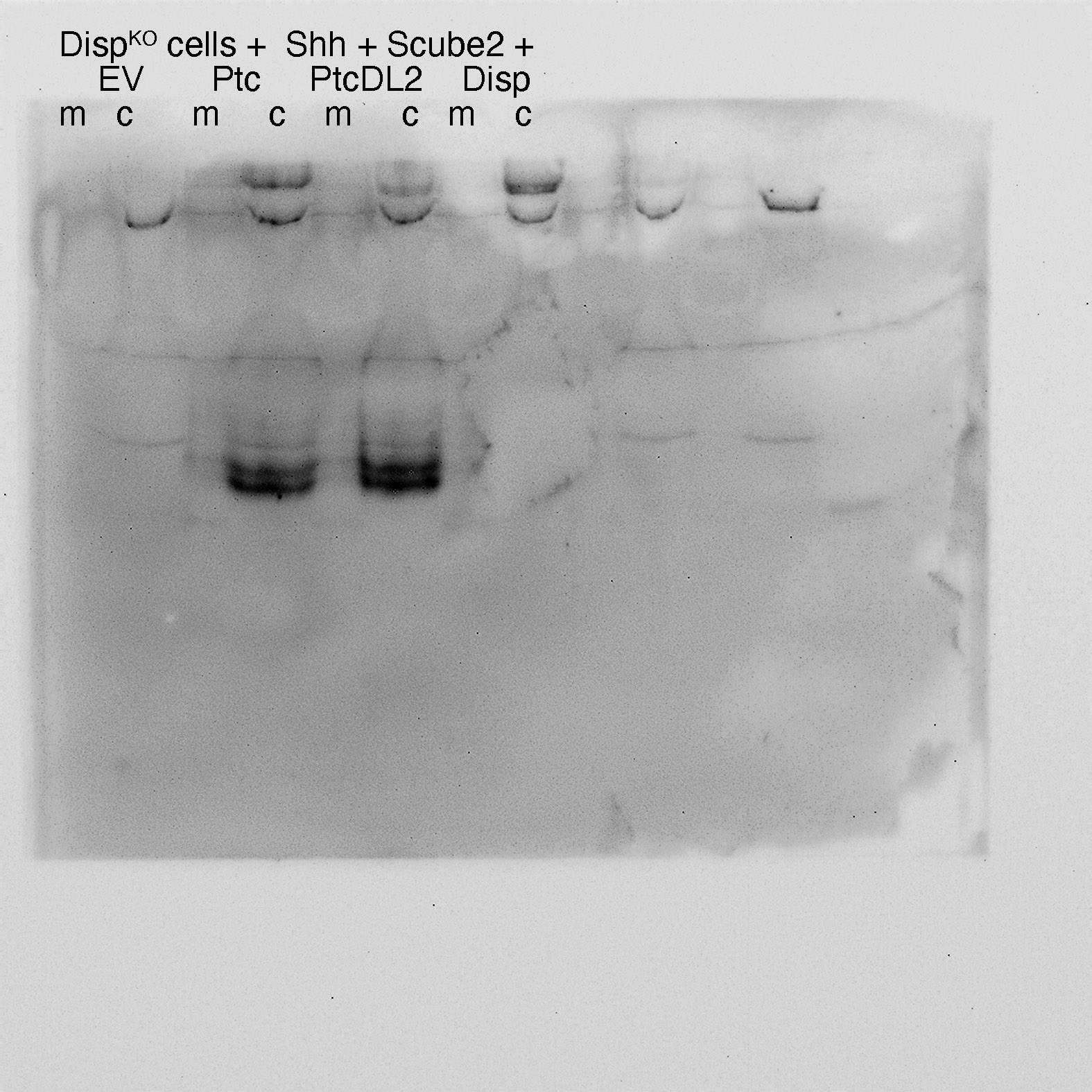

Supplement: Figure 1—figure supplement 2—source data 1. [file elife-86920-fig1-figsupp2-data1.zip › Figure 1-Figure Supplement 1 - Source Data 1/H_V725_1_strip1_antiHA_1min clabelled.jpg]

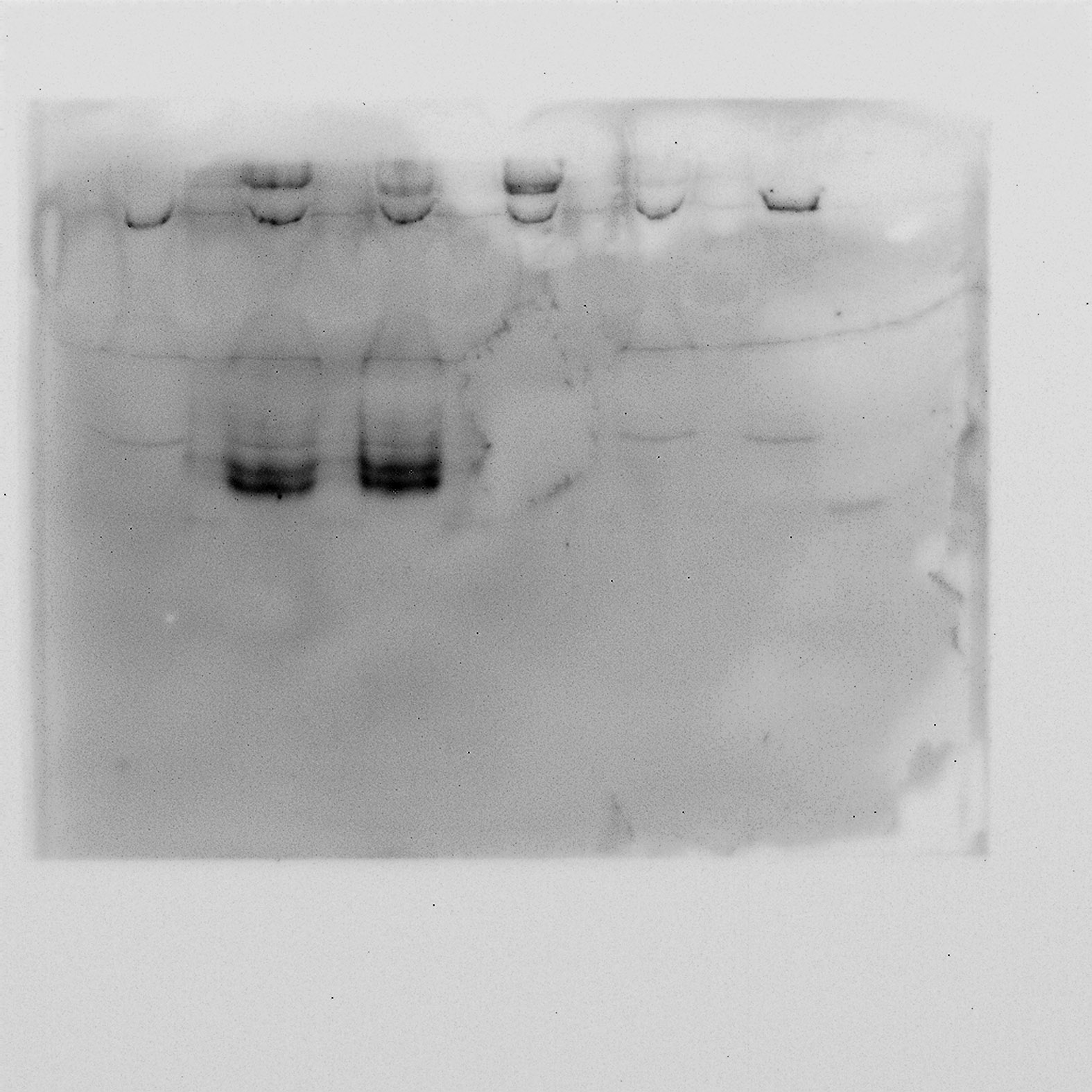

Supplement: Figure 1—figure supplement 2—source data 1. [file elife-86920-fig1-figsupp2-data1.zip › Figure 1-Figure Supplement 1 - Source Data 1/H_V725_1_strip1_antiHA_1min.jpg]

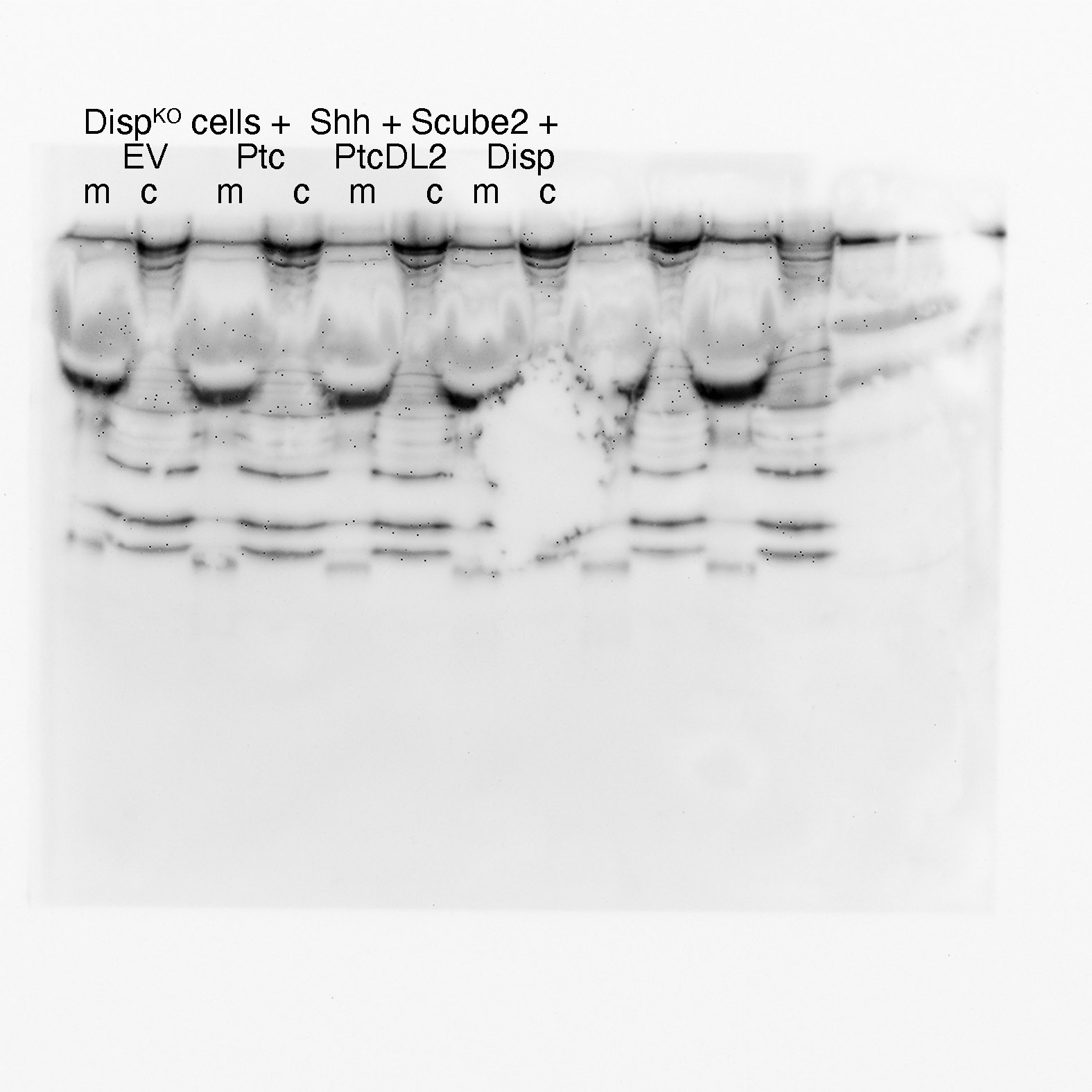

Supplement: Figure 1—figure supplement 2—source data 1. [file elife-86920-fig1-figsupp2-data1.zip › Figure 1-Figure Supplement 1 - Source Data 1/H_V725_1_strip_antiFLAG_1min labelled.jpg]

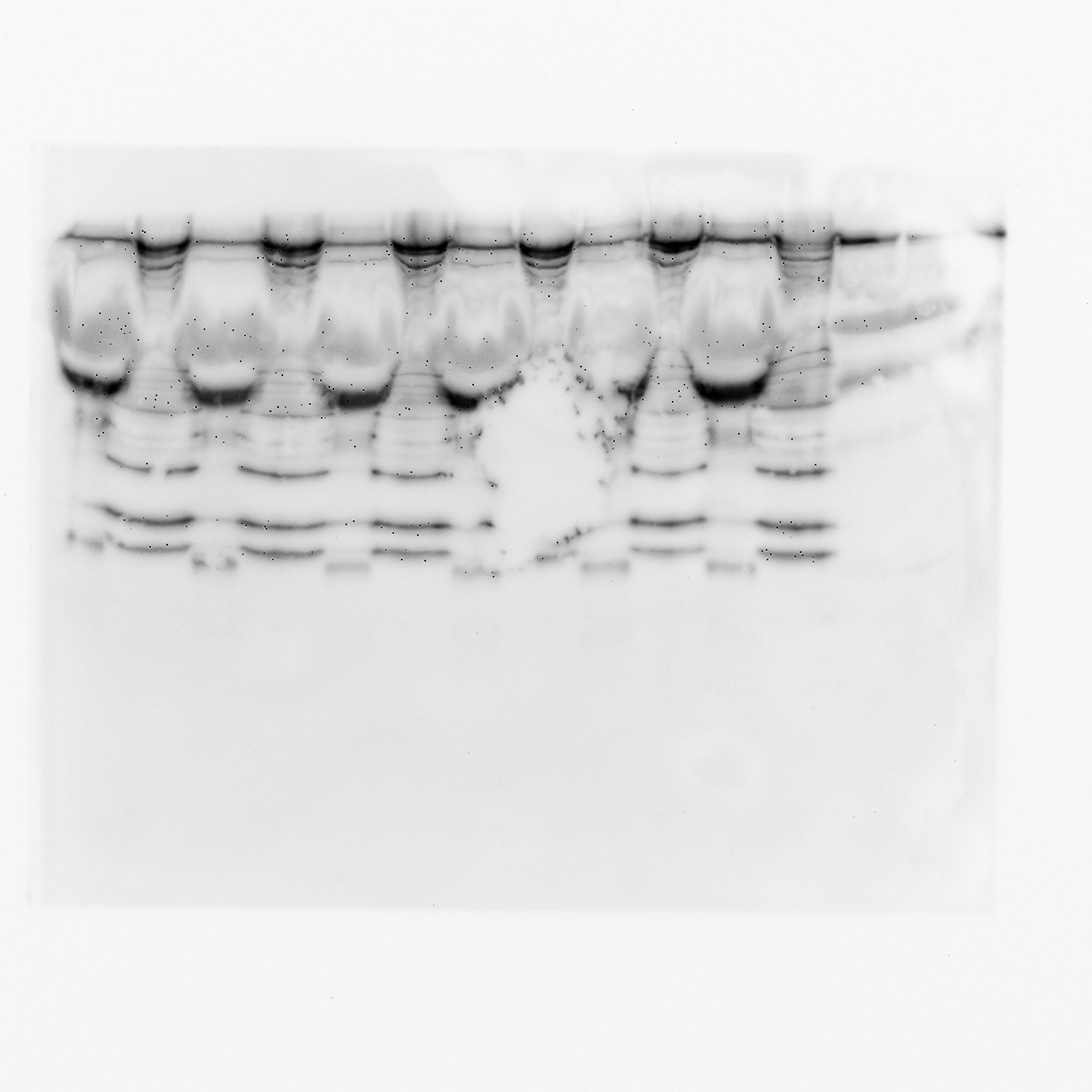

Supplement: Figure 1—figure supplement 2—source data 1. [file elife-86920-fig1-figsupp2-data1.zip › Figure 1-Figure Supplement 1 - Source Data 1/H_V725_1_strip_antiFLAG_1min.jpg]

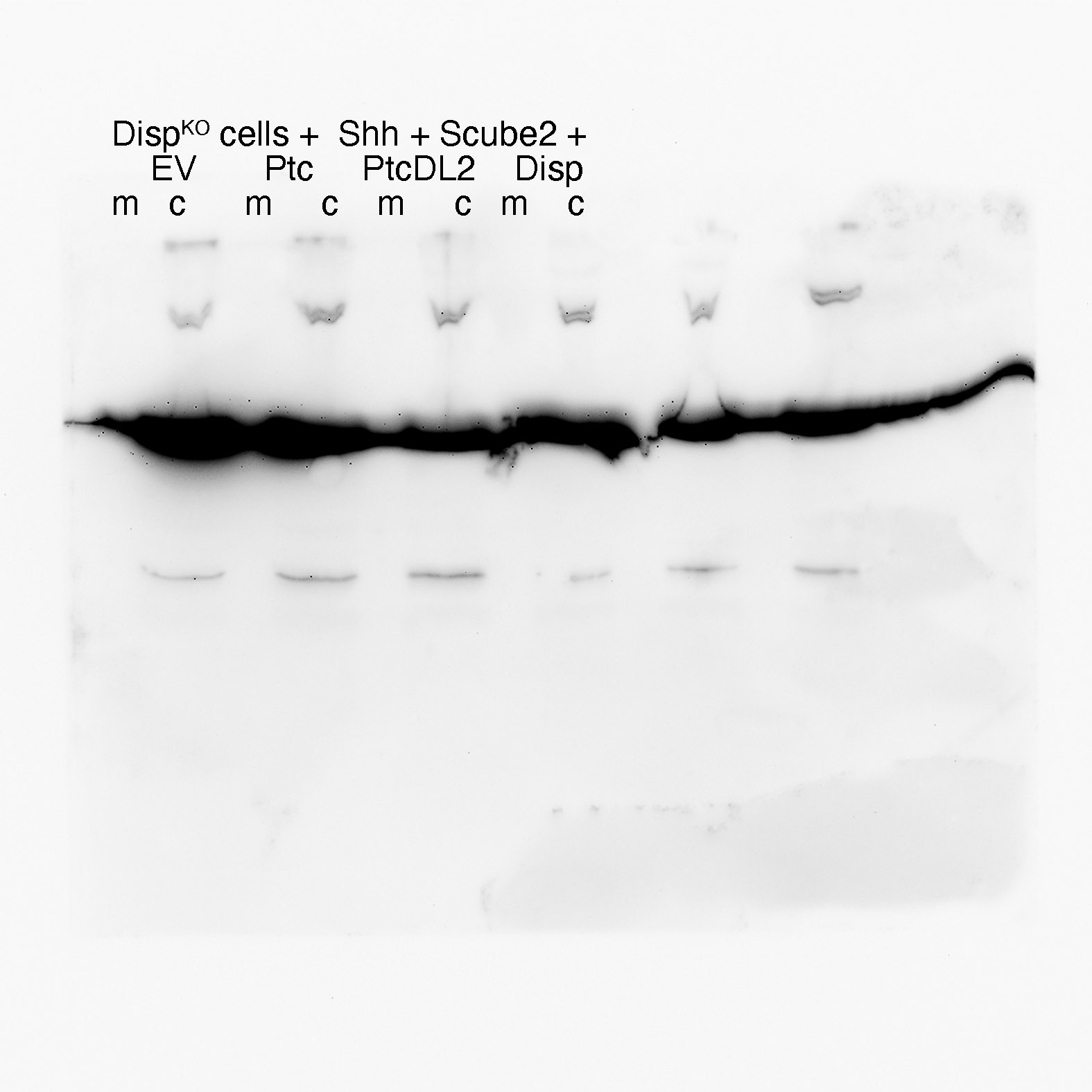

Supplement: Figure 1—figure supplement 2—source data 1. [file elife-86920-fig1-figsupp2-data1.zip › Figure 1-Figure Supplement 1 - Source Data 1/H_V725_Actin (1) labelled.jpg]

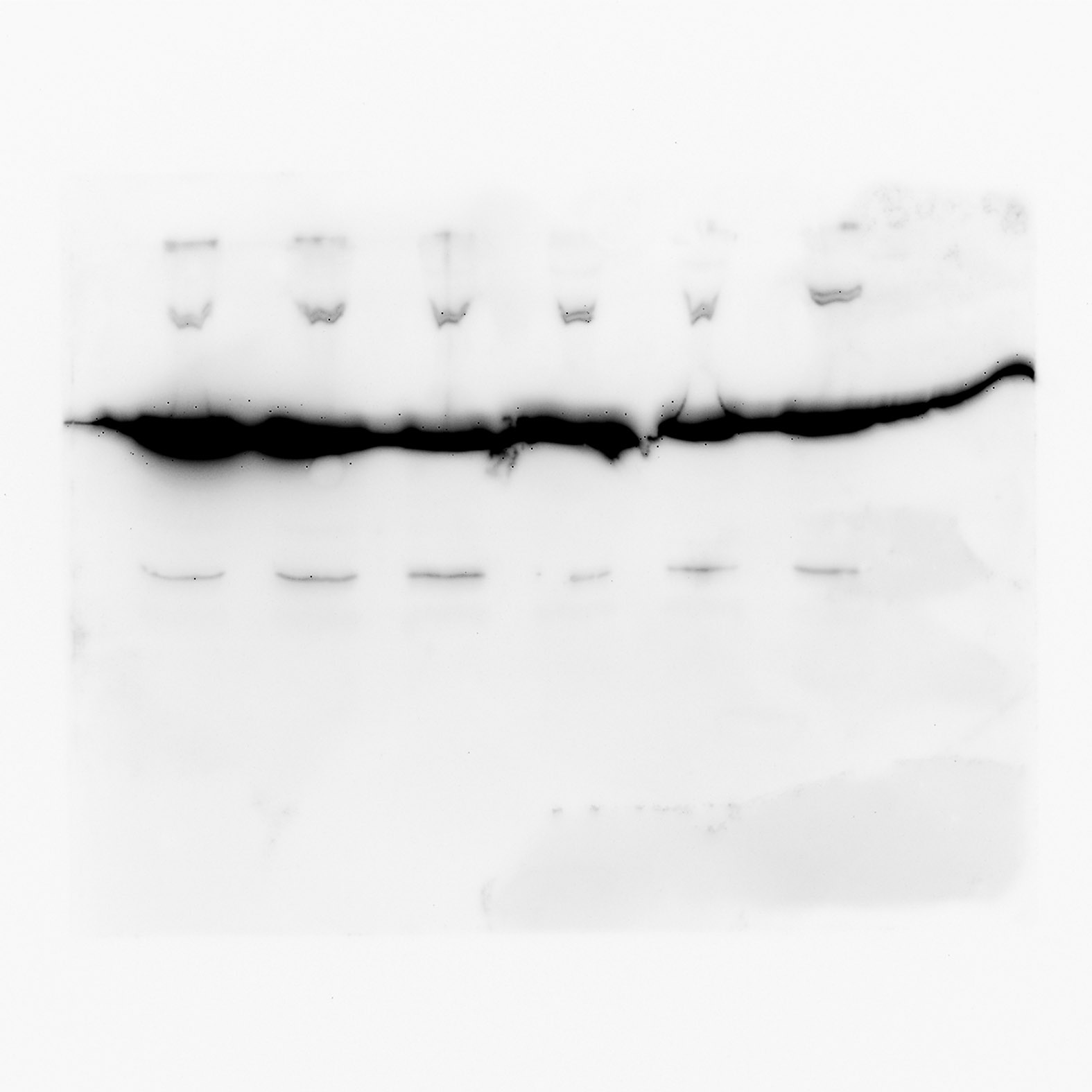

Supplement: Figure 1—figure supplement 2—source data 1. [file elife-86920-fig1-figsupp2-data1.zip › Figure 1-Figure Supplement 1 - Source Data 1/H_V725_Actin (1).jpg]

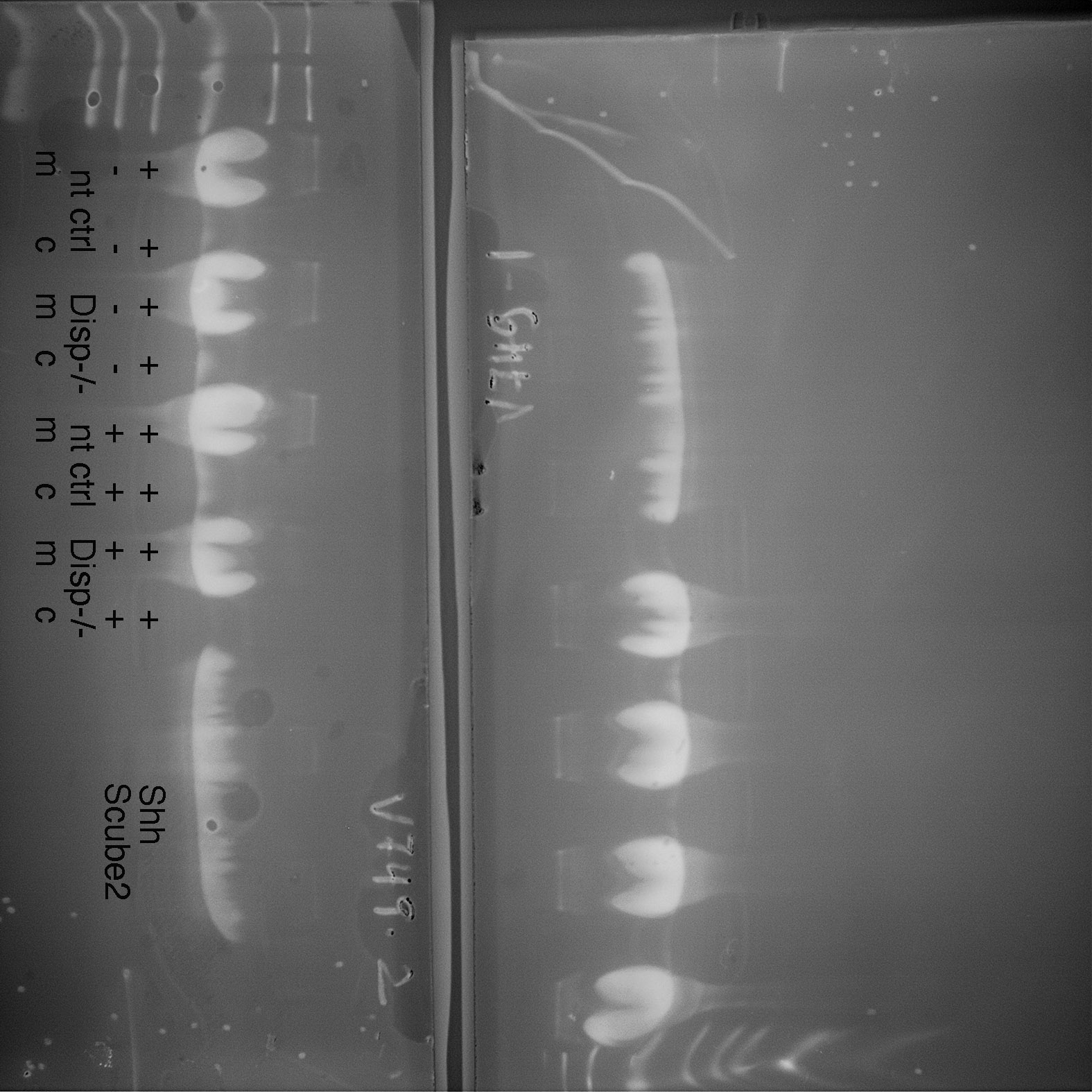

Supplement: Figure 1—figure supplement 2—source data 1. [file elife-86920-fig1-figsupp2-data1.zip › Figure 1-Figure Supplement 1 - Source Data 1/I_V749_1+2_Pon2 labelled.jpg]

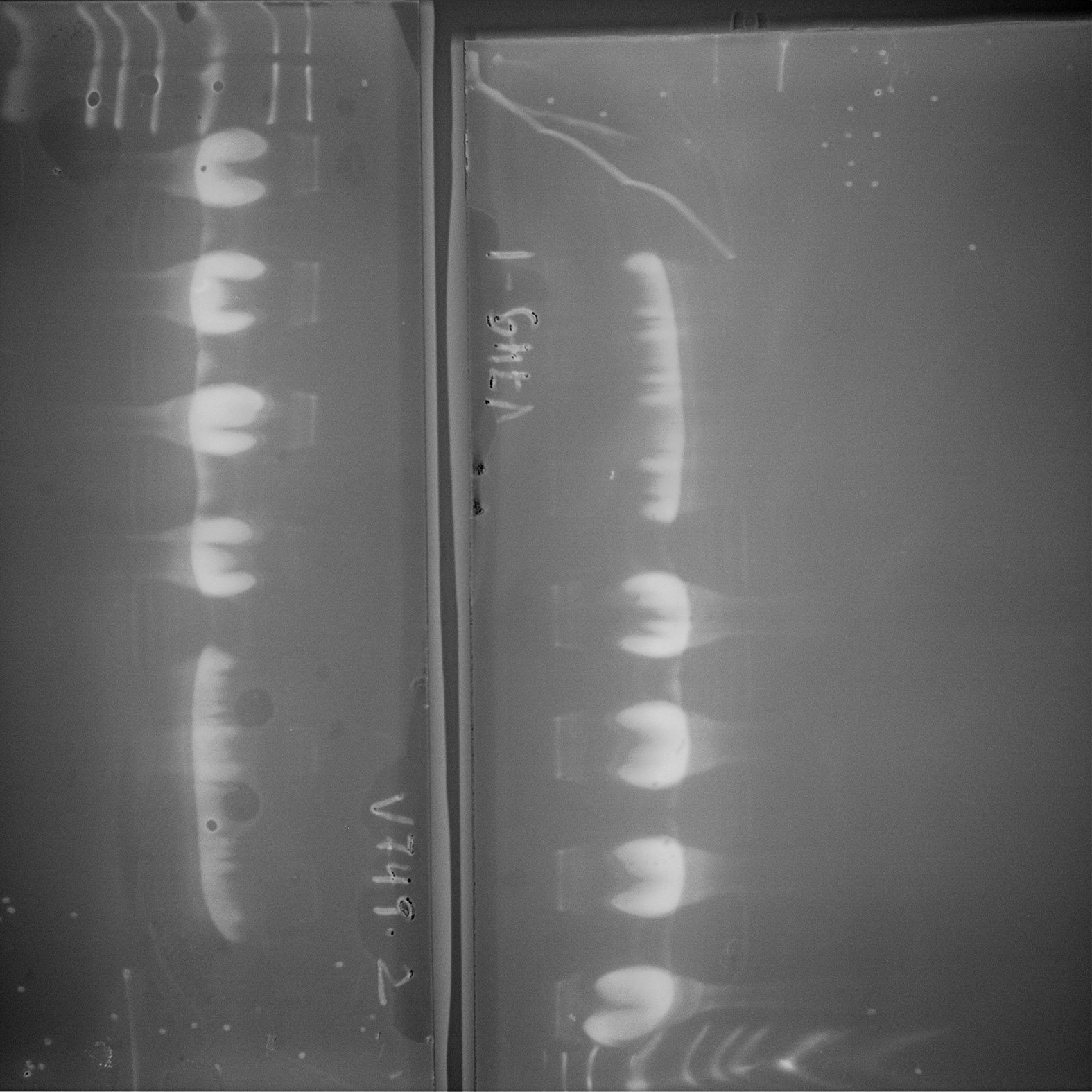

Supplement: Figure 1—figure supplement 2—source data 1. [file elife-86920-fig1-figsupp2-data1.zip › Figure 1-Figure Supplement 1 - Source Data 1/I_V749_1+2_Pon2.jpg]

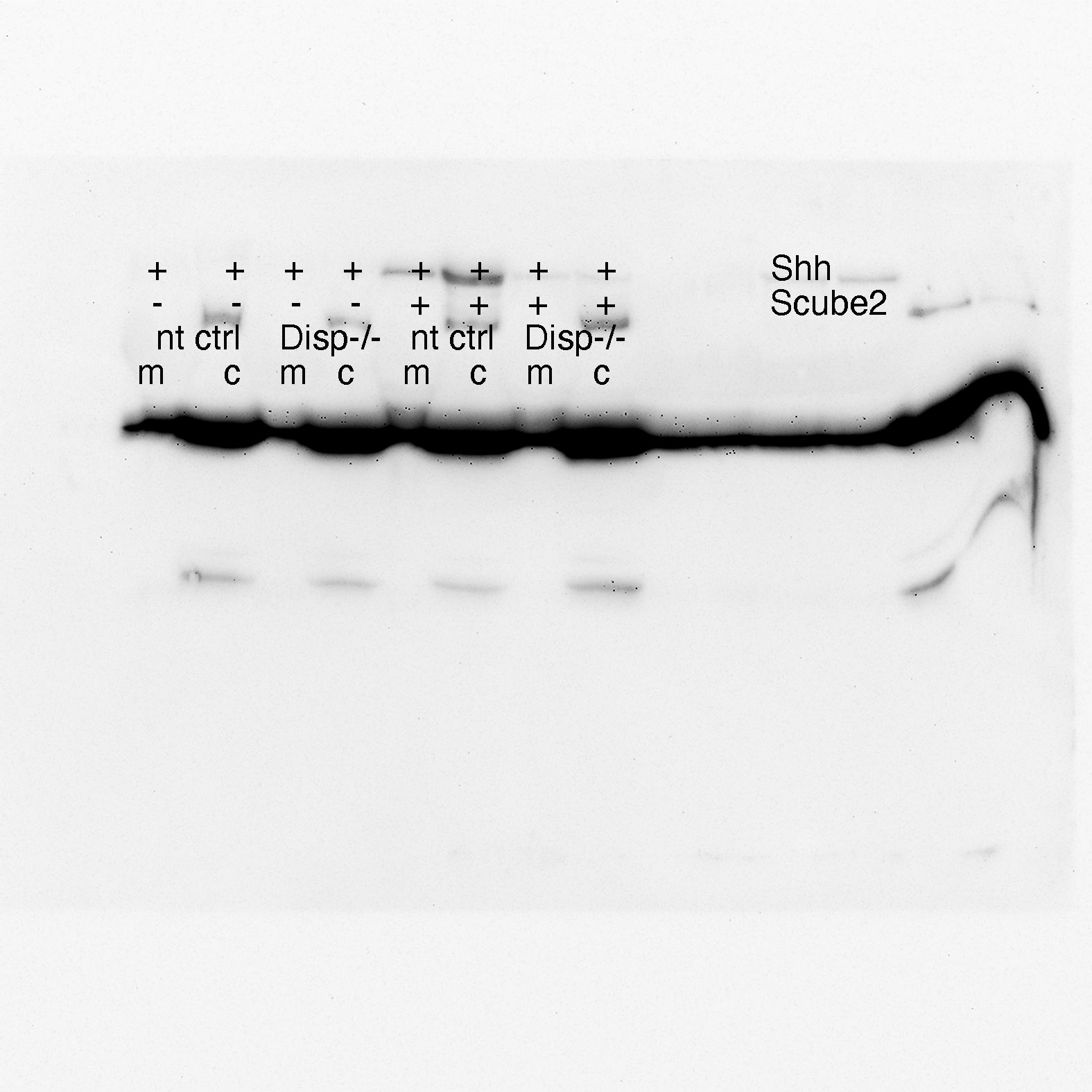

Supplement: Figure 1—figure supplement 2—source data 1. [file elife-86920-fig1-figsupp2-data1.zip › Figure 1-Figure Supplement 1 - Source Data 1/I_V749_2_act_300sec labelled.jpg]

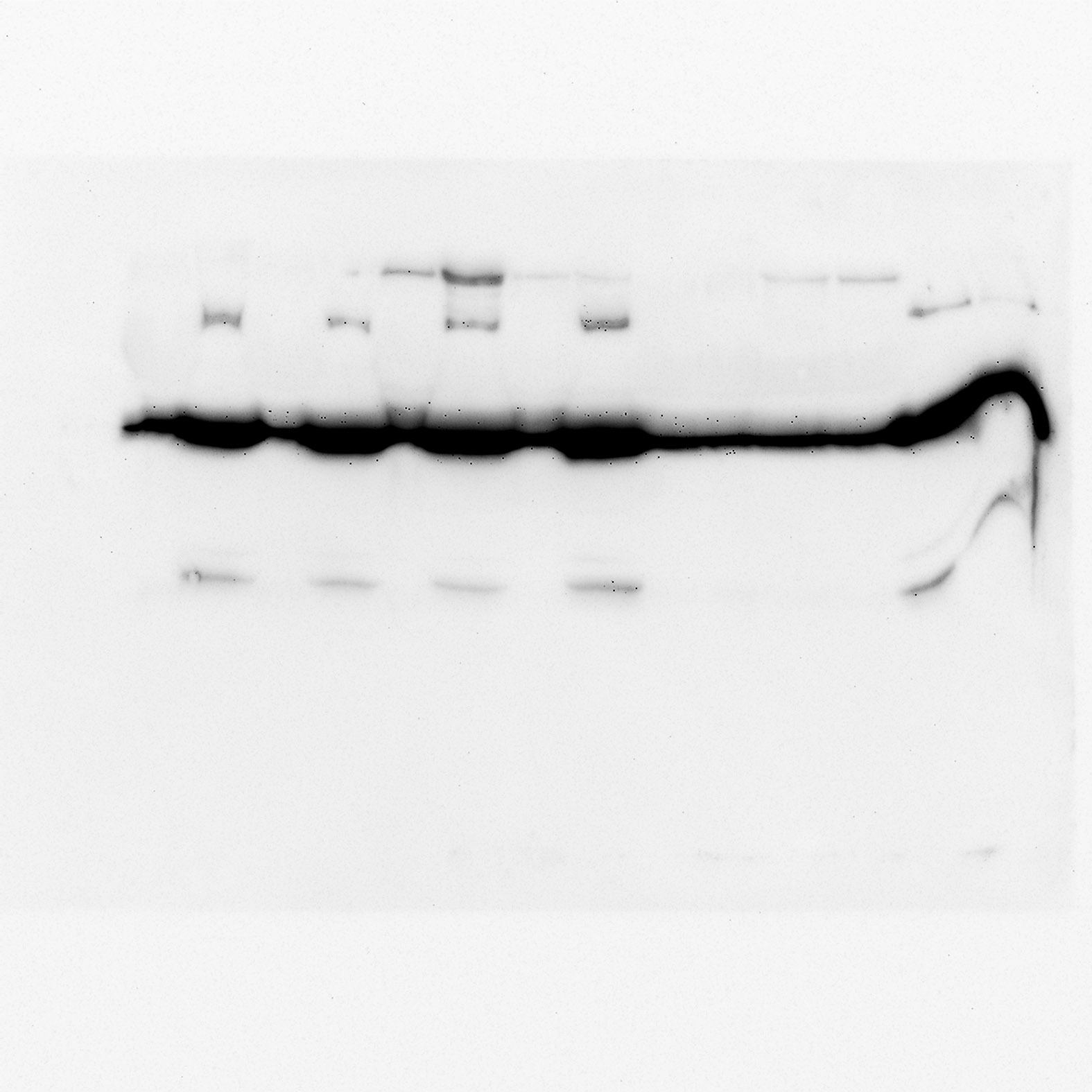

Supplement: Figure 1—figure supplement 2—source data 1. [file elife-86920-fig1-figsupp2-data1.zip › Figure 1-Figure Supplement 1 - Source Data 1/I_V749_2_act_300sec.jpg]

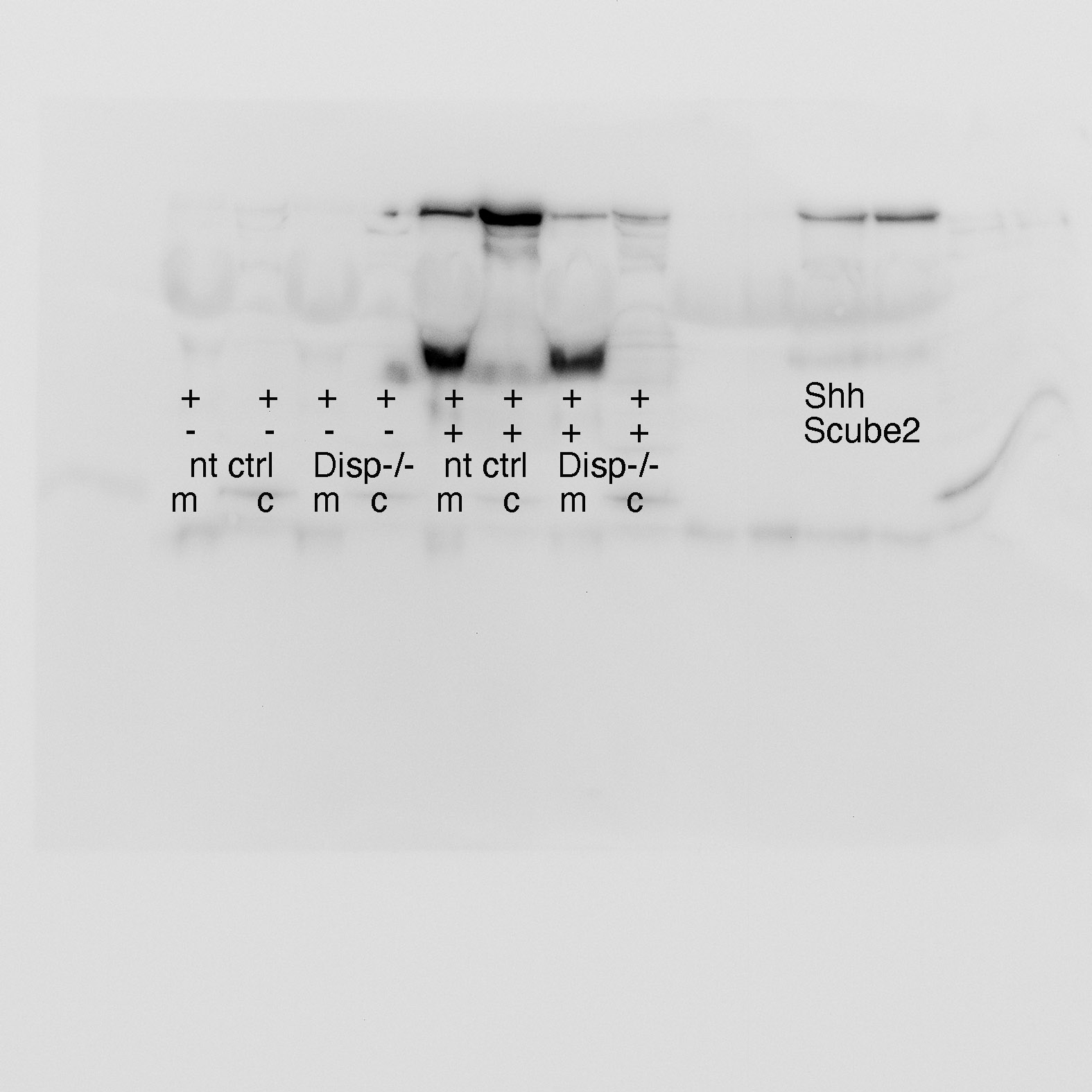

Supplement: Figure 1—figure supplement 2—source data 1. [file elife-86920-fig1-figsupp2-data1.zip › Figure 1-Figure Supplement 1 - Source Data 1/I_V749_2_Flag_30sec labelled.jpg]

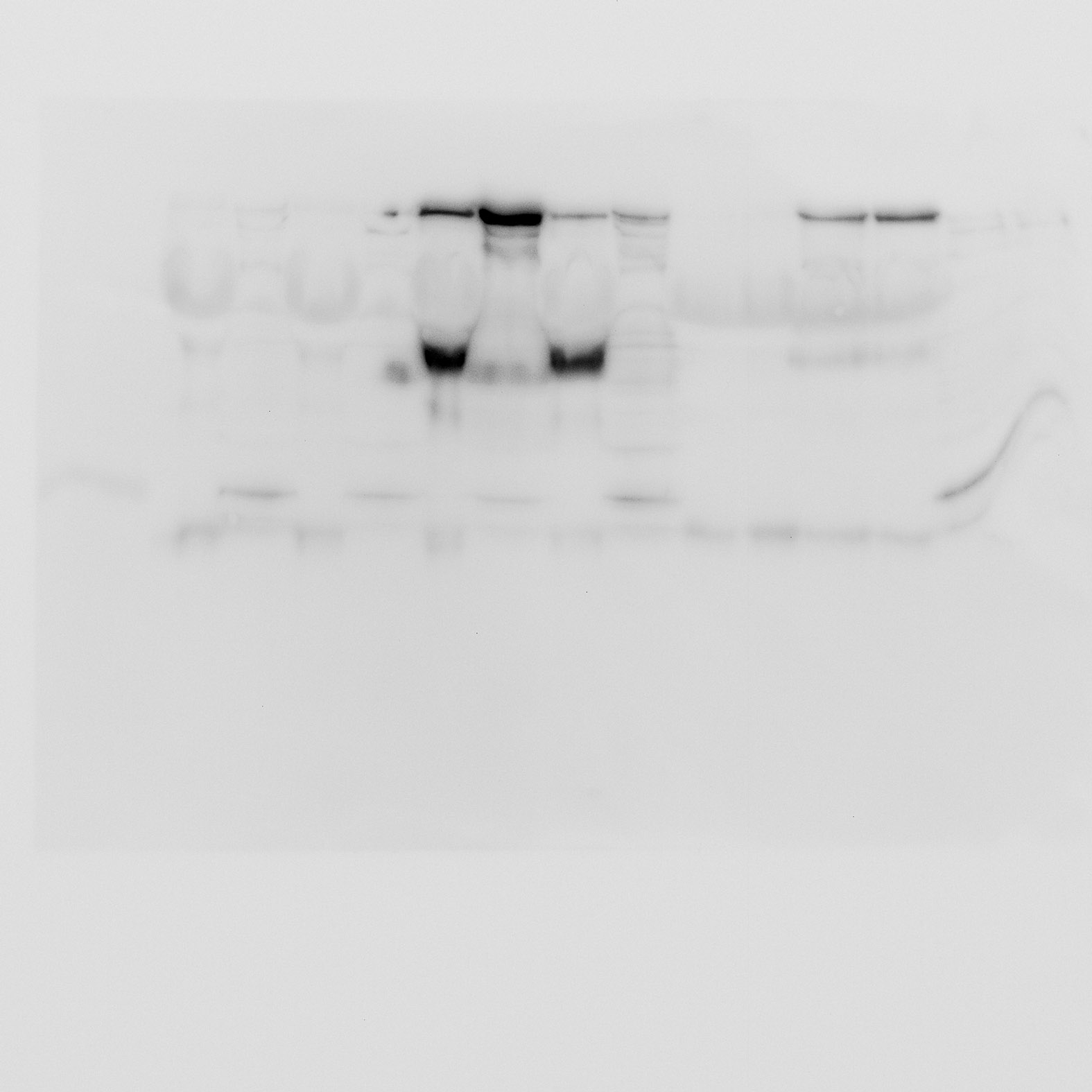

Supplement: Figure 1—figure supplement 2—source data 1. [file elife-86920-fig1-figsupp2-data1.zip › Figure 1-Figure Supplement 1 - Source Data 1/I_V749_2_Flag_30sec.jpg]

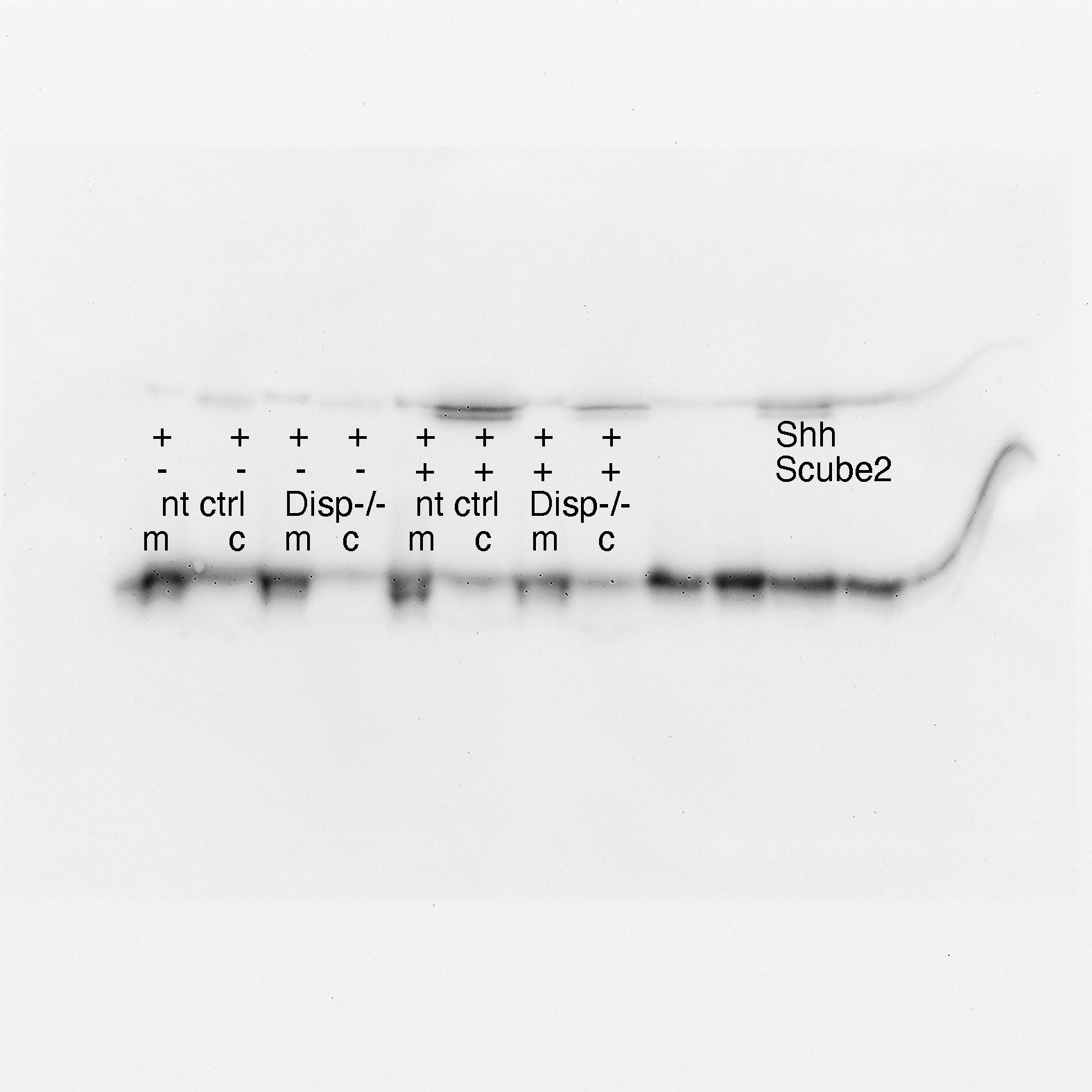

Supplement: Figure 1—figure supplement 2—source data 1. [file elife-86920-fig1-figsupp2-data1.zip › Figure 1-Figure Supplement 1 - Source Data 1/I_V749_2_Shh_2min labelled.jpg]

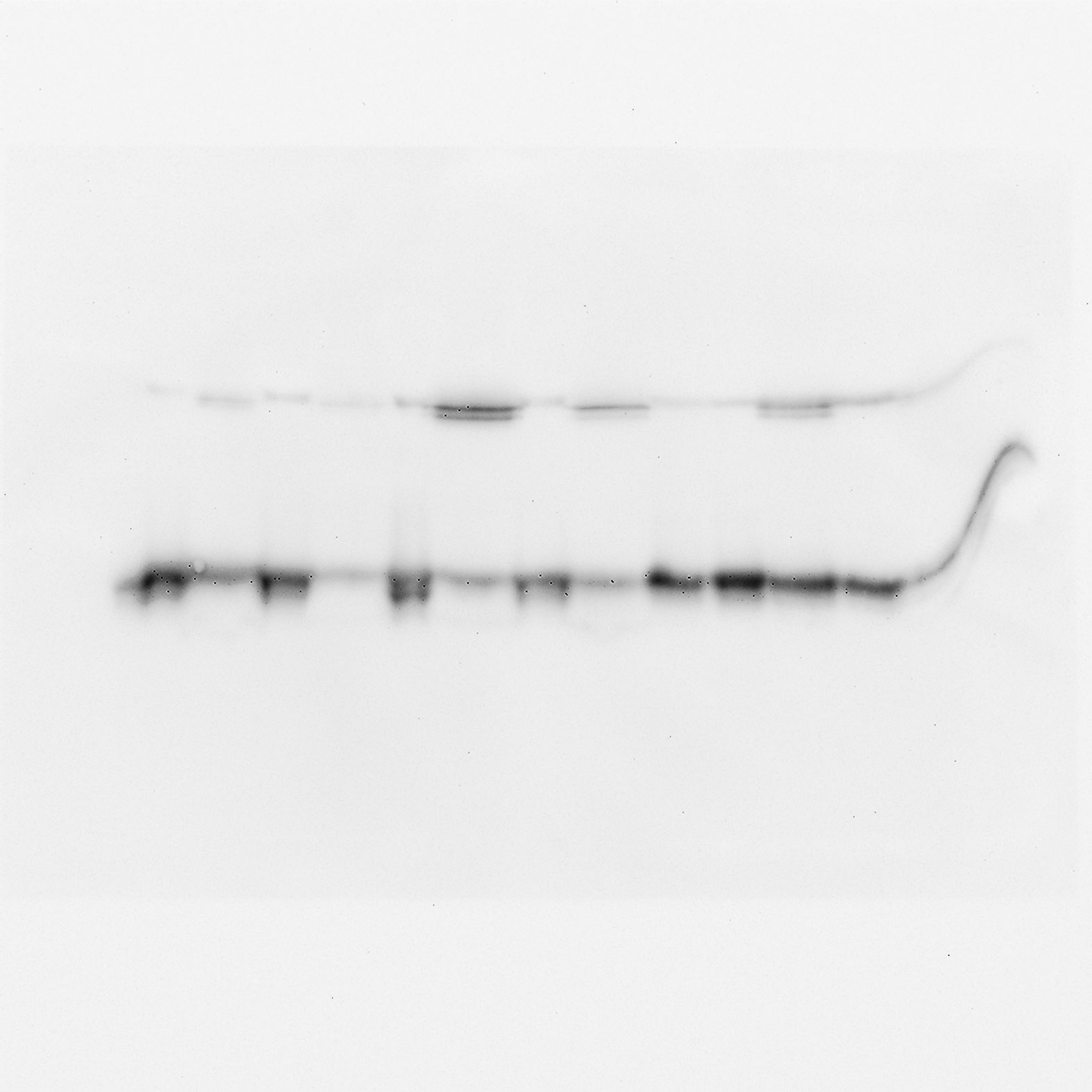

Supplement: Figure 1—figure supplement 2—source data 1. [file elife-86920-fig1-figsupp2-data1.zip › Figure 1-Figure Supplement 1 - Source Data 1/I_V749_2_Shh_2min.jpg]

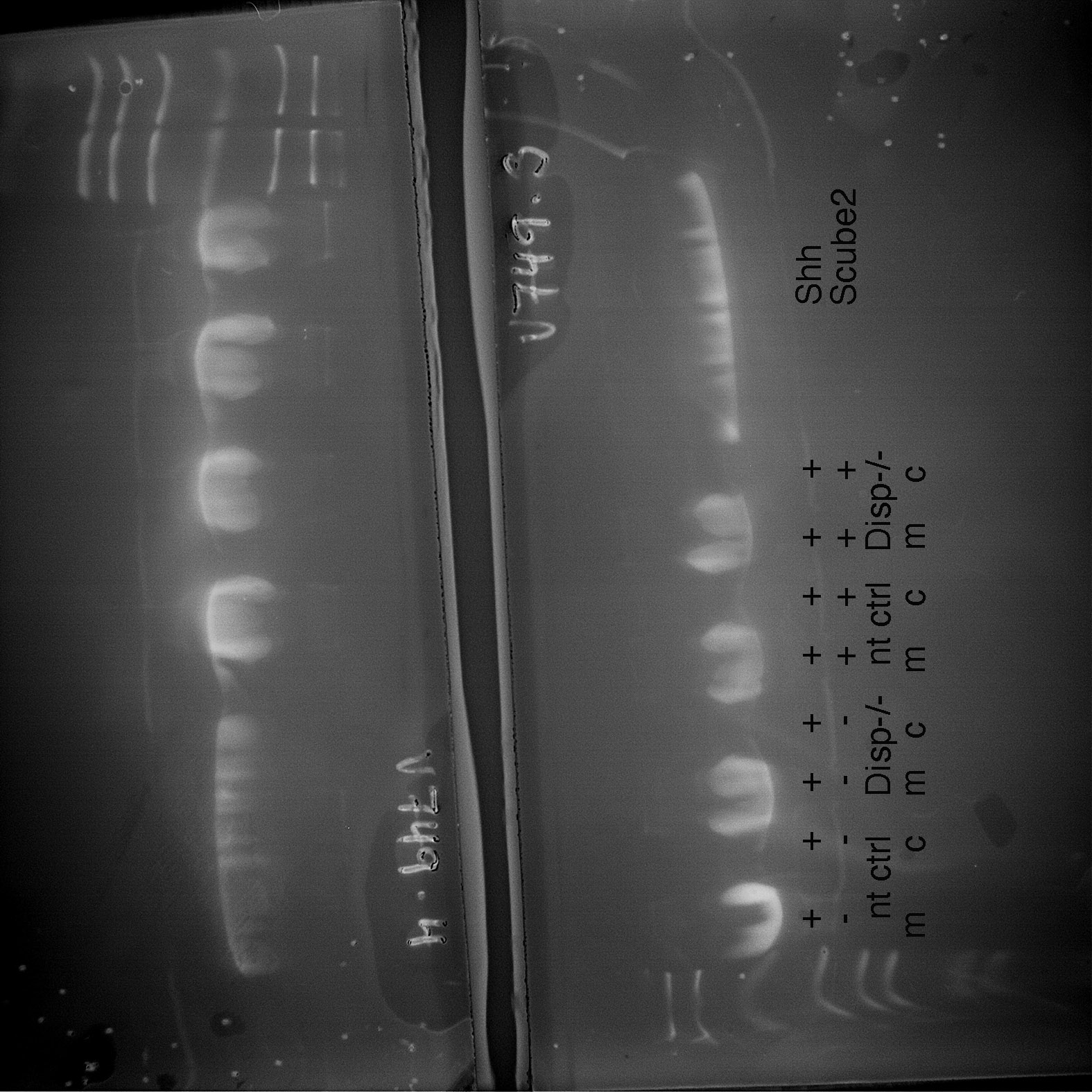

Supplement: Figure 1—figure supplement 2—source data 1. [file elife-86920-fig1-figsupp2-data1.zip › Figure 1-Figure Supplement 1 - Source Data 1/J_N_V749_3+4 fu╠êr H18 & S242_Pon1 labelled.jpg]

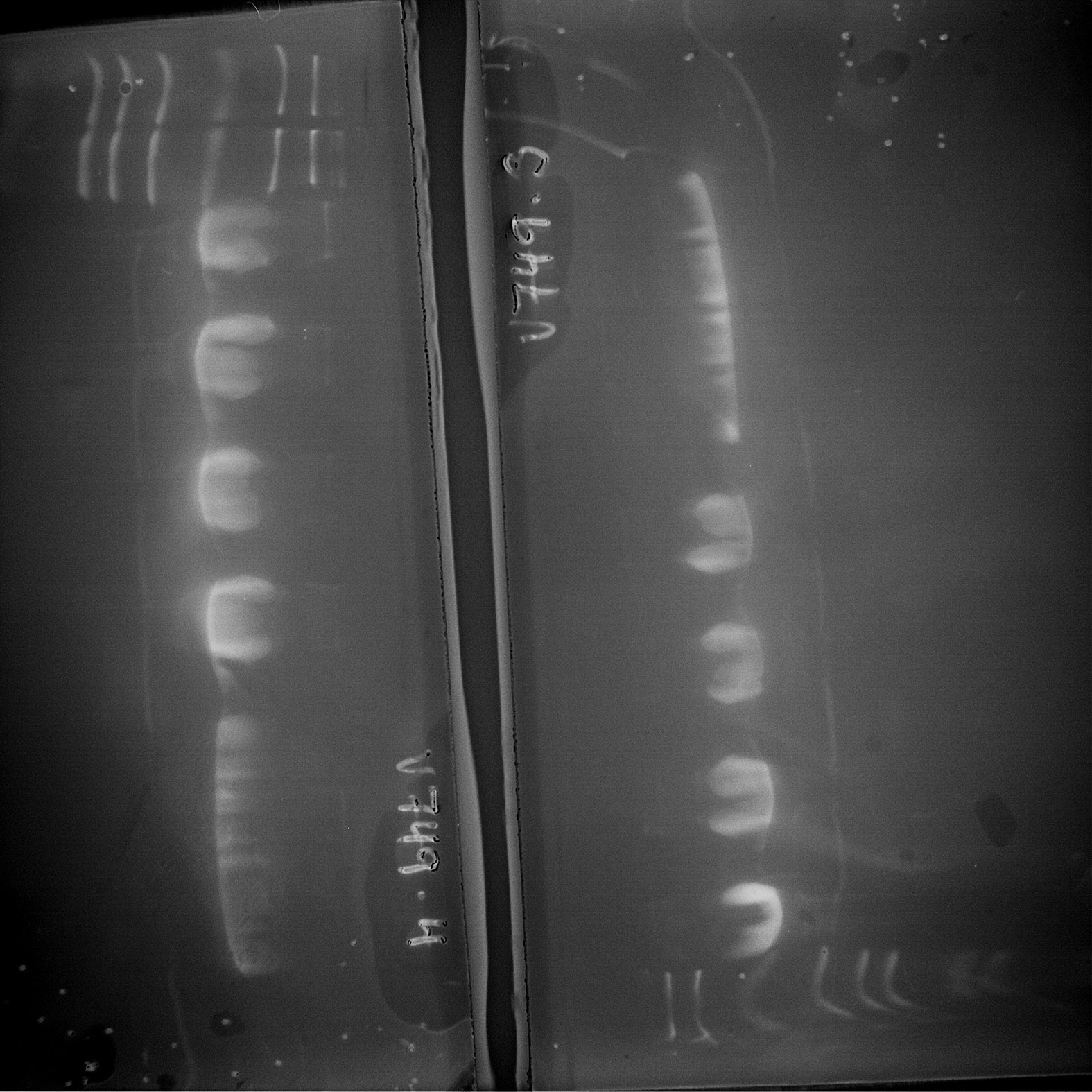

Supplement: Figure 1—figure supplement 2—source data 1. [file elife-86920-fig1-figsupp2-data1.zip › Figure 1-Figure Supplement 1 - Source Data 1/J_N_V749_3+4 fu╠êr H18 & S242_Pon1.jpg]

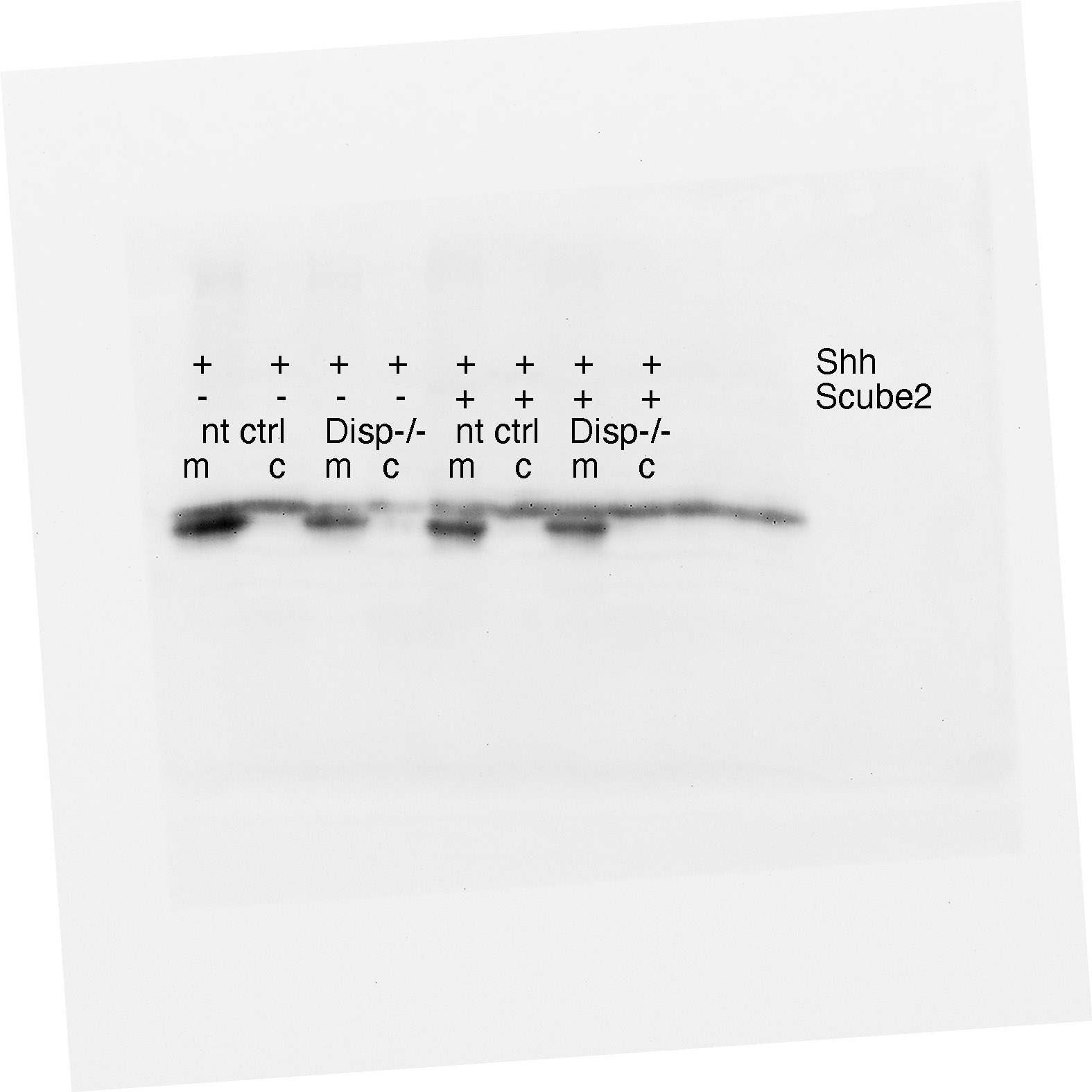

Supplement: Figure 1—figure supplement 2—source data 1. [file elife-86920-fig1-figsupp2-data1.zip › Figure 1-Figure Supplement 1 - Source Data 1/J_V757_3_0.5min_shh labelled.jpg]

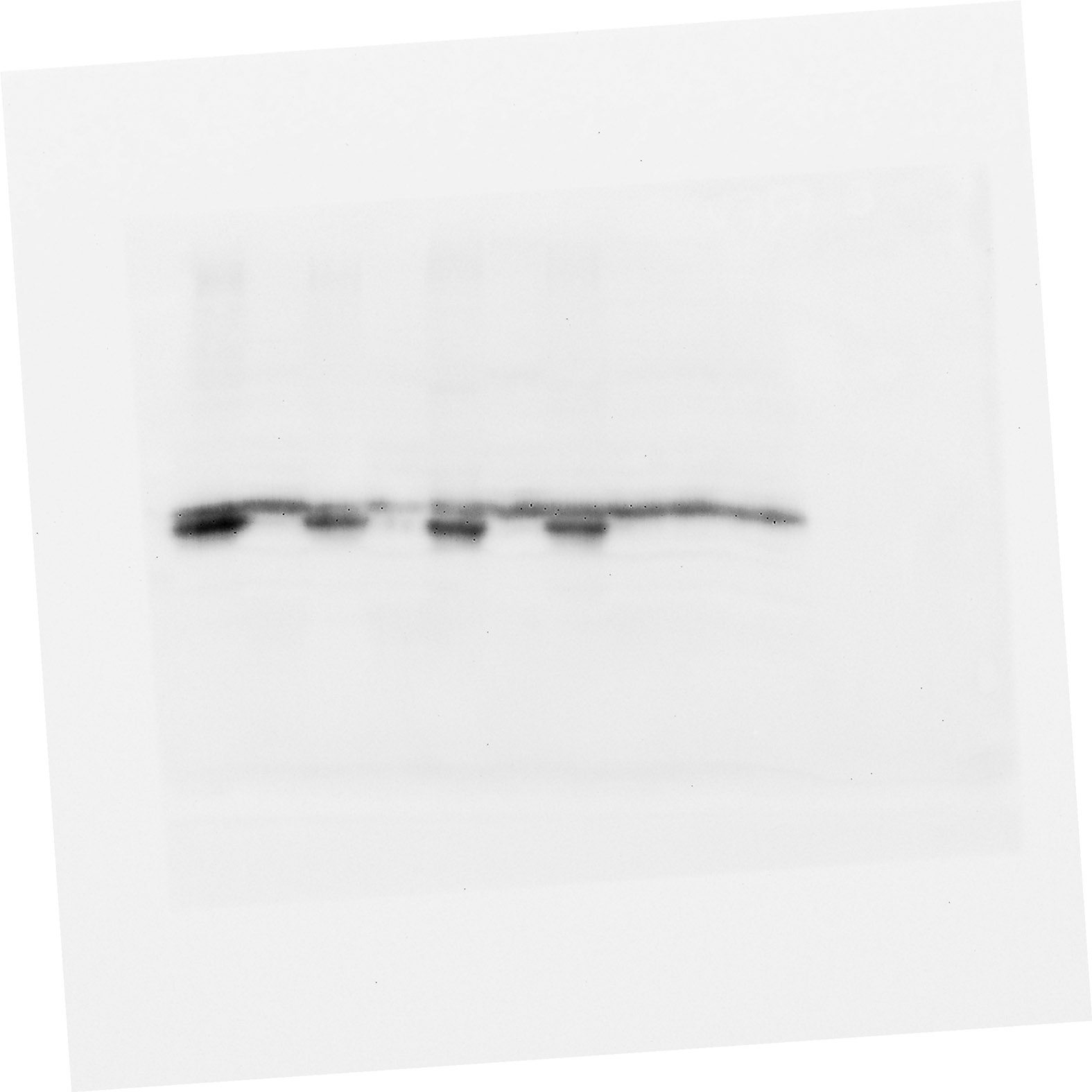

Supplement: Figure 1—figure supplement 2—source data 1. [file elife-86920-fig1-figsupp2-data1.zip › Figure 1-Figure Supplement 1 - Source Data 1/J_V757_3_0.5min_shh.jpg]

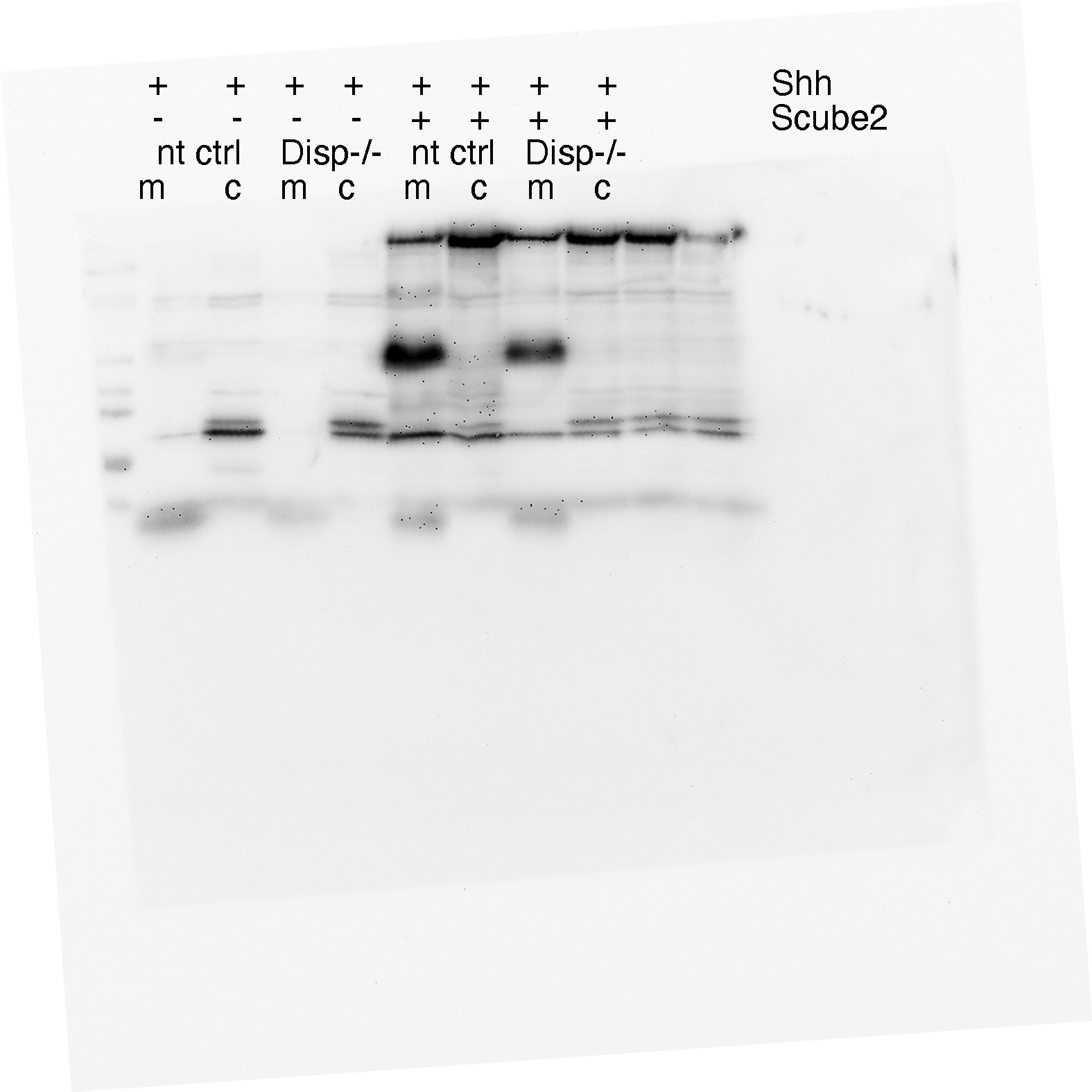

Supplement: Figure 1—figure supplement 2—source data 1. [file elife-86920-fig1-figsupp2-data1.zip › Figure 1-Figure Supplement 1 - Source Data 1/J_V757_3_Flag_1min labelled.jpg]

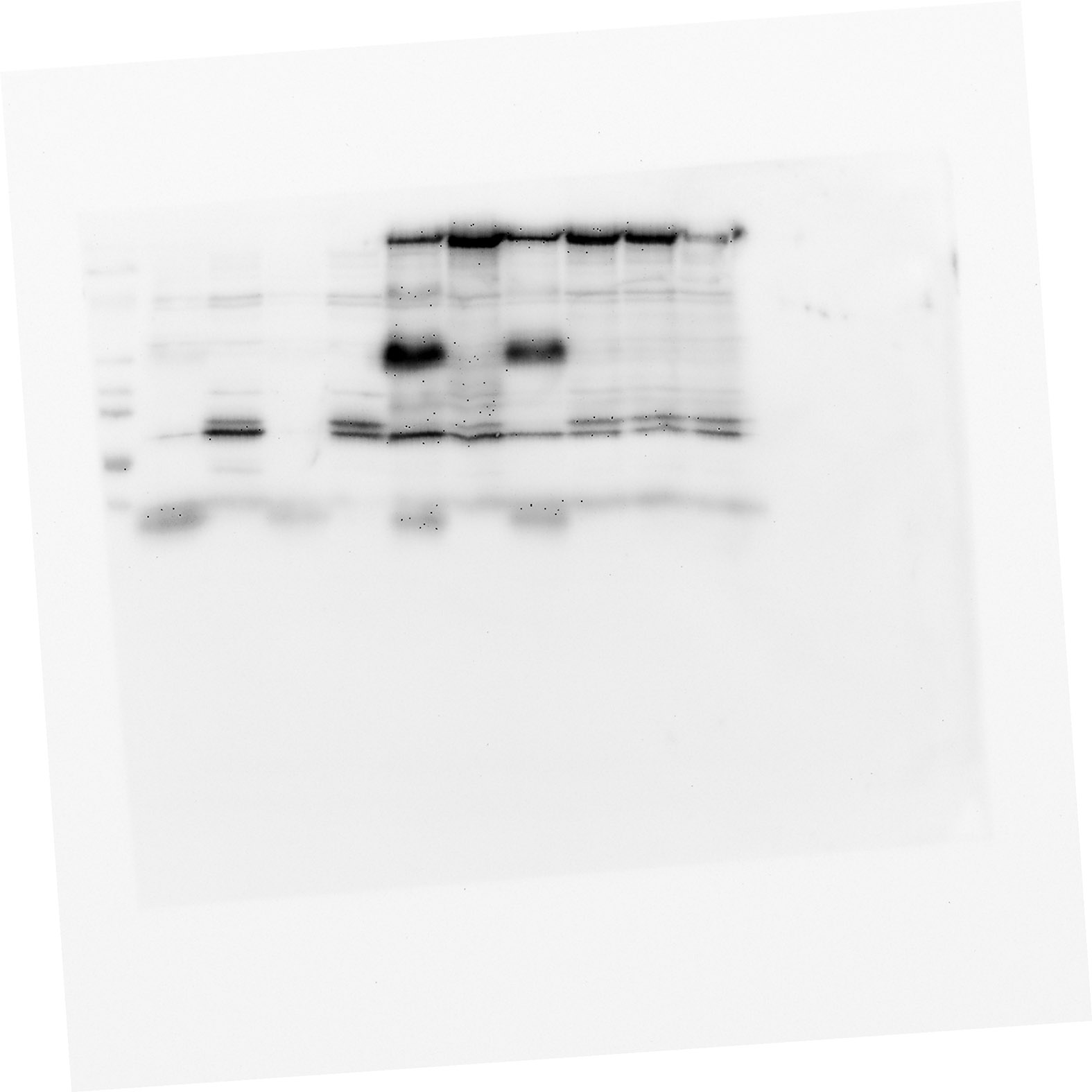

Supplement: Figure 1—figure supplement 2—source data 1. [file elife-86920-fig1-figsupp2-data1.zip › Figure 1-Figure Supplement 1 - Source Data 1/J_V757_3_Flag_1min.jpg]

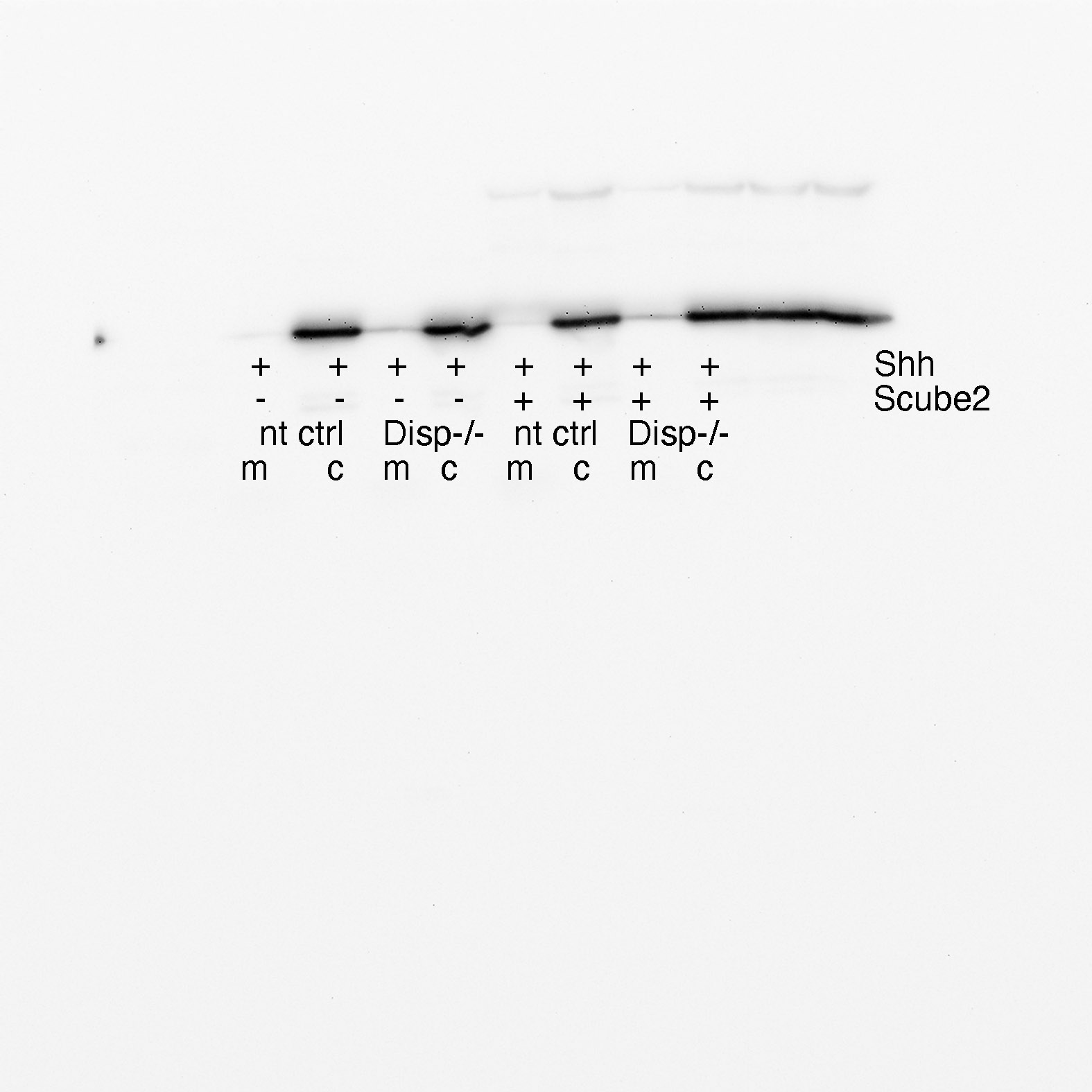

Supplement: Figure 1—figure supplement 2—source data 1. [file elife-86920-fig1-figsupp2-data1.zip › Figure 1-Figure Supplement 1 - Source Data 1/J_V757_actin_4-1min labelled.jpg]

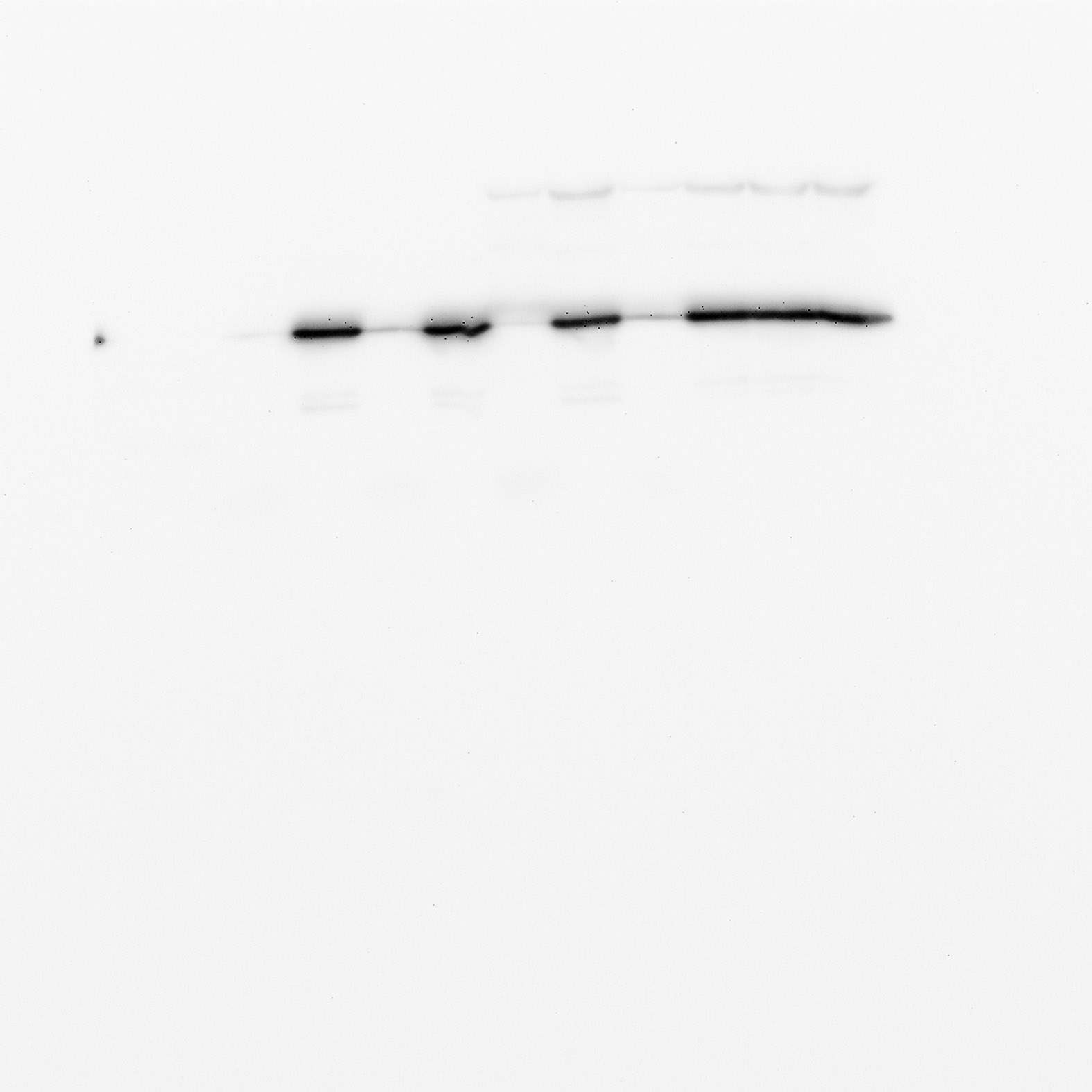

Supplement: Figure 1—figure supplement 2—source data 1. [file elife-86920-fig1-figsupp2-data1.zip › Figure 1-Figure Supplement 1 - Source Data 1/J_V757_actin_4-1min.jpg]

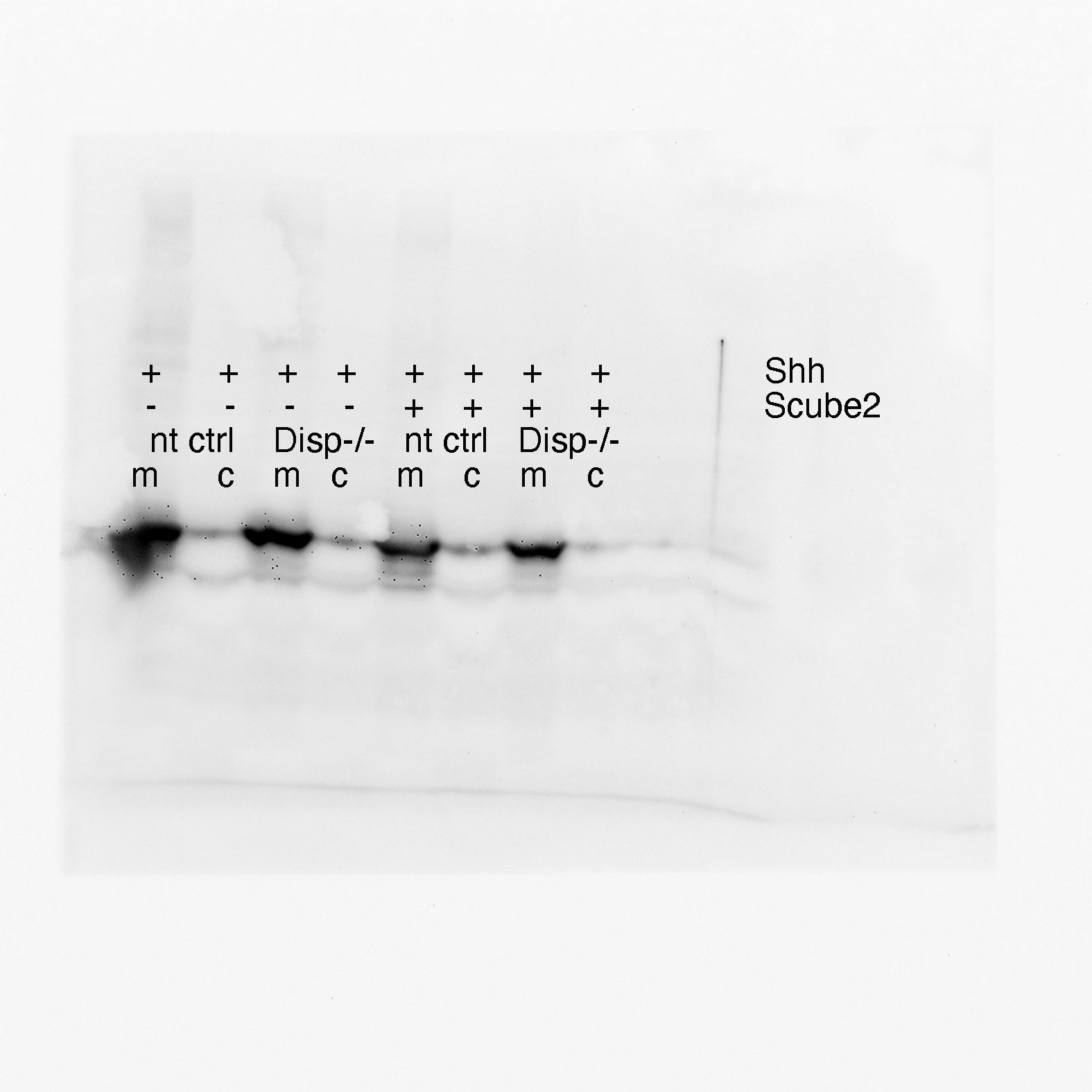

Supplement: Figure 1—figure supplement 2—source data 1. [file elife-86920-fig1-figsupp2-data1.zip › Figure 1-Figure Supplement 1 - Source Data 1/N_V757_6_0.5min_aShh labelled.jpg]

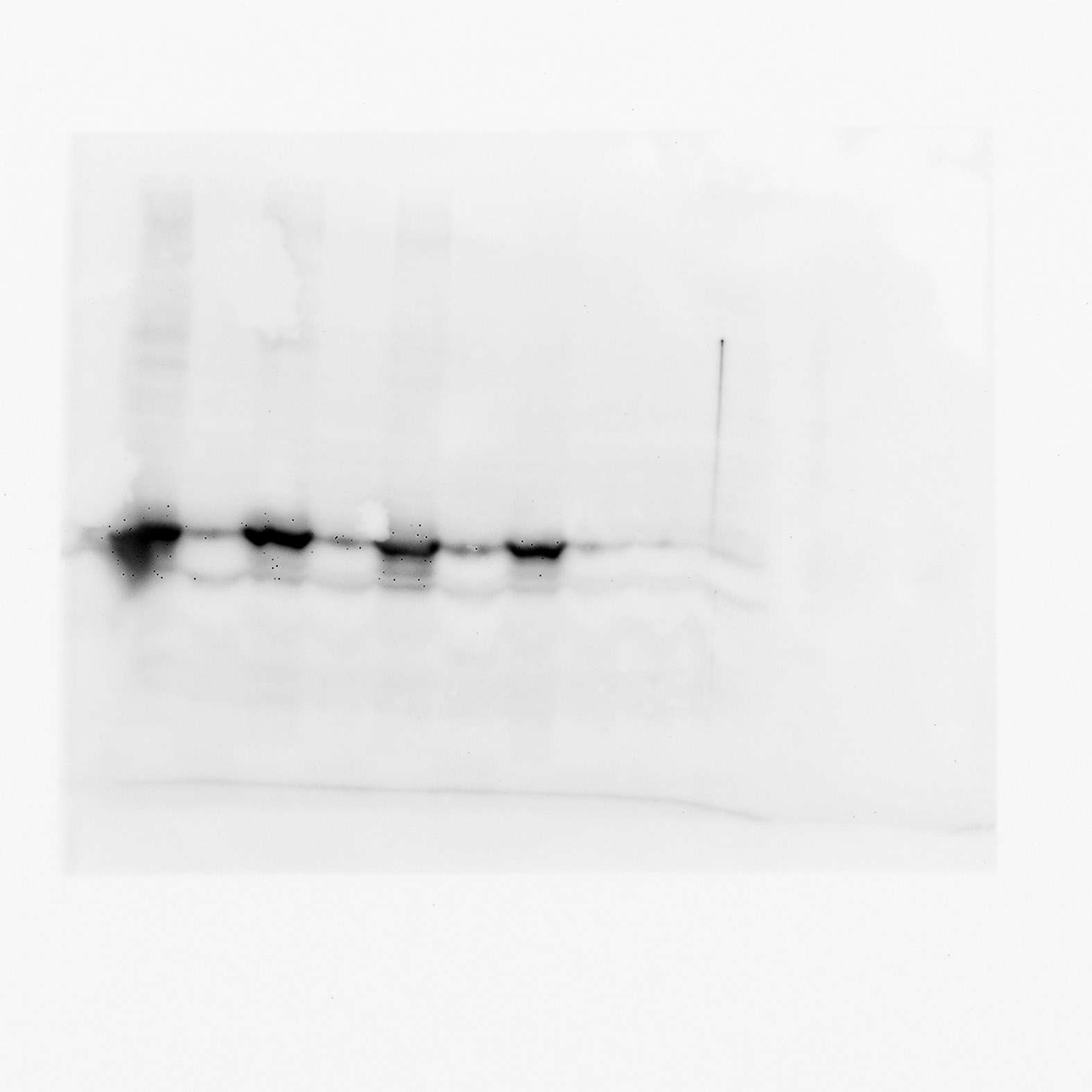

Supplement: Figure 1—figure supplement 2—source data 1. [file elife-86920-fig1-figsupp2-data1.zip › Figure 1-Figure Supplement 1 - Source Data 1/N_V757_6_0.5min_aShh.jpg]

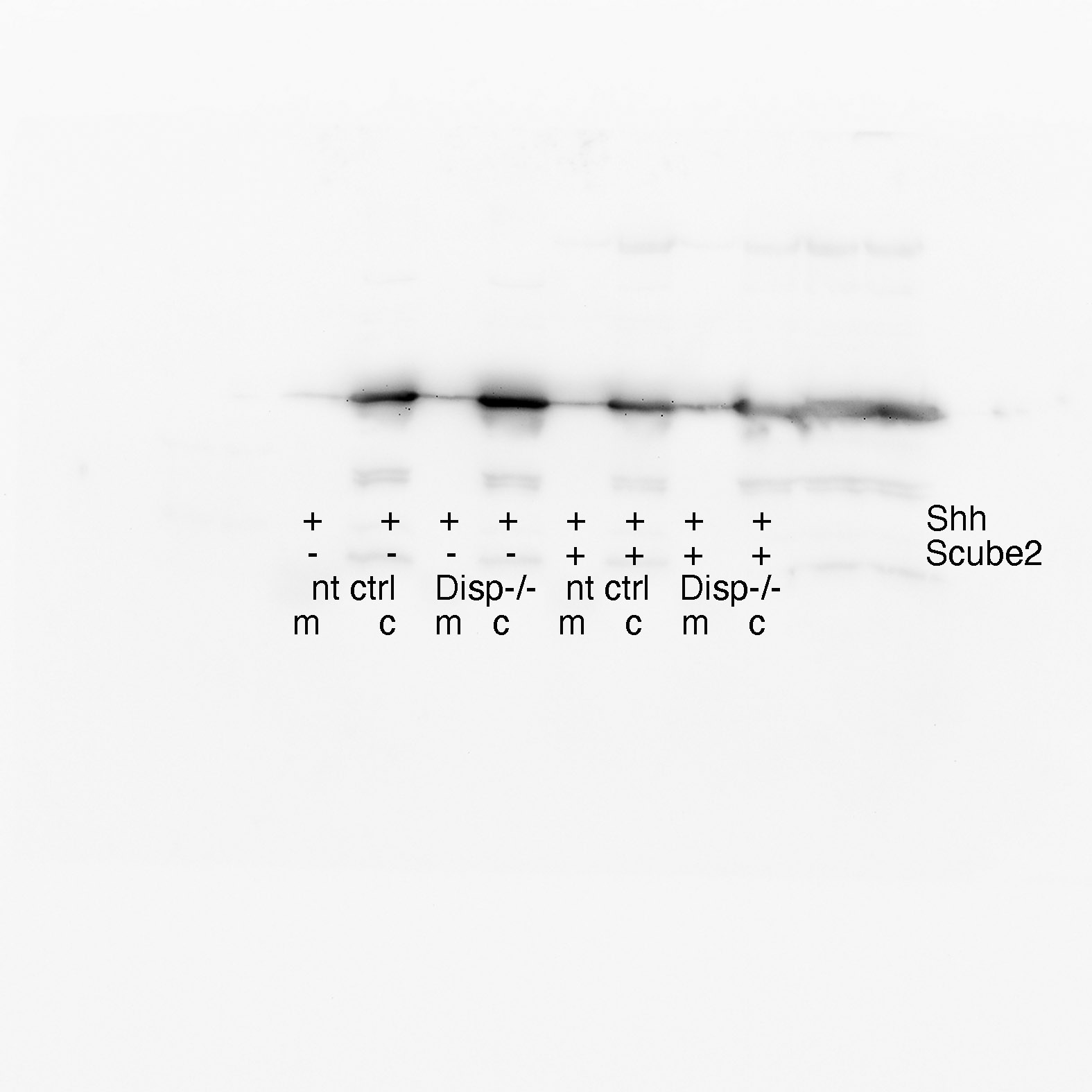

Supplement: Figure 1—figure supplement 2—source data 1. [file elife-86920-fig1-figsupp2-data1.zip › Figure 1-Figure Supplement 1 - Source Data 1/N_V757_7_actin_top15sec labelled.jpg]

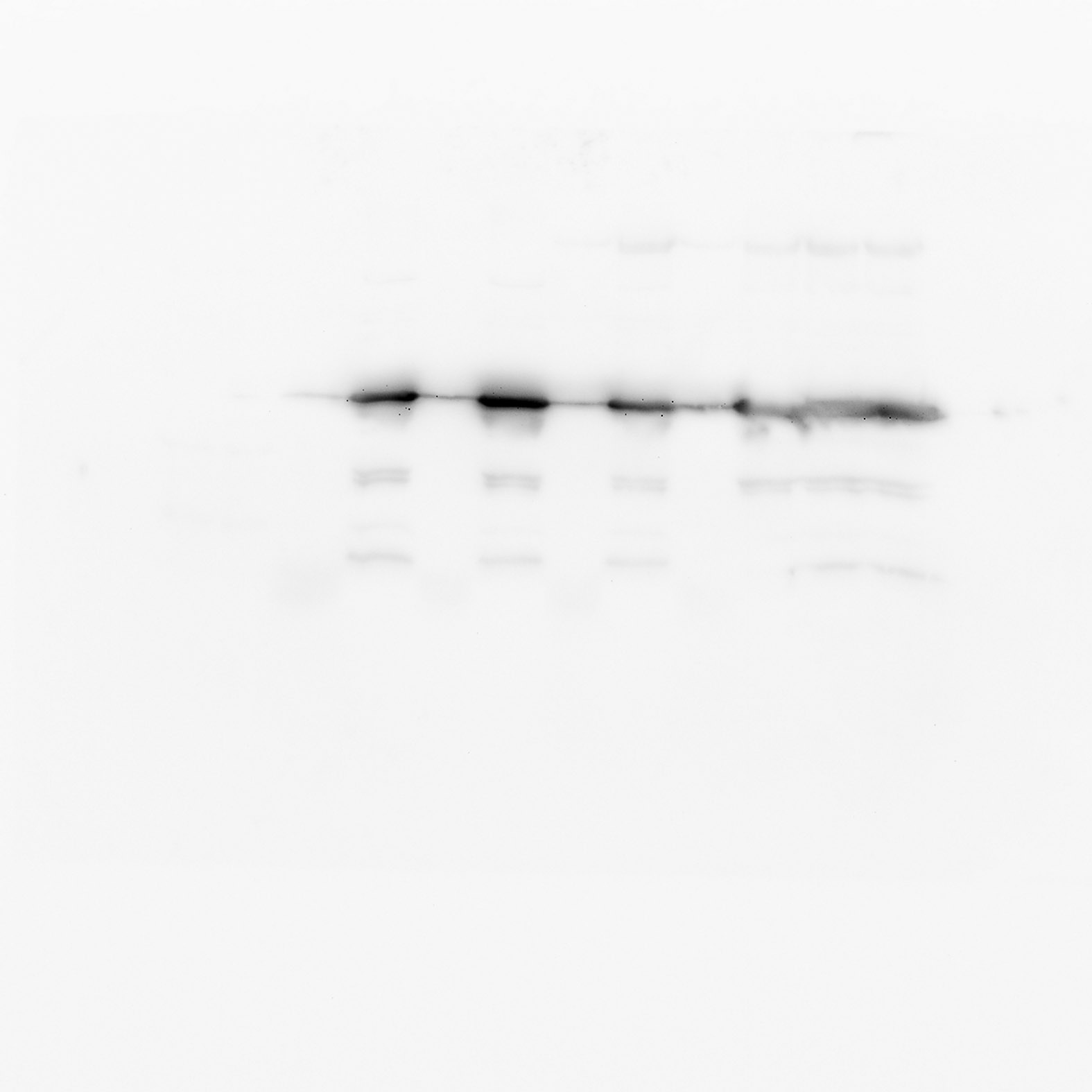

Supplement: Figure 1—figure supplement 2—source data 1. [file elife-86920-fig1-figsupp2-data1.zip › Figure 1-Figure Supplement 1 - Source Data 1/N_V757_7_actin_top15sec.jpg]

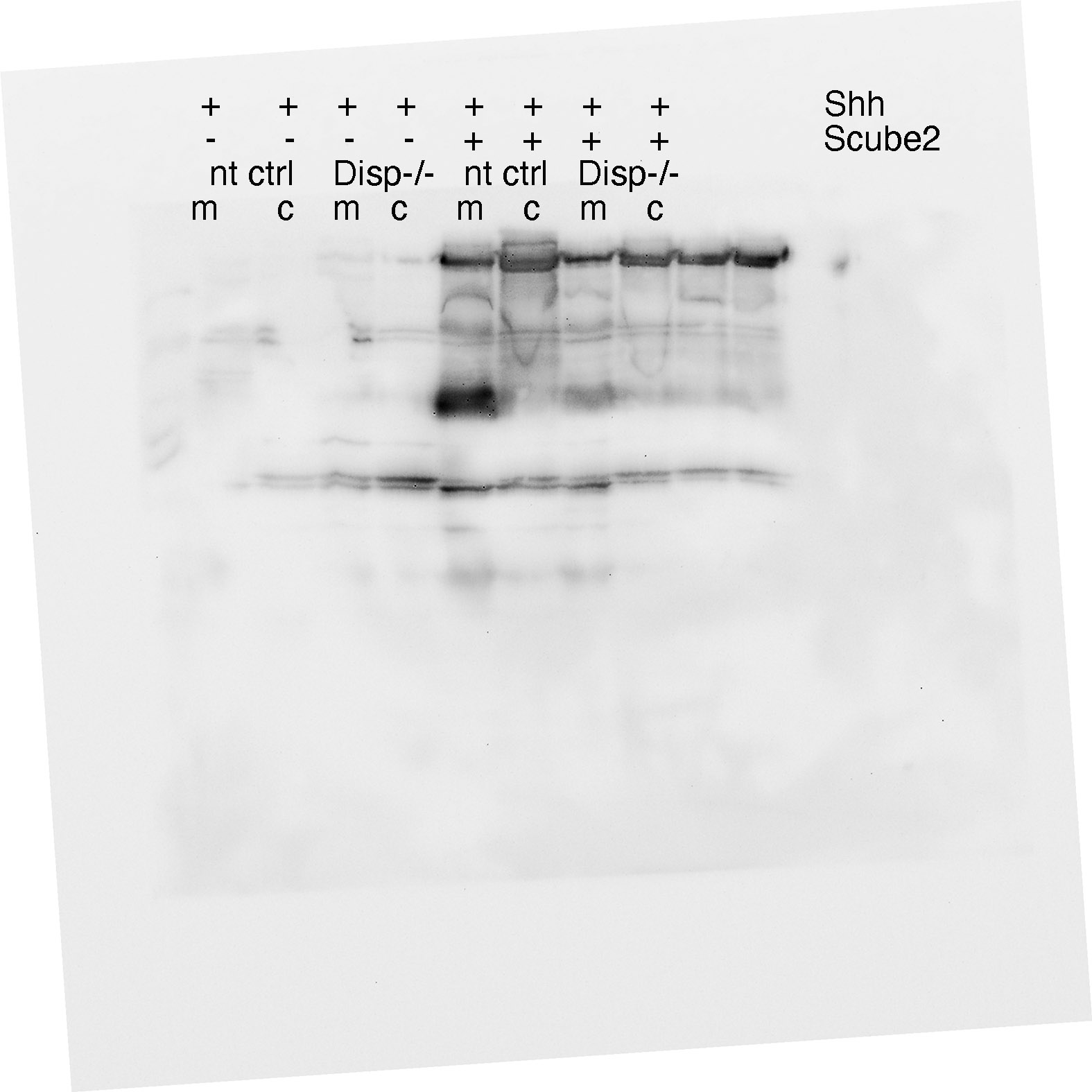

Supplement: Figure 1—figure supplement 2—source data 1. [file elife-86920-fig1-figsupp2-data1.zip › Figure 1-Figure Supplement 1 - Source Data 1/N_V757_Flag_6_20sec labelled.jpg]

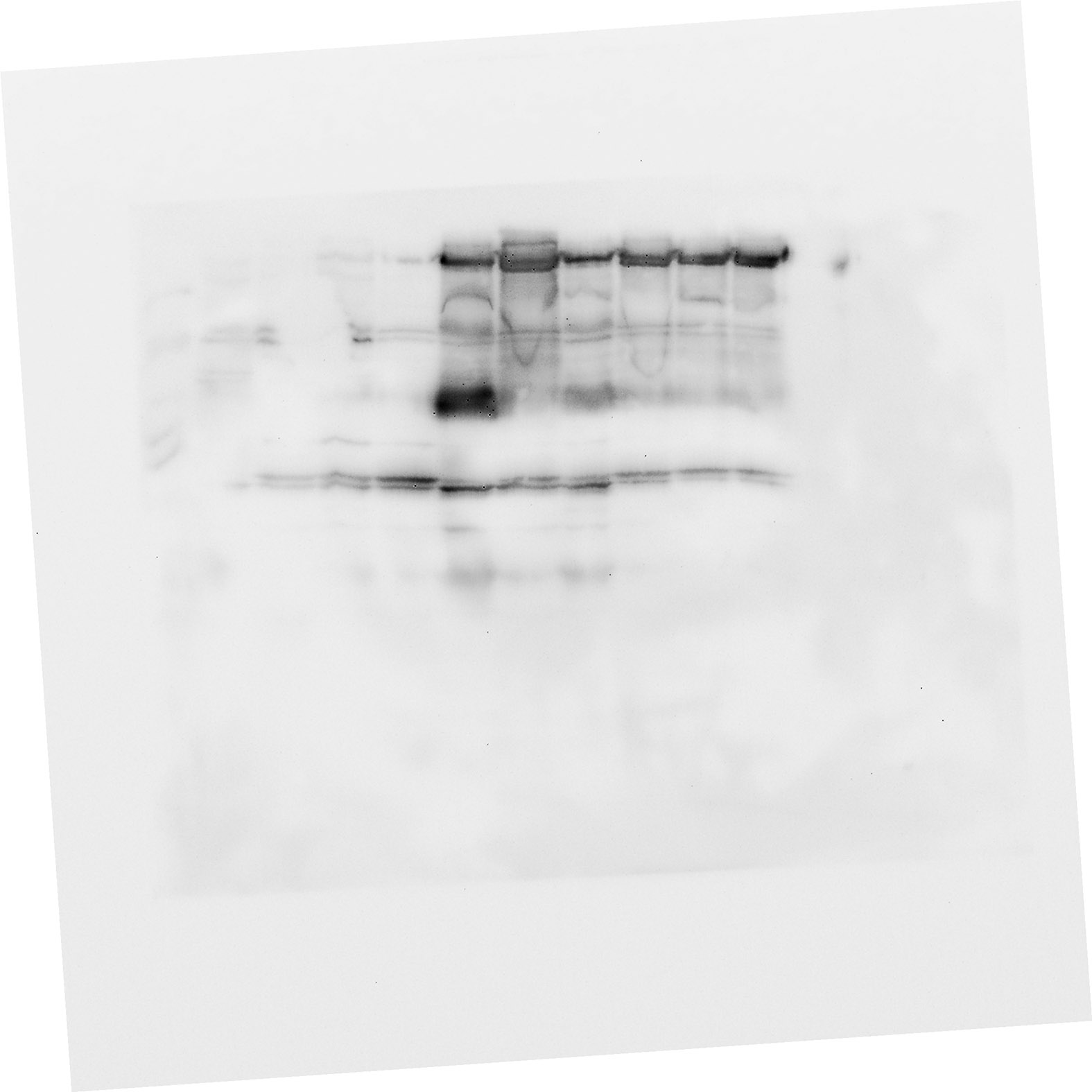

Supplement: Figure 1—figure supplement 2—source data 1. [file elife-86920-fig1-figsupp2-data1.zip › Figure 1-Figure Supplement 1 - Source Data 1/N_V757_Flag_6_20sec.jpg]

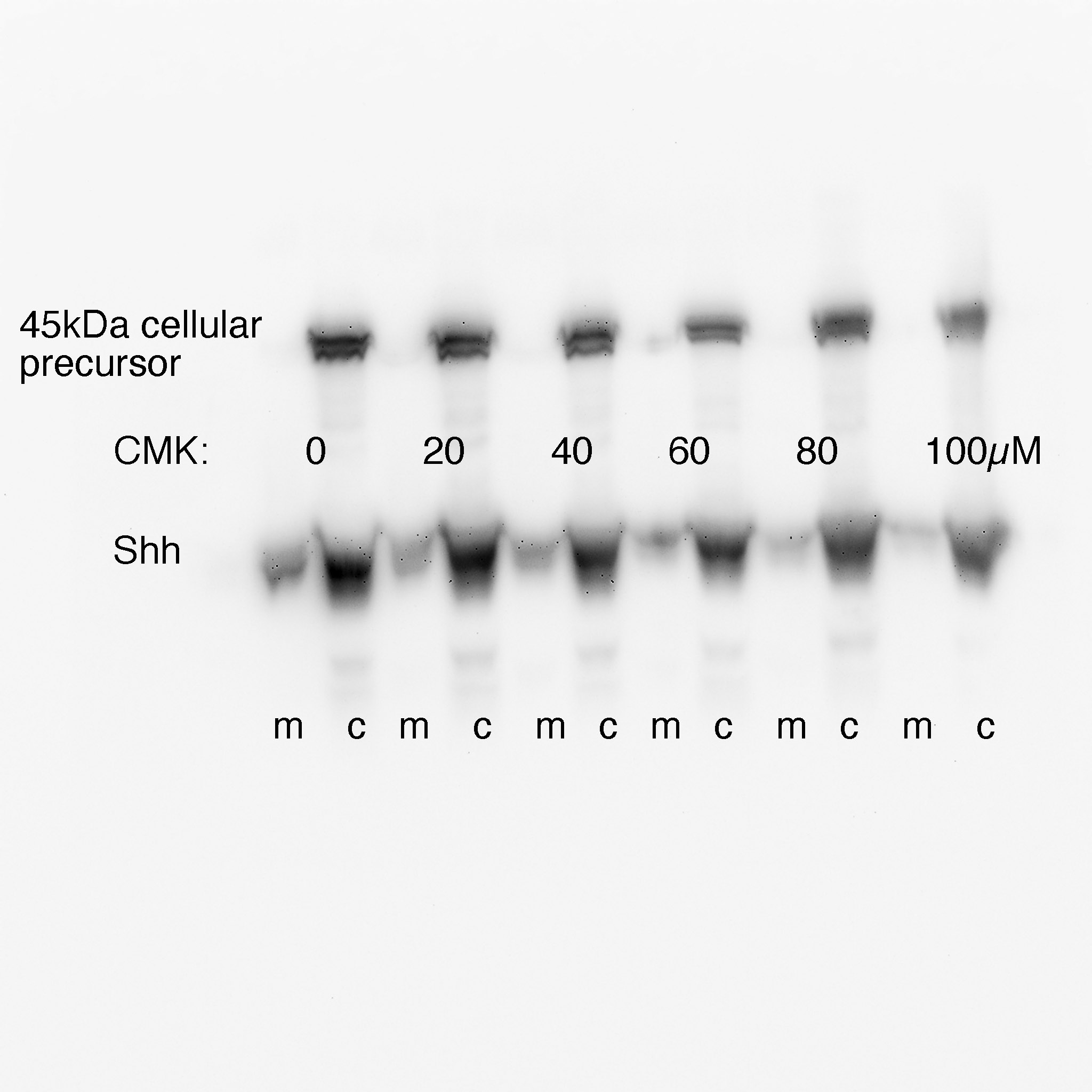

Supplement: Figure 2—source data 1. — A, B, D contain uncropped western blots of data shown in Figure 2A, B and D. C shows three additional representative examples of CMK-inhibited Shh release. E shows six biological replicates of impaired Shh release in the absence of serum that were quantified and displayed in Figure 2E. Prizm files C and E quantify relative Shh release rates based on the data shown in tiff-files B + C and E. [file elife-86920-fig2-data1.zip › Figure_2_Source_Data_1 /A_VP025_Furin_Inhibitor labelled.jpg]

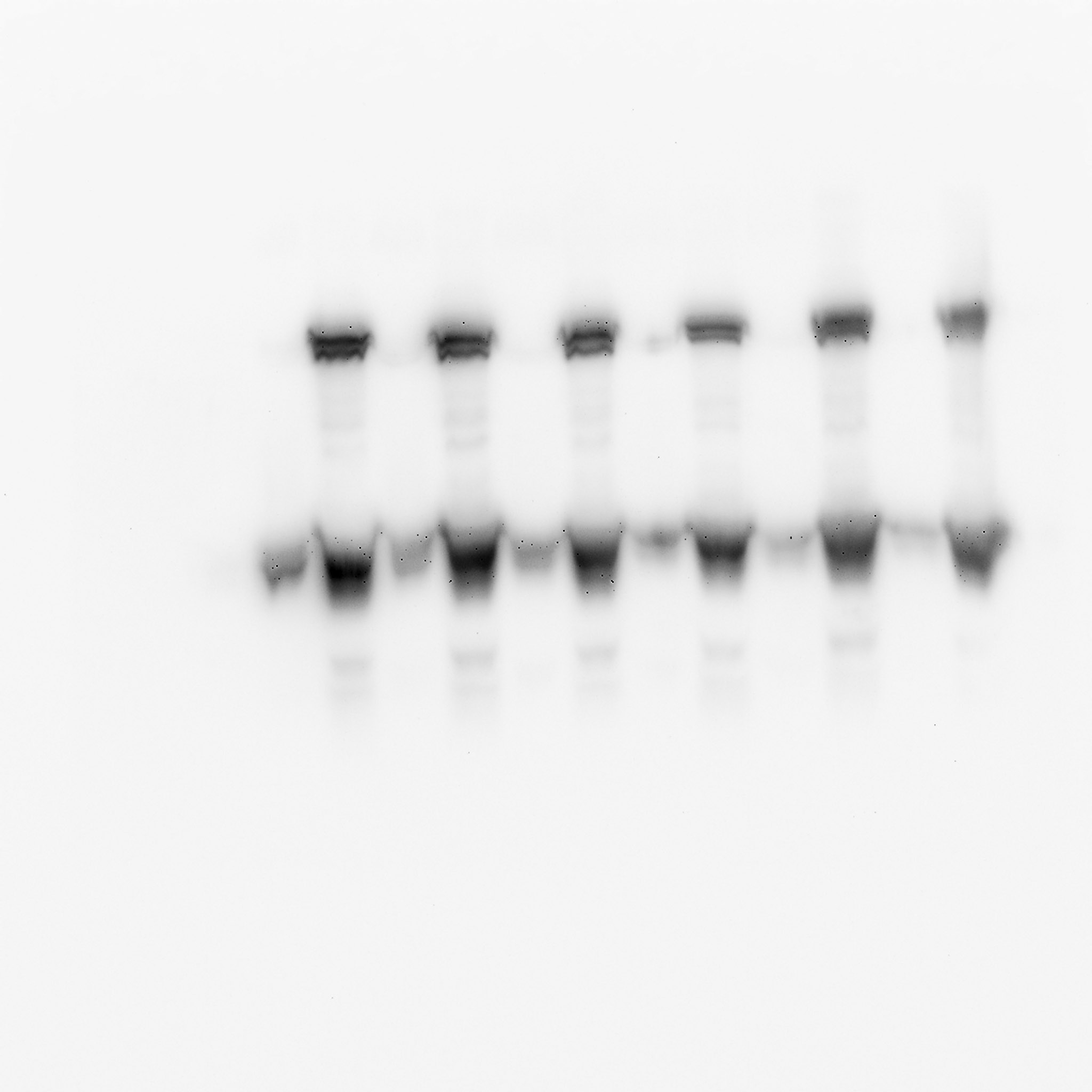

Supplement: Figure 2—source data 1. — A, B, D contain uncropped western blots of data shown in Figure 2A, B and D. C shows three additional representative examples of CMK-inhibited Shh release. E shows six biological replicates of impaired Shh release in the absence of serum that were quantified and displayed in Figure 2E. Prizm files C and E quantify relative Shh release rates based on the data shown in tiff-files B + C and E. [file elife-86920-fig2-data1.zip › Figure_2_Source_Data_1 /A_VP025_Furin_Inhibitor.jpg]

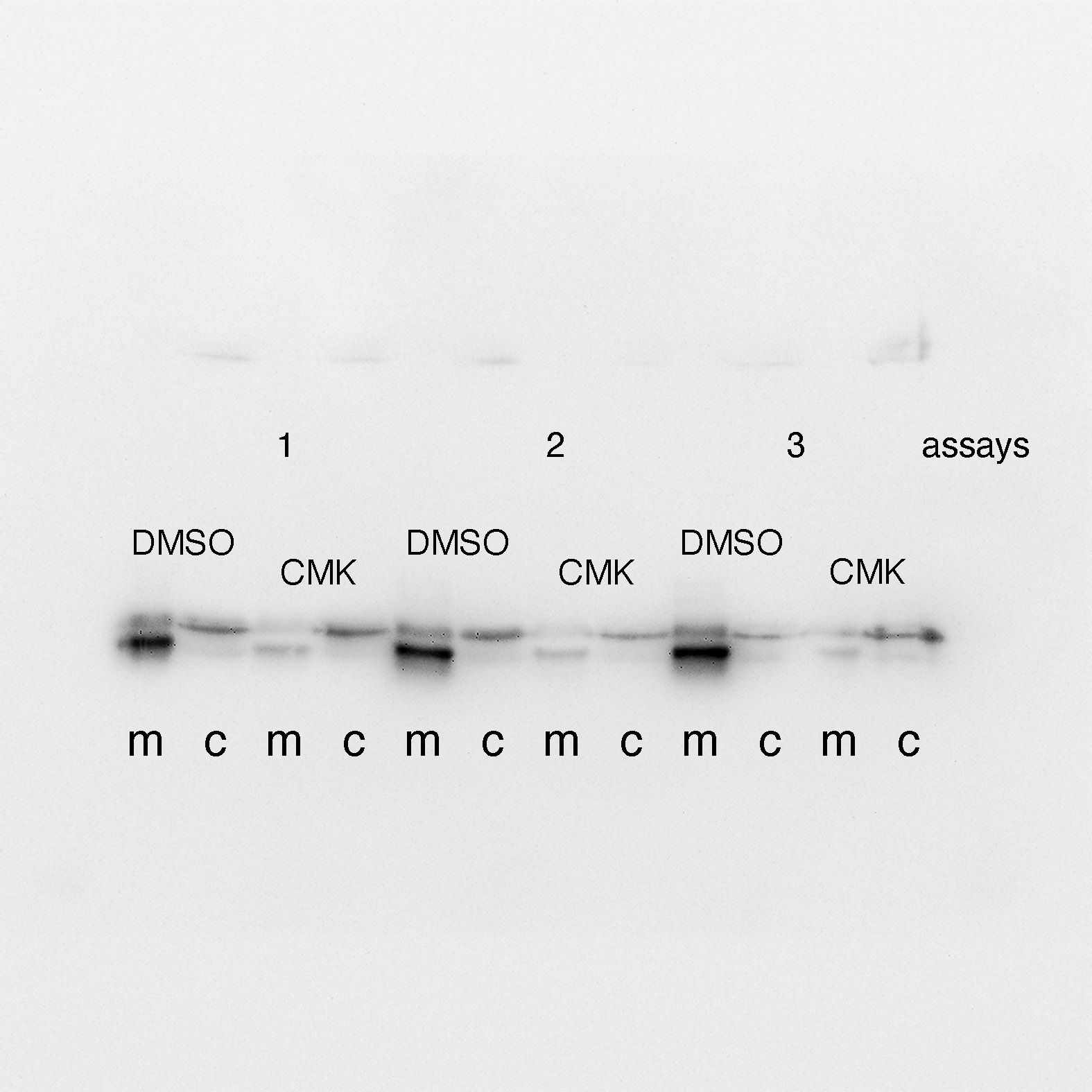

Supplement: Figure 2—source data 1. — A, B, D contain uncropped western blots of data shown in Figure 2A, B and D. C shows three additional representative examples of CMK-inhibited Shh release. E shows six biological replicates of impaired Shh release in the absence of serum that were quantified and displayed in Figure 2E. Prizm files C and E quantify relative Shh release rates based on the data shown in tiff-files B + C and E. [file elife-86920-fig2-data1.zip › Figure_2_Source_Data_1 /B_anti-Shh_CMK labelled.jpg]

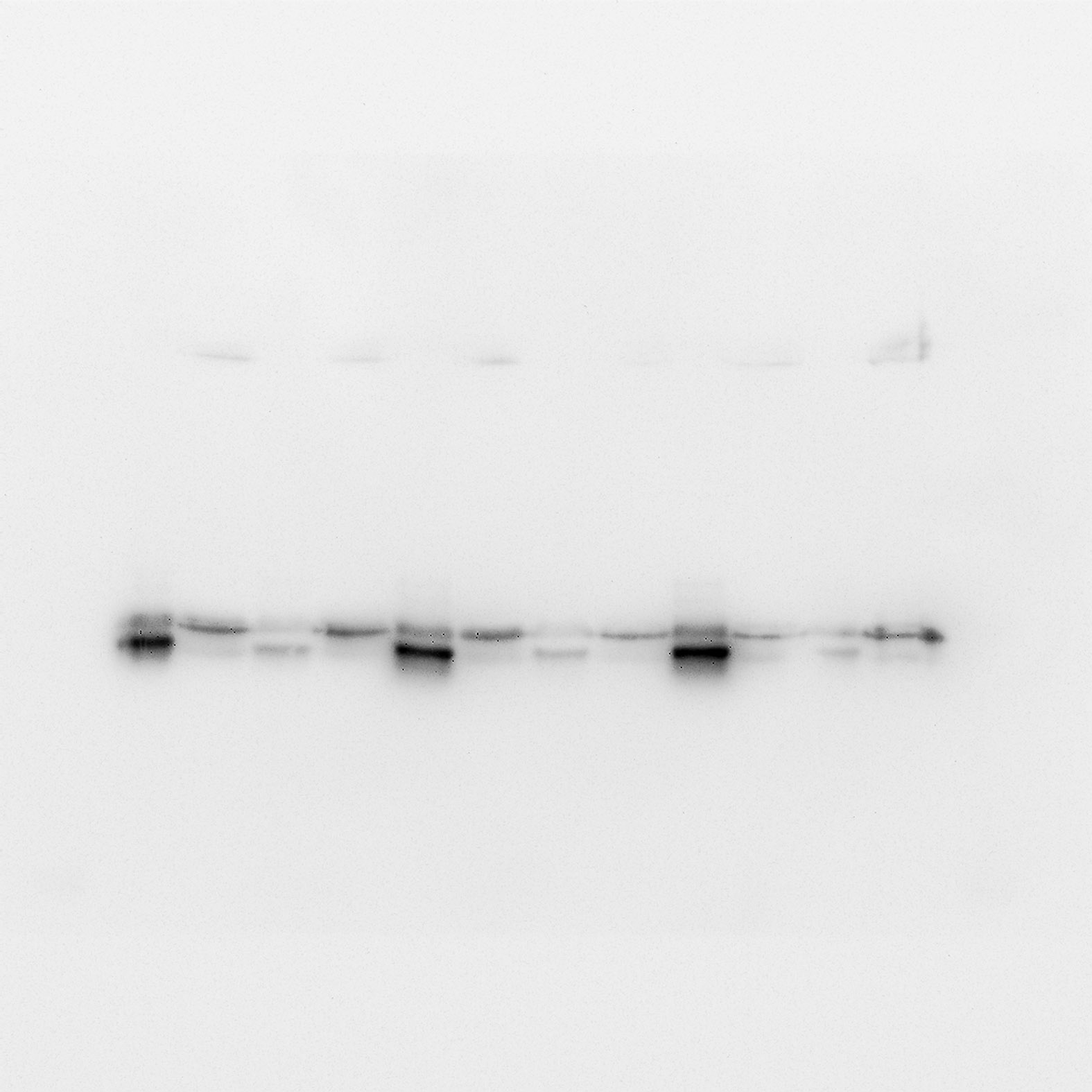

Supplement: Figure 2—source data 1. — A, B, D contain uncropped western blots of data shown in Figure 2A, B and D. C shows three additional representative examples of CMK-inhibited Shh release. E shows six biological replicates of impaired Shh release in the absence of serum that were quantified and displayed in Figure 2E. Prizm files C and E quantify relative Shh release rates based on the data shown in tiff-files B + C and E. [file elife-86920-fig2-data1.zip › Figure_2_Source_Data_1 /B_anti-Shh_CMK.jpg]

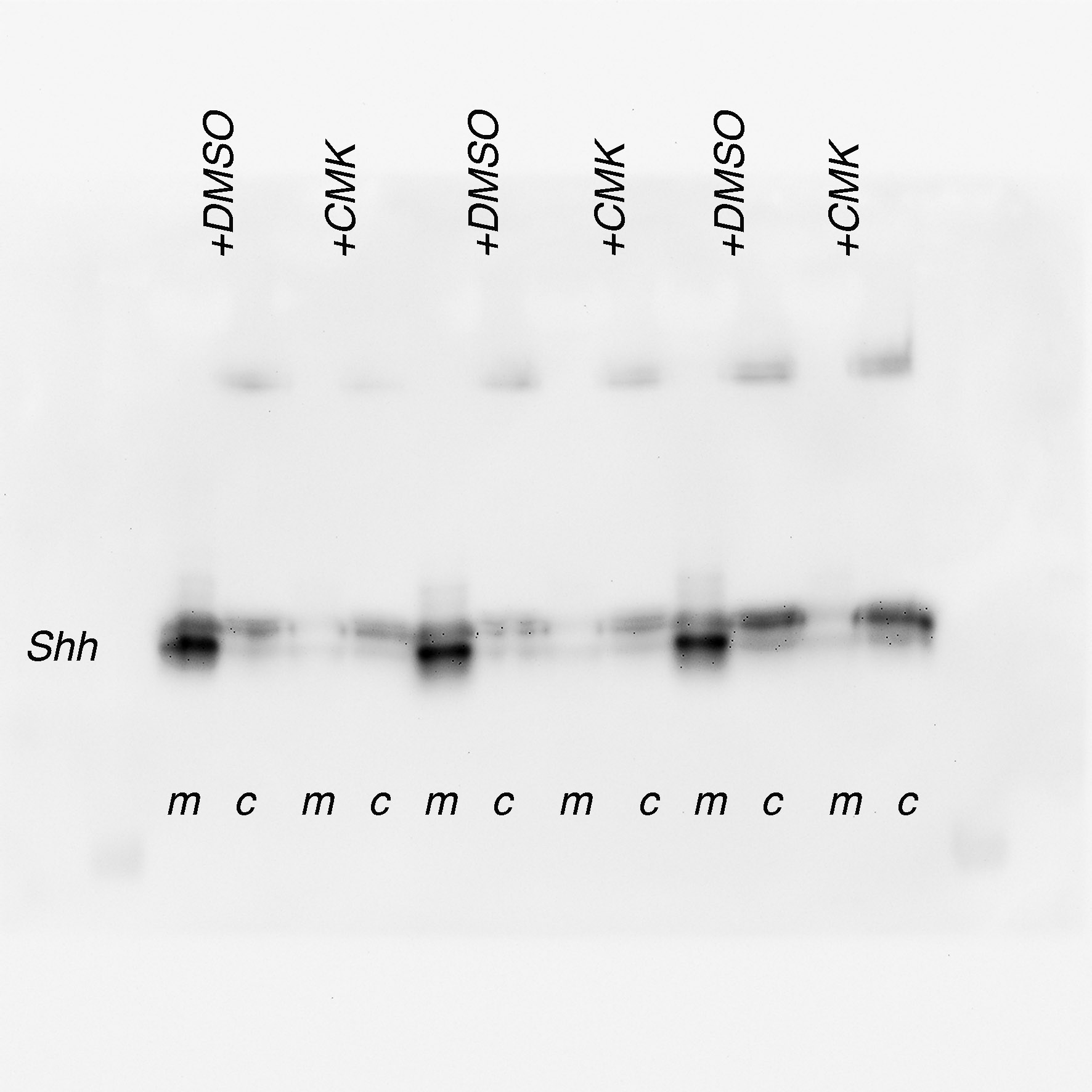

Supplement: Figure 2—source data 1. — A, B, D contain uncropped western blots of data shown in Figure 2A, B and D. C shows three additional representative examples of CMK-inhibited Shh release. E shows six biological replicates of impaired Shh release in the absence of serum that were quantified and displayed in Figure 2E. Prizm files C and E quantify relative Shh release rates based on the data shown in tiff-files B + C and E. [file elife-86920-fig2-data1.zip › Figure_2_Source_Data_1 /C_antiShh_second_blot_for_graph labelled.jpg]

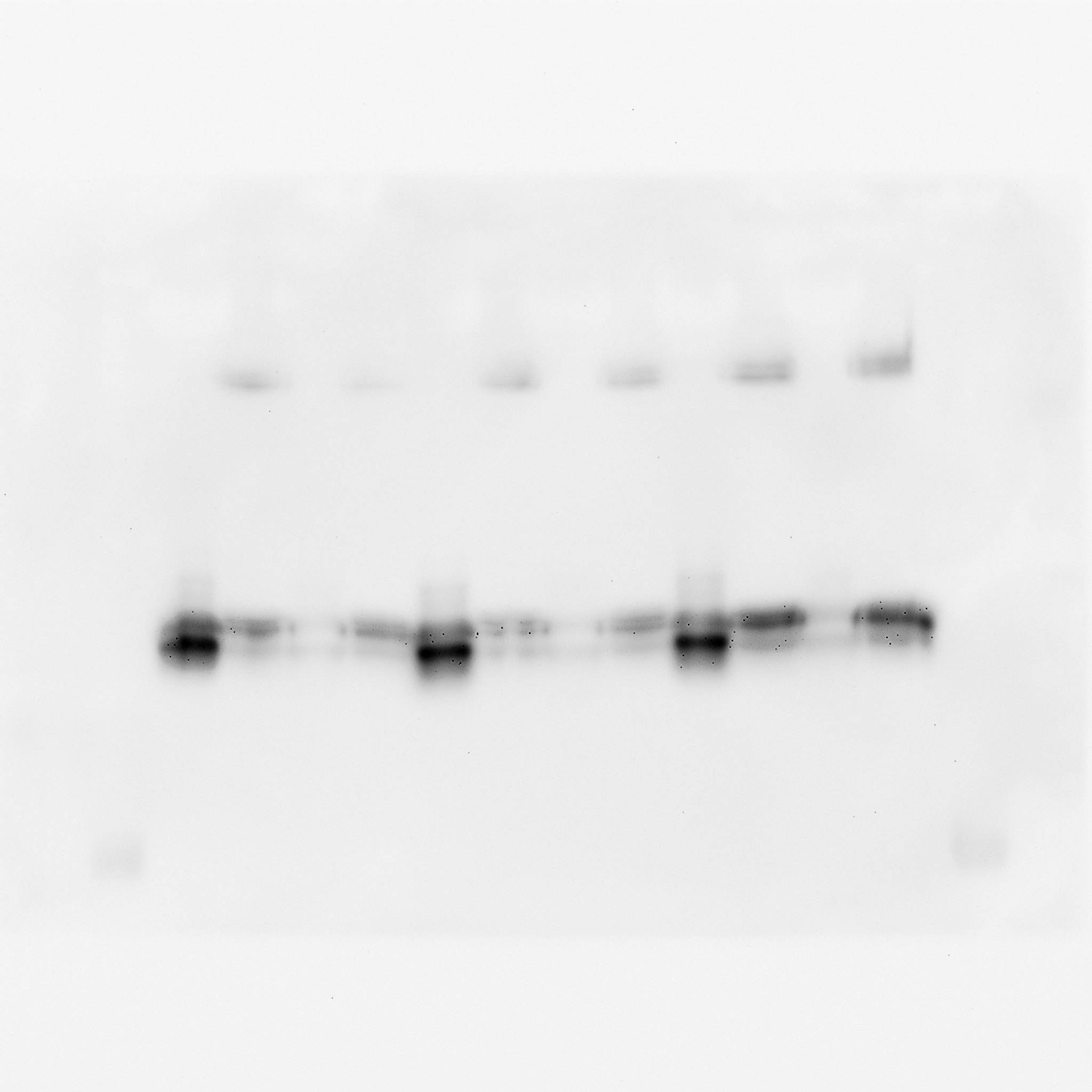

Supplement: Figure 2—source data 1. — A, B, D contain uncropped western blots of data shown in Figure 2A, B and D. C shows three additional representative examples of CMK-inhibited Shh release. E shows six biological replicates of impaired Shh release in the absence of serum that were quantified and displayed in Figure 2E. Prizm files C and E quantify relative Shh release rates based on the data shown in tiff-files B + C and E. [file elife-86920-fig2-data1.zip › Figure_2_Source_Data_1 /C_antiShh_second_blot_for_graph.jpg]

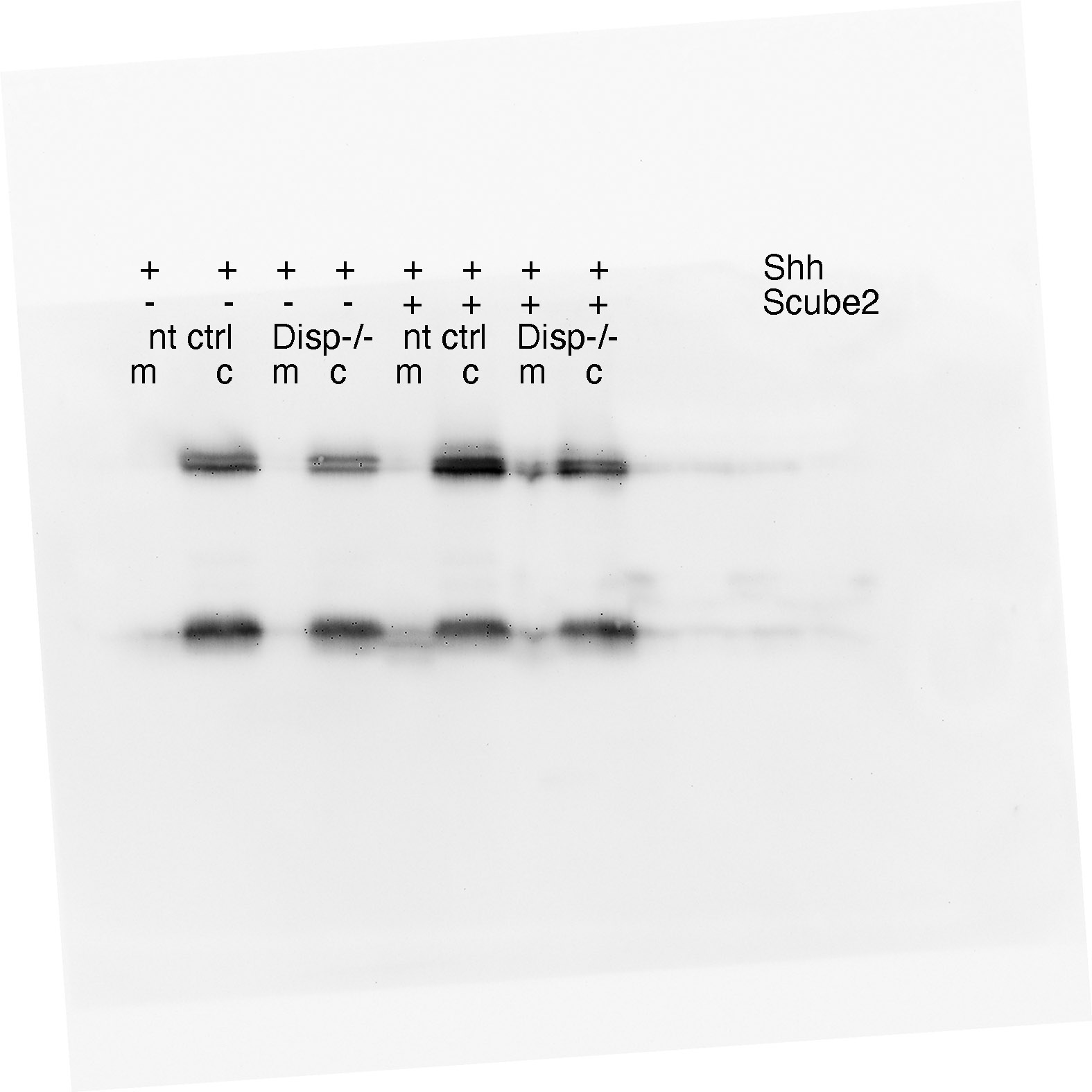

Supplement: Figure 2—source data 1. — A, B, D contain uncropped western blots of data shown in Figure 2A, B and D. C shows three additional representative examples of CMK-inhibited Shh release. E shows six biological replicates of impaired Shh release in the absence of serum that were quantified and displayed in Figure 2E. Prizm files C and E quantify relative Shh release rates based on the data shown in tiff-files B + C and E. [file elife-86920-fig2-data1.zip › Figure_2_Source_Data_1 /D_V752_1_Shh_1min labelled.jpg]

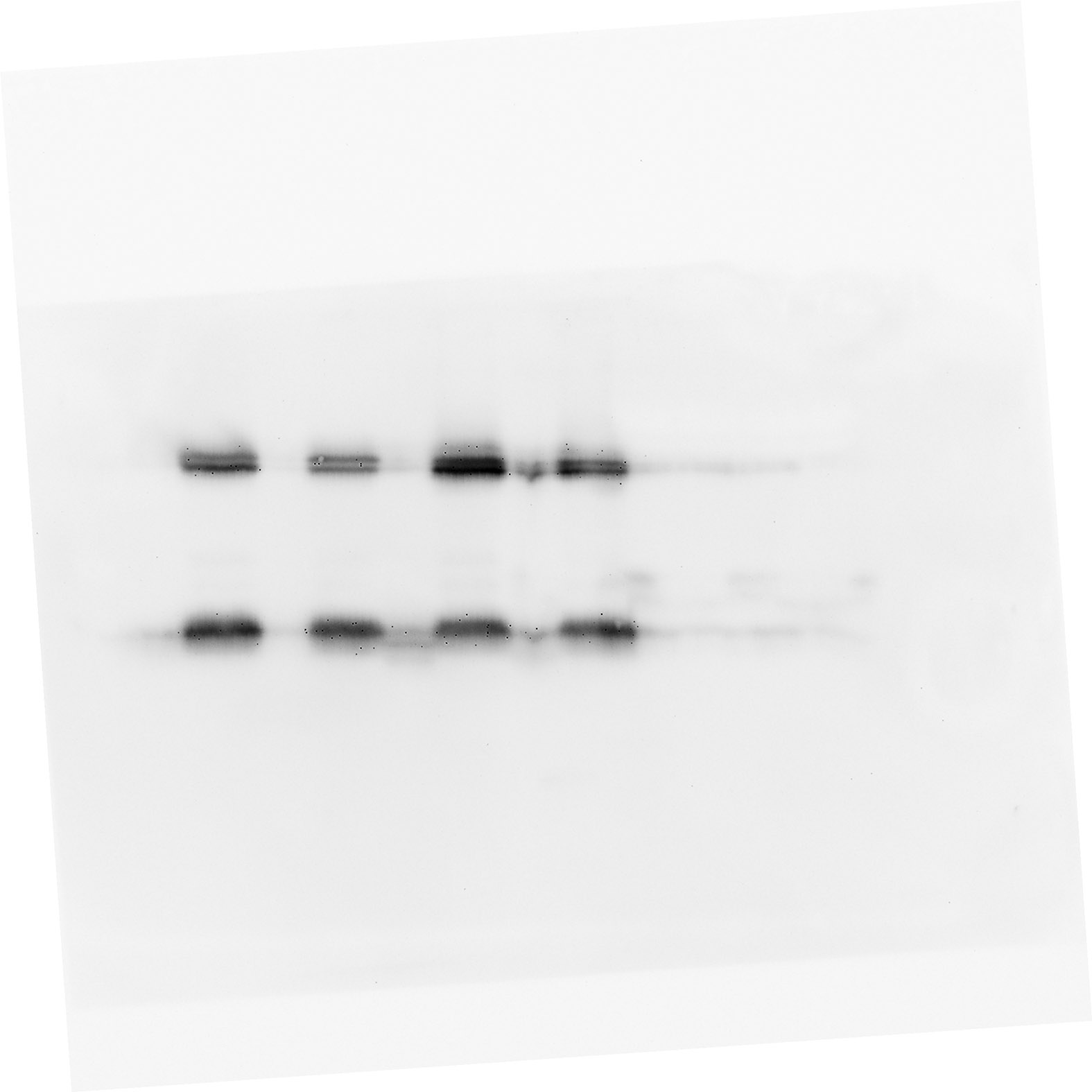

Supplement: Figure 2—source data 1. — A, B, D contain uncropped western blots of data shown in Figure 2A, B and D. C shows three additional representative examples of CMK-inhibited Shh release. E shows six biological replicates of impaired Shh release in the absence of serum that were quantified and displayed in Figure 2E. Prizm files C and E quantify relative Shh release rates based on the data shown in tiff-files B + C and E. [file elife-86920-fig2-data1.zip › Figure_2_Source_Data_1 /D_V752_1_Shh_1min.jpg]

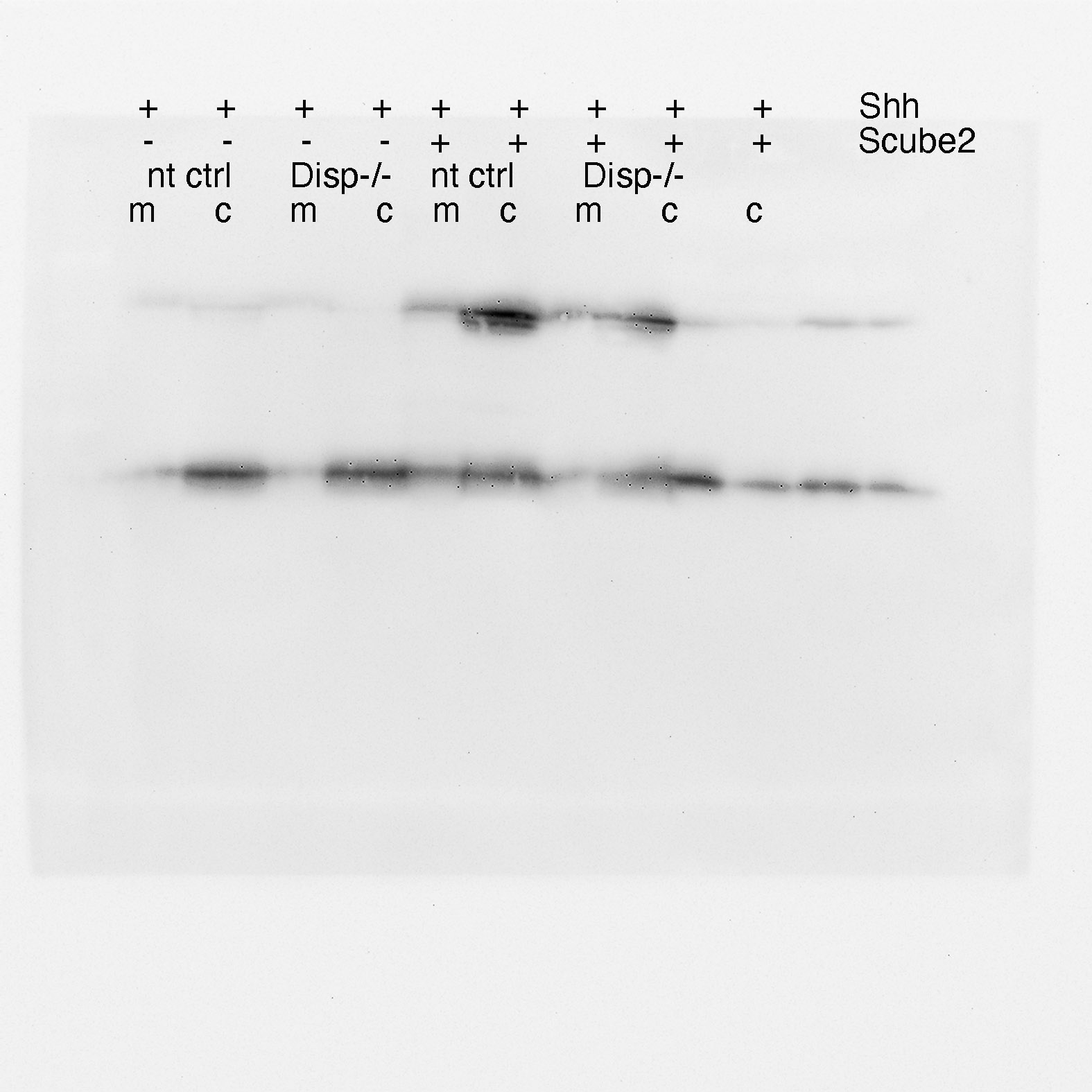

Supplement: Figure 2—source data 1. — A, B, D contain uncropped western blots of data shown in Figure 2A, B and D. C shows three additional representative examples of CMK-inhibited Shh release. E shows six biological replicates of impaired Shh release in the absence of serum that were quantified and displayed in Figure 2E. Prizm files C and E quantify relative Shh release rates based on the data shown in tiff-files B + C and E. [file elife-86920-fig2-data1.zip › Figure_2_Source_Data_1 /E_raw-blot_2_V752_4_Shh_2min labelled.jpg]

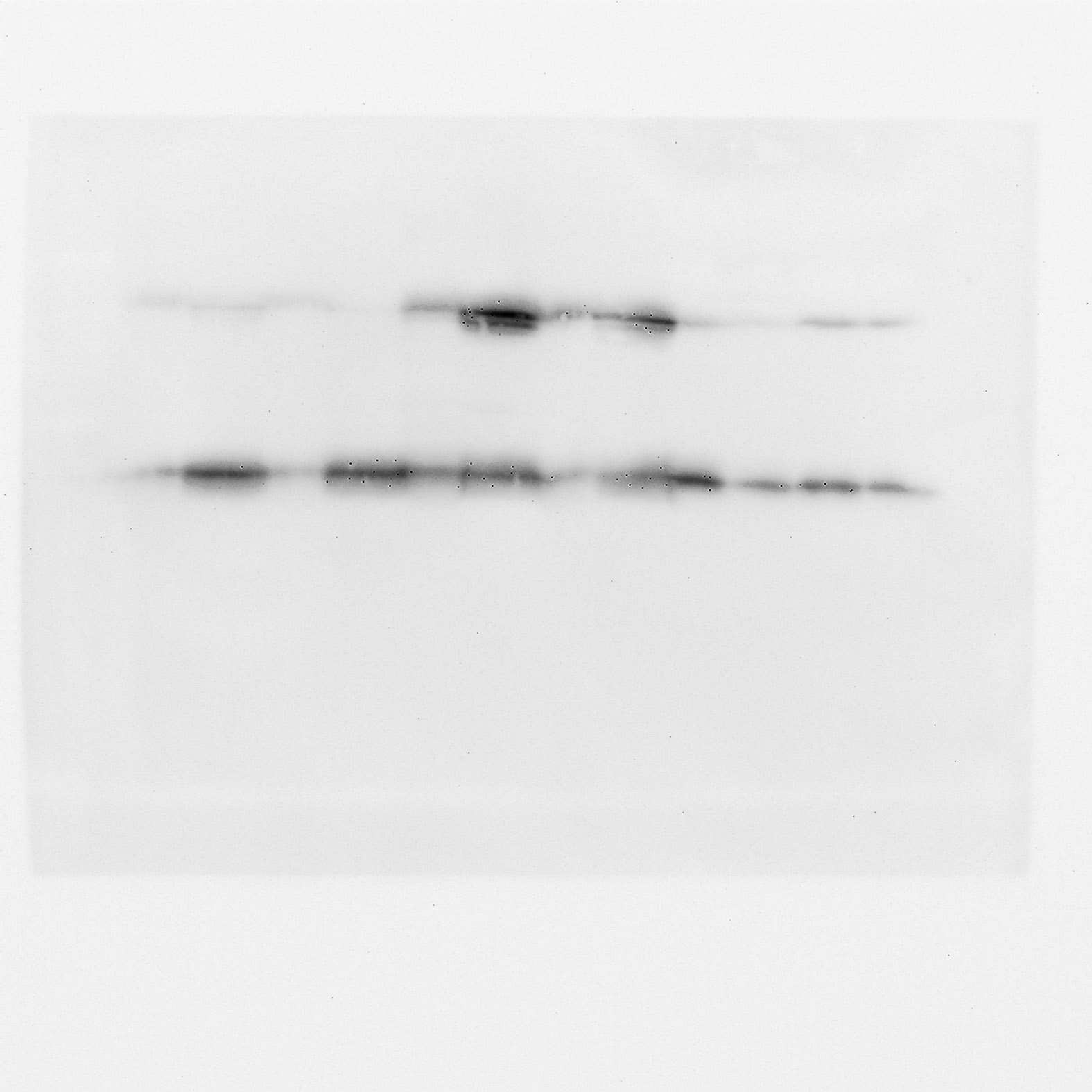

Supplement: Figure 2—source data 1. — A, B, D contain uncropped western blots of data shown in Figure 2A, B and D. C shows three additional representative examples of CMK-inhibited Shh release. E shows six biological replicates of impaired Shh release in the absence of serum that were quantified and displayed in Figure 2E. Prizm files C and E quantify relative Shh release rates based on the data shown in tiff-files B + C and E. [file elife-86920-fig2-data1.zip › Figure_2_Source_Data_1 /E_raw-blot_2_V752_4_Shh_2min.jpg]

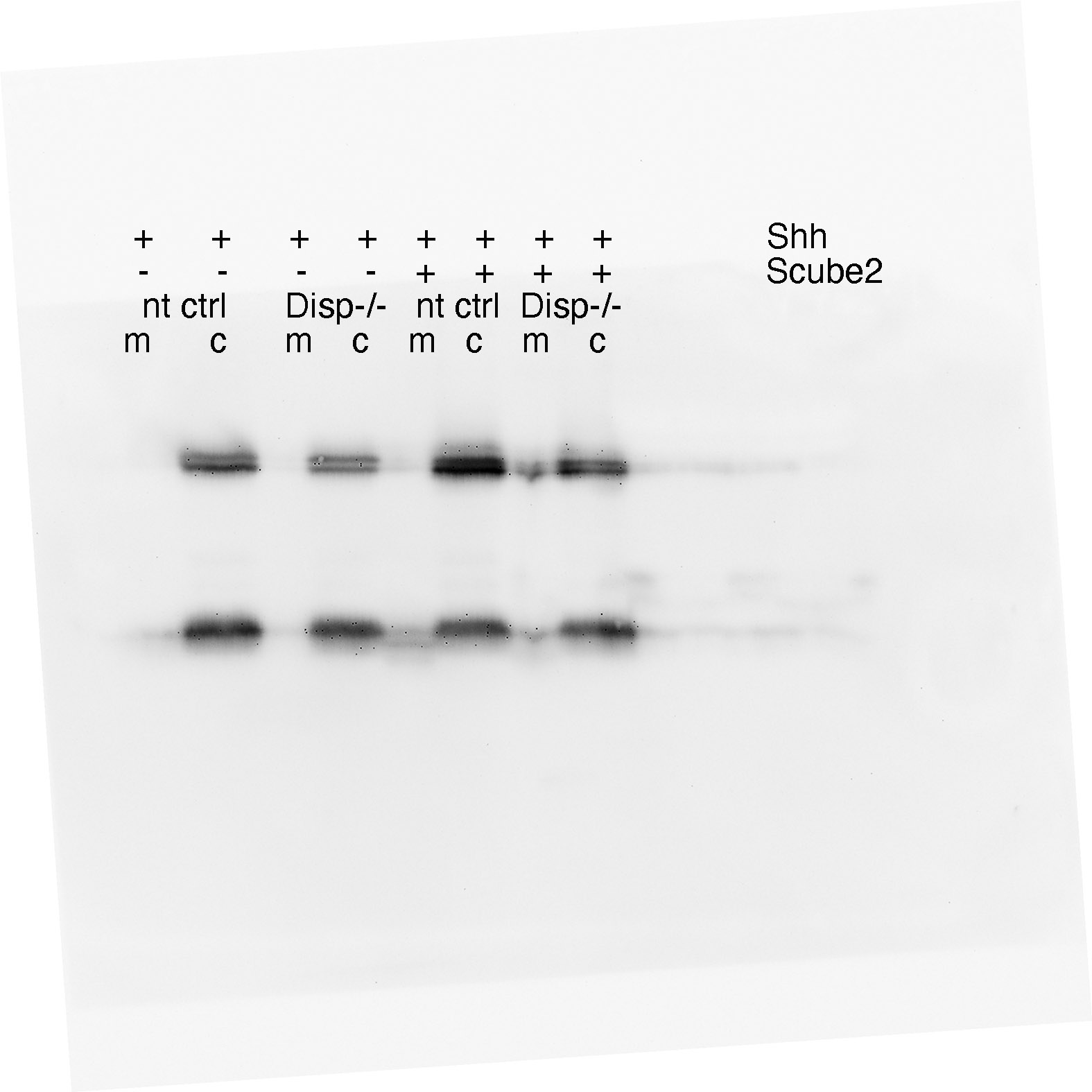

Supplement: Figure 2—source data 1. — A, B, D contain uncropped western blots of data shown in Figure 2A, B and D. C shows three additional representative examples of CMK-inhibited Shh release. E shows six biological replicates of impaired Shh release in the absence of serum that were quantified and displayed in Figure 2E. Prizm files C and E quantify relative Shh release rates based on the data shown in tiff-files B + C and E. [file elife-86920-fig2-data1.zip › Figure_2_Source_Data_1 /E_raw_blot_1_V752_1_Shh_1min labelled.jpg]
