# Supplementary figures and images for: Two-way Dispatched function in Sonic hedgehog shedding and transfer to high-density lipoproteins (part 2 of 3)
Source: eLife. 2024 Sep 19;12:RP86920. doi: 10.7554/eLife.86920 (PMC11412720; doi:10.7554/eLife.86920)

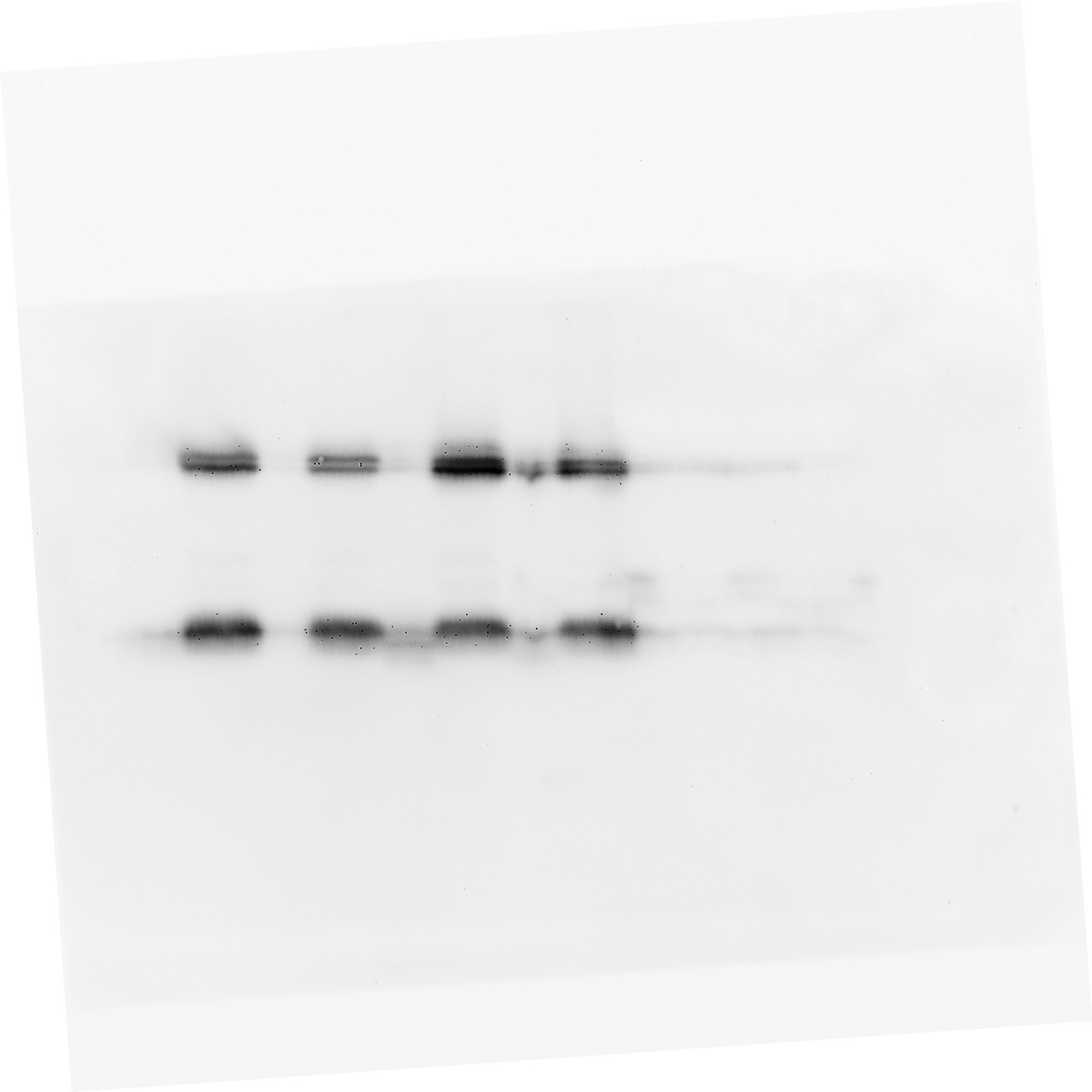

Supplement: Figure 2—source data 1. — A, B, D contain uncropped western blots of data shown in Figure 2A, B and D. C shows three additional representative examples of CMK-inhibited Shh release. E shows six biological replicates of impaired Shh release in the absence of serum that were quantified and displayed in Figure 2E. Prizm files C and E quantify relative Shh release rates based on the data shown in tiff-files B + C and E. [file elife-86920-fig2-data1.zip › Figure_2_Source_Data_1 /E_raw_blot_1_V752_1_Shh_1min.jpg]

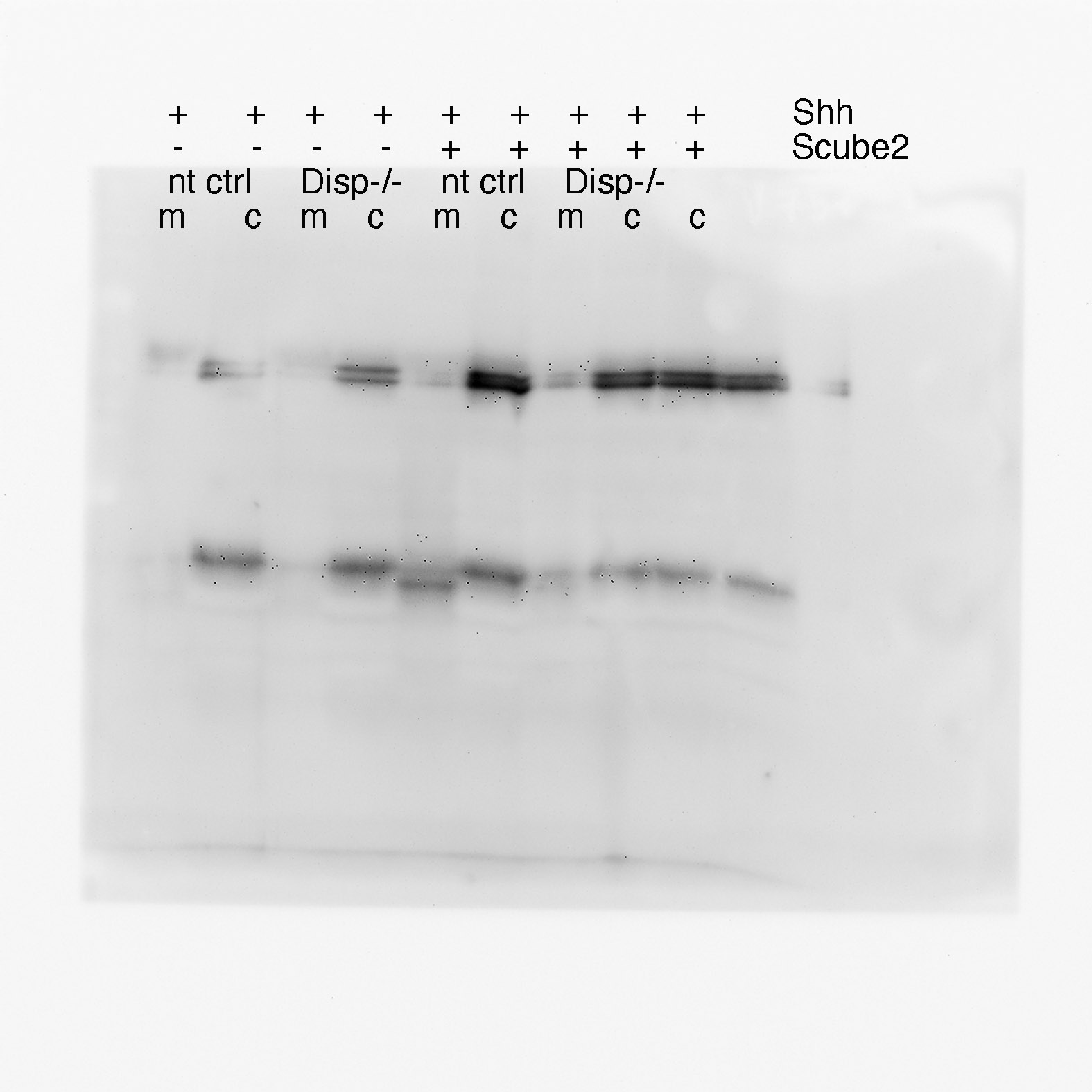

Supplement: Figure 2—source data 1. — A, B, D contain uncropped western blots of data shown in Figure 2A, B and D. C shows three additional representative examples of CMK-inhibited Shh release. E shows six biological replicates of impaired Shh release in the absence of serum that were quantified and displayed in Figure 2E. Prizm files C and E quantify relative Shh release rates based on the data shown in tiff-files B + C and E. [file elife-86920-fig2-data1.zip › Figure_2_Source_Data_1 /E_raw_blot_3_V757_1_1min_shh labelled.jpg]

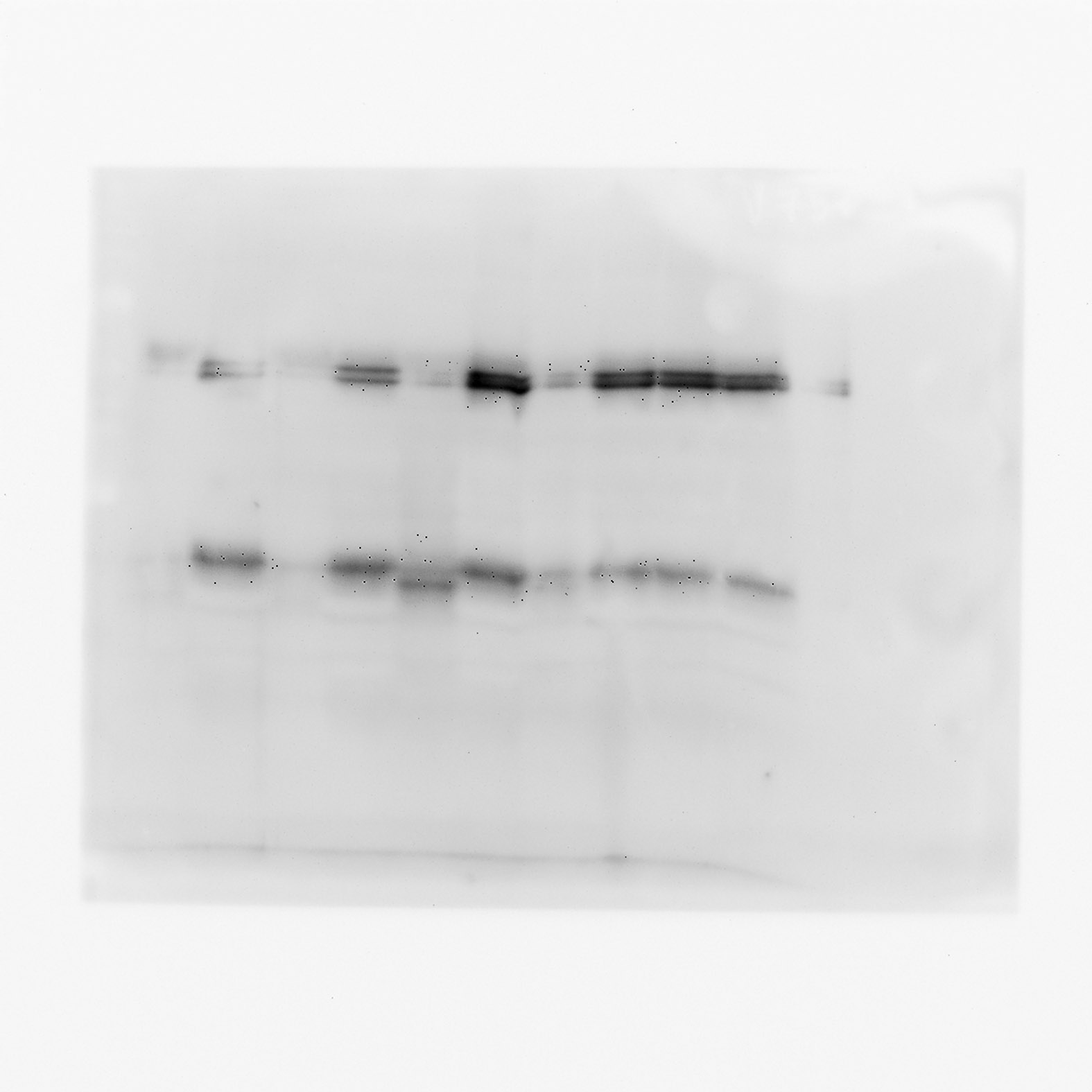

Supplement: Figure 2—source data 1. — A, B, D contain uncropped western blots of data shown in Figure 2A, B and D. C shows three additional representative examples of CMK-inhibited Shh release. E shows six biological replicates of impaired Shh release in the absence of serum that were quantified and displayed in Figure 2E. Prizm files C and E quantify relative Shh release rates based on the data shown in tiff-files B + C and E. [file elife-86920-fig2-data1.zip › Figure_2_Source_Data_1 /E_raw_blot_3_V757_1_1min_shh.jpg]

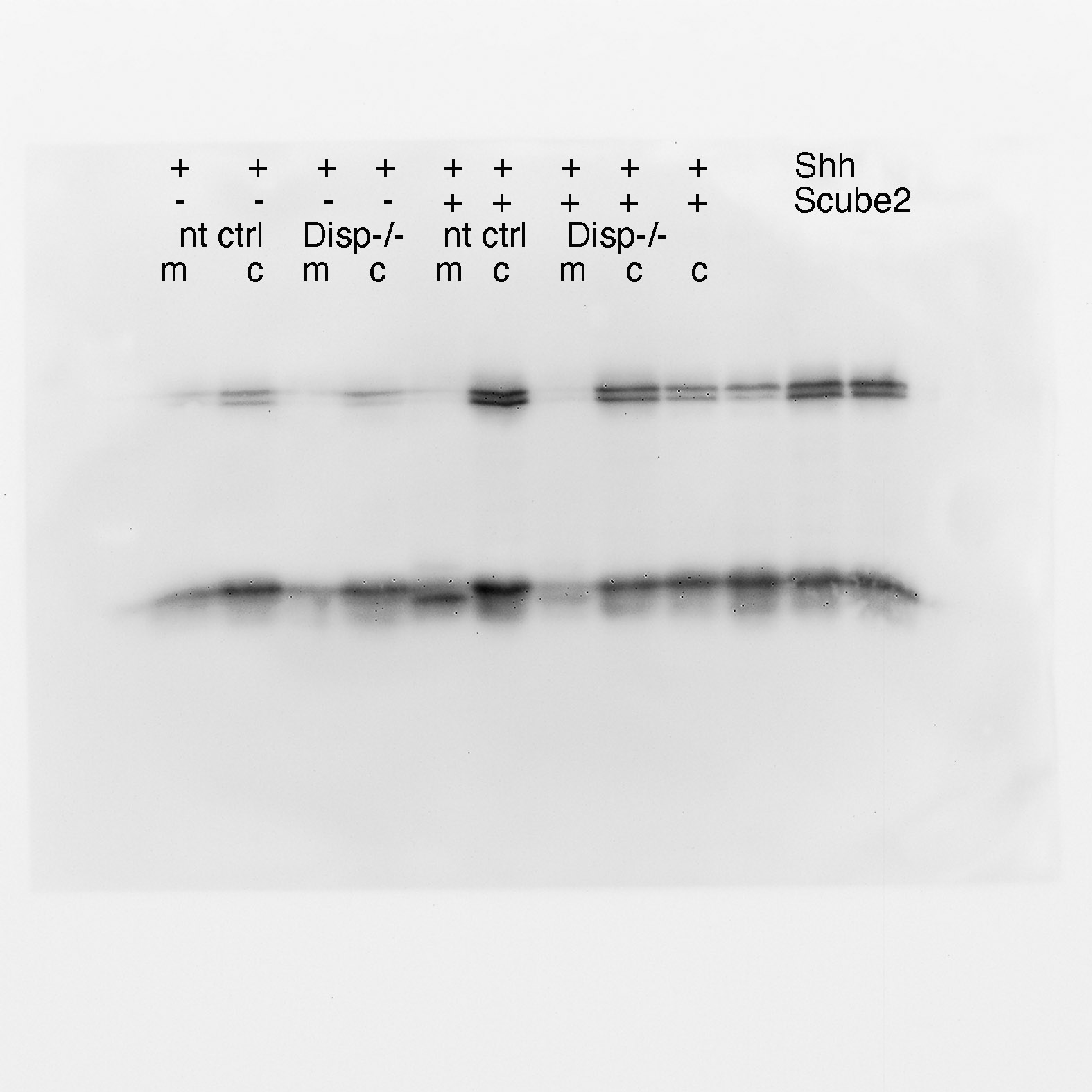

Supplement: Figure 2—source data 1. — A, B, D contain uncropped western blots of data shown in Figure 2A, B and D. C shows three additional representative examples of CMK-inhibited Shh release. E shows six biological replicates of impaired Shh release in the absence of serum that were quantified and displayed in Figure 2E. Prizm files C and E quantify relative Shh release rates based on the data shown in tiff-files B + C and E. [file elife-86920-fig2-data1.zip › Figure_2_Source_Data_1 /E_raw_blot_4_V787_Shh_1_20sec labelled.jpg]

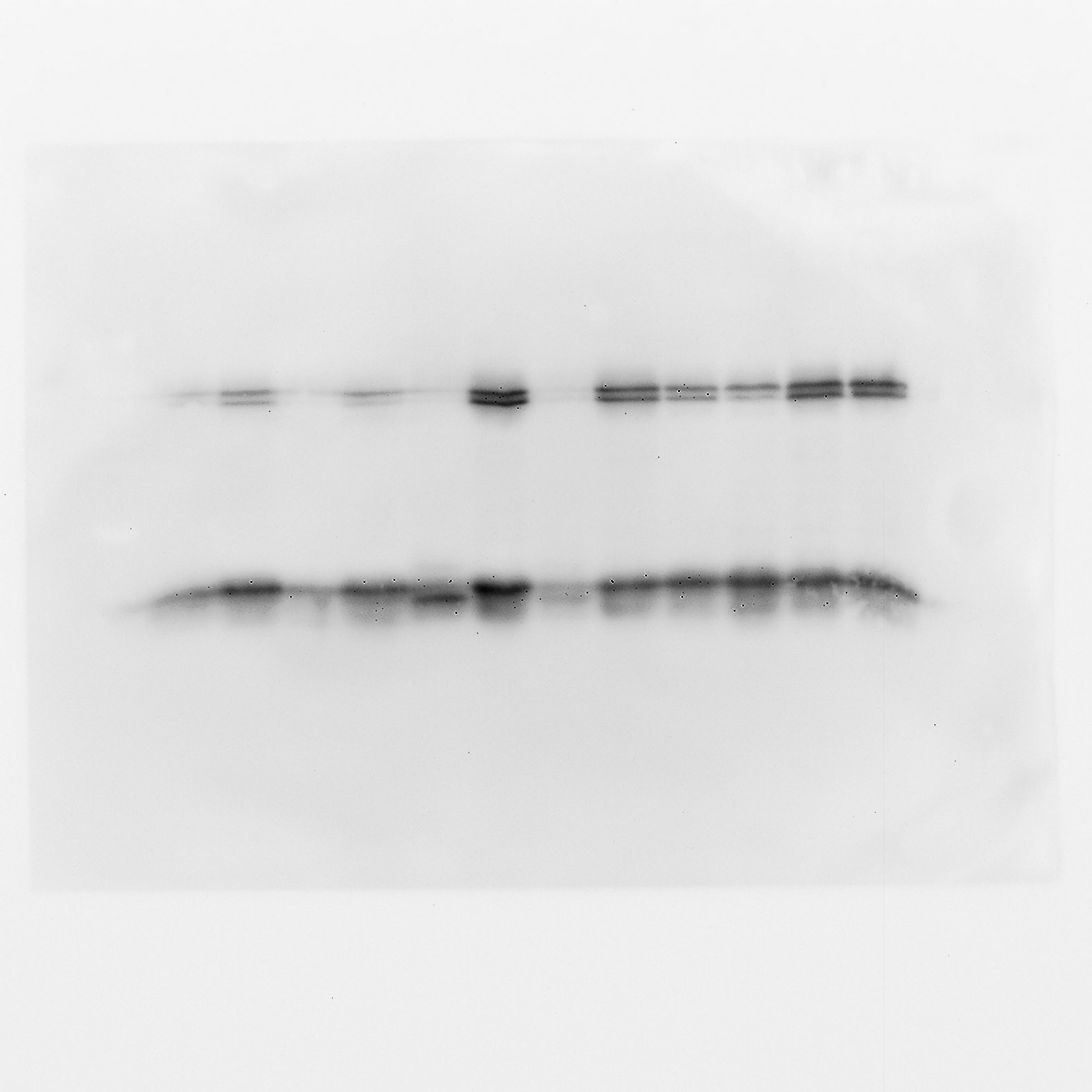

Supplement: Figure 2—source data 1. — A, B, D contain uncropped western blots of data shown in Figure 2A, B and D. C shows three additional representative examples of CMK-inhibited Shh release. E shows six biological replicates of impaired Shh release in the absence of serum that were quantified and displayed in Figure 2E. Prizm files C and E quantify relative Shh release rates based on the data shown in tiff-files B + C and E. [file elife-86920-fig2-data1.zip › Figure_2_Source_Data_1 /E_raw_blot_4_V787_Shh_1_20sec.jpg]

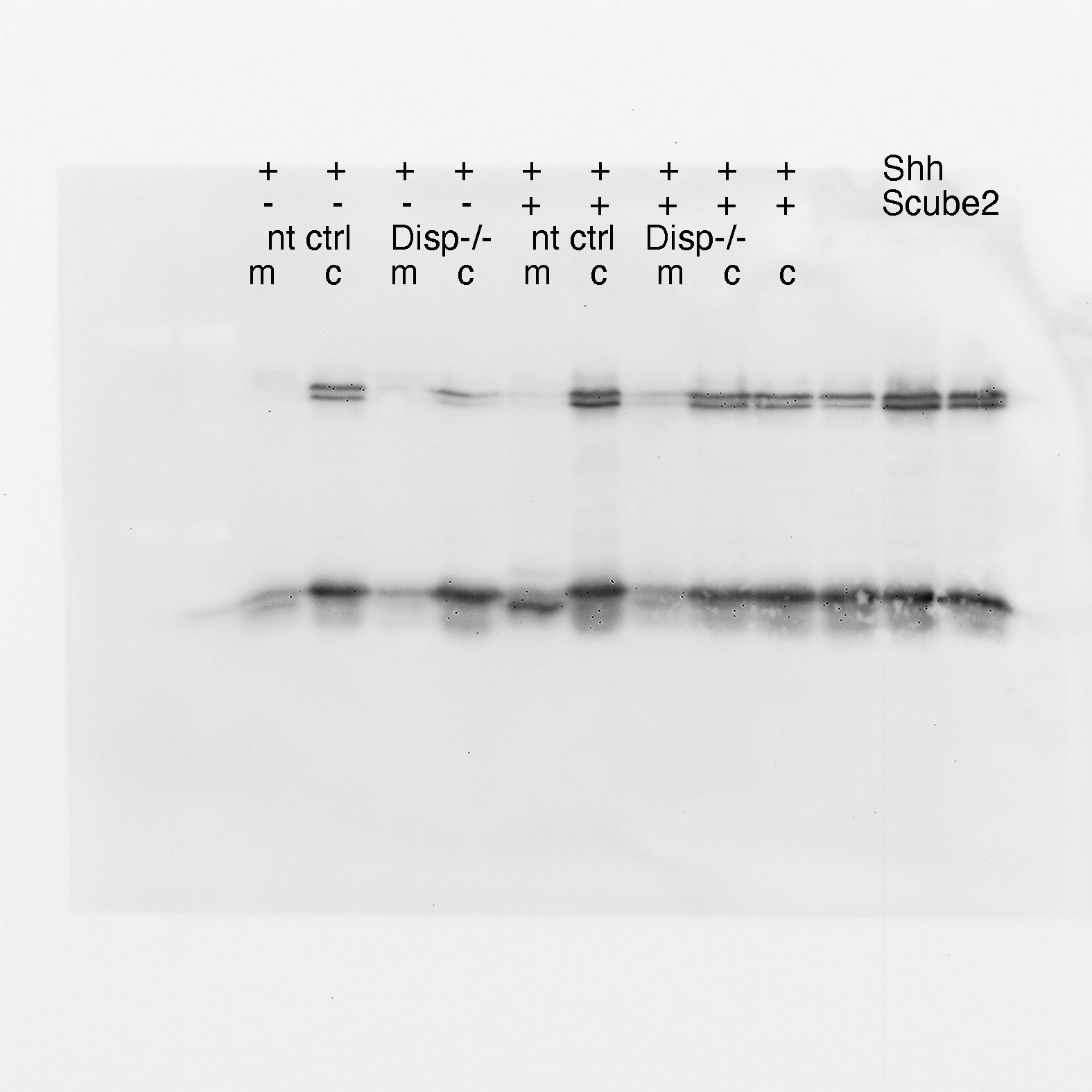

Supplement: Figure 2—source data 1. — A, B, D contain uncropped western blots of data shown in Figure 2A, B and D. C shows three additional representative examples of CMK-inhibited Shh release. E shows six biological replicates of impaired Shh release in the absence of serum that were quantified and displayed in Figure 2E. Prizm files C and E quantify relative Shh release rates based on the data shown in tiff-files B + C and E. [file elife-86920-fig2-data1.zip › Figure_2_Source_Data_1 /E_raw_blot_5_V787_Shh_2_7sec labelled.jpg]

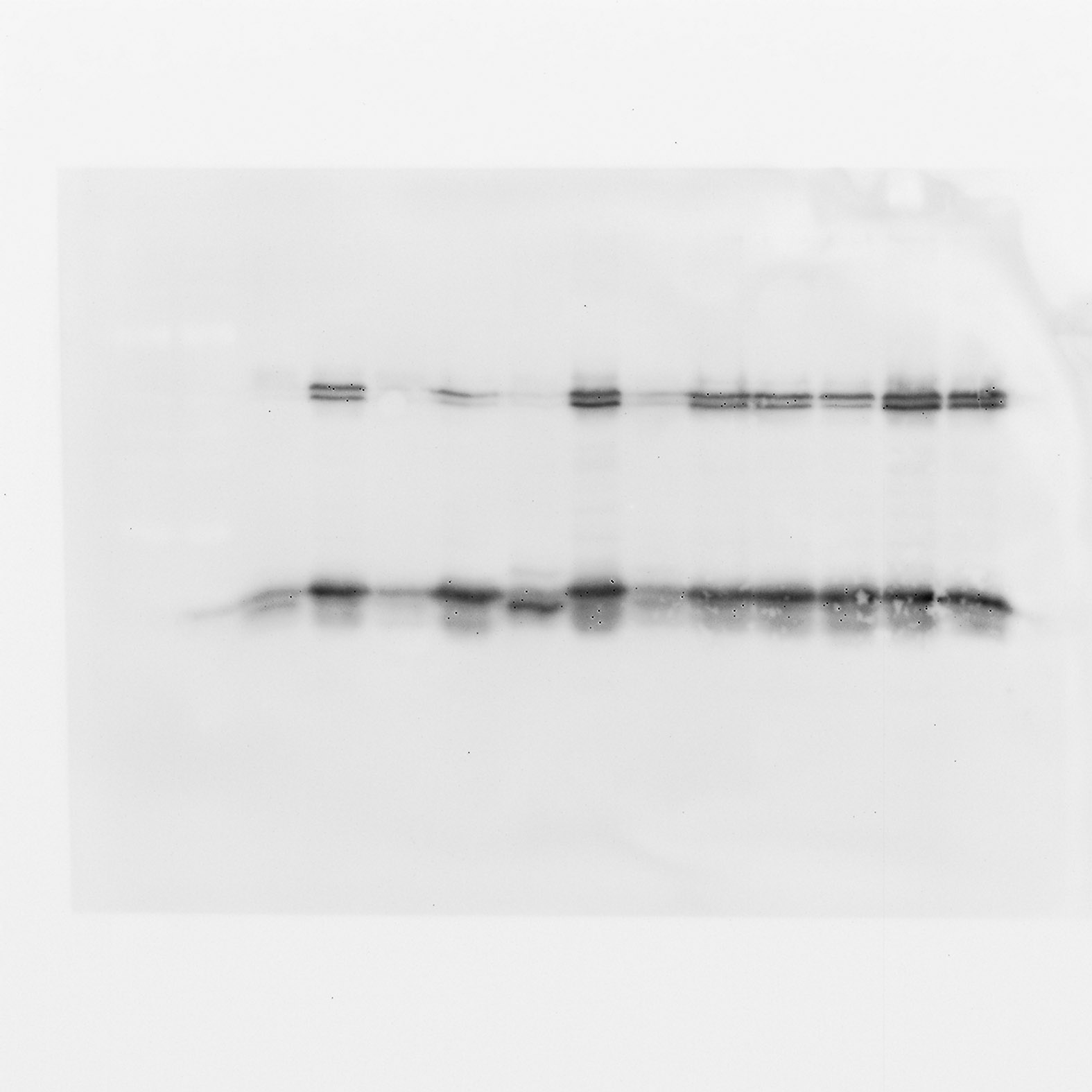

Supplement: Figure 2—source data 1. — A, B, D contain uncropped western blots of data shown in Figure 2A, B and D. C shows three additional representative examples of CMK-inhibited Shh release. E shows six biological replicates of impaired Shh release in the absence of serum that were quantified and displayed in Figure 2E. Prizm files C and E quantify relative Shh release rates based on the data shown in tiff-files B + C and E. [file elife-86920-fig2-data1.zip › Figure_2_Source_Data_1 /E_raw_blot_5_V787_Shh_2_7sec.jpg]

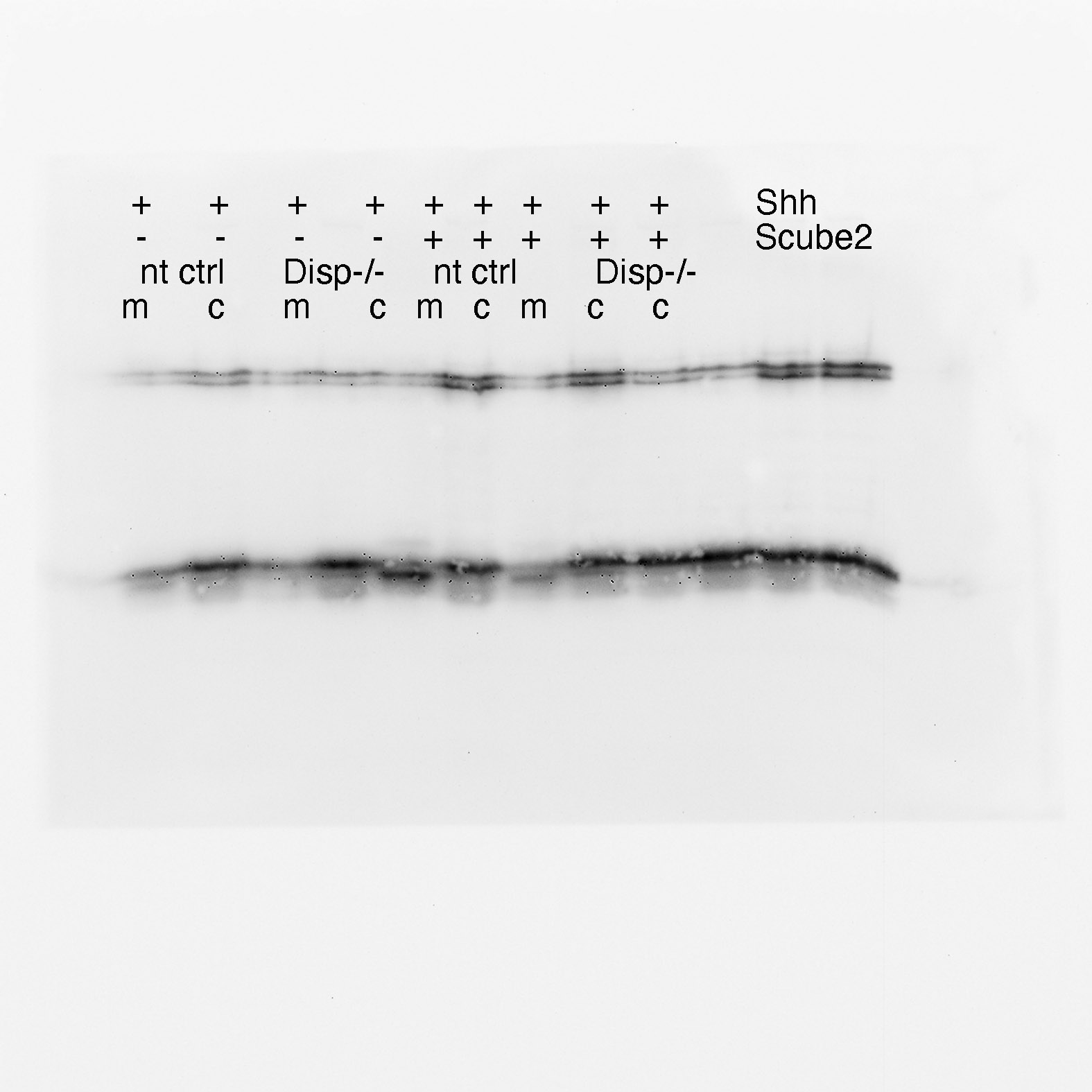

Supplement: Figure 2—source data 1. — A, B, D contain uncropped western blots of data shown in Figure 2A, B and D. C shows three additional representative examples of CMK-inhibited Shh release. E shows six biological replicates of impaired Shh release in the absence of serum that were quantified and displayed in Figure 2E. Prizm files C and E quantify relative Shh release rates based on the data shown in tiff-files B + C and E. [file elife-86920-fig2-data1.zip › Figure_2_Source_Data_1 /E_raw_blot_6_V787_Shh_3_7sec labelled.jpg]

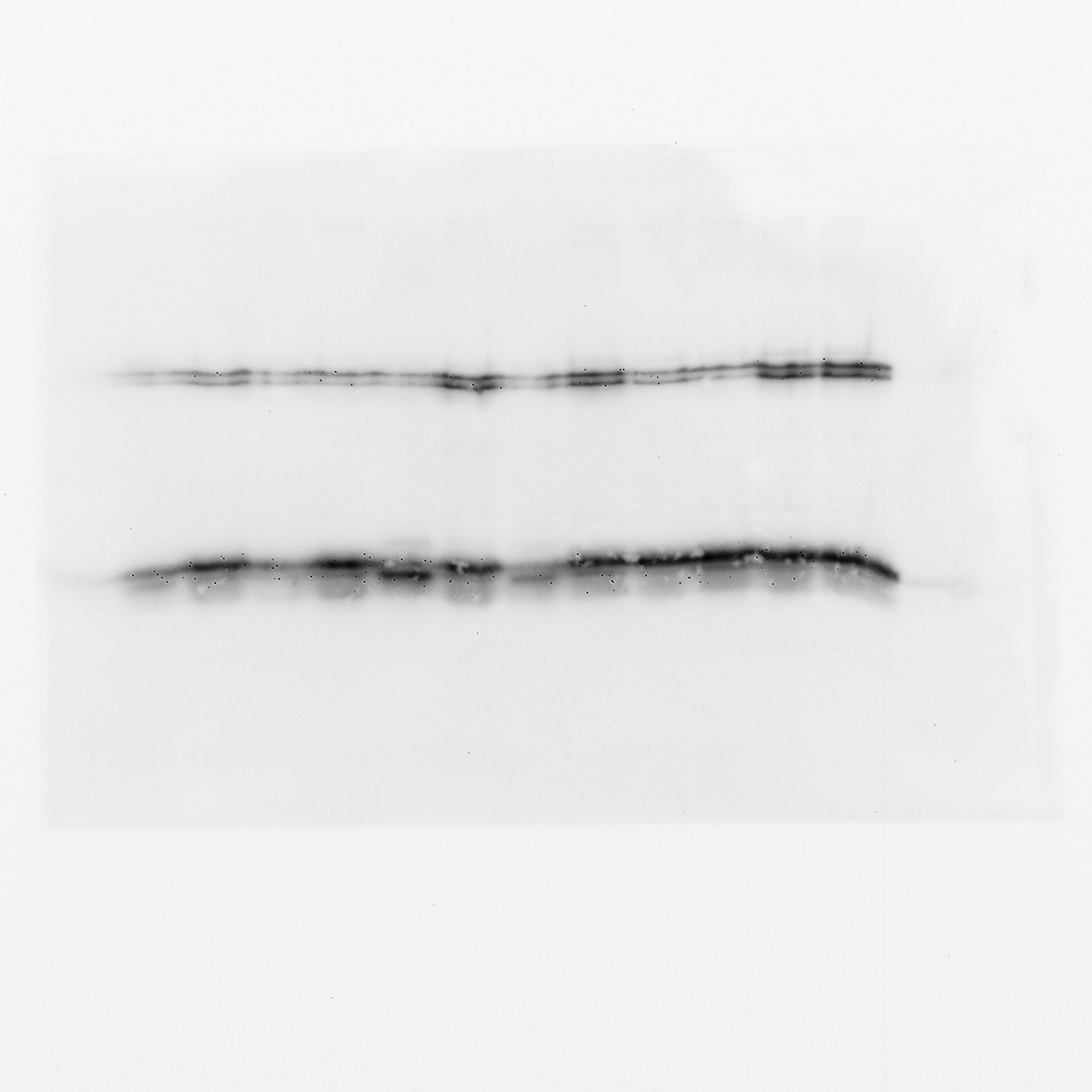

Supplement: Figure 2—source data 1. — A, B, D contain uncropped western blots of data shown in Figure 2A, B and D. C shows three additional representative examples of CMK-inhibited Shh release. E shows six biological replicates of impaired Shh release in the absence of serum that were quantified and displayed in Figure 2E. Prizm files C and E quantify relative Shh release rates based on the data shown in tiff-files B + C and E. [file elife-86920-fig2-data1.zip › Figure_2_Source_Data_1 /E_raw_blot_6_V787_Shh_3_7sec.jpg]

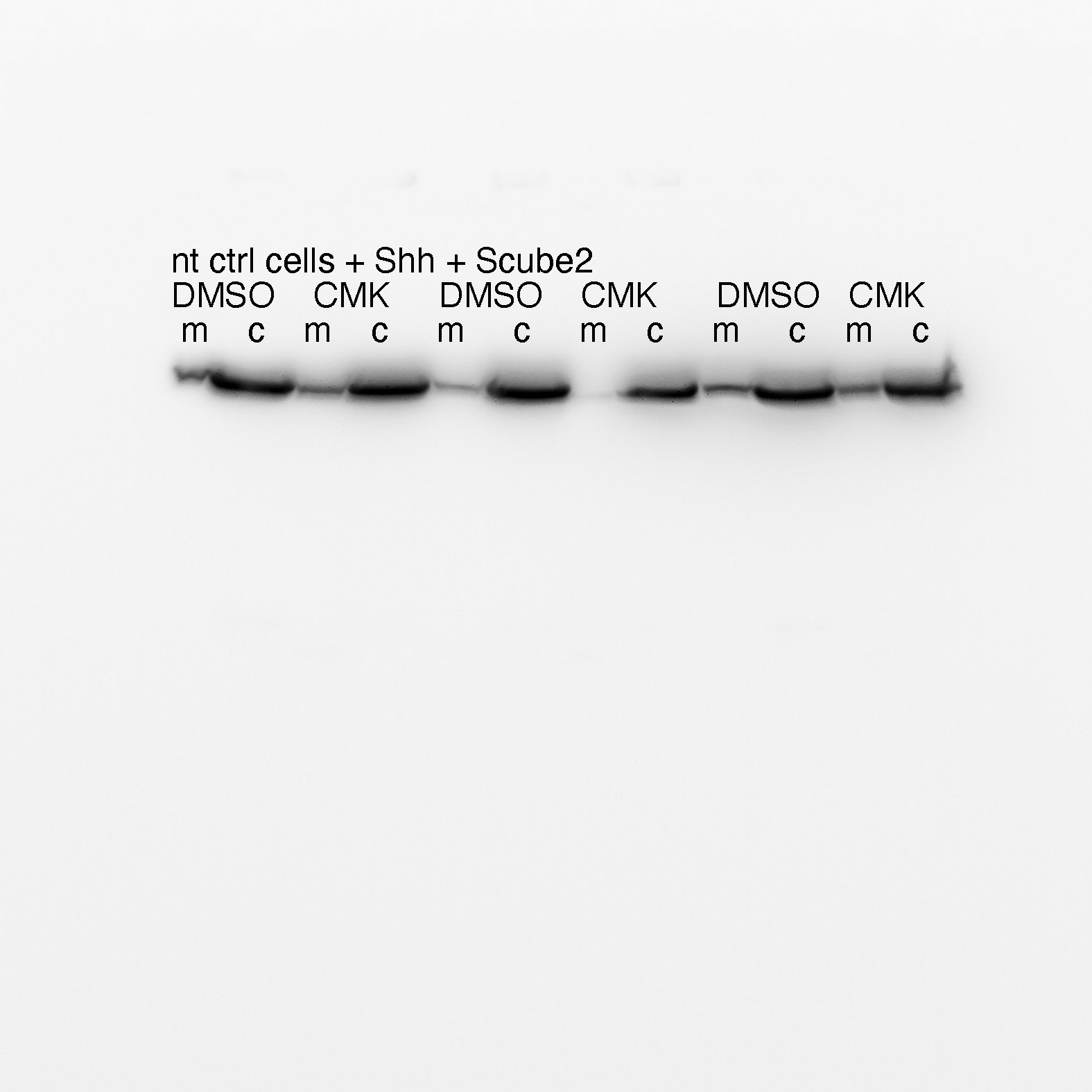

Supplement: Figure 2—figure supplement 1—source data 1. [file elife-86920-fig2-figsupp1-data1.zip › Figure 2-Figure Supplement 1 - Source Data 1/A_20200316_VK122_Gel1_anti Aktin_16bit_12sec_ohne Artefakte labelled.jpg]

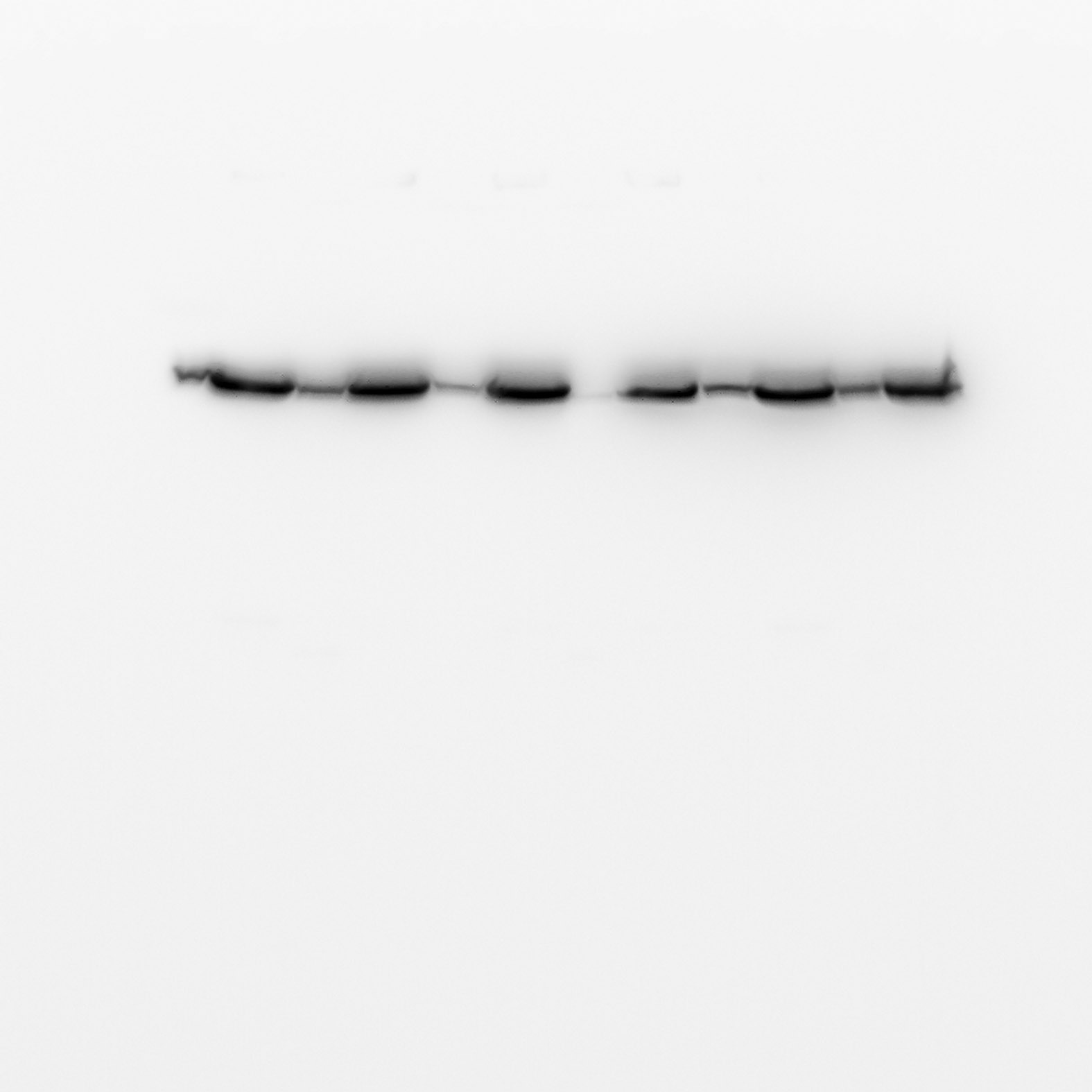

Supplement: Figure 2—figure supplement 1—source data 1. [file elife-86920-fig2-figsupp1-data1.zip › Figure 2-Figure Supplement 1 - Source Data 1/A_20200316_VK122_Gel1_anti Aktin_16bit_12sec_ohne Artefakte.jpg]

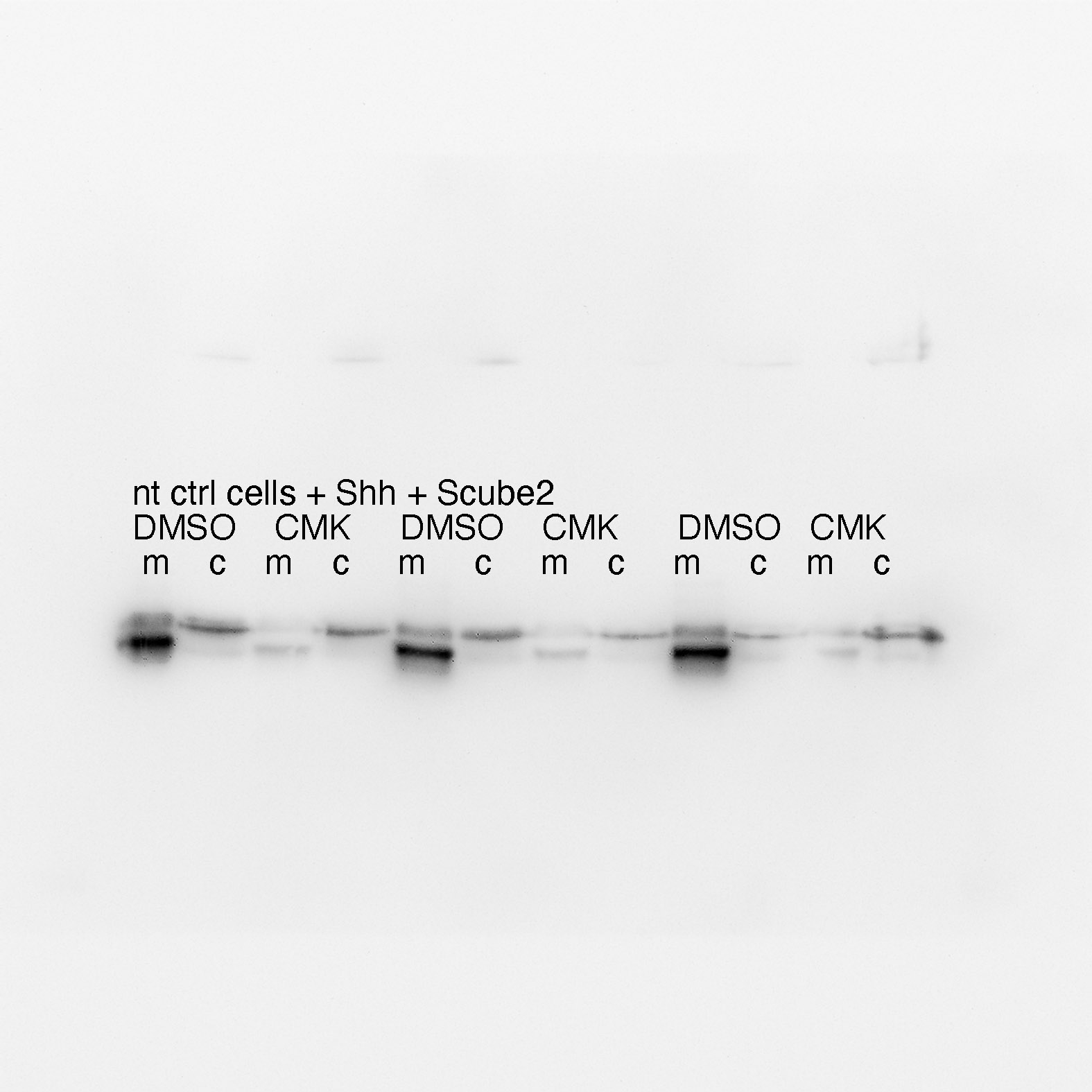

Supplement: Figure 2—figure supplement 1—source data 1. [file elife-86920-fig2-figsupp1-data1.zip › Figure 2-Figure Supplement 1 - Source Data 1/A_20200511_VK122_Gel1_anti-Shh_57sec_16bit_ohne Artefakte labelled.jpg]

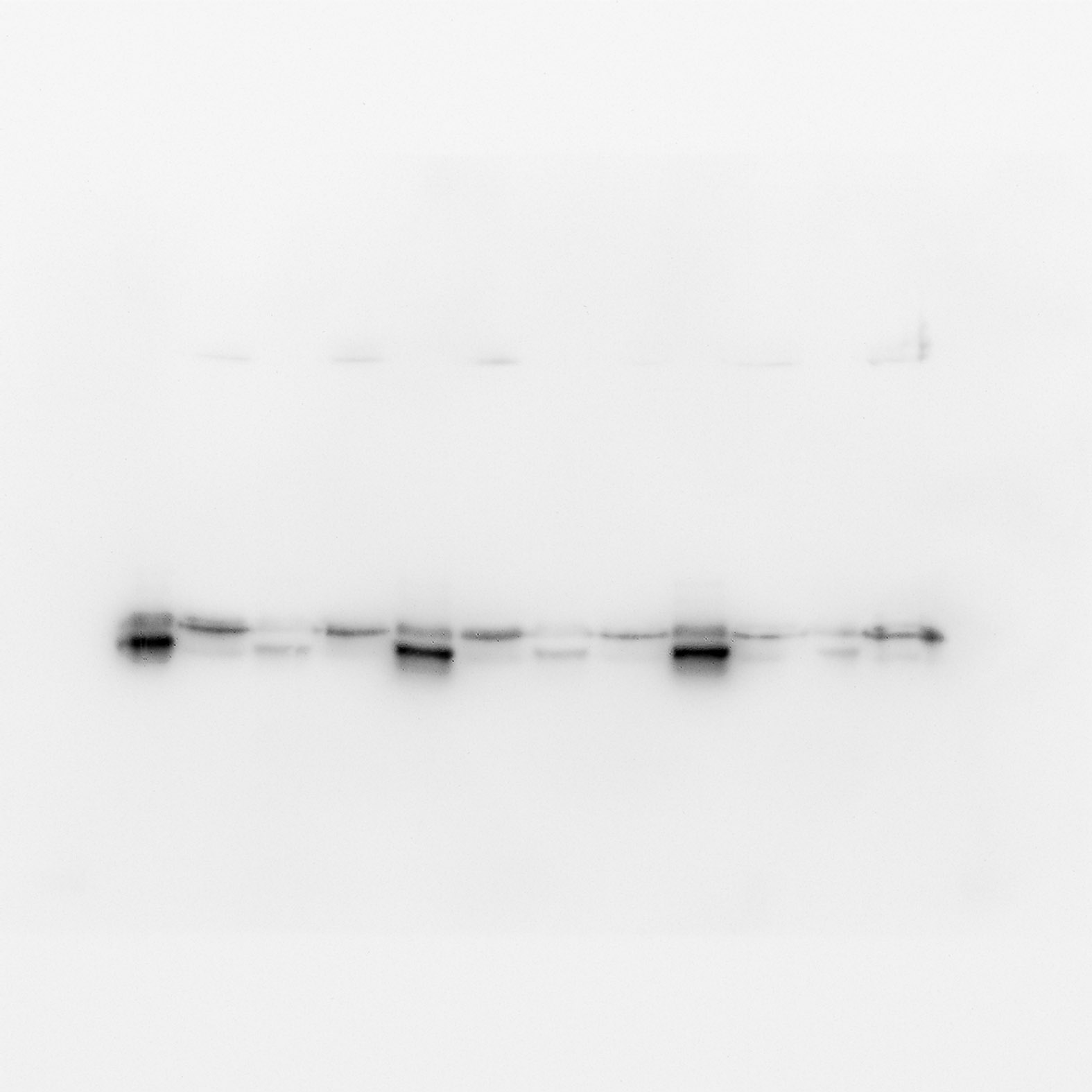

Supplement: Figure 2—figure supplement 1—source data 1. [file elife-86920-fig2-figsupp1-data1.zip › Figure 2-Figure Supplement 1 - Source Data 1/A_20200511_VK122_Gel1_anti-Shh_57sec_16bit_ohne Artefakte.jpg]

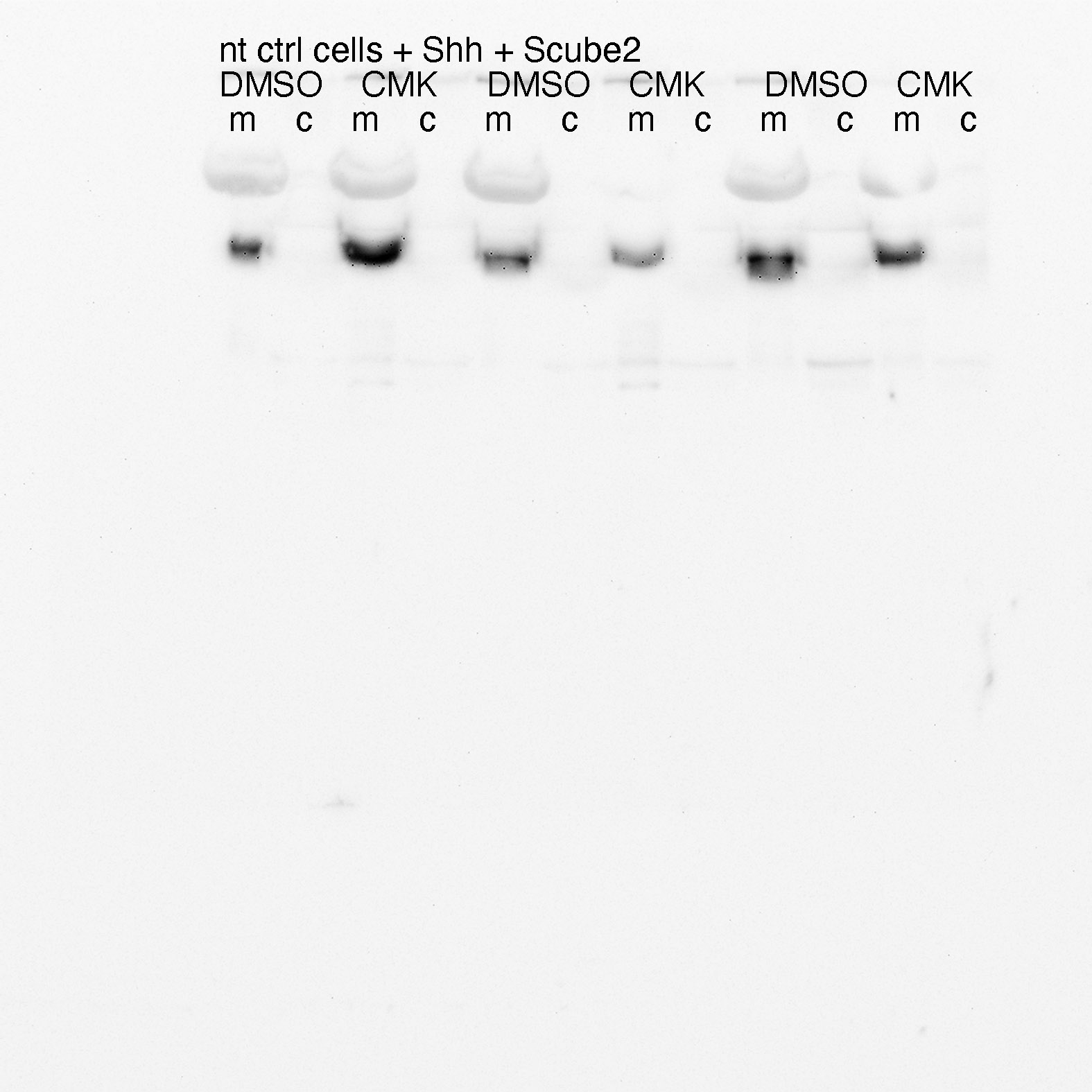

Supplement: Figure 2—figure supplement 1—source data 1. [file elife-86920-fig2-figsupp1-data1.zip › Figure 2-Figure Supplement 1 - Source Data 1/A_VK122_120sek_Blot1_Anti-Flag_16bit labelled.jpg]

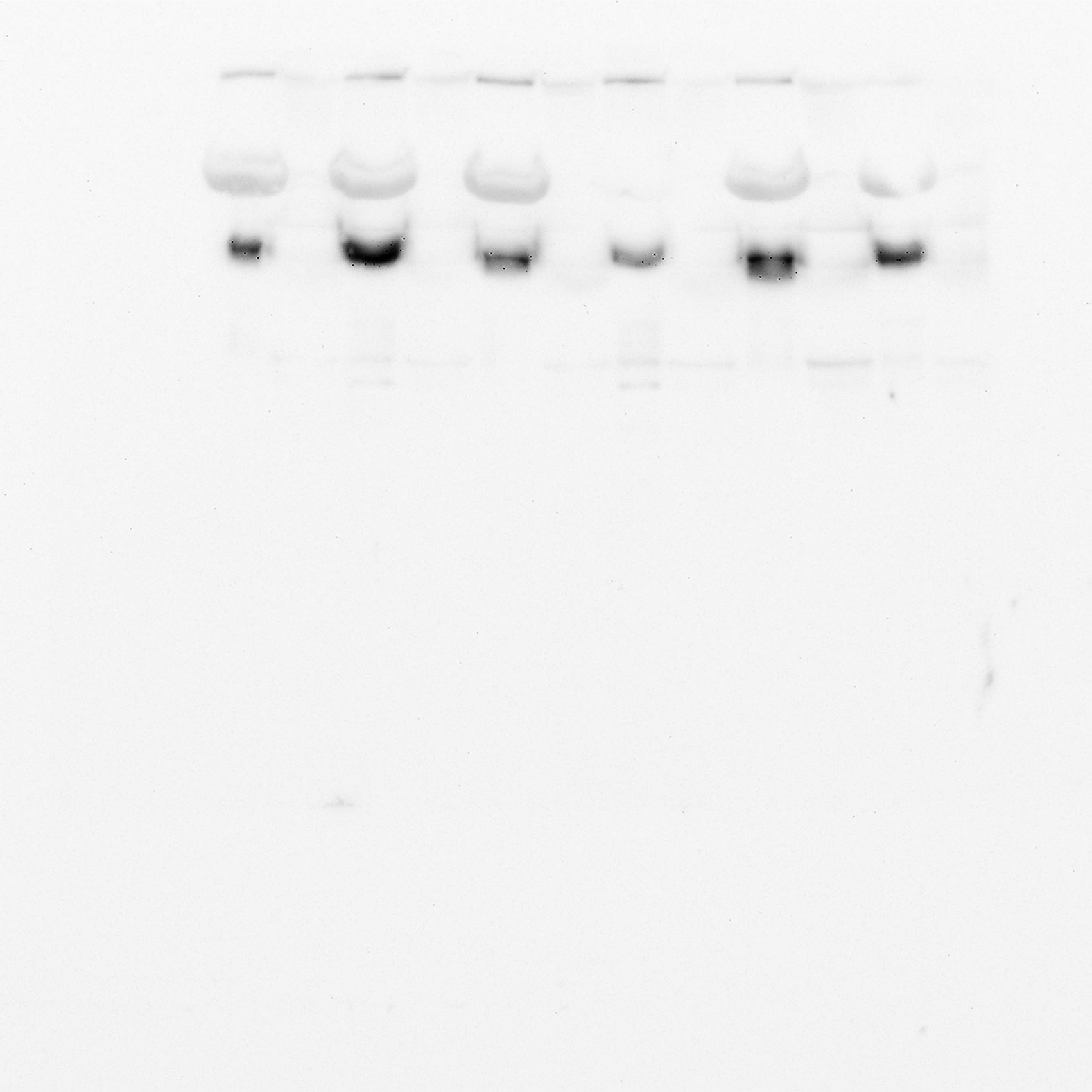

Supplement: Figure 2—figure supplement 1—source data 1. [file elife-86920-fig2-figsupp1-data1.zip › Figure 2-Figure Supplement 1 - Source Data 1/A_VK122_120sek_Blot1_Anti-Flag_16bit.jpg]

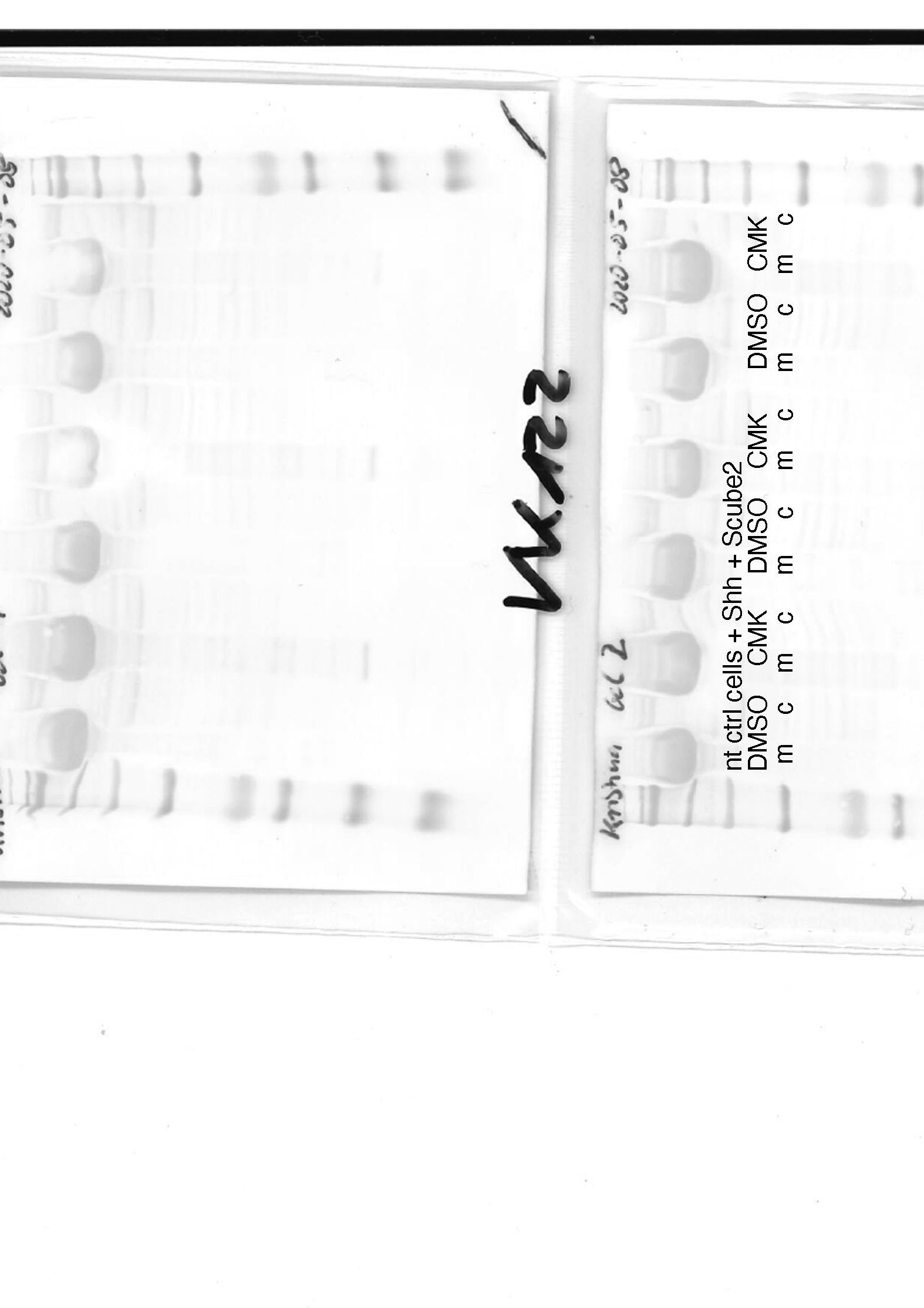

Supplement: Figure 2—figure supplement 1—source data 1. [file elife-86920-fig2-figsupp1-data1.zip › Figure 2-Figure Supplement 1 - Source Data 1/A_VK122_Ponceau Fa╠êrbung labelled.jpg]

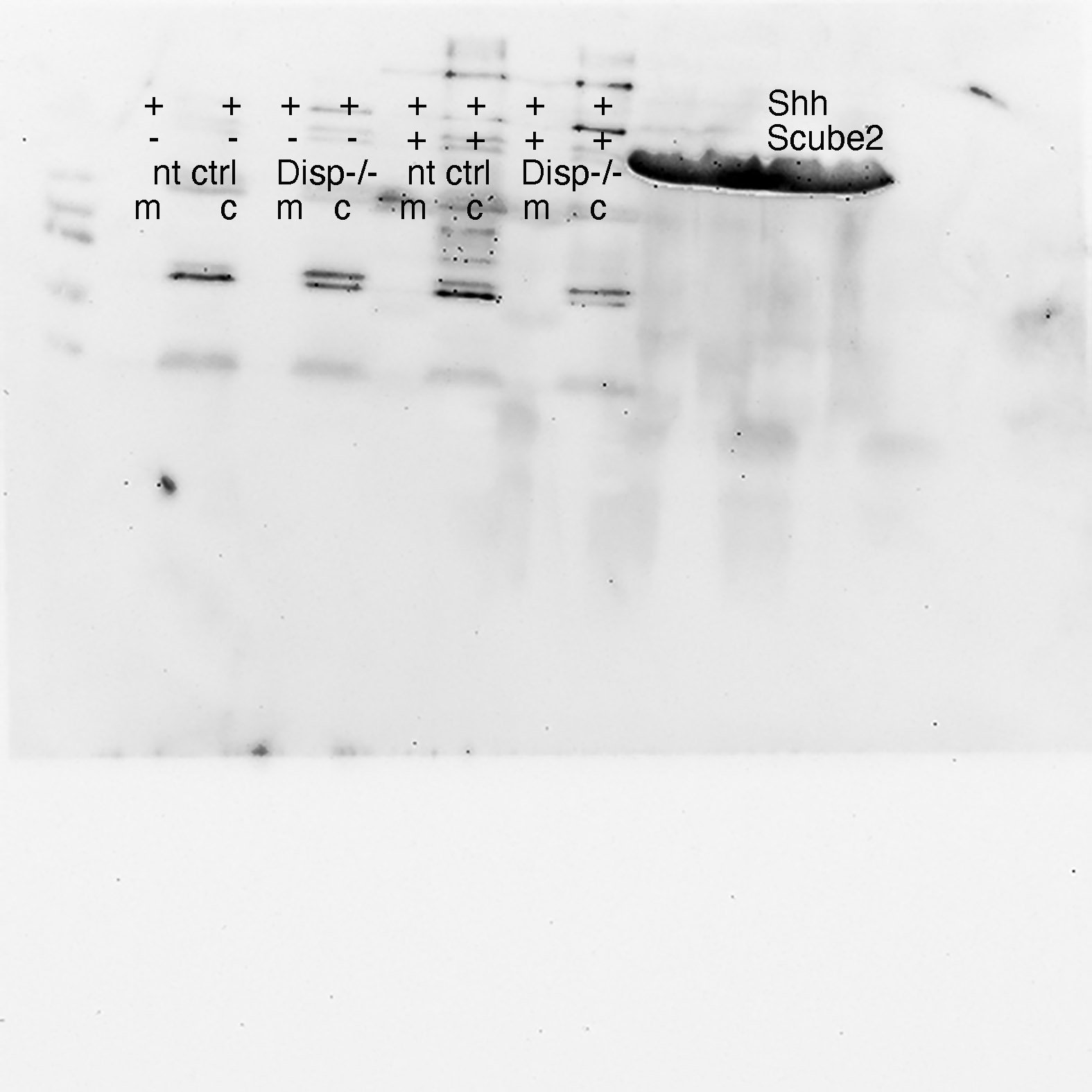

Supplement: Figure 2—figure supplement 1—source data 1. [file elife-86920-fig2-figsupp1-data1.zip › Figure 2-Figure Supplement 1 - Source Data 1/B_18-Feb_2022_4x4 16Bit_752-1_antiFlag_26sec labelled.jpg]

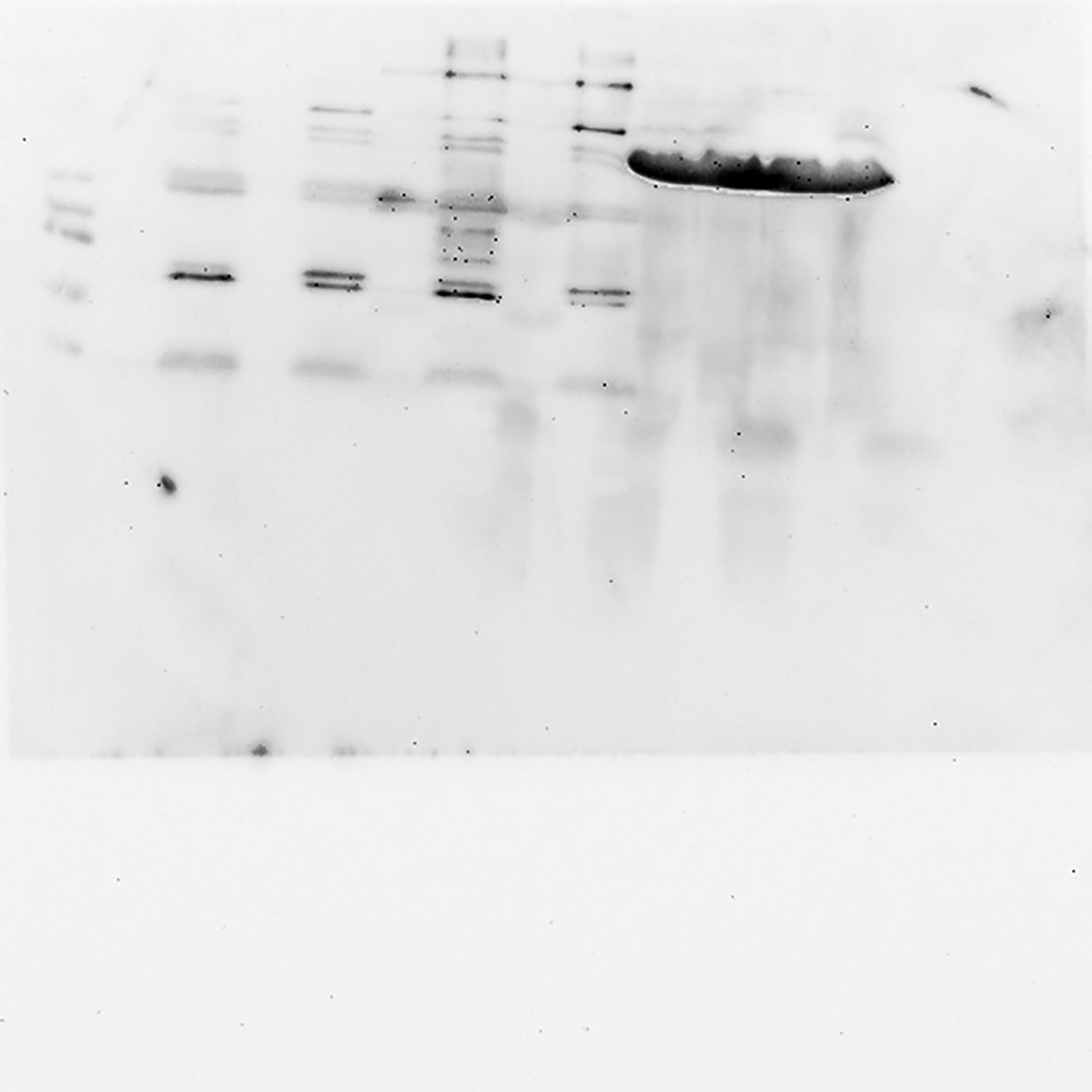

Supplement: Figure 2—figure supplement 1—source data 1. [file elife-86920-fig2-figsupp1-data1.zip › Figure 2-Figure Supplement 1 - Source Data 1/B_18-Feb_2022_4x4 16Bit_752-1_antiFlag_26sec.jpg]

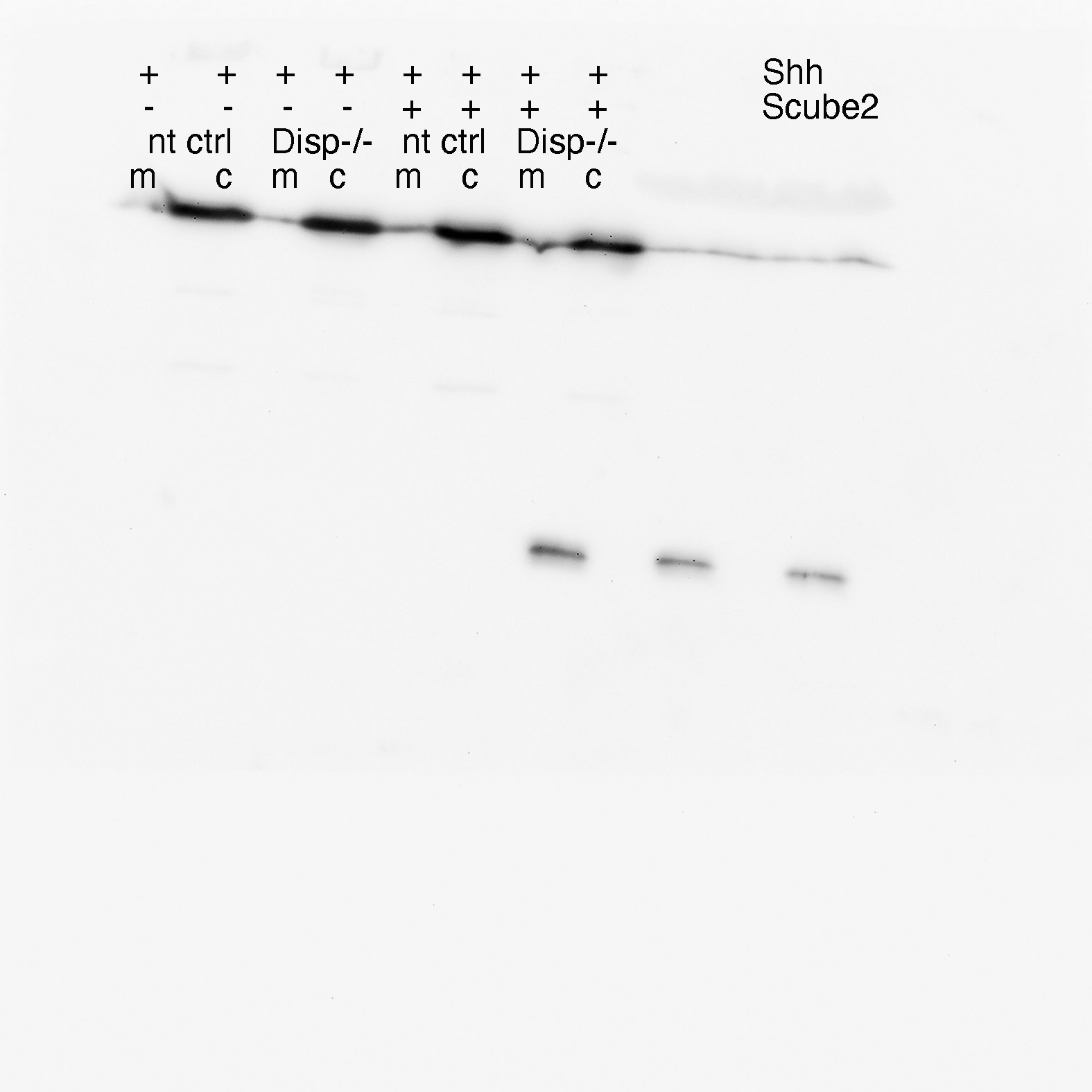

Supplement: Figure 2—figure supplement 1—source data 1. [file elife-86920-fig2-figsupp1-data1.zip › Figure 2-Figure Supplement 1 - Source Data 1/B_21-02-2022_16Bit_V753-1_anti Actin_1min labelled.jpg]

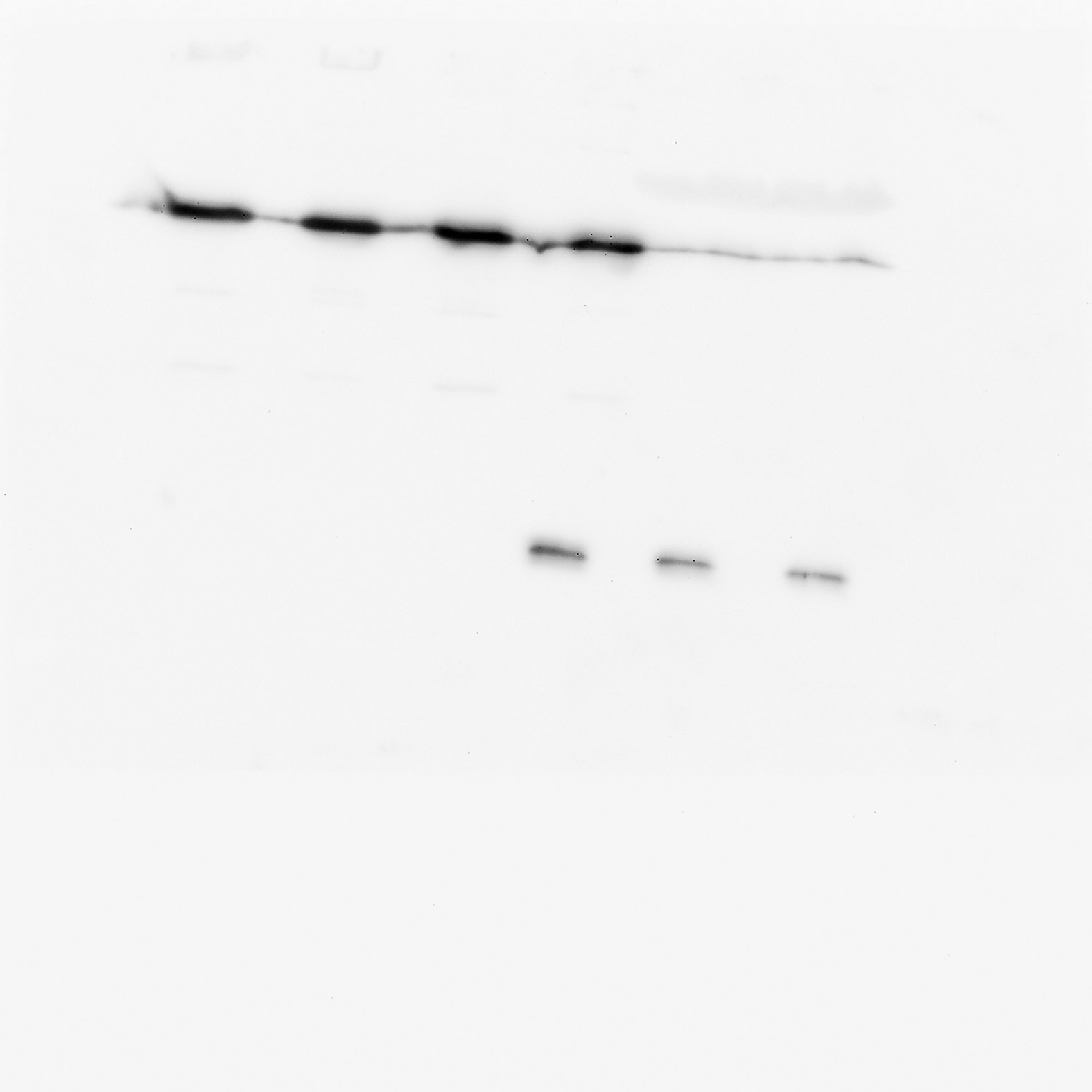

Supplement: Figure 2—figure supplement 1—source data 1. [file elife-86920-fig2-figsupp1-data1.zip › Figure 2-Figure Supplement 1 - Source Data 1/B_21-02-2022_16Bit_V753-1_anti Actin_1min.jpg]

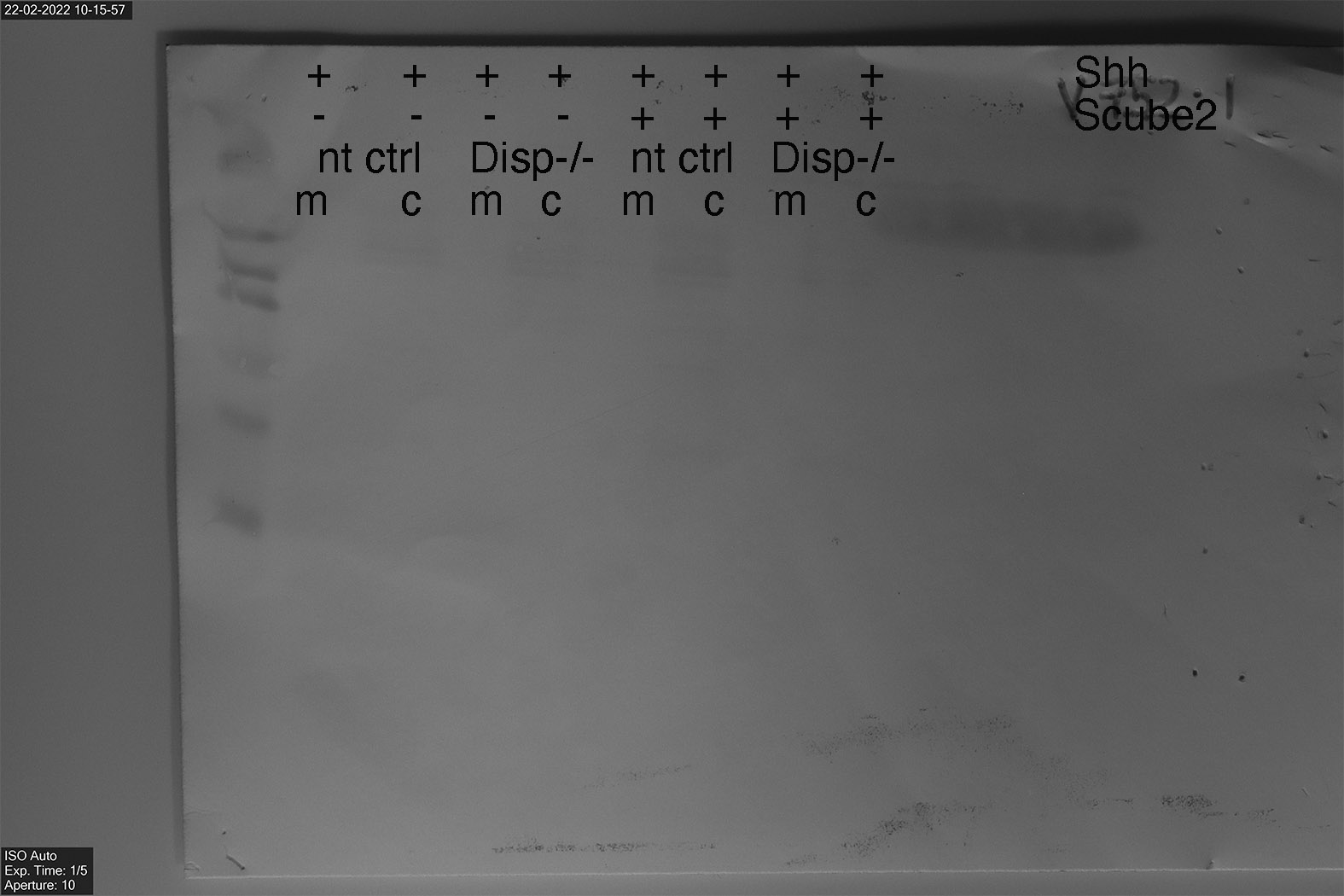

Supplement: Figure 2—figure supplement 1—source data 1. [file elife-86920-fig2-figsupp1-data1.zip › Figure 2-Figure Supplement 1 - Source Data 1/B_22-Feb-22 V572-1 Poc-rotB labelled.jpg]

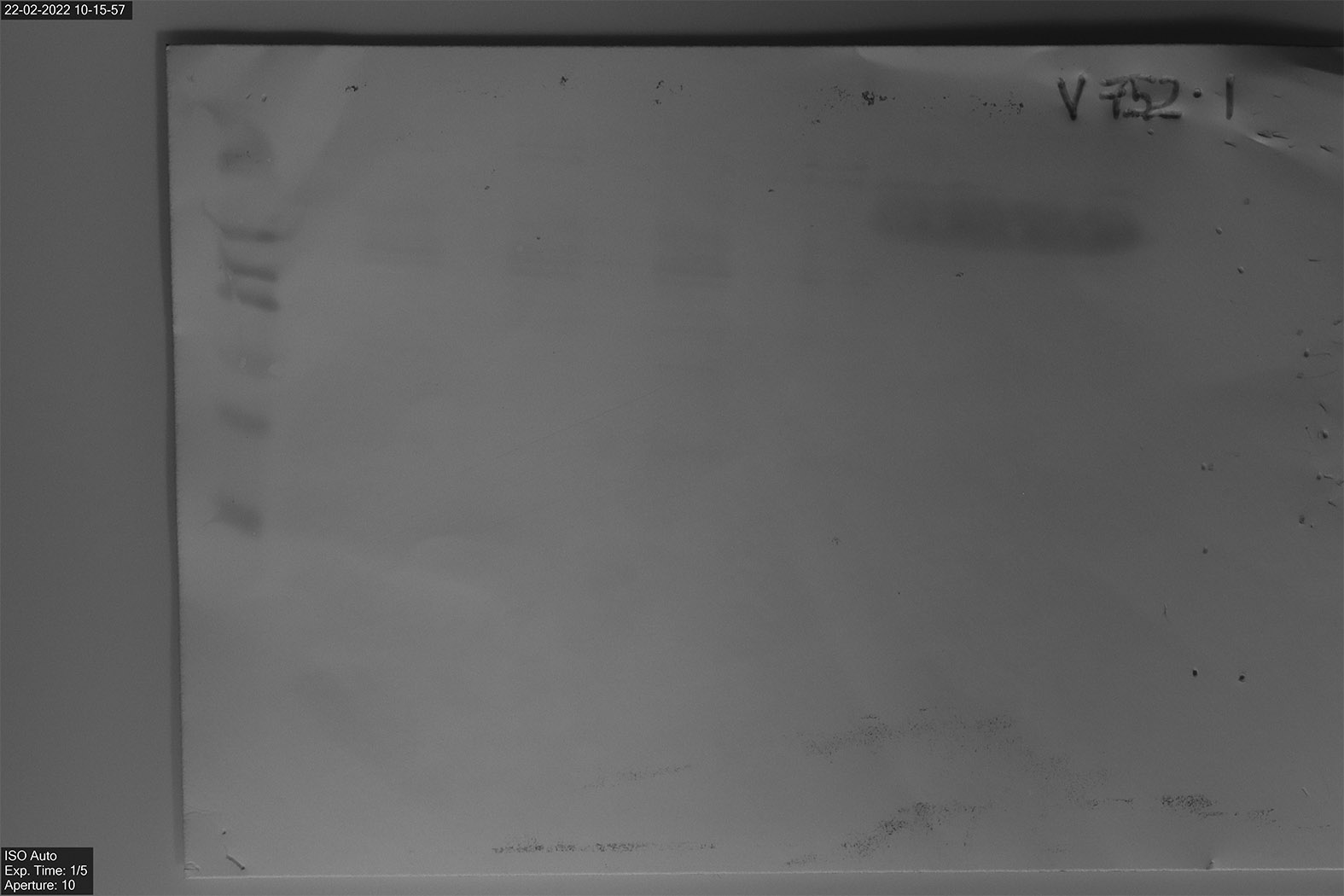

Supplement: Figure 2—figure supplement 1—source data 1. [file elife-86920-fig2-figsupp1-data1.zip › Figure 2-Figure Supplement 1 - Source Data 1/B_22-Feb-22 V572-1 Poc-rotB.jpg]

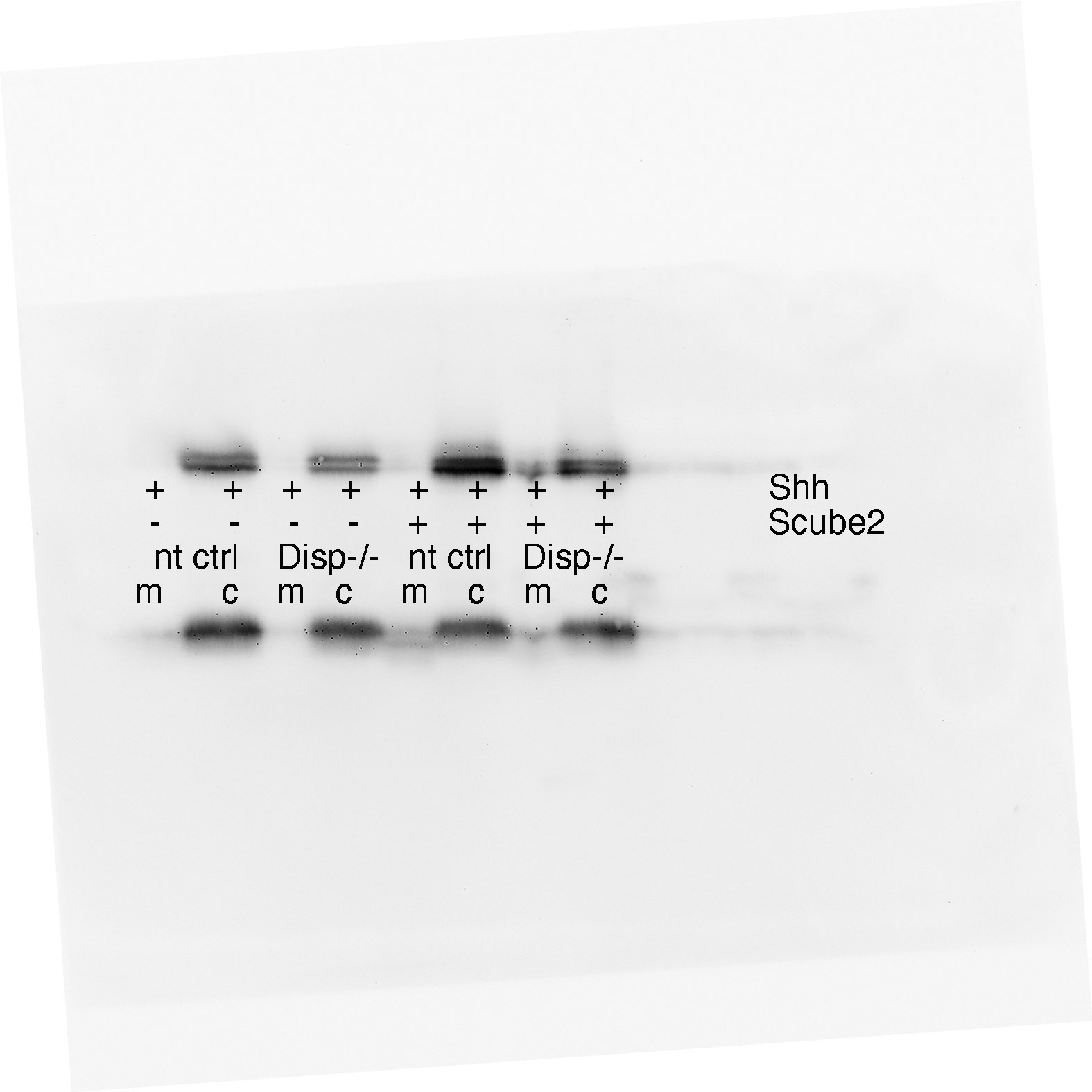

Supplement: Figure 2—figure supplement 1—source data 1. [file elife-86920-fig2-figsupp1-data1.zip › Figure 2-Figure Supplement 1 - Source Data 1/B_V752_1_Shh_1min labelled.jpg]

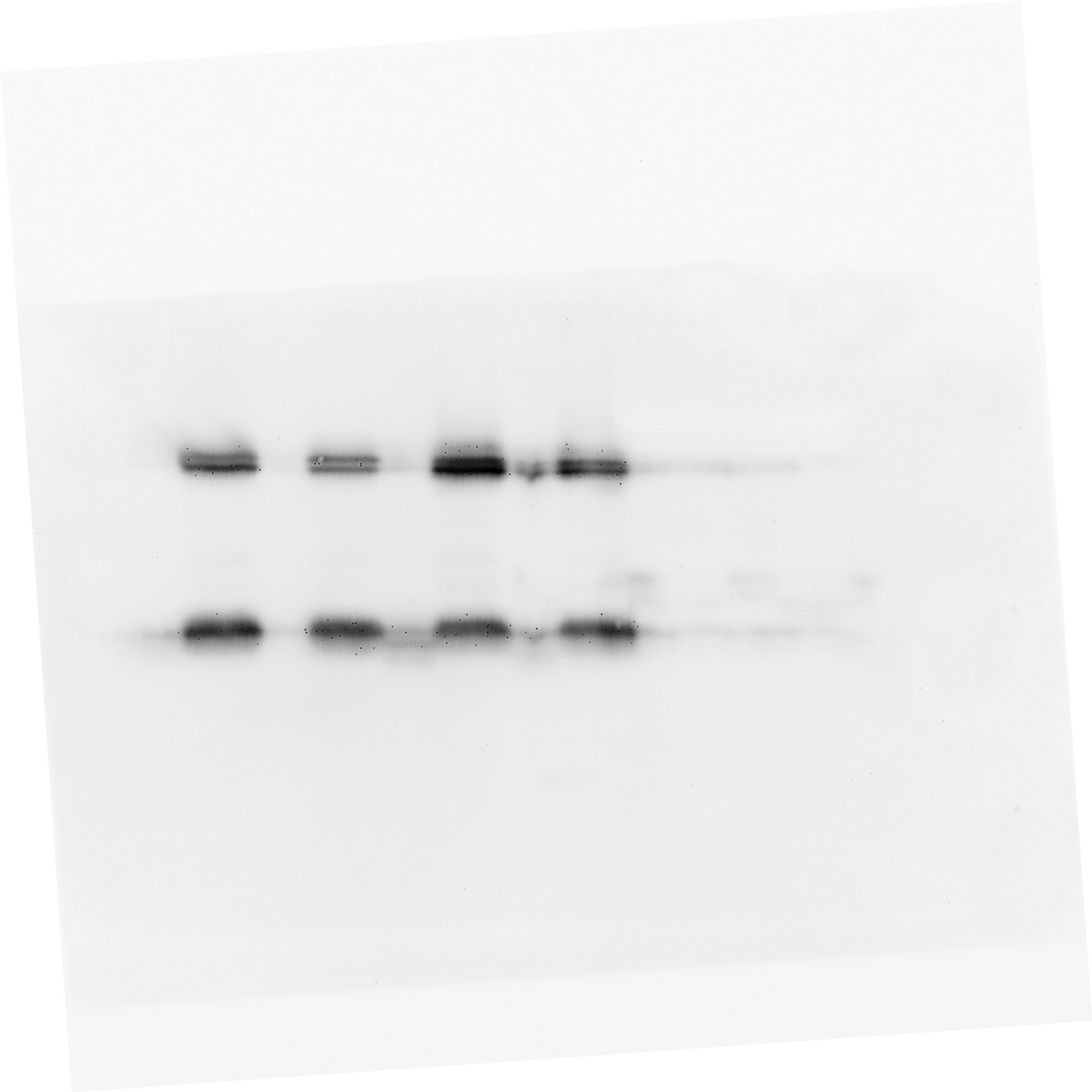

Supplement: Figure 2—figure supplement 1—source data 1. [file elife-86920-fig2-figsupp1-data1.zip › Figure 2-Figure Supplement 1 - Source Data 1/B_V752_1_Shh_1min.jpg]

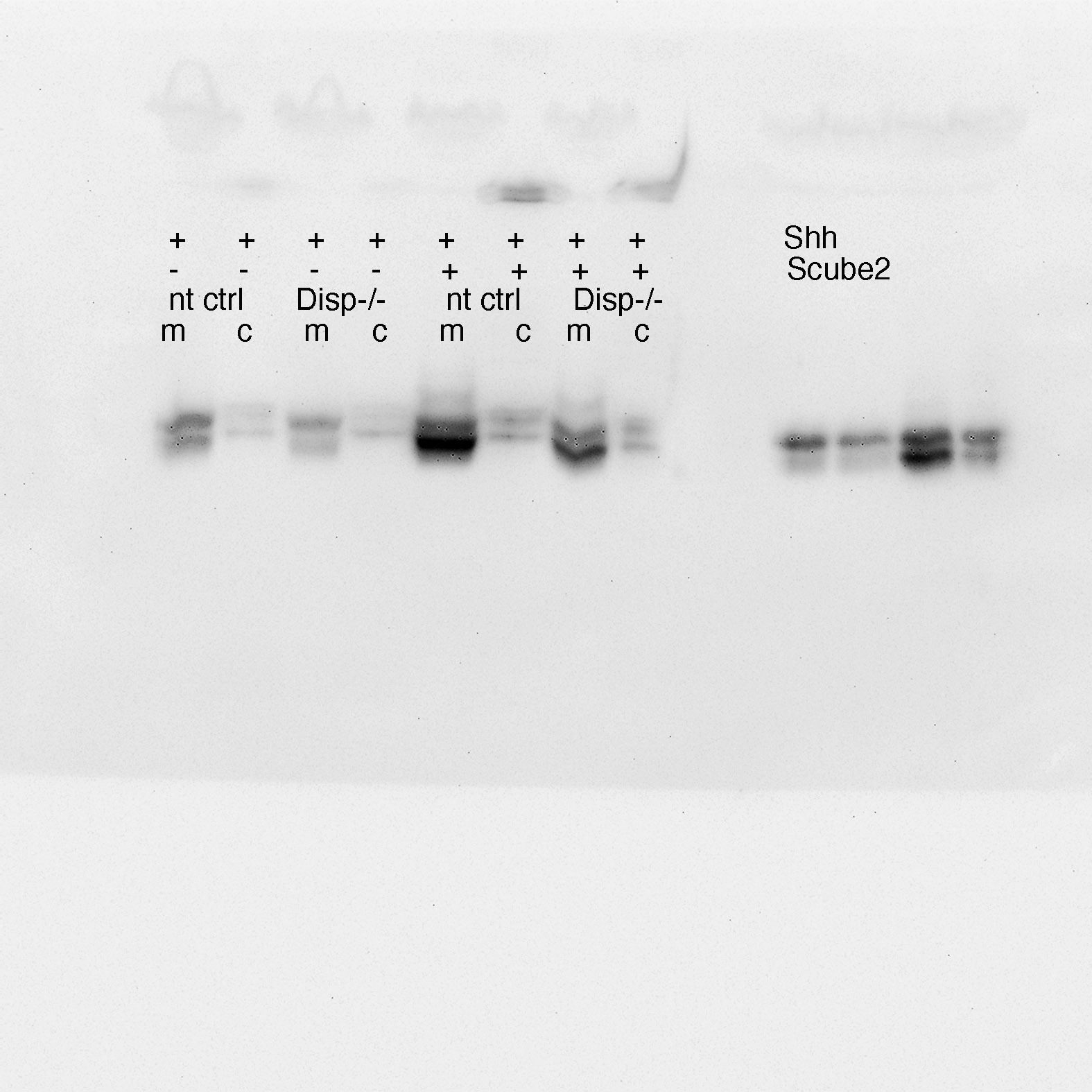

Supplement: Figure 3—source data 1. — A–D contain uncropped western blots shown in Figure 3A–D. Prizm files contain all raw data and statistical analysis to quantify serum-dependent Shh release. B’–E contain uncropped western blots used for the quantification. D` quantifies truncated (proteolytically processed) solubilized Shh, E quantifies relative amounts of unprocessed Shh in media. A’–D’’ Excel file containing raw Shh RP-HPLC elution data as shown in Figure 3A’’–D’’. [file elife-86920-fig3-data1.zip › Figure_3_Source_Data_1 /A_antiShh_serumdepleted labelled.jpg]

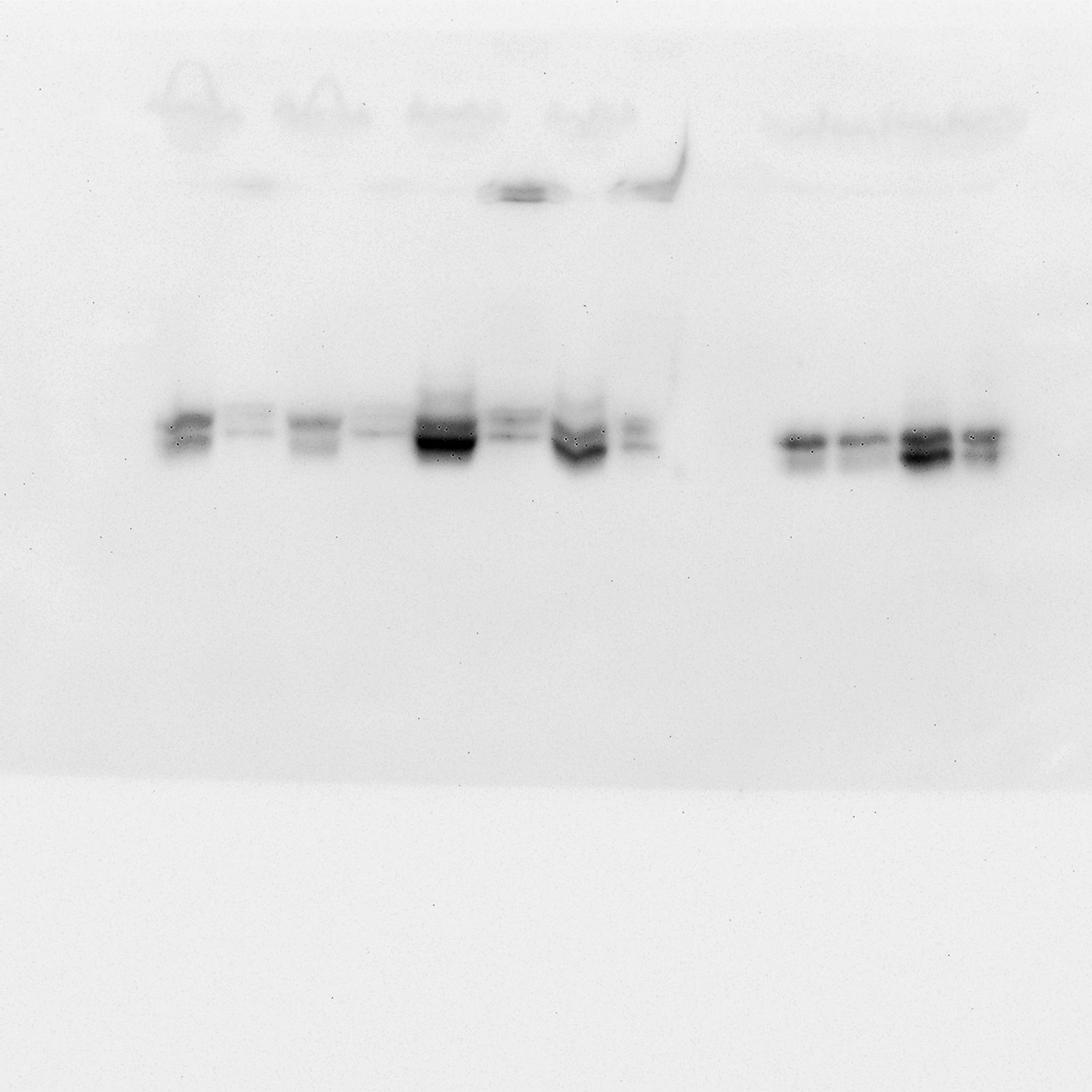

Supplement: Figure 3—source data 1. — A–D contain uncropped western blots shown in Figure 3A–D. Prizm files contain all raw data and statistical analysis to quantify serum-dependent Shh release. B’–E contain uncropped western blots used for the quantification. D` quantifies truncated (proteolytically processed) solubilized Shh, E quantifies relative amounts of unprocessed Shh in media. A’–D’’ Excel file containing raw Shh RP-HPLC elution data as shown in Figure 3A’’–D’’. [file elife-86920-fig3-data1.zip › Figure_3_Source_Data_1 /A_antiShh_serumdepleted.jpg]

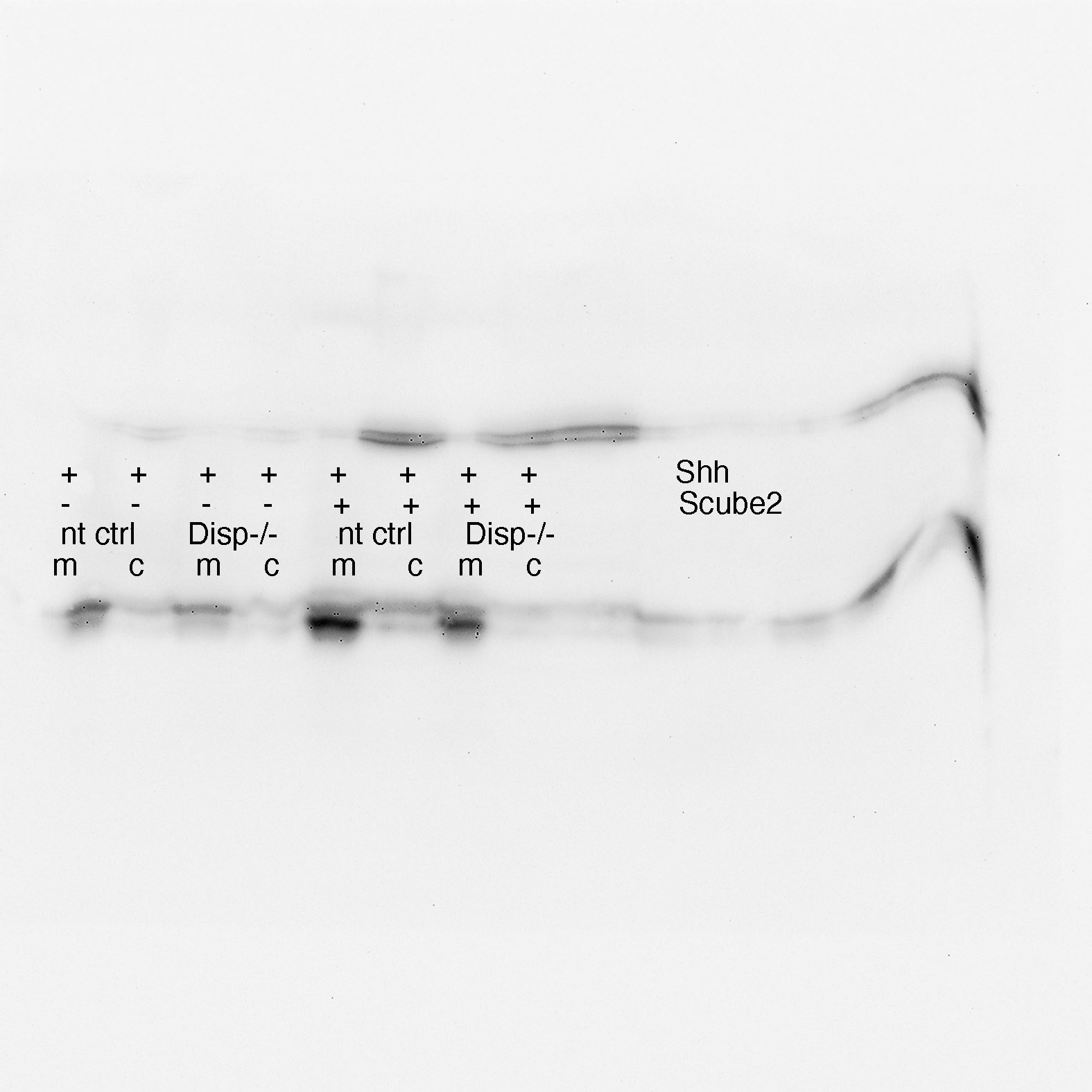

Supplement: Figure 3—source data 1. — A–D contain uncropped western blots shown in Figure 3A–D. Prizm files contain all raw data and statistical analysis to quantify serum-dependent Shh release. B’–E contain uncropped western blots used for the quantification. D` quantifies truncated (proteolytically processed) solubilized Shh, E quantifies relative amounts of unprocessed Shh in media. A’–D’’ Excel file containing raw Shh RP-HPLC elution data as shown in Figure 3A’’–D’’. [file elife-86920-fig3-data1.zip › Figure_3_Source_Data_1 /B_V744_serum_005% labelled.jpg]

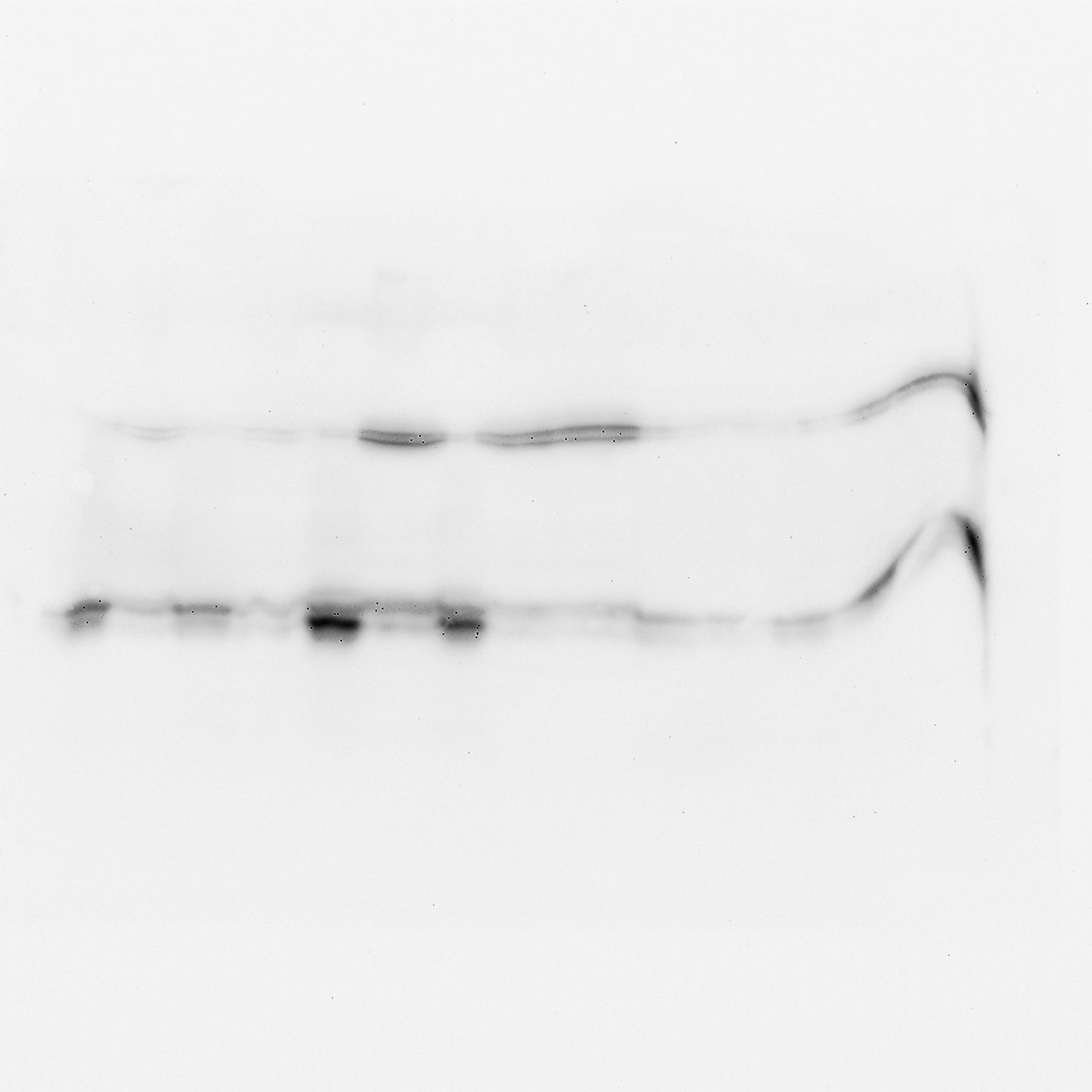

Supplement: Figure 3—source data 1. — A–D contain uncropped western blots shown in Figure 3A–D. Prizm files contain all raw data and statistical analysis to quantify serum-dependent Shh release. B’–E contain uncropped western blots used for the quantification. D` quantifies truncated (proteolytically processed) solubilized Shh, E quantifies relative amounts of unprocessed Shh in media. A’–D’’ Excel file containing raw Shh RP-HPLC elution data as shown in Figure 3A’’–D’’. [file elife-86920-fig3-data1.zip › Figure_3_Source_Data_1 /B_V744_serum_005%.jpg]

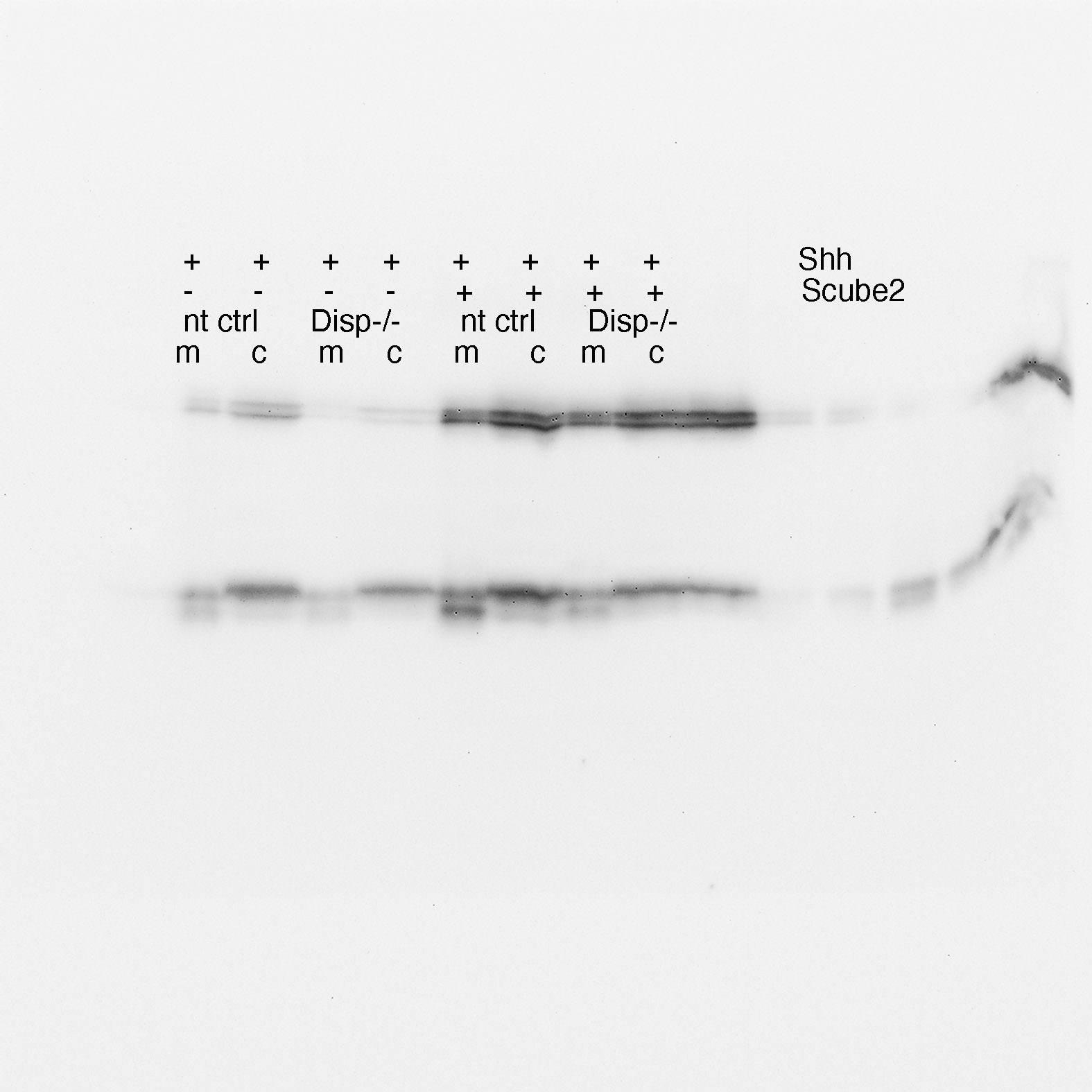

Supplement: Figure 3—source data 1. — A–D contain uncropped western blots shown in Figure 3A–D. Prizm files contain all raw data and statistical analysis to quantify serum-dependent Shh release. B’–E contain uncropped western blots used for the quantification. D` quantifies truncated (proteolytically processed) solubilized Shh, E quantifies relative amounts of unprocessed Shh in media. A’–D’’ Excel file containing raw Shh RP-HPLC elution data as shown in Figure 3A’’–D’’. [file elife-86920-fig3-data1.zip › Figure_3_Source_Data_1 /C_V744_serum_5% labelled.jpg]

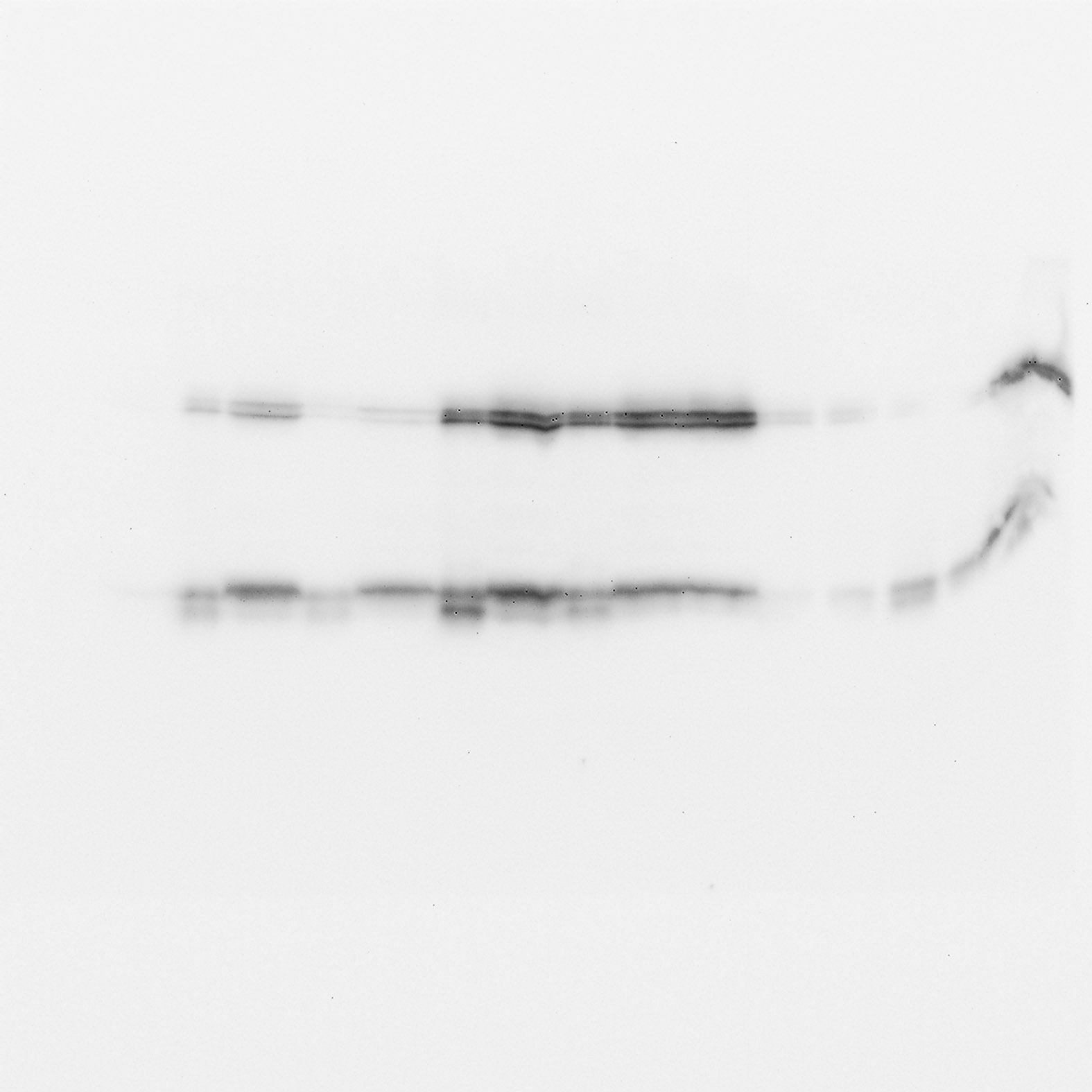

Supplement: Figure 3—source data 1. — A–D contain uncropped western blots shown in Figure 3A–D. Prizm files contain all raw data and statistical analysis to quantify serum-dependent Shh release. B’–E contain uncropped western blots used for the quantification. D` quantifies truncated (proteolytically processed) solubilized Shh, E quantifies relative amounts of unprocessed Shh in media. A’–D’’ Excel file containing raw Shh RP-HPLC elution data as shown in Figure 3A’’–D’’. [file elife-86920-fig3-data1.zip › Figure_3_Source_Data_1 /C_V744_serum_5%.jpg]

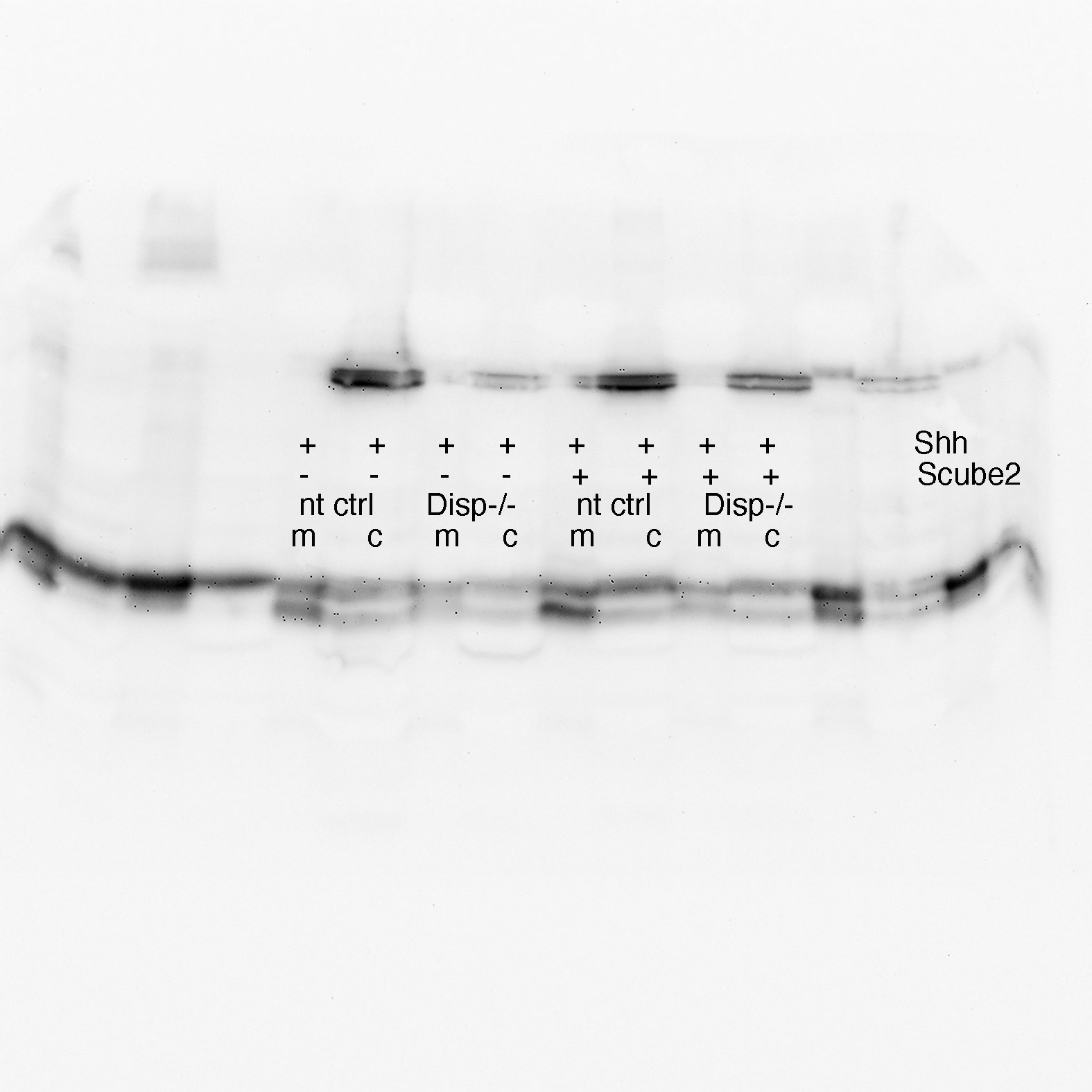

Supplement: Figure 3—source data 1. — A–D contain uncropped western blots shown in Figure 3A–D. Prizm files contain all raw data and statistical analysis to quantify serum-dependent Shh release. B’–E contain uncropped western blots used for the quantification. D` quantifies truncated (proteolytically processed) solubilized Shh, E quantifies relative amounts of unprocessed Shh in media. A’–D’’ Excel file containing raw Shh RP-HPLC elution data as shown in Figure 3A’’–D’’. [file elife-86920-fig3-data1.zip › Figure_3_Source_Data_1 /D_V742_serum_10% labelled.jpg]

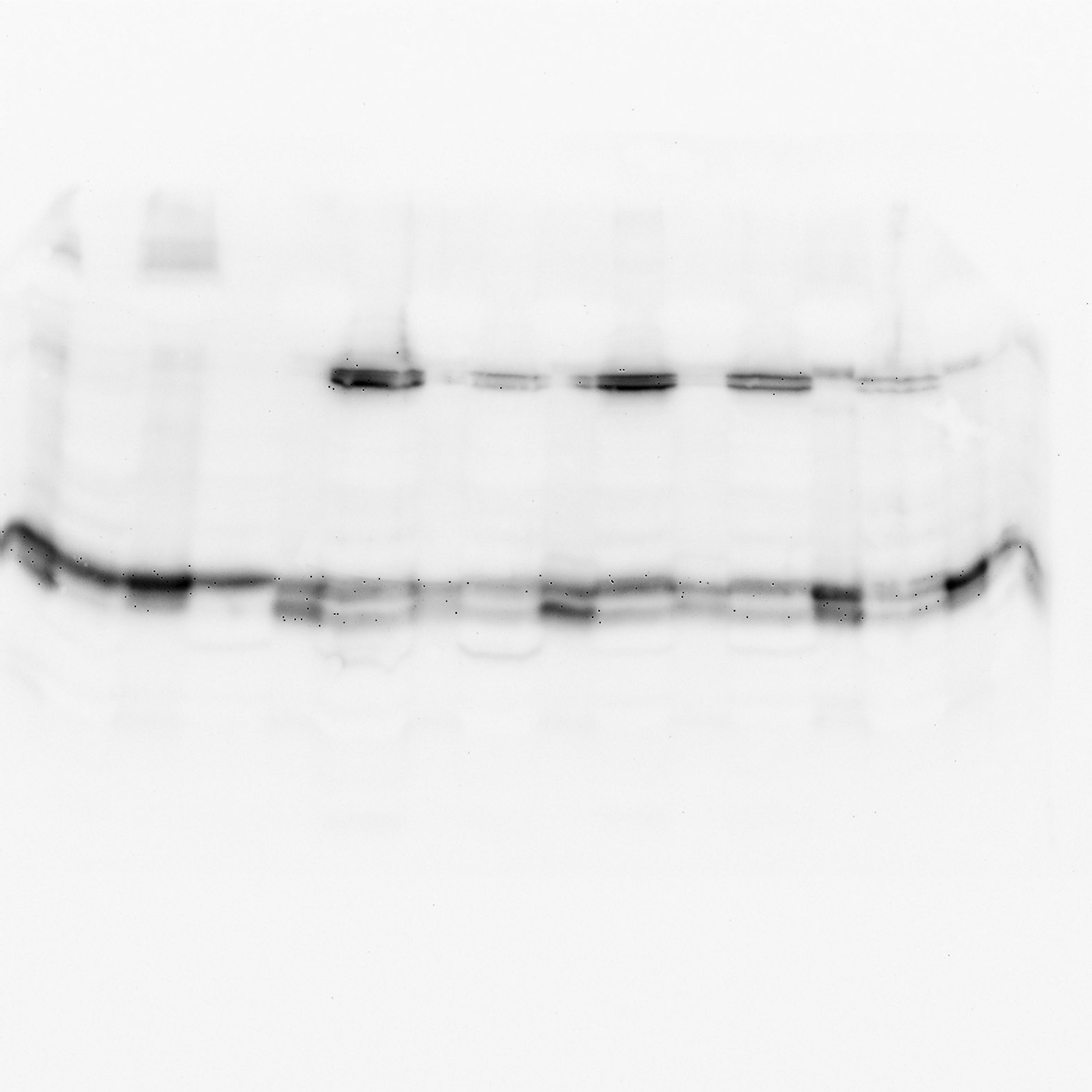

Supplement: Figure 3—source data 1. — A–D contain uncropped western blots shown in Figure 3A–D. Prizm files contain all raw data and statistical analysis to quantify serum-dependent Shh release. B’–E contain uncropped western blots used for the quantification. D` quantifies truncated (proteolytically processed) solubilized Shh, E quantifies relative amounts of unprocessed Shh in media. A’–D’’ Excel file containing raw Shh RP-HPLC elution data as shown in Figure 3A’’–D’’. [file elife-86920-fig3-data1.zip › Figure_3_Source_Data_1 /D_V742_serum_10%.jpg]

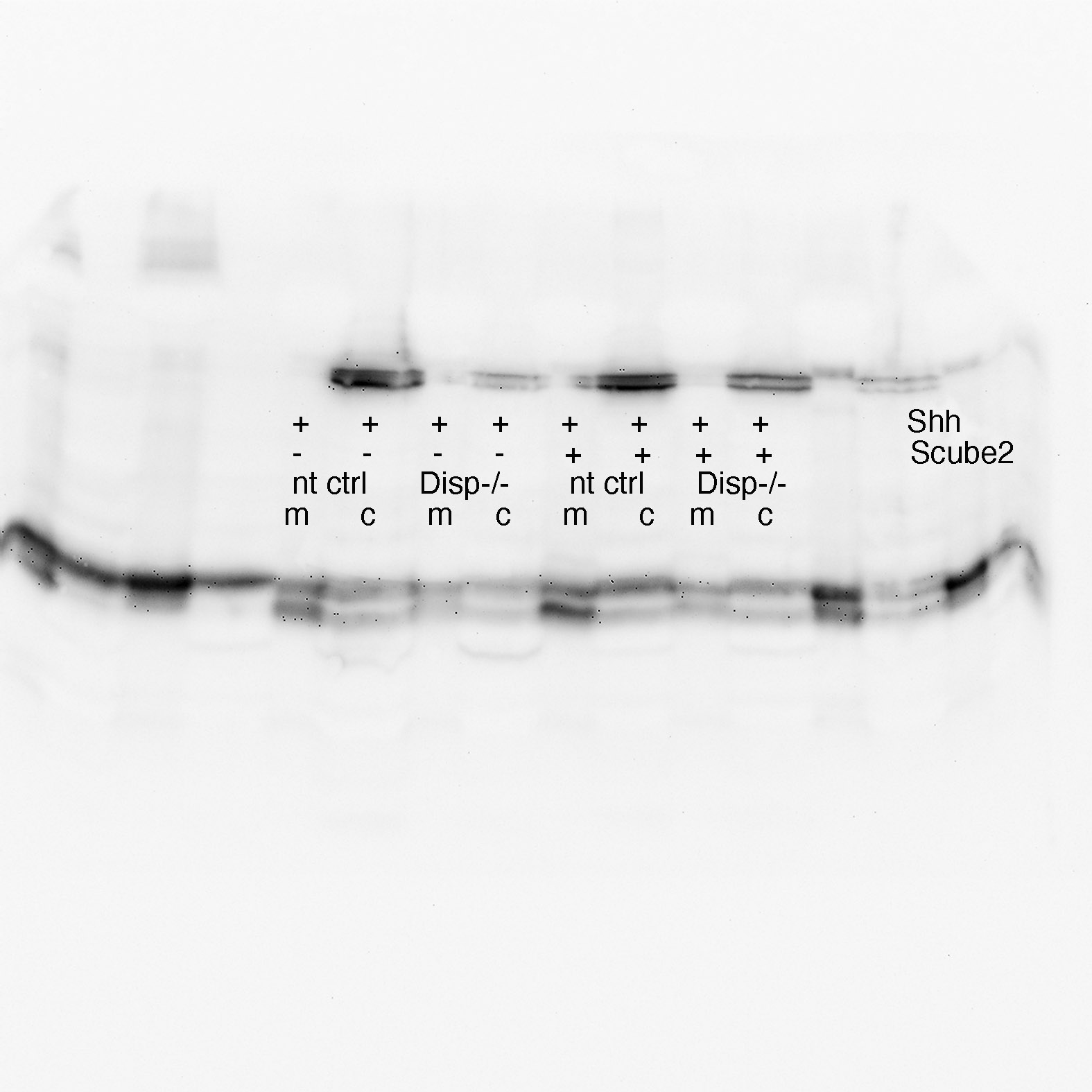

Supplement: Figure 3—source data 1. — A–D contain uncropped western blots shown in Figure 3A–D. Prizm files contain all raw data and statistical analysis to quantify serum-dependent Shh release. B’–E contain uncropped western blots used for the quantification. D` quantifies truncated (proteolytically processed) solubilized Shh, E quantifies relative amounts of unprocessed Shh in media. A’–D’’ Excel file containing raw Shh RP-HPLC elution data as shown in Figure 3A’’–D’’. [file elife-86920-fig3-data1.zip › Figure_3_Source_Data_1 /B'-E_quantification/V742_serum_10%serum labelled.jpg]

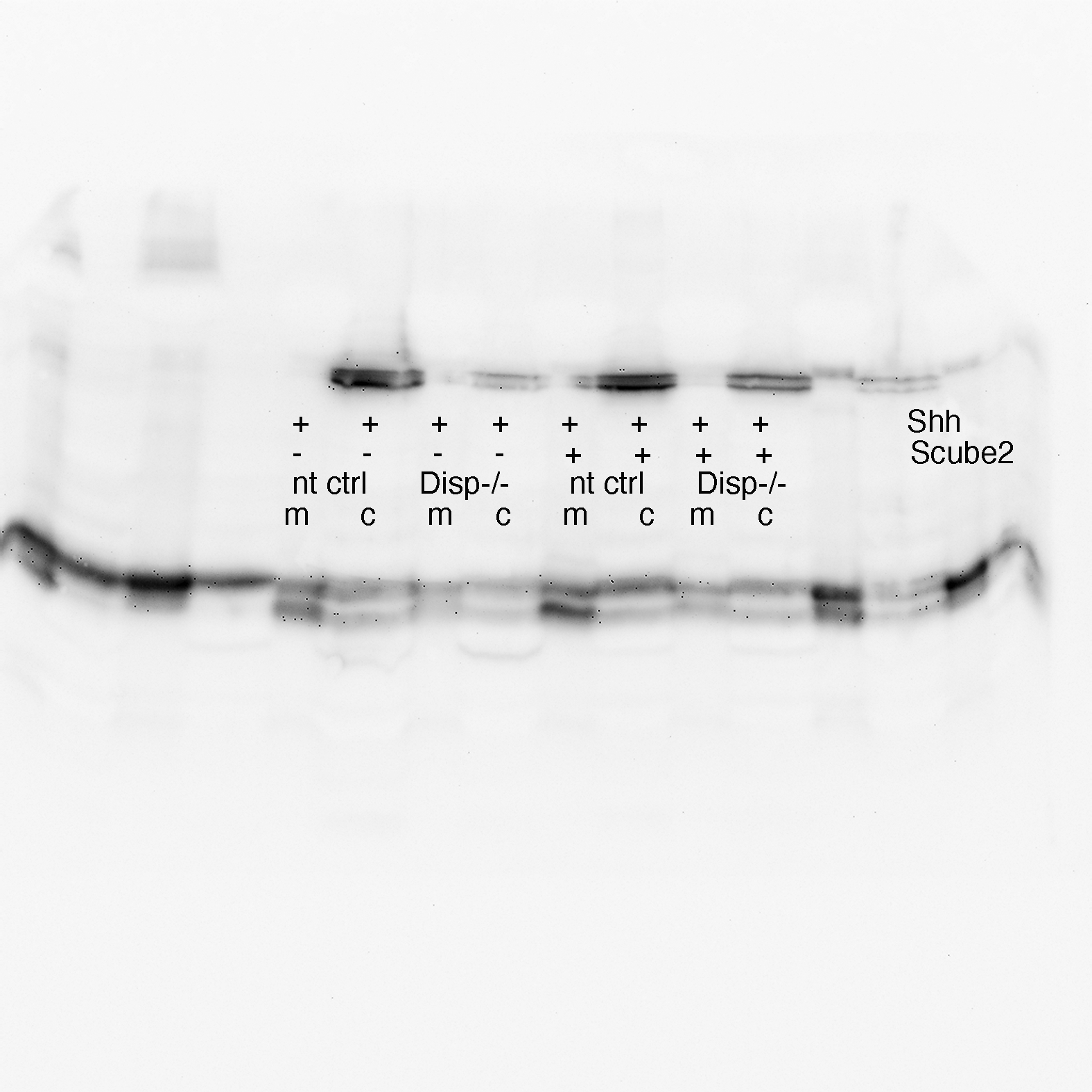

Supplement: Figure 3—source data 1. — A–D contain uncropped western blots shown in Figure 3A–D. Prizm files contain all raw data and statistical analysis to quantify serum-dependent Shh release. B’–E contain uncropped western blots used for the quantification. D` quantifies truncated (proteolytically processed) solubilized Shh, E quantifies relative amounts of unprocessed Shh in media. A’–D’’ Excel file containing raw Shh RP-HPLC elution data as shown in Figure 3A’’–D’’. [file elife-86920-fig3-data1.zip › Figure_3_Source_Data_1 /B'-E_quantification/V742_serum_10%serum labelled.Tif]

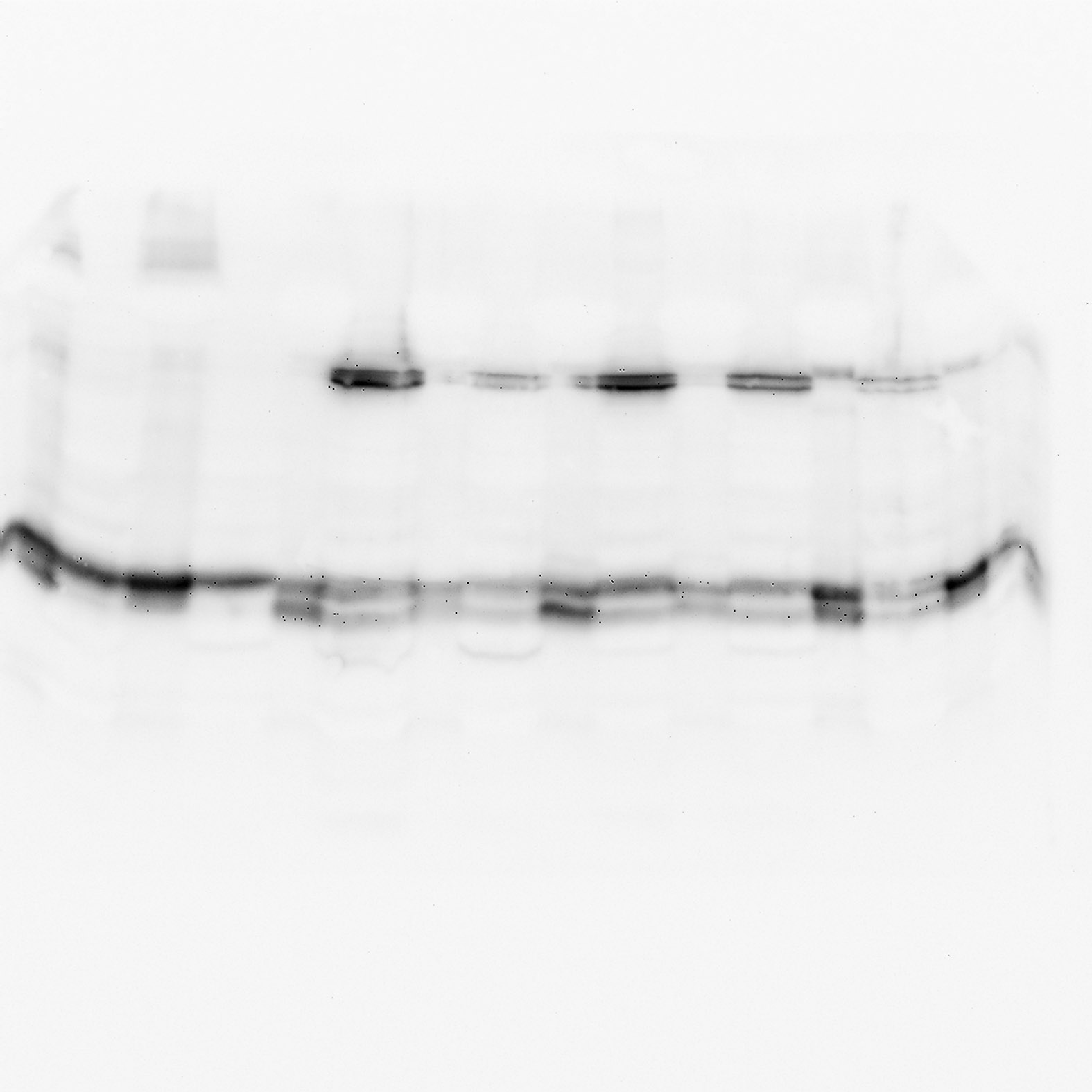

Supplement: Figure 3—source data 1. — A–D contain uncropped western blots shown in Figure 3A–D. Prizm files contain all raw data and statistical analysis to quantify serum-dependent Shh release. B’–E contain uncropped western blots used for the quantification. D` quantifies truncated (proteolytically processed) solubilized Shh, E quantifies relative amounts of unprocessed Shh in media. A’–D’’ Excel file containing raw Shh RP-HPLC elution data as shown in Figure 3A’’–D’’. [file elife-86920-fig3-data1.zip › Figure_3_Source_Data_1 /B'-E_quantification/V742_serum_10%serum.jpg]

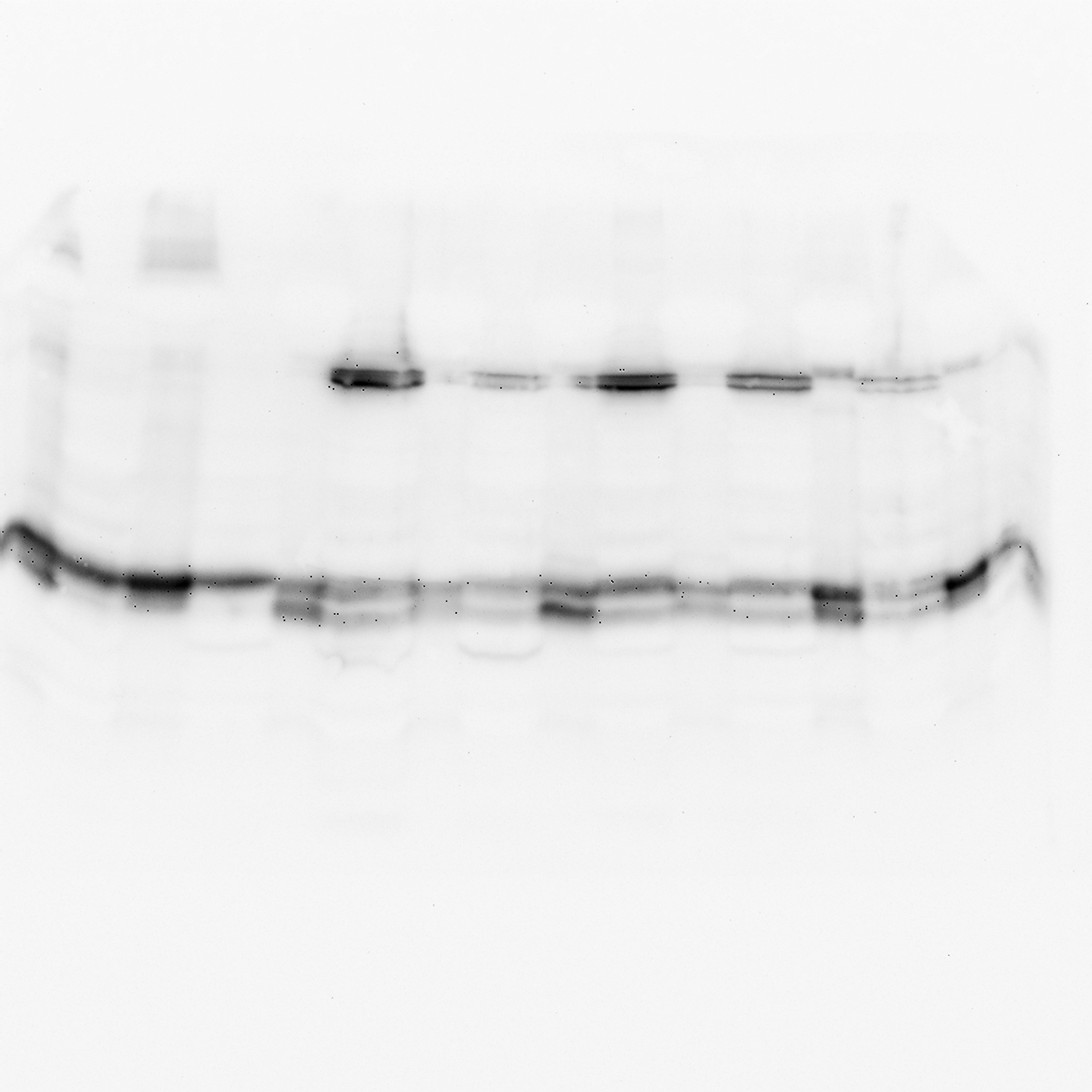

Supplement: Figure 3—source data 1. — A–D contain uncropped western blots shown in Figure 3A–D. Prizm files contain all raw data and statistical analysis to quantify serum-dependent Shh release. B’–E contain uncropped western blots used for the quantification. D` quantifies truncated (proteolytically processed) solubilized Shh, E quantifies relative amounts of unprocessed Shh in media. A’–D’’ Excel file containing raw Shh RP-HPLC elution data as shown in Figure 3A’’–D’’. [file elife-86920-fig3-data1.zip › Figure_3_Source_Data_1 /B'-E_quantification/V742_serum_10%serum.Tif]

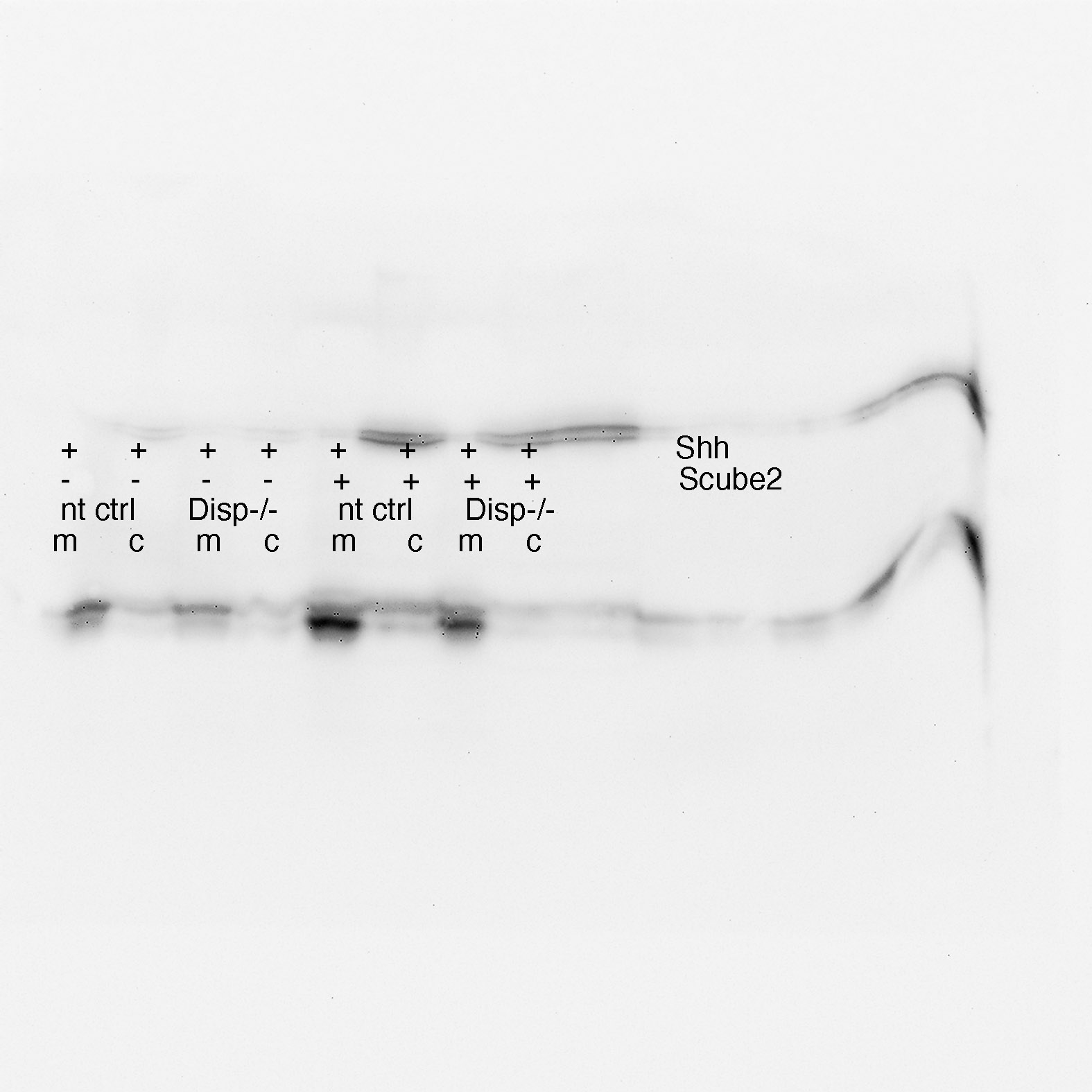

Supplement: Figure 3—source data 1. — A–D contain uncropped western blots shown in Figure 3A–D. Prizm files contain all raw data and statistical analysis to quantify serum-dependent Shh release. B’–E contain uncropped western blots used for the quantification. D` quantifies truncated (proteolytically processed) solubilized Shh, E quantifies relative amounts of unprocessed Shh in media. A’–D’’ Excel file containing raw Shh RP-HPLC elution data as shown in Figure 3A’’–D’’. [file elife-86920-fig3-data1.zip › Figure_3_Source_Data_1 /B'-E_quantification/V744_serum_005%serum labelled.jpg]

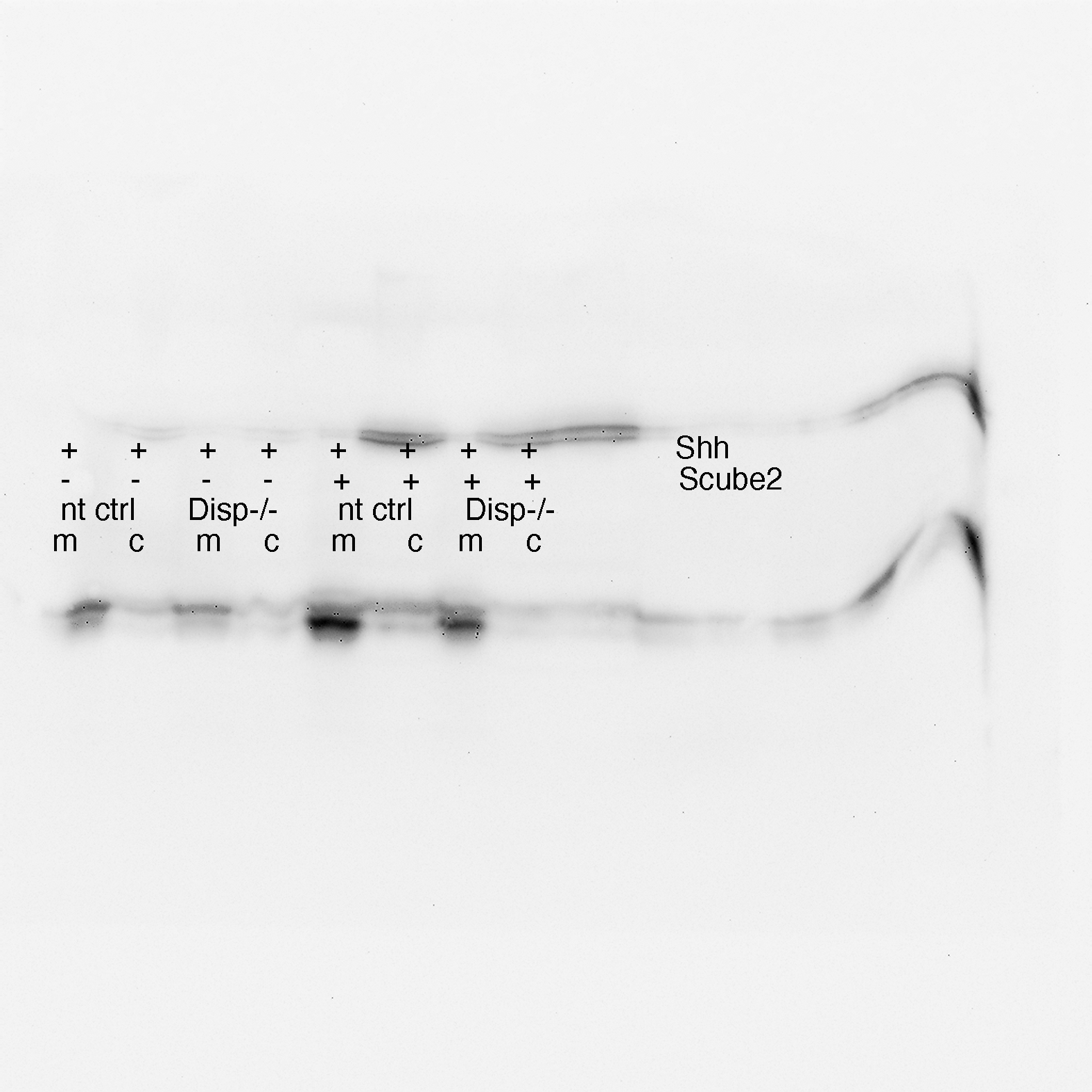

Supplement: Figure 3—source data 1. — A–D contain uncropped western blots shown in Figure 3A–D. Prizm files contain all raw data and statistical analysis to quantify serum-dependent Shh release. B’–E contain uncropped western blots used for the quantification. D` quantifies truncated (proteolytically processed) solubilized Shh, E quantifies relative amounts of unprocessed Shh in media. A’–D’’ Excel file containing raw Shh RP-HPLC elution data as shown in Figure 3A’’–D’’. [file elife-86920-fig3-data1.zip › Figure_3_Source_Data_1 /B'-E_quantification/V744_serum_005%serum labelled.Tif]

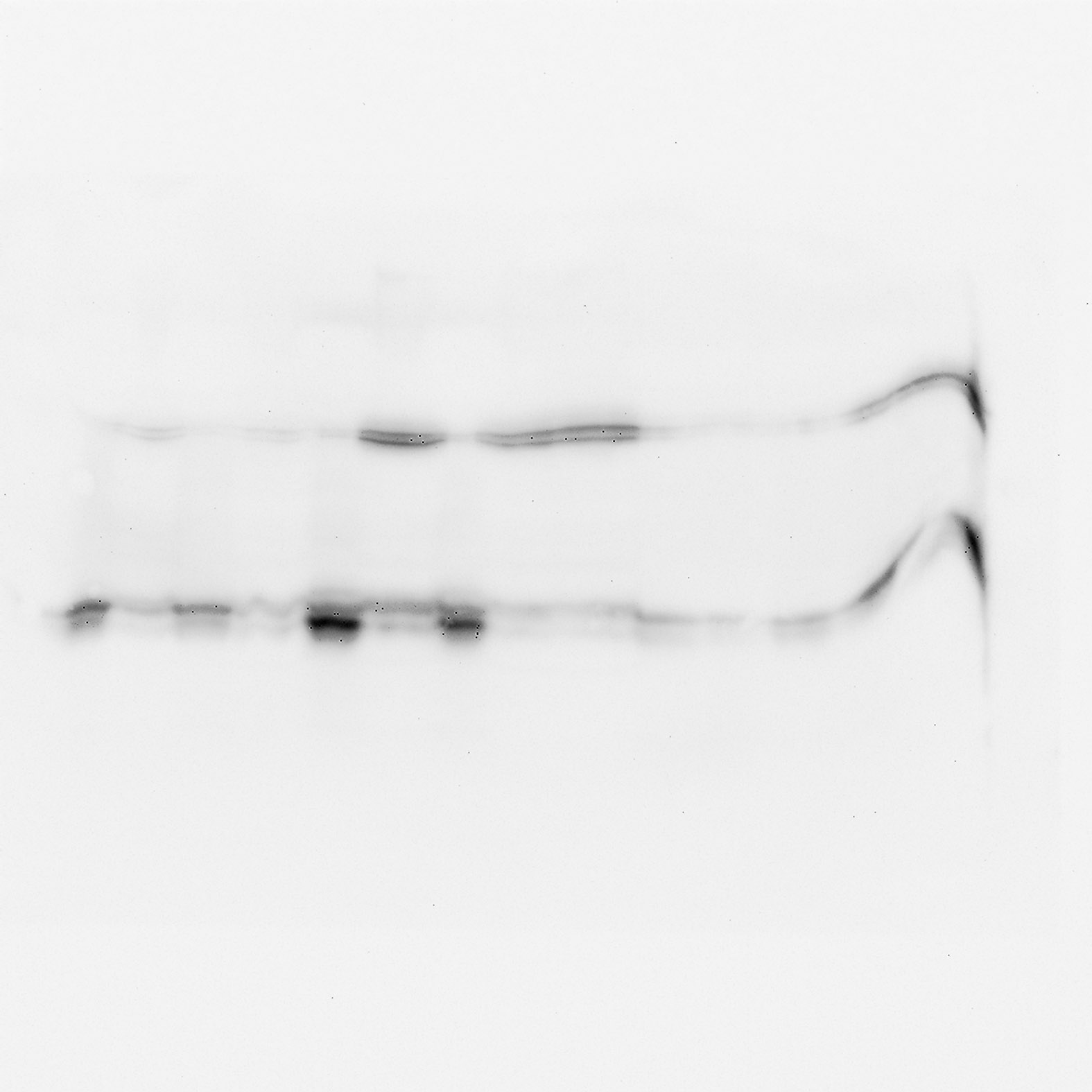

Supplement: Figure 3—source data 1. — A–D contain uncropped western blots shown in Figure 3A–D. Prizm files contain all raw data and statistical analysis to quantify serum-dependent Shh release. B’–E contain uncropped western blots used for the quantification. D` quantifies truncated (proteolytically processed) solubilized Shh, E quantifies relative amounts of unprocessed Shh in media. A’–D’’ Excel file containing raw Shh RP-HPLC elution data as shown in Figure 3A’’–D’’. [file elife-86920-fig3-data1.zip › Figure_3_Source_Data_1 /B'-E_quantification/V744_serum_005%serum.jpg]

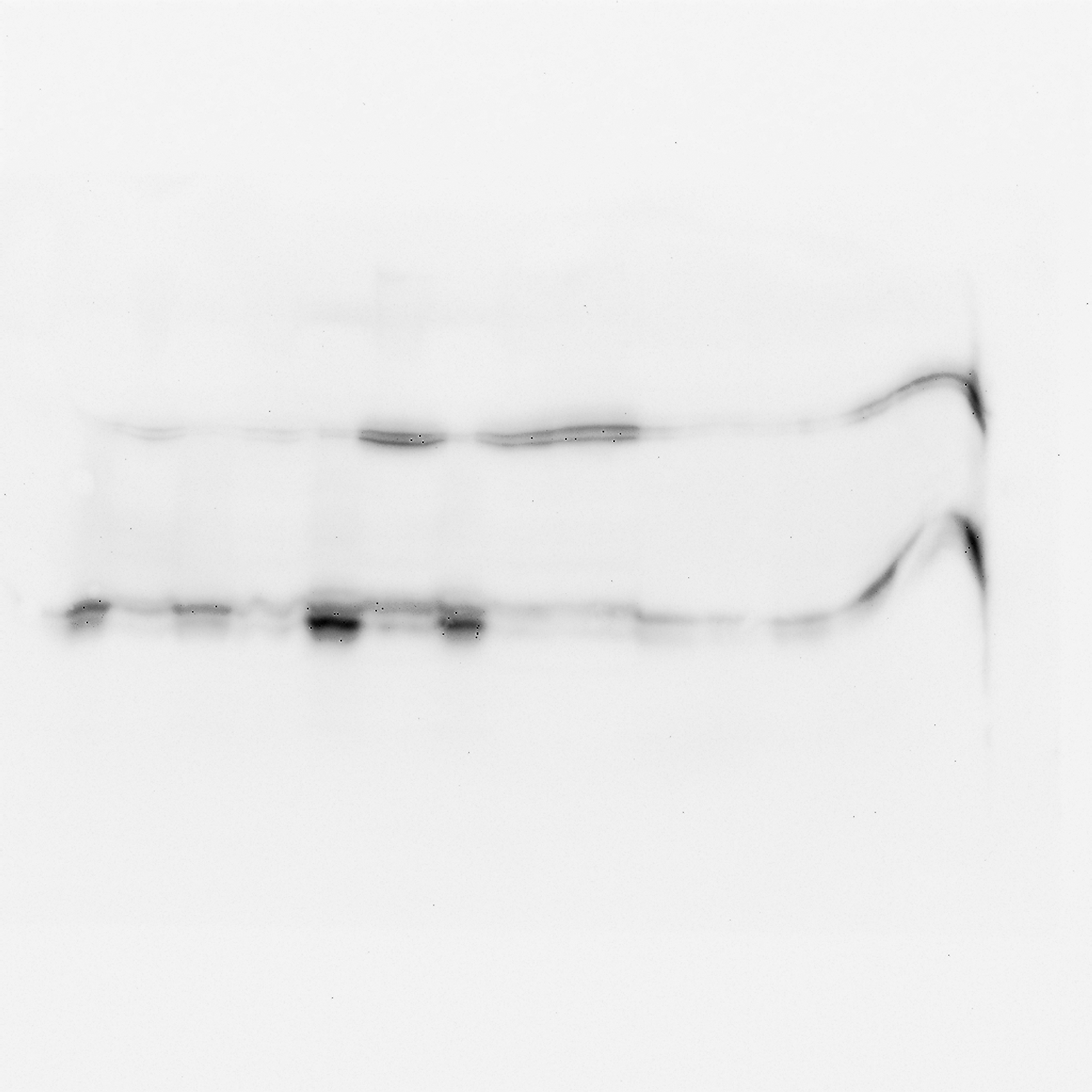

Supplement: Figure 3—source data 1. — A–D contain uncropped western blots shown in Figure 3A–D. Prizm files contain all raw data and statistical analysis to quantify serum-dependent Shh release. B’–E contain uncropped western blots used for the quantification. D` quantifies truncated (proteolytically processed) solubilized Shh, E quantifies relative amounts of unprocessed Shh in media. A’–D’’ Excel file containing raw Shh RP-HPLC elution data as shown in Figure 3A’’–D’’. [file elife-86920-fig3-data1.zip › Figure_3_Source_Data_1 /B'-E_quantification/V744_serum_005%serum.Tif]

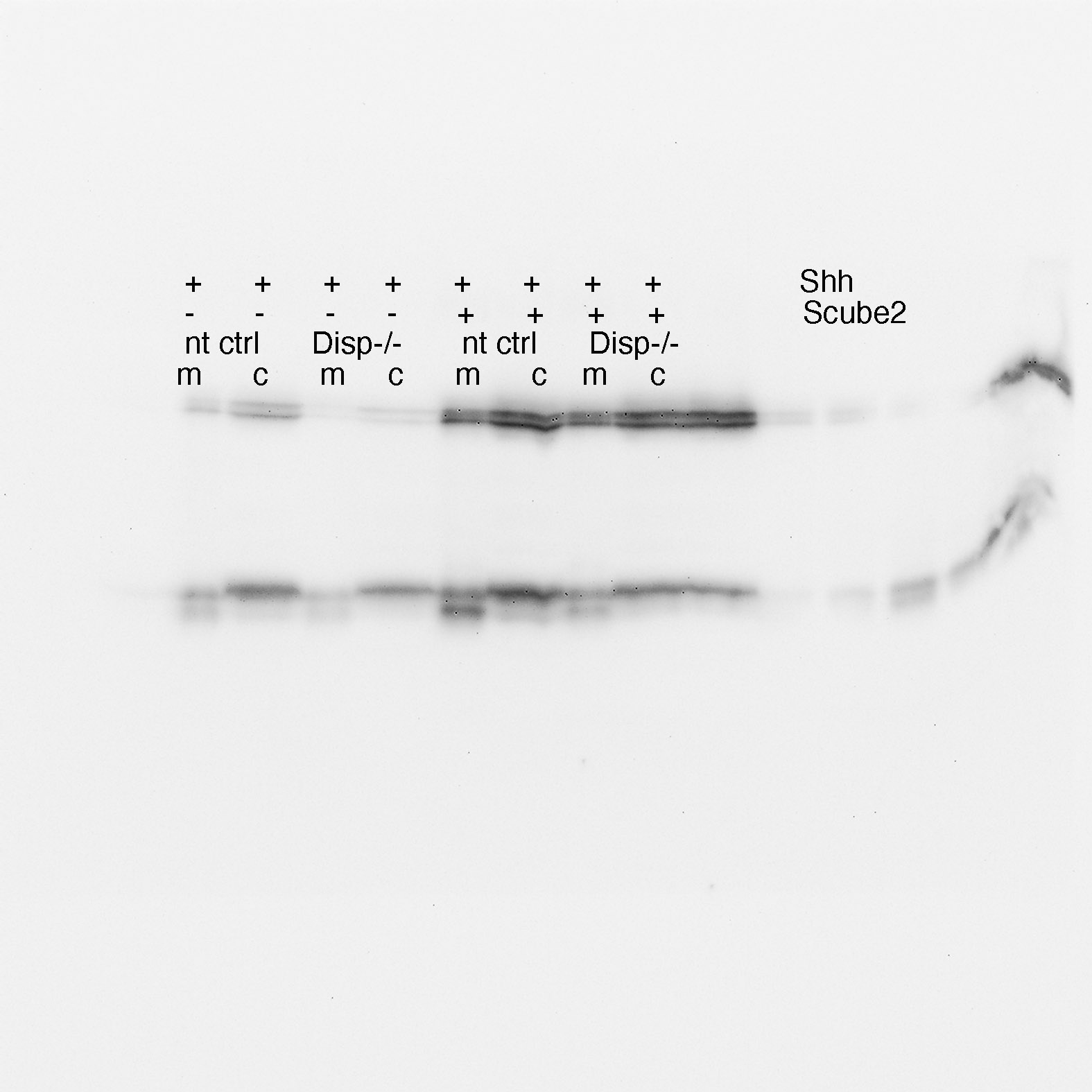

Supplement: Figure 3—source data 1. — A–D contain uncropped western blots shown in Figure 3A–D. Prizm files contain all raw data and statistical analysis to quantify serum-dependent Shh release. B’–E contain uncropped western blots used for the quantification. D` quantifies truncated (proteolytically processed) solubilized Shh, E quantifies relative amounts of unprocessed Shh in media. A’–D’’ Excel file containing raw Shh RP-HPLC elution data as shown in Figure 3A’’–D’’. [file elife-86920-fig3-data1.zip › Figure_3_Source_Data_1 /B'-E_quantification/V744_serum_5%serum labelled.jpg]

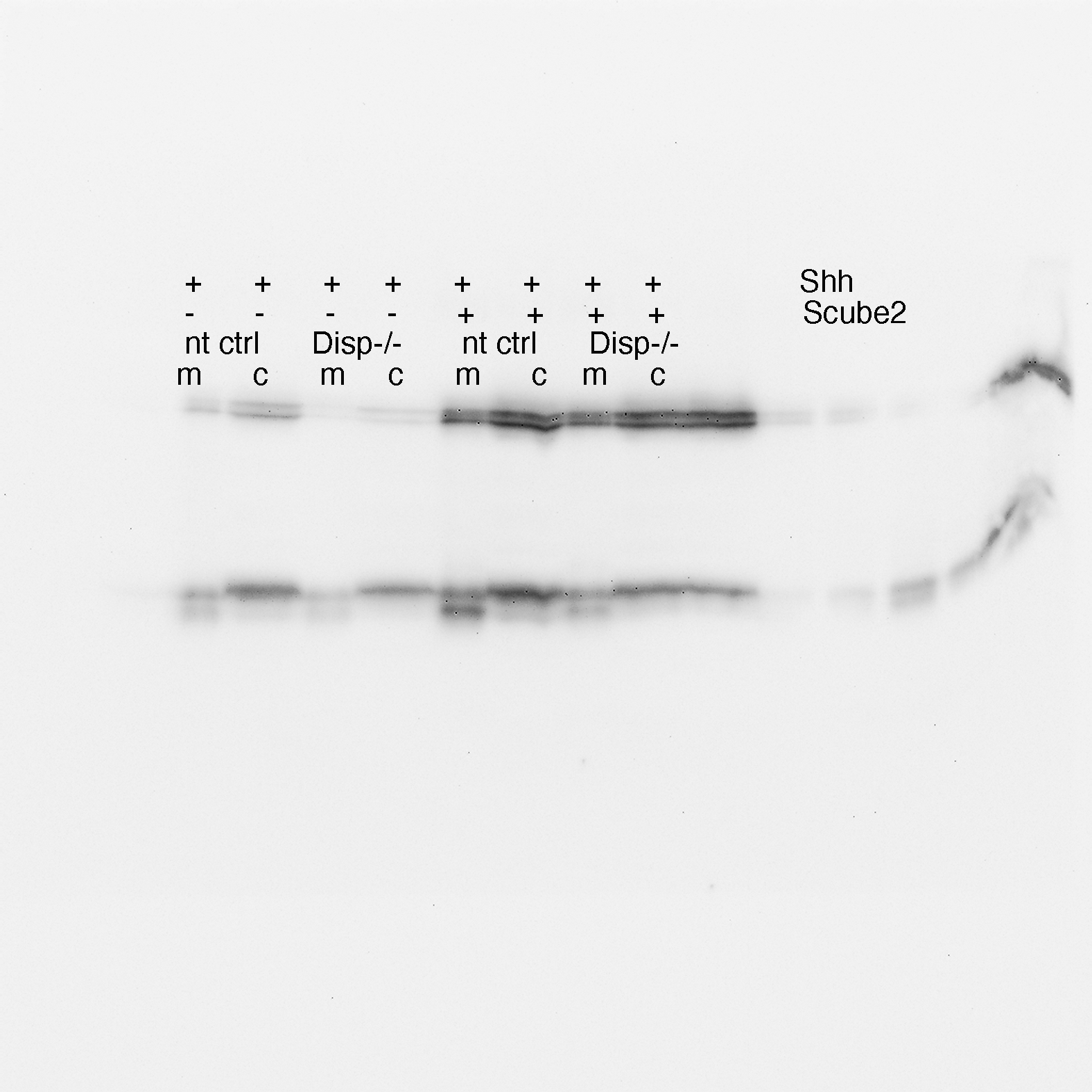

Supplement: Figure 3—source data 1. — A–D contain uncropped western blots shown in Figure 3A–D. Prizm files contain all raw data and statistical analysis to quantify serum-dependent Shh release. B’–E contain uncropped western blots used for the quantification. D` quantifies truncated (proteolytically processed) solubilized Shh, E quantifies relative amounts of unprocessed Shh in media. A’–D’’ Excel file containing raw Shh RP-HPLC elution data as shown in Figure 3A’’–D’’. [file elife-86920-fig3-data1.zip › Figure_3_Source_Data_1 /B'-E_quantification/V744_serum_5%serum labelled.Tif]

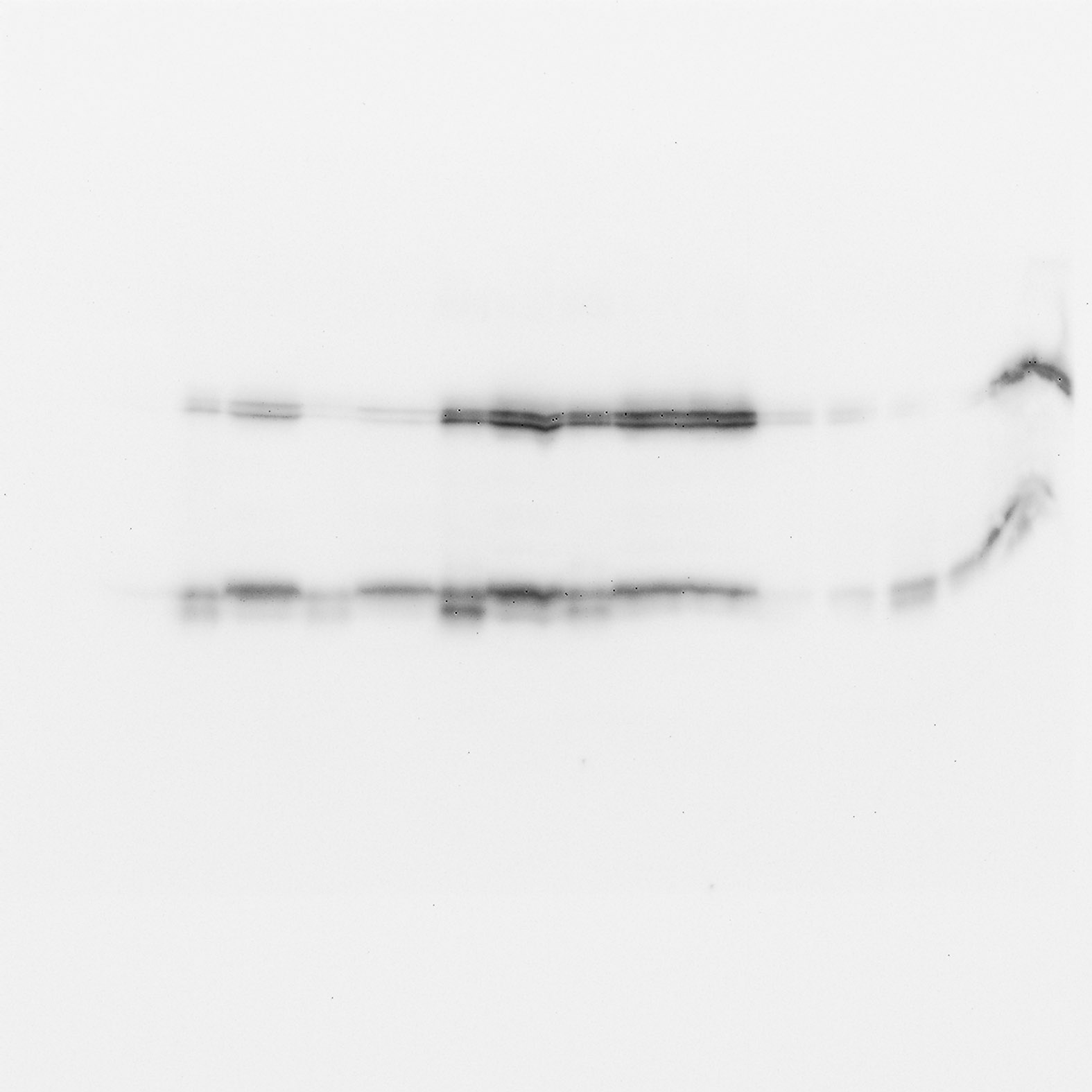

Supplement: Figure 3—source data 1. — A–D contain uncropped western blots shown in Figure 3A–D. Prizm files contain all raw data and statistical analysis to quantify serum-dependent Shh release. B’–E contain uncropped western blots used for the quantification. D` quantifies truncated (proteolytically processed) solubilized Shh, E quantifies relative amounts of unprocessed Shh in media. A’–D’’ Excel file containing raw Shh RP-HPLC elution data as shown in Figure 3A’’–D’’. [file elife-86920-fig3-data1.zip › Figure_3_Source_Data_1 /B'-E_quantification/V744_serum_5%serum.jpg]

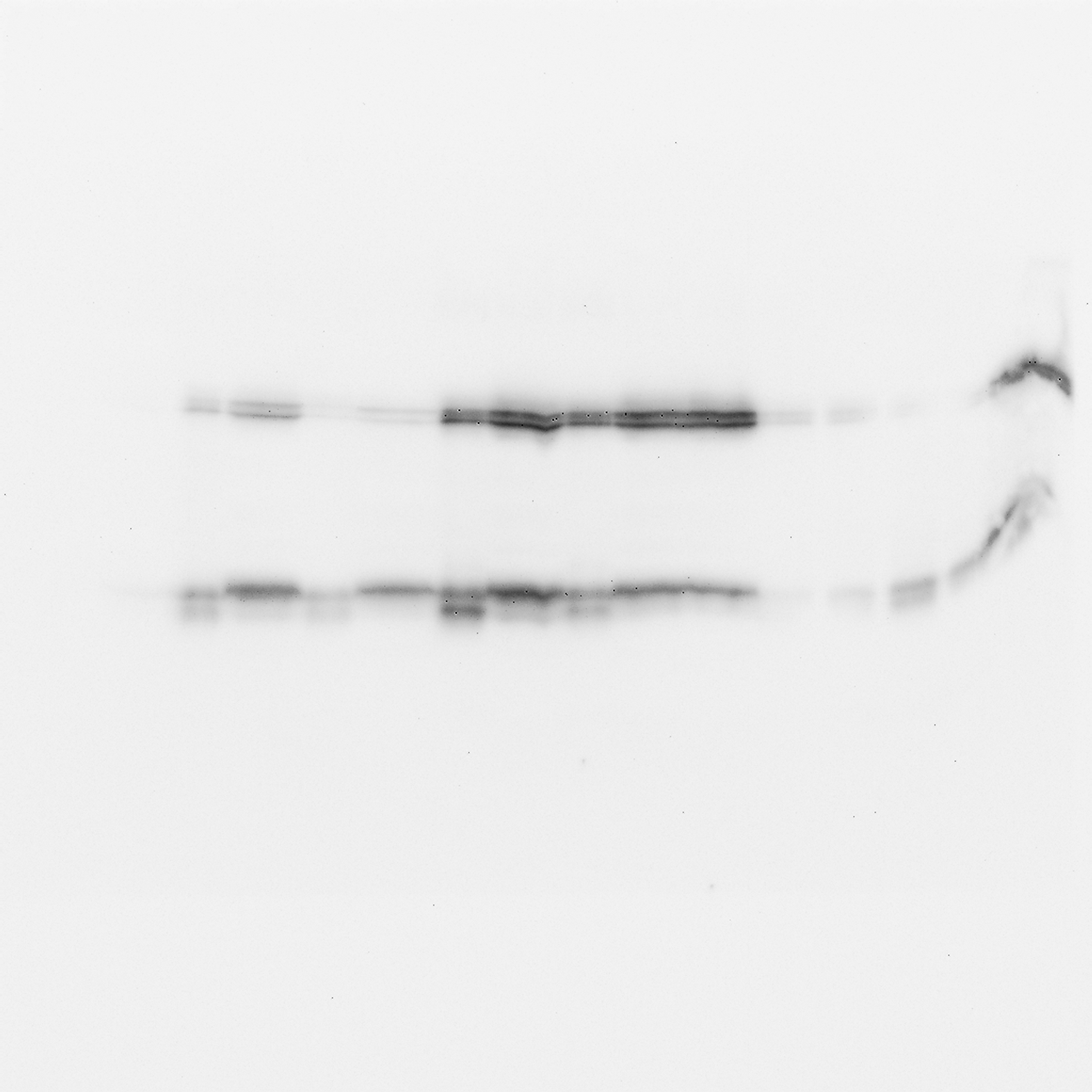

Supplement: Figure 3—source data 1. — A–D contain uncropped western blots shown in Figure 3A–D. Prizm files contain all raw data and statistical analysis to quantify serum-dependent Shh release. B’–E contain uncropped western blots used for the quantification. D` quantifies truncated (proteolytically processed) solubilized Shh, E quantifies relative amounts of unprocessed Shh in media. A’–D’’ Excel file containing raw Shh RP-HPLC elution data as shown in Figure 3A’’–D’’. [file elife-86920-fig3-data1.zip › Figure_3_Source_Data_1 /B'-E_quantification/V744_serum_5%serum.Tif]

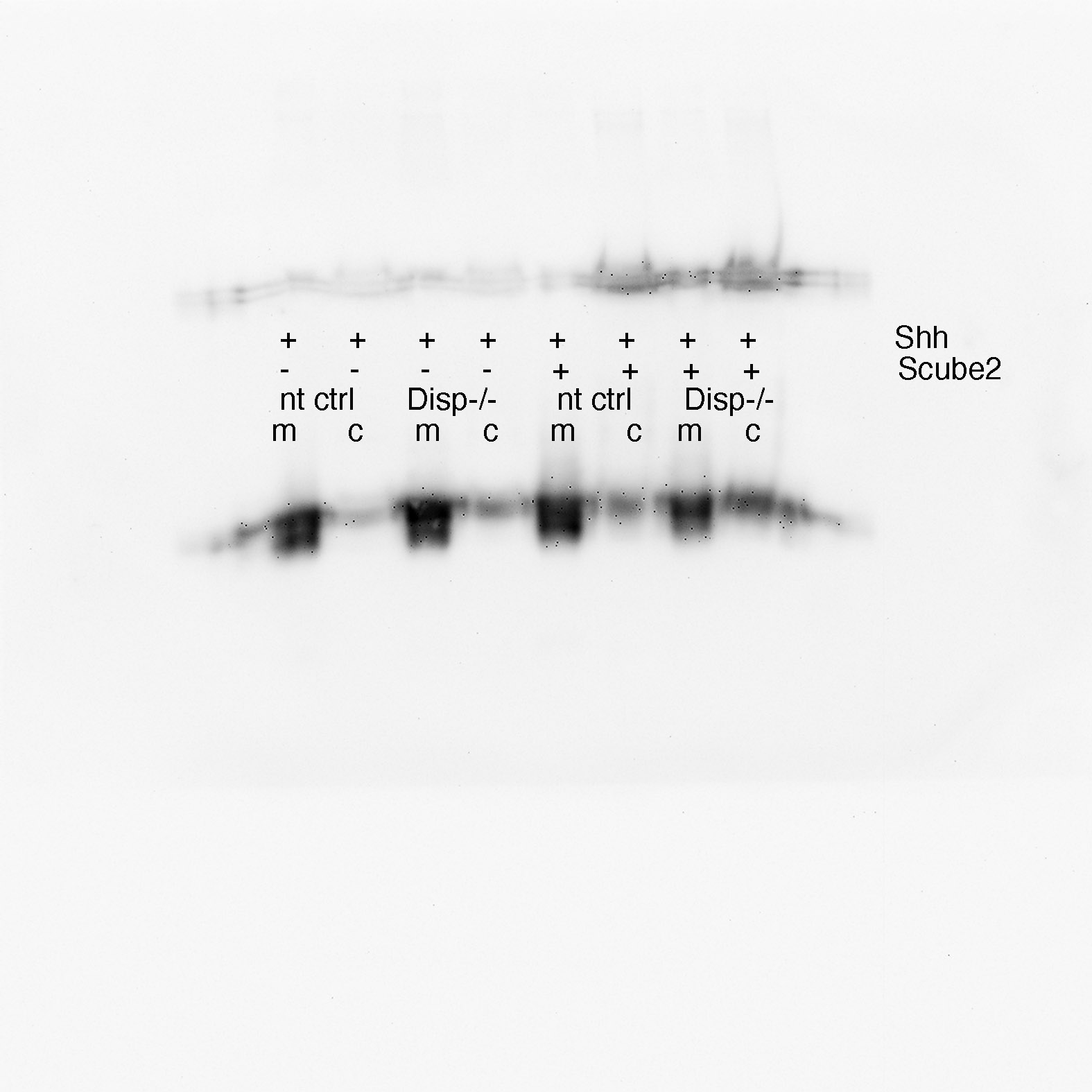

Supplement: Figure 3—source data 1. — A–D contain uncropped western blots shown in Figure 3A–D. Prizm files contain all raw data and statistical analysis to quantify serum-dependent Shh release. B’–E contain uncropped western blots used for the quantification. D` quantifies truncated (proteolytically processed) solubilized Shh, E quantifies relative amounts of unprocessed Shh in media. A’–D’’ Excel file containing raw Shh RP-HPLC elution data as shown in Figure 3A’’–D’’. [file elife-86920-fig3-data1.zip › Figure_3_Source_Data_1 /B'-E_quantification/V789_10%serum labelled.jpg]

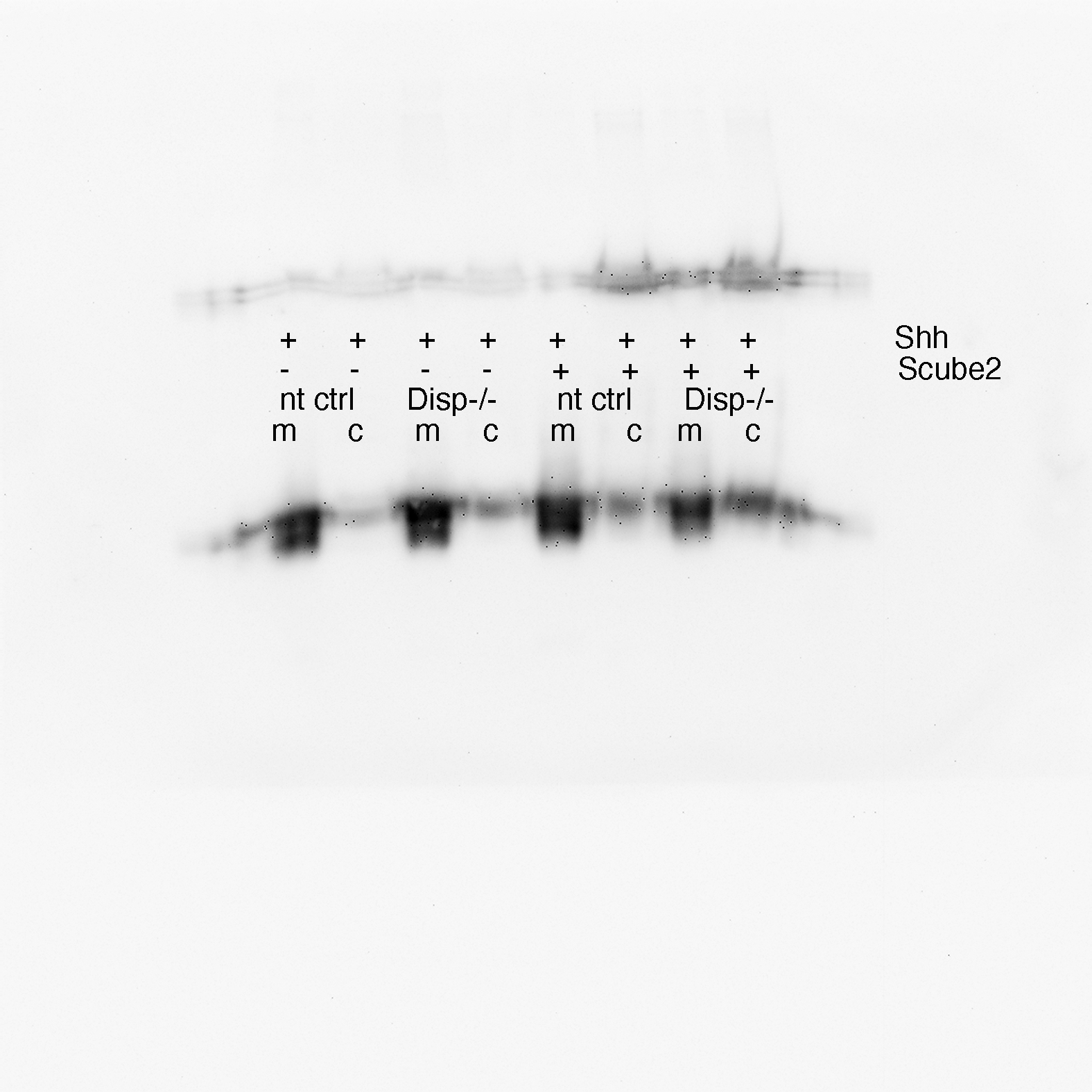

Supplement: Figure 3—source data 1. — A–D contain uncropped western blots shown in Figure 3A–D. Prizm files contain all raw data and statistical analysis to quantify serum-dependent Shh release. B’–E contain uncropped western blots used for the quantification. D` quantifies truncated (proteolytically processed) solubilized Shh, E quantifies relative amounts of unprocessed Shh in media. A’–D’’ Excel file containing raw Shh RP-HPLC elution data as shown in Figure 3A’’–D’’. [file elife-86920-fig3-data1.zip › Figure_3_Source_Data_1 /B'-E_quantification/V789_10%serum labelled.Tif]

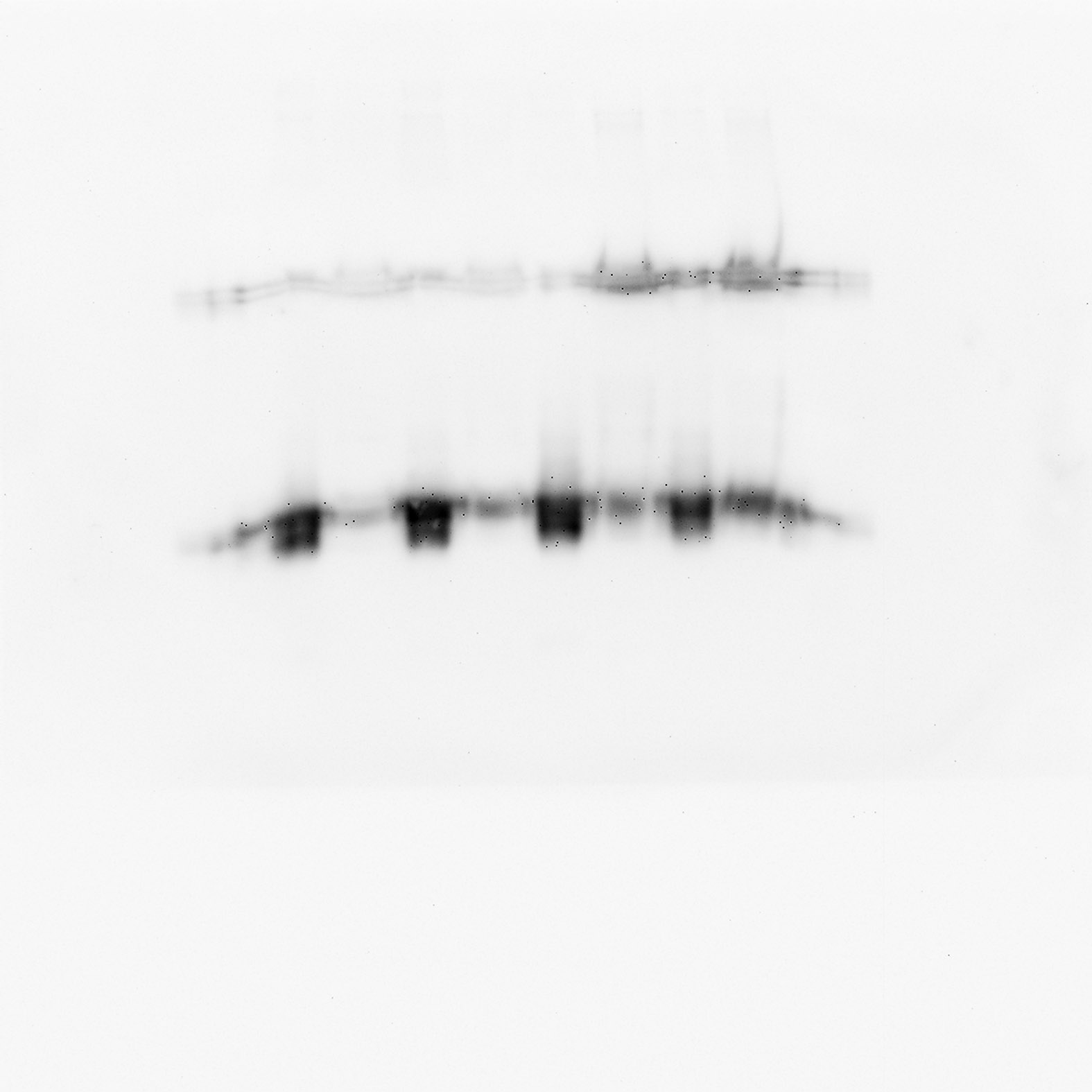

Supplement: Figure 3—source data 1. — A–D contain uncropped western blots shown in Figure 3A–D. Prizm files contain all raw data and statistical analysis to quantify serum-dependent Shh release. B’–E contain uncropped western blots used for the quantification. D` quantifies truncated (proteolytically processed) solubilized Shh, E quantifies relative amounts of unprocessed Shh in media. A’–D’’ Excel file containing raw Shh RP-HPLC elution data as shown in Figure 3A’’–D’’. [file elife-86920-fig3-data1.zip › Figure_3_Source_Data_1 /B'-E_quantification/V789_10%serum.jpg]

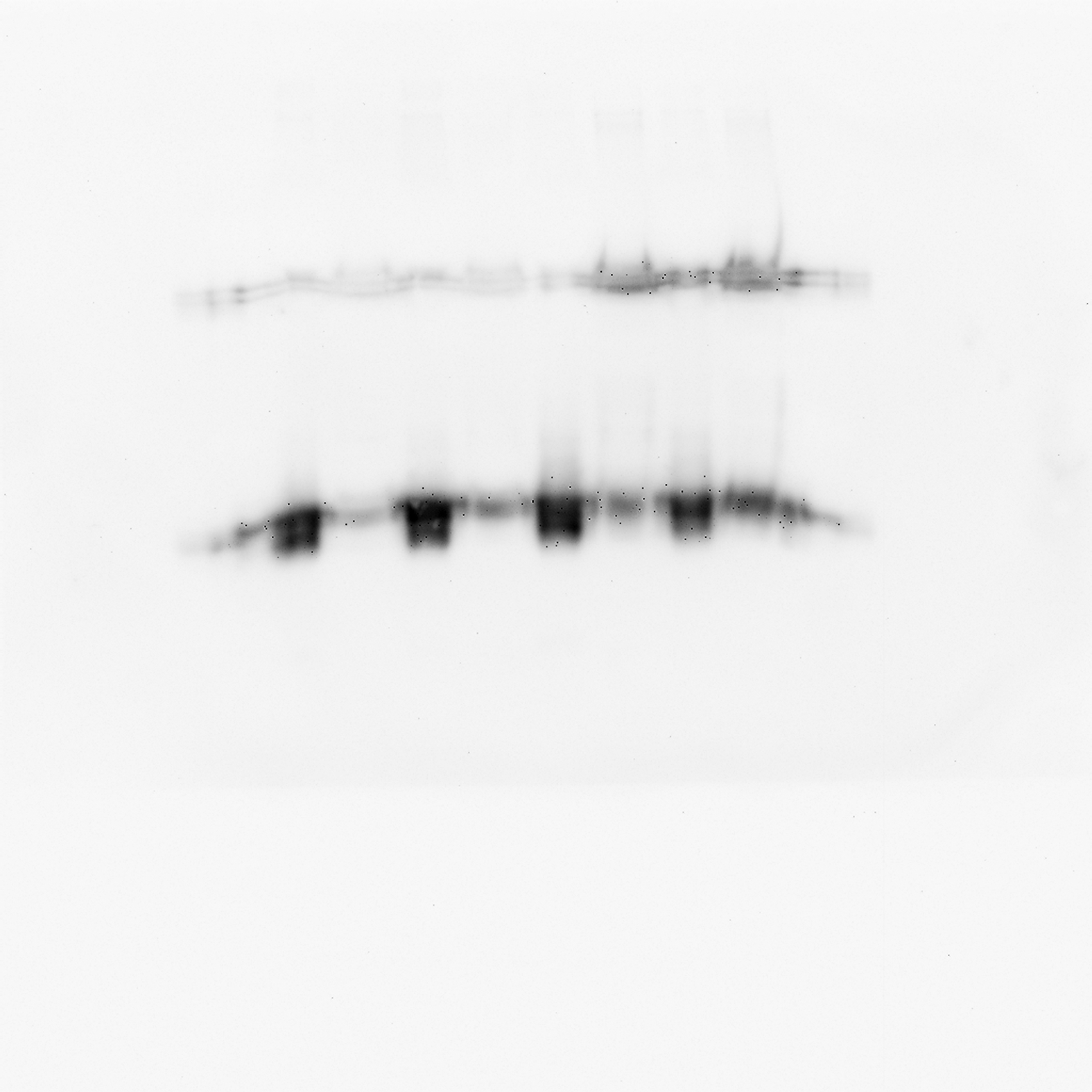

Supplement: Figure 3—source data 1. — A–D contain uncropped western blots shown in Figure 3A–D. Prizm files contain all raw data and statistical analysis to quantify serum-dependent Shh release. B’–E contain uncropped western blots used for the quantification. D` quantifies truncated (proteolytically processed) solubilized Shh, E quantifies relative amounts of unprocessed Shh in media. A’–D’’ Excel file containing raw Shh RP-HPLC elution data as shown in Figure 3A’’–D’’. [file elife-86920-fig3-data1.zip › Figure_3_Source_Data_1 /B'-E_quantification/V789_10%serum.Tif]

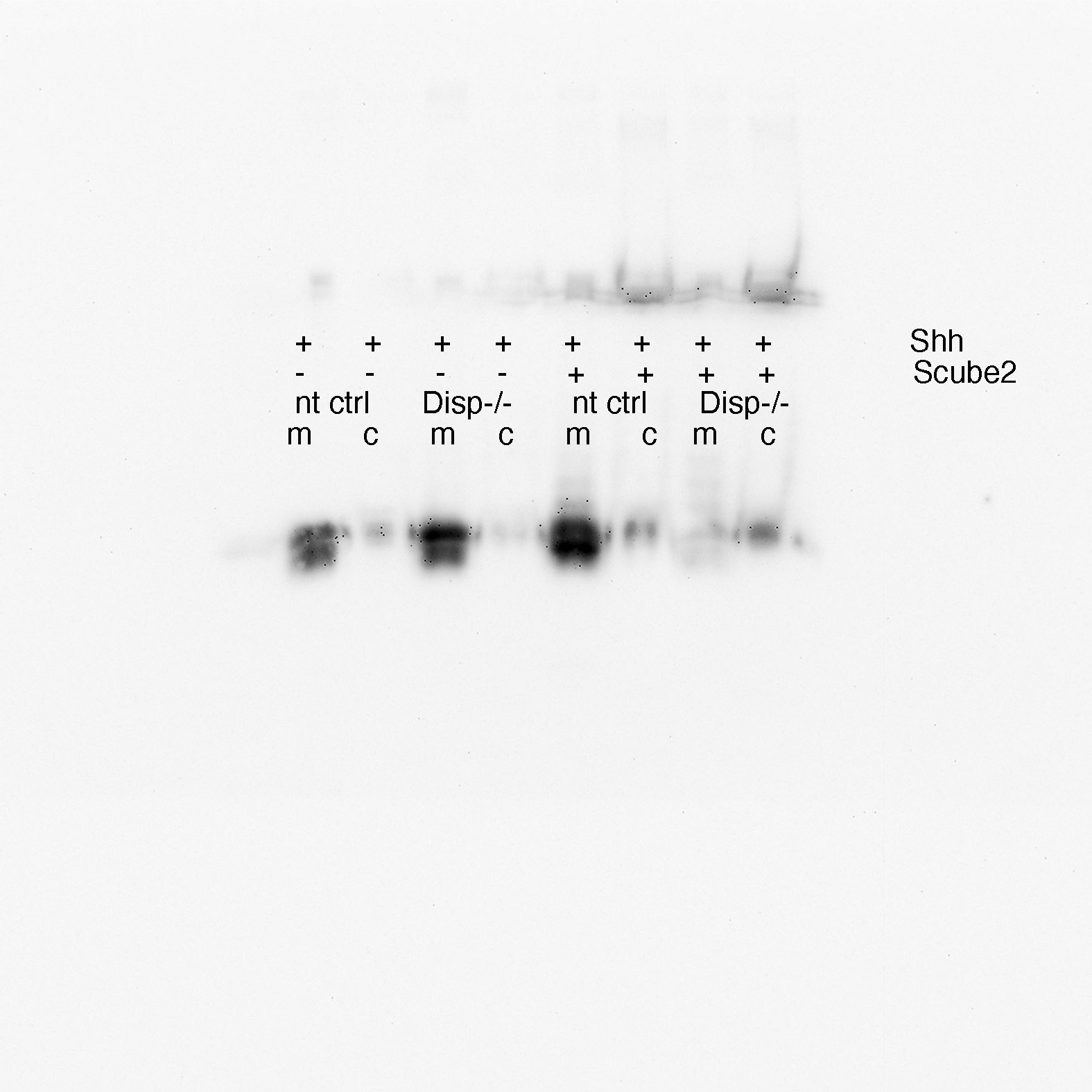

Supplement: Figure 3—source data 1. — A–D contain uncropped western blots shown in Figure 3A–D. Prizm files contain all raw data and statistical analysis to quantify serum-dependent Shh release. B’–E contain uncropped western blots used for the quantification. D` quantifies truncated (proteolytically processed) solubilized Shh, E quantifies relative amounts of unprocessed Shh in media. A’–D’’ Excel file containing raw Shh RP-HPLC elution data as shown in Figure 3A’’–D’’. [file elife-86920-fig3-data1.zip › Figure_3_Source_Data_1 /B'-E_quantification/V789_10%serum_2 labelled.jpg]

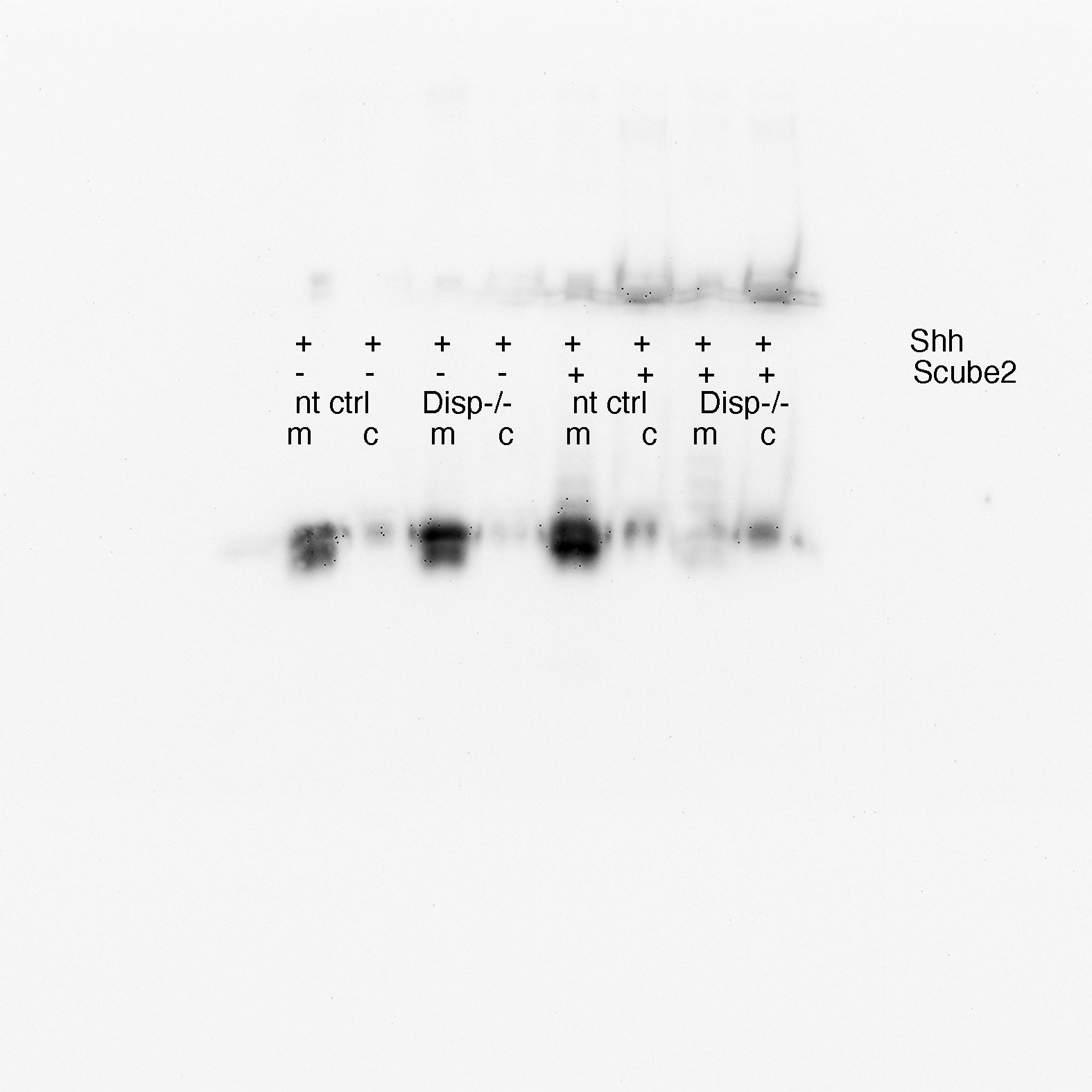

Supplement: Figure 3—source data 1. — A–D contain uncropped western blots shown in Figure 3A–D. Prizm files contain all raw data and statistical analysis to quantify serum-dependent Shh release. B’–E contain uncropped western blots used for the quantification. D` quantifies truncated (proteolytically processed) solubilized Shh, E quantifies relative amounts of unprocessed Shh in media. A’–D’’ Excel file containing raw Shh RP-HPLC elution data as shown in Figure 3A’’–D’’. [file elife-86920-fig3-data1.zip › Figure_3_Source_Data_1 /B'-E_quantification/V789_10%serum_2 labelled.Tif]

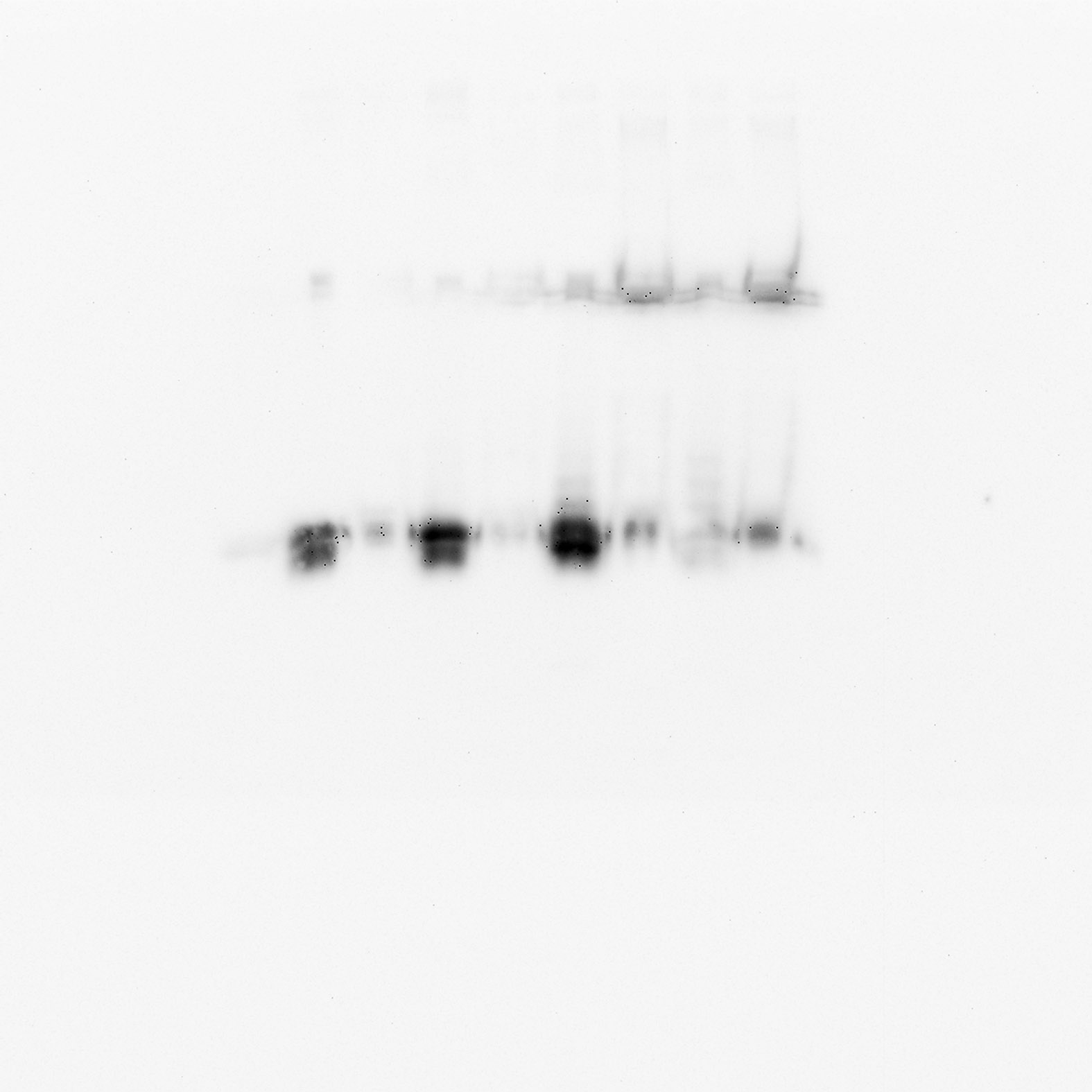

Supplement: Figure 3—source data 1. — A–D contain uncropped western blots shown in Figure 3A–D. Prizm files contain all raw data and statistical analysis to quantify serum-dependent Shh release. B’–E contain uncropped western blots used for the quantification. D` quantifies truncated (proteolytically processed) solubilized Shh, E quantifies relative amounts of unprocessed Shh in media. A’–D’’ Excel file containing raw Shh RP-HPLC elution data as shown in Figure 3A’’–D’’. [file elife-86920-fig3-data1.zip › Figure_3_Source_Data_1 /B'-E_quantification/V789_10%serum_2.jpg]

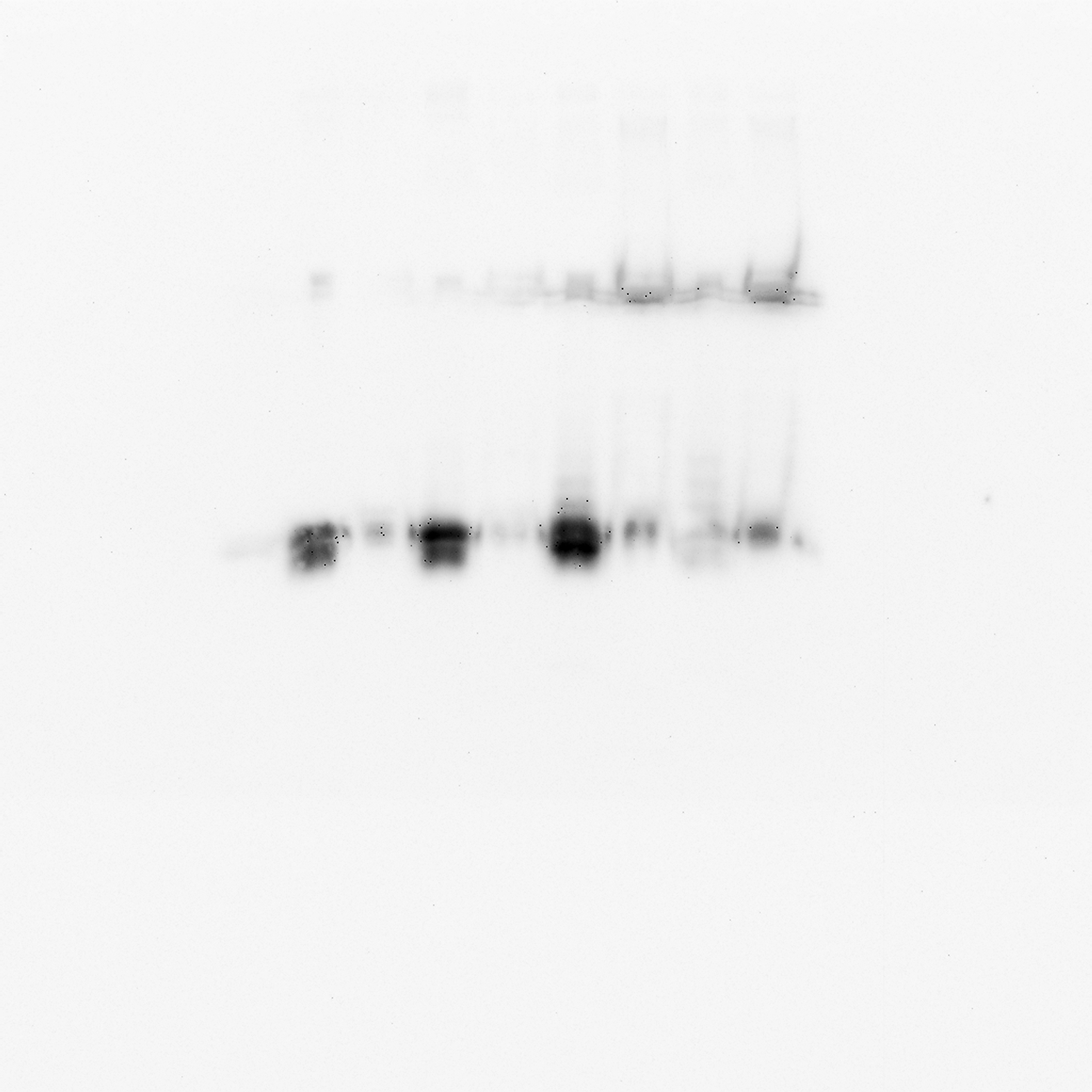

Supplement: Figure 3—source data 1. — A–D contain uncropped western blots shown in Figure 3A–D. Prizm files contain all raw data and statistical analysis to quantify serum-dependent Shh release. B’–E contain uncropped western blots used for the quantification. D` quantifies truncated (proteolytically processed) solubilized Shh, E quantifies relative amounts of unprocessed Shh in media. A’–D’’ Excel file containing raw Shh RP-HPLC elution data as shown in Figure 3A’’–D’’. [file elife-86920-fig3-data1.zip › Figure_3_Source_Data_1 /B'-E_quantification/V789_10%serum_2.Tif]

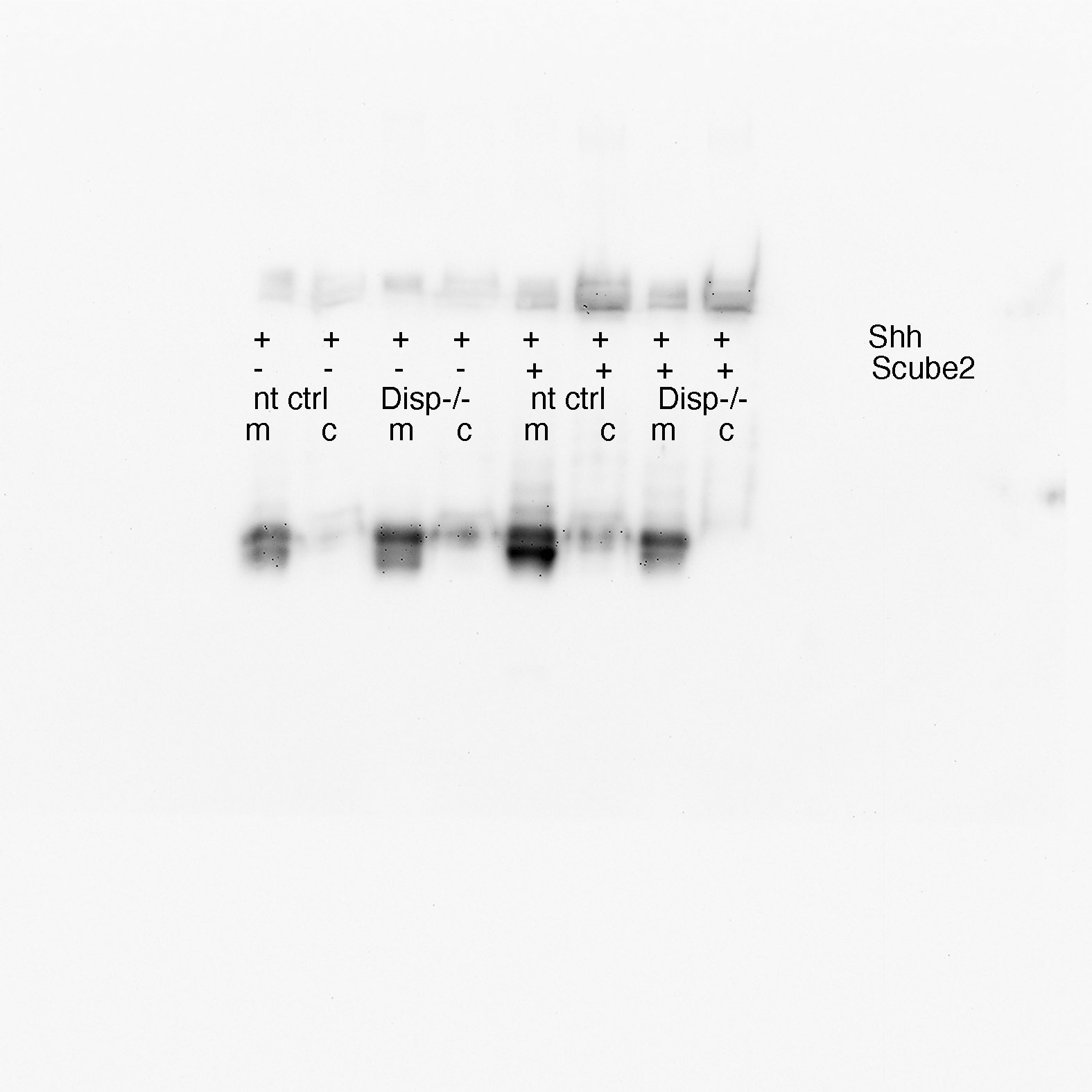

Supplement: Figure 3—source data 1. — A–D contain uncropped western blots shown in Figure 3A–D. Prizm files contain all raw data and statistical analysis to quantify serum-dependent Shh release. B’–E contain uncropped western blots used for the quantification. D` quantifies truncated (proteolytically processed) solubilized Shh, E quantifies relative amounts of unprocessed Shh in media. A’–D’’ Excel file containing raw Shh RP-HPLC elution data as shown in Figure 3A’’–D’’. [file elife-86920-fig3-data1.zip › Figure_3_Source_Data_1 /B'-E_quantification/V789_5%serum labelled.jpg]

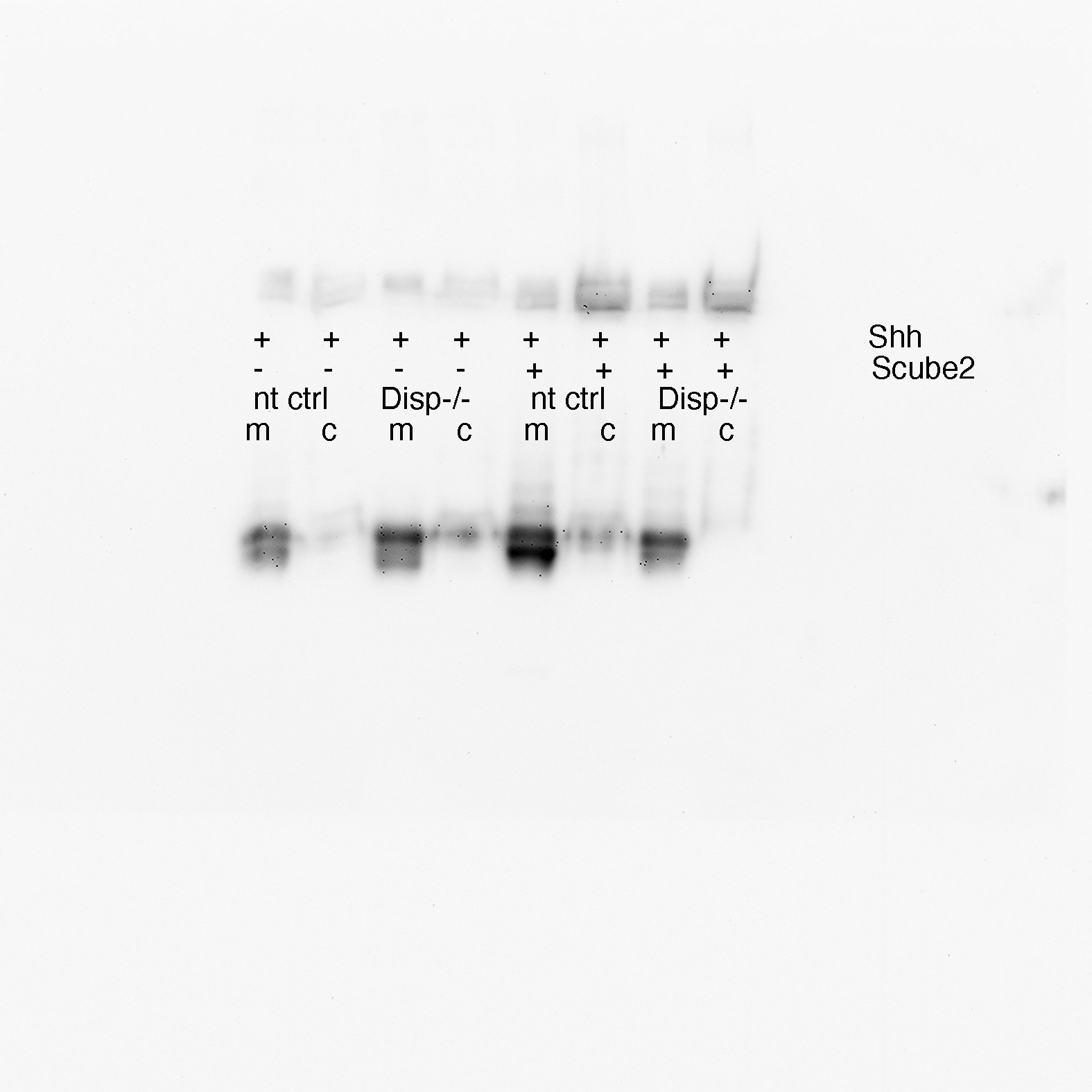

Supplement: Figure 3—source data 1. — A–D contain uncropped western blots shown in Figure 3A–D. Prizm files contain all raw data and statistical analysis to quantify serum-dependent Shh release. B’–E contain uncropped western blots used for the quantification. D` quantifies truncated (proteolytically processed) solubilized Shh, E quantifies relative amounts of unprocessed Shh in media. A’–D’’ Excel file containing raw Shh RP-HPLC elution data as shown in Figure 3A’’–D’’. [file elife-86920-fig3-data1.zip › Figure_3_Source_Data_1 /B'-E_quantification/V789_5%serum labelled.Tif]

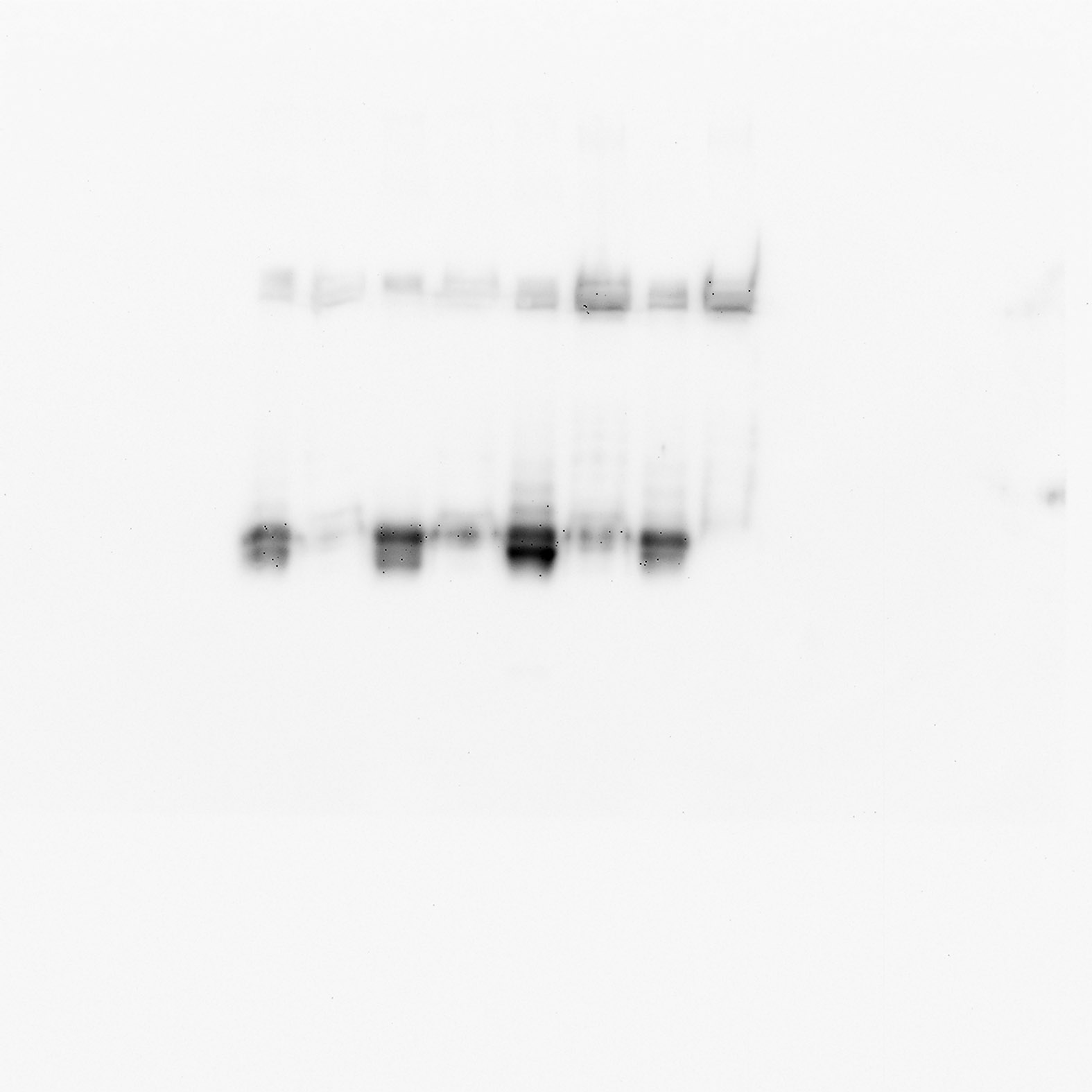

Supplement: Figure 3—source data 1. — A–D contain uncropped western blots shown in Figure 3A–D. Prizm files contain all raw data and statistical analysis to quantify serum-dependent Shh release. B’–E contain uncropped western blots used for the quantification. D` quantifies truncated (proteolytically processed) solubilized Shh, E quantifies relative amounts of unprocessed Shh in media. A’–D’’ Excel file containing raw Shh RP-HPLC elution data as shown in Figure 3A’’–D’’. [file elife-86920-fig3-data1.zip › Figure_3_Source_Data_1 /B'-E_quantification/V789_5%serum.jpg]

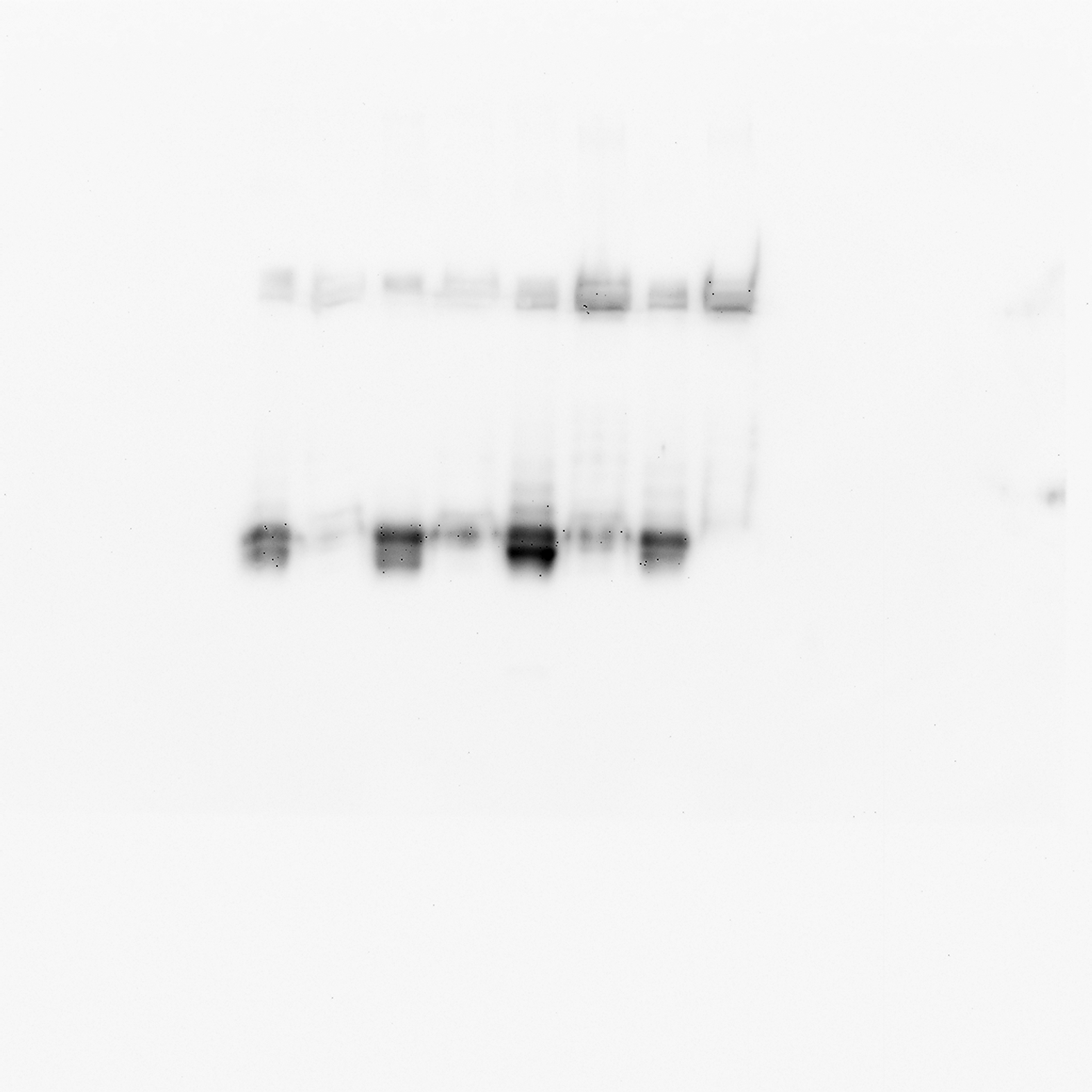

Supplement: Figure 3—source data 1. — A–D contain uncropped western blots shown in Figure 3A–D. Prizm files contain all raw data and statistical analysis to quantify serum-dependent Shh release. B’–E contain uncropped western blots used for the quantification. D` quantifies truncated (proteolytically processed) solubilized Shh, E quantifies relative amounts of unprocessed Shh in media. A’–D’’ Excel file containing raw Shh RP-HPLC elution data as shown in Figure 3A’’–D’’. [file elife-86920-fig3-data1.zip › Figure_3_Source_Data_1 /B'-E_quantification/V789_5%serum.Tif]

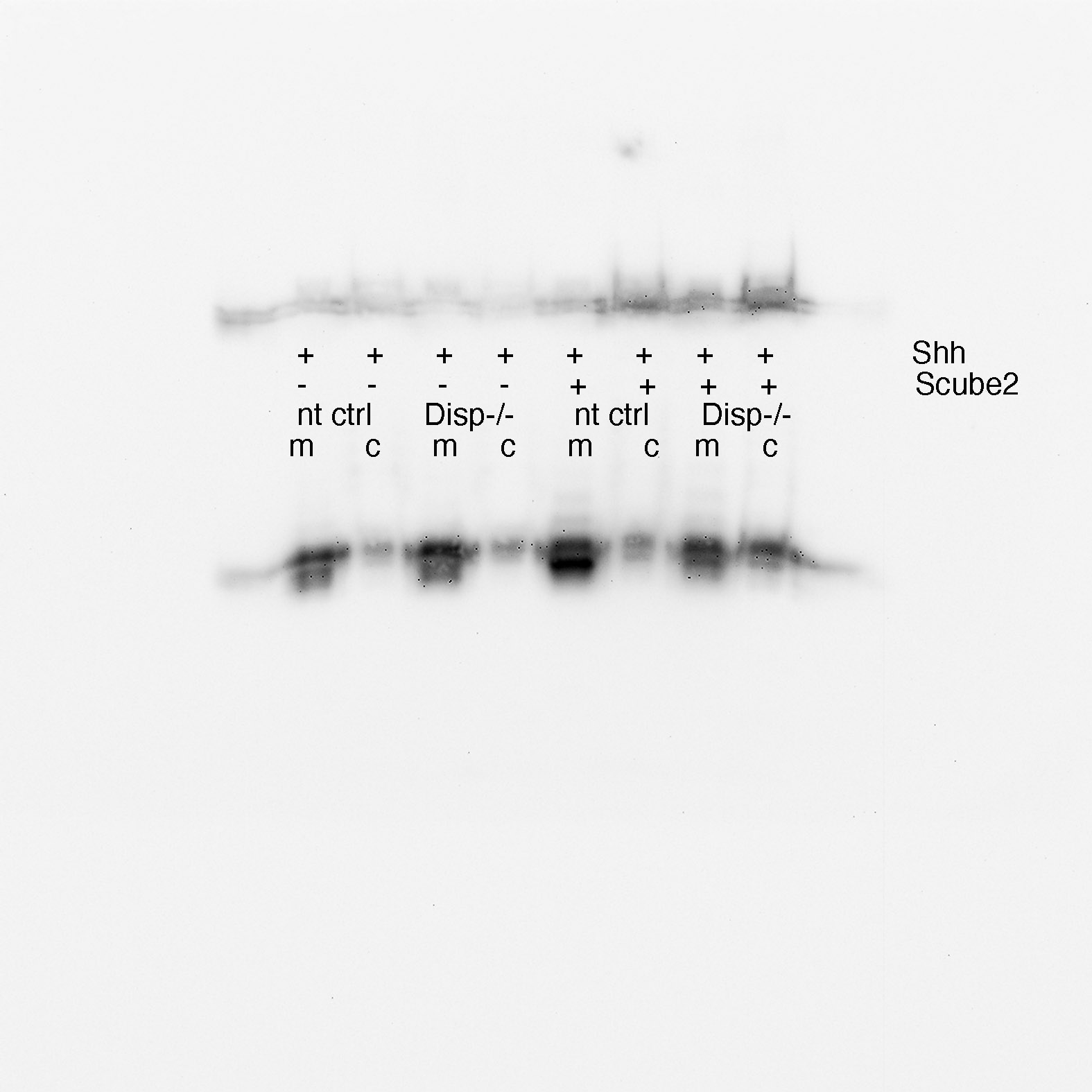

Supplement: Figure 3—source data 1. — A–D contain uncropped western blots shown in Figure 3A–D. Prizm files contain all raw data and statistical analysis to quantify serum-dependent Shh release. B’–E contain uncropped western blots used for the quantification. D` quantifies truncated (proteolytically processed) solubilized Shh, E quantifies relative amounts of unprocessed Shh in media. A’–D’’ Excel file containing raw Shh RP-HPLC elution data as shown in Figure 3A’’–D’’. [file elife-86920-fig3-data1.zip › Figure_3_Source_Data_1 /B'-E_quantification/V789_5%serum_2 labelled.jpg]

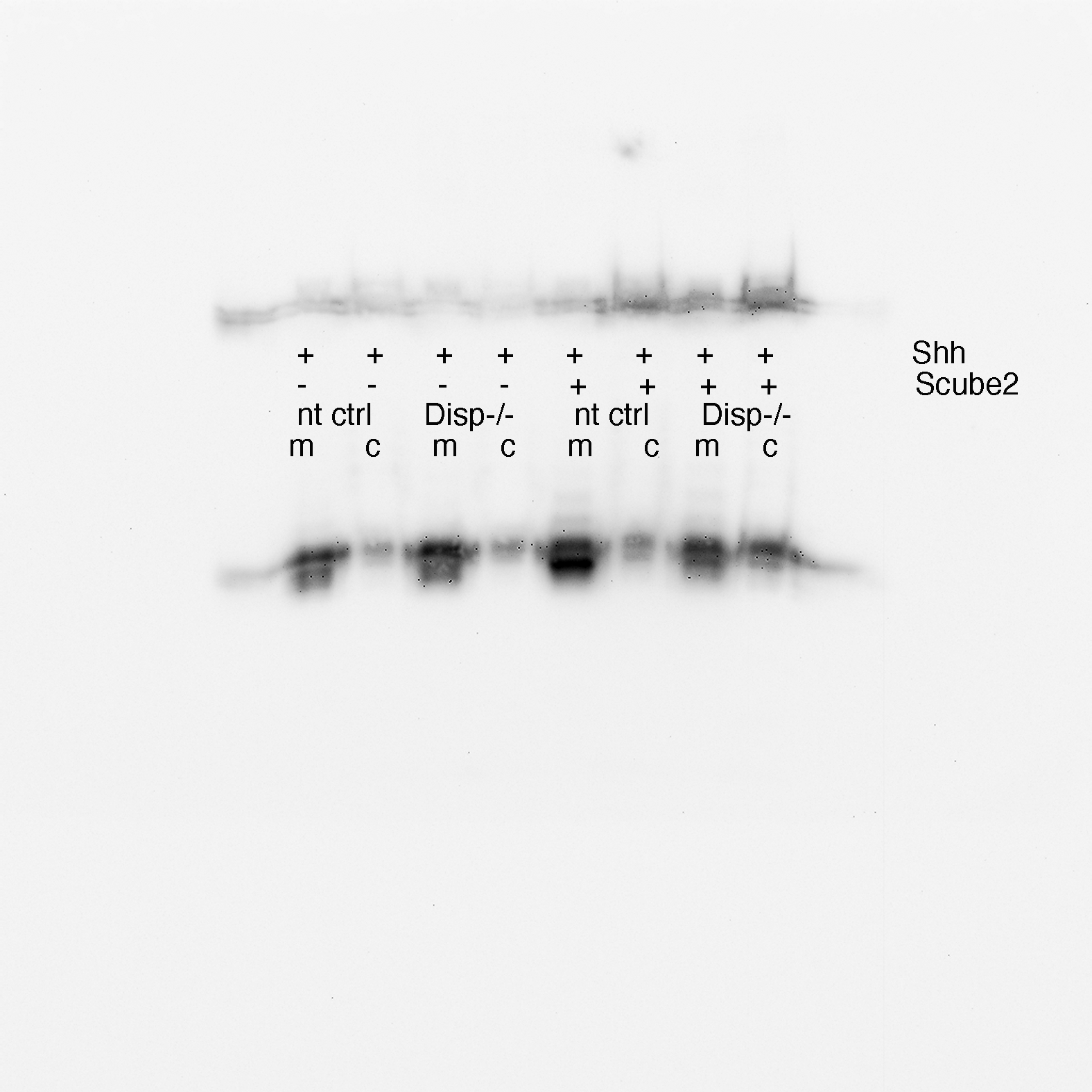

Supplement: Figure 3—source data 1. — A–D contain uncropped western blots shown in Figure 3A–D. Prizm files contain all raw data and statistical analysis to quantify serum-dependent Shh release. B’–E contain uncropped western blots used for the quantification. D` quantifies truncated (proteolytically processed) solubilized Shh, E quantifies relative amounts of unprocessed Shh in media. A’–D’’ Excel file containing raw Shh RP-HPLC elution data as shown in Figure 3A’’–D’’. [file elife-86920-fig3-data1.zip › Figure_3_Source_Data_1 /B'-E_quantification/V789_5%serum_2 labelled.Tif]

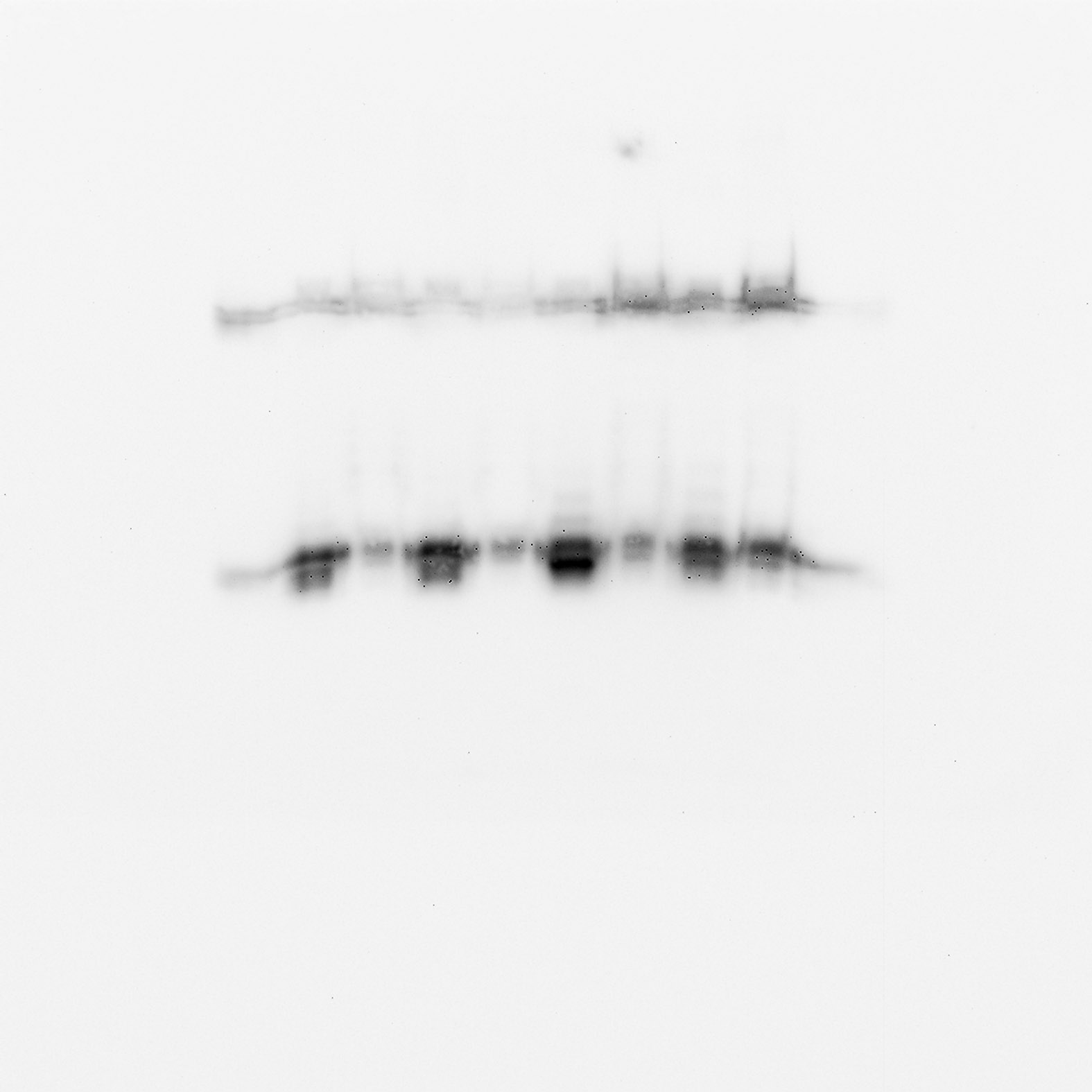

Supplement: Figure 3—source data 1. — A–D contain uncropped western blots shown in Figure 3A–D. Prizm files contain all raw data and statistical analysis to quantify serum-dependent Shh release. B’–E contain uncropped western blots used for the quantification. D` quantifies truncated (proteolytically processed) solubilized Shh, E quantifies relative amounts of unprocessed Shh in media. A’–D’’ Excel file containing raw Shh RP-HPLC elution data as shown in Figure 3A’’–D’’. [file elife-86920-fig3-data1.zip › Figure_3_Source_Data_1 /B'-E_quantification/V789_5%serum_2.jpg]

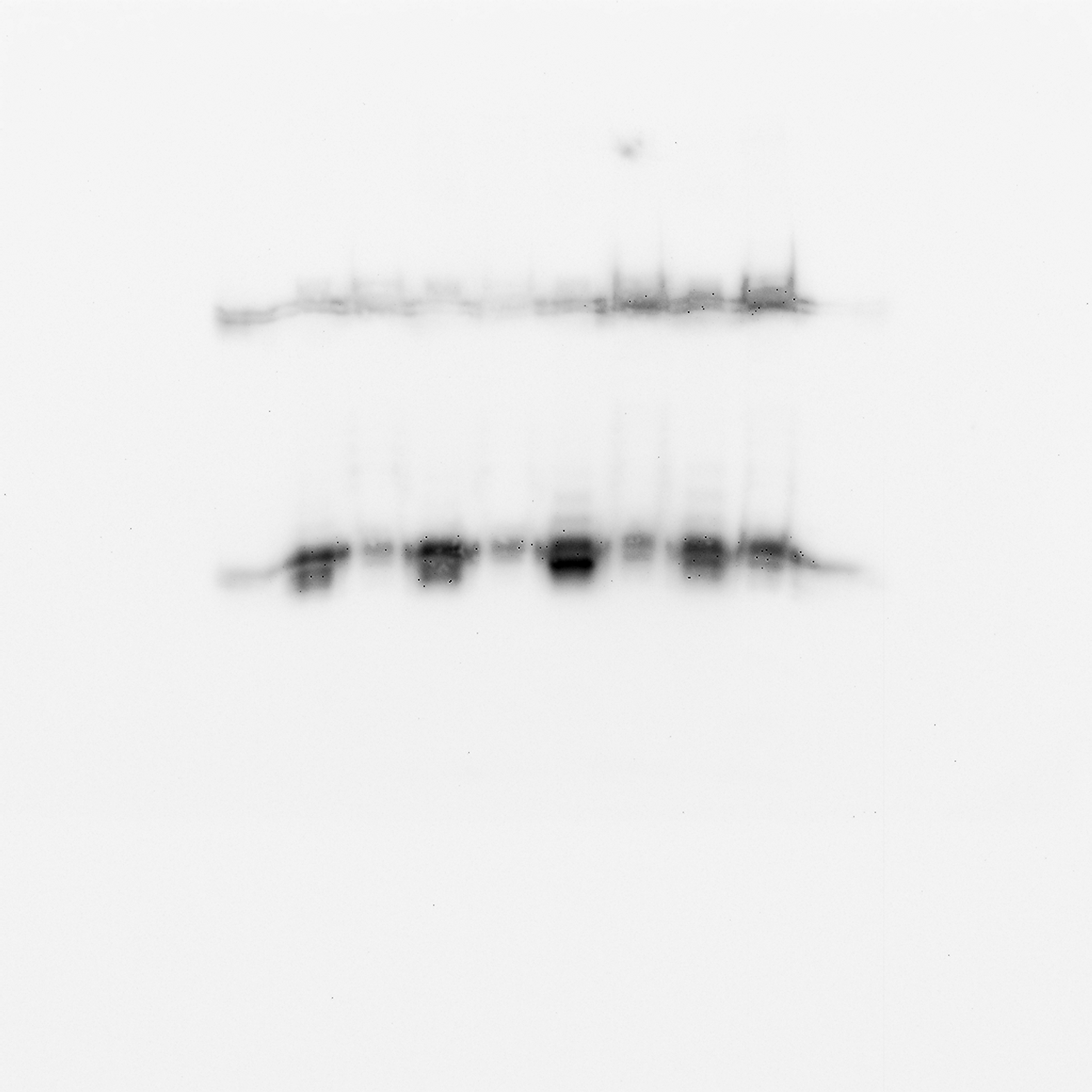

Supplement: Figure 3—source data 1. — A–D contain uncropped western blots shown in Figure 3A–D. Prizm files contain all raw data and statistical analysis to quantify serum-dependent Shh release. B’–E contain uncropped western blots used for the quantification. D` quantifies truncated (proteolytically processed) solubilized Shh, E quantifies relative amounts of unprocessed Shh in media. A’–D’’ Excel file containing raw Shh RP-HPLC elution data as shown in Figure 3A’’–D’’. [file elife-86920-fig3-data1.zip › Figure_3_Source_Data_1 /B'-E_quantification/V789_5%serum_2.Tif]

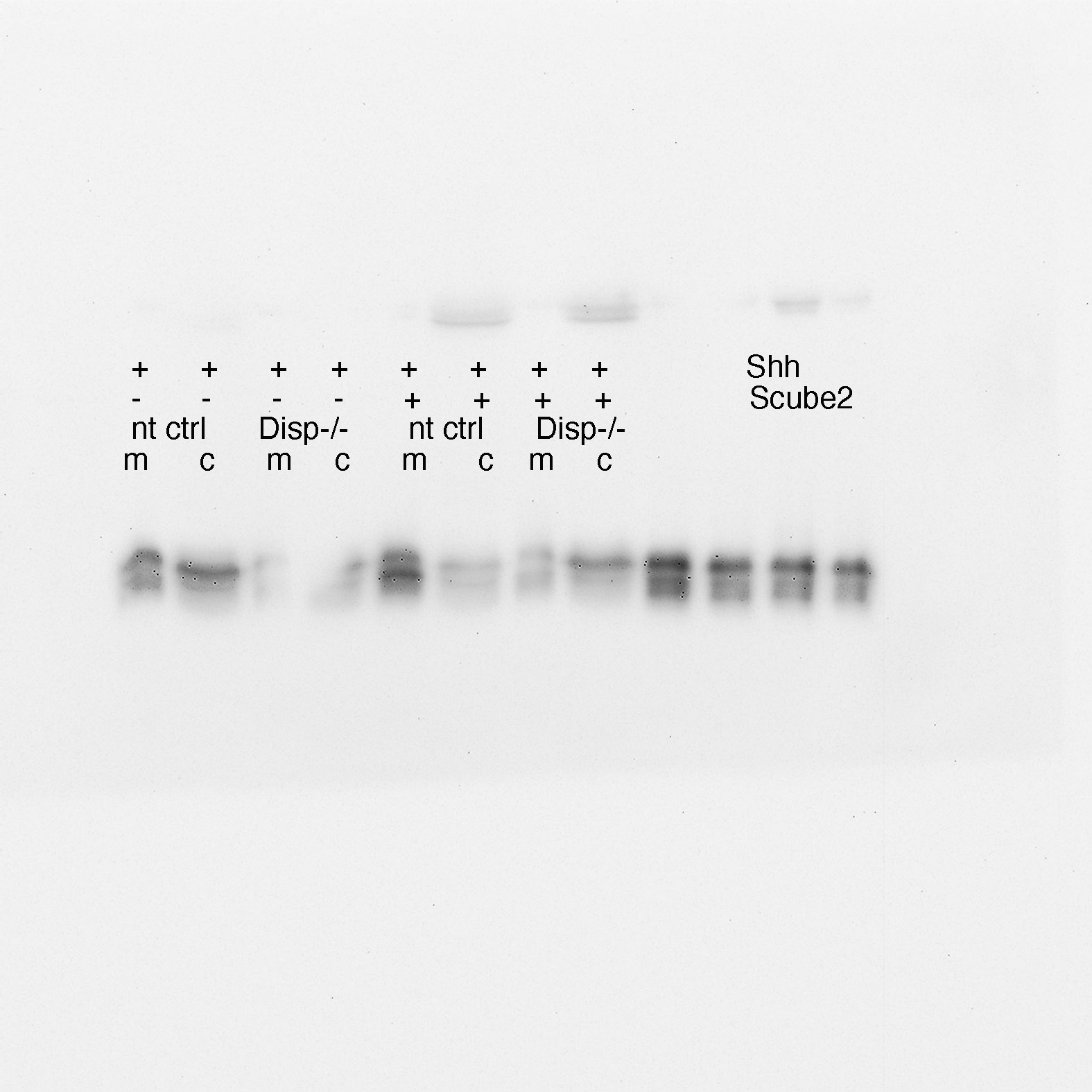

Supplement: Figure 3—source data 1. — A–D contain uncropped western blots shown in Figure 3A–D. Prizm files contain all raw data and statistical analysis to quantify serum-dependent Shh release. B’–E contain uncropped western blots used for the quantification. D` quantifies truncated (proteolytically processed) solubilized Shh, E quantifies relative amounts of unprocessed Shh in media. A’–D’’ Excel file containing raw Shh RP-HPLC elution data as shown in Figure 3A’’–D’’. [file elife-86920-fig3-data1.zip › Figure_3_Source_Data_1 /B'-E_quantification/V794_005%serum labelled.jpg]

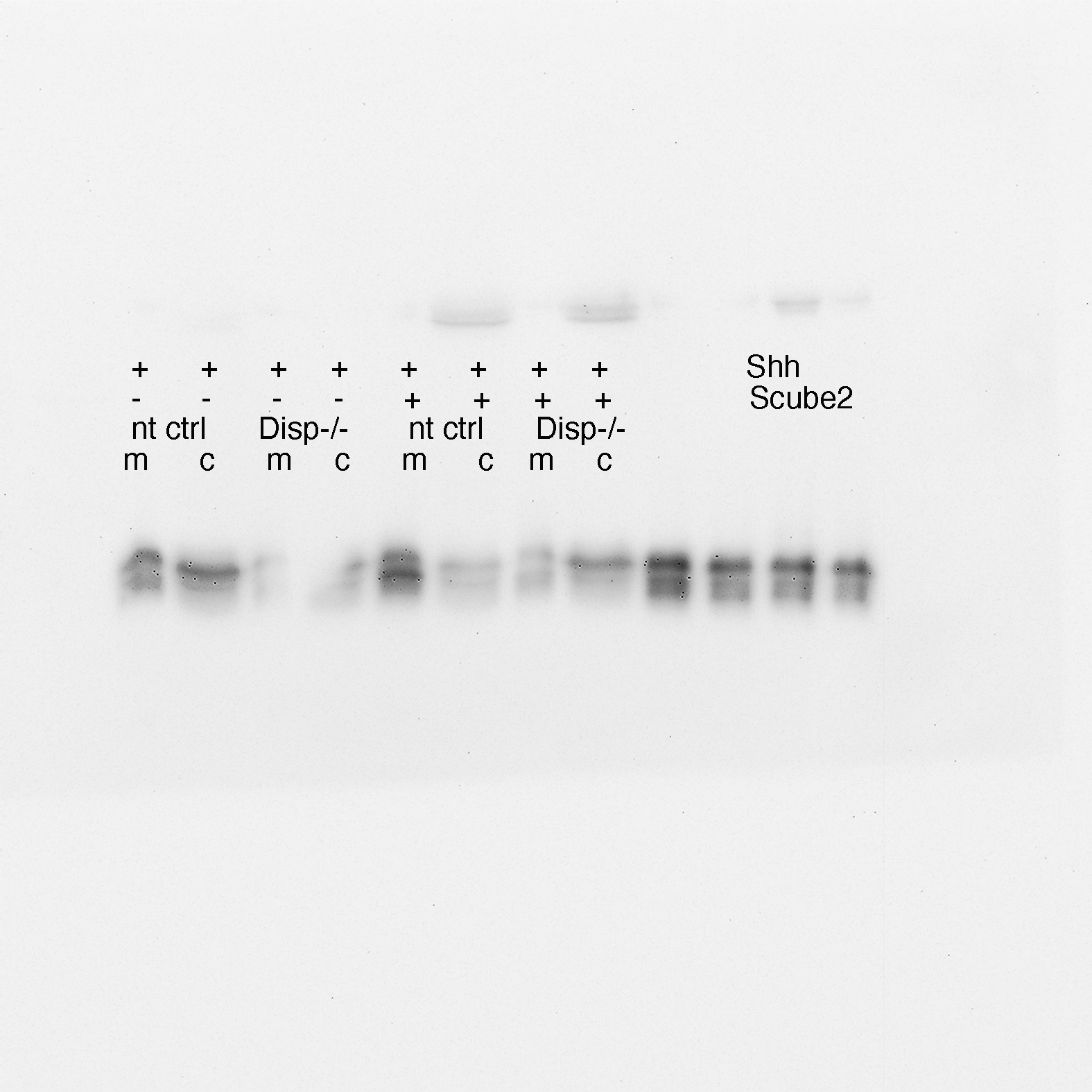

Supplement: Figure 3—source data 1. — A–D contain uncropped western blots shown in Figure 3A–D. Prizm files contain all raw data and statistical analysis to quantify serum-dependent Shh release. B’–E contain uncropped western blots used for the quantification. D` quantifies truncated (proteolytically processed) solubilized Shh, E quantifies relative amounts of unprocessed Shh in media. A’–D’’ Excel file containing raw Shh RP-HPLC elution data as shown in Figure 3A’’–D’’. [file elife-86920-fig3-data1.zip › Figure_3_Source_Data_1 /B'-E_quantification/V794_005%serum labelled.Tif]

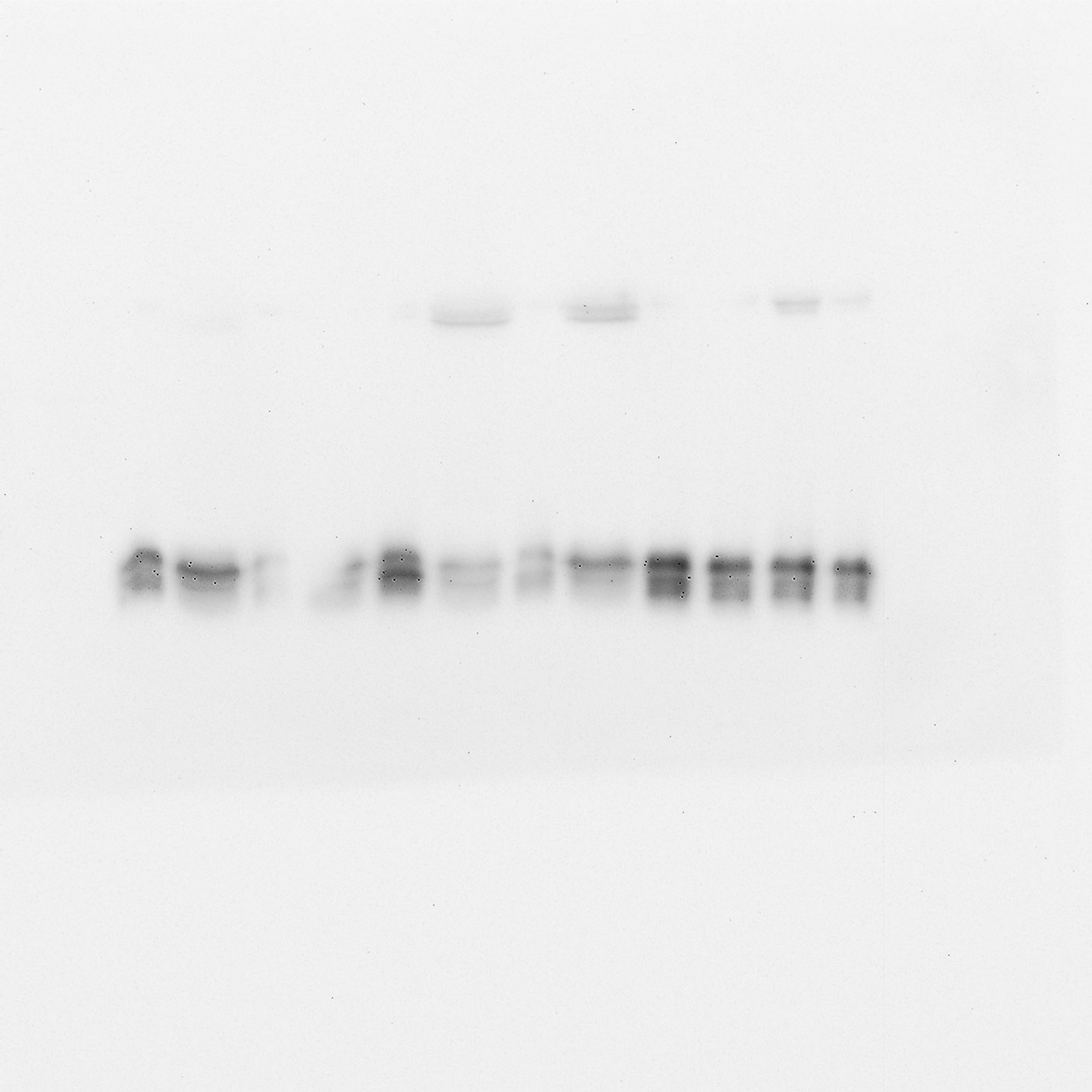

Supplement: Figure 3—source data 1. — A–D contain uncropped western blots shown in Figure 3A–D. Prizm files contain all raw data and statistical analysis to quantify serum-dependent Shh release. B’–E contain uncropped western blots used for the quantification. D` quantifies truncated (proteolytically processed) solubilized Shh, E quantifies relative amounts of unprocessed Shh in media. A’–D’’ Excel file containing raw Shh RP-HPLC elution data as shown in Figure 3A’’–D’’. [file elife-86920-fig3-data1.zip › Figure_3_Source_Data_1 /B'-E_quantification/V794_005%serum.jpg]

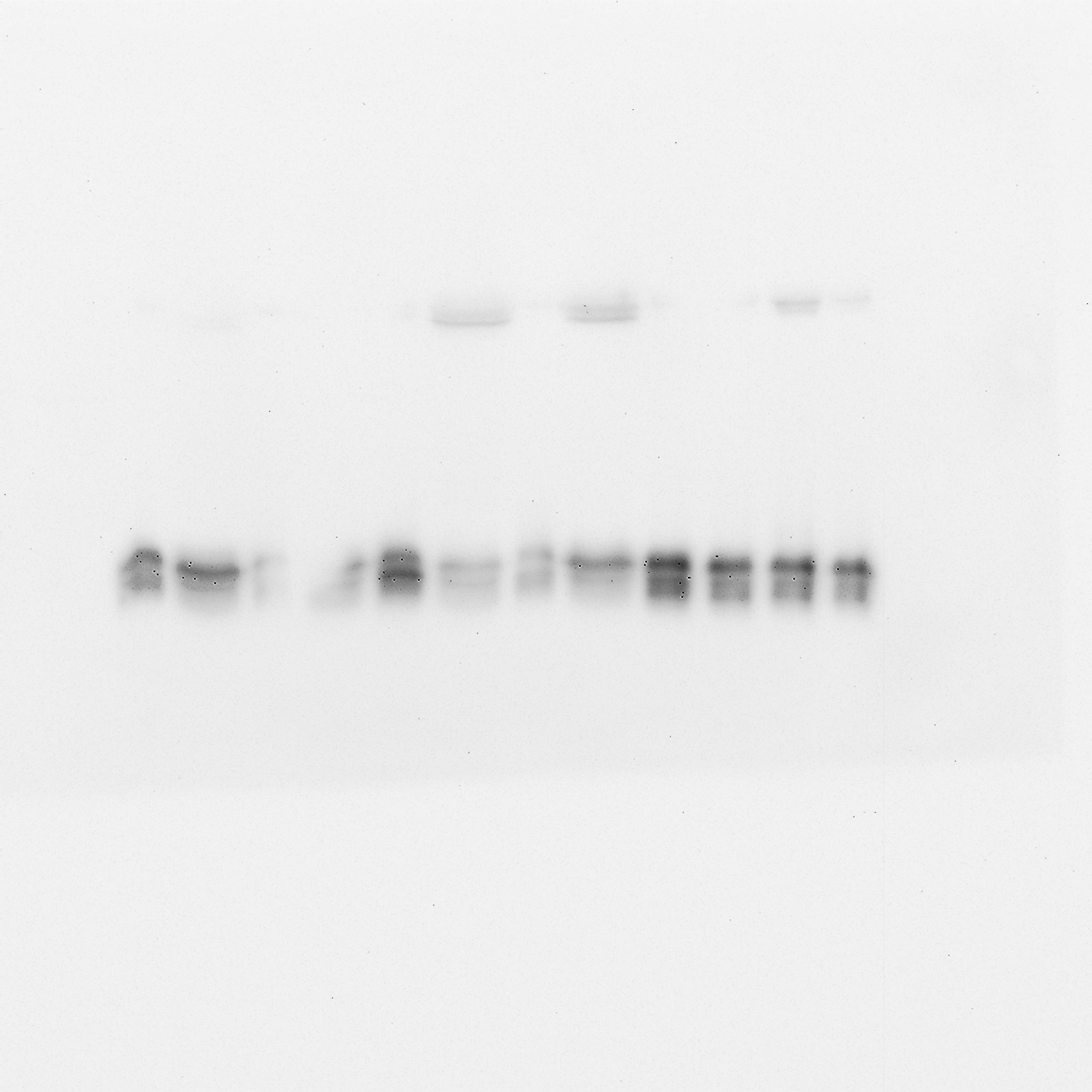

Supplement: Figure 3—source data 1. — A–D contain uncropped western blots shown in Figure 3A–D. Prizm files contain all raw data and statistical analysis to quantify serum-dependent Shh release. B’–E contain uncropped western blots used for the quantification. D` quantifies truncated (proteolytically processed) solubilized Shh, E quantifies relative amounts of unprocessed Shh in media. A’–D’’ Excel file containing raw Shh RP-HPLC elution data as shown in Figure 3A’’–D’’. [file elife-86920-fig3-data1.zip › Figure_3_Source_Data_1 /B'-E_quantification/V794_005%serum.Tif]

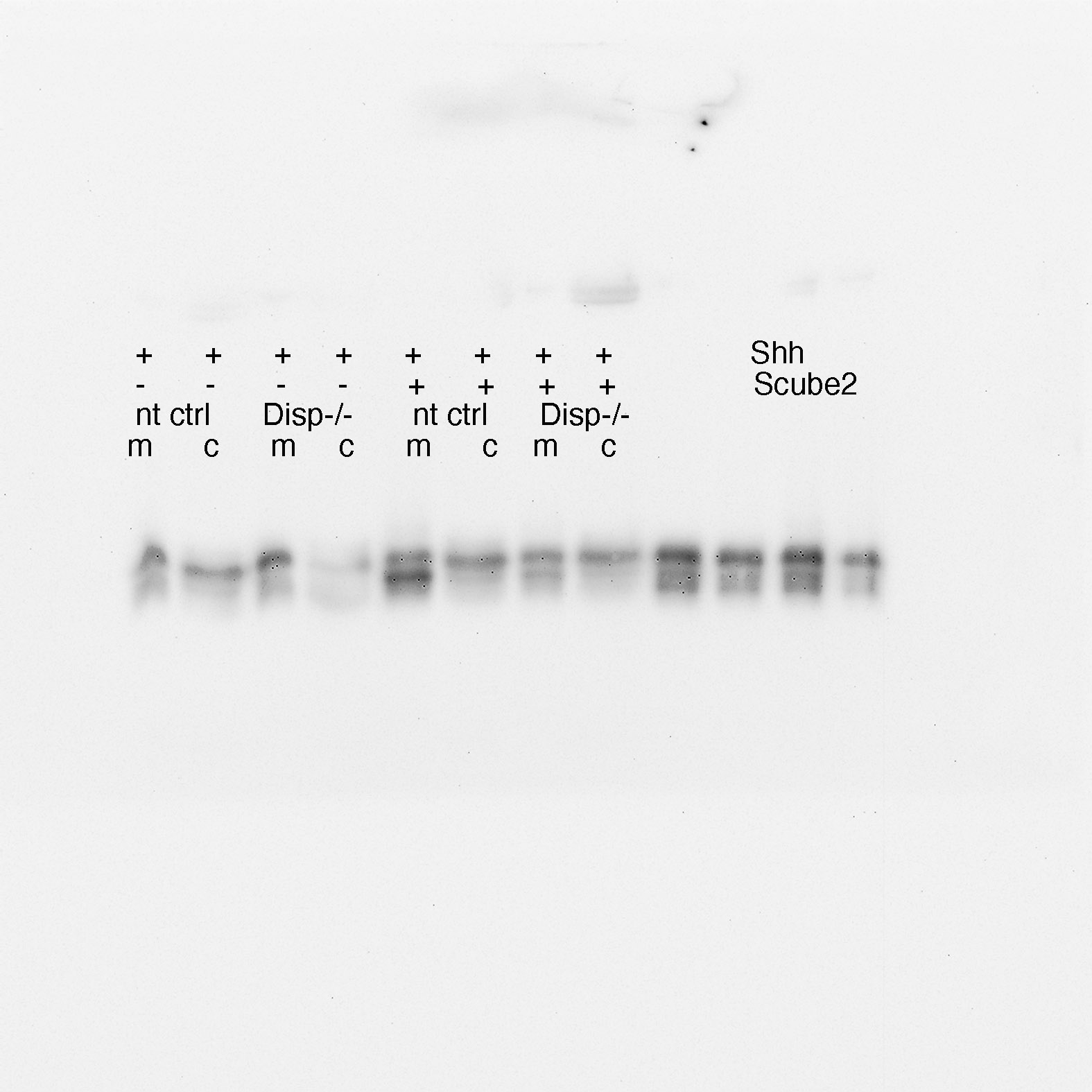

Supplement: Figure 3—source data 1. — A–D contain uncropped western blots shown in Figure 3A–D. Prizm files contain all raw data and statistical analysis to quantify serum-dependent Shh release. B’–E contain uncropped western blots used for the quantification. D` quantifies truncated (proteolytically processed) solubilized Shh, E quantifies relative amounts of unprocessed Shh in media. A’–D’’ Excel file containing raw Shh RP-HPLC elution data as shown in Figure 3A’’–D’’. [file elife-86920-fig3-data1.zip › Figure_3_Source_Data_1 /B'-E_quantification/V794_005%serum_2 labelled.jpg]

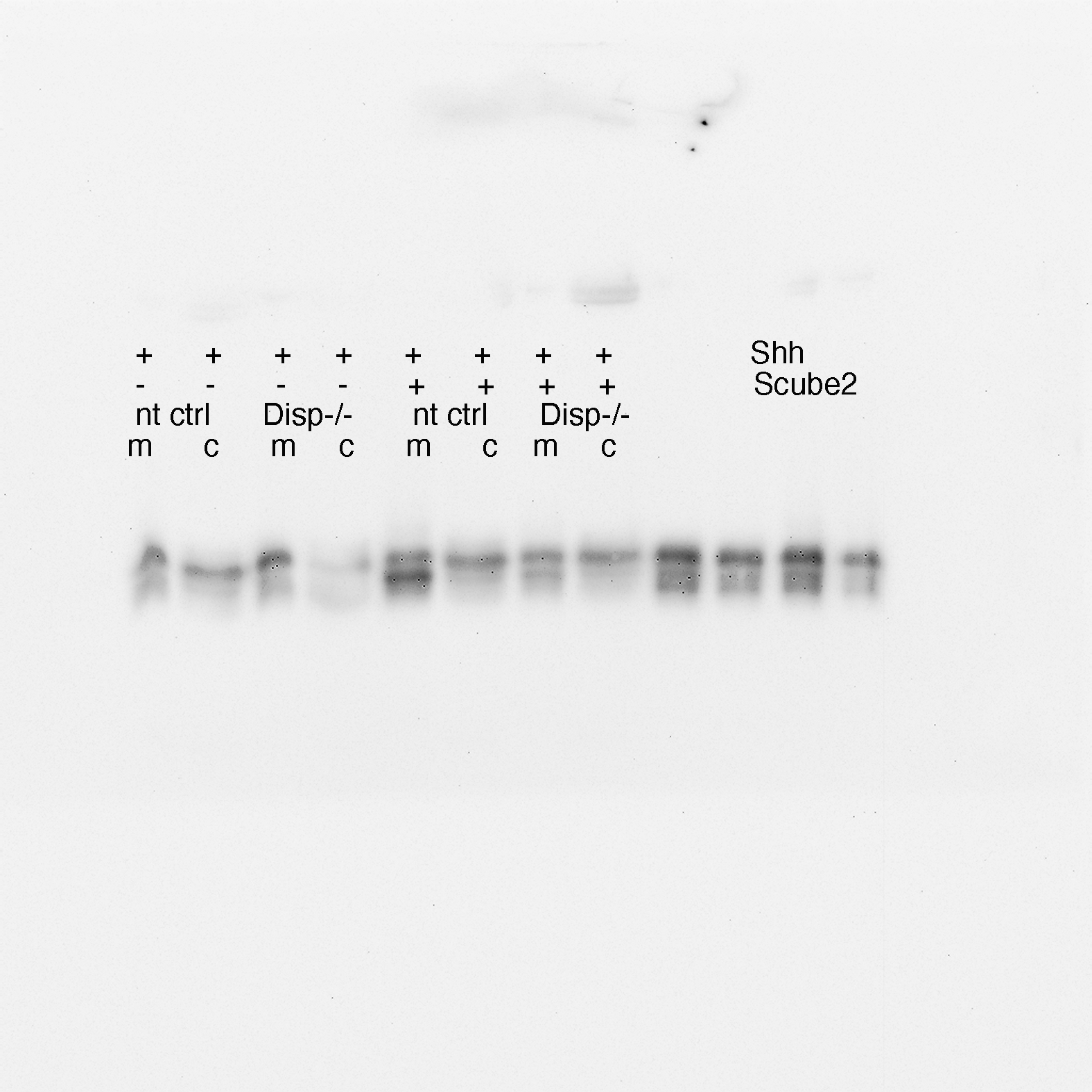

Supplement: Figure 3—source data 1. — A–D contain uncropped western blots shown in Figure 3A–D. Prizm files contain all raw data and statistical analysis to quantify serum-dependent Shh release. B’–E contain uncropped western blots used for the quantification. D` quantifies truncated (proteolytically processed) solubilized Shh, E quantifies relative amounts of unprocessed Shh in media. A’–D’’ Excel file containing raw Shh RP-HPLC elution data as shown in Figure 3A’’–D’’. [file elife-86920-fig3-data1.zip › Figure_3_Source_Data_1 /B'-E_quantification/V794_005%serum_2 labelled.Tif]

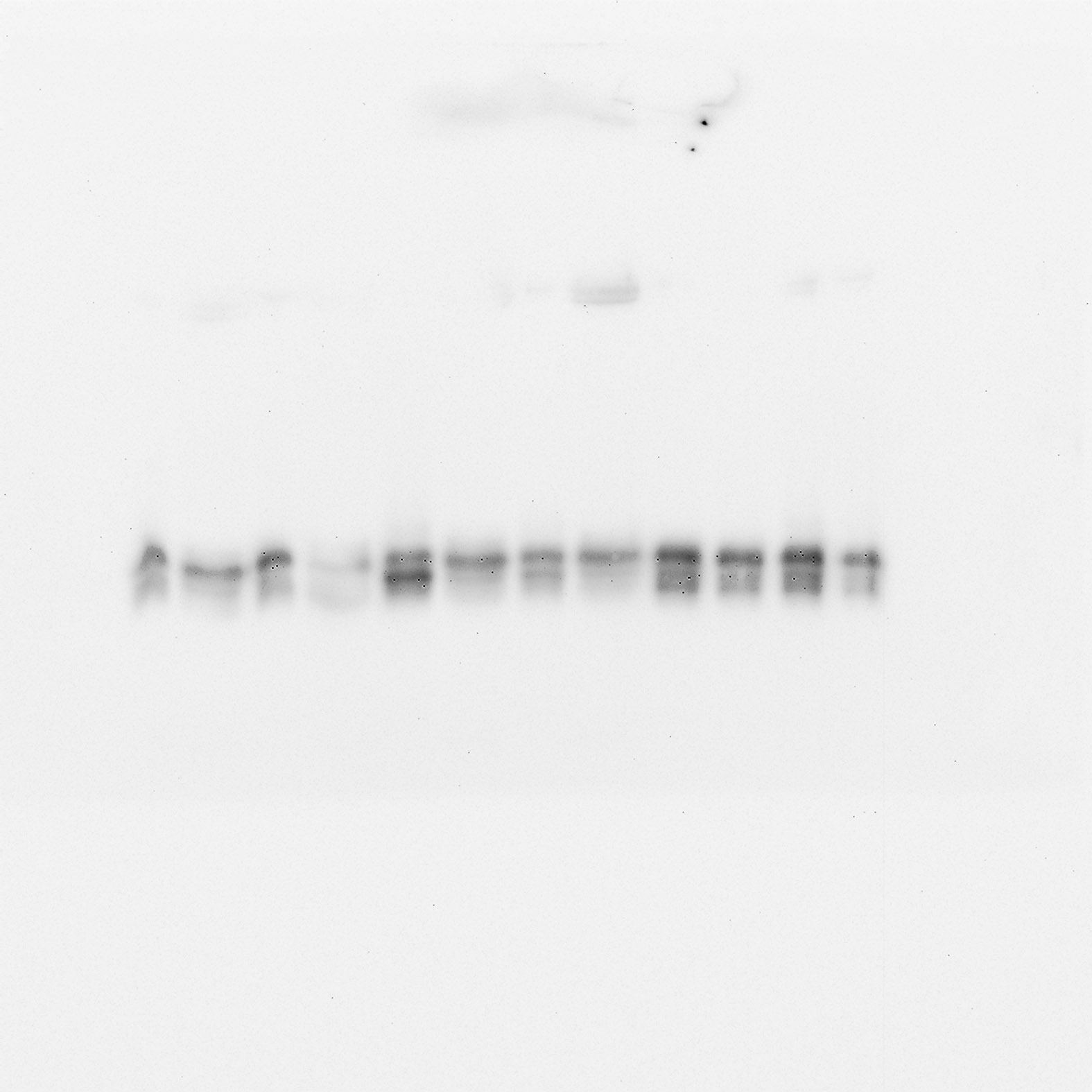

Supplement: Figure 3—source data 1. — A–D contain uncropped western blots shown in Figure 3A–D. Prizm files contain all raw data and statistical analysis to quantify serum-dependent Shh release. B’–E contain uncropped western blots used for the quantification. D` quantifies truncated (proteolytically processed) solubilized Shh, E quantifies relative amounts of unprocessed Shh in media. A’–D’’ Excel file containing raw Shh RP-HPLC elution data as shown in Figure 3A’’–D’’. [file elife-86920-fig3-data1.zip › Figure_3_Source_Data_1 /B'-E_quantification/V794_005%serum_2.jpg]

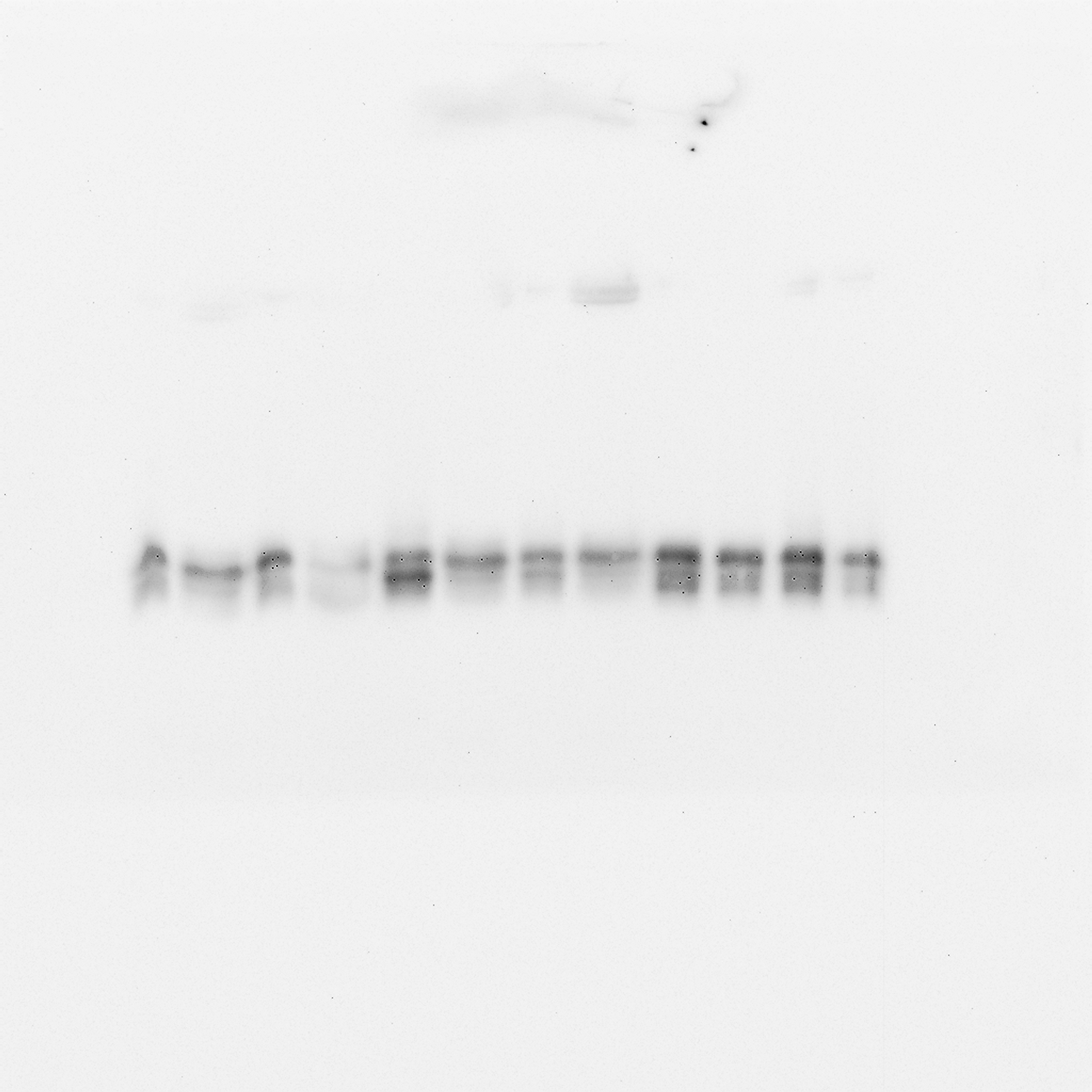

Supplement: Figure 3—source data 1. — A–D contain uncropped western blots shown in Figure 3A–D. Prizm files contain all raw data and statistical analysis to quantify serum-dependent Shh release. B’–E contain uncropped western blots used for the quantification. D` quantifies truncated (proteolytically processed) solubilized Shh, E quantifies relative amounts of unprocessed Shh in media. A’–D’’ Excel file containing raw Shh RP-HPLC elution data as shown in Figure 3A’’–D’’. [file elife-86920-fig3-data1.zip › Figure_3_Source_Data_1 /B'-E_quantification/V794_005%serum_2.Tif]

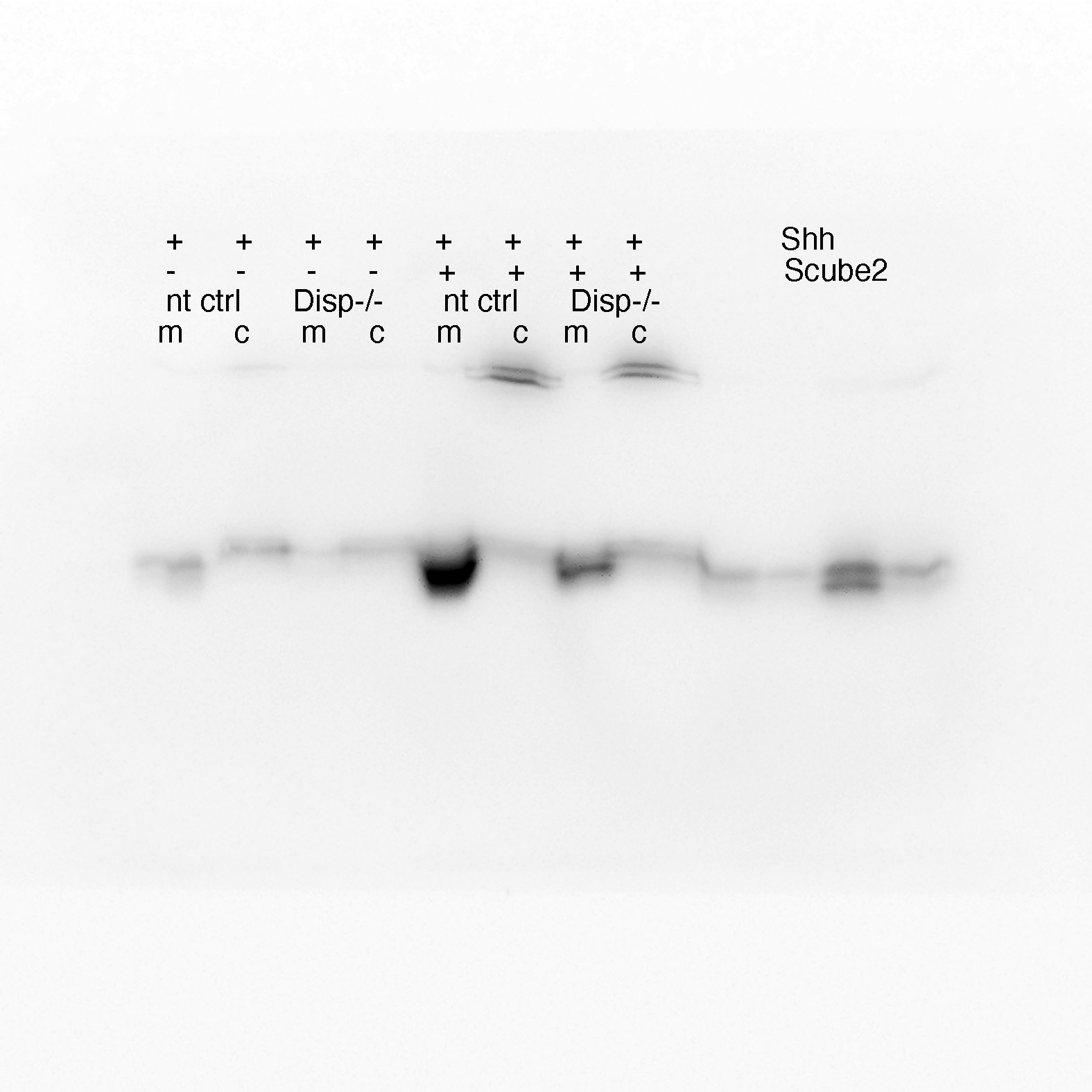

Supplement: Figure 3—source data 1. — A–D contain uncropped western blots shown in Figure 3A–D. Prizm files contain all raw data and statistical analysis to quantify serum-dependent Shh release. B’–E contain uncropped western blots used for the quantification. D` quantifies truncated (proteolytically processed) solubilized Shh, E quantifies relative amounts of unprocessed Shh in media. A’–D’’ Excel file containing raw Shh RP-HPLC elution data as shown in Figure 3A’’–D’’. [file elife-86920-fig3-data1.zip › Figure_3_Source_Data_1 /B'-E_quantification/VK193_10%serum labelled.jpg]

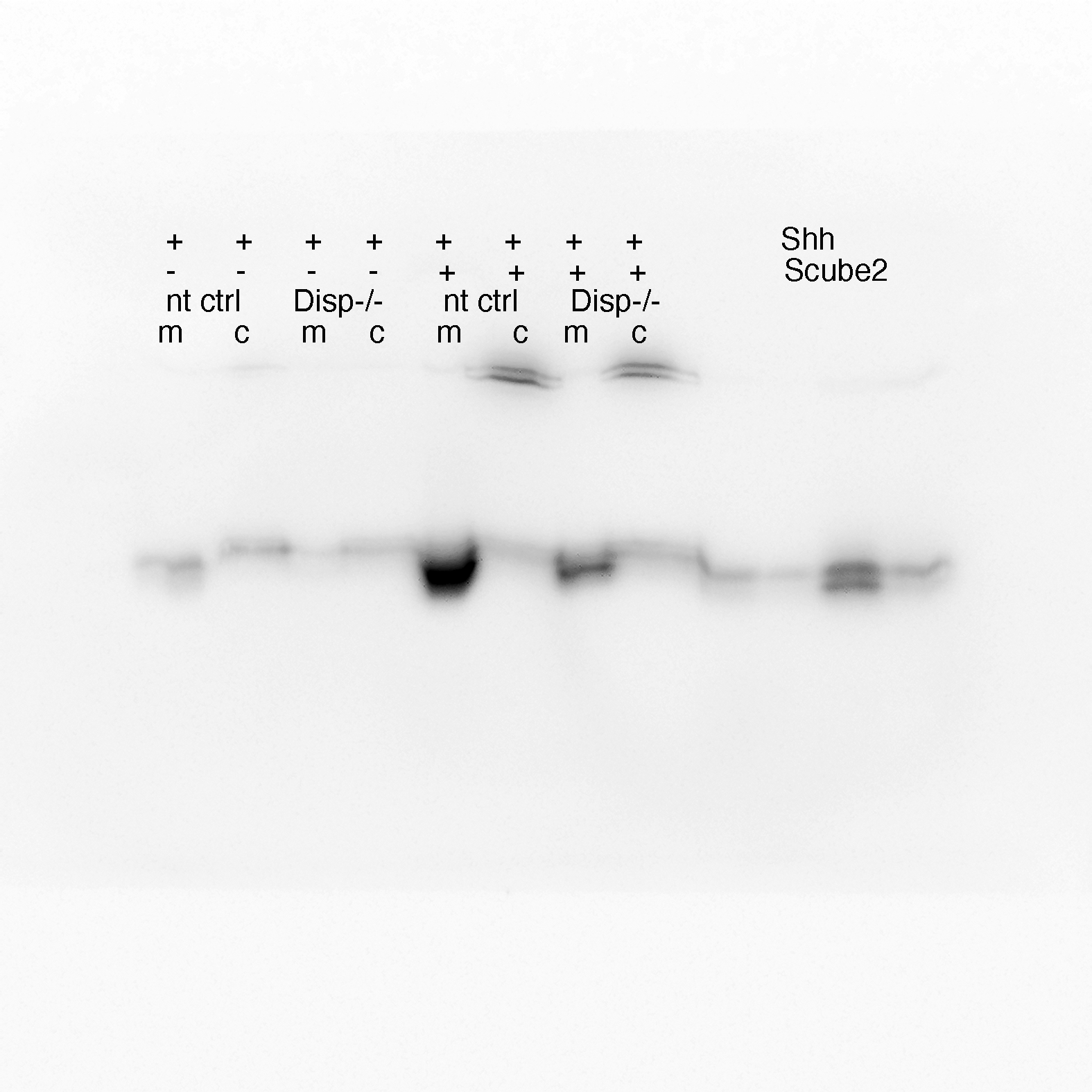

Supplement: Figure 3—source data 1. — A–D contain uncropped western blots shown in Figure 3A–D. Prizm files contain all raw data and statistical analysis to quantify serum-dependent Shh release. B’–E contain uncropped western blots used for the quantification. D` quantifies truncated (proteolytically processed) solubilized Shh, E quantifies relative amounts of unprocessed Shh in media. A’–D’’ Excel file containing raw Shh RP-HPLC elution data as shown in Figure 3A’’–D’’. [file elife-86920-fig3-data1.zip › Figure_3_Source_Data_1 /B'-E_quantification/VK193_10%serum labelled.tif]

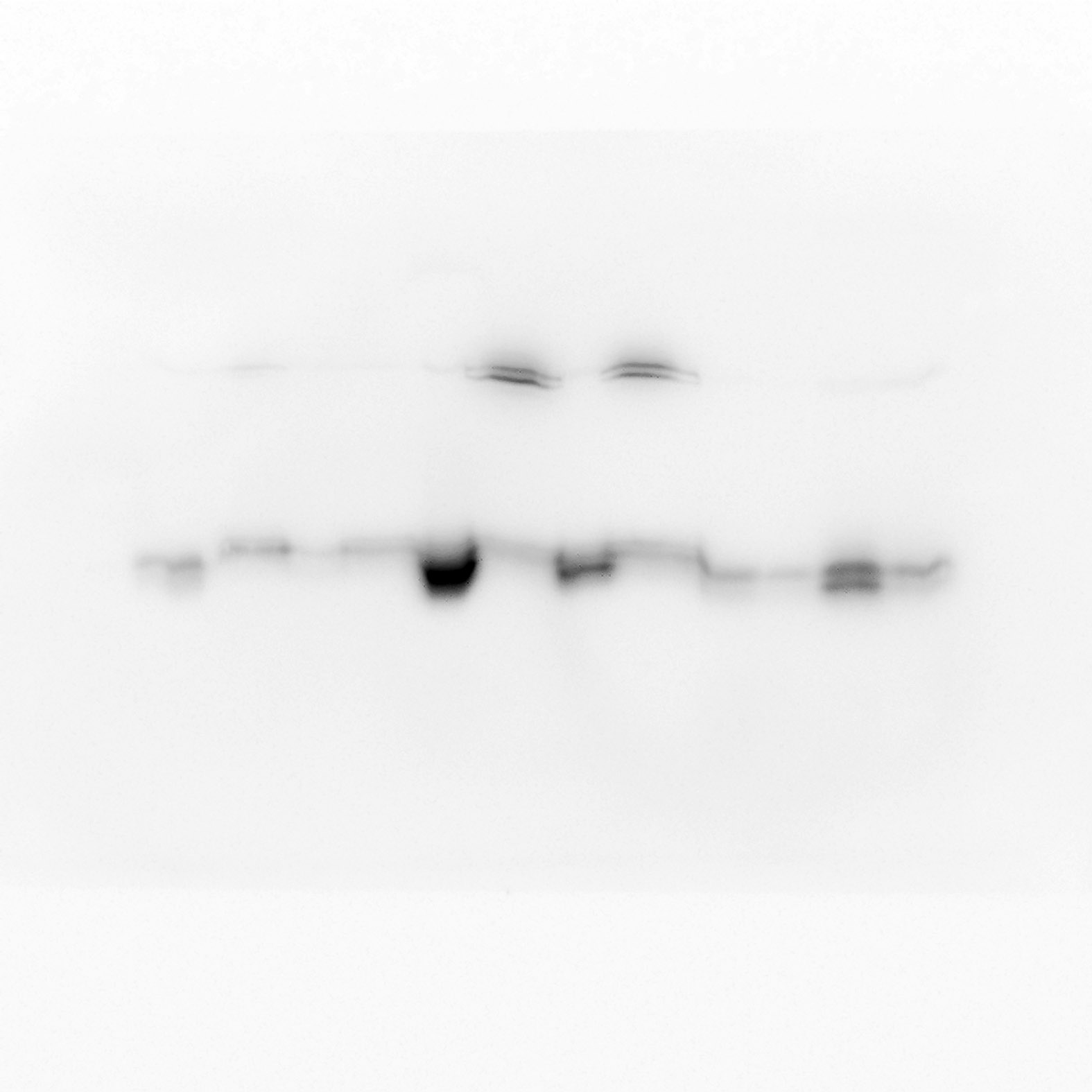

Supplement: Figure 3—source data 1. — A–D contain uncropped western blots shown in Figure 3A–D. Prizm files contain all raw data and statistical analysis to quantify serum-dependent Shh release. B’–E contain uncropped western blots used for the quantification. D` quantifies truncated (proteolytically processed) solubilized Shh, E quantifies relative amounts of unprocessed Shh in media. A’–D’’ Excel file containing raw Shh RP-HPLC elution data as shown in Figure 3A’’–D’’. [file elife-86920-fig3-data1.zip › Figure_3_Source_Data_1 /B'-E_quantification/VK193_10%serum.jpg]

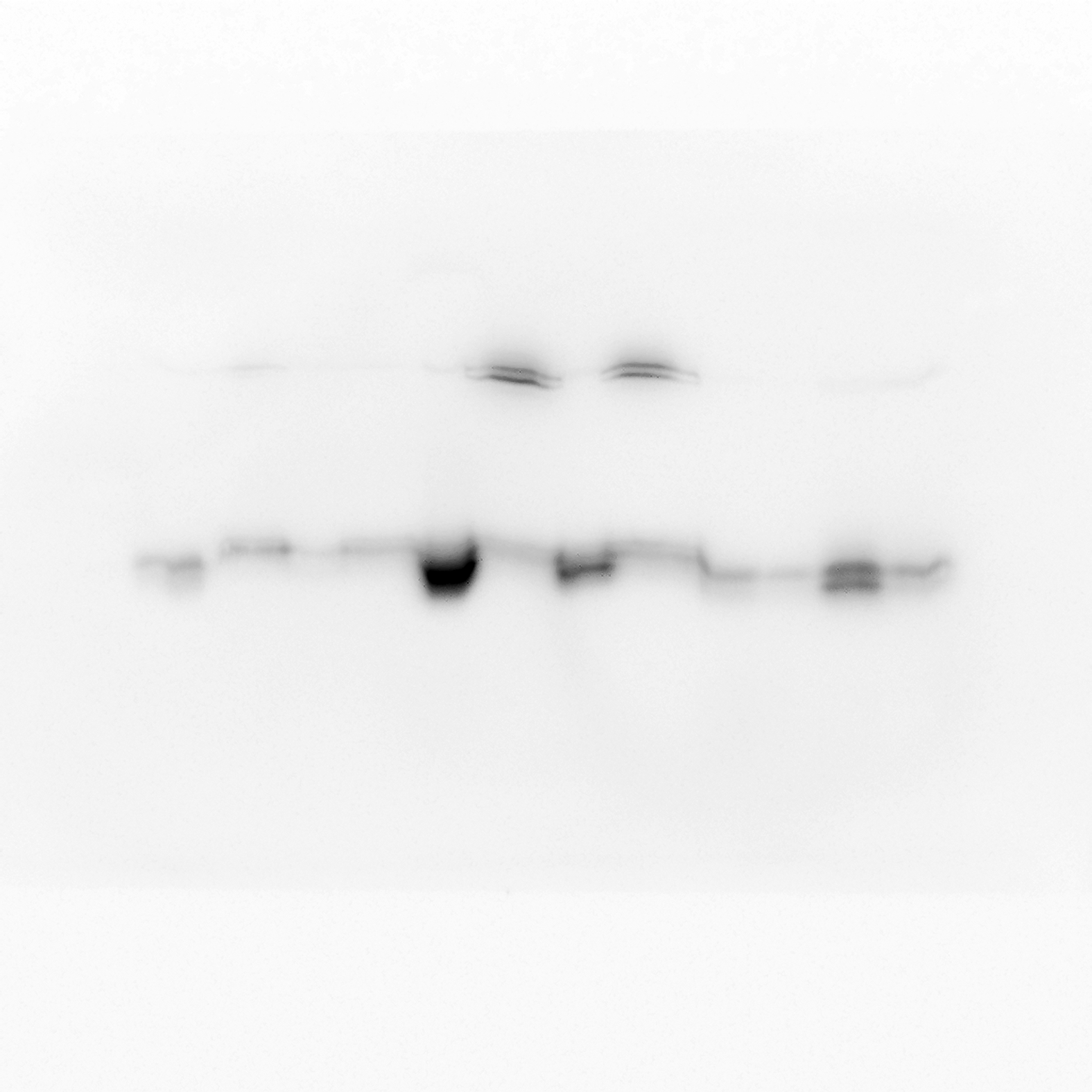

Supplement: Figure 3—source data 1. — A–D contain uncropped western blots shown in Figure 3A–D. Prizm files contain all raw data and statistical analysis to quantify serum-dependent Shh release. B’–E contain uncropped western blots used for the quantification. D` quantifies truncated (proteolytically processed) solubilized Shh, E quantifies relative amounts of unprocessed Shh in media. A’–D’’ Excel file containing raw Shh RP-HPLC elution data as shown in Figure 3A’’–D’’. [file elife-86920-fig3-data1.zip › Figure_3_Source_Data_1 /B'-E_quantification/VK193_10%serum.tif]

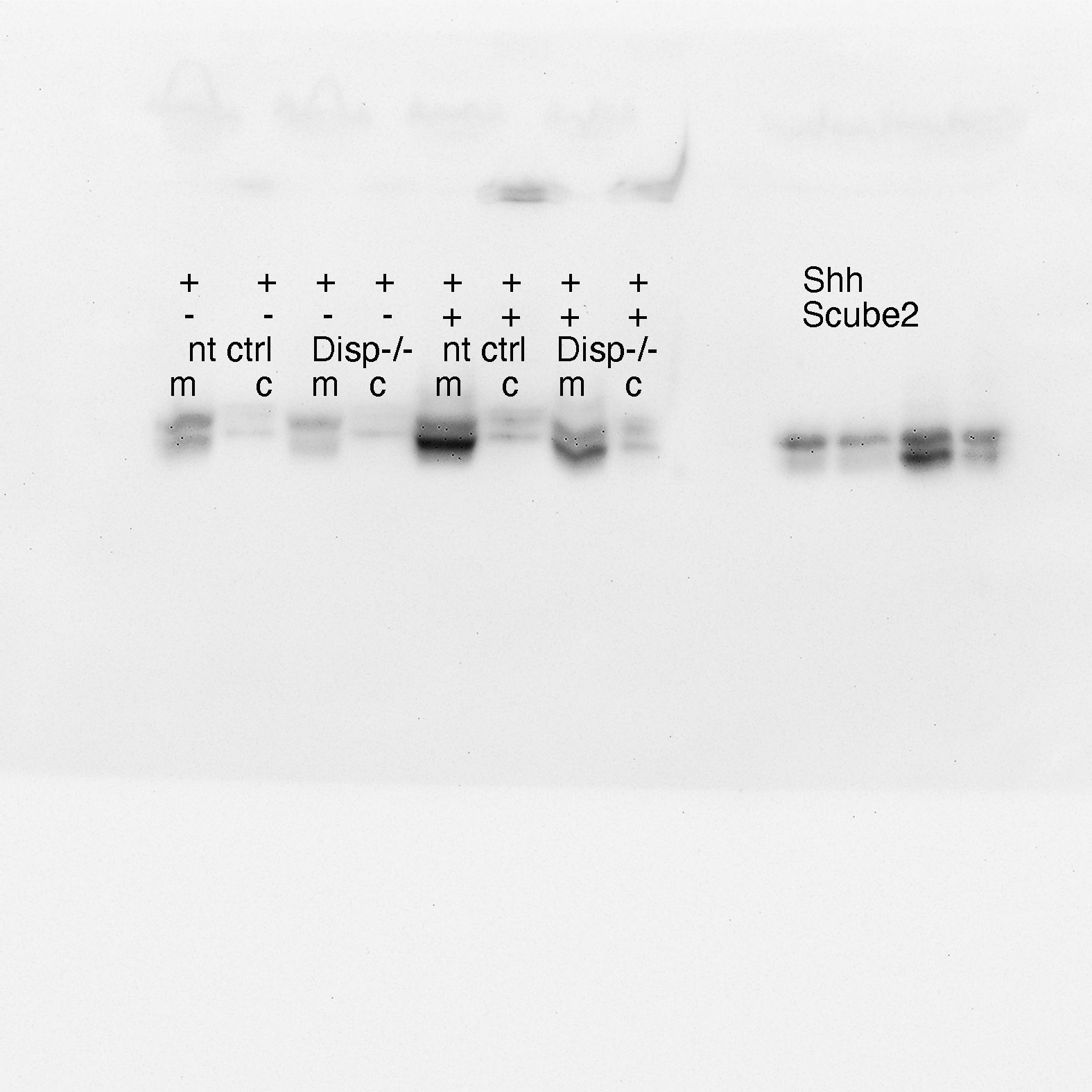

Supplement: Figure 3—figure supplement 1—source data 1. [file elife-86920-fig3-figsupp1-data1.zip › Figure 3-Figure Supplement 1 - Source Data 1/A_20-01-22_Gel 2_antiShh Rabbit_3min labelled.jpg]

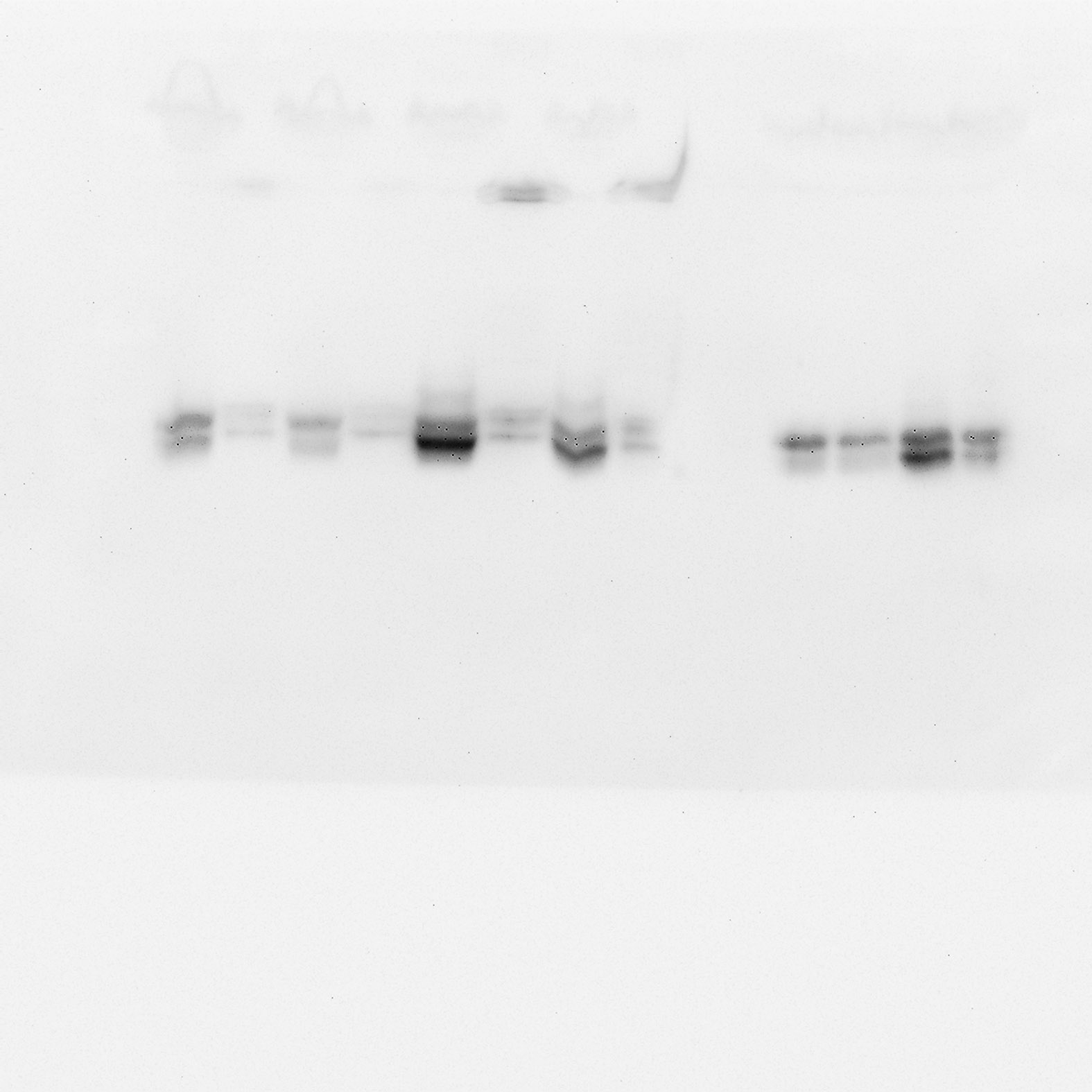

Supplement: Figure 3—figure supplement 1—source data 1. [file elife-86920-fig3-figsupp1-data1.zip › Figure 3-Figure Supplement 1 - Source Data 1/A_20-01-22_Gel 2_antiShh Rabbit_3min.jpg]

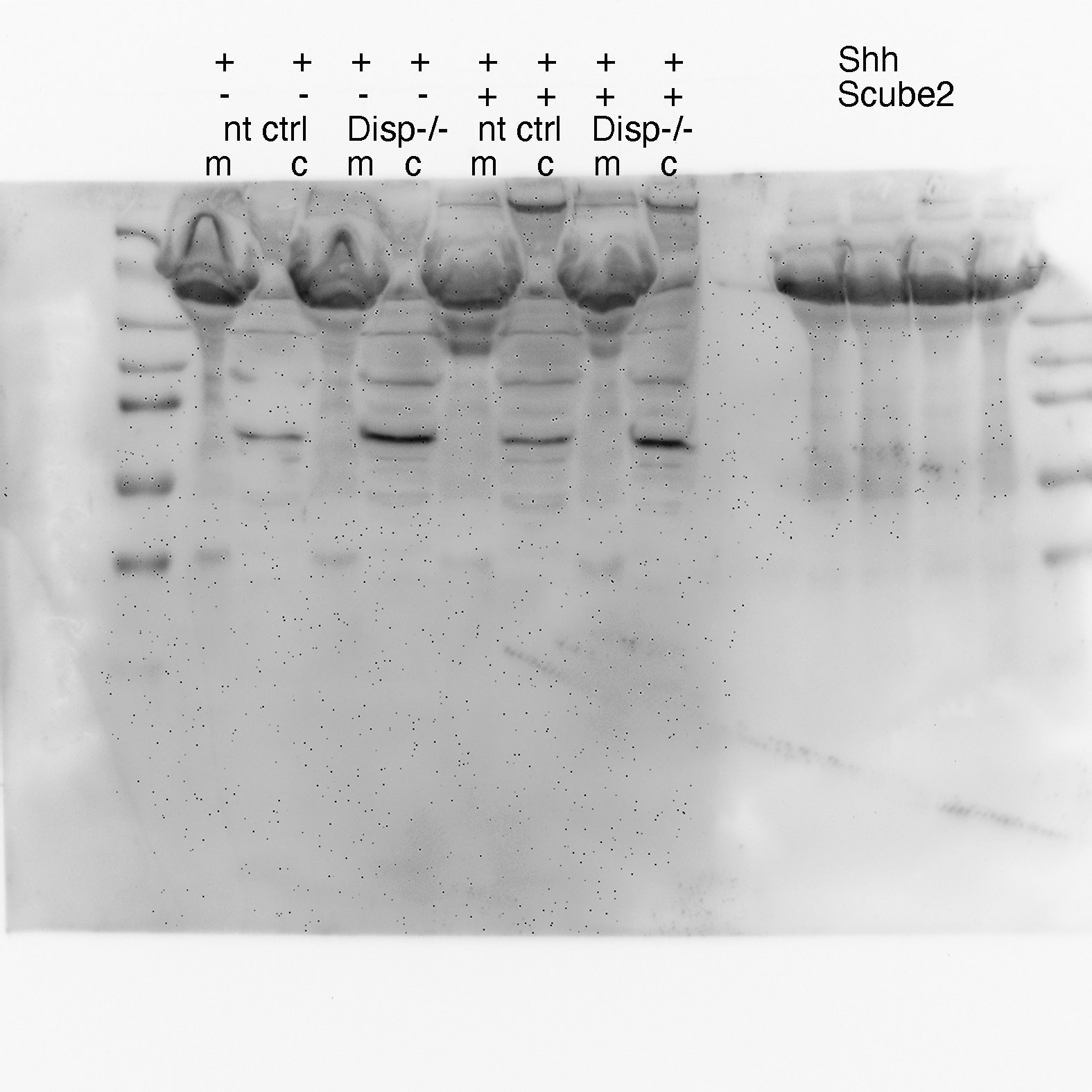

Supplement: Figure 3—figure supplement 1—source data 1. [file elife-86920-fig3-figsupp1-data1.zip › Figure 3-Figure Supplement 1 - Source Data 1/A_V750_2_FLAG_1min labelled.jpg]

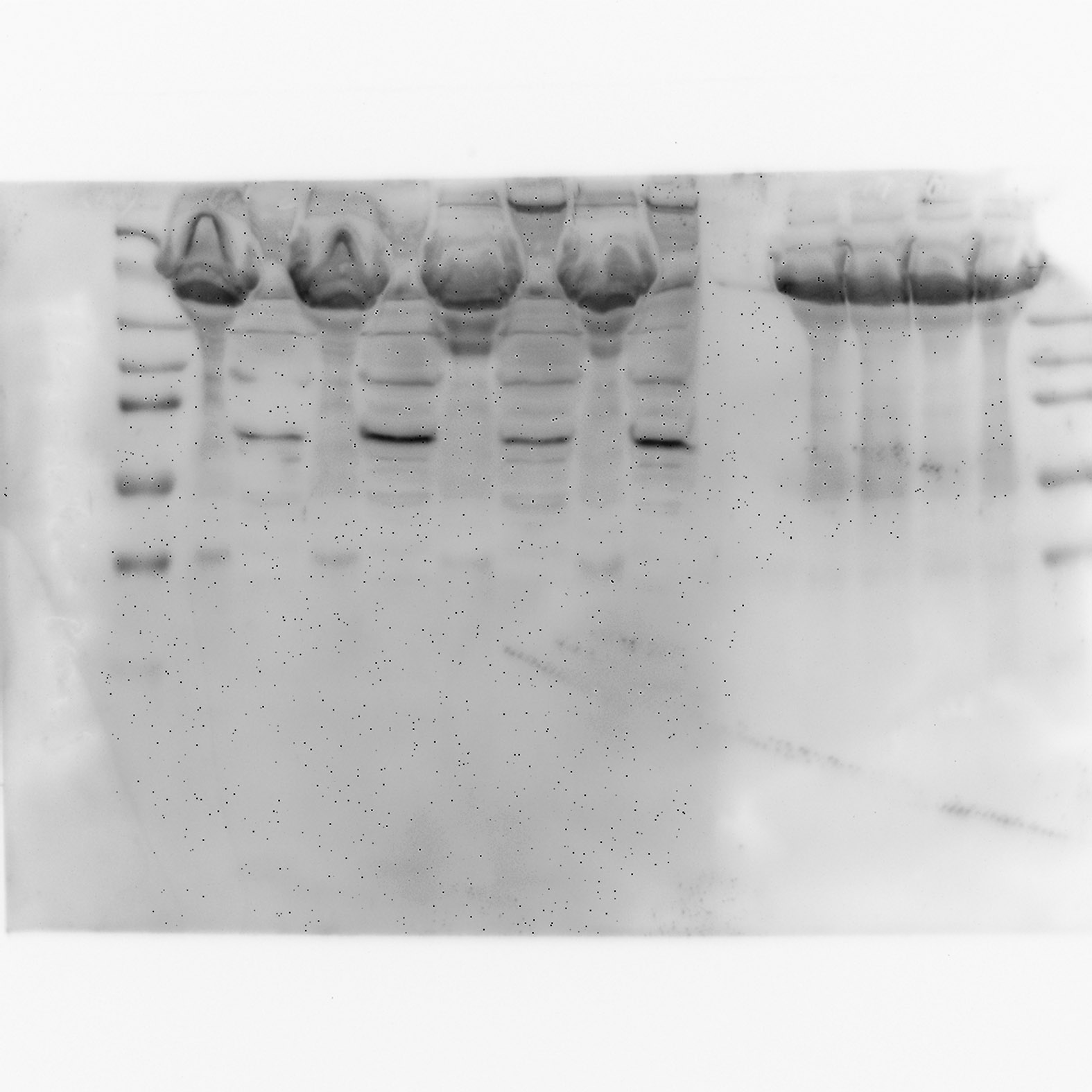

Supplement: Figure 3—figure supplement 1—source data 1. [file elife-86920-fig3-figsupp1-data1.zip › Figure 3-Figure Supplement 1 - Source Data 1/A_V750_2_FLAG_1min.jpg]

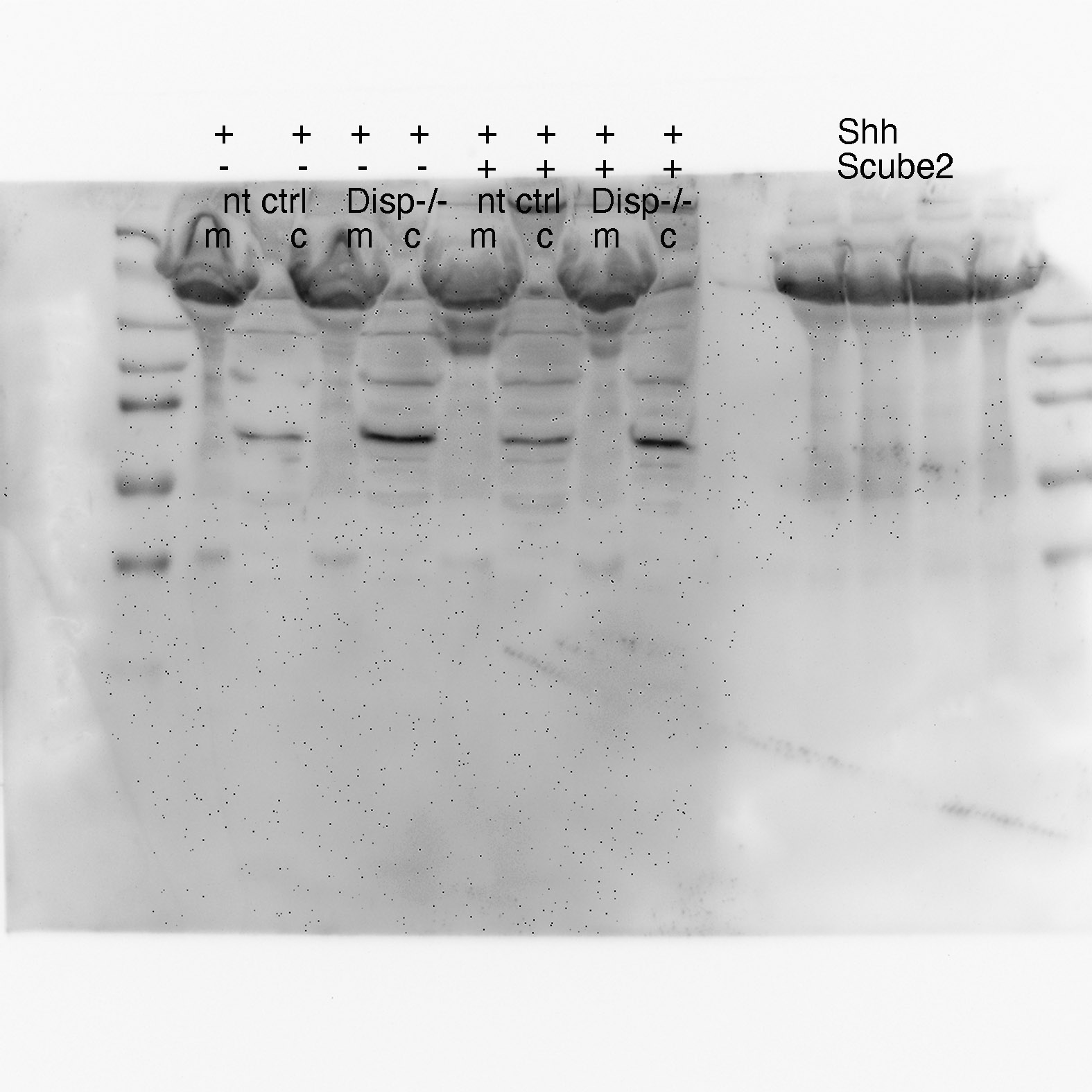

Supplement: Figure 3—figure supplement 1—source data 1. [file elife-86920-fig3-figsupp1-data1.zip › Figure 3-Figure Supplement 1 - Source Data 1/A_V750_2_Pon_1min labelled.jpg]

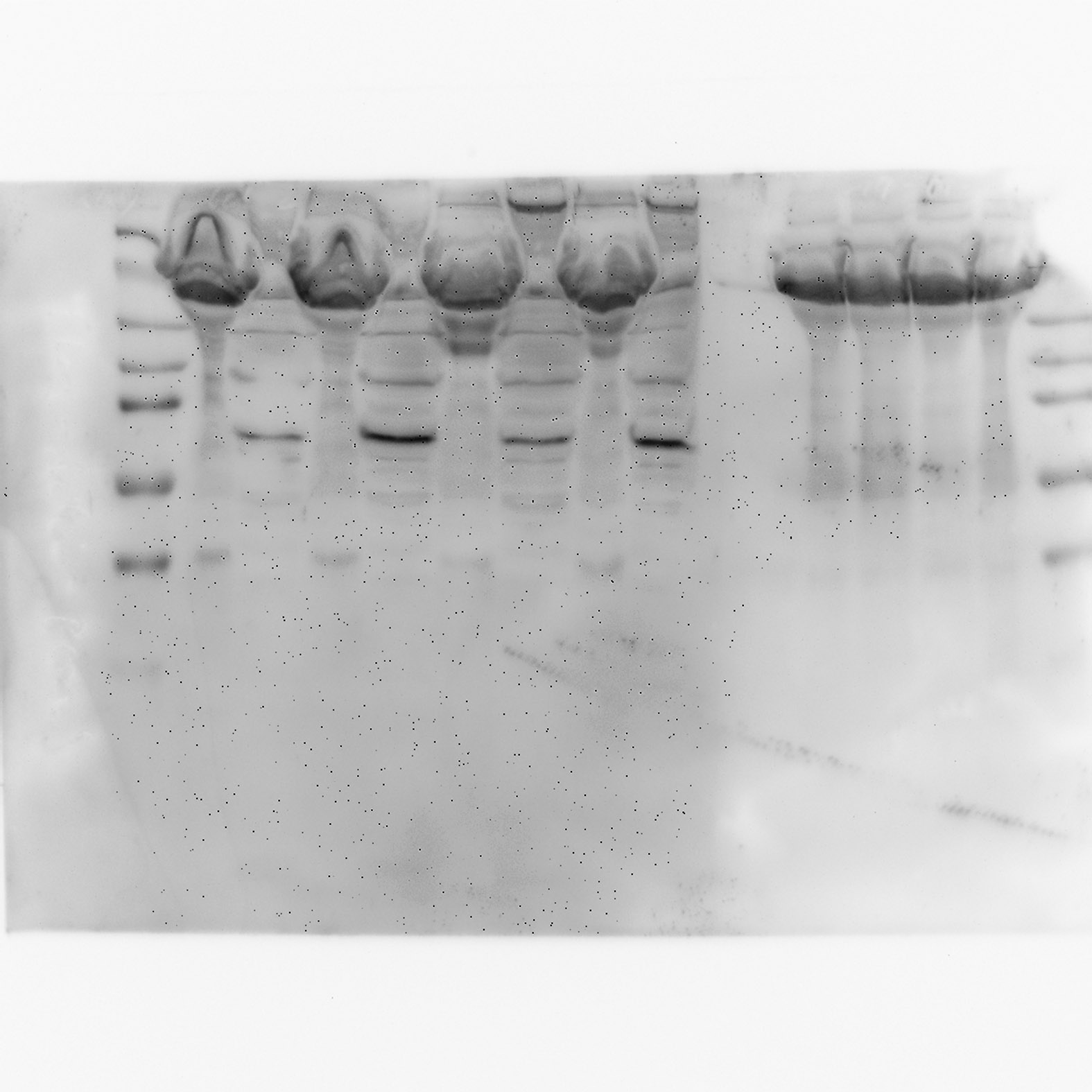

Supplement: Figure 3—figure supplement 1—source data 1. [file elife-86920-fig3-figsupp1-data1.zip › Figure 3-Figure Supplement 1 - Source Data 1/A_V750_2_Pon_1min.jpg]

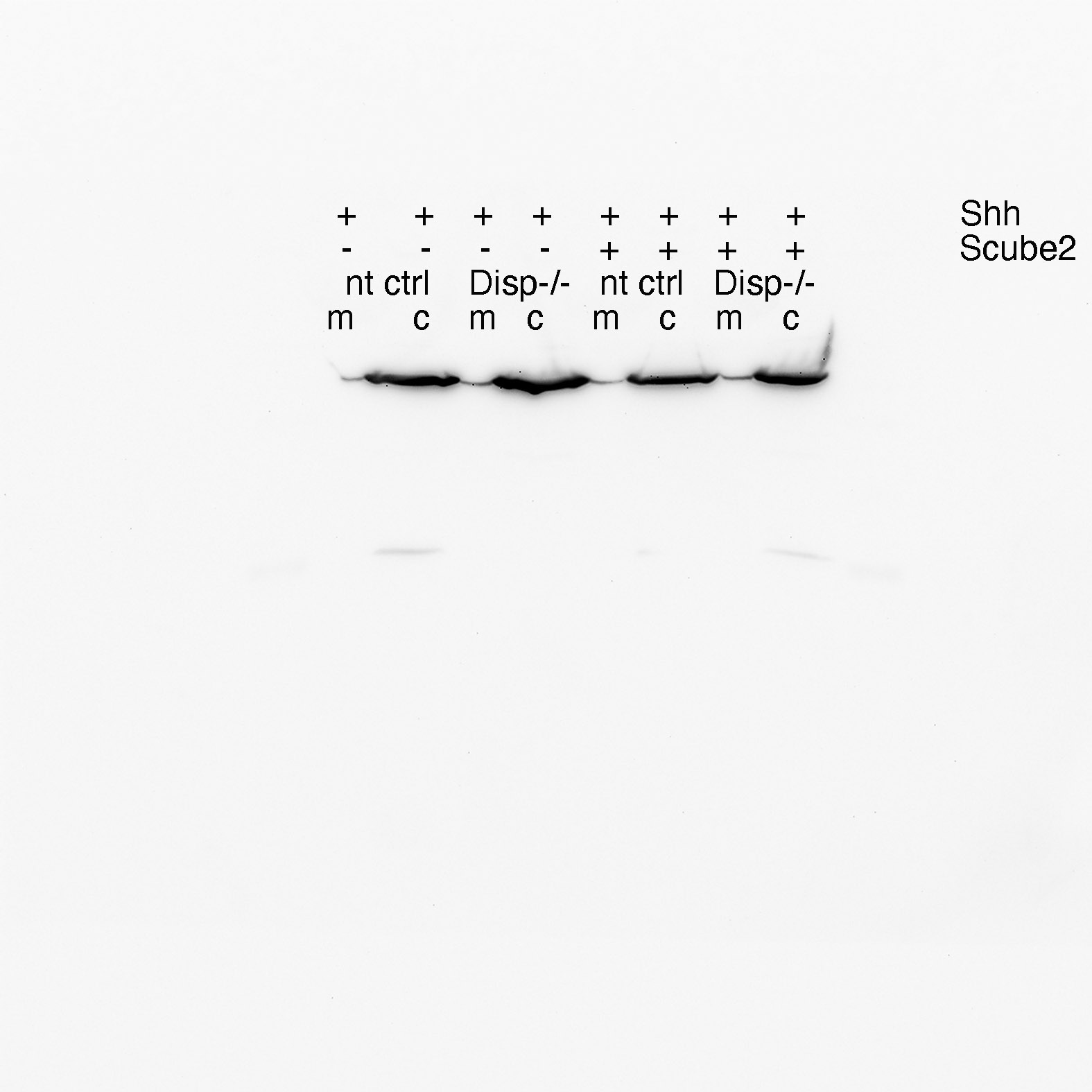

Supplement: Figure 3—figure supplement 1—source data 1. [file elife-86920-fig3-figsupp1-data1.zip › Figure 3-Figure Supplement 1 - Source Data 1/A_V750_4_actin_2min labelled.jpg]

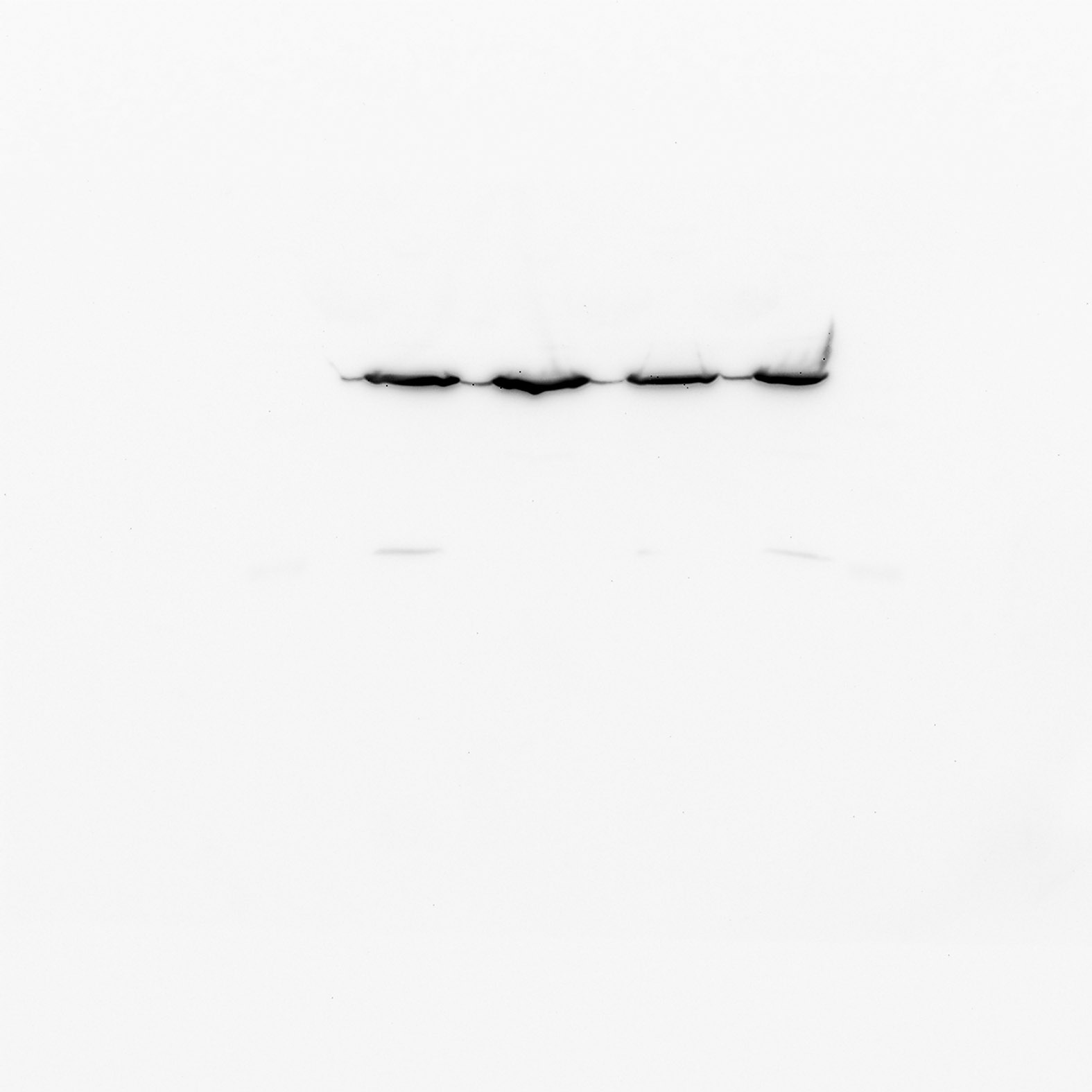

Supplement: Figure 3—figure supplement 1—source data 1. [file elife-86920-fig3-figsupp1-data1.zip › Figure 3-Figure Supplement 1 - Source Data 1/A_V750_4_actin_2min.jpg]

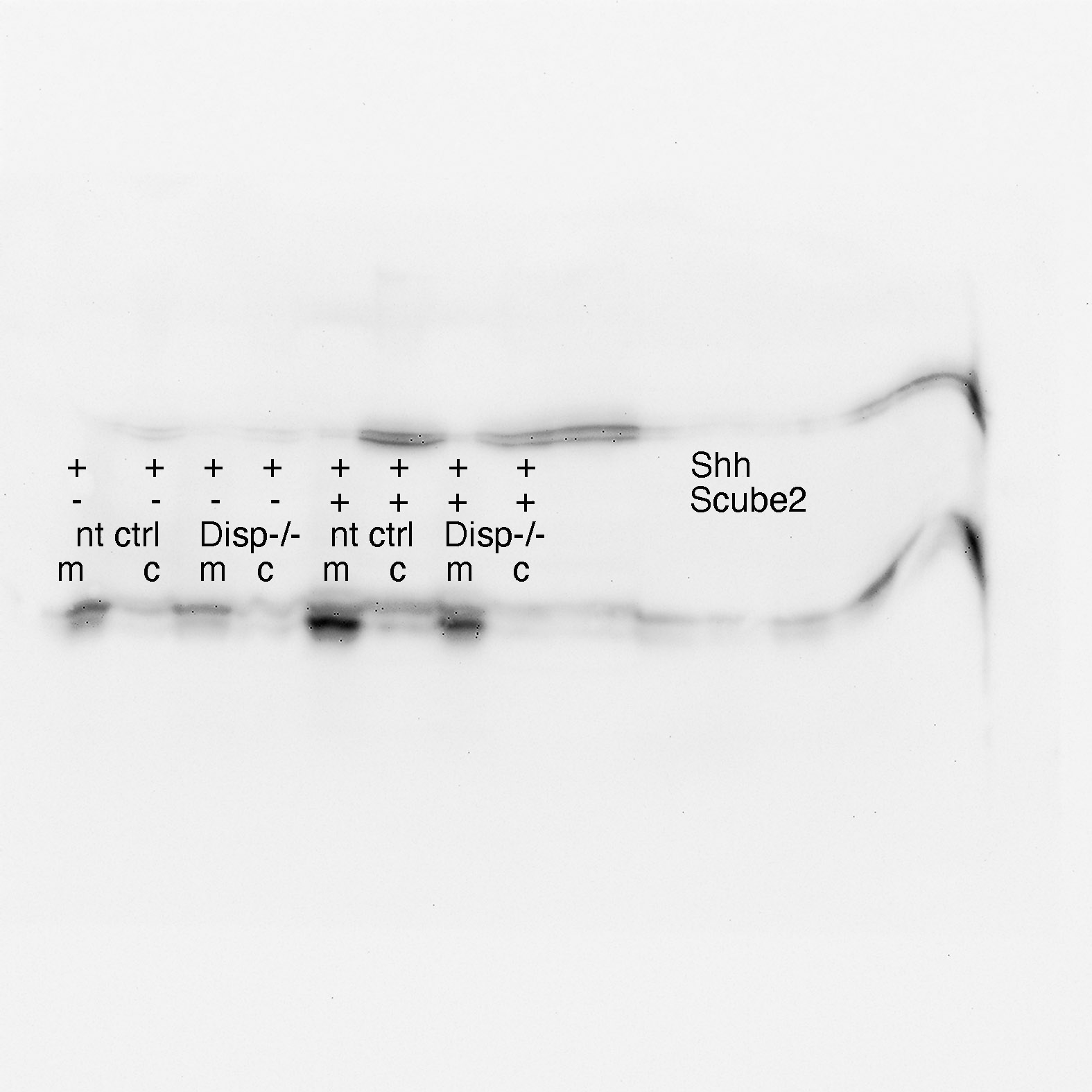

Supplement: Figure 3—figure supplement 1—source data 1. [file elife-86920-fig3-figsupp1-data1.zip › Figure 3-Figure Supplement 1 - Source Data 1/B_V744_2_2min labelled.jpg]

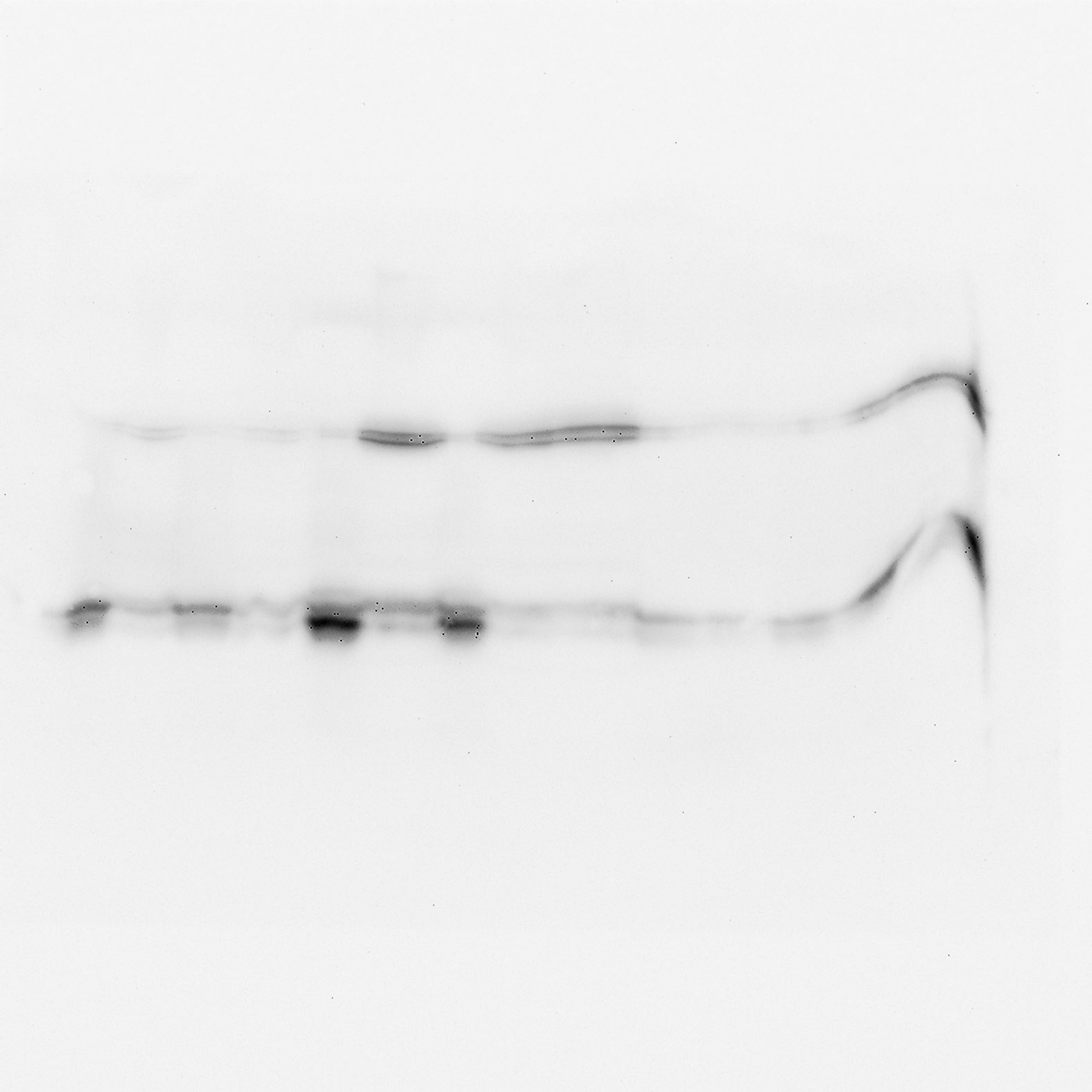

Supplement: Figure 3—figure supplement 1—source data 1. [file elife-86920-fig3-figsupp1-data1.zip › Figure 3-Figure Supplement 1 - Source Data 1/B_V744_2_2min.jpg]

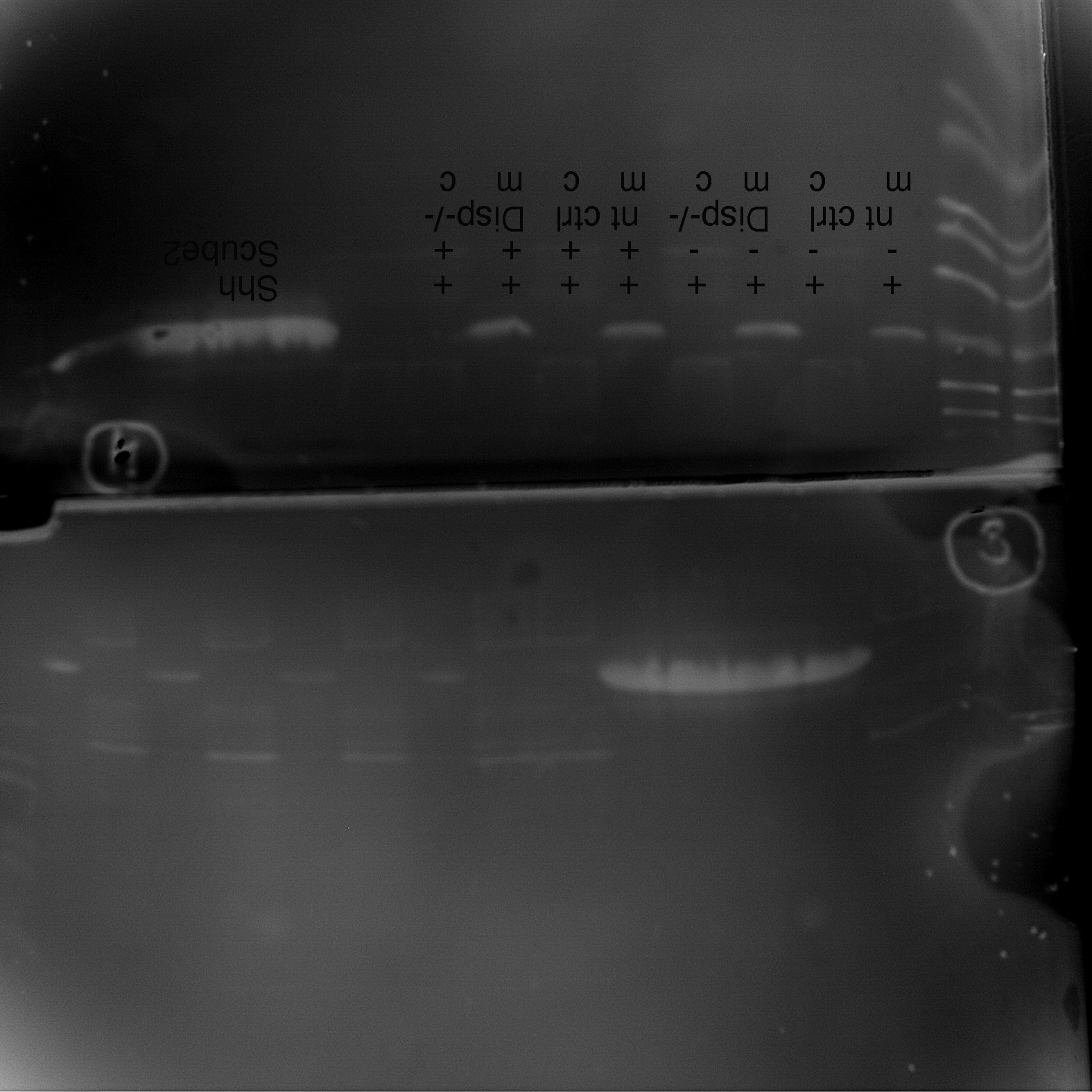

Supplement: Figure 3—figure supplement 1—source data 1. [file elife-86920-fig3-figsupp1-data1.zip › Figure 3-Figure Supplement 1 - Source Data 1/B_V744_3+4_Pon labelled.jpg]

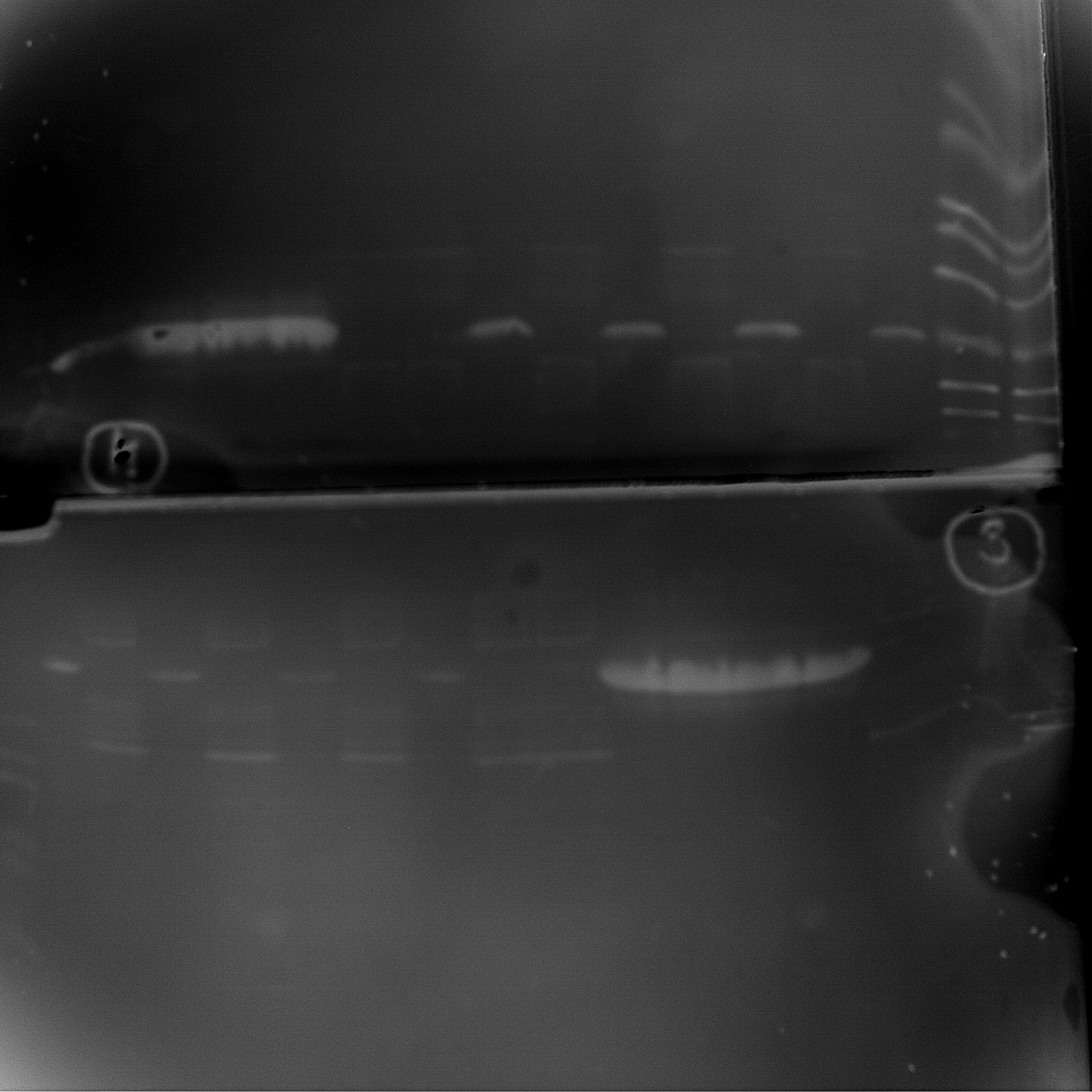

Supplement: Figure 3—figure supplement 1—source data 1. [file elife-86920-fig3-figsupp1-data1.zip › Figure 3-Figure Supplement 1 - Source Data 1/B_V744_3+4_Pon.jpg]

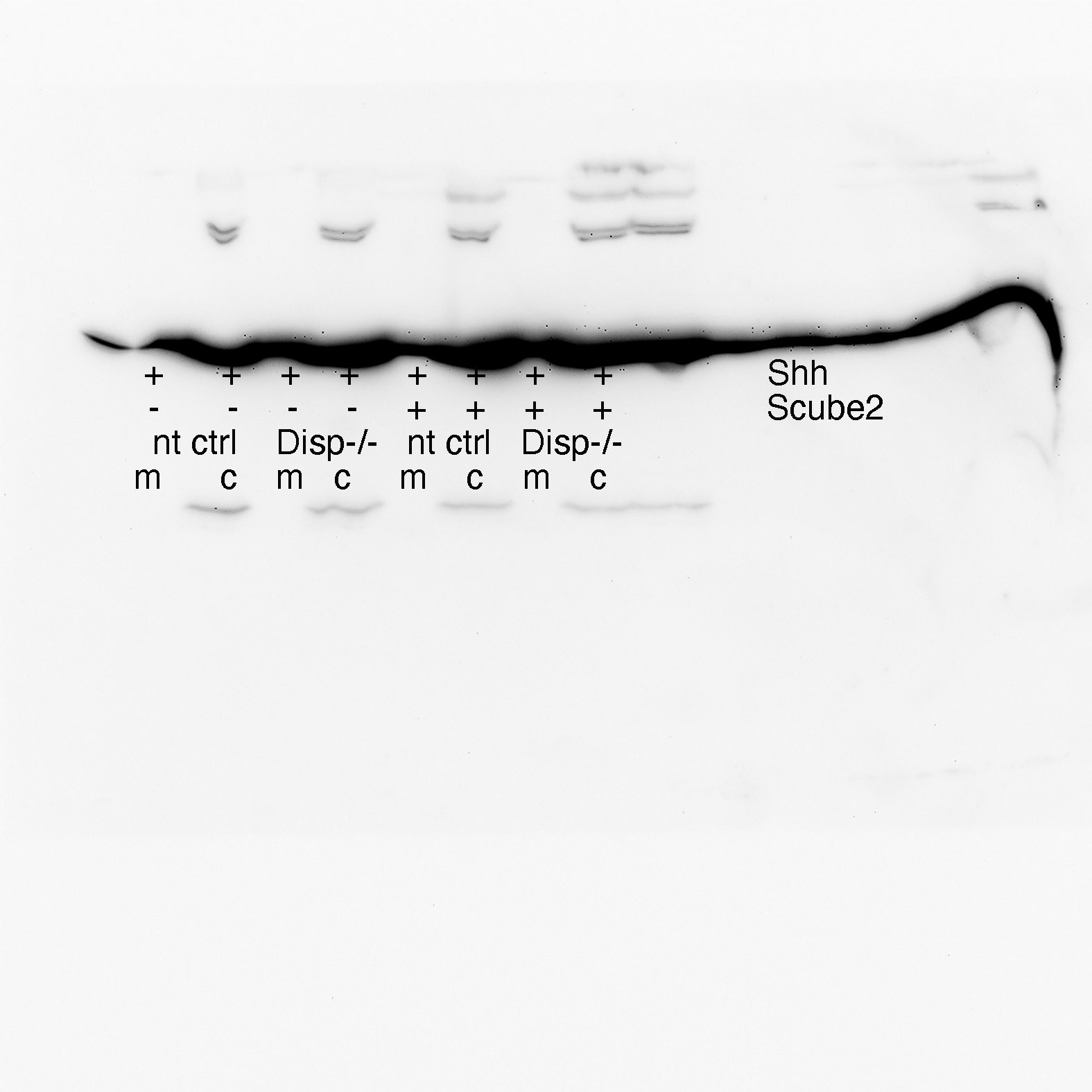

Supplement: Figure 3—figure supplement 1—source data 1. [file elife-86920-fig3-figsupp1-data1.zip › Figure 3-Figure Supplement 1 - Source Data 1/B_V744actin_2_80sec labelled.jpg]

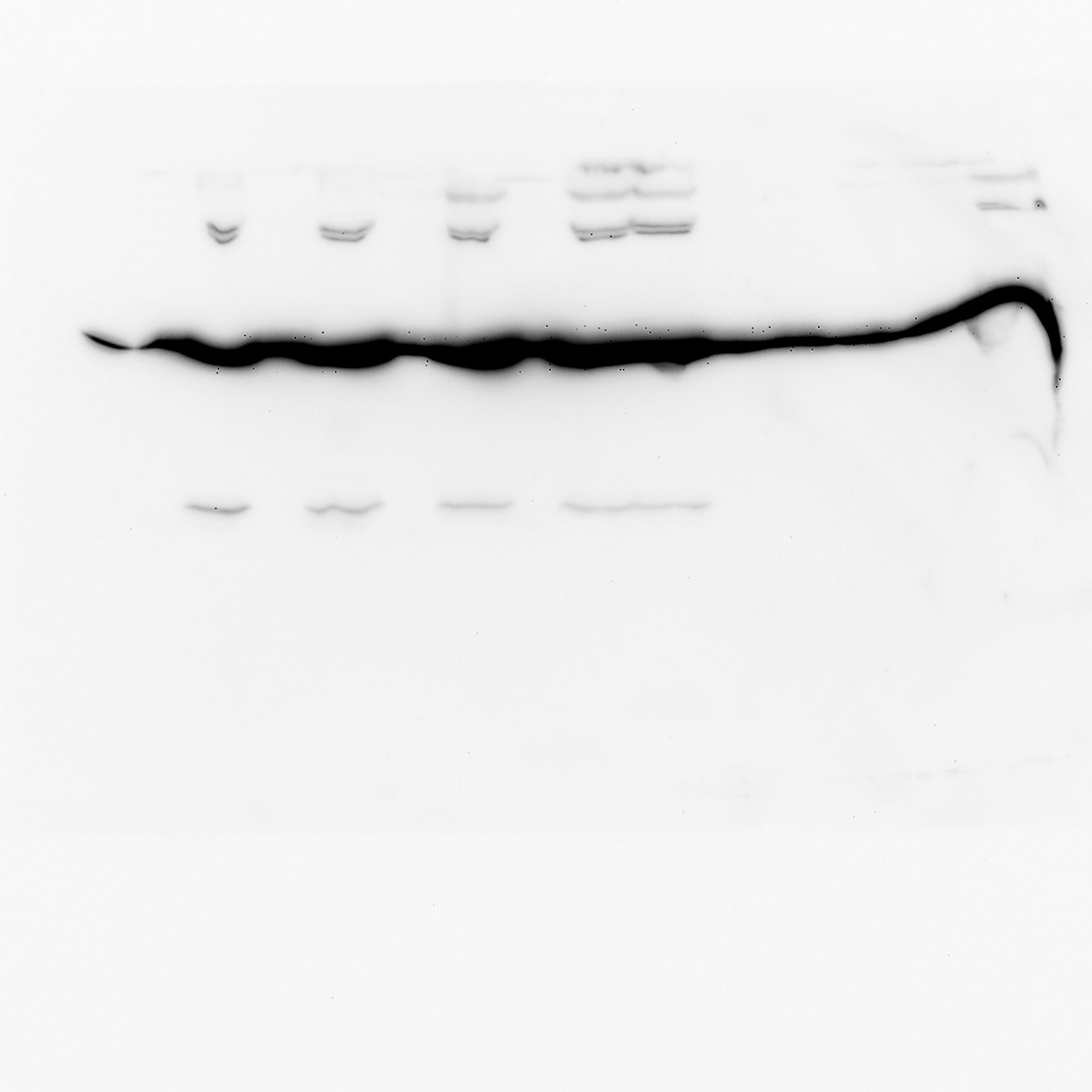

Supplement: Figure 3—figure supplement 1—source data 1. [file elife-86920-fig3-figsupp1-data1.zip › Figure 3-Figure Supplement 1 - Source Data 1/B_V744actin_2_80sec.jpg]

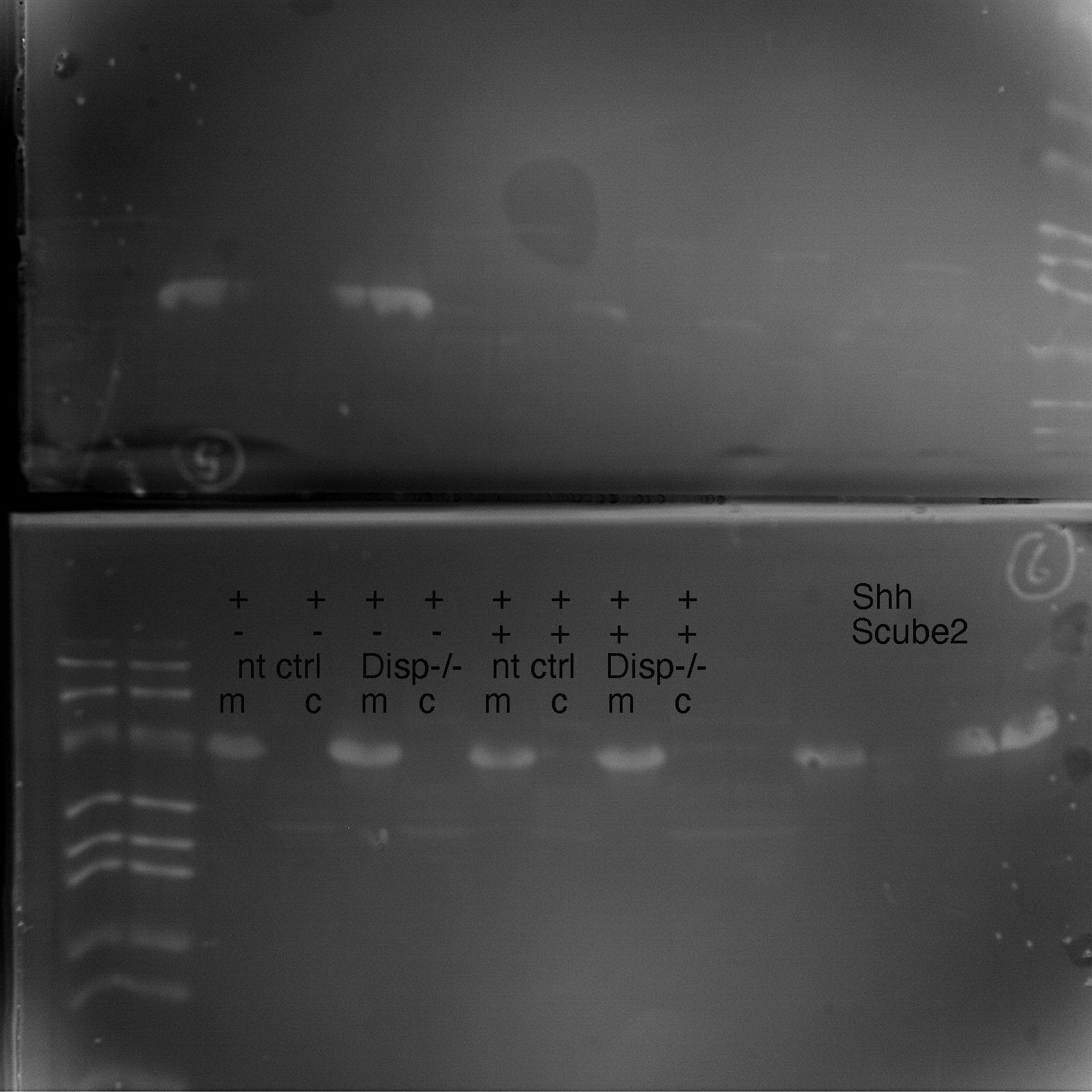

Supplement: Figure 3—figure supplement 1—source data 1. [file elife-86920-fig3-figsupp1-data1.zip › Figure 3-Figure Supplement 1 - Source Data 1/C_V744_5+6_Pon labelled.jpg]

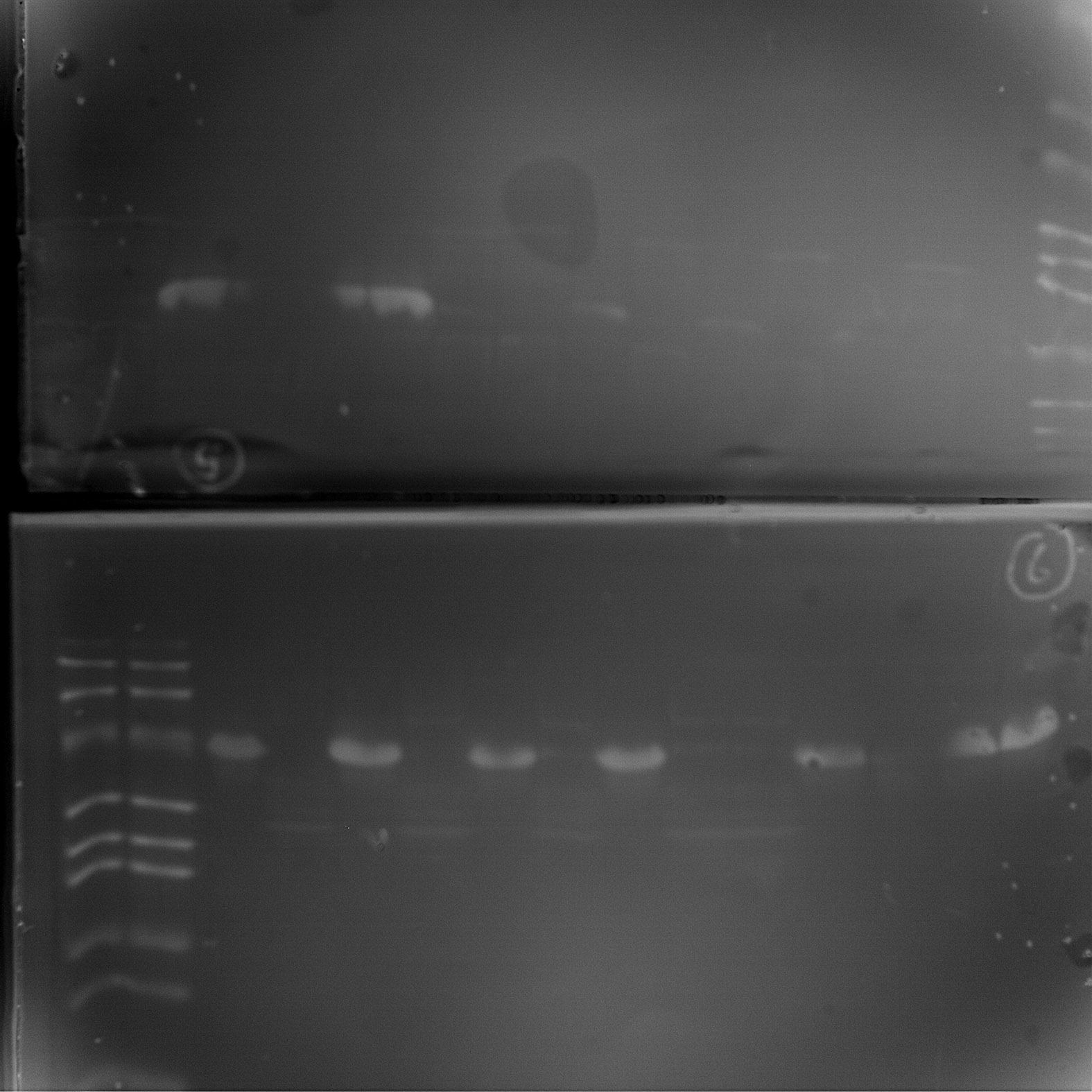

Supplement: Figure 3—figure supplement 1—source data 1. [file elife-86920-fig3-figsupp1-data1.zip › Figure 3-Figure Supplement 1 - Source Data 1/C_V744_5+6_Pon.jpg]

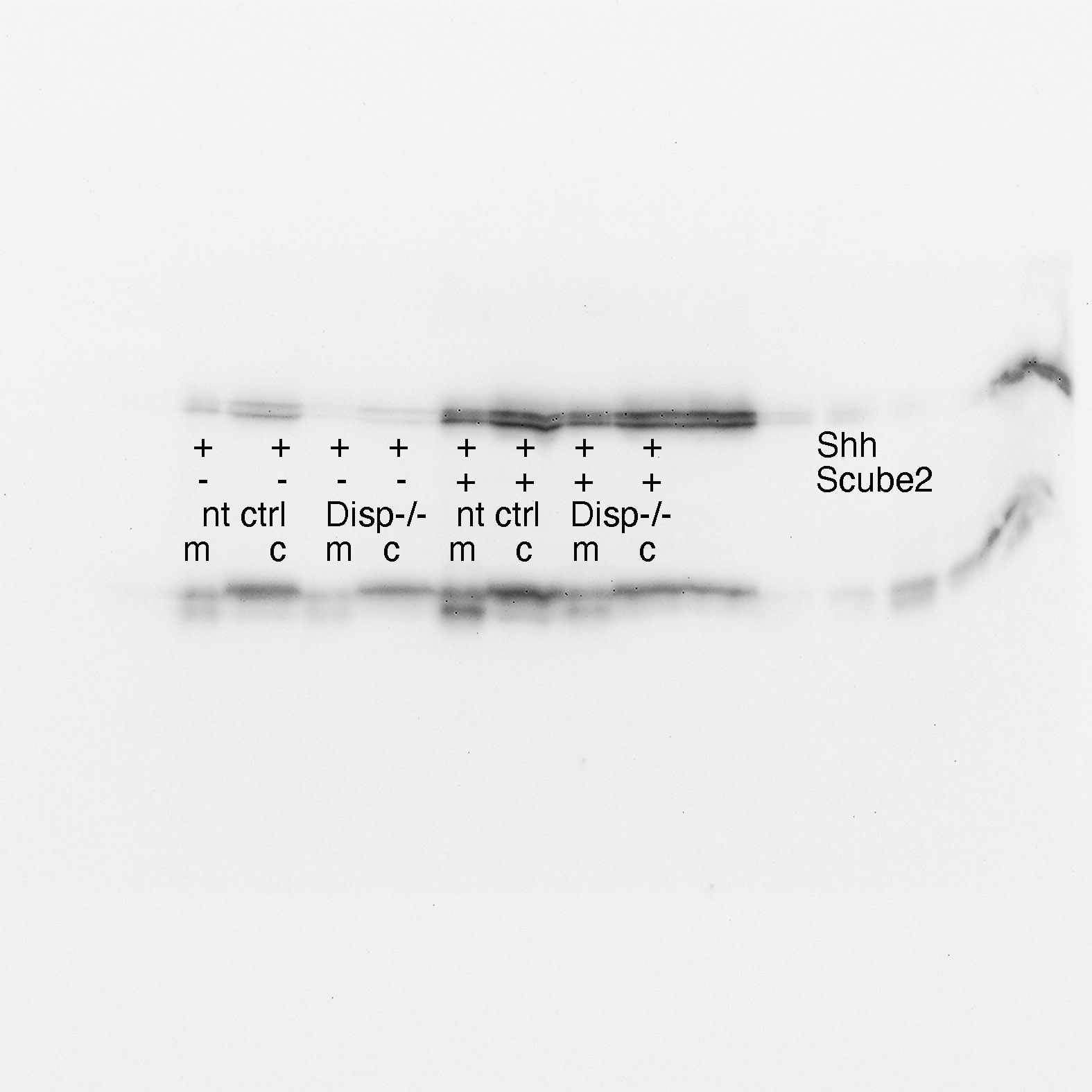

Supplement: Figure 3—figure supplement 1—source data 1. [file elife-86920-fig3-figsupp1-data1.zip › Figure 3-Figure Supplement 1 - Source Data 1/C_V744_6_1min labelled.jpg]

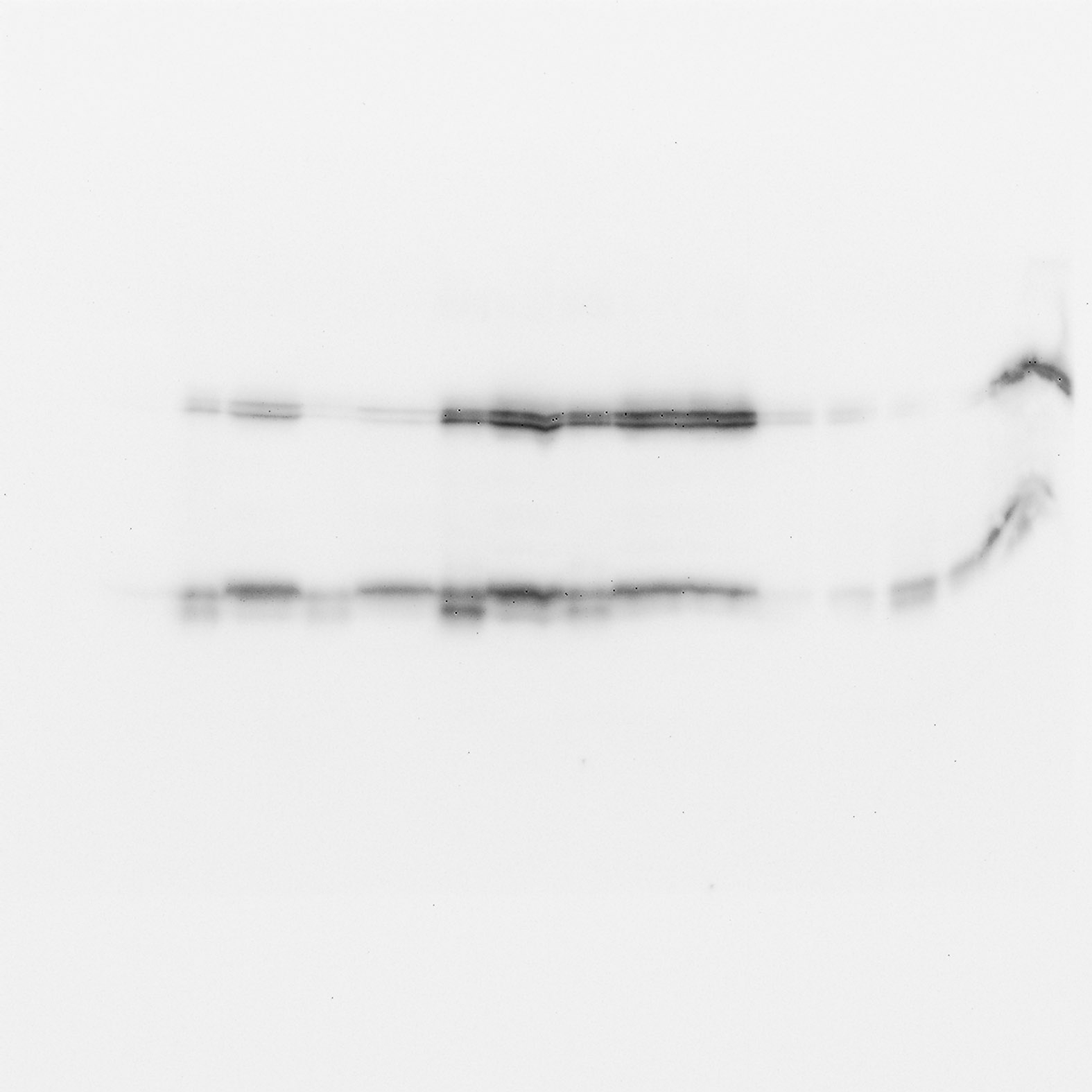

Supplement: Figure 3—figure supplement 1—source data 1. [file elife-86920-fig3-figsupp1-data1.zip › Figure 3-Figure Supplement 1 - Source Data 1/C_V744_6_1min.jpg]

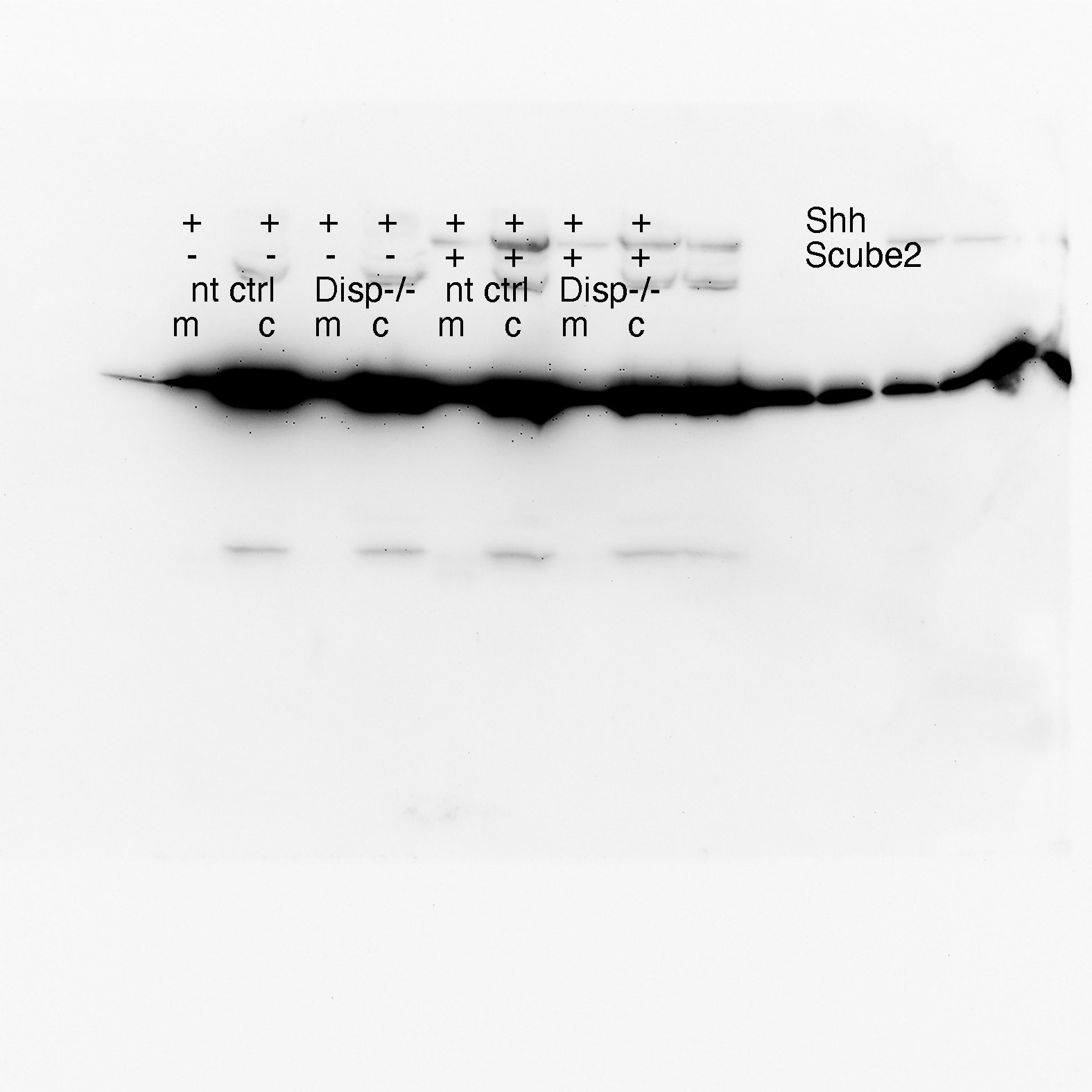

Supplement: Figure 3—figure supplement 1—source data 1. [file elife-86920-fig3-figsupp1-data1.zip › Figure 3-Figure Supplement 1 - Source Data 1/C_V744actin_6_80sec labelled.jpg]

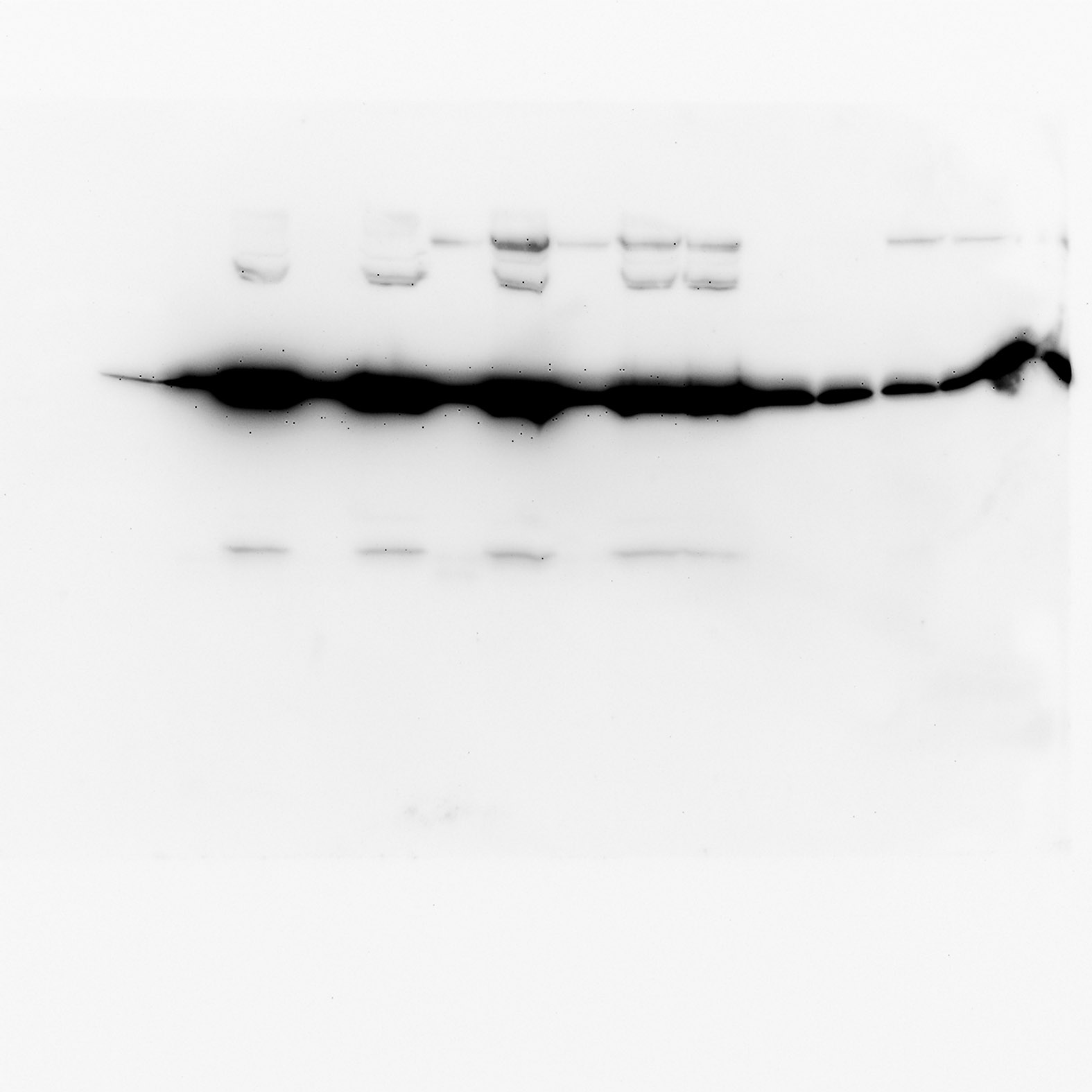

Supplement: Figure 3—figure supplement 1—source data 1. [file elife-86920-fig3-figsupp1-data1.zip › Figure 3-Figure Supplement 1 - Source Data 1/C_V744actin_6_80sec.jpg]

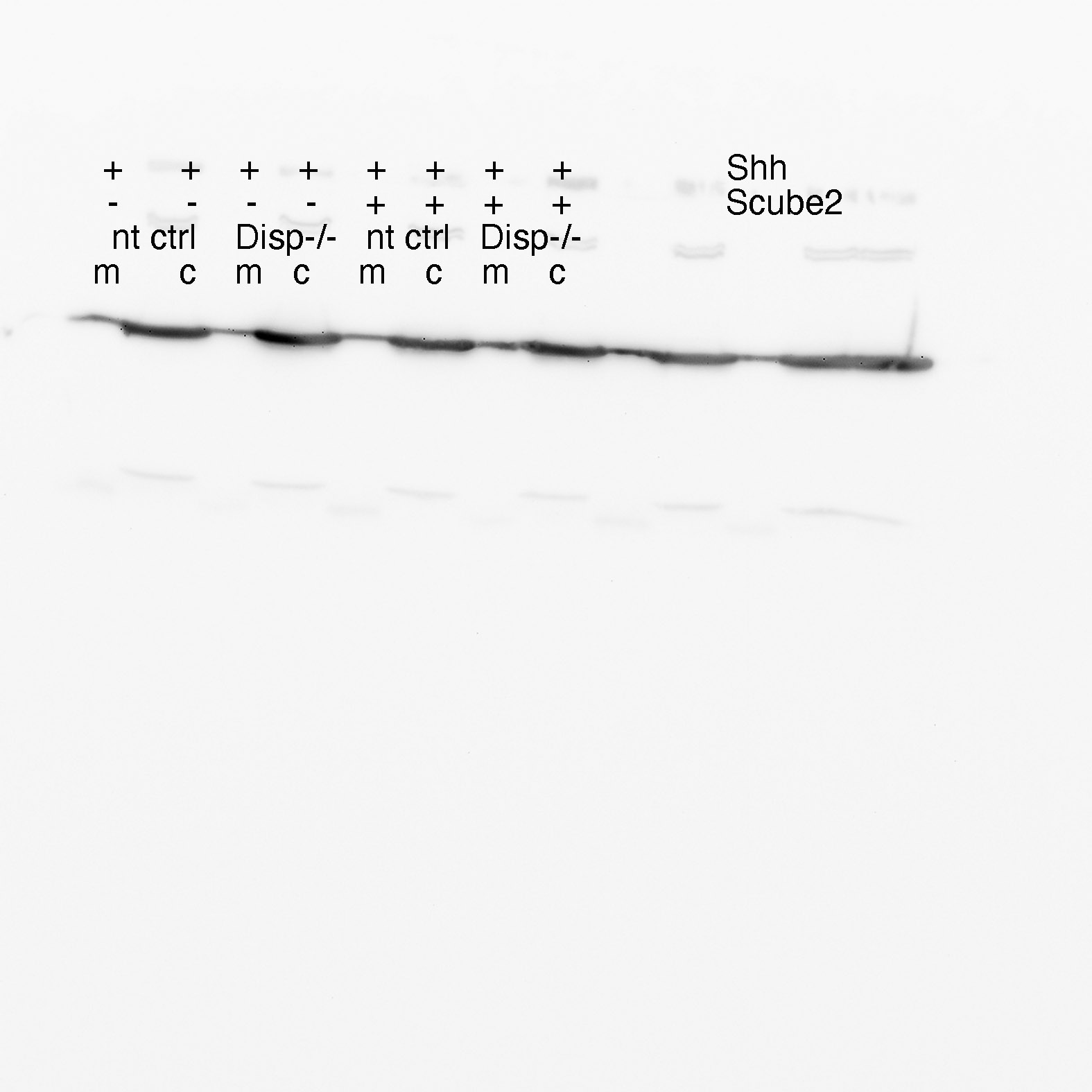

Supplement: Figure 3—figure supplement 1—source data 1. [file elife-86920-fig3-figsupp1-data1.zip › Figure 3-Figure Supplement 1 - Source Data 1/D_V741_1 actin_1 labelled.jpg]

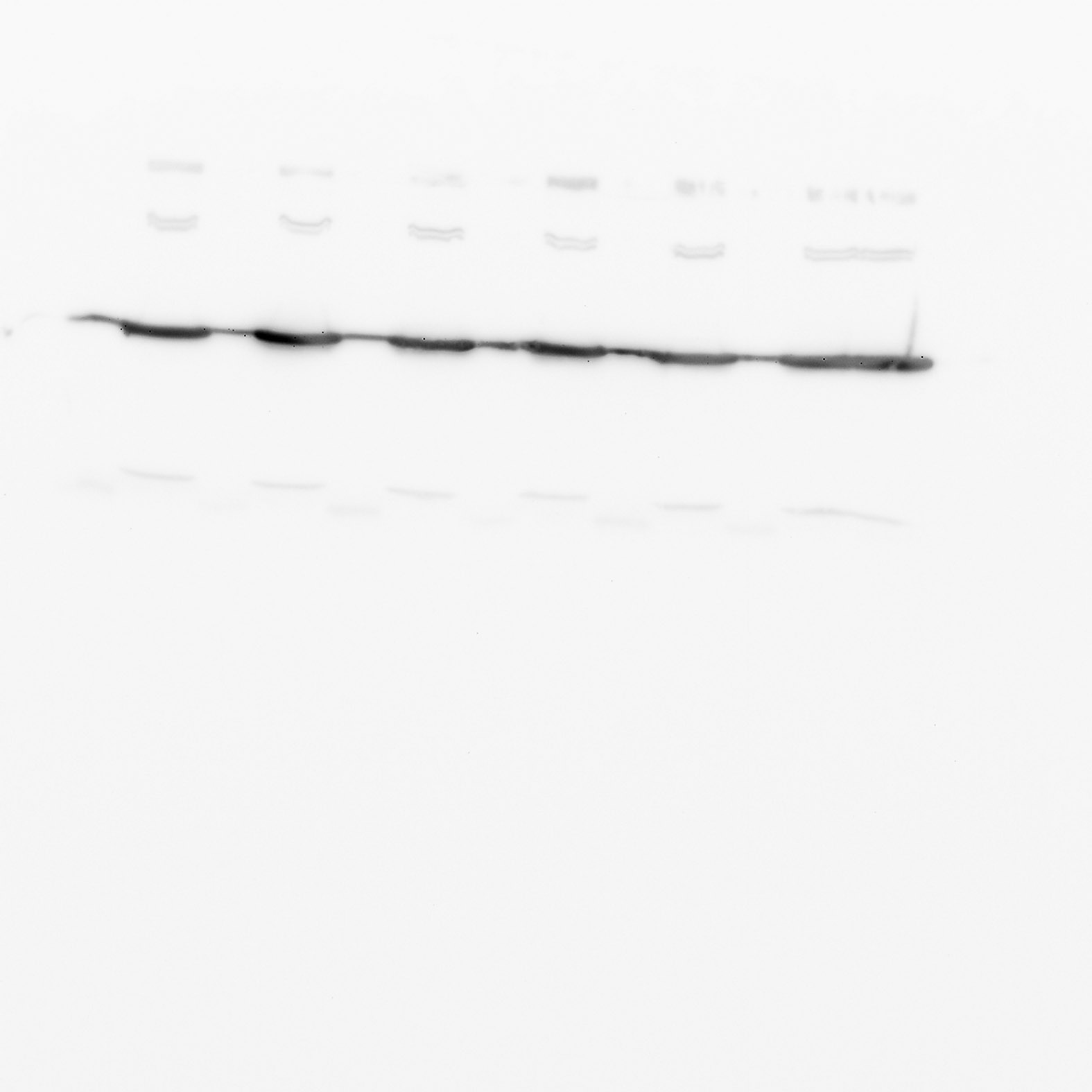

Supplement: Figure 3—figure supplement 1—source data 1. [file elife-86920-fig3-figsupp1-data1.zip › Figure 3-Figure Supplement 1 - Source Data 1/D_V741_1 actin_1.jpg]

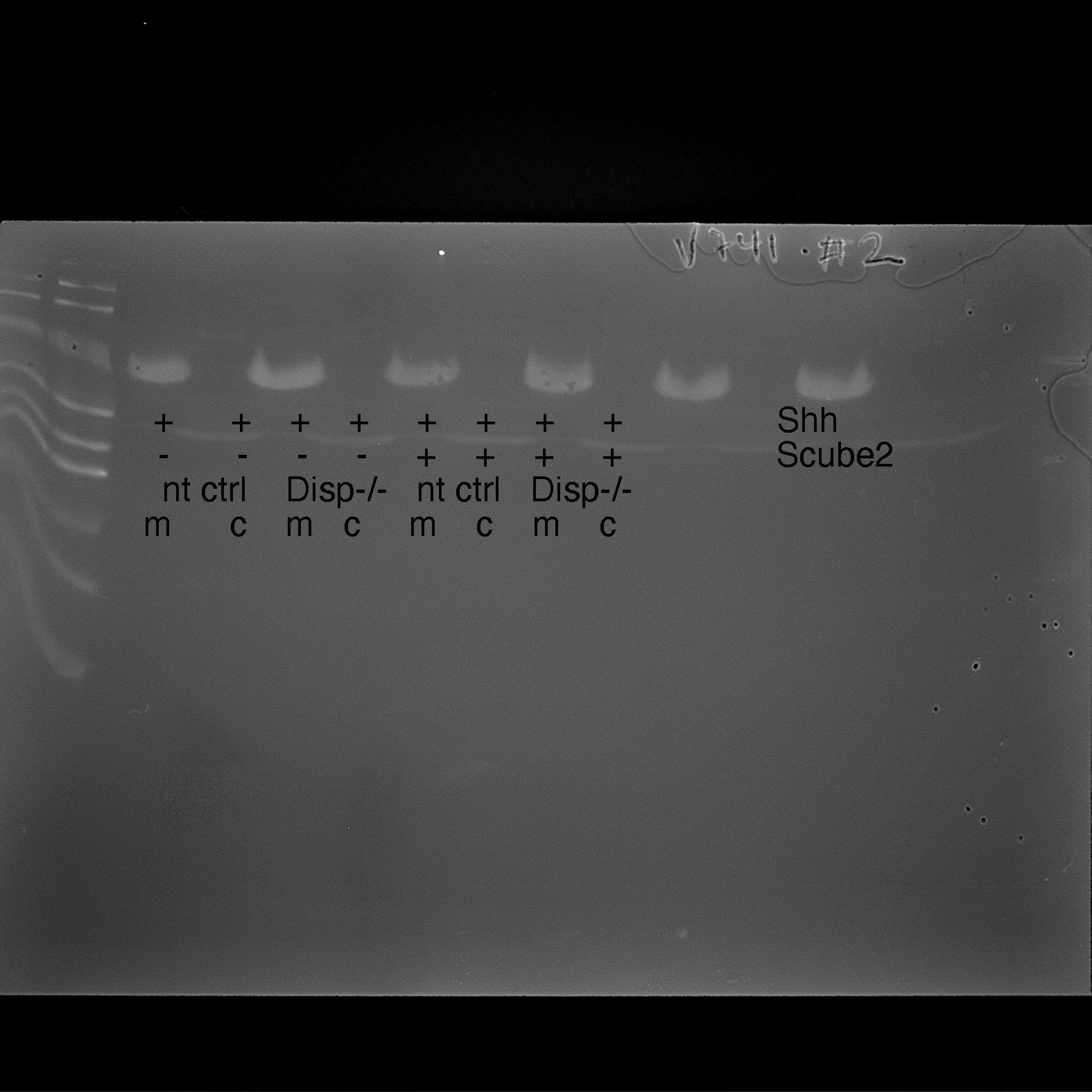

Supplement: Figure 3—figure supplement 1—source data 1. [file elife-86920-fig3-figsupp1-data1.zip › Figure 3-Figure Supplement 1 - Source Data 1/D_V741_Pon_2_1 labelled.jpg]

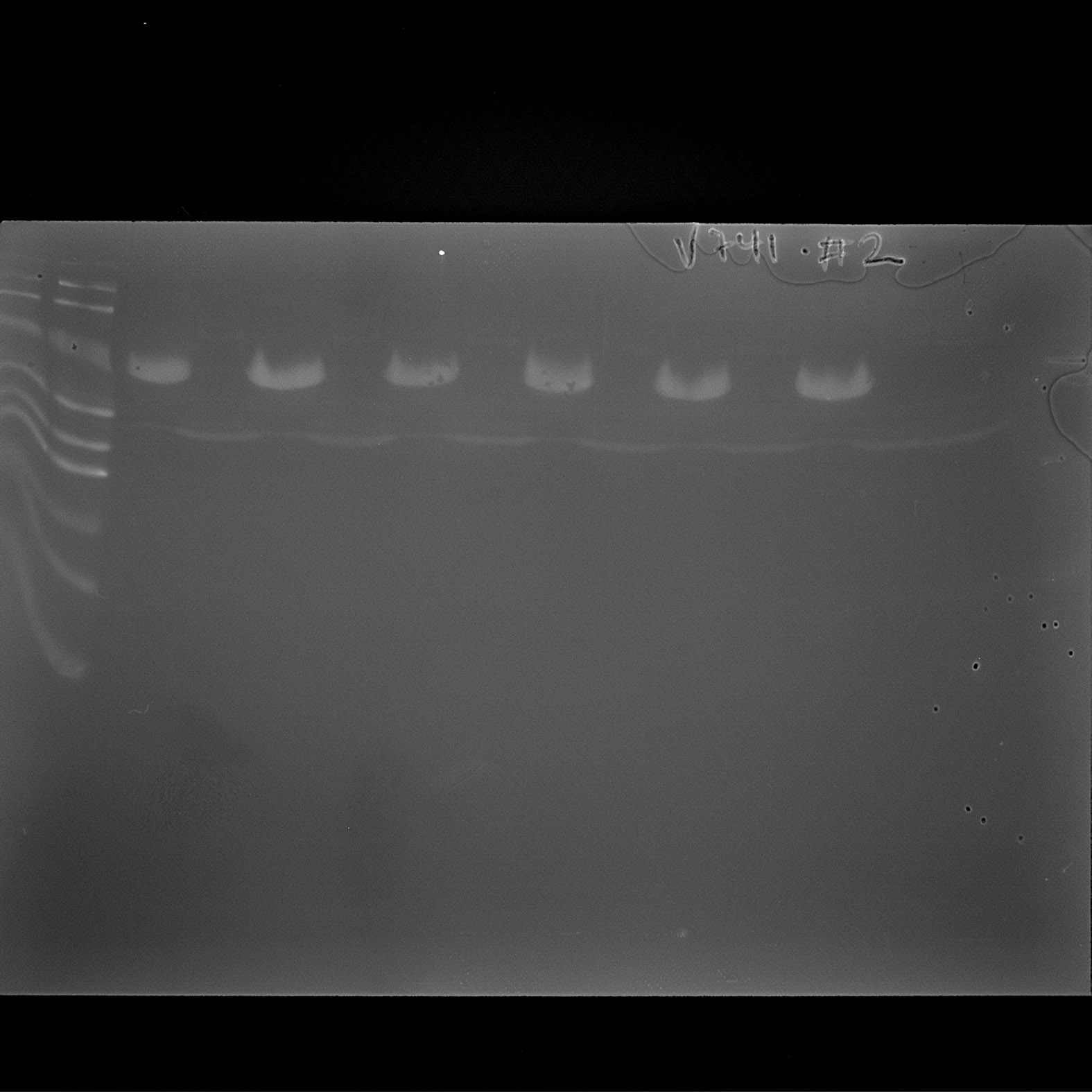

Supplement: Figure 3—figure supplement 1—source data 1. [file elife-86920-fig3-figsupp1-data1.zip › Figure 3-Figure Supplement 1 - Source Data 1/D_V741_Pon_2_1.jpg]

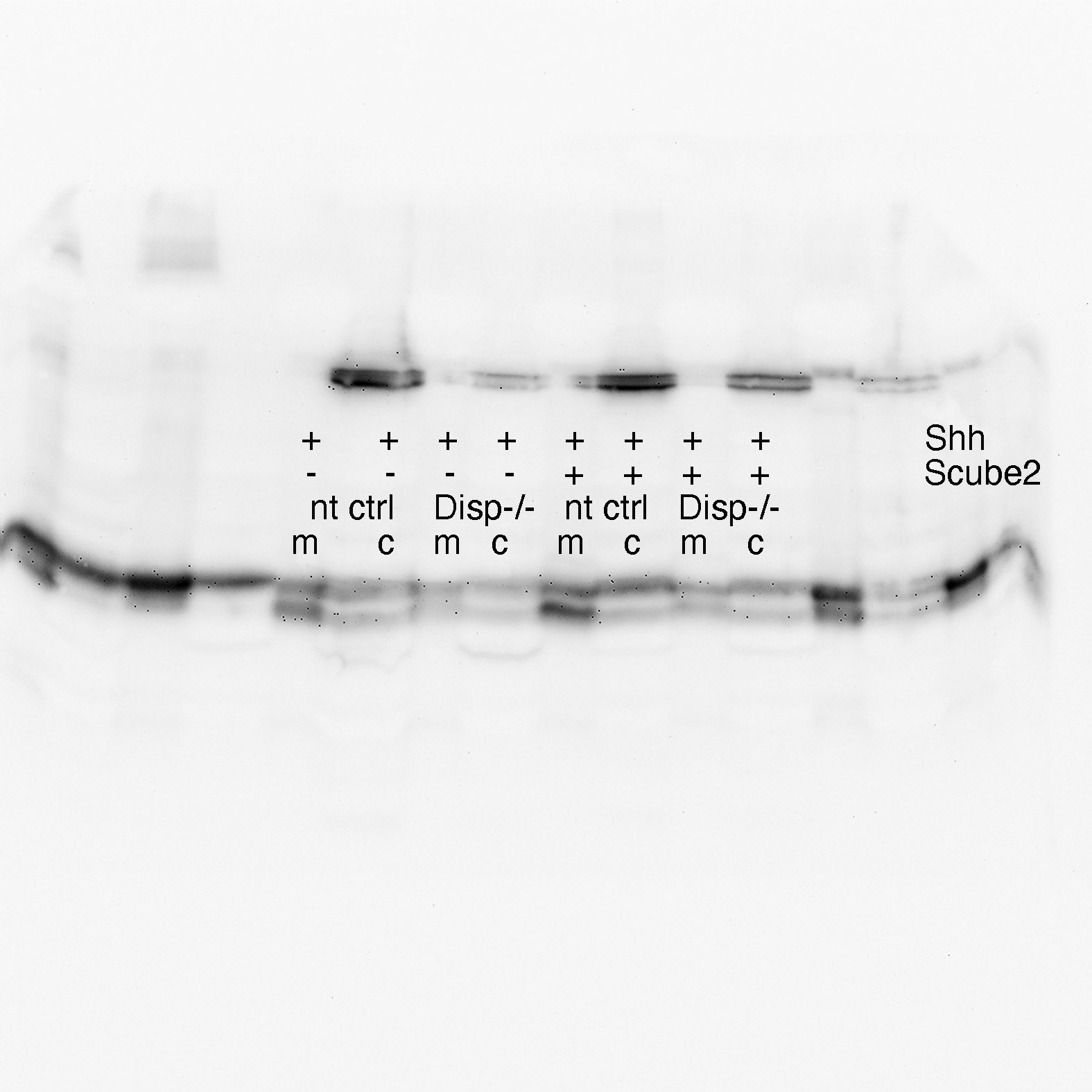

Supplement: Figure 3—figure supplement 1—source data 1. [file elife-86920-fig3-figsupp1-data1.zip › Figure 3-Figure Supplement 1 - Source Data 1/D_V742serum_5_90sec labelled.jpg]

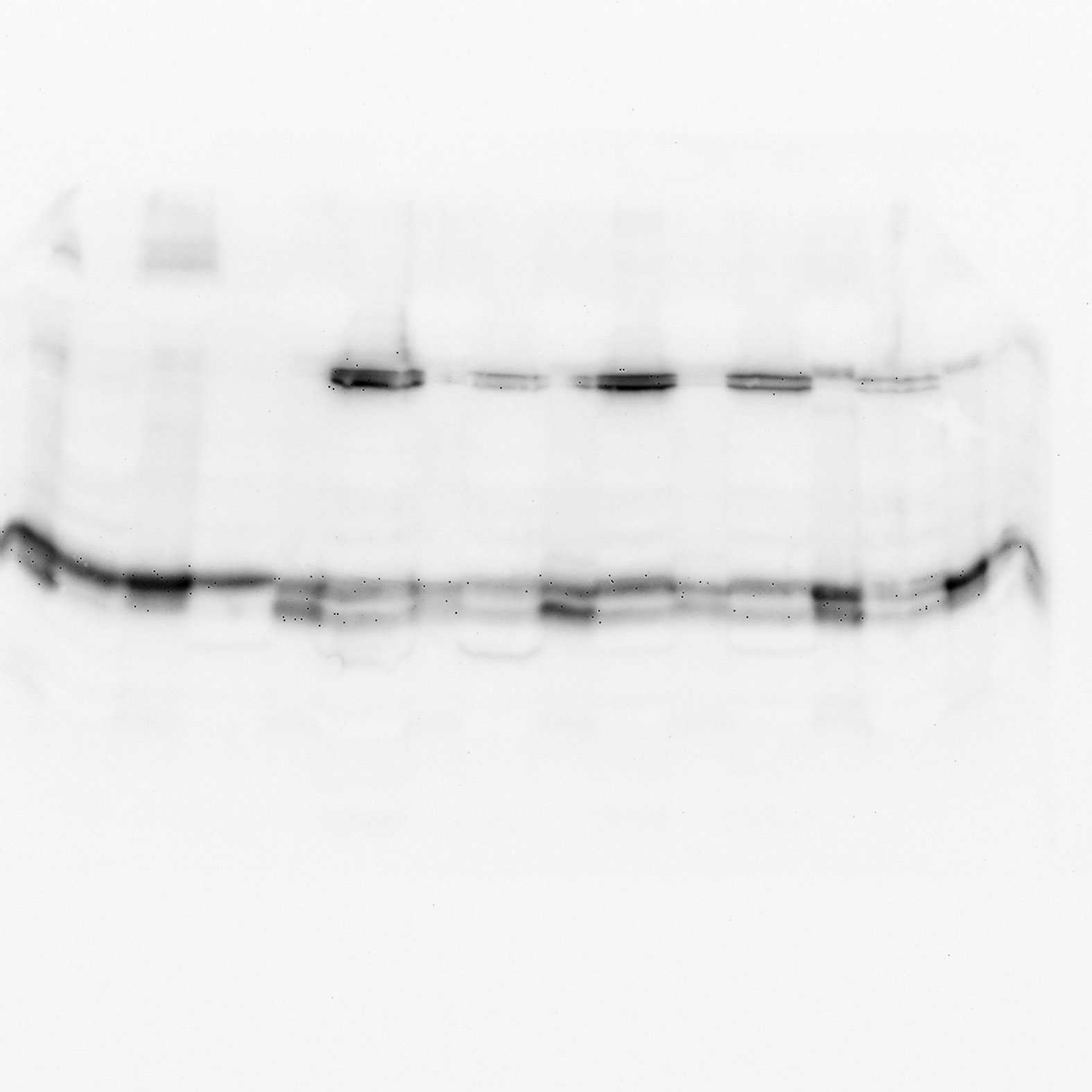

Supplement: Figure 3—figure supplement 1—source data 1. [file elife-86920-fig3-figsupp1-data1.zip › Figure 3-Figure Supplement 1 - Source Data 1/D_V742serum_5_90sec.jpg]

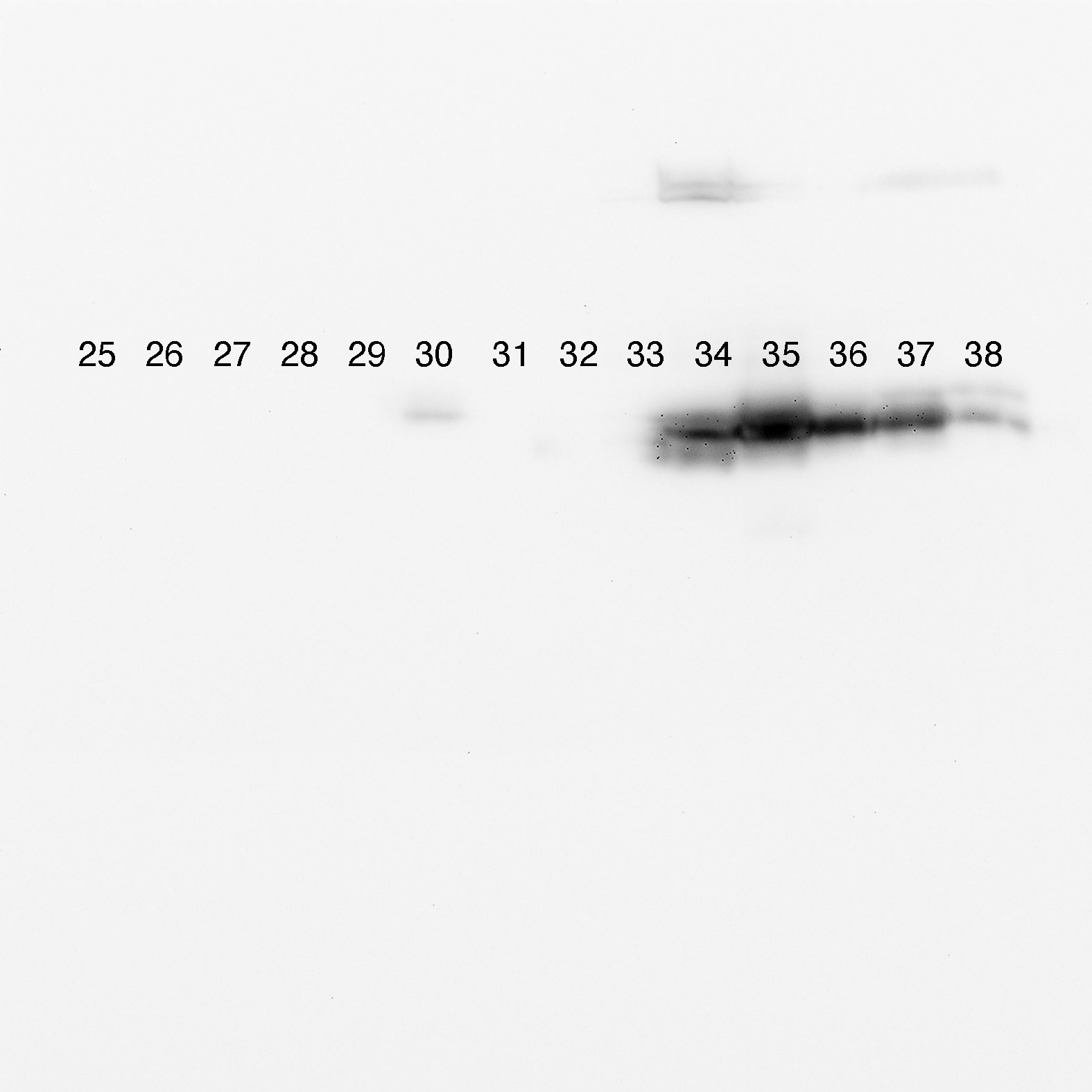

Supplement: Figure 3—figure supplement 2—source data 1. [file elife-86920-fig3-figsupp2-data1.zip › Figure 3-Figure Supplement 2 - Source Data 1/A_27-09-21_16Bit_17C_antiShh Rabbit_37sec labelled.jpg]

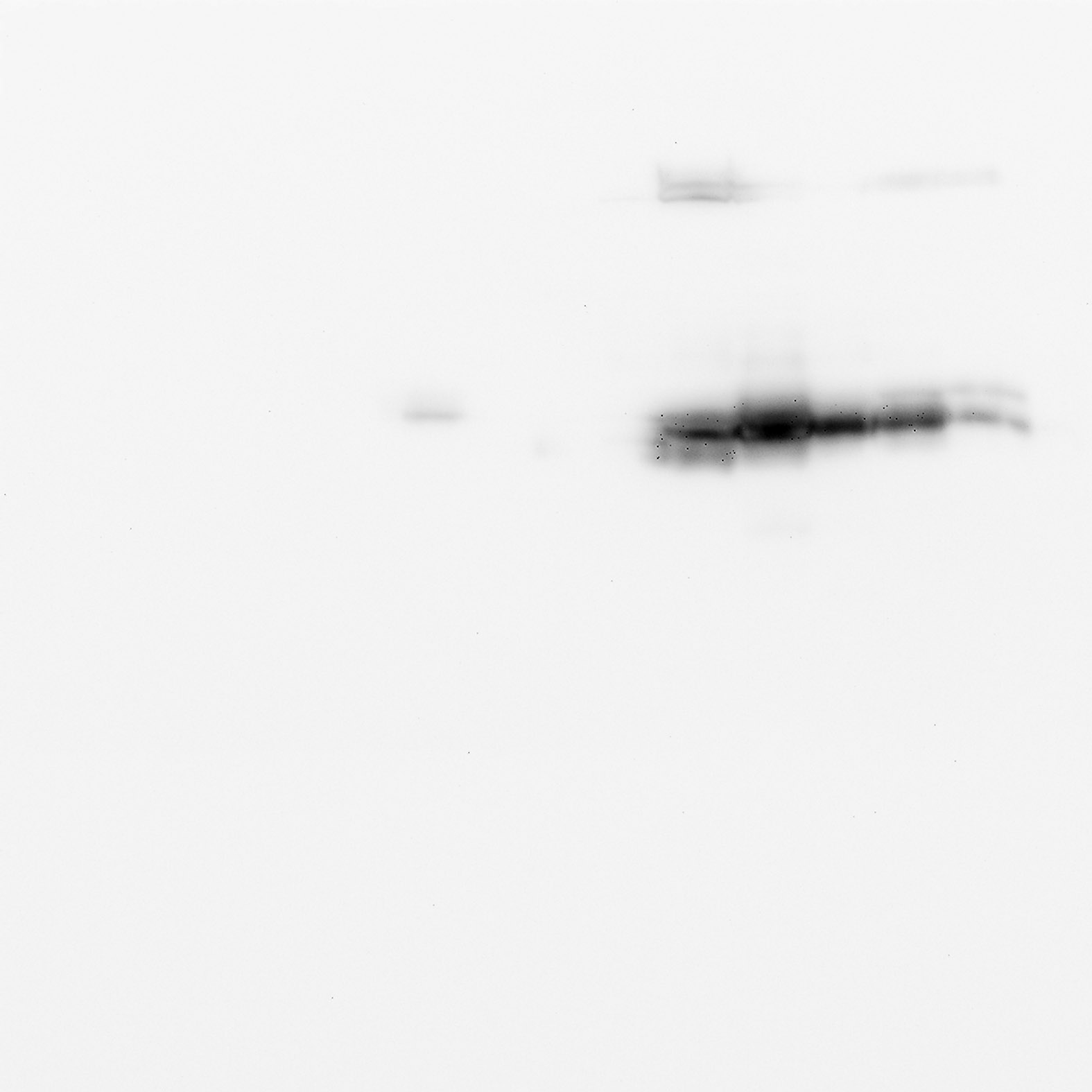

Supplement: Figure 3—figure supplement 2—source data 1. [file elife-86920-fig3-figsupp2-data1.zip › Figure 3-Figure Supplement 2 - Source Data 1/A_27-09-21_16Bit_17C_antiShh Rabbit_37sec.jpg]
