# Supplementary figures and images for: Two-way Dispatched function in Sonic hedgehog shedding and transfer to high-density lipoproteins (part 3 of 3)
Source: eLife. 2024 Sep 19;12:RP86920. doi: 10.7554/eLife.86920 (PMC11412720; doi:10.7554/eLife.86920)

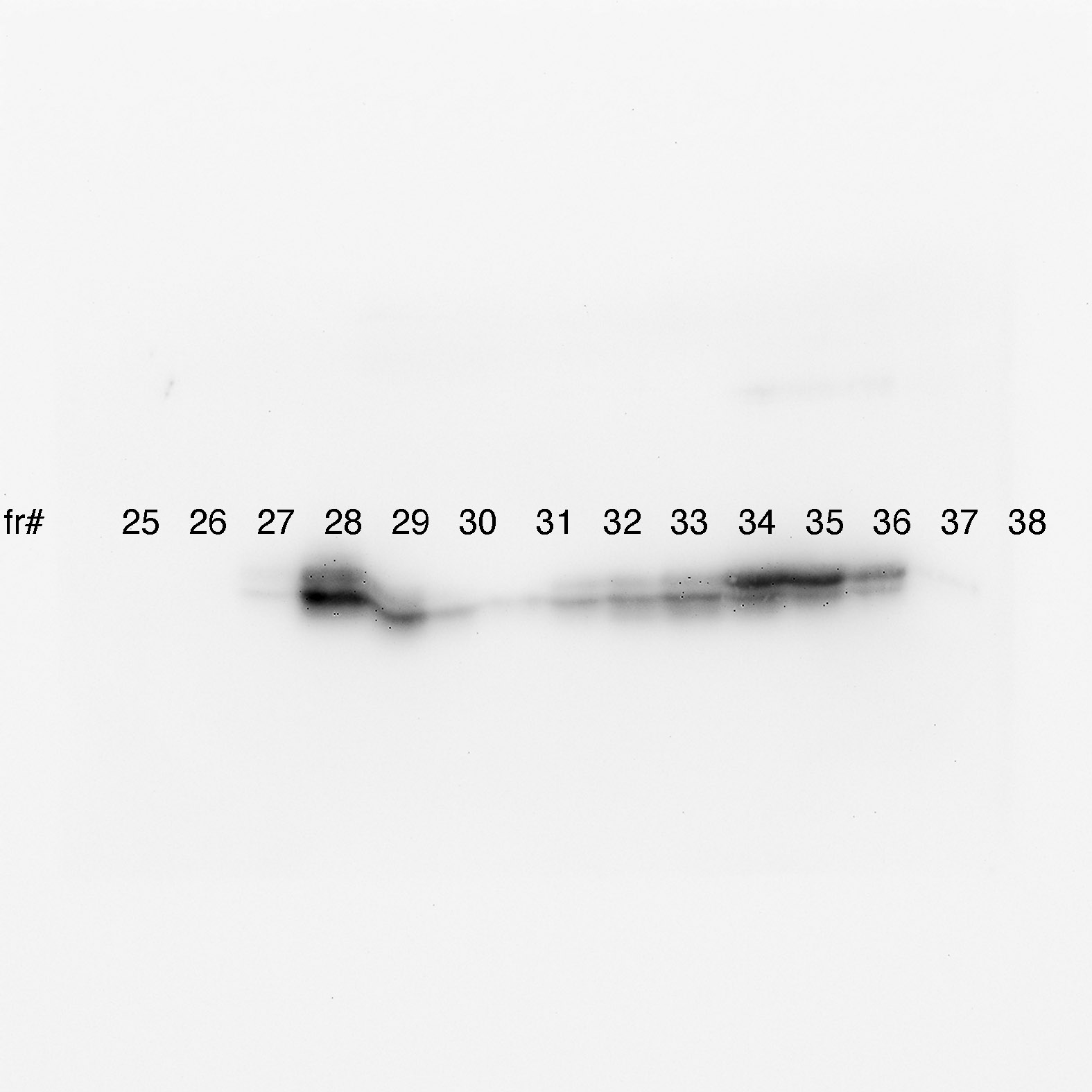

Supplement: Figure 3—figure supplement 2—source data 1. [file elife-86920-fig3-figsupp2-data1.zip › Figure 3-Figure Supplement 2 - Source Data 1/B_20210825_VK187 Gel 2 A media UZ Fr25-38_Shh-rabbit_16bit_53sec labelled.jpg]

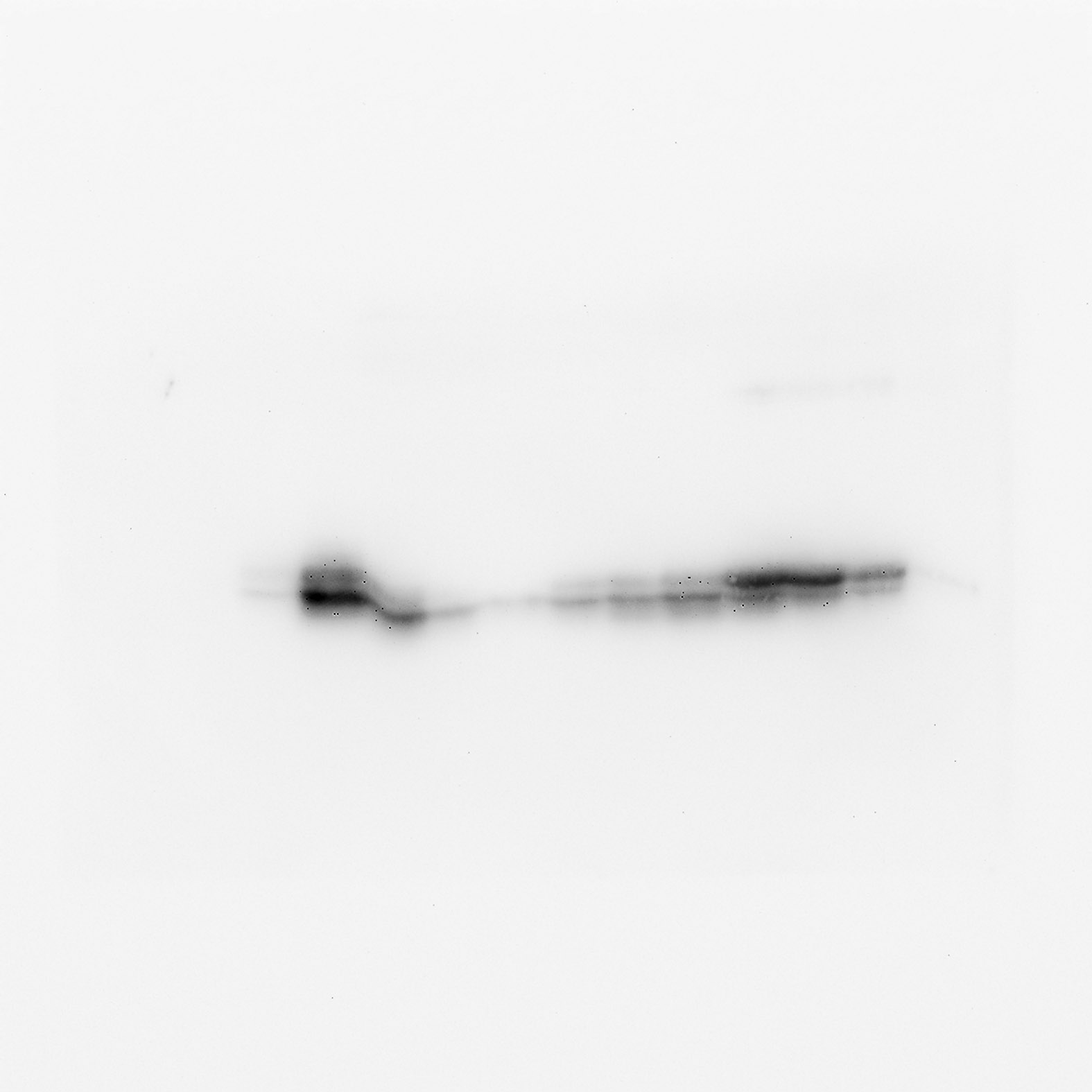

Supplement: Figure 3—figure supplement 2—source data 1. [file elife-86920-fig3-figsupp2-data1.zip › Figure 3-Figure Supplement 2 - Source Data 1/B_20210825_VK187 Gel 2 A media UZ Fr25-38_Shh-rabbit_16bit_53sec.jpg]

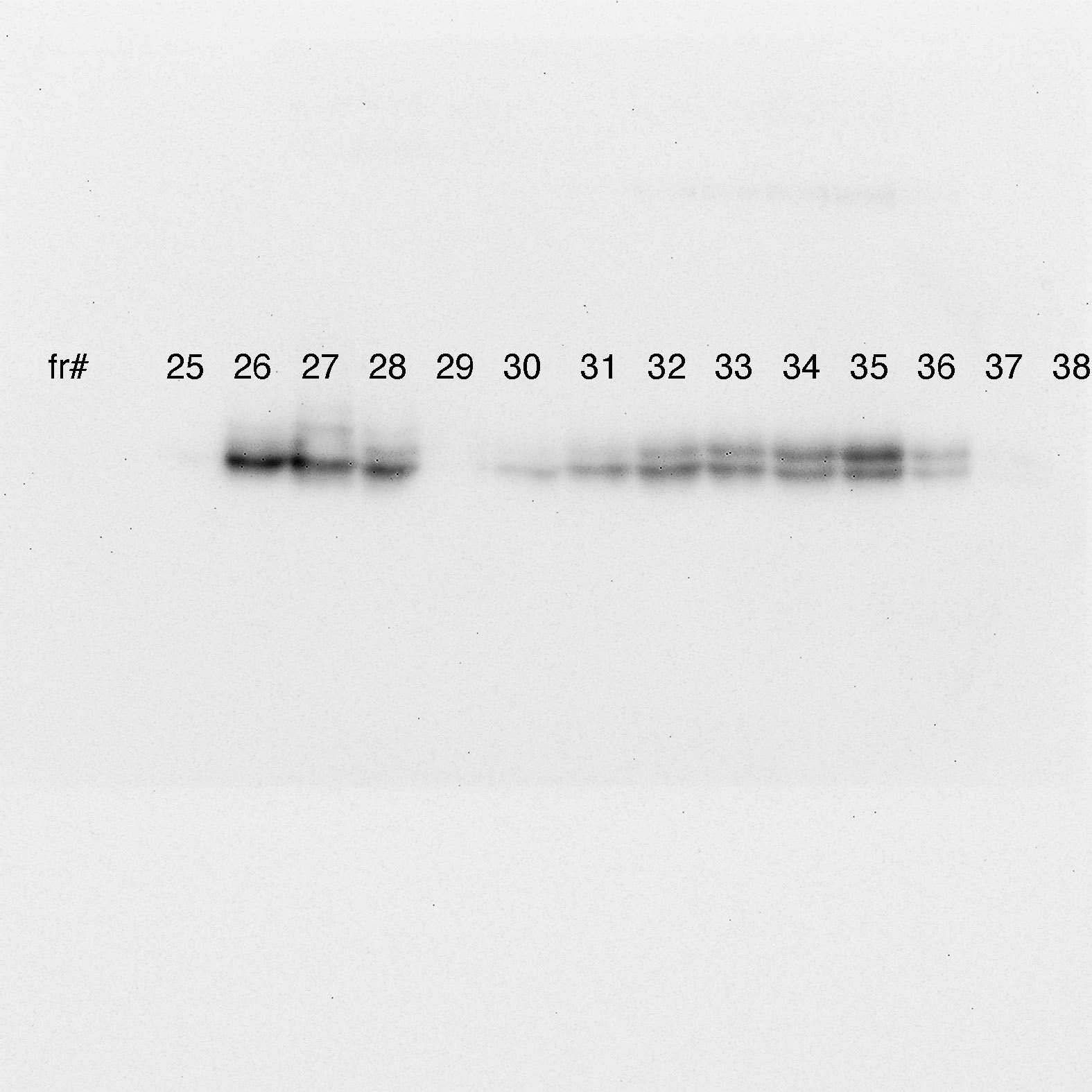

Supplement: Figure 3—figure supplement 2—source data 1. [file elife-86920-fig3-figsupp2-data1.zip › Figure 3-Figure Supplement 2 - Source Data 1/C_30-11-2021_16Bit_HPLC_Pr 5_antiShh Rabbit_2min47sec labelled.jpg]

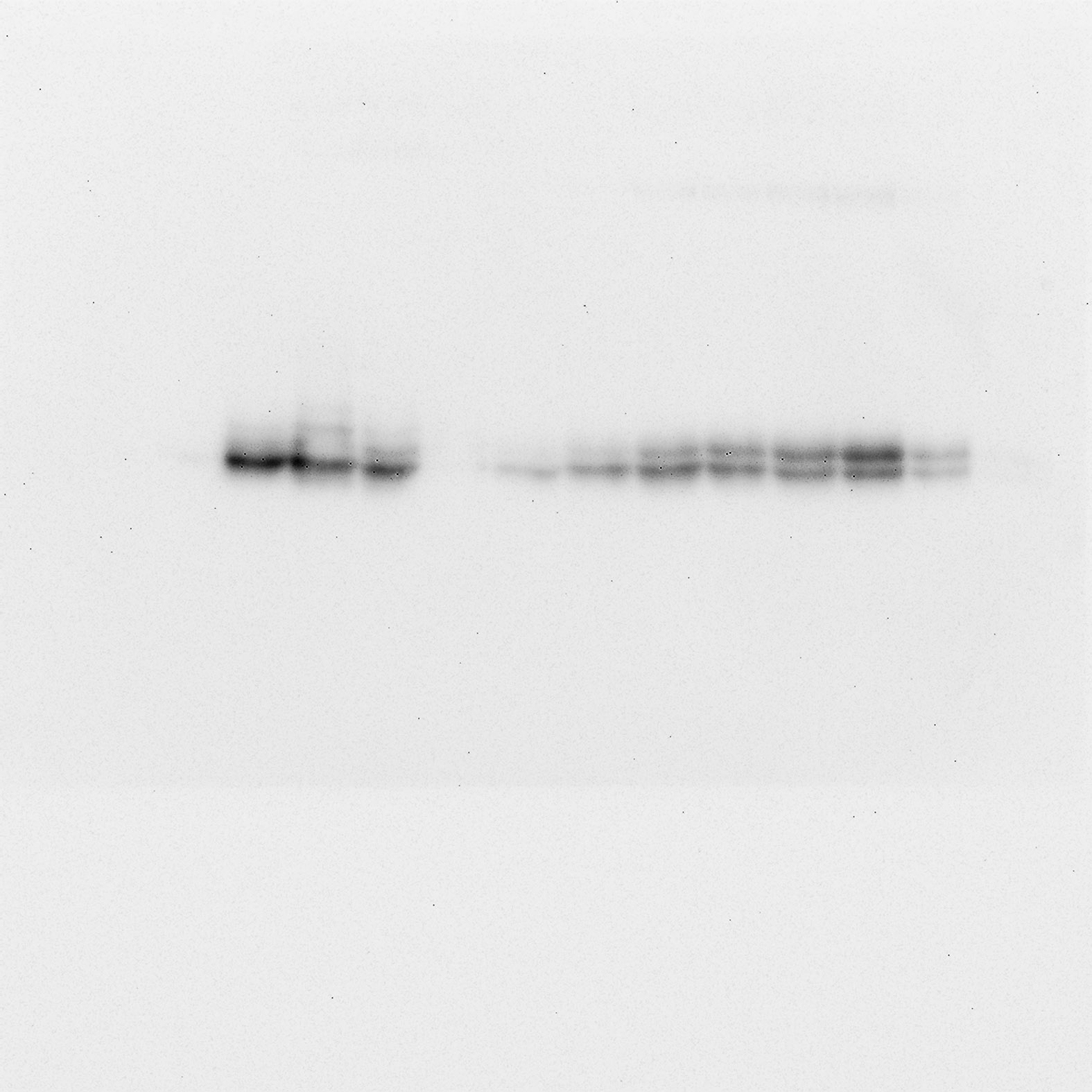

Supplement: Figure 3—figure supplement 2—source data 1. [file elife-86920-fig3-figsupp2-data1.zip › Figure 3-Figure Supplement 2 - Source Data 1/C_30-11-2021_16Bit_HPLC_Pr 5_antiShh Rabbit_2min47sec.jpg]

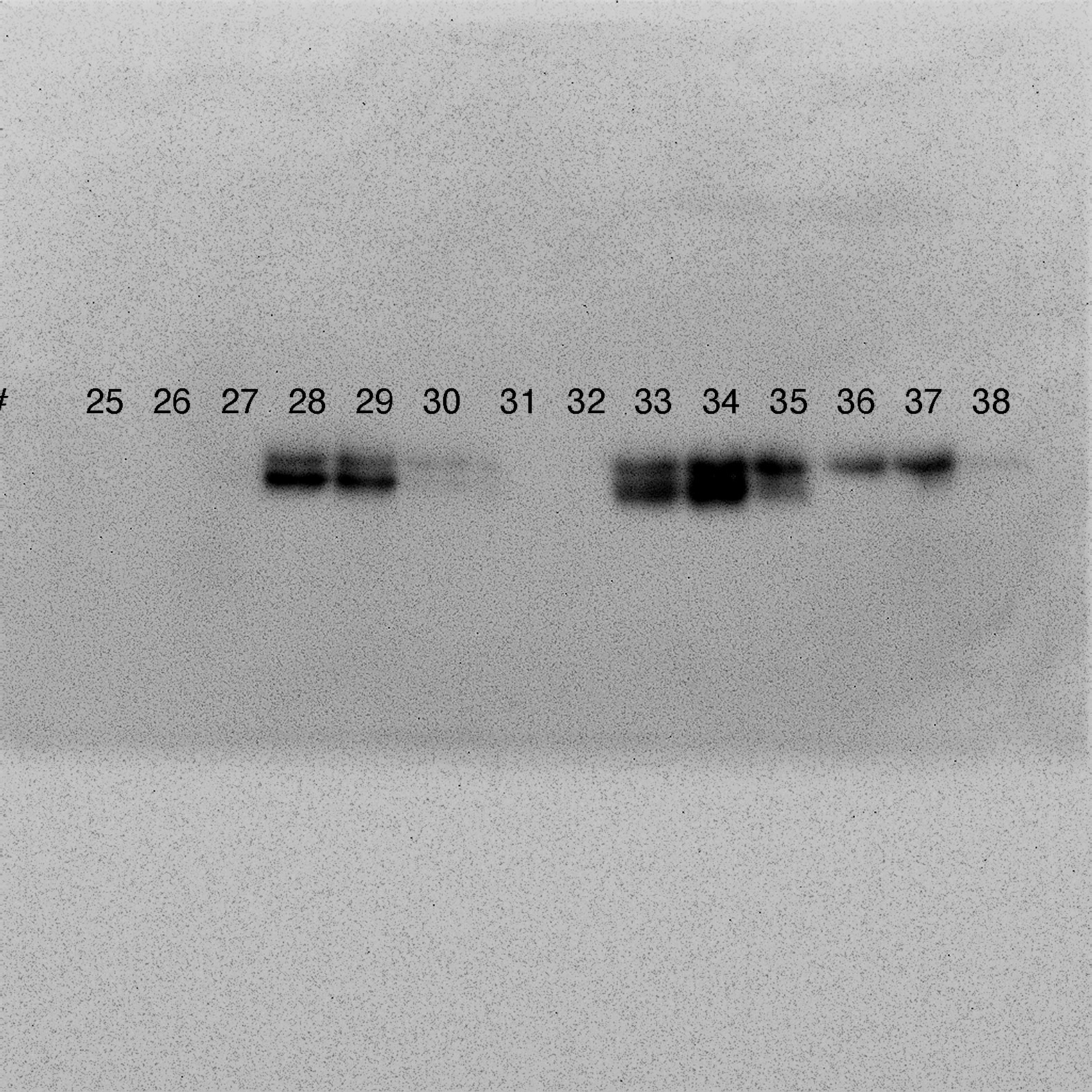

Supplement: Figure 3—figure supplement 2—source data 1. [file elife-86920-fig3-figsupp2-data1.zip › Figure 3-Figure Supplement 2 - Source Data 1/D_24-09-21_16 Bit_Gel 12_anti Shh Rabbit_2min36sec labelled.jpg]

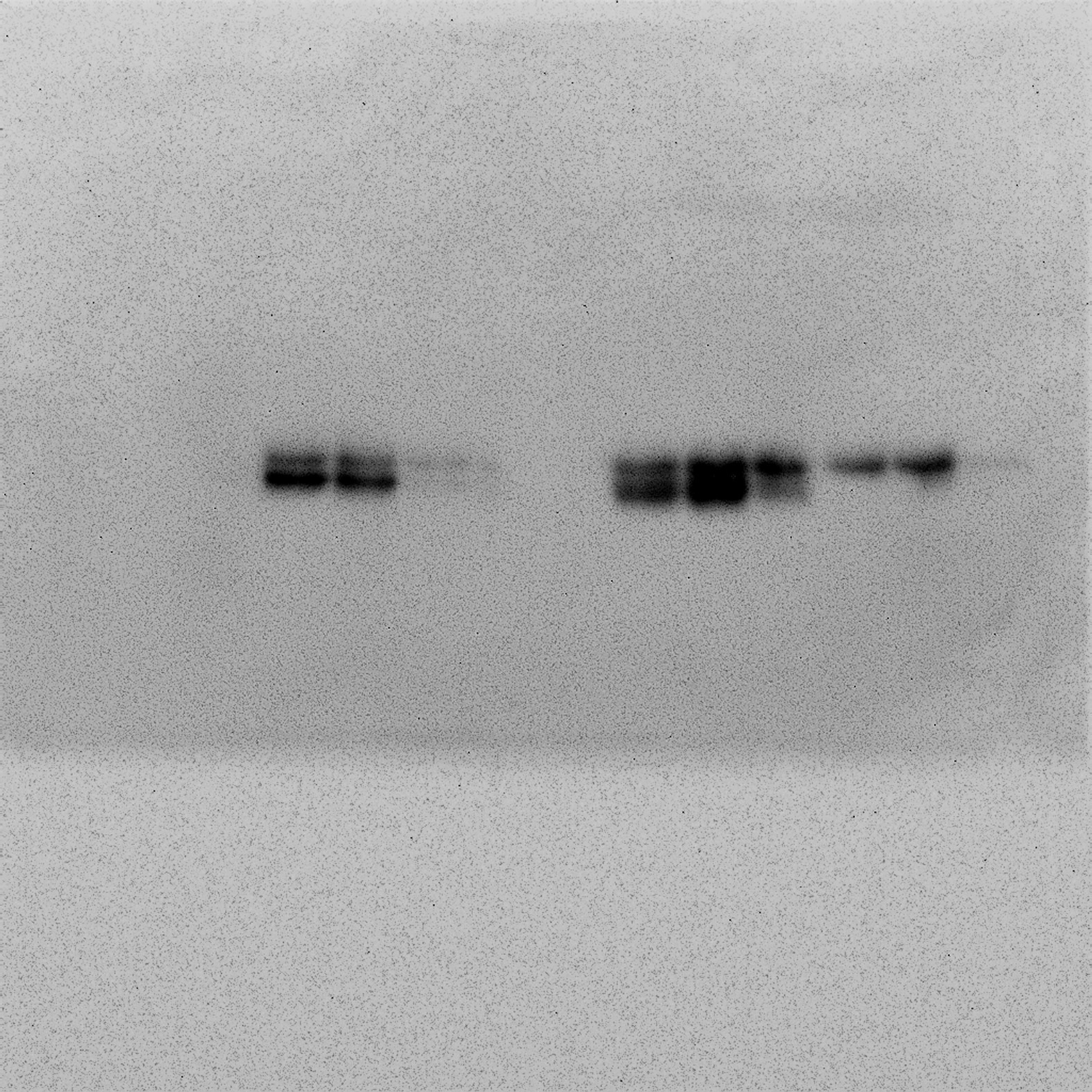

Supplement: Figure 3—figure supplement 2—source data 1. [file elife-86920-fig3-figsupp2-data1.zip › Figure 3-Figure Supplement 2 - Source Data 1/D_24-09-21_16 Bit_Gel 12_anti Shh Rabbit_2min36sec.jpg]

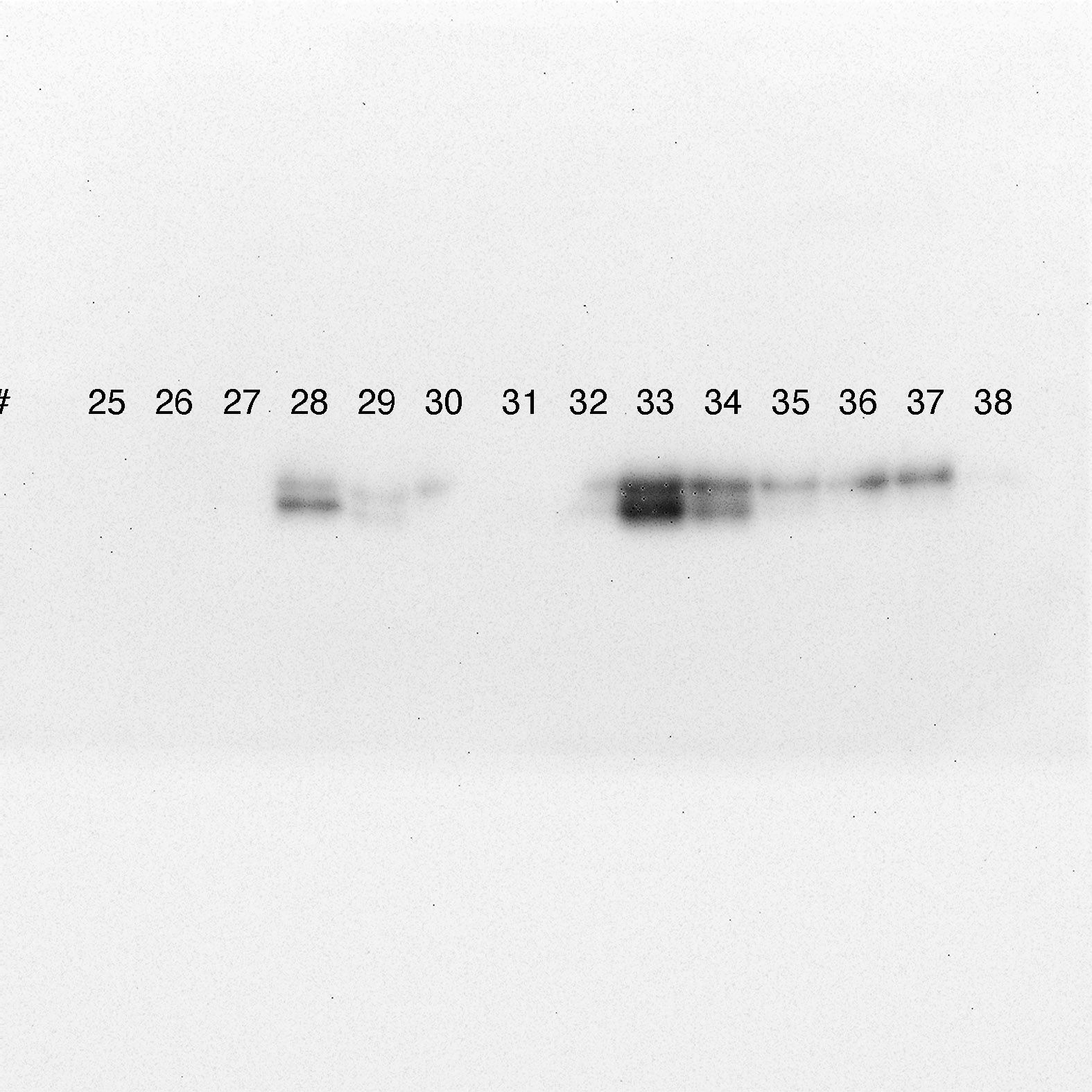

Supplement: Figure 3—figure supplement 2—source data 1. [file elife-86920-fig3-figsupp2-data1.zip › Figure 3-Figure Supplement 2 - Source Data 1/E_24-09-21_16 Bit_Gel 13_anti Shh Rabbit_2min35sec_Tonwertkorrektur labelled.jpg]

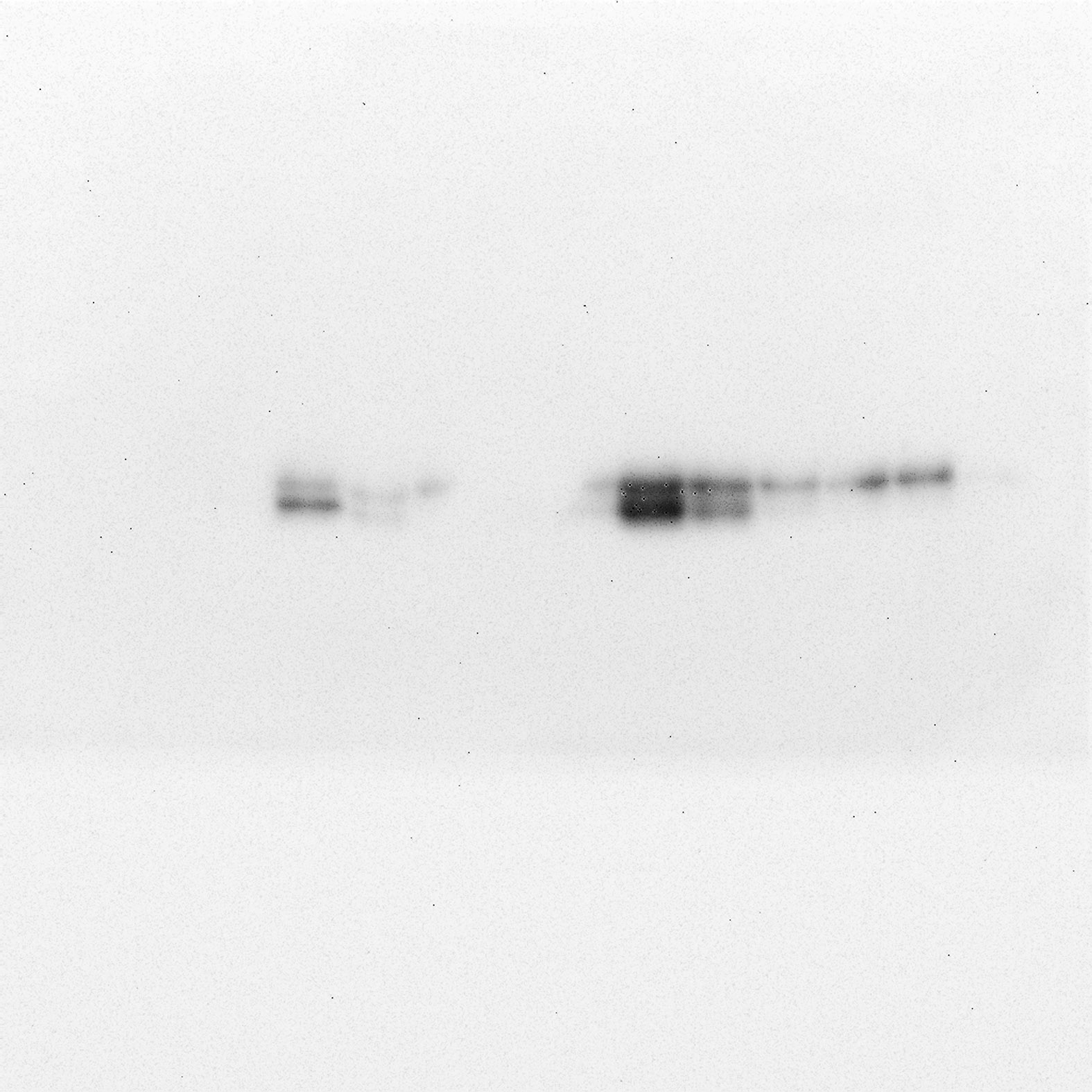

Supplement: Figure 3—figure supplement 2—source data 1. [file elife-86920-fig3-figsupp2-data1.zip › Figure 3-Figure Supplement 2 - Source Data 1/E_24-09-21_16 Bit_Gel 13_anti Shh Rabbit_2min35sec_Tonwertkorrektur.jpg]

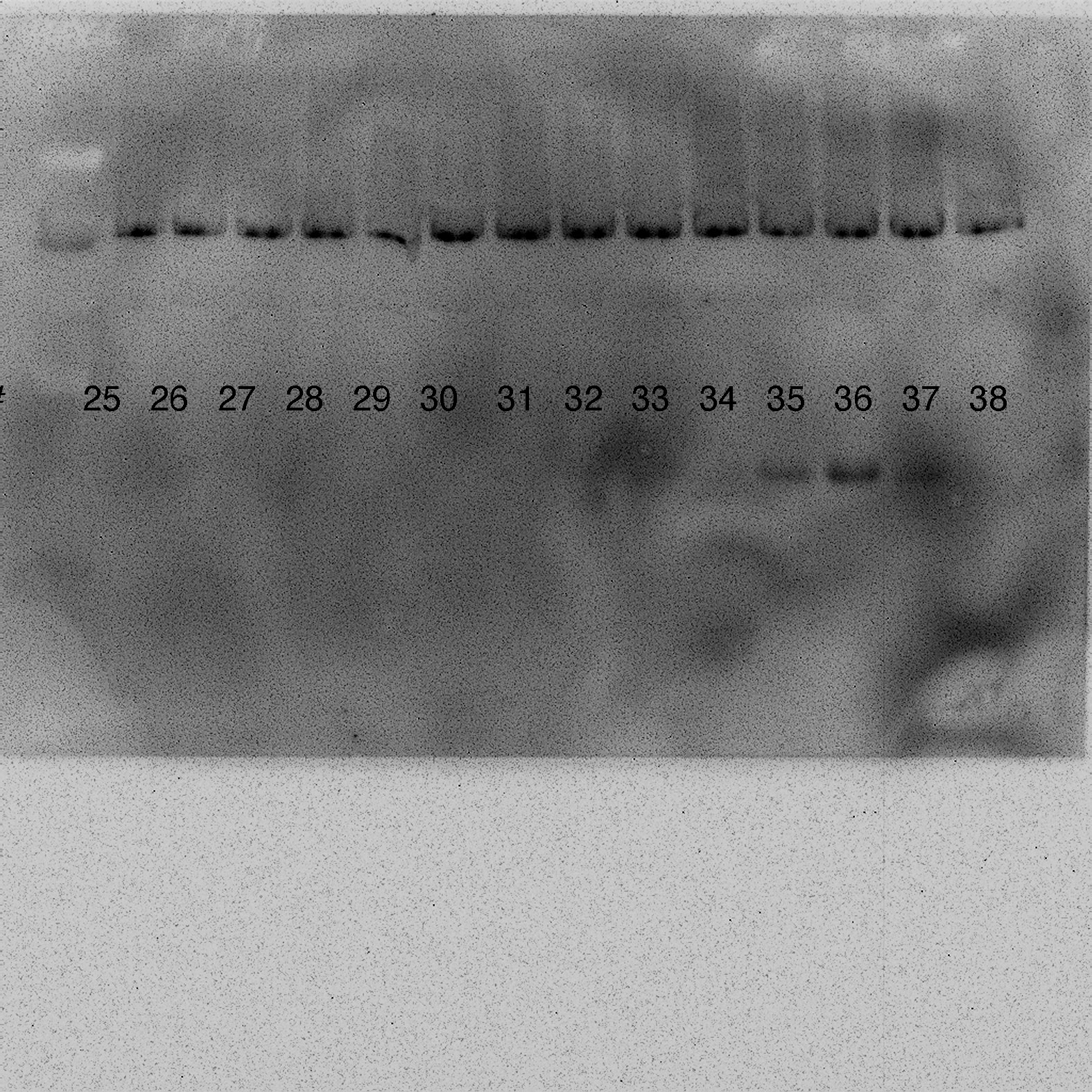

Supplement: Figure 3—figure supplement 2—source data 1. [file elife-86920-fig3-figsupp2-data1.zip › Figure 3-Figure Supplement 2 - Source Data 1/F_2024_02_09_16Bit_HPLC Gel 9_antiSHH_5min labelled.jpg]

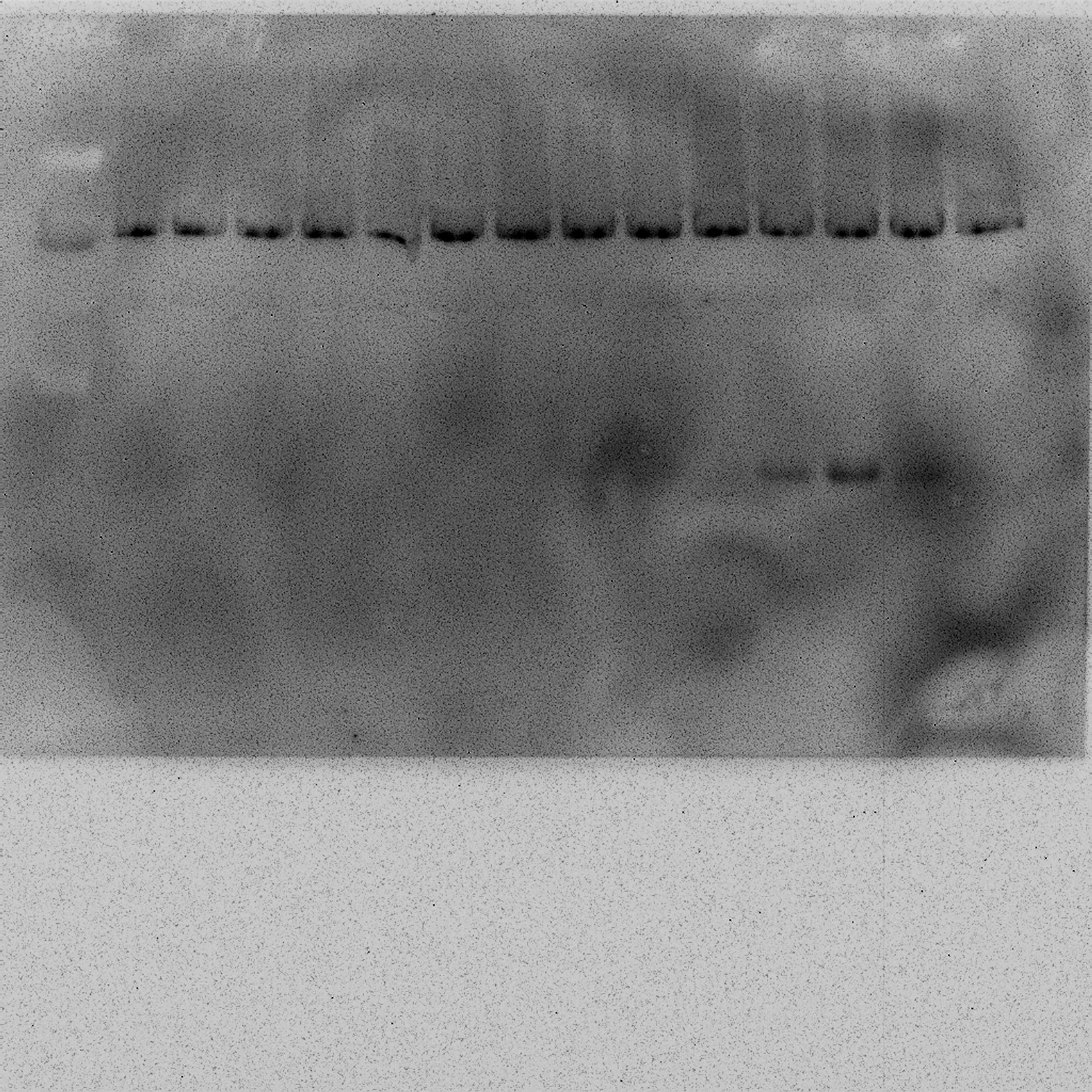

Supplement: Figure 3—figure supplement 2—source data 1. [file elife-86920-fig3-figsupp2-data1.zip › Figure 3-Figure Supplement 2 - Source Data 1/F_2024_02_09_16Bit_HPLC Gel 9_antiSHH_5min.jpg]

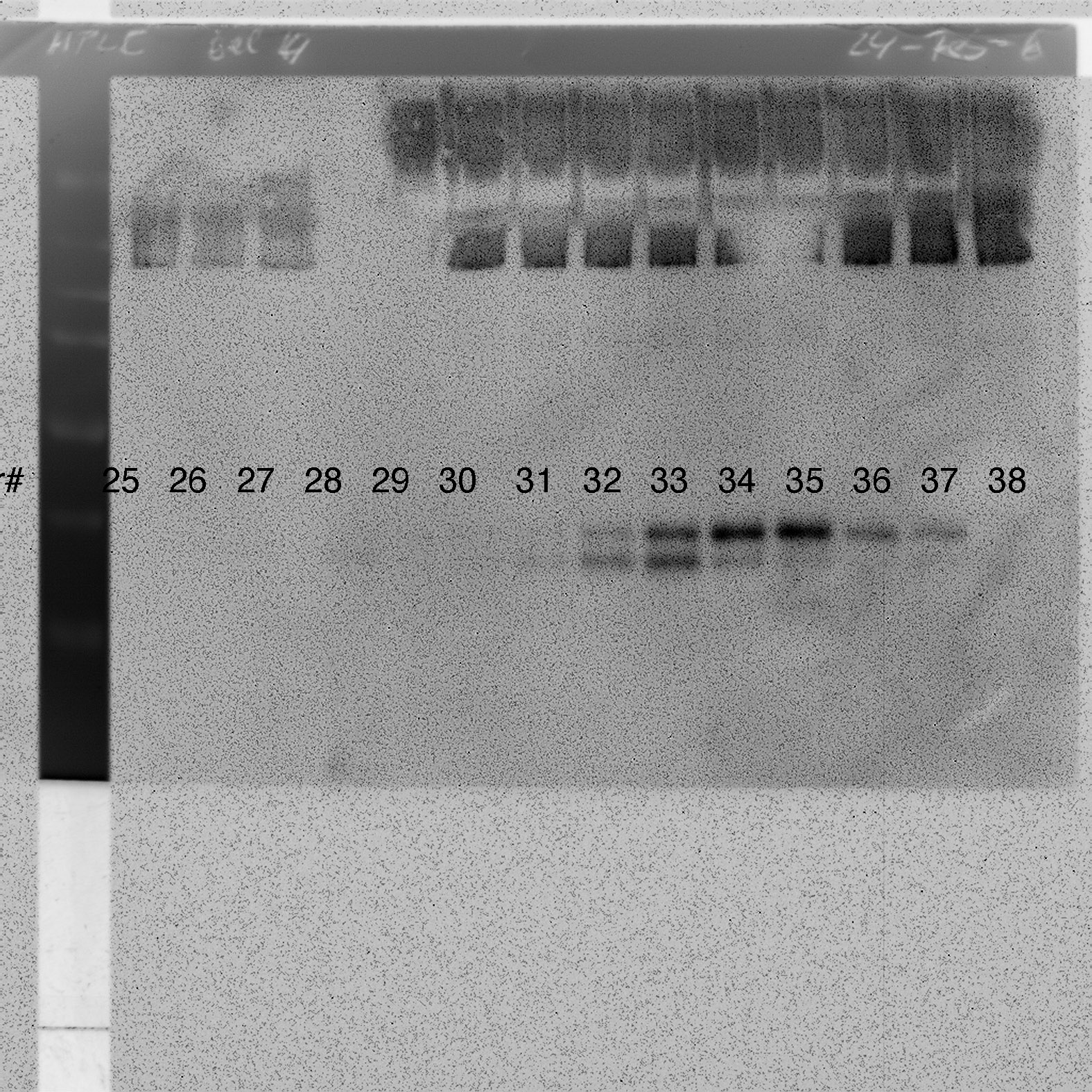

Supplement: Figure 3—figure supplement 2—source data 1. [file elife-86920-fig3-figsupp2-data1.zip › Figure 3-Figure Supplement 2 - Source Data 1/G_2024-02-07_16 Bit_HPLC Lauf Gel 4_anti SHH_5min labelled.jpg]

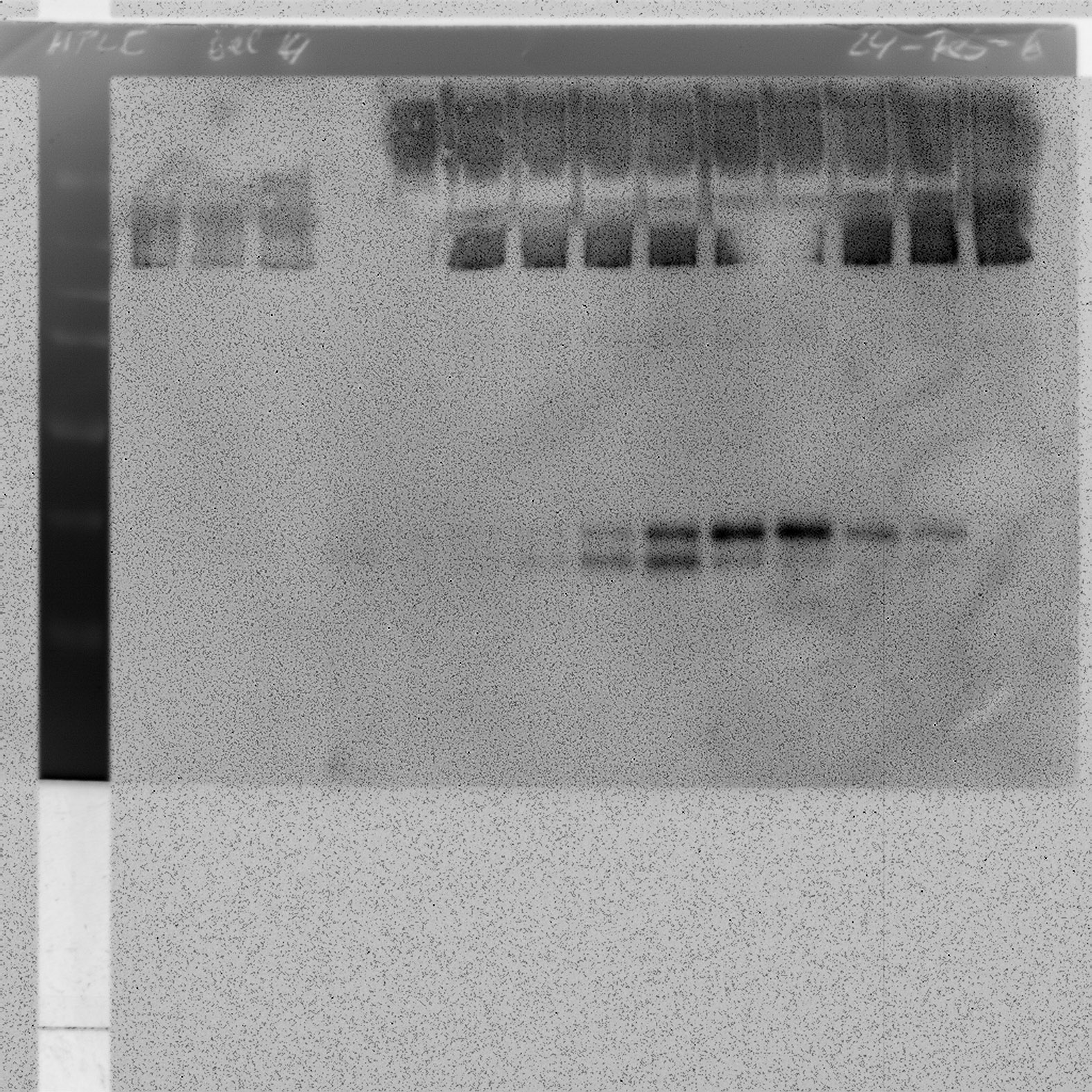

Supplement: Figure 3—figure supplement 2—source data 1. [file elife-86920-fig3-figsupp2-data1.zip › Figure 3-Figure Supplement 2 - Source Data 1/G_2024-02-07_16 Bit_HPLC Lauf Gel 4_anti SHH_5min.jpg]

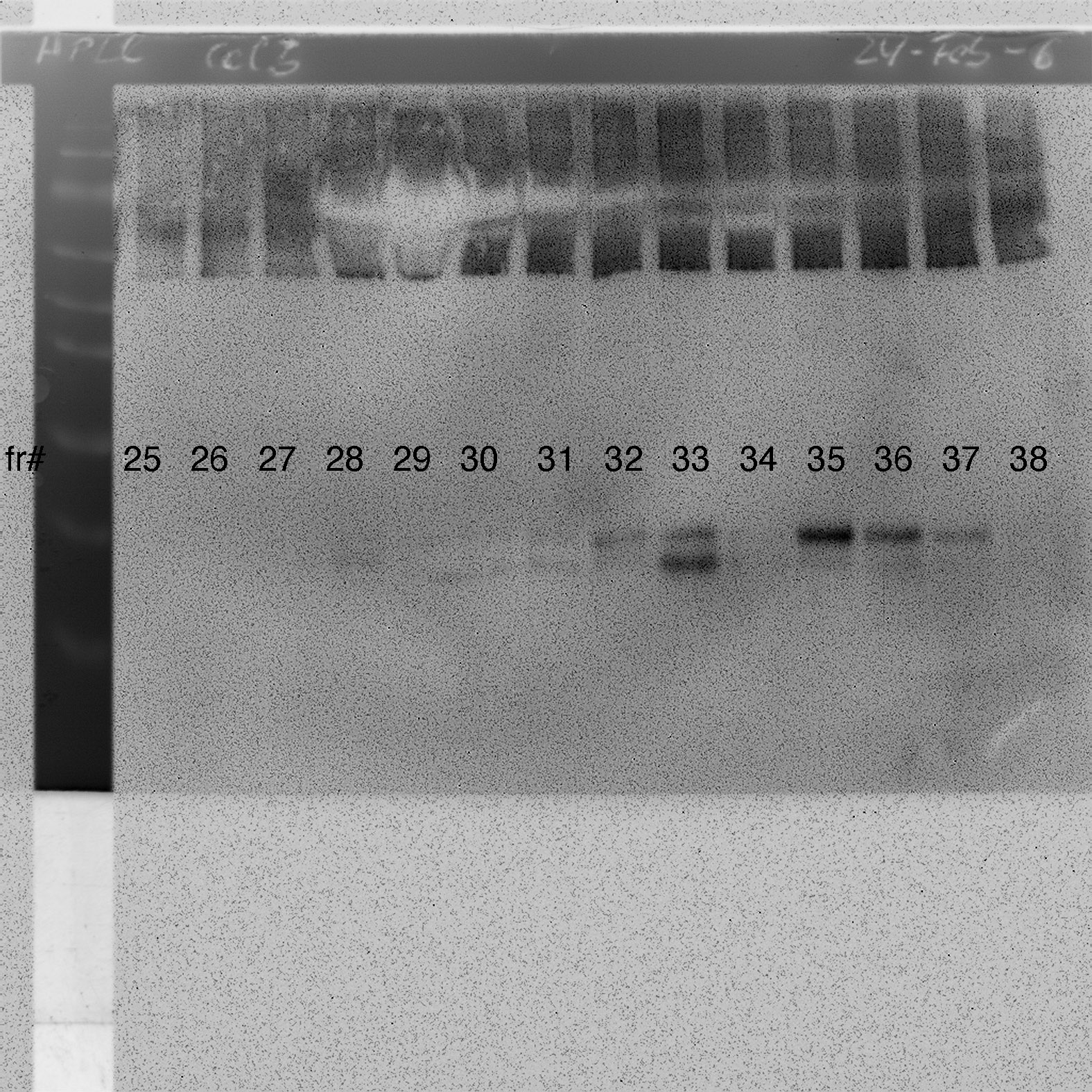

Supplement: Figure 3—figure supplement 2—source data 1. [file elife-86920-fig3-figsupp2-data1.zip › Figure 3-Figure Supplement 2 - Source Data 1/H_2024-02-07_16 Bit_HPLC Lauf Gel 3_anti SHH_5min labelled.jpg]

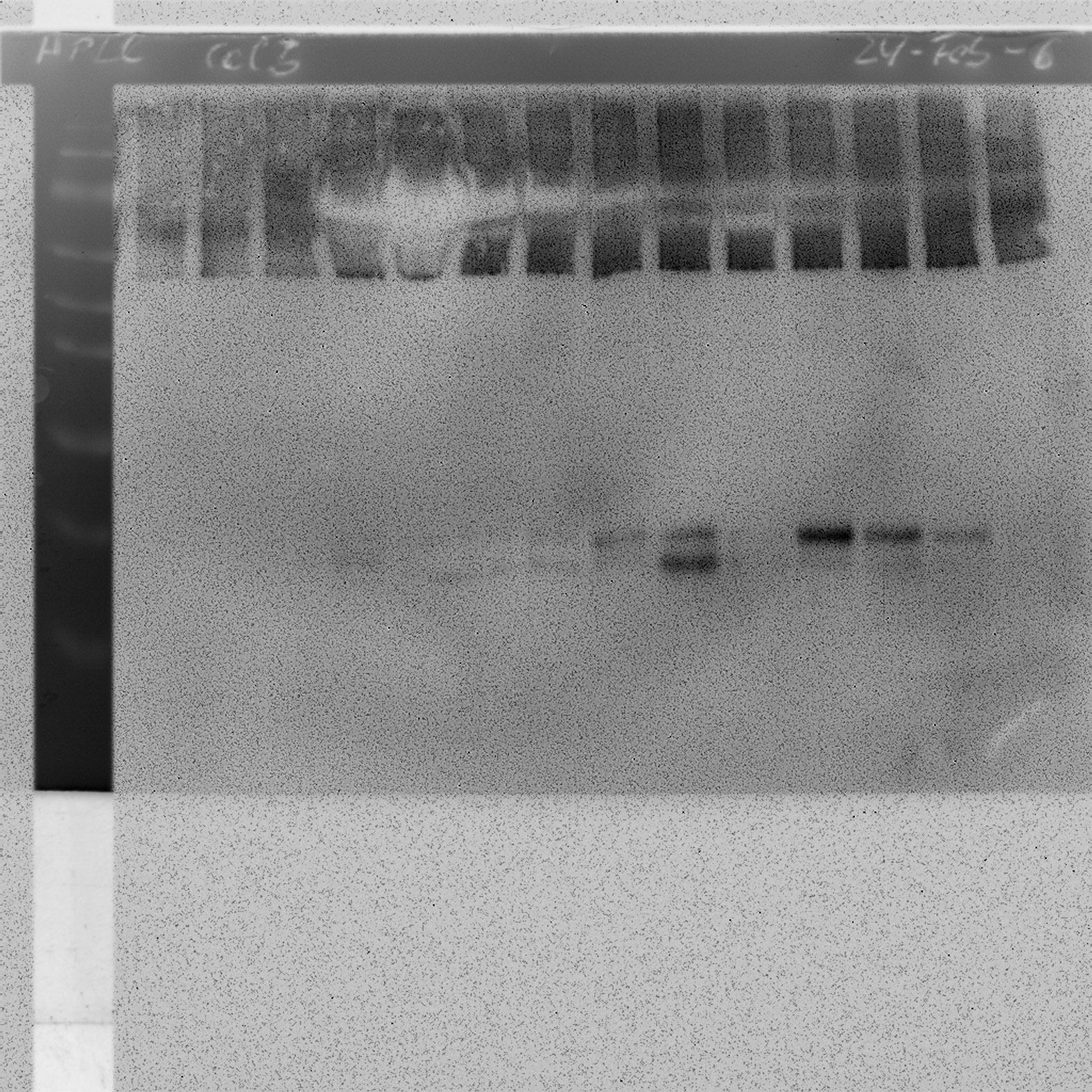

Supplement: Figure 3—figure supplement 2—source data 1. [file elife-86920-fig3-figsupp2-data1.zip › Figure 3-Figure Supplement 2 - Source Data 1/H_2024-02-07_16 Bit_HPLC Lauf Gel 3_anti SHH_5min.jpg]

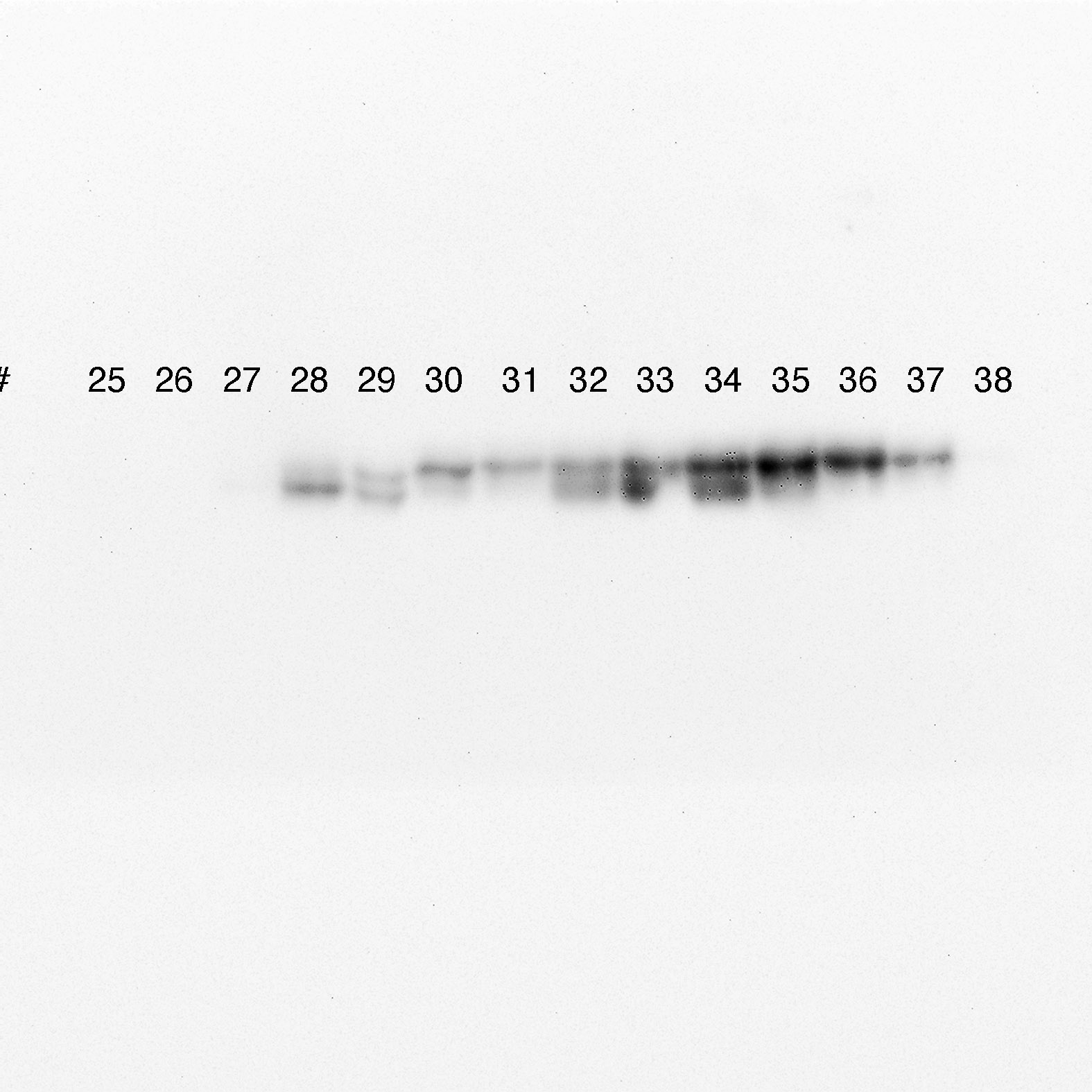

Supplement: Figure 3—figure supplement 2—source data 1. [file elife-86920-fig3-figsupp2-data1.zip › Figure 3-Figure Supplement 2 - Source Data 1/I_24-09-21_16 Bit_Gel 14_anti Shh Rabbit_2min11sec_Tonwertkorrektur labelled.jpg]

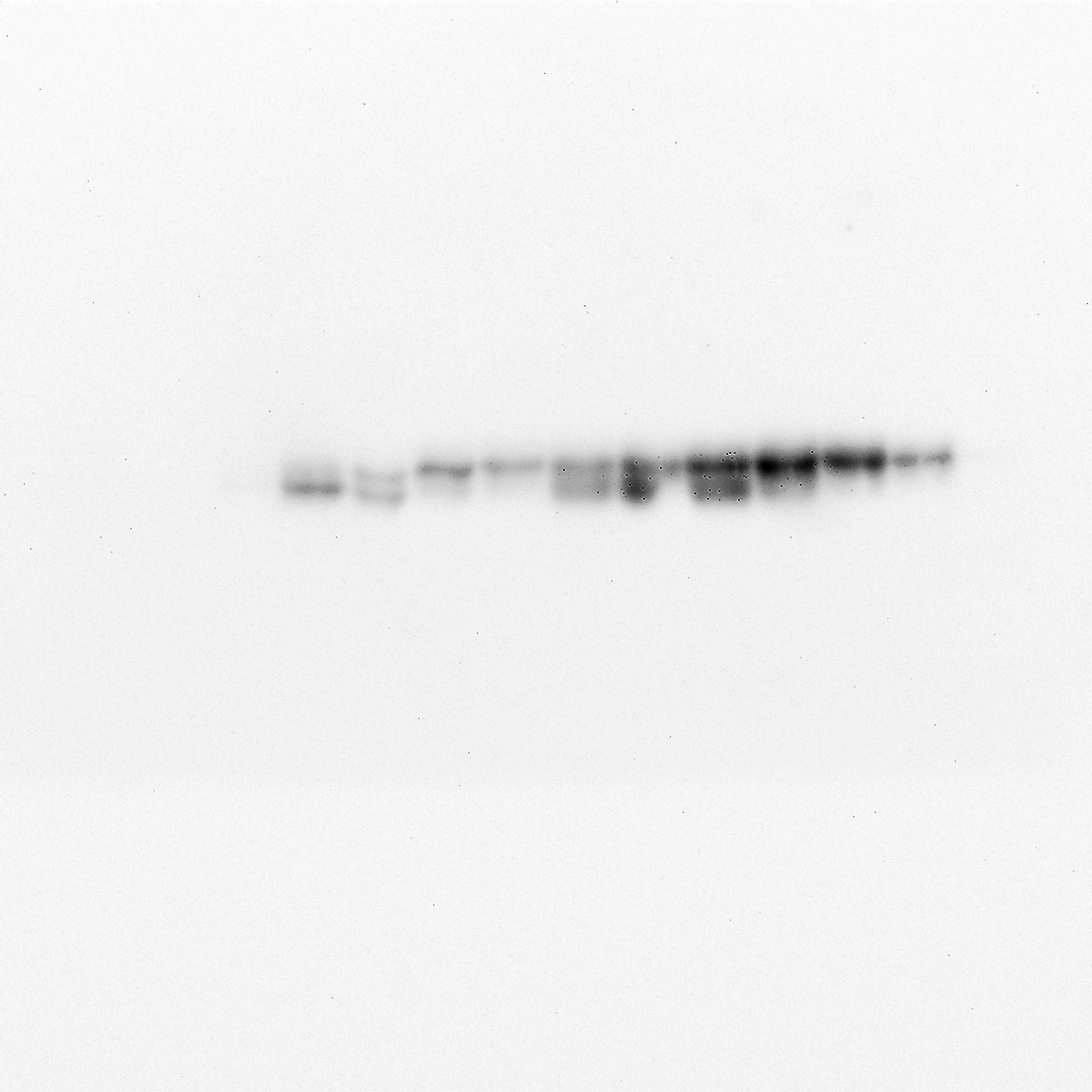

Supplement: Figure 3—figure supplement 2—source data 1. [file elife-86920-fig3-figsupp2-data1.zip › Figure 3-Figure Supplement 2 - Source Data 1/I_24-09-21_16 Bit_Gel 14_anti Shh Rabbit_2min11sec_Tonwertkorrektur.jpg]

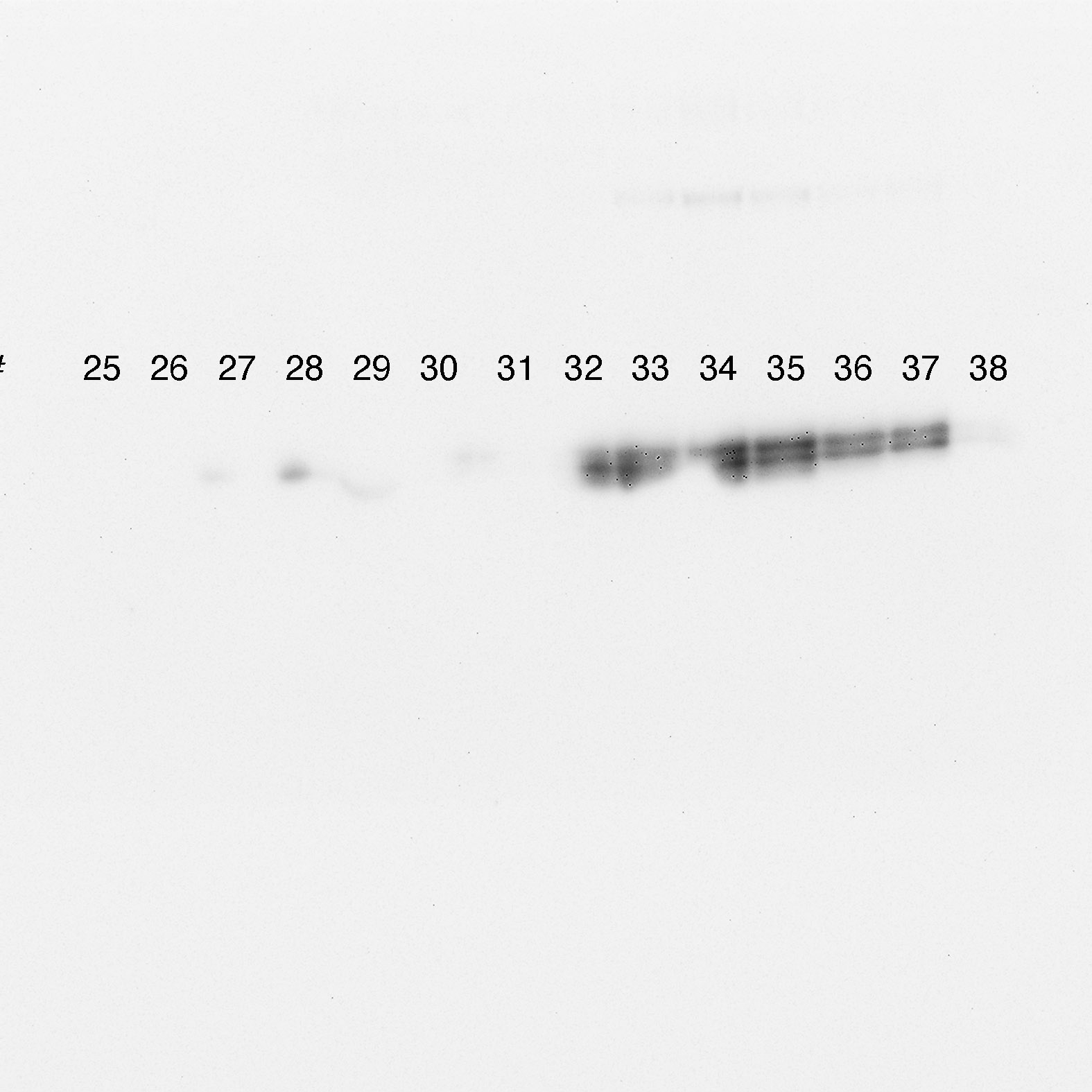

Supplement: Figure 3—figure supplement 2—source data 1. [file elife-86920-fig3-figsupp2-data1.zip › Figure 3-Figure Supplement 2 - Source Data 1/J_20210923_Gel 6_anti Shh Rabbit_16bit_2min33sec labelled.jpg]

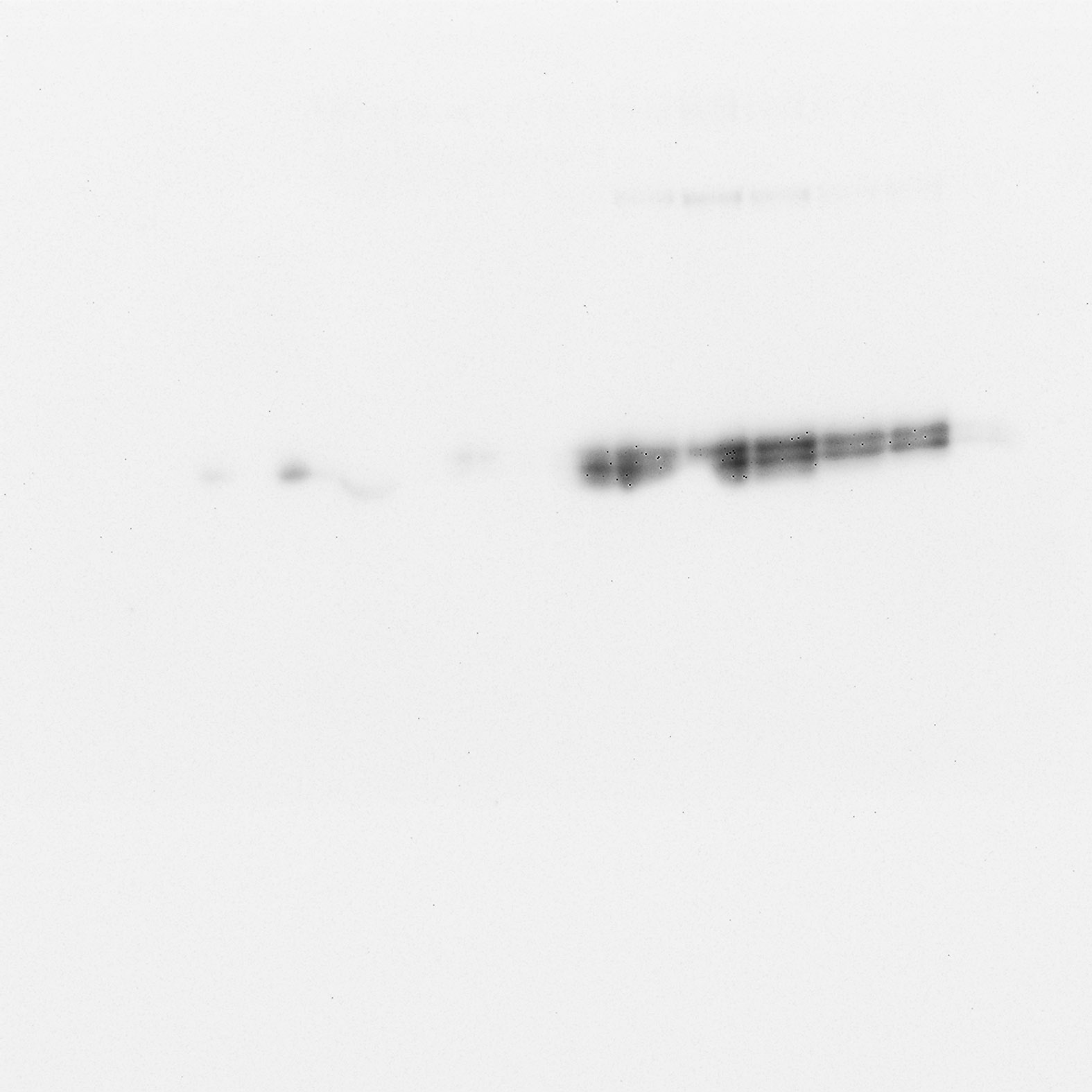

Supplement: Figure 3—figure supplement 2—source data 1. [file elife-86920-fig3-figsupp2-data1.zip › Figure 3-Figure Supplement 2 - Source Data 1/J_20210923_Gel 6_anti Shh Rabbit_16bit_2min33sec.jpg]

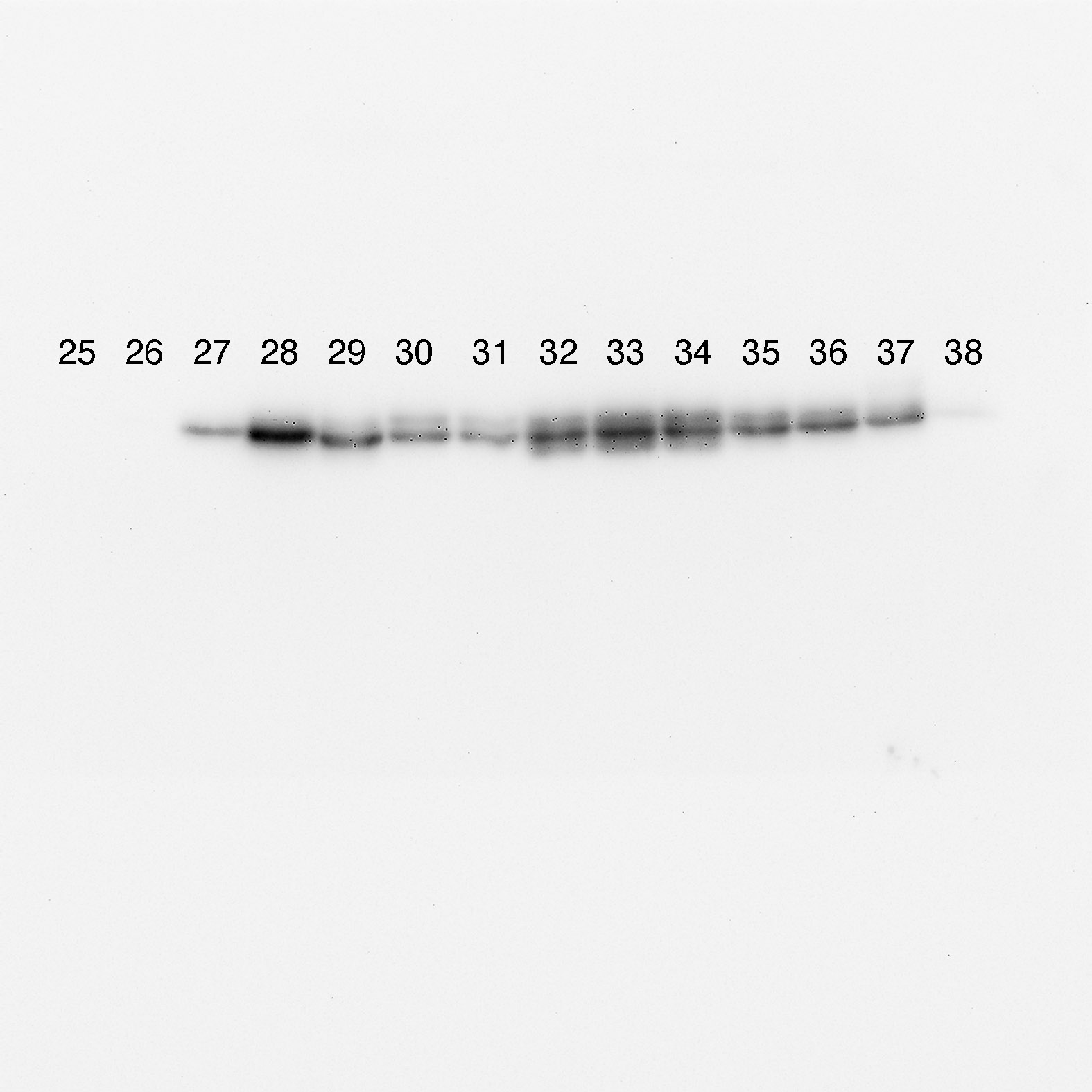

Supplement: Figure 3—figure supplement 2—source data 1. [file elife-86920-fig3-figsupp2-data1.zip › Figure 3-Figure Supplement 2 - Source Data 1/K_20210923_Gel 5_anti Shh Rabbit_16bit_1min51sec labelled.jpg]

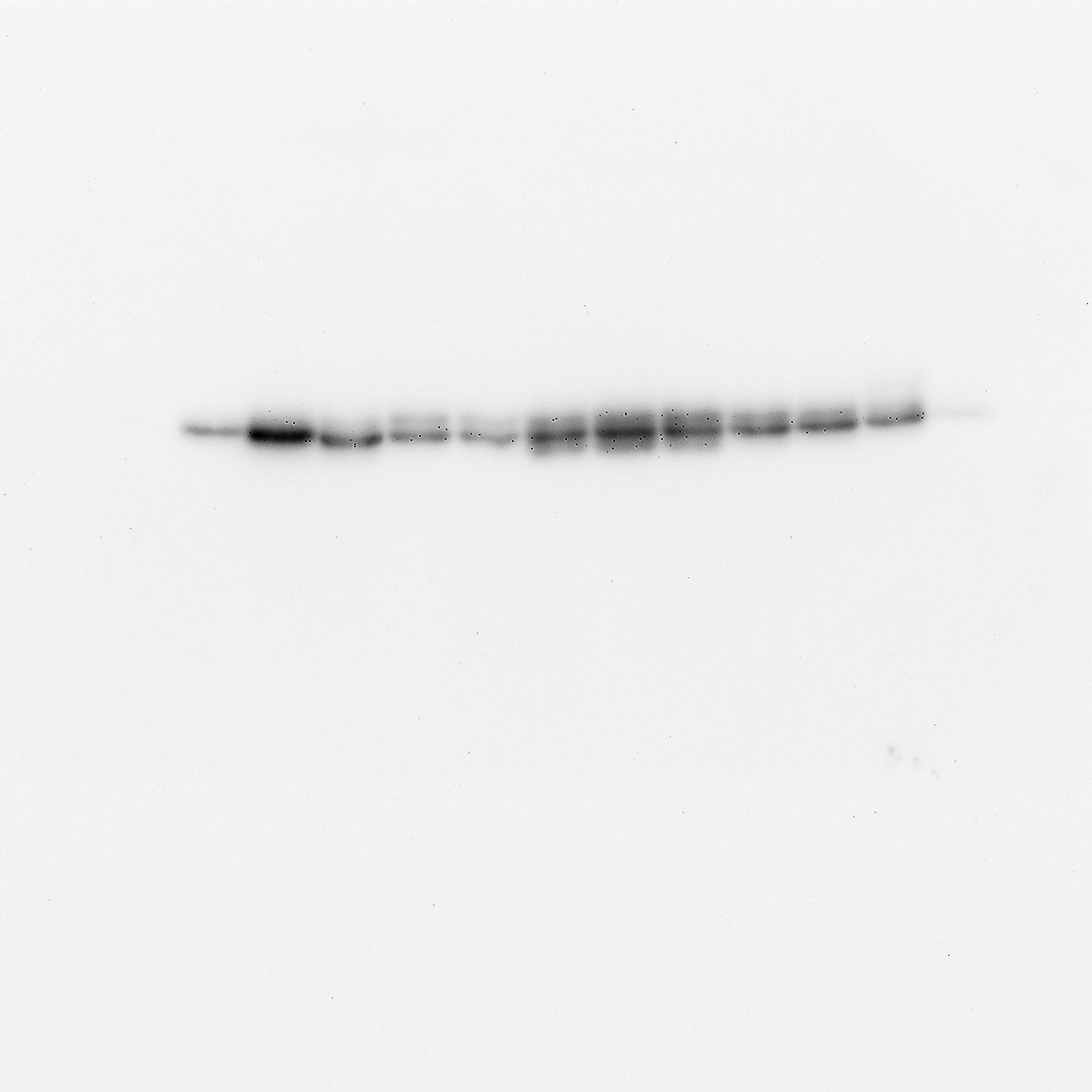

Supplement: Figure 3—figure supplement 2—source data 1. [file elife-86920-fig3-figsupp2-data1.zip › Figure 3-Figure Supplement 2 - Source Data 1/K_20210923_Gel 5_anti Shh Rabbit_16bit_1min51sec.jpg]

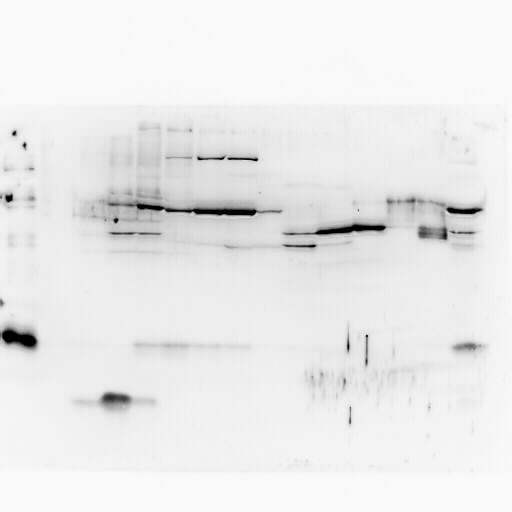

Supplement: Figure 4—source data 1. — A, B contains files demonstrating that palmitoylated Shh and non-palmitoylated C25SShh are both biologically active. C, D Excel file containing raw Shh gel filtration and bioactivity data (as shown in Excel file C, a duplicate bioactivity analysis of eluted fractions is shown) as shown in Figure 4C. D shows an uncropped western blot of Drosophila Hh expressed from S2 cells into media containing 10%FCS subsequent gel filtration analysis. Hh proteins are marked. C, D Quantification of Hh detected in gel filtration analysis. [file elife-86920-fig4-data1.zip › Figure_4_Source_Data_1 /D_antiHH_4-5_2_4x4_3m.JPG]

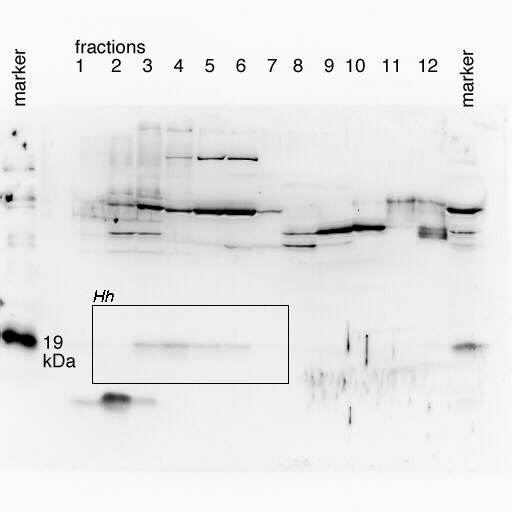

Supplement: Figure 4—source data 1. — A, B contains files demonstrating that palmitoylated Shh and non-palmitoylated C25SShh are both biologically active. C, D Excel file containing raw Shh gel filtration and bioactivity data (as shown in Excel file C, a duplicate bioactivity analysis of eluted fractions is shown) as shown in Figure 4C. D shows an uncropped western blot of Drosophila Hh expressed from S2 cells into media containing 10%FCS subsequent gel filtration analysis. Hh proteins are marked. C, D Quantification of Hh detected in gel filtration analysis. [file elife-86920-fig4-data1.zip › Figure_4_Source_Data_1 /D_antiHH_4-5_2_4x4_3m_labelled.JPG]

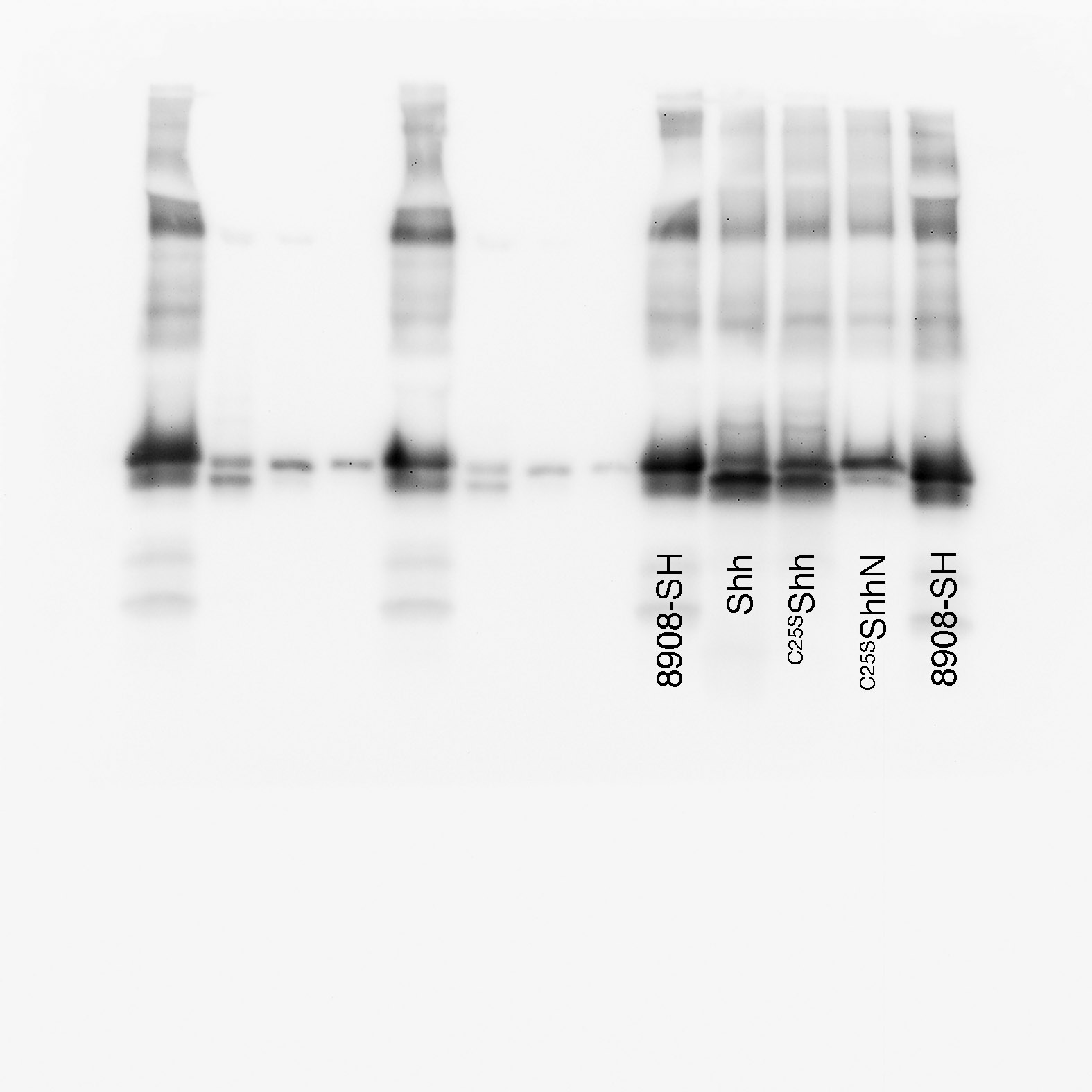

Supplement: Figure 4—figure supplement 1—source data 1. [file elife-86920-fig4-figsupp1-data1.zip › Figure Supplement 5 - Source Data 1/E_24-01-16_16 Bit_Kay_Gel 2_antiShh Rabbit_10-4sec labelled.jpg]

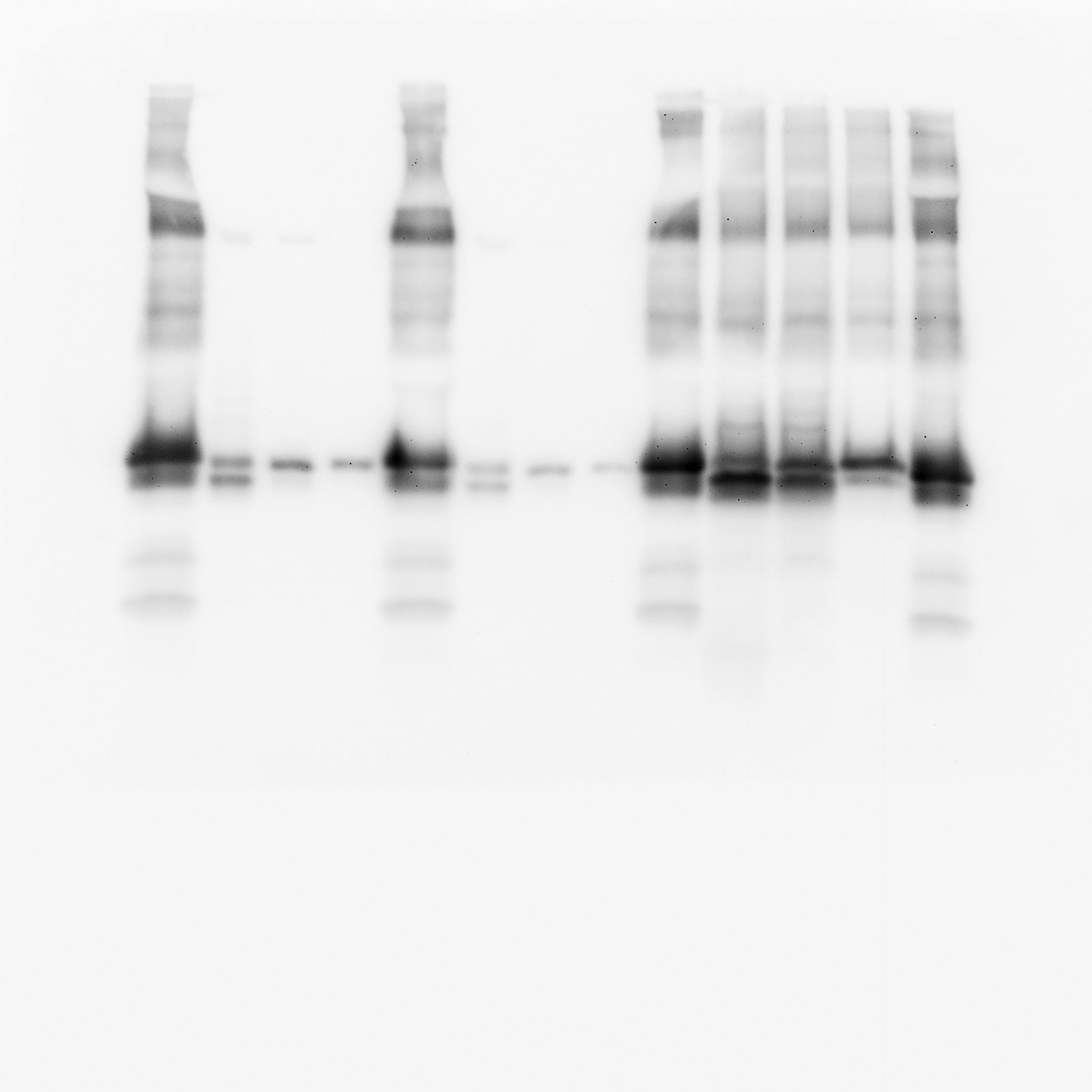

Supplement: Figure 4—figure supplement 1—source data 1. [file elife-86920-fig4-figsupp1-data1.zip › Figure Supplement 5 - Source Data 1/E_24-01-16_16 Bit_Kay_Gel 2_antiShh Rabbit_10-4sec.jpg]

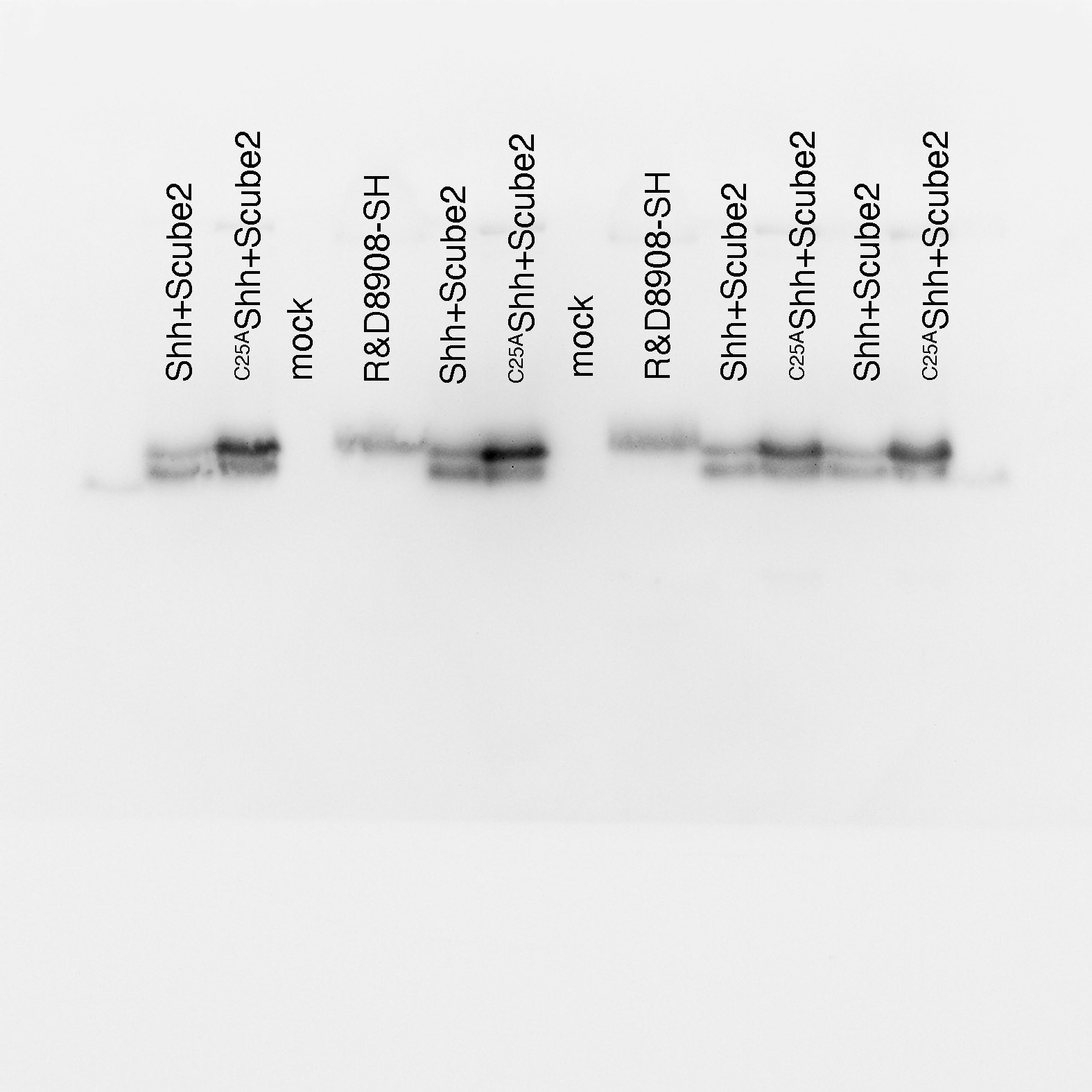

Supplement: Figure 4—figure supplement 1—source data 1. [file elife-86920-fig4-figsupp1-data1.zip › Figure Supplement 5 - Source Data 1/F_2021-03-21_VS32_H7 S268 48h+_Gel1_aShh_16bit_SS_3sec labelled.jpg]

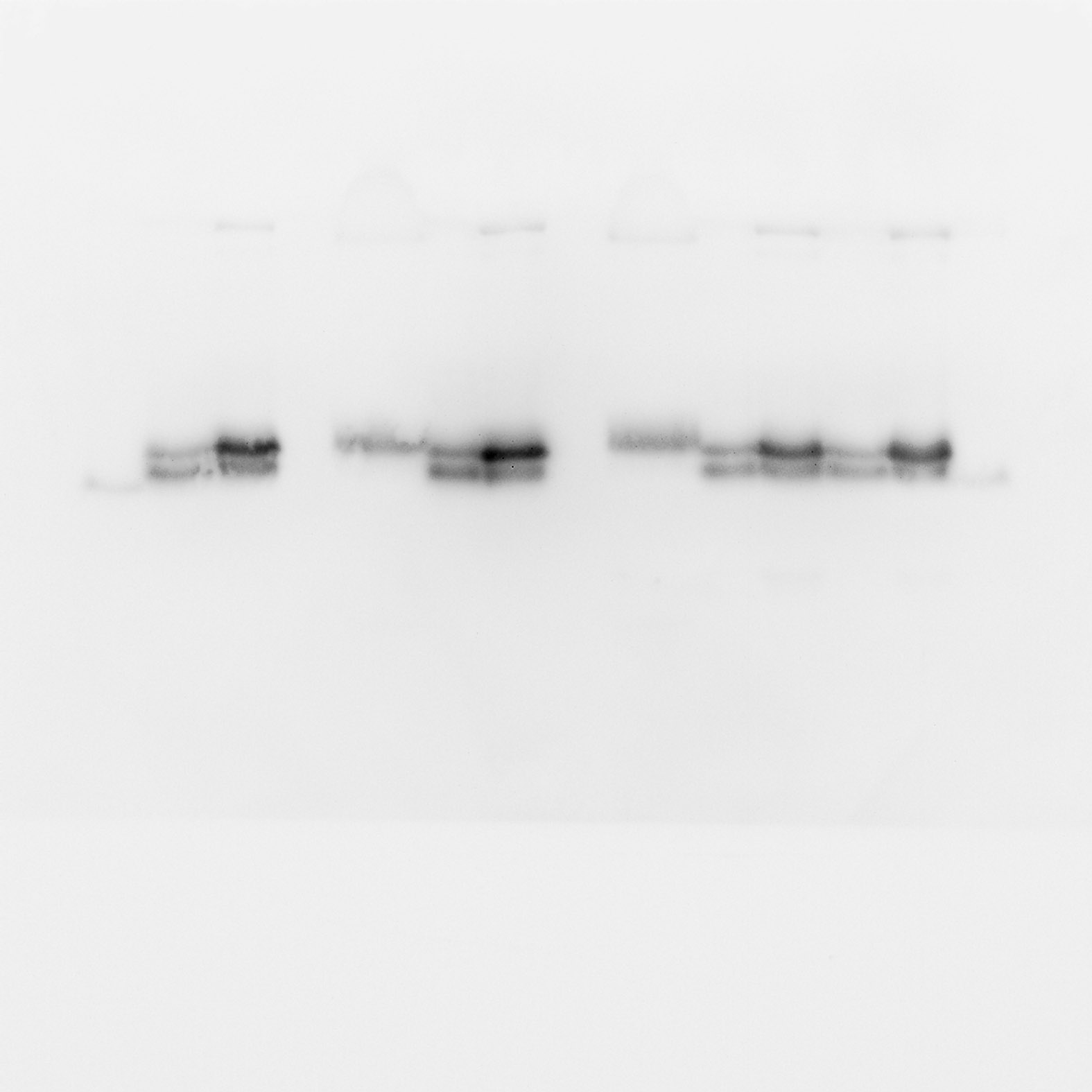

Supplement: Figure 4—figure supplement 1—source data 1. [file elife-86920-fig4-figsupp1-data1.zip › Figure Supplement 5 - Source Data 1/F_2021-03-21_VS32_H7 S268 48h+_Gel1_aShh_16bit_SS_3sec.jpg]

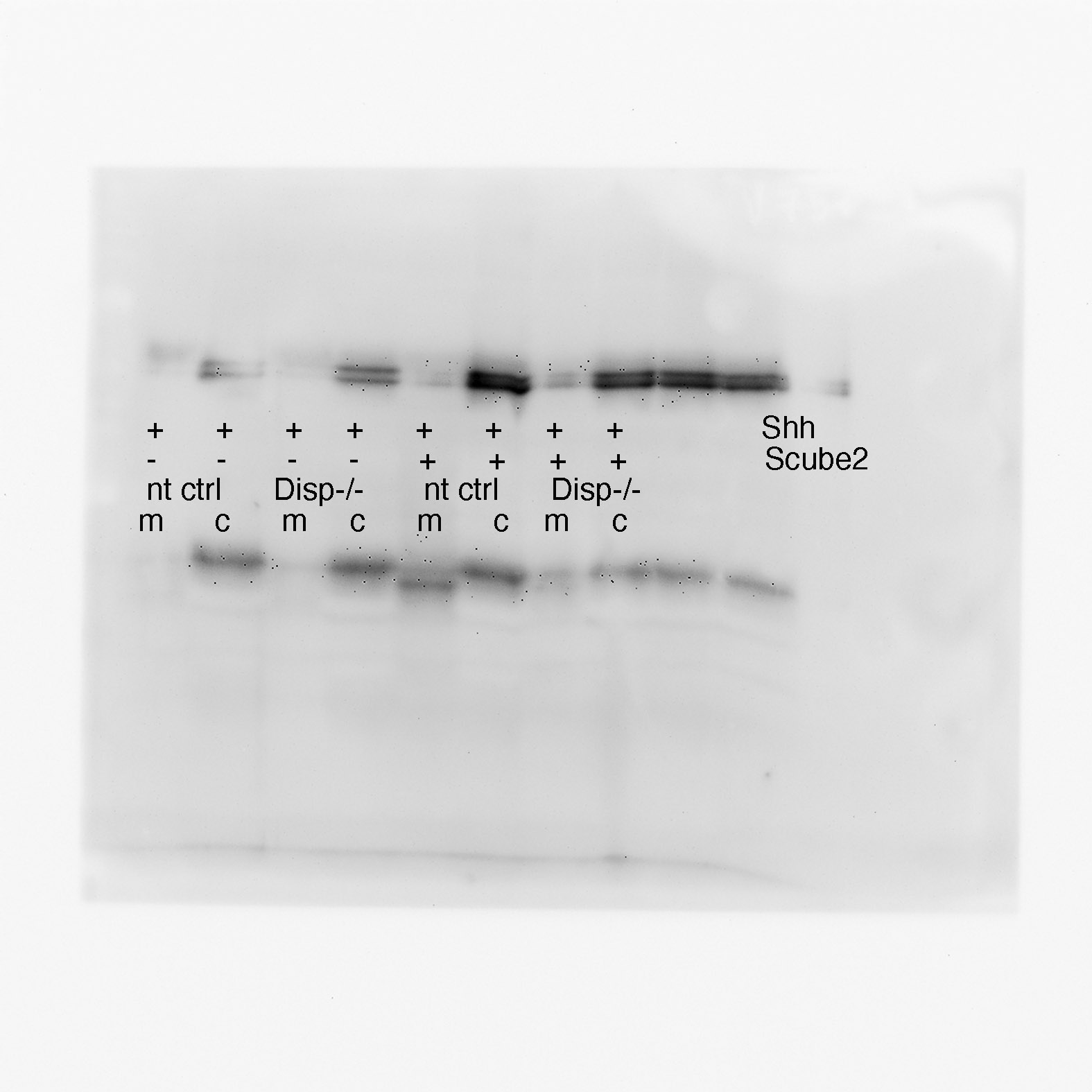

Supplement: Figure 5—source data 1. — A, B contain uncropped western blots shown in Figure 5A and B. C contains uncropped western blots of seven biological replicates showing that HDL presence renders Scube2 function obsolete. Prizm file C quantifies similar relative Shh release from nt Ctrl cells in the presence of HDL, irrespective of Scube2 presence or absence (as shown in Figure 5C). The Excel file contains raw data of Figure 5A’, A’’, B’ and B’’. [file elife-86920-fig5-data1.zip › Figure_5_Source_Data_1 /A_V757_shh labelled.jpg]

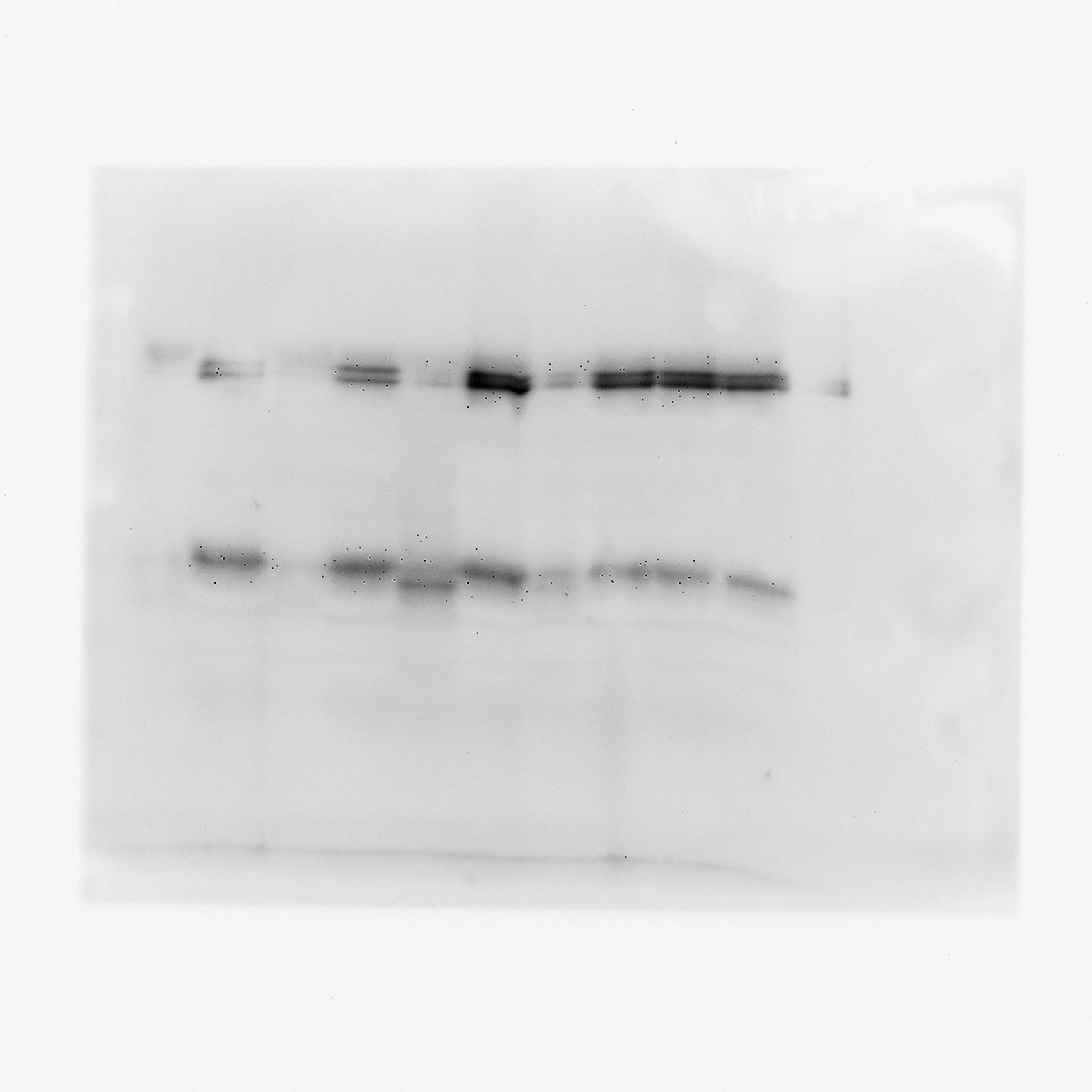

Supplement: Figure 5—source data 1. — A, B contain uncropped western blots shown in Figure 5A and B. C contains uncropped western blots of seven biological replicates showing that HDL presence renders Scube2 function obsolete. Prizm file C quantifies similar relative Shh release from nt Ctrl cells in the presence of HDL, irrespective of Scube2 presence or absence (as shown in Figure 5C). The Excel file contains raw data of Figure 5A’, A’’, B’ and B’’. [file elife-86920-fig5-data1.zip › Figure_5_Source_Data_1 /A_V757_shh.jpg]

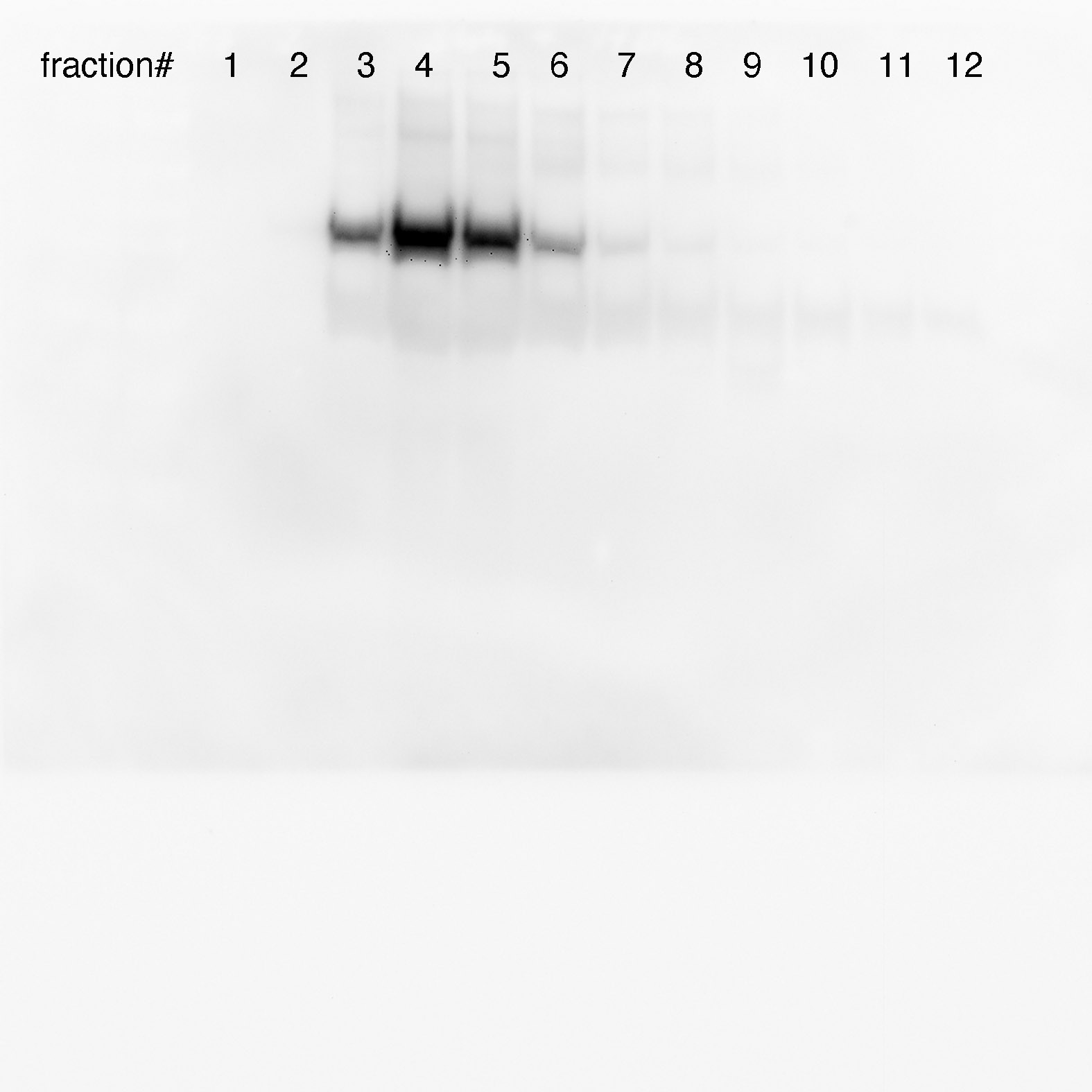

Supplement: Figure 5—source data 1. — A, B contain uncropped western blots shown in Figure 5A and B. C contains uncropped western blots of seven biological replicates showing that HDL presence renders Scube2 function obsolete. Prizm file C quantifies similar relative Shh release from nt Ctrl cells in the presence of HDL, irrespective of Scube2 presence or absence (as shown in Figure 5C). The Excel file contains raw data of Figure 5A’, A’’, B’ and B’’. [file elife-86920-fig5-data1.zip › Figure_5_Source_Data_1 /B''_GF2_Shh+HDL_antiApoE4 labelled.jpg]

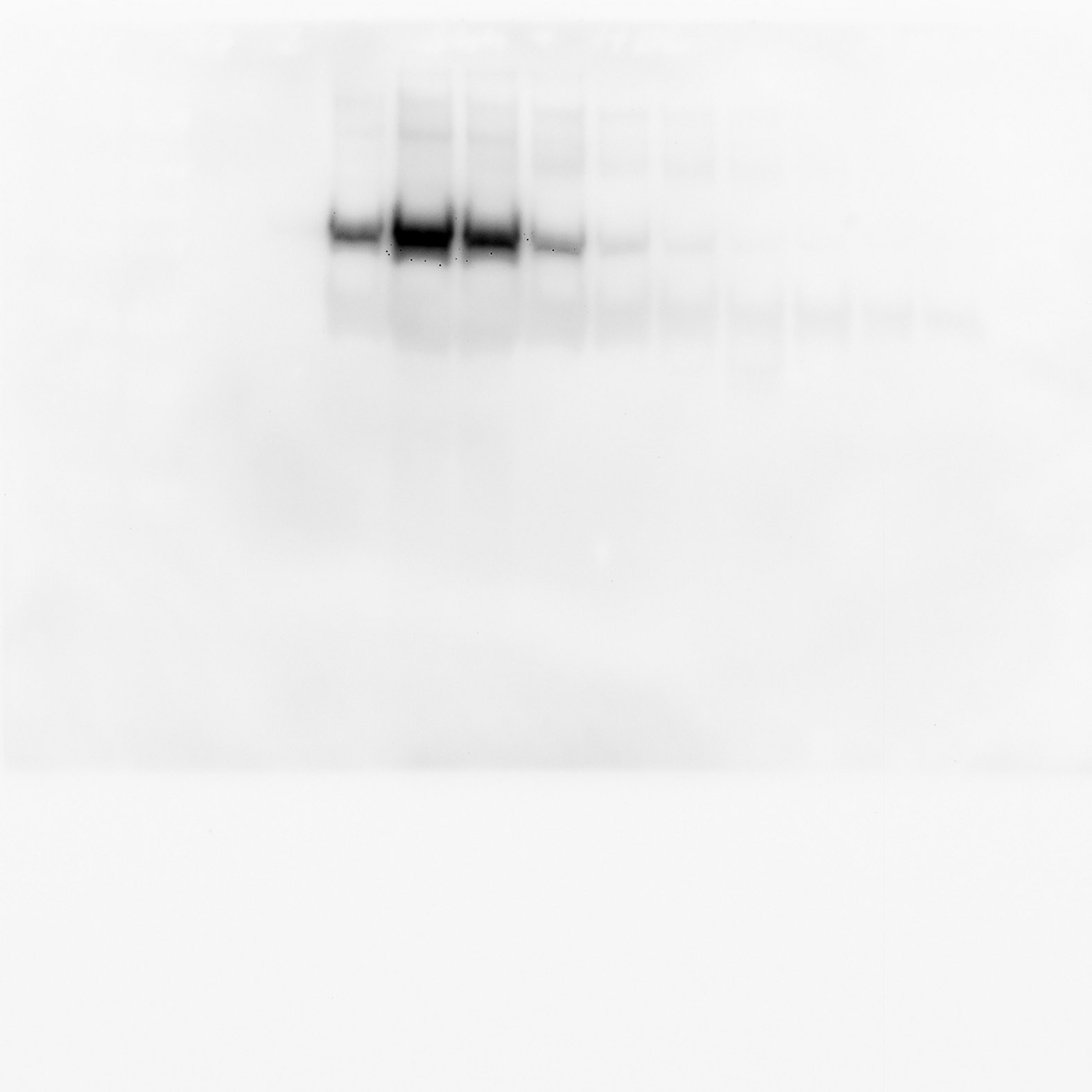

Supplement: Figure 5—source data 1. — A, B contain uncropped western blots shown in Figure 5A and B. C contains uncropped western blots of seven biological replicates showing that HDL presence renders Scube2 function obsolete. Prizm file C quantifies similar relative Shh release from nt Ctrl cells in the presence of HDL, irrespective of Scube2 presence or absence (as shown in Figure 5C). The Excel file contains raw data of Figure 5A’, A’’, B’ and B’’. [file elife-86920-fig5-data1.zip › Figure_5_Source_Data_1 /B''_GF2_Shh+HDL_antiApoE4.jpg]

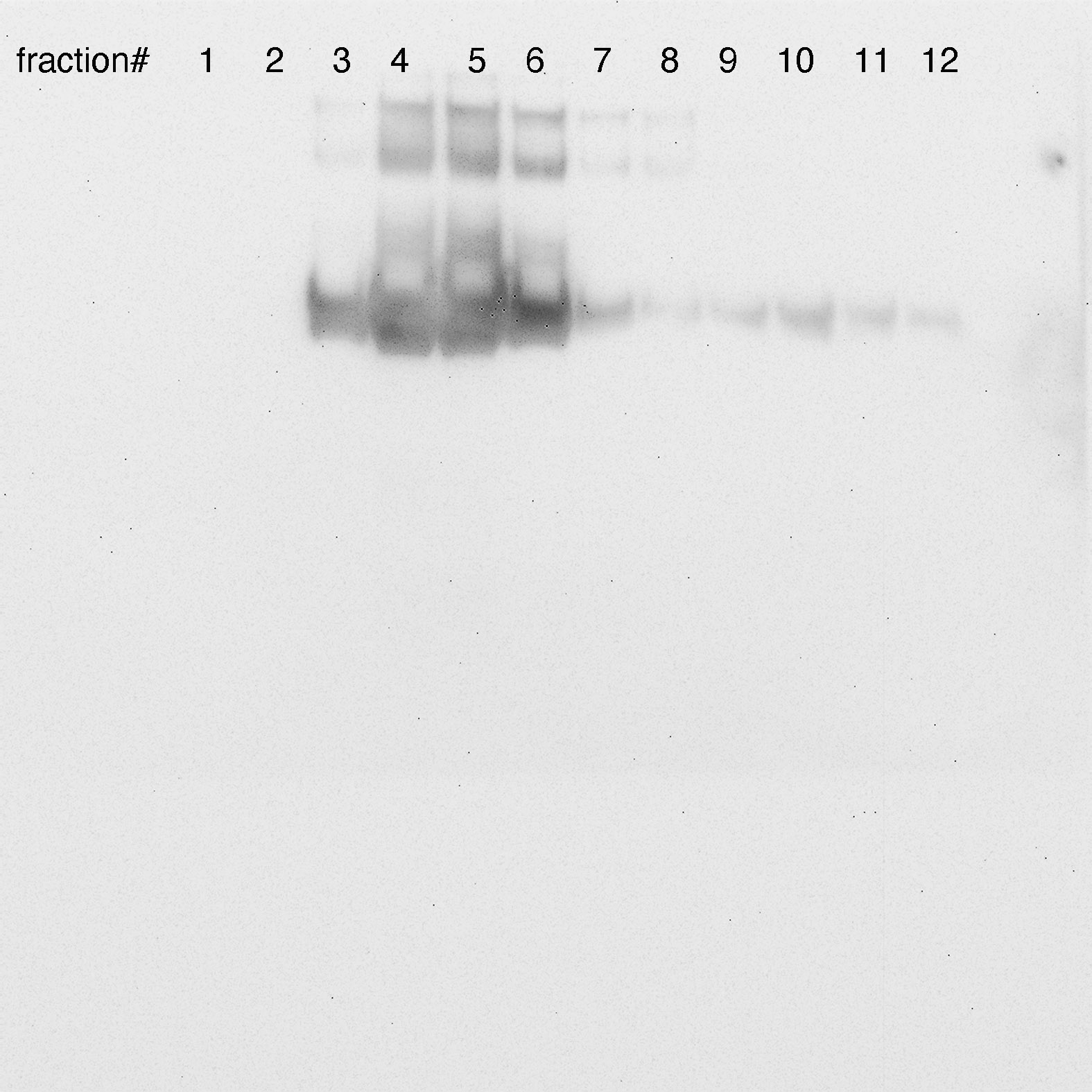

Supplement: Figure 5—source data 1. — A, B contain uncropped western blots shown in Figure 5A and B. C contains uncropped western blots of seven biological replicates showing that HDL presence renders Scube2 function obsolete. Prizm file C quantifies similar relative Shh release from nt Ctrl cells in the presence of HDL, irrespective of Scube2 presence or absence (as shown in Figure 5C). The Excel file contains raw data of Figure 5A’, A’’, B’ and B’’. [file elife-86920-fig5-data1.zip › Figure_5_Source_Data_1 /B''_GF_antiApoA1 labelled.jpg]

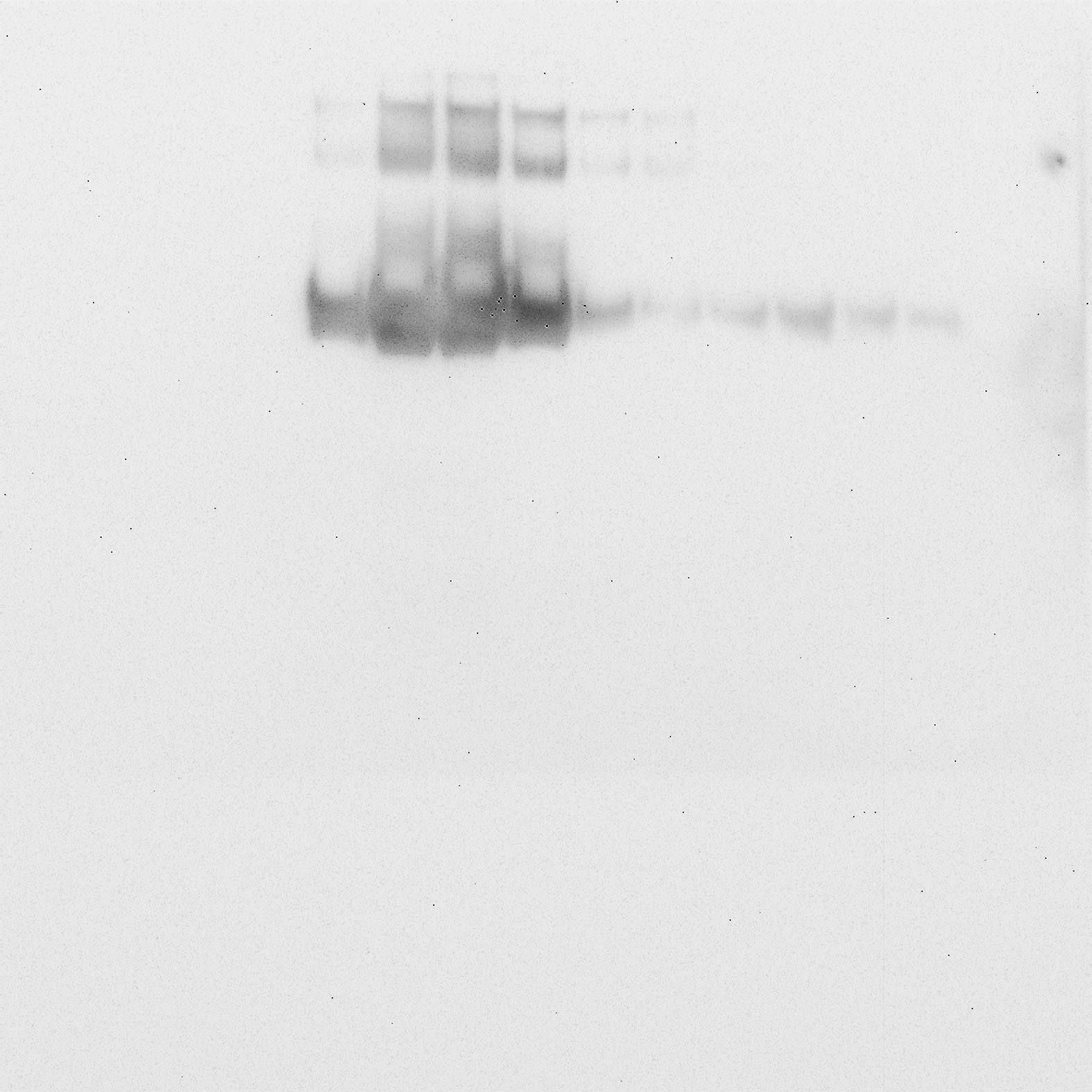

Supplement: Figure 5—source data 1. — A, B contain uncropped western blots shown in Figure 5A and B. C contains uncropped western blots of seven biological replicates showing that HDL presence renders Scube2 function obsolete. Prizm file C quantifies similar relative Shh release from nt Ctrl cells in the presence of HDL, irrespective of Scube2 presence or absence (as shown in Figure 5C). The Excel file contains raw data of Figure 5A’, A’’, B’ and B’’. [file elife-86920-fig5-data1.zip › Figure_5_Source_Data_1 /B''_GF_antiApoA1.jpg]

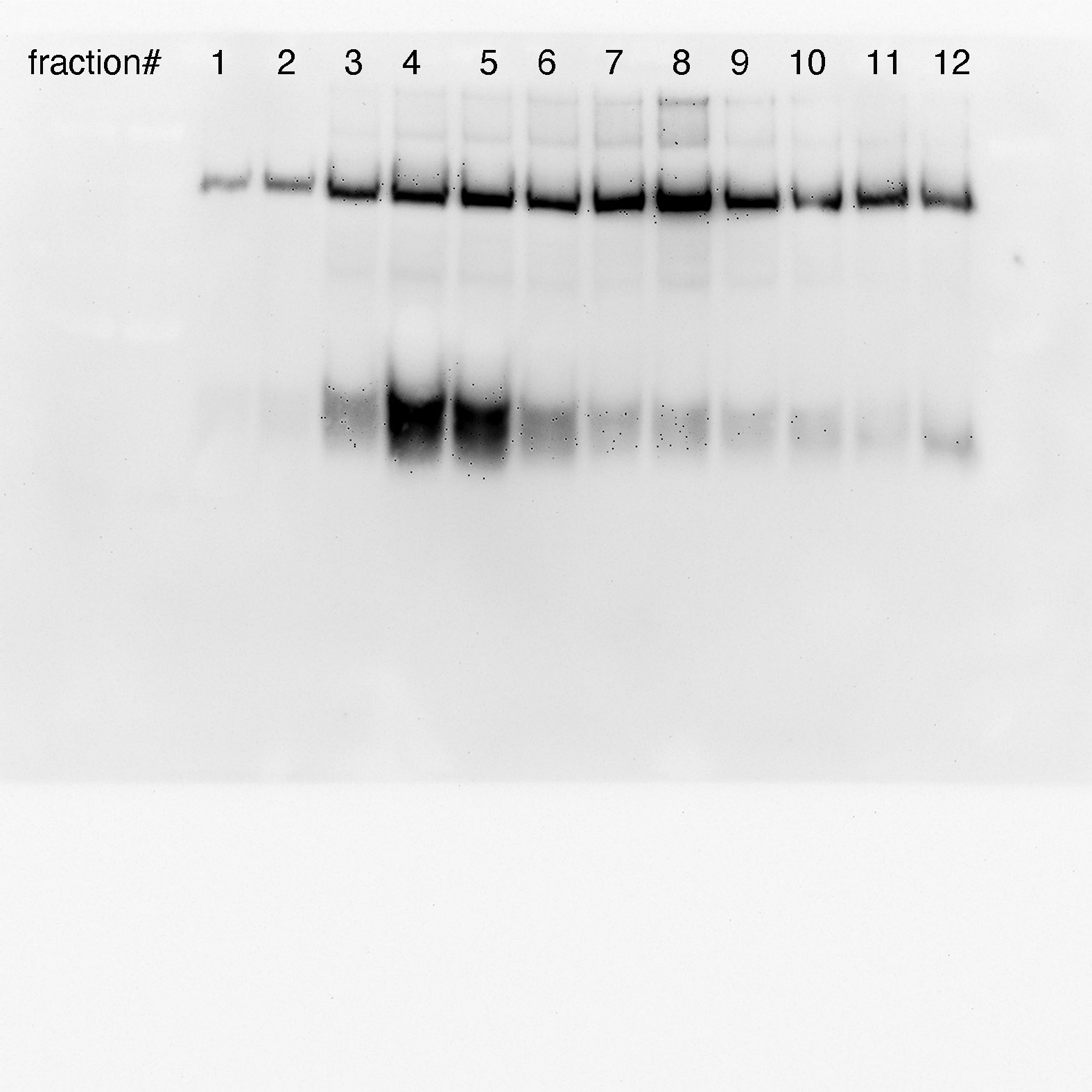

Supplement: Figure 5—source data 1. — A, B contain uncropped western blots shown in Figure 5A and B. C contains uncropped western blots of seven biological replicates showing that HDL presence renders Scube2 function obsolete. Prizm file C quantifies similar relative Shh release from nt Ctrl cells in the presence of HDL, irrespective of Scube2 presence or absence (as shown in Figure 5C). The Excel file contains raw data of Figure 5A’, A’’, B’ and B’’. [file elife-86920-fig5-data1.zip › Figure_5_Source_Data_1 /B''_GF_Gel 2_Shh+HDL_antiShh rabbit labelled.jpg]

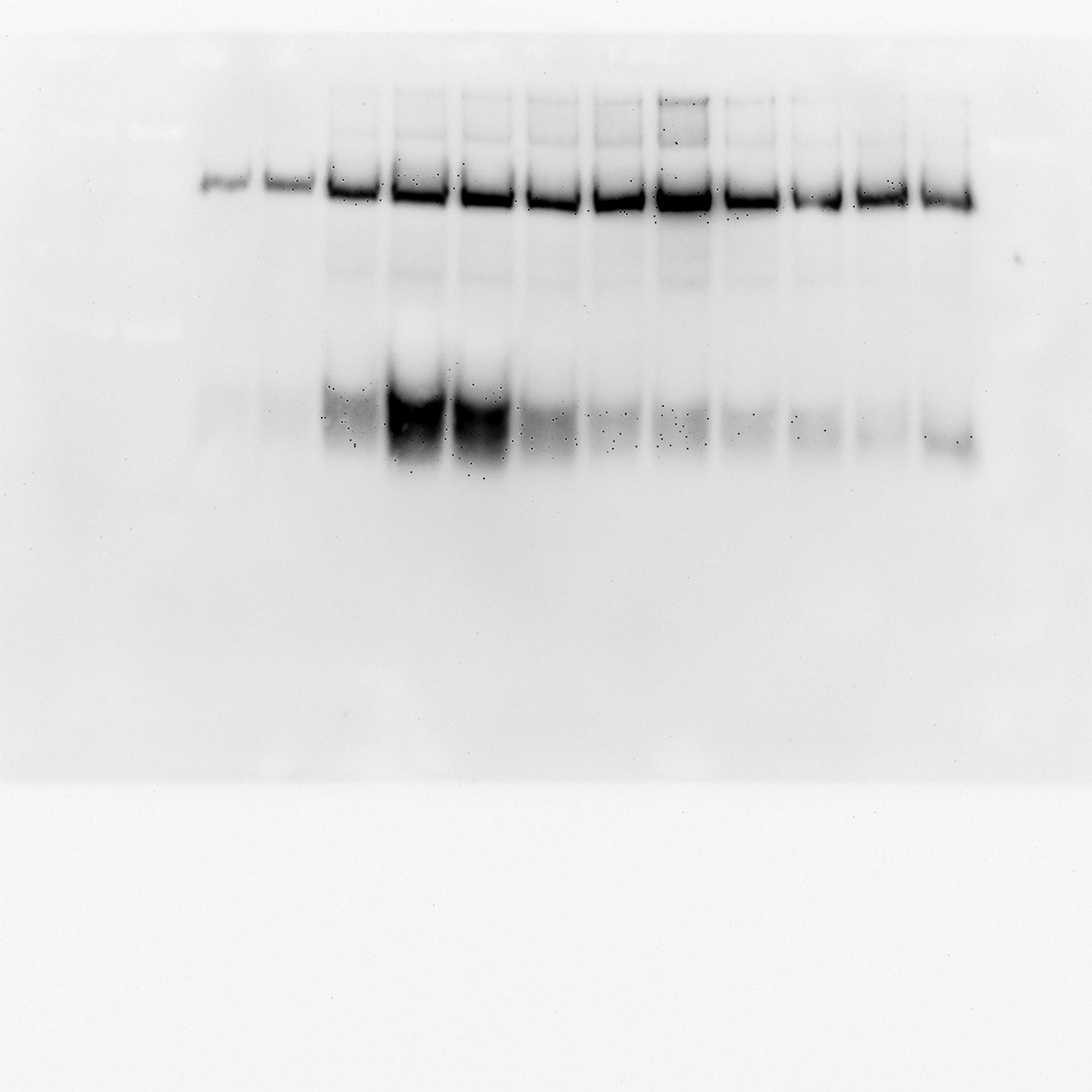

Supplement: Figure 5—source data 1. — A, B contain uncropped western blots shown in Figure 5A and B. C contains uncropped western blots of seven biological replicates showing that HDL presence renders Scube2 function obsolete. Prizm file C quantifies similar relative Shh release from nt Ctrl cells in the presence of HDL, irrespective of Scube2 presence or absence (as shown in Figure 5C). The Excel file contains raw data of Figure 5A’, A’’, B’ and B’’. [file elife-86920-fig5-data1.zip › Figure_5_Source_Data_1 /B''_GF_Gel 2_Shh+HDL_antiShh rabbit.jpg]

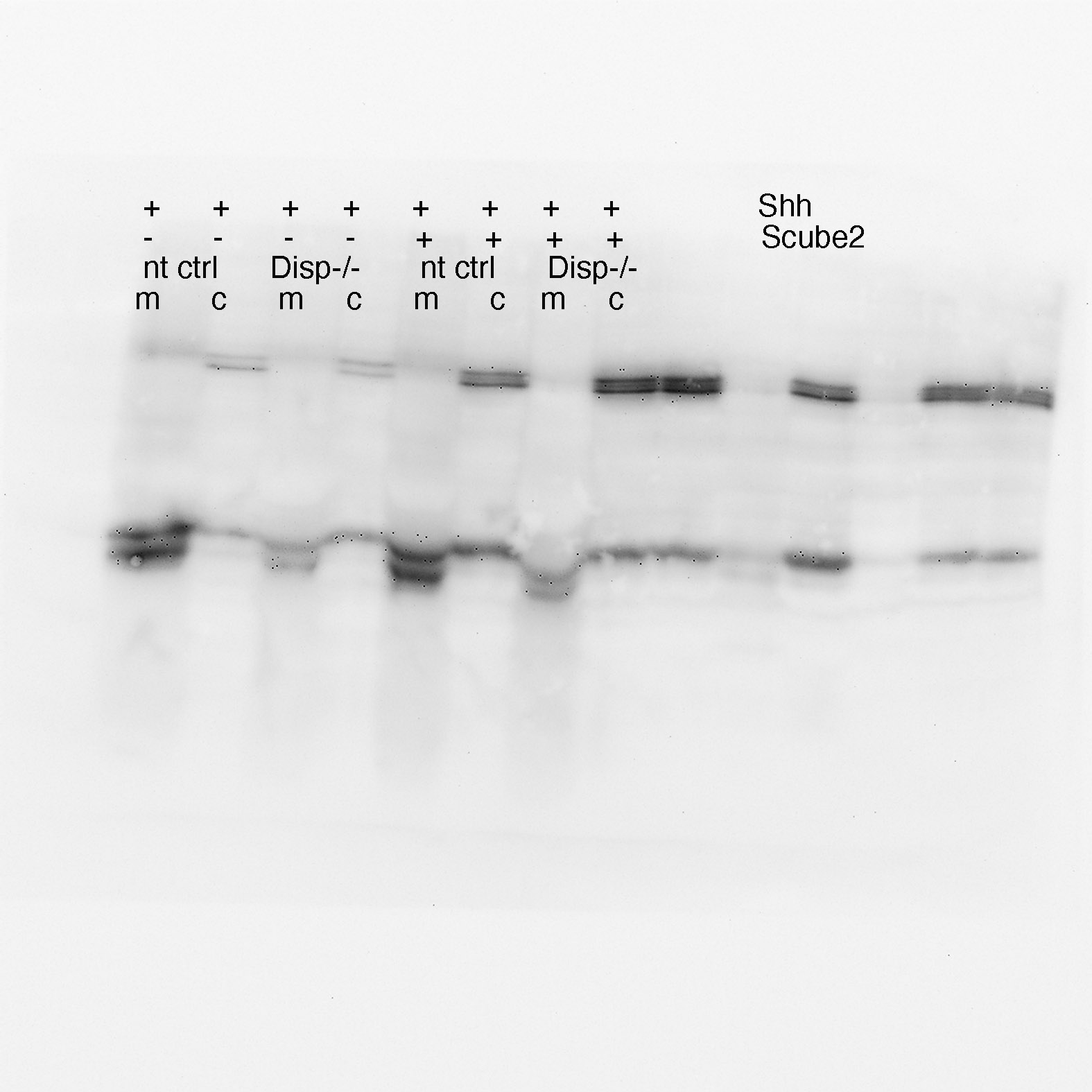

Supplement: Figure 5—source data 1. — A, B contain uncropped western blots shown in Figure 5A and B. C contains uncropped western blots of seven biological replicates showing that HDL presence renders Scube2 function obsolete. Prizm file C quantifies similar relative Shh release from nt Ctrl cells in the presence of HDL, irrespective of Scube2 presence or absence (as shown in Figure 5C). The Excel file contains raw data of Figure 5A’, A’’, B’ and B’’. [file elife-86920-fig5-data1.zip › Figure_5_Source_Data_1 /B_V753_2_1min labelled.jpg]

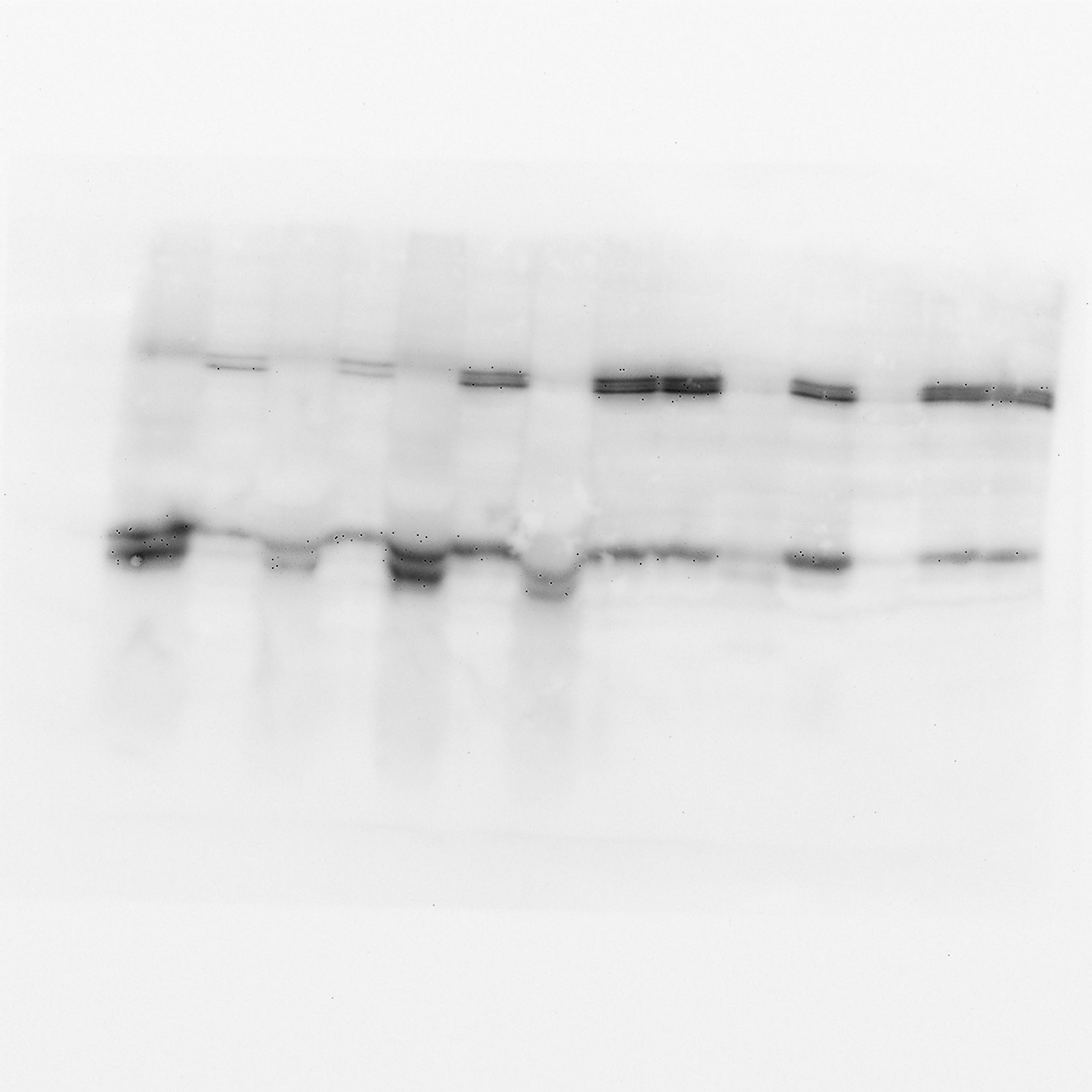

Supplement: Figure 5—source data 1. — A, B contain uncropped western blots shown in Figure 5A and B. C contains uncropped western blots of seven biological replicates showing that HDL presence renders Scube2 function obsolete. Prizm file C quantifies similar relative Shh release from nt Ctrl cells in the presence of HDL, irrespective of Scube2 presence or absence (as shown in Figure 5C). The Excel file contains raw data of Figure 5A’, A’’, B’ and B’’. [file elife-86920-fig5-data1.zip › Figure_5_Source_Data_1 /B_V753_2_1min.jpg]

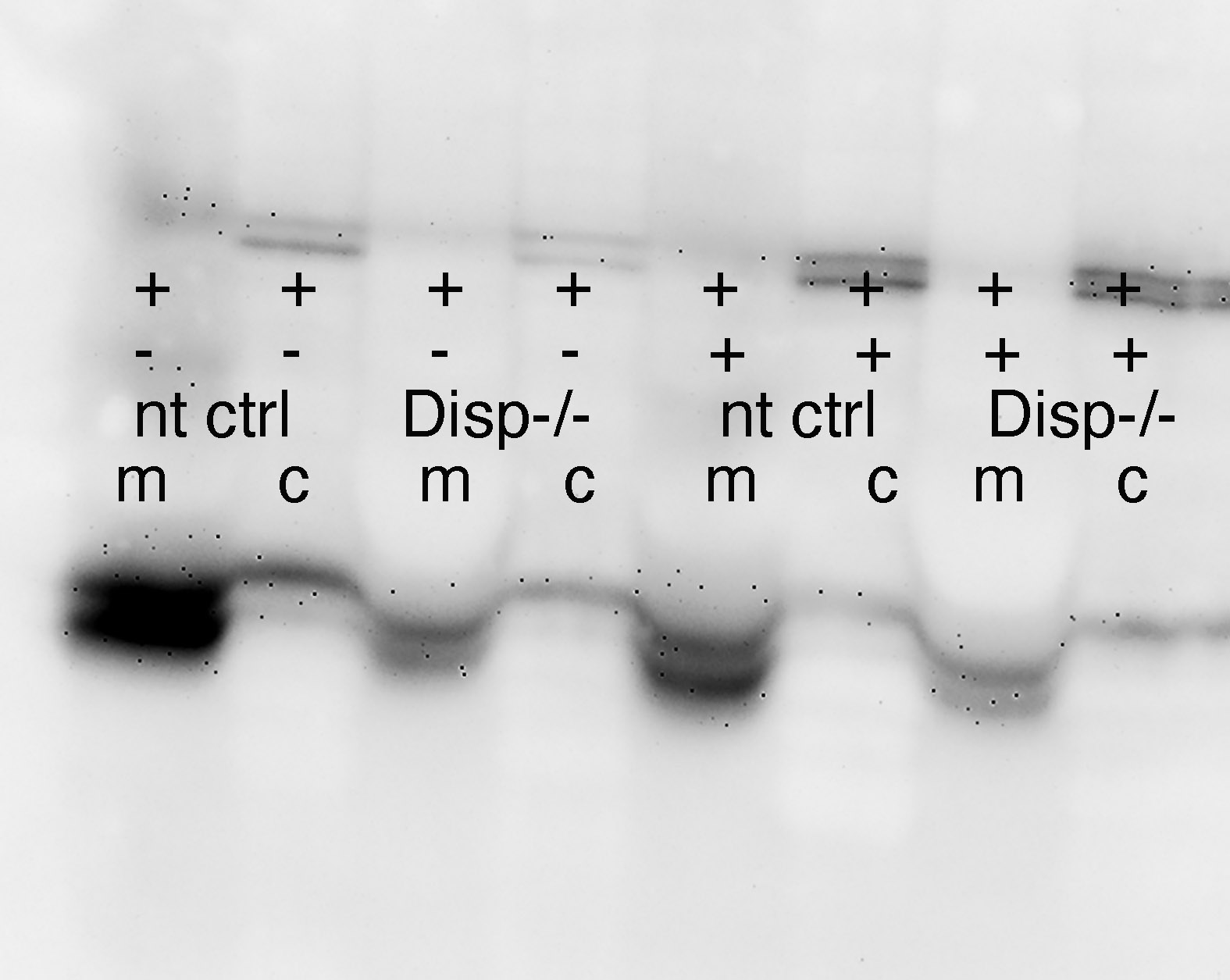

Supplement: Figure 5—source data 1. — A, B contain uncropped western blots shown in Figure 5A and B. C contains uncropped western blots of seven biological replicates showing that HDL presence renders Scube2 function obsolete. Prizm file C quantifies similar relative Shh release from nt Ctrl cells in the presence of HDL, irrespective of Scube2 presence or absence (as shown in Figure 5C). The Excel file contains raw data of Figure 5A’, A’’, B’ and B’’. [file elife-86920-fig5-data1.zip › Figure_5_Source_Data_1 /C_raw_blot_1_H7 HDL 1 labelled.jpg]

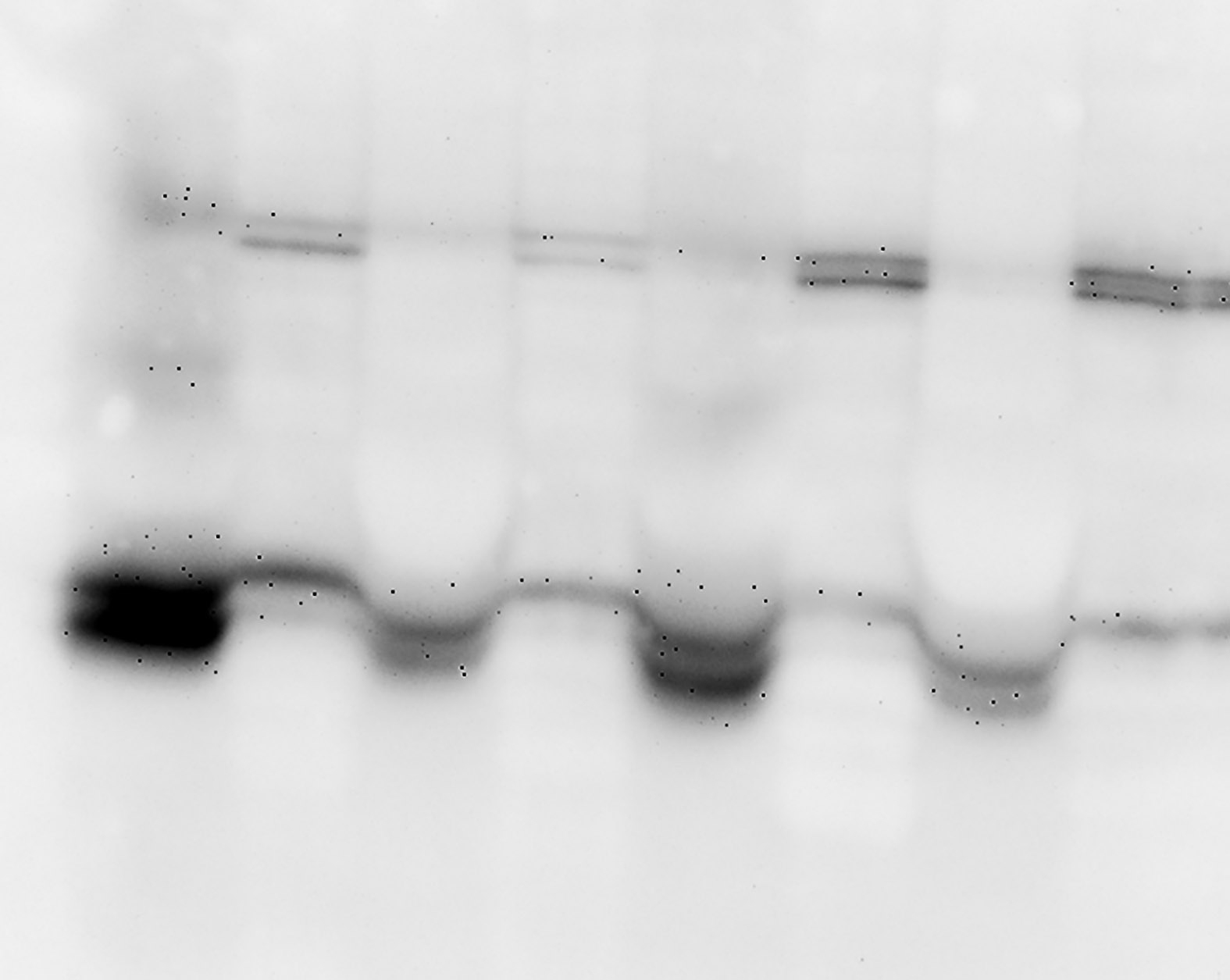

Supplement: Figure 5—source data 1. — A, B contain uncropped western blots shown in Figure 5A and B. C contains uncropped western blots of seven biological replicates showing that HDL presence renders Scube2 function obsolete. Prizm file C quantifies similar relative Shh release from nt Ctrl cells in the presence of HDL, irrespective of Scube2 presence or absence (as shown in Figure 5C). The Excel file contains raw data of Figure 5A’, A’’, B’ and B’’. [file elife-86920-fig5-data1.zip › Figure_5_Source_Data_1 /C_raw_blot_1_H7 HDL 1.jpg]

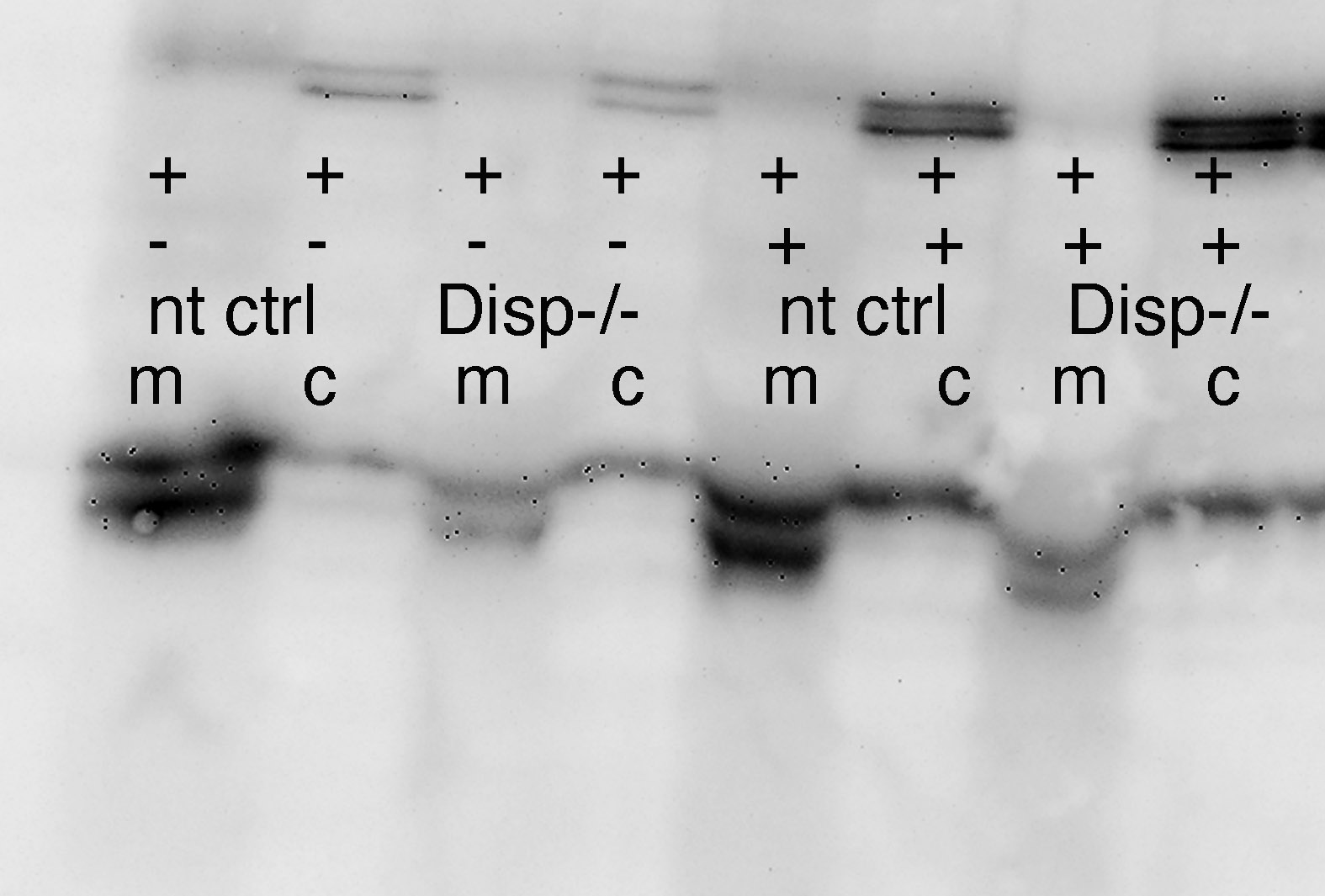

Supplement: Figure 5—source data 1. — A, B contain uncropped western blots shown in Figure 5A and B. C contains uncropped western blots of seven biological replicates showing that HDL presence renders Scube2 function obsolete. Prizm file C quantifies similar relative Shh release from nt Ctrl cells in the presence of HDL, irrespective of Scube2 presence or absence (as shown in Figure 5C). The Excel file contains raw data of Figure 5A’, A’’, B’ and B’’. [file elife-86920-fig5-data1.zip › Figure_5_Source_Data_1 /C_raw_blot_2_H7 HDL 2 labelled.jpg]

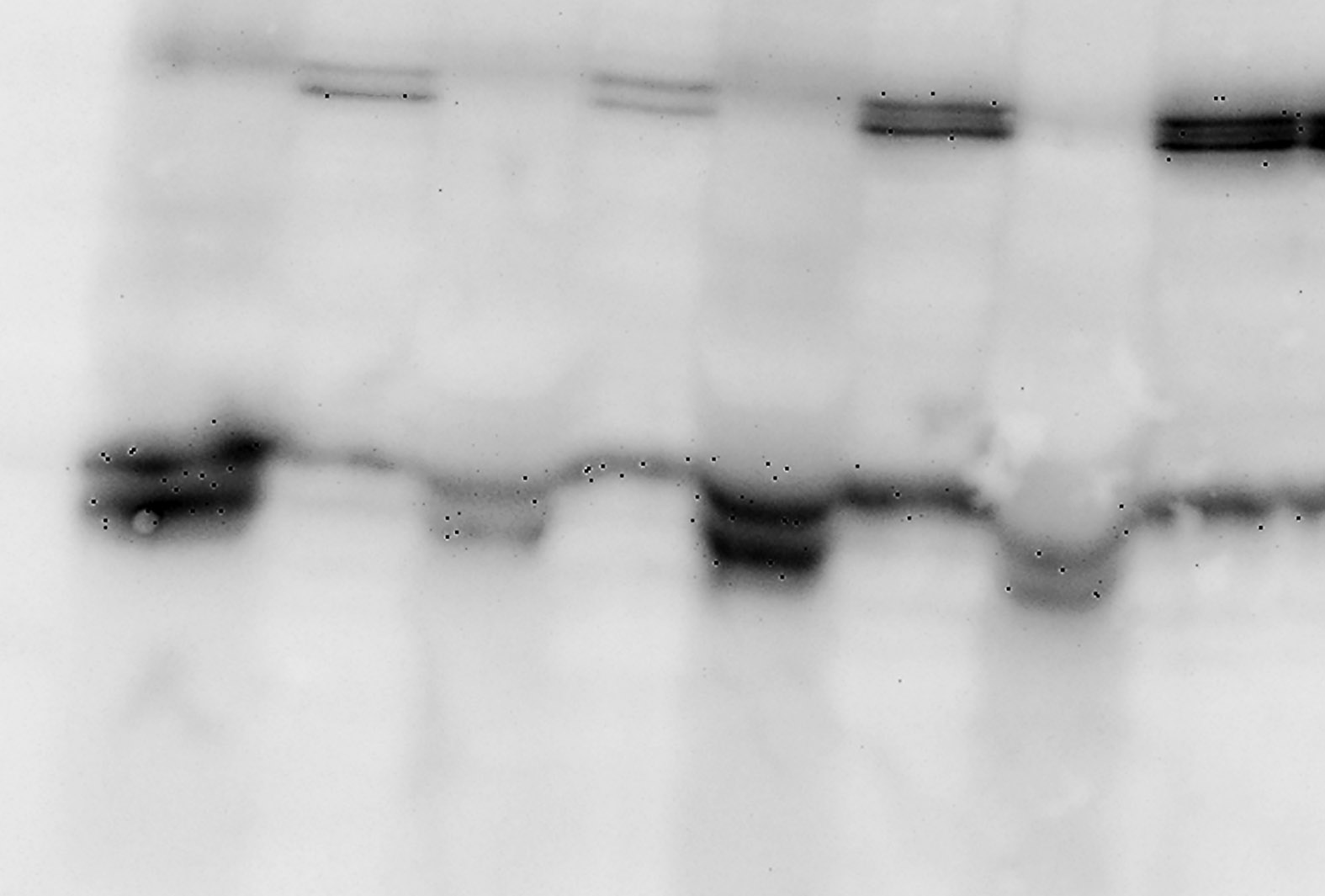

Supplement: Figure 5—source data 1. — A, B contain uncropped western blots shown in Figure 5A and B. C contains uncropped western blots of seven biological replicates showing that HDL presence renders Scube2 function obsolete. Prizm file C quantifies similar relative Shh release from nt Ctrl cells in the presence of HDL, irrespective of Scube2 presence or absence (as shown in Figure 5C). The Excel file contains raw data of Figure 5A’, A’’, B’ and B’’. [file elife-86920-fig5-data1.zip › Figure_5_Source_Data_1 /C_raw_blot_2_H7 HDL 2.jpg]

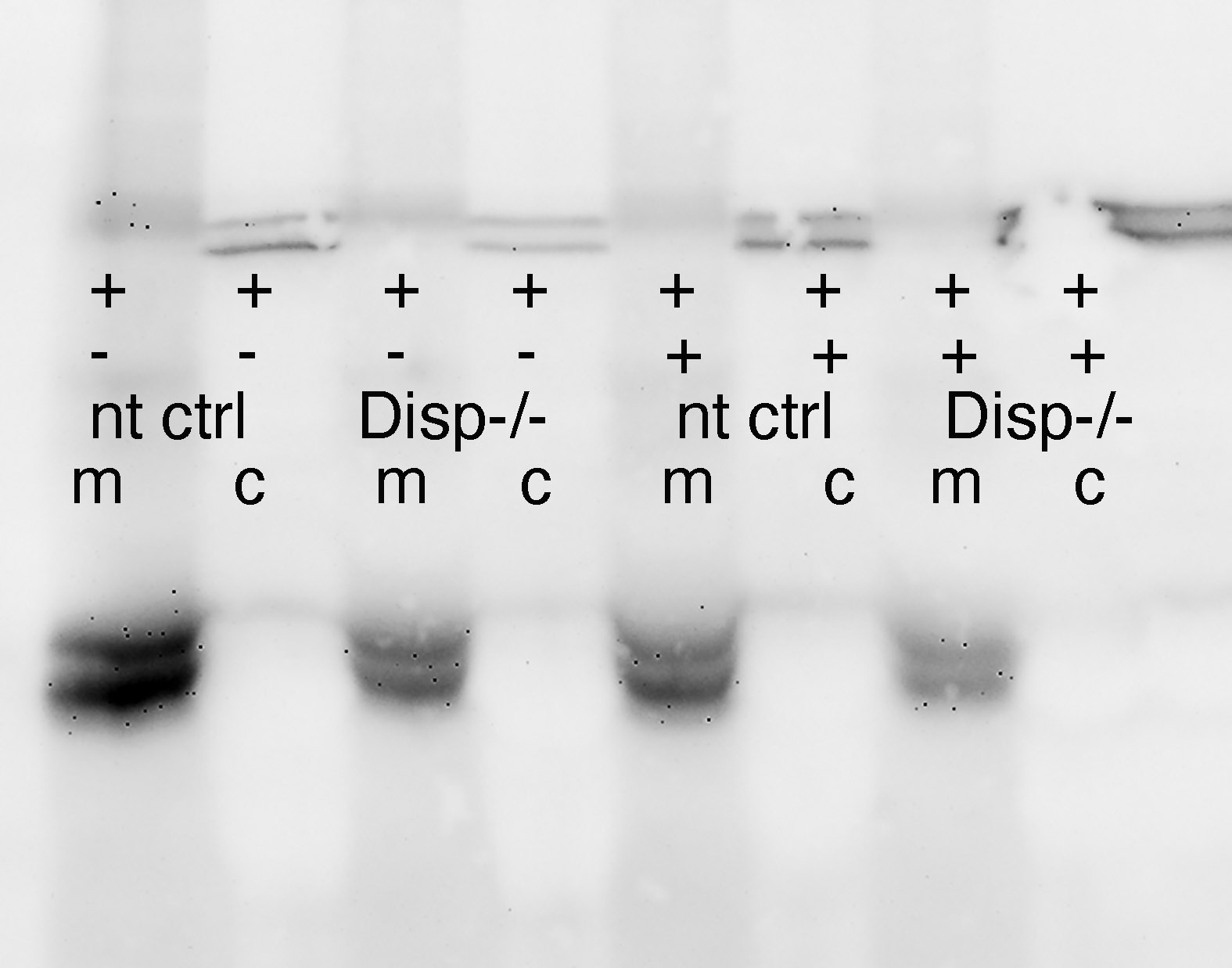

Supplement: Figure 5—source data 1. — A, B contain uncropped western blots shown in Figure 5A and B. C contains uncropped western blots of seven biological replicates showing that HDL presence renders Scube2 function obsolete. Prizm file C quantifies similar relative Shh release from nt Ctrl cells in the presence of HDL, irrespective of Scube2 presence or absence (as shown in Figure 5C). The Excel file contains raw data of Figure 5A’, A’’, B’ and B’’. [file elife-86920-fig5-data1.zip › Figure_5_Source_Data_1 /C_raw_blot_3_H7 HDL labelled.jpg]

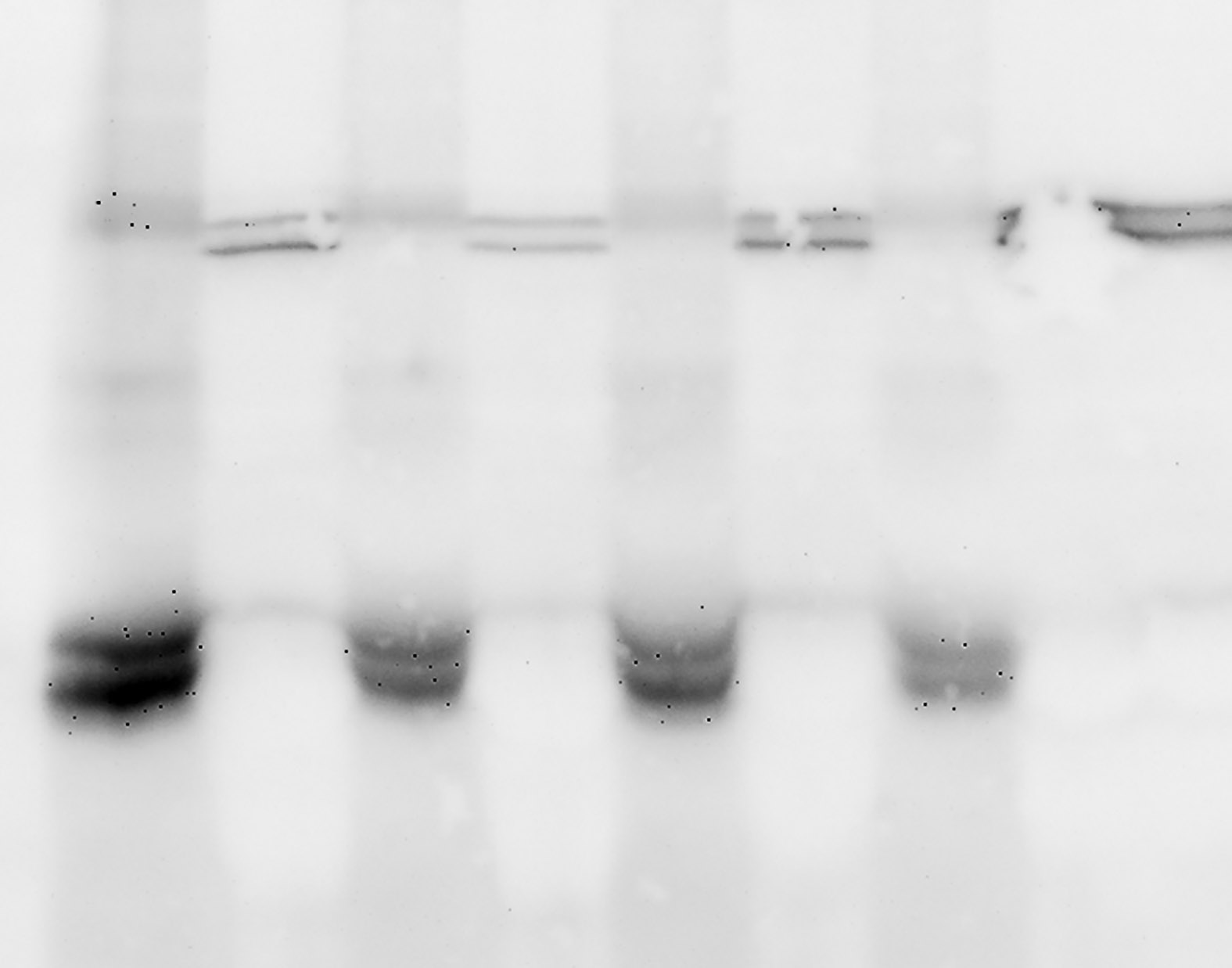

Supplement: Figure 5—source data 1. — A, B contain uncropped western blots shown in Figure 5A and B. C contains uncropped western blots of seven biological replicates showing that HDL presence renders Scube2 function obsolete. Prizm file C quantifies similar relative Shh release from nt Ctrl cells in the presence of HDL, irrespective of Scube2 presence or absence (as shown in Figure 5C). The Excel file contains raw data of Figure 5A’, A’’, B’ and B’’. [file elife-86920-fig5-data1.zip › Figure_5_Source_Data_1 /C_raw_blot_3_H7 HDL.jpg]

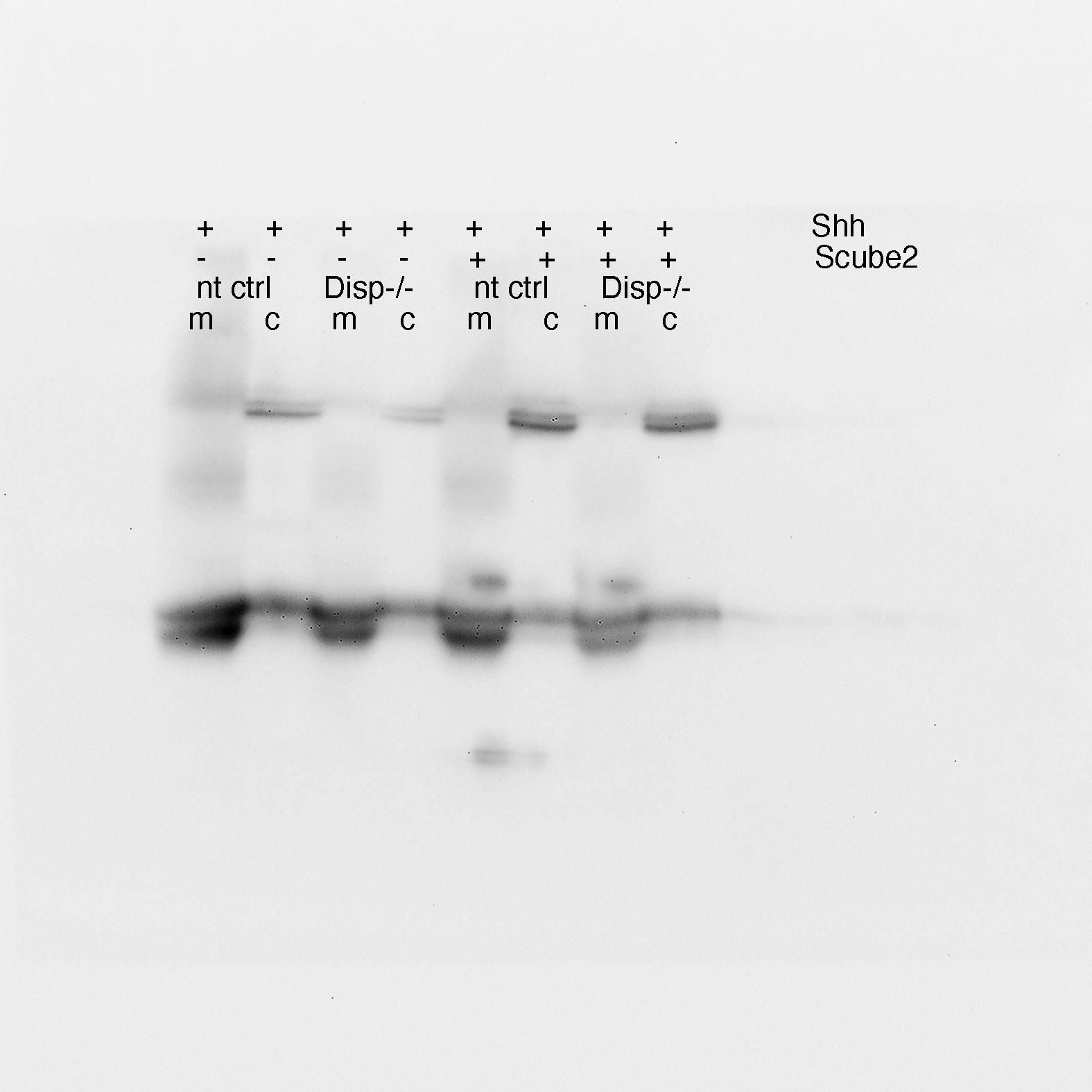

Supplement: Figure 5—source data 1. — A, B contain uncropped western blots shown in Figure 5A and B. C contains uncropped western blots of seven biological replicates showing that HDL presence renders Scube2 function obsolete. Prizm file C quantifies similar relative Shh release from nt Ctrl cells in the presence of HDL, irrespective of Scube2 presence or absence (as shown in Figure 5C). The Excel file contains raw data of Figure 5A’, A’’, B’ and B’’. [file elife-86920-fig5-data1.zip › Figure_5_Source_Data_1 /C_raw_blot_4_V752_2_Shh_30sec labelled.jpg]

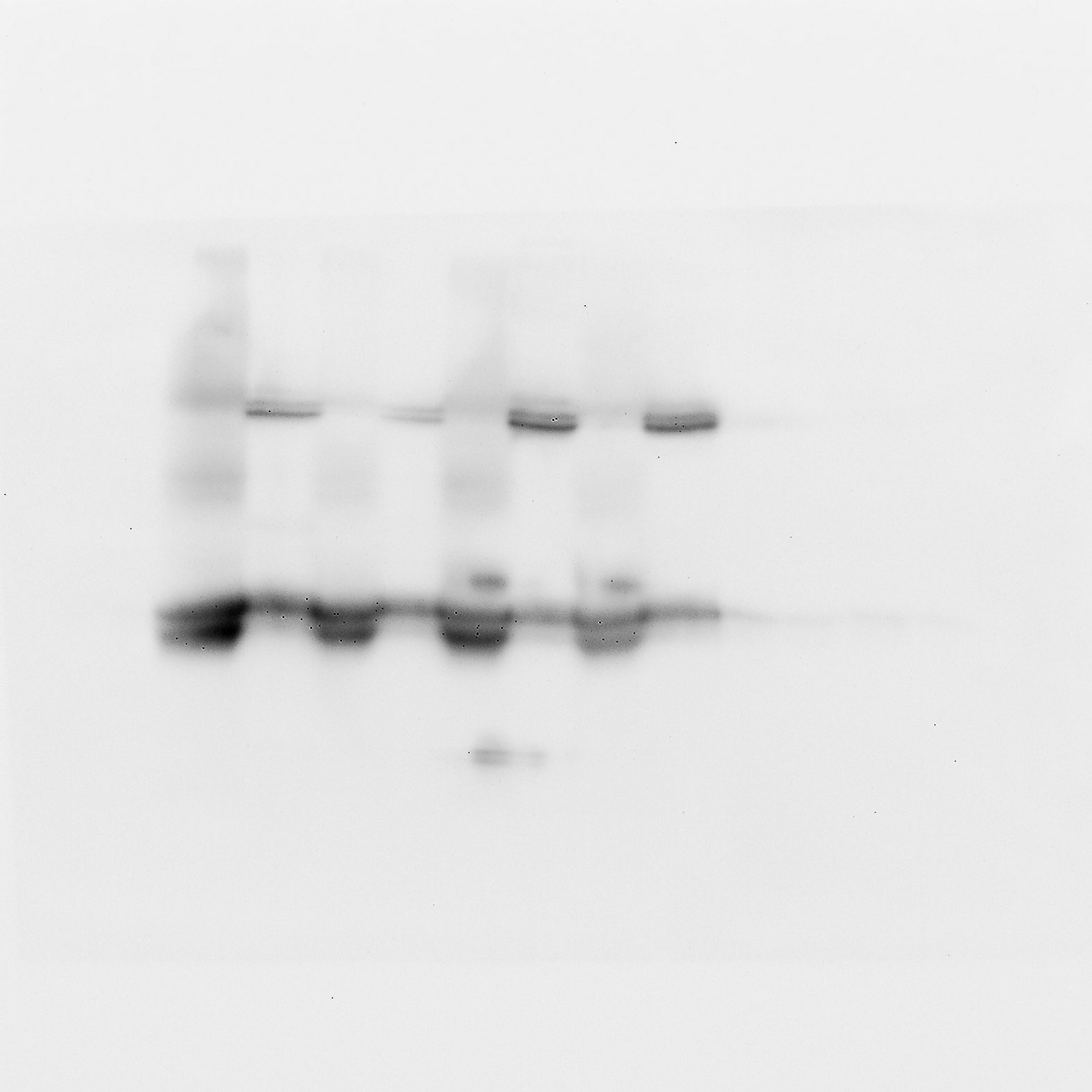

Supplement: Figure 5—source data 1. — A, B contain uncropped western blots shown in Figure 5A and B. C contains uncropped western blots of seven biological replicates showing that HDL presence renders Scube2 function obsolete. Prizm file C quantifies similar relative Shh release from nt Ctrl cells in the presence of HDL, irrespective of Scube2 presence or absence (as shown in Figure 5C). The Excel file contains raw data of Figure 5A’, A’’, B’ and B’’. [file elife-86920-fig5-data1.zip › Figure_5_Source_Data_1 /C_raw_blot_4_V752_2_Shh_30sec.jpg]

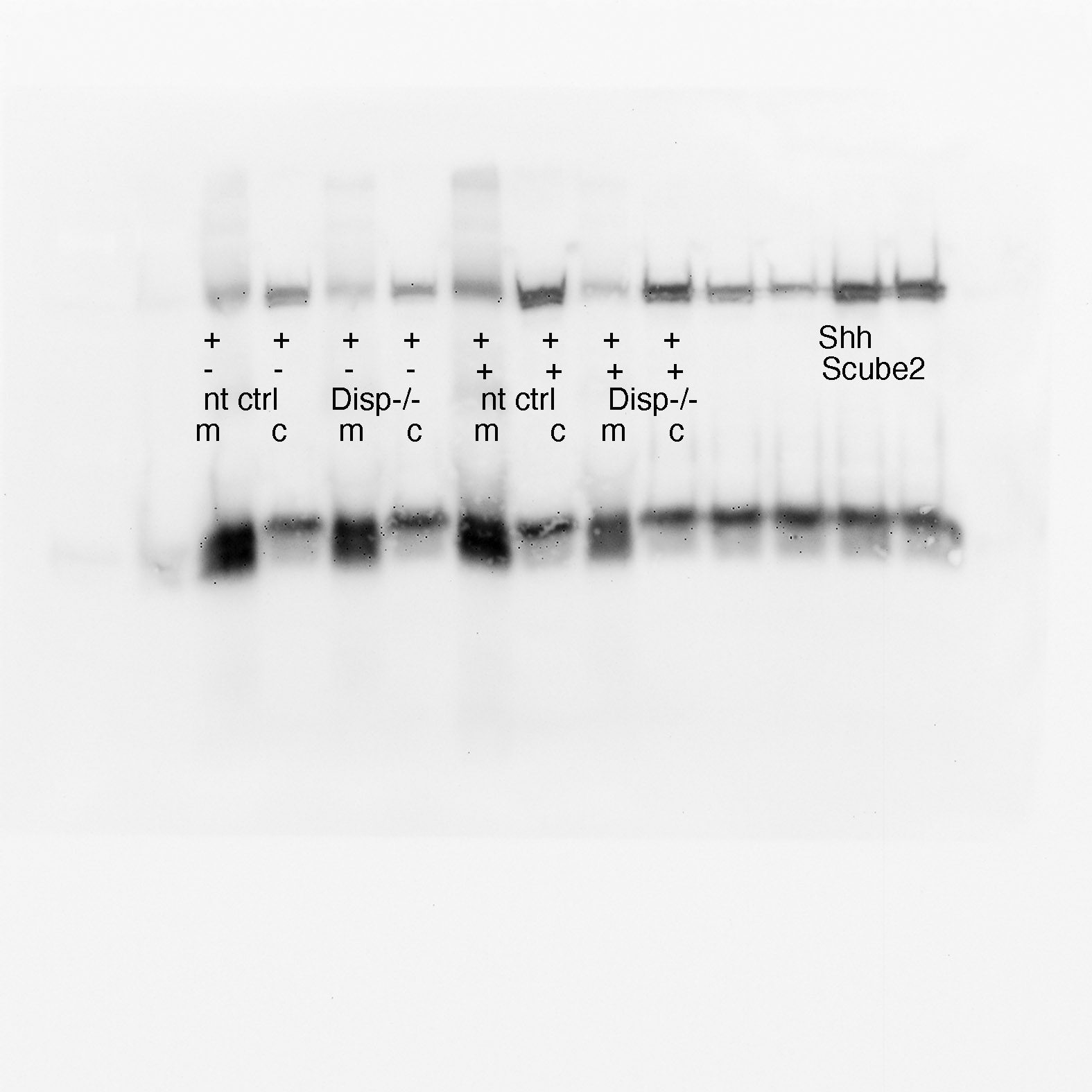

Supplement: Figure 5—source data 1. — A, B contain uncropped western blots shown in Figure 5A and B. C contains uncropped western blots of seven biological replicates showing that HDL presence renders Scube2 function obsolete. Prizm file C quantifies similar relative Shh release from nt Ctrl cells in the presence of HDL, irrespective of Scube2 presence or absence (as shown in Figure 5C). The Excel file contains raw data of Figure 5A’, A’’, B’ and B’’. [file elife-86920-fig5-data1.zip › Figure_5_Source_Data_1 /C_raw_blot_5_V787_Shh_4_7sec labelled.jpg]

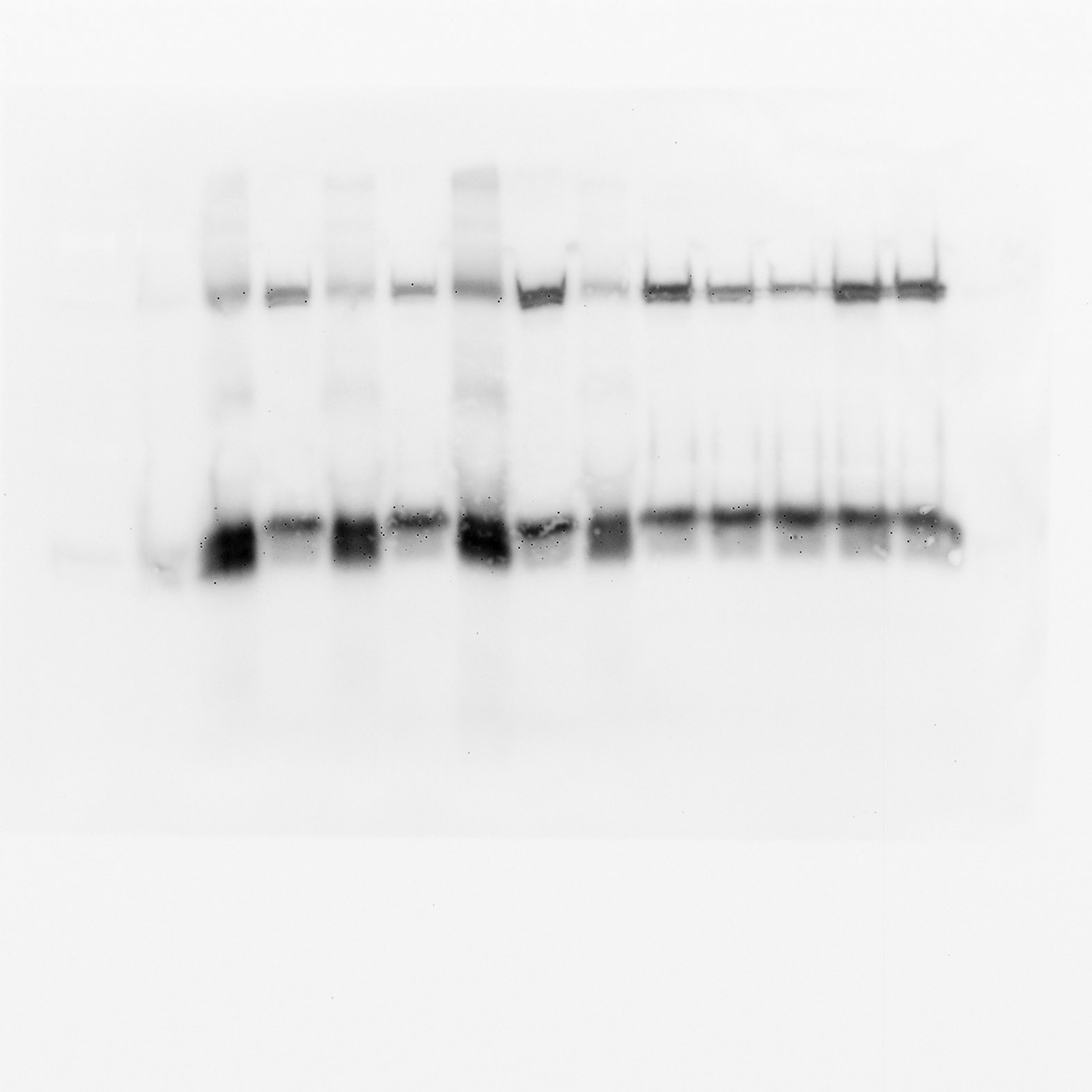

Supplement: Figure 5—source data 1. — A, B contain uncropped western blots shown in Figure 5A and B. C contains uncropped western blots of seven biological replicates showing that HDL presence renders Scube2 function obsolete. Prizm file C quantifies similar relative Shh release from nt Ctrl cells in the presence of HDL, irrespective of Scube2 presence or absence (as shown in Figure 5C). The Excel file contains raw data of Figure 5A’, A’’, B’ and B’’. [file elife-86920-fig5-data1.zip › Figure_5_Source_Data_1 /C_raw_blot_5_V787_Shh_4_7sec.jpg]

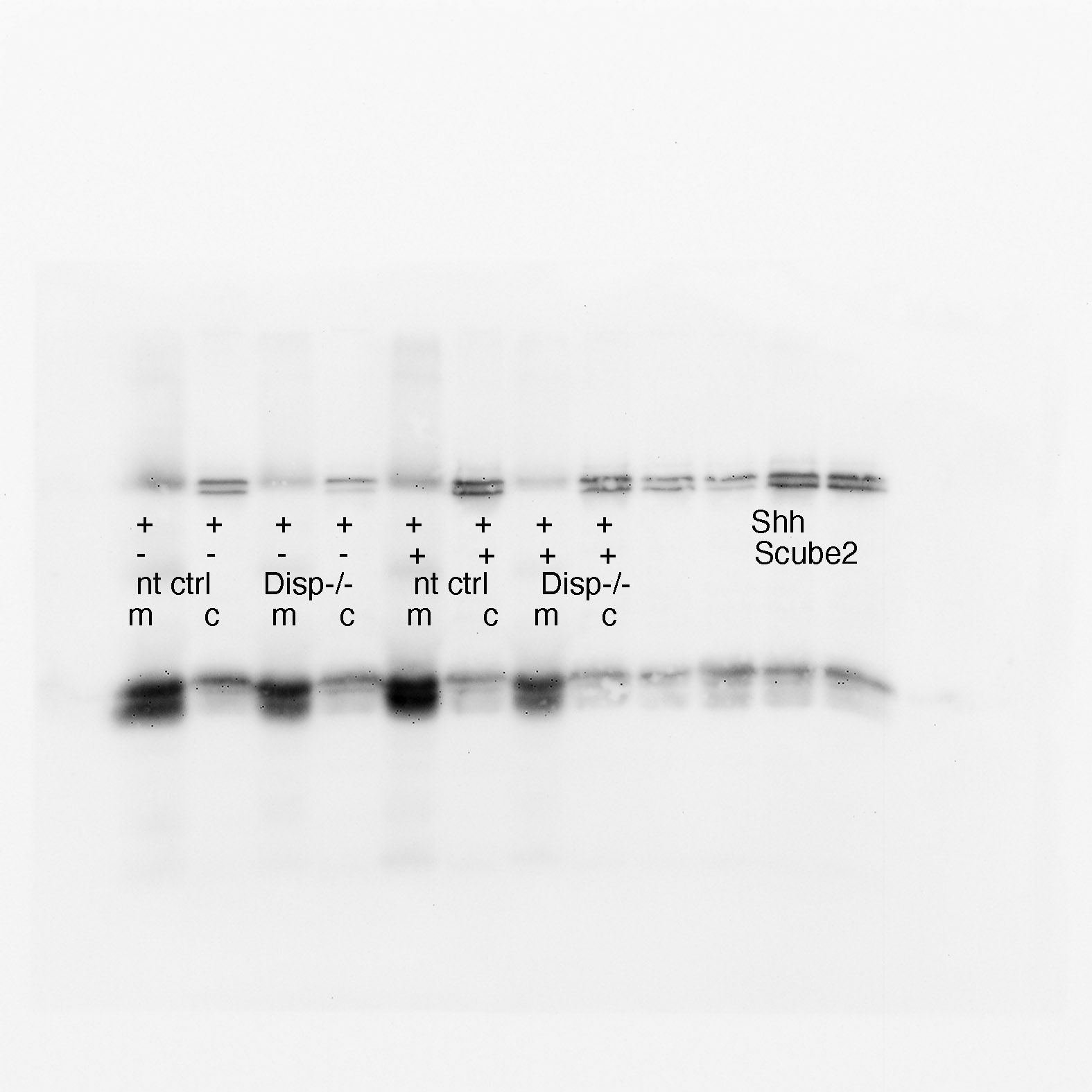

Supplement: Figure 5—source data 1. — A, B contain uncropped western blots shown in Figure 5A and B. C contains uncropped western blots of seven biological replicates showing that HDL presence renders Scube2 function obsolete. Prizm file C quantifies similar relative Shh release from nt Ctrl cells in the presence of HDL, irrespective of Scube2 presence or absence (as shown in Figure 5C). The Excel file contains raw data of Figure 5A’, A’’, B’ and B’’. [file elife-86920-fig5-data1.zip › Figure_5_Source_Data_1 /C_raw_blot_6_V787_Shh_5_7sec labelled.jpg]

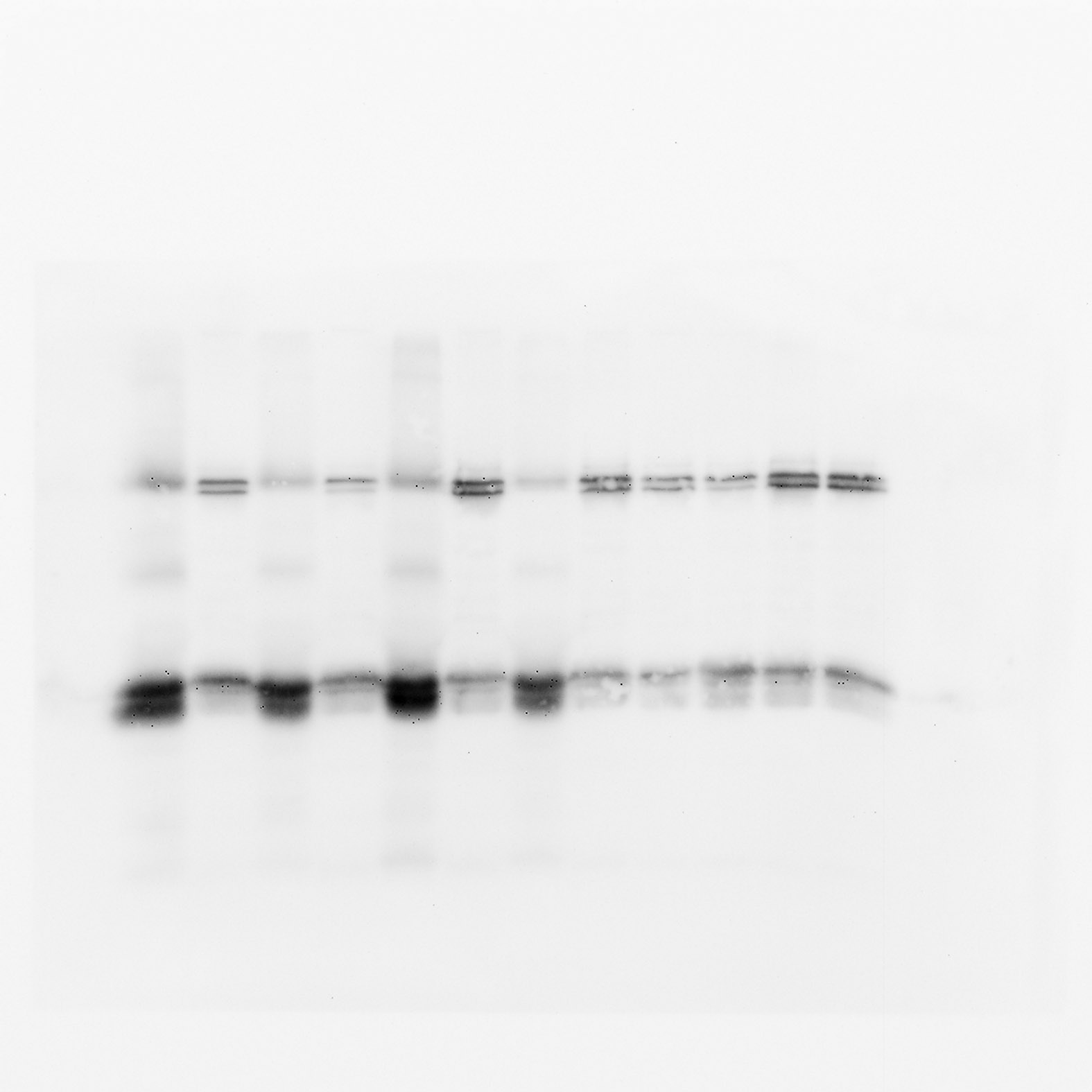

Supplement: Figure 5—source data 1. — A, B contain uncropped western blots shown in Figure 5A and B. C contains uncropped western blots of seven biological replicates showing that HDL presence renders Scube2 function obsolete. Prizm file C quantifies similar relative Shh release from nt Ctrl cells in the presence of HDL, irrespective of Scube2 presence or absence (as shown in Figure 5C). The Excel file contains raw data of Figure 5A’, A’’, B’ and B’’. [file elife-86920-fig5-data1.zip › Figure_5_Source_Data_1 /C_raw_blot_6_V787_Shh_5_7sec.jpg]

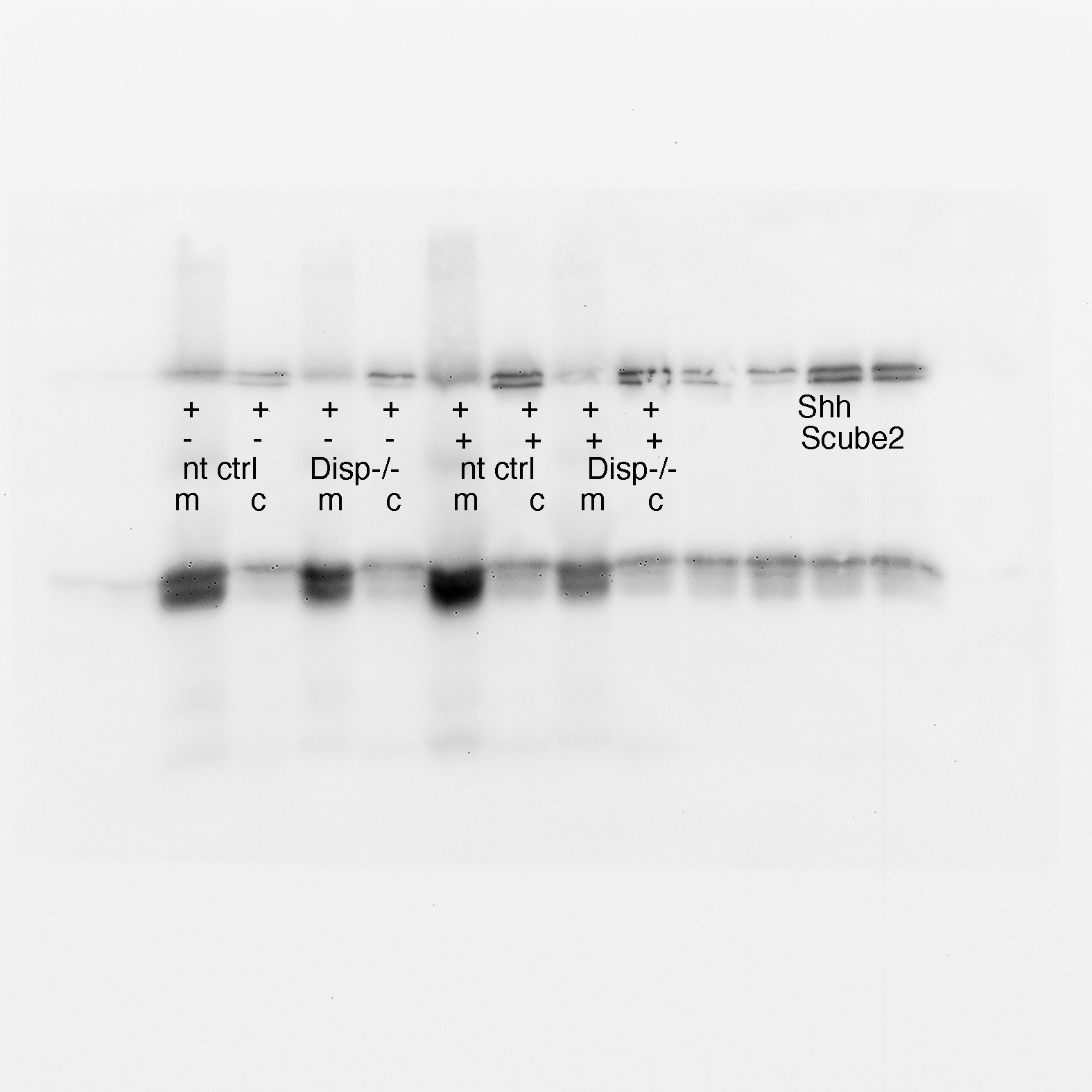

Supplement: Figure 5—source data 1. — A, B contain uncropped western blots shown in Figure 5A and B. C contains uncropped western blots of seven biological replicates showing that HDL presence renders Scube2 function obsolete. Prizm file C quantifies similar relative Shh release from nt Ctrl cells in the presence of HDL, irrespective of Scube2 presence or absence (as shown in Figure 5C). The Excel file contains raw data of Figure 5A’, A’’, B’ and B’’. [file elife-86920-fig5-data1.zip › Figure_5_Source_Data_1 /C_raw_blot_7_V787_Shh_6_4sec labelled.jpg]

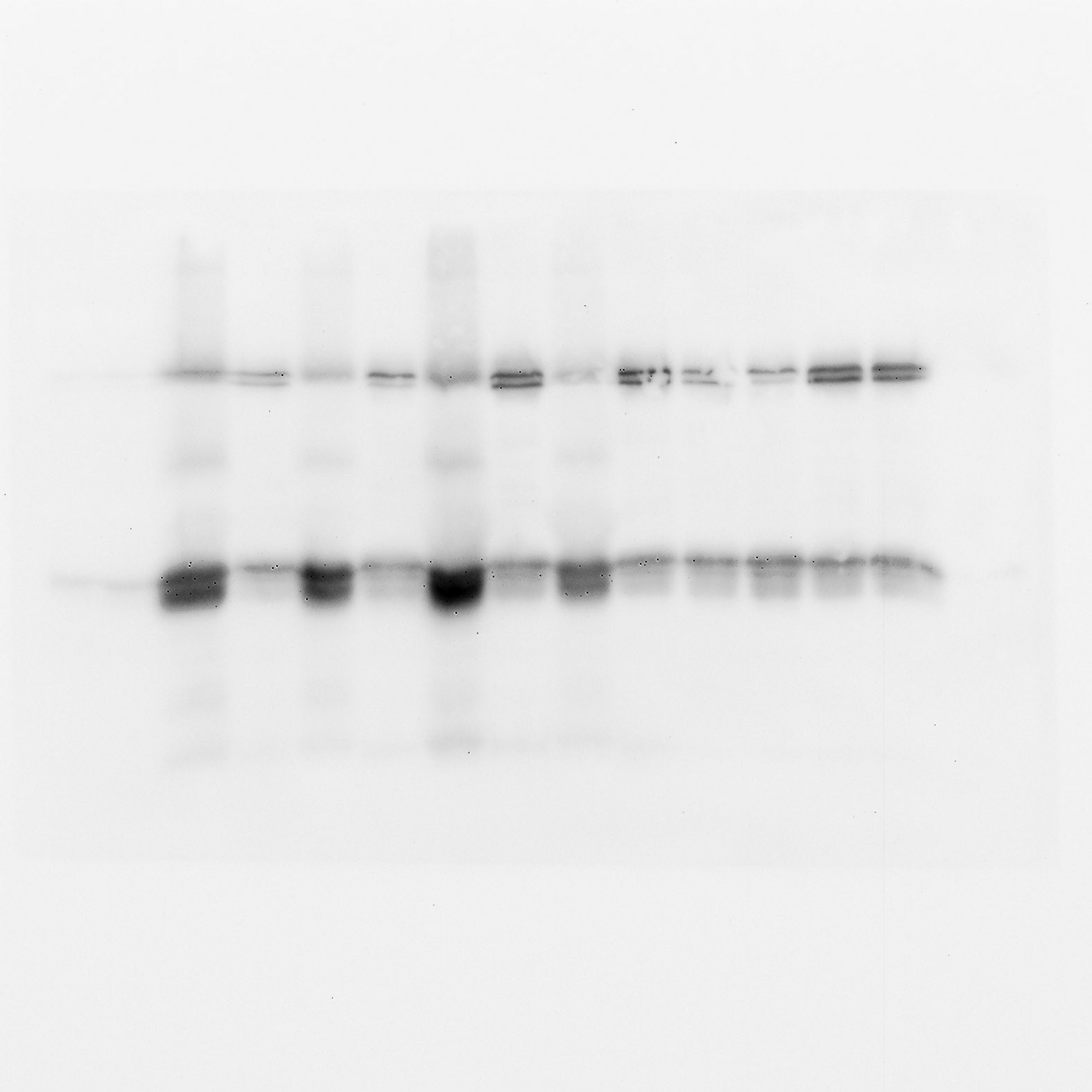

Supplement: Figure 5—source data 1. — A, B contain uncropped western blots shown in Figure 5A and B. C contains uncropped western blots of seven biological replicates showing that HDL presence renders Scube2 function obsolete. Prizm file C quantifies similar relative Shh release from nt Ctrl cells in the presence of HDL, irrespective of Scube2 presence or absence (as shown in Figure 5C). The Excel file contains raw data of Figure 5A’, A’’, B’ and B’’. [file elife-86920-fig5-data1.zip › Figure_5_Source_Data_1 /C_raw_blot_7_V787_Shh_6_4sec.jpg]

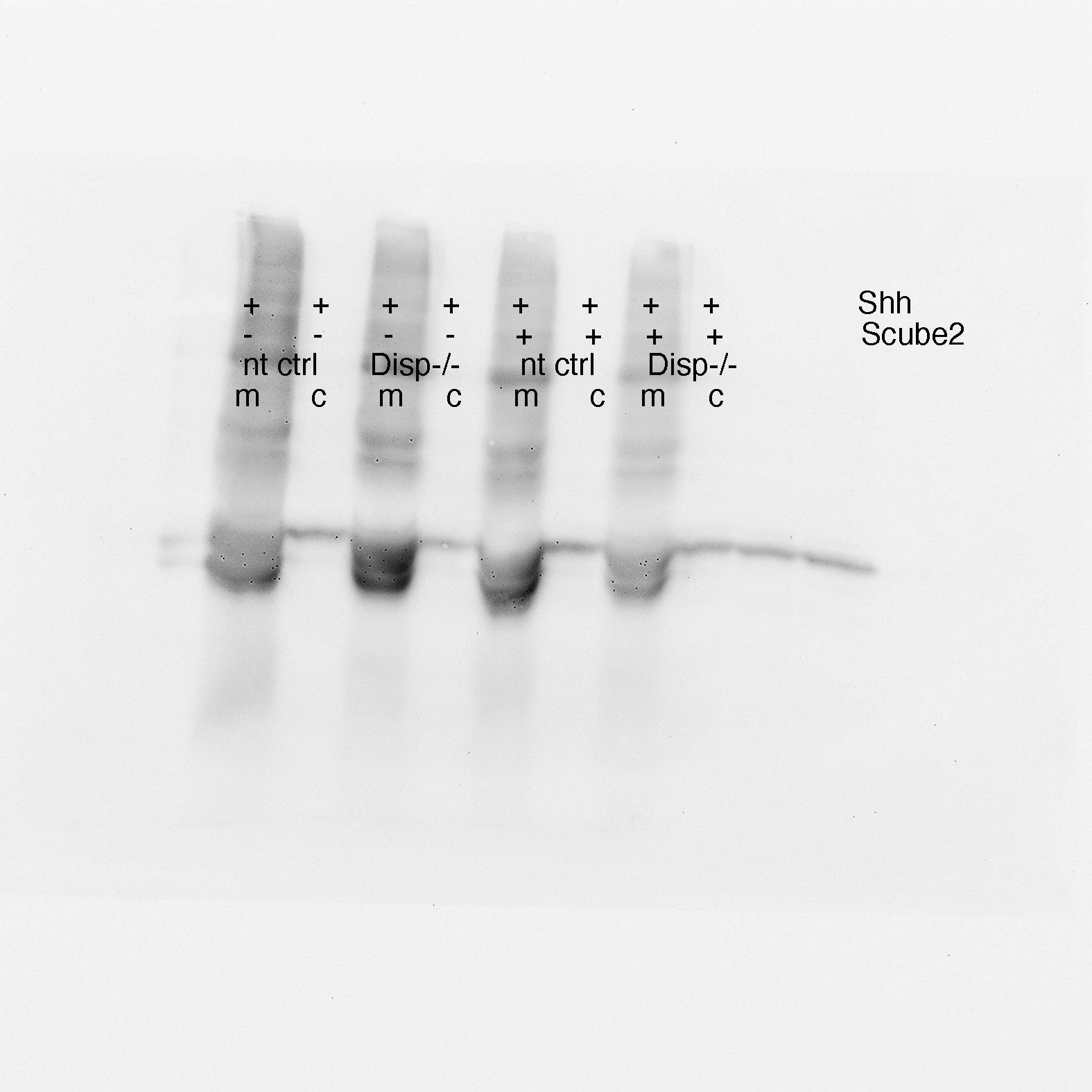

Supplement: Figure 7—source data 1. — A, B contain uncropped western blots shown in Figure 7A and B. The Excel file contains raw data used in Figure 7A’–E. [file elife-86920-fig7-data1.zip › Figure_7_Source_Data_1 /A_V757_9_aShh labelled.jpg]

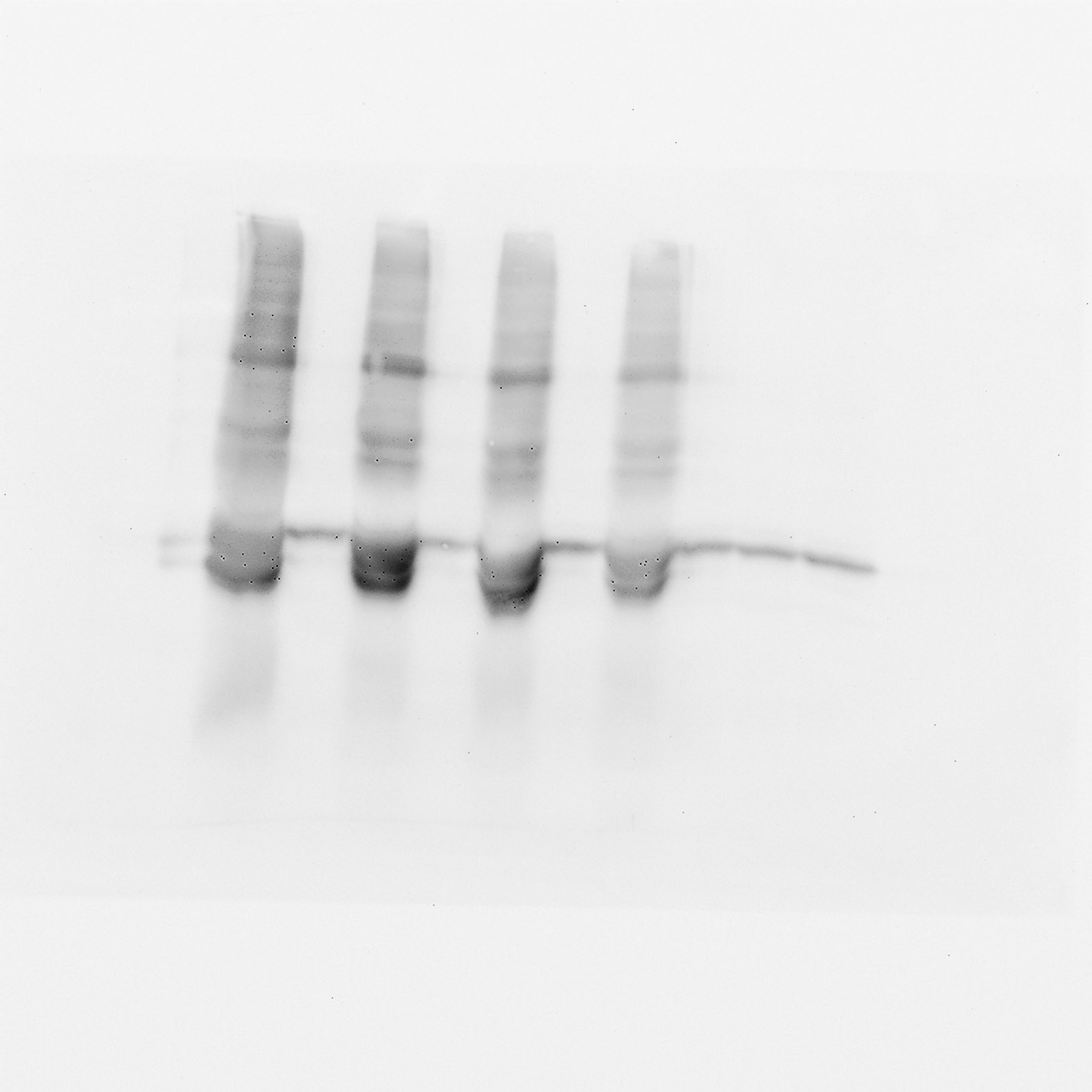

Supplement: Figure 7—source data 1. — A, B contain uncropped western blots shown in Figure 7A and B. The Excel file contains raw data used in Figure 7A’–E. [file elife-86920-fig7-data1.zip › Figure_7_Source_Data_1 /A_V757_9_aShh.jpg]

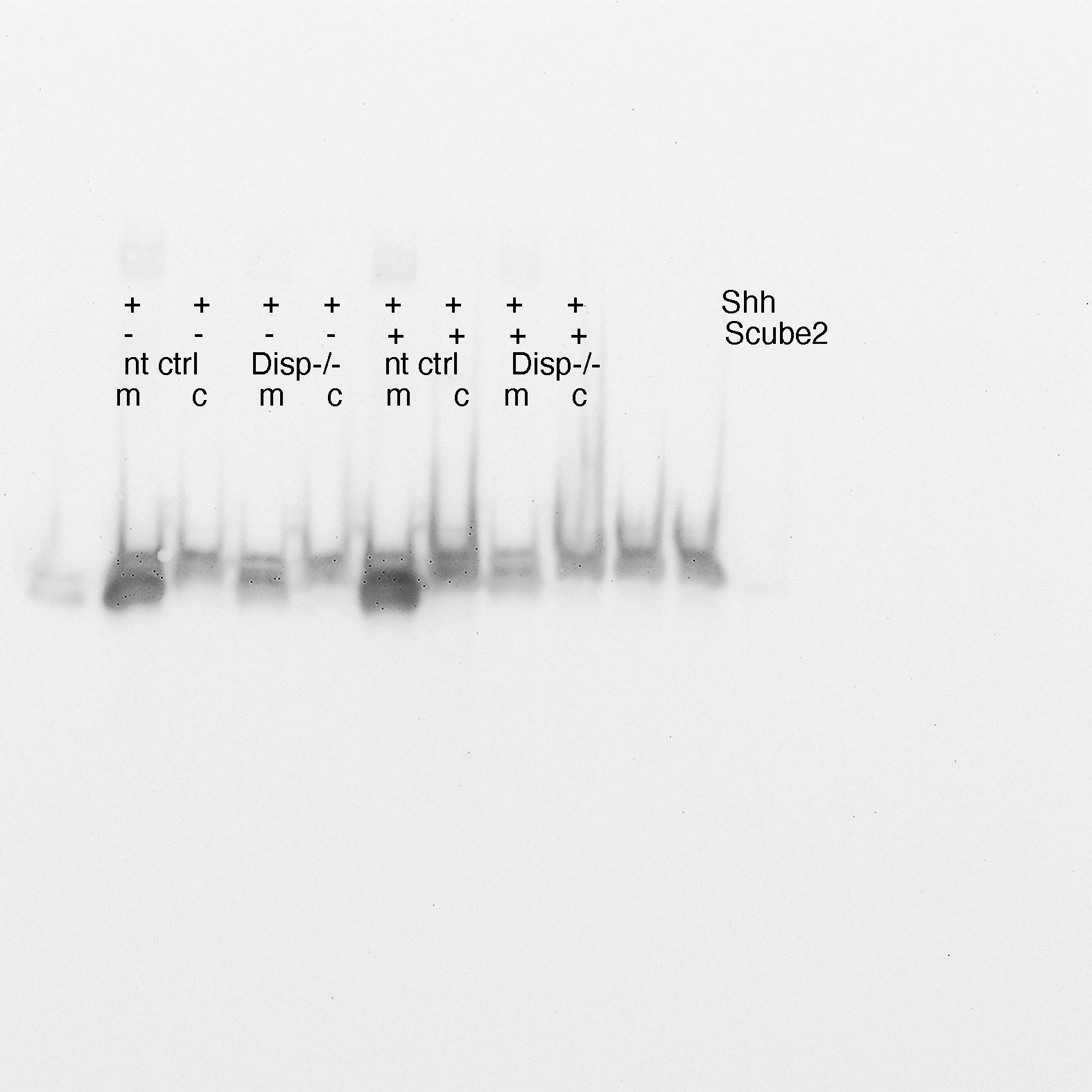

Supplement: Figure 7—source data 1. — A, B contain uncropped western blots shown in Figure 7A and B. The Excel file contains raw data used in Figure 7A’–E. [file elife-86920-fig7-data1.zip › Figure_7_Source_Data_1 /A_V765_H18 labelled.jpg]

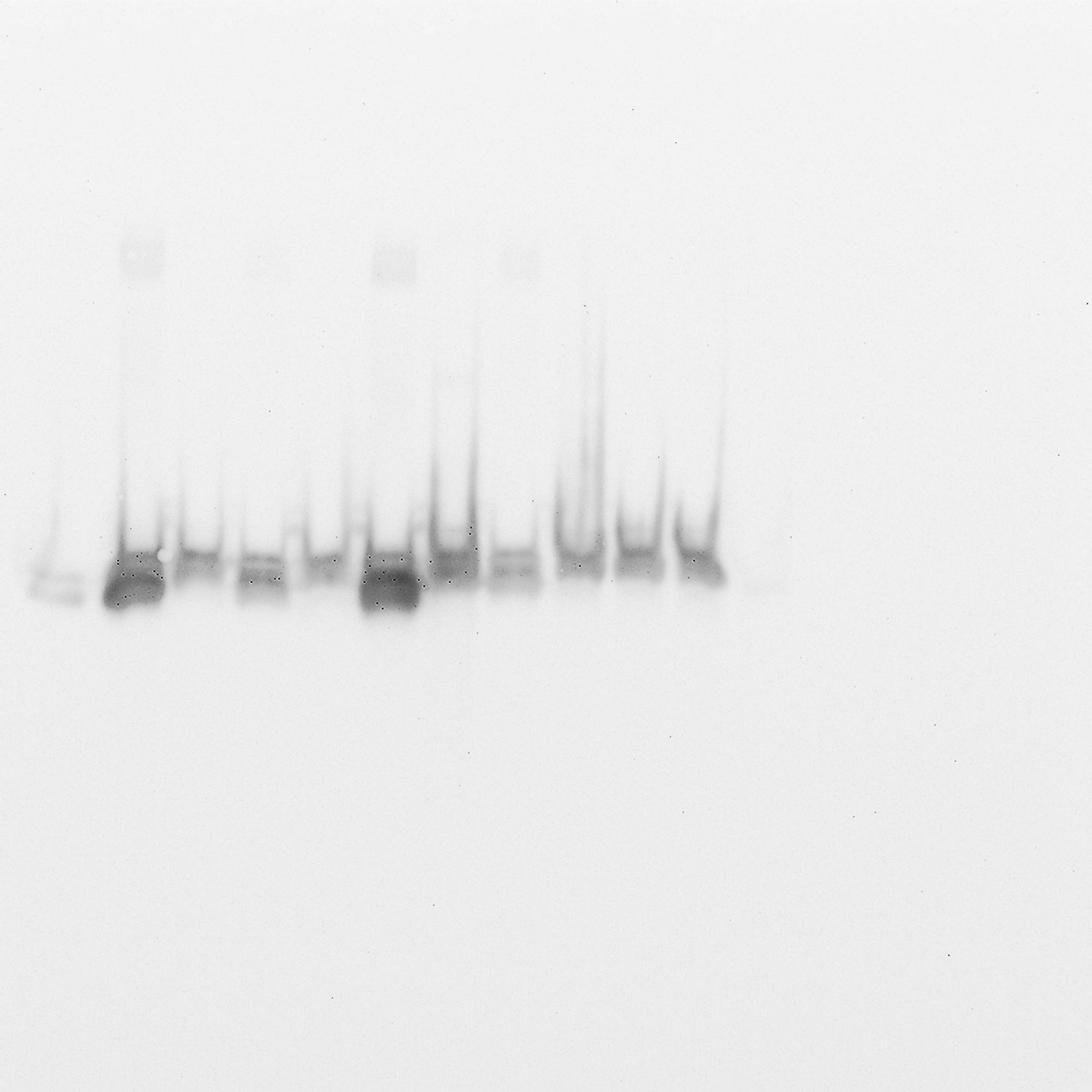

Supplement: Figure 7—source data 1. — A, B contain uncropped western blots shown in Figure 7A and B. The Excel file contains raw data used in Figure 7A’–E. [file elife-86920-fig7-data1.zip › Figure_7_Source_Data_1 /A_V765_H18.jpg]

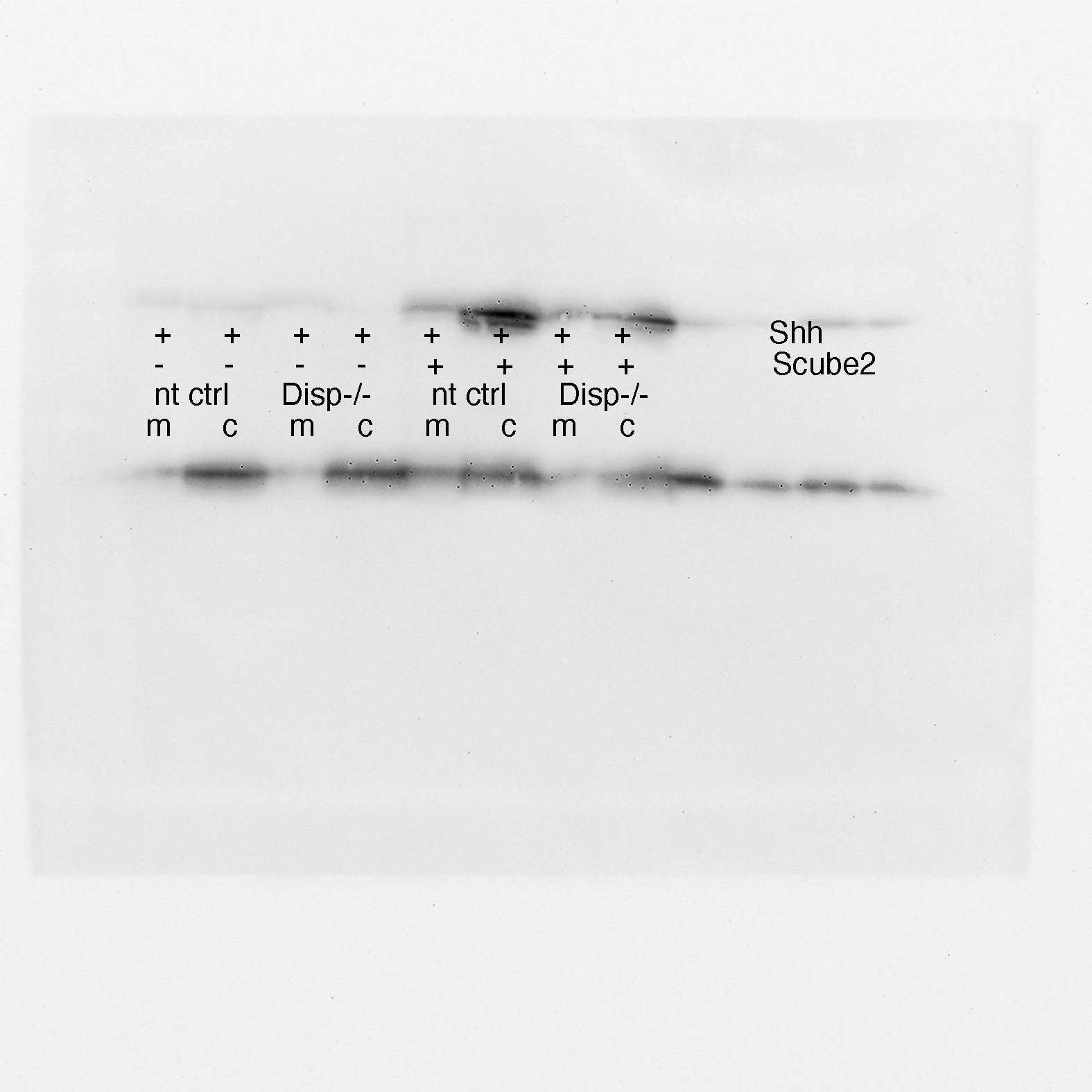

Supplement: Figure 7—source data 1. — A, B contain uncropped western blots shown in Figure 7A and B. The Excel file contains raw data used in Figure 7A’–E. [file elife-86920-fig7-data1.zip › Figure_7_Source_Data_1 /B_V752_4_Shh_ labelled.jpg]

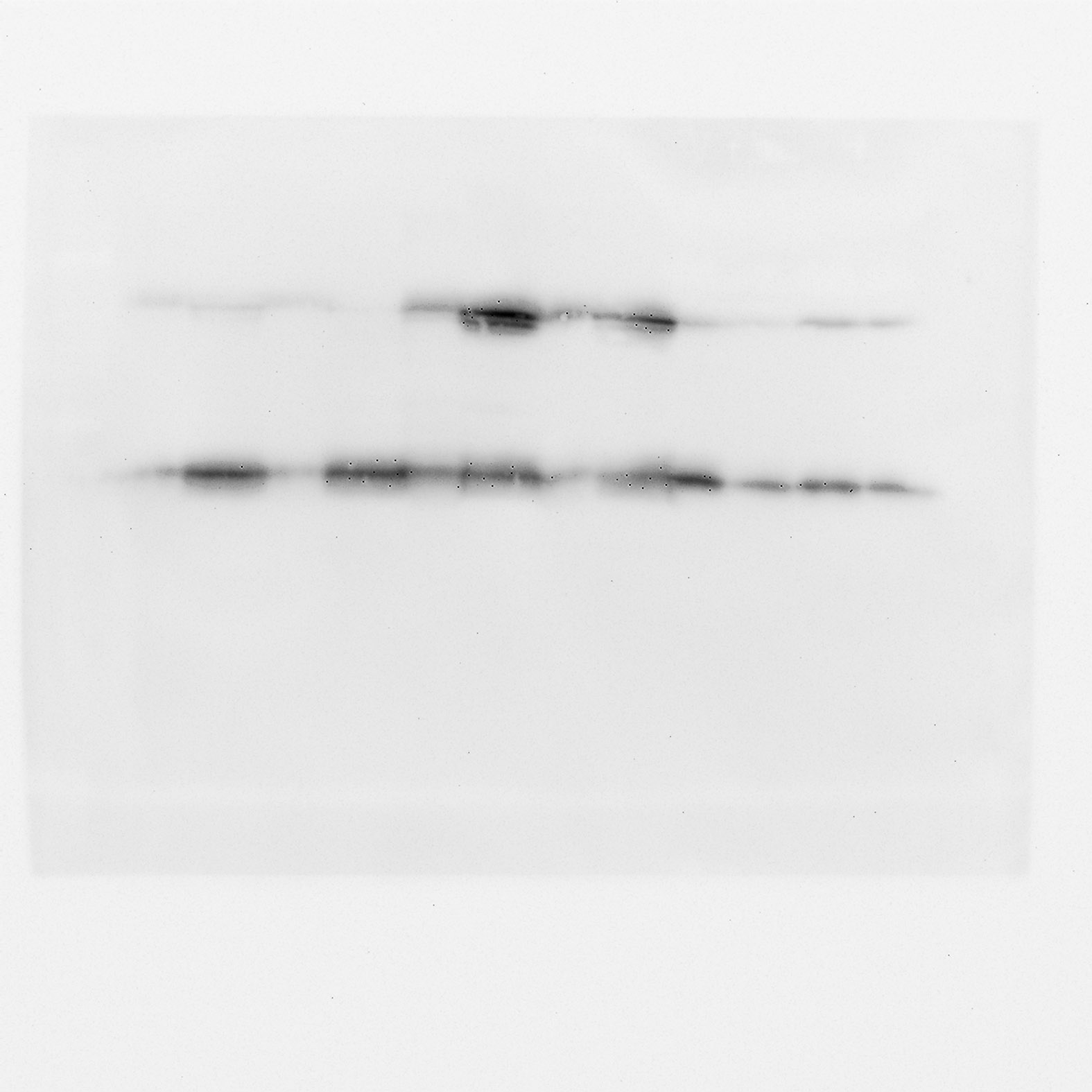

Supplement: Figure 7—source data 1. — A, B contain uncropped western blots shown in Figure 7A and B. The Excel file contains raw data used in Figure 7A’–E. [file elife-86920-fig7-data1.zip › Figure_7_Source_Data_1 /B_V752_4_Shh_.jpg]

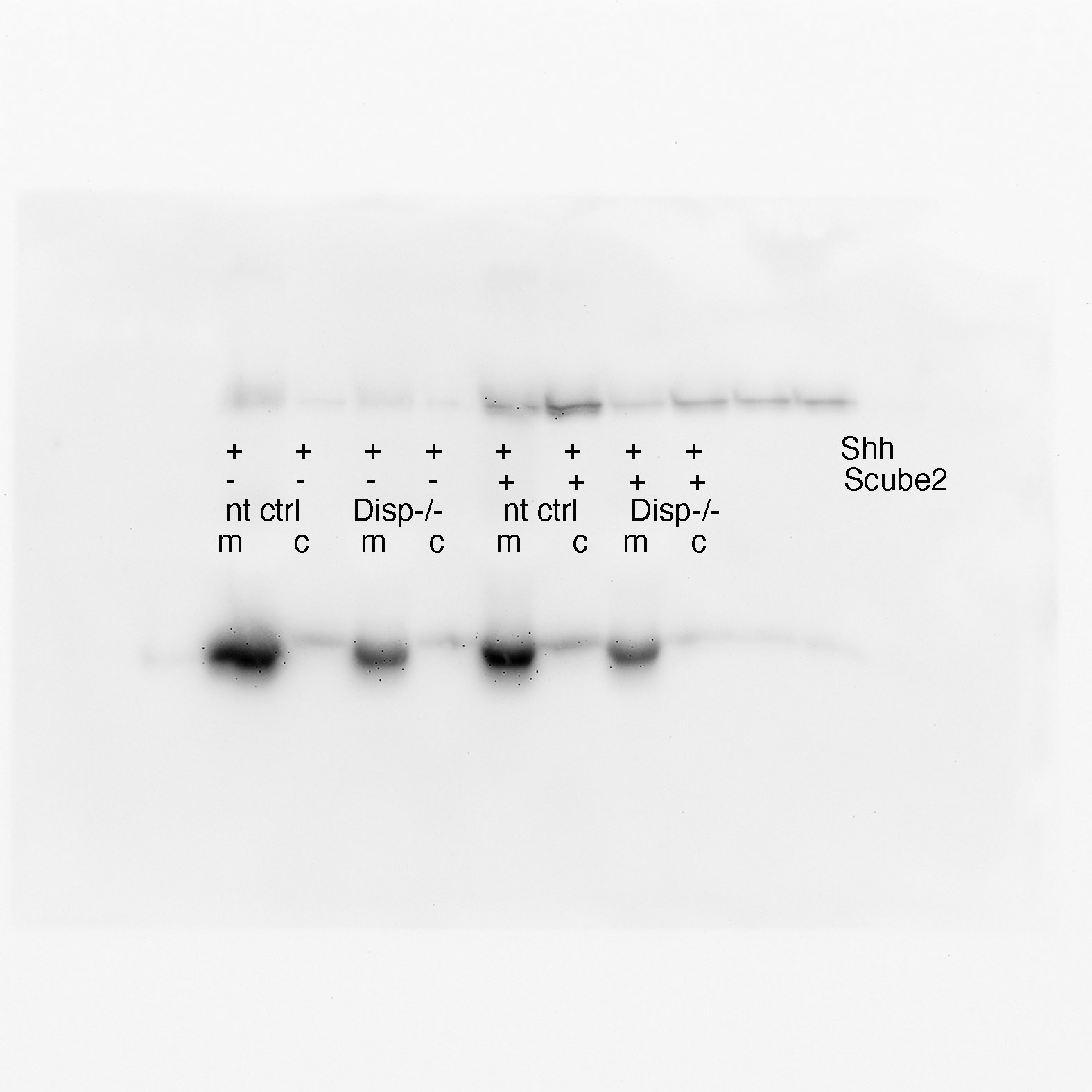

Supplement: Figure 7—source data 1. — A, B contain uncropped western blots shown in Figure 7A and B. The Excel file contains raw data used in Figure 7A’–E. [file elife-86920-fig7-data1.zip › Figure_7_Source_Data_1 /B_V760_2_Shh labelled.jpg]

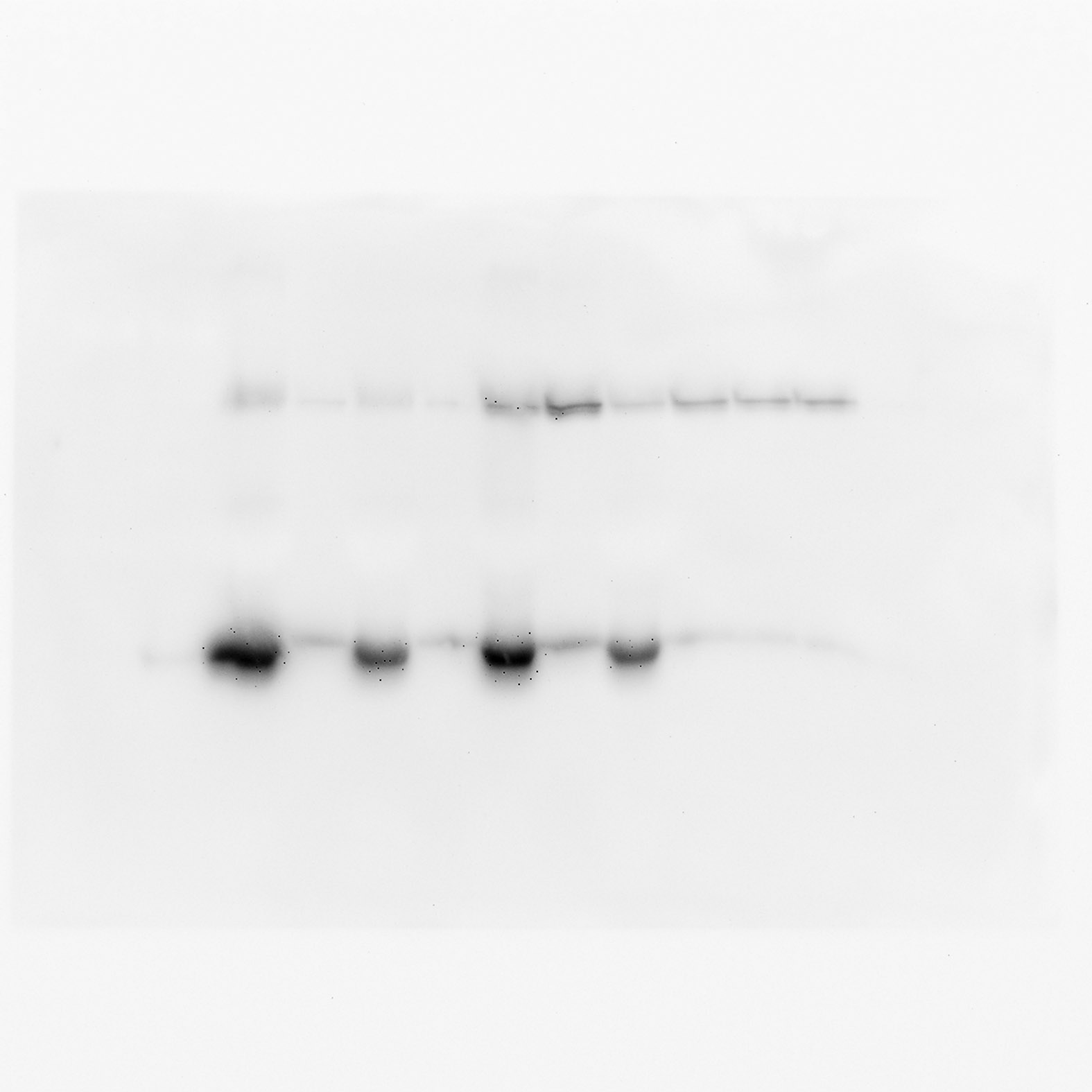

Supplement: Figure 7—source data 1. — A, B contain uncropped western blots shown in Figure 7A and B. The Excel file contains raw data used in Figure 7A’–E. [file elife-86920-fig7-data1.zip › Figure_7_Source_Data_1 /B_V760_2_Shh.jpg]

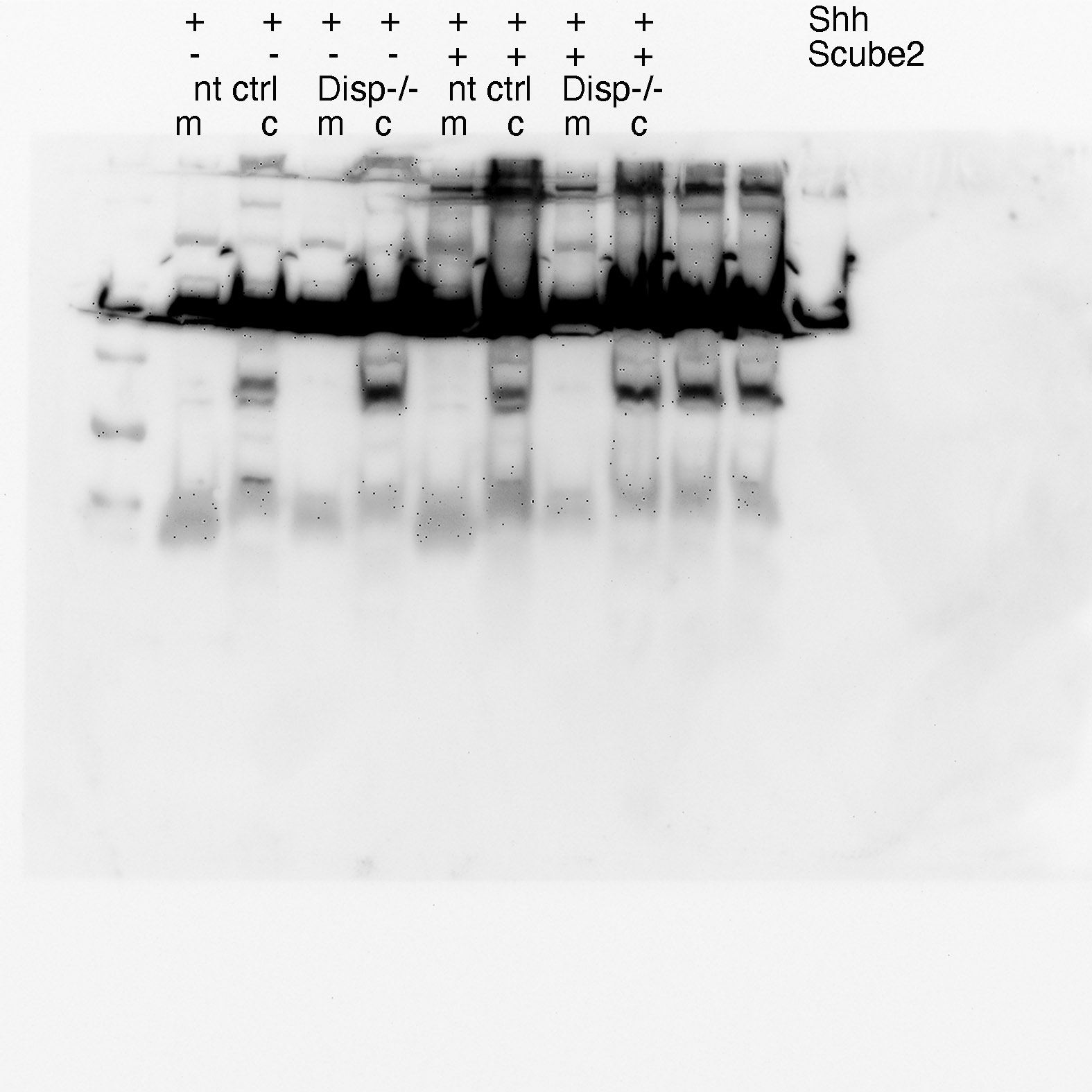

Supplement: Figure 7—figure supplement 1—source data 1. [file elife-86920-fig7-figsupp1-data1.zip › Figure 7-Figure Supplement 1 - Source Data 1/A_V765_1_wash_actin_1min labelled.jpg]

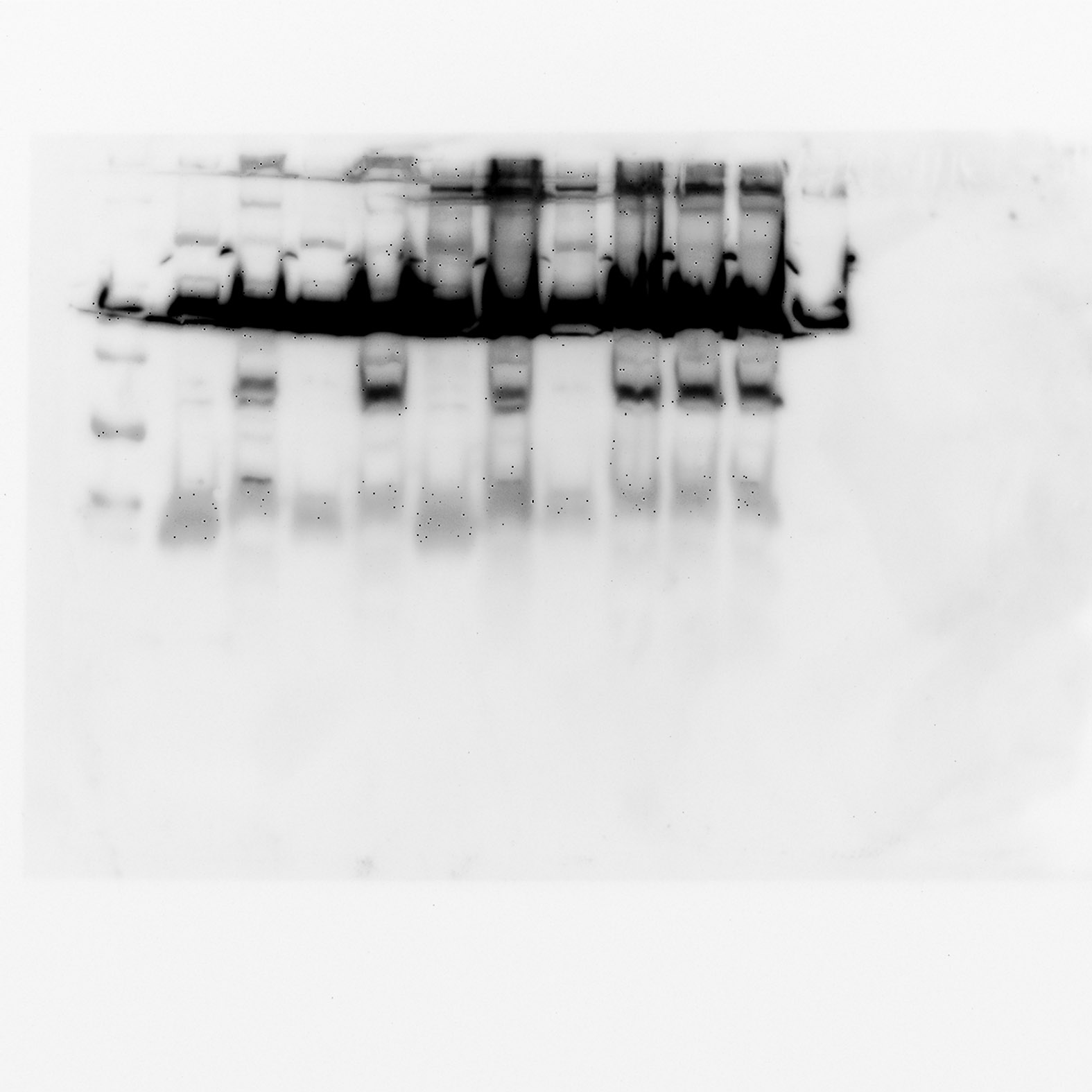

Supplement: Figure 7—figure supplement 1—source data 1. [file elife-86920-fig7-figsupp1-data1.zip › Figure 7-Figure Supplement 1 - Source Data 1/A_V765_1_wash_actin_1min.jpg]

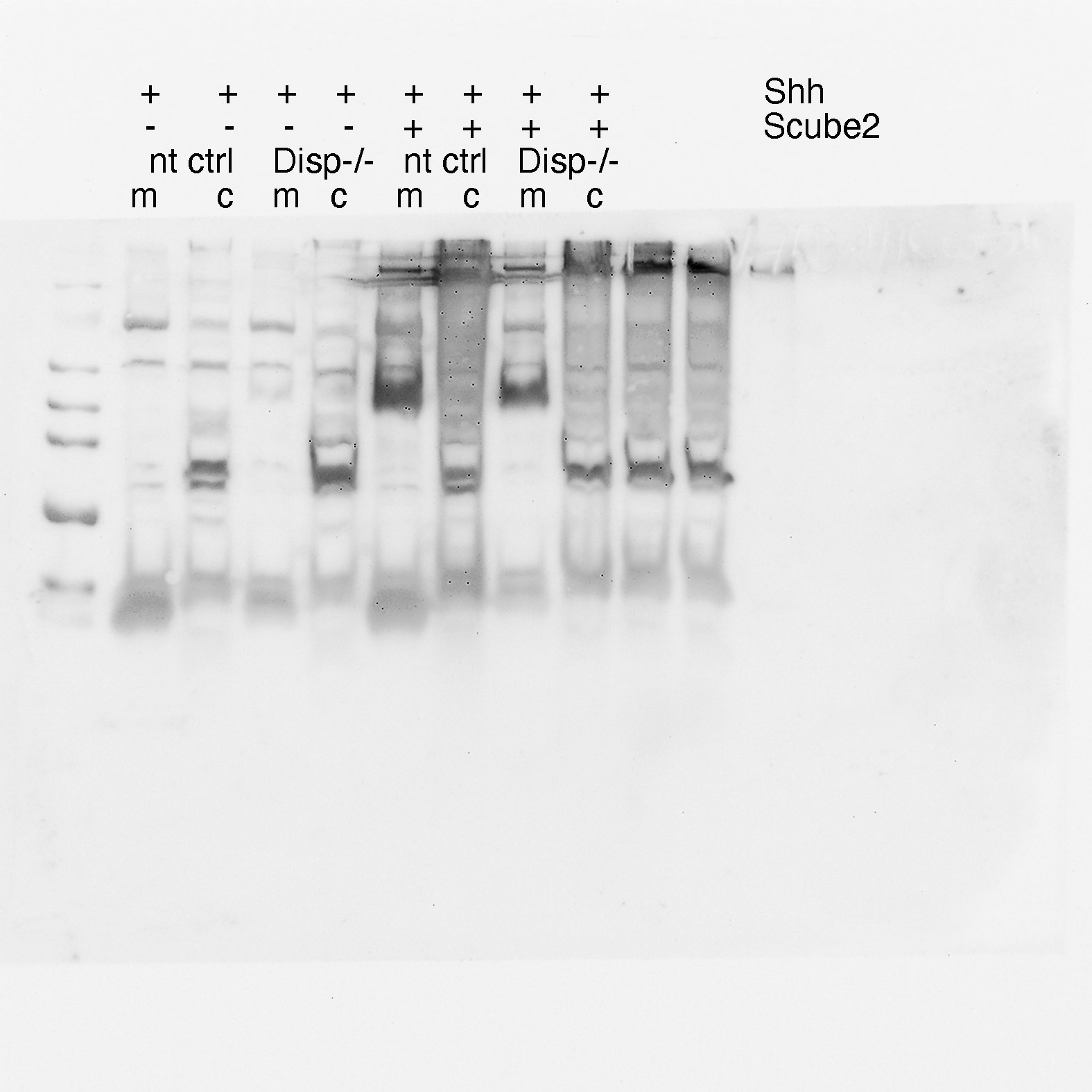

Supplement: Figure 7—figure supplement 1—source data 1. [file elife-86920-fig7-figsupp1-data1.zip › Figure 7-Figure Supplement 1 - Source Data 1/A_V765_1_wash_Flag_1min labelled.jpg]

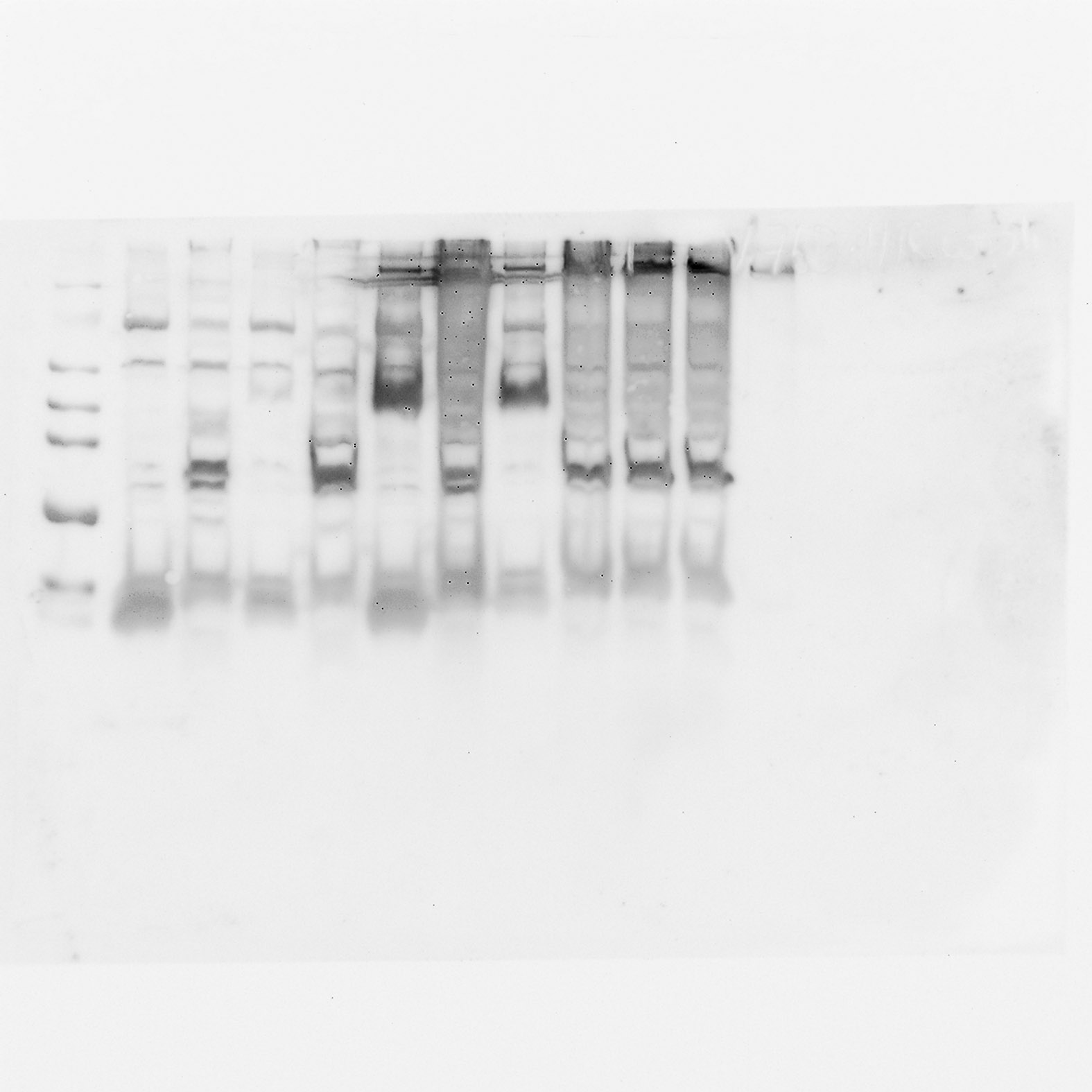

Supplement: Figure 7—figure supplement 1—source data 1. [file elife-86920-fig7-figsupp1-data1.zip › Figure 7-Figure Supplement 1 - Source Data 1/A_V765_1_wash_Flag_1min.jpg]

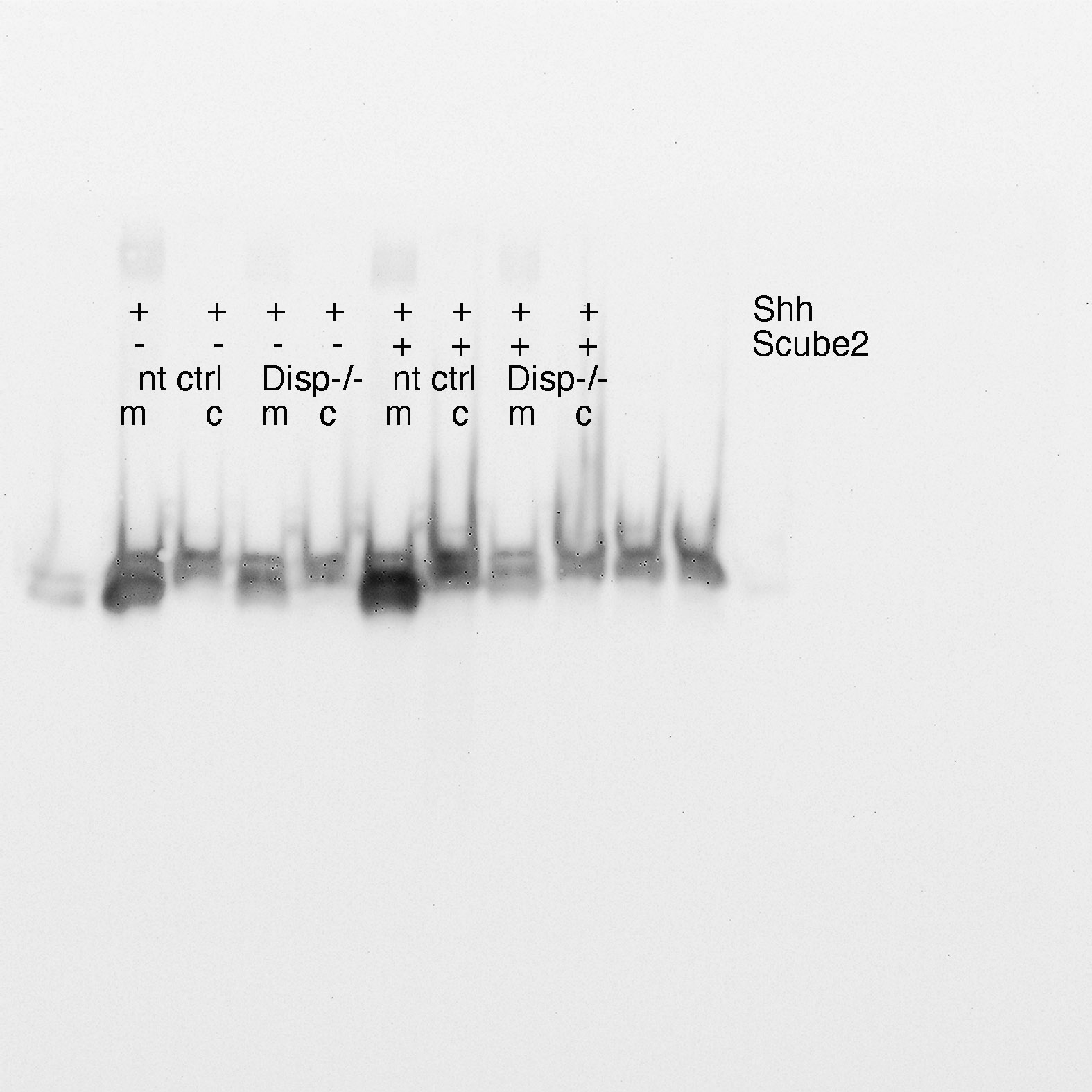

Supplement: Figure 7—figure supplement 1—source data 1. [file elife-86920-fig7-figsupp1-data1.zip › Figure 7-Figure Supplement 1 - Source Data 1/A_V765_H18_wash_30sec labelled.jpg]

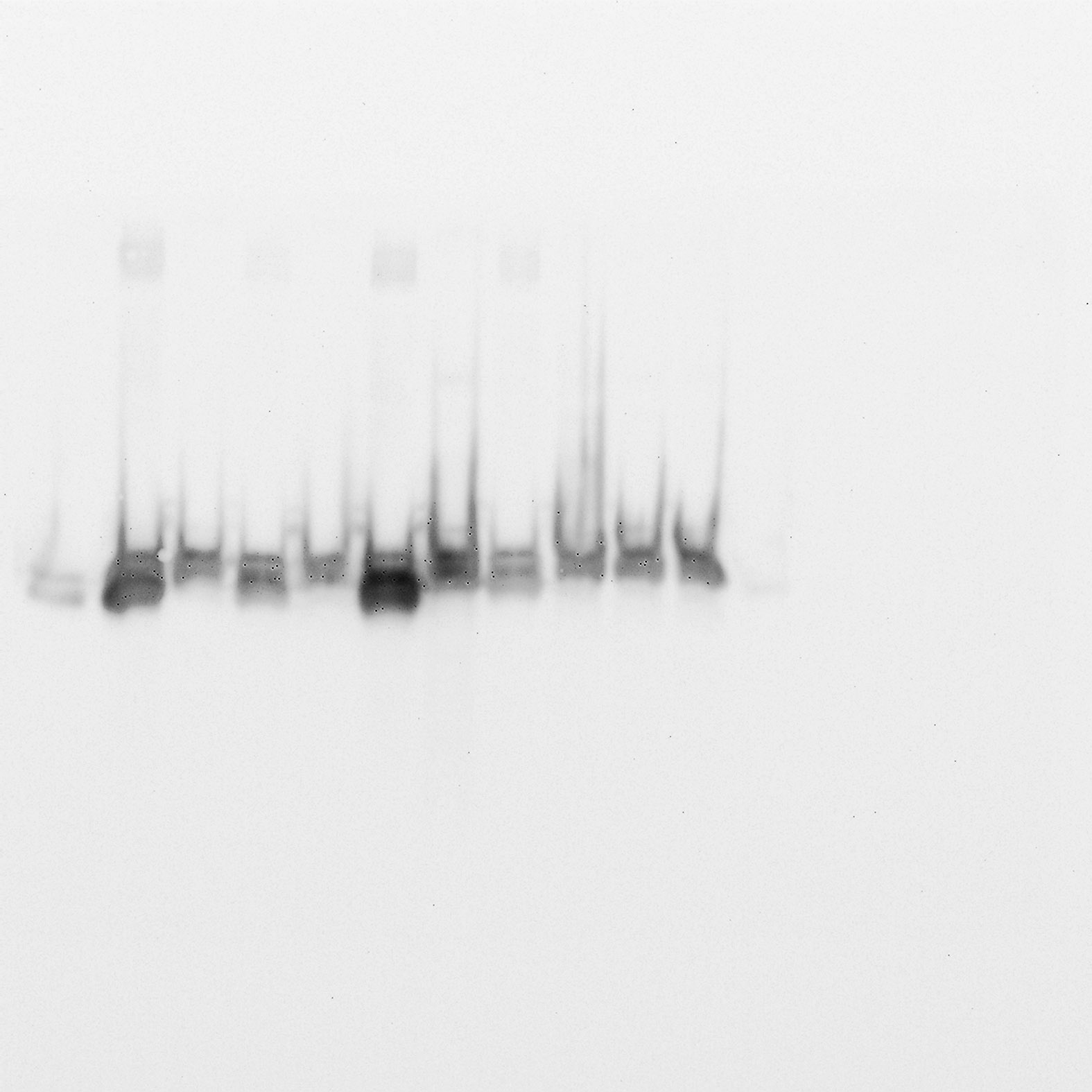

Supplement: Figure 7—figure supplement 1—source data 1. [file elife-86920-fig7-figsupp1-data1.zip › Figure 7-Figure Supplement 1 - Source Data 1/A_V765_H18_wash_30sec.jpg]

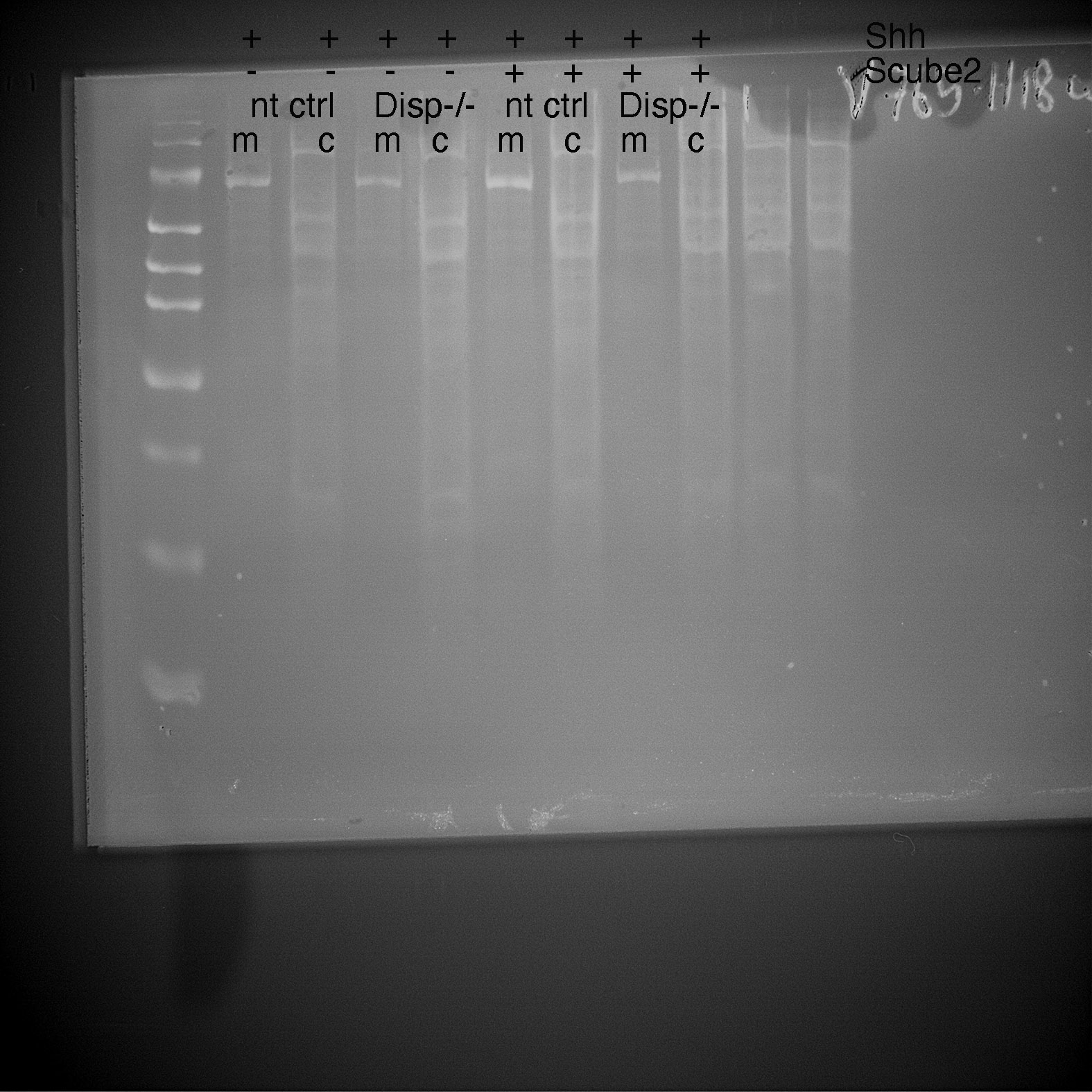

Supplement: Figure 7—figure supplement 1—source data 1. [file elife-86920-fig7-figsupp1-data1.zip › Figure 7-Figure Supplement 1 - Source Data 1/A_V765_H18w_ponS labelled.jpg]

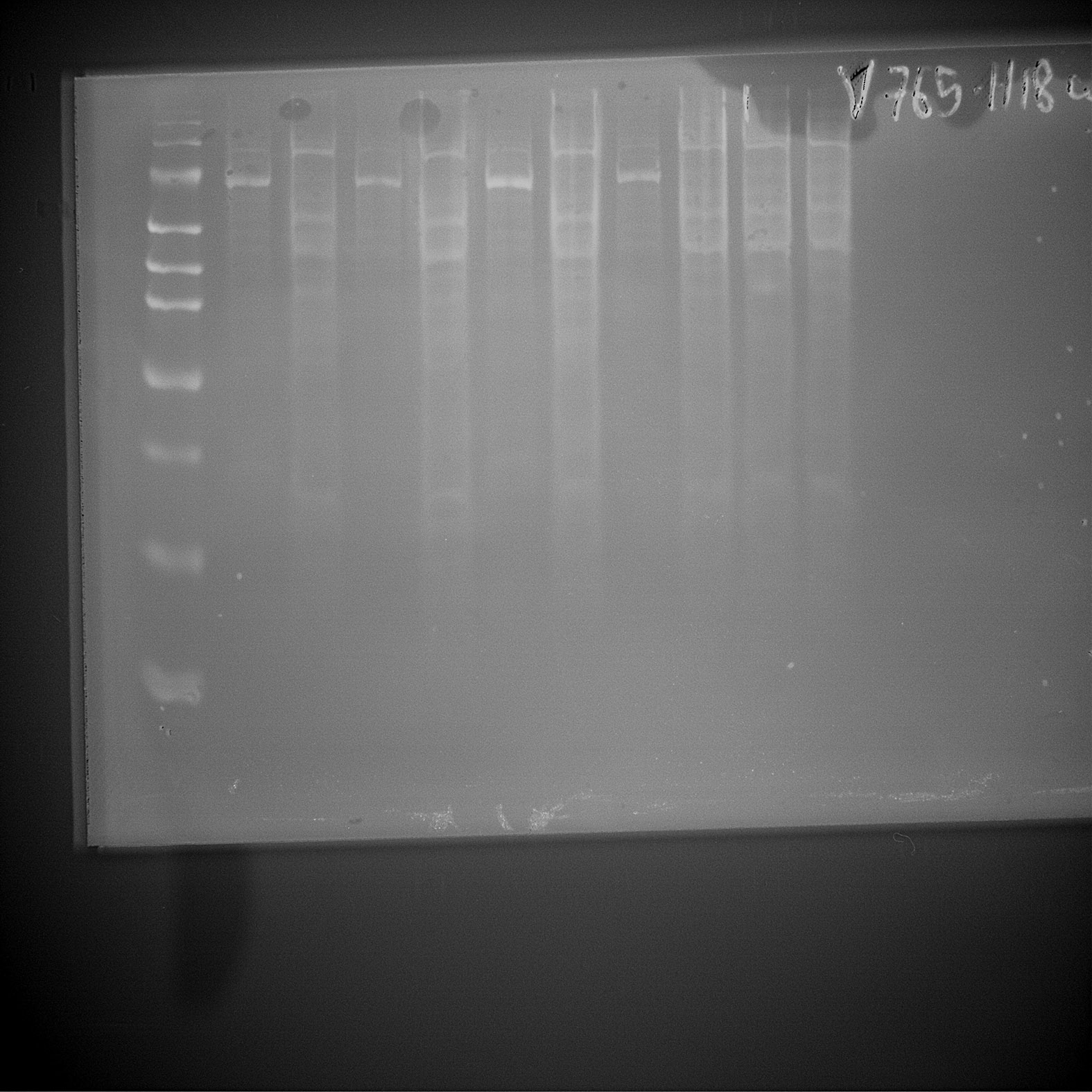

Supplement: Figure 7—figure supplement 1—source data 1. [file elife-86920-fig7-figsupp1-data1.zip › Figure 7-Figure Supplement 1 - Source Data 1/A_V765_H18w_ponS.jpg]

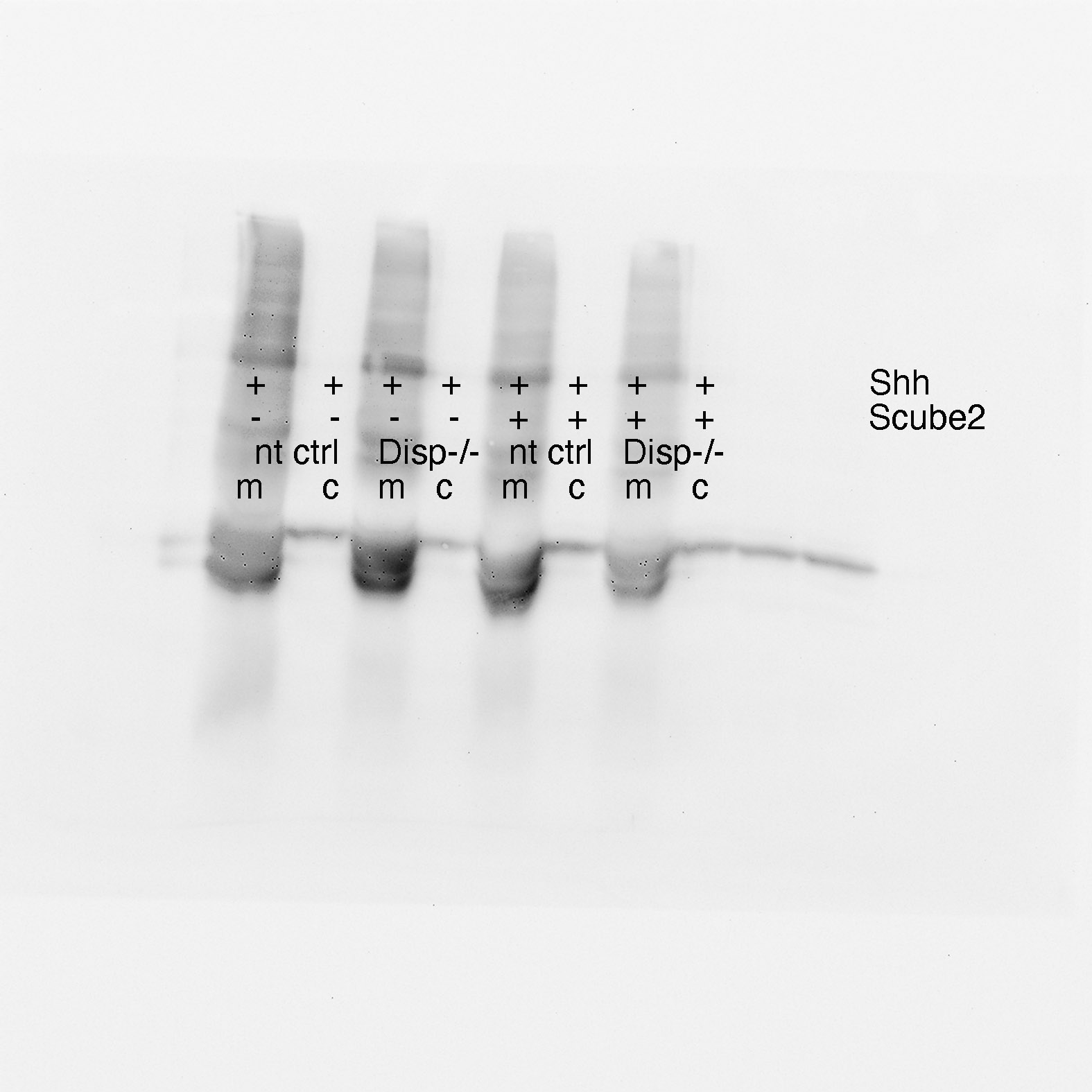

Supplement: Figure 7—figure supplement 1—source data 1. [file elife-86920-fig7-figsupp1-data1.zip › Figure 7-Figure Supplement 1 - Source Data 1/B_V757_9_0.6min_aShh labelled.jpg]

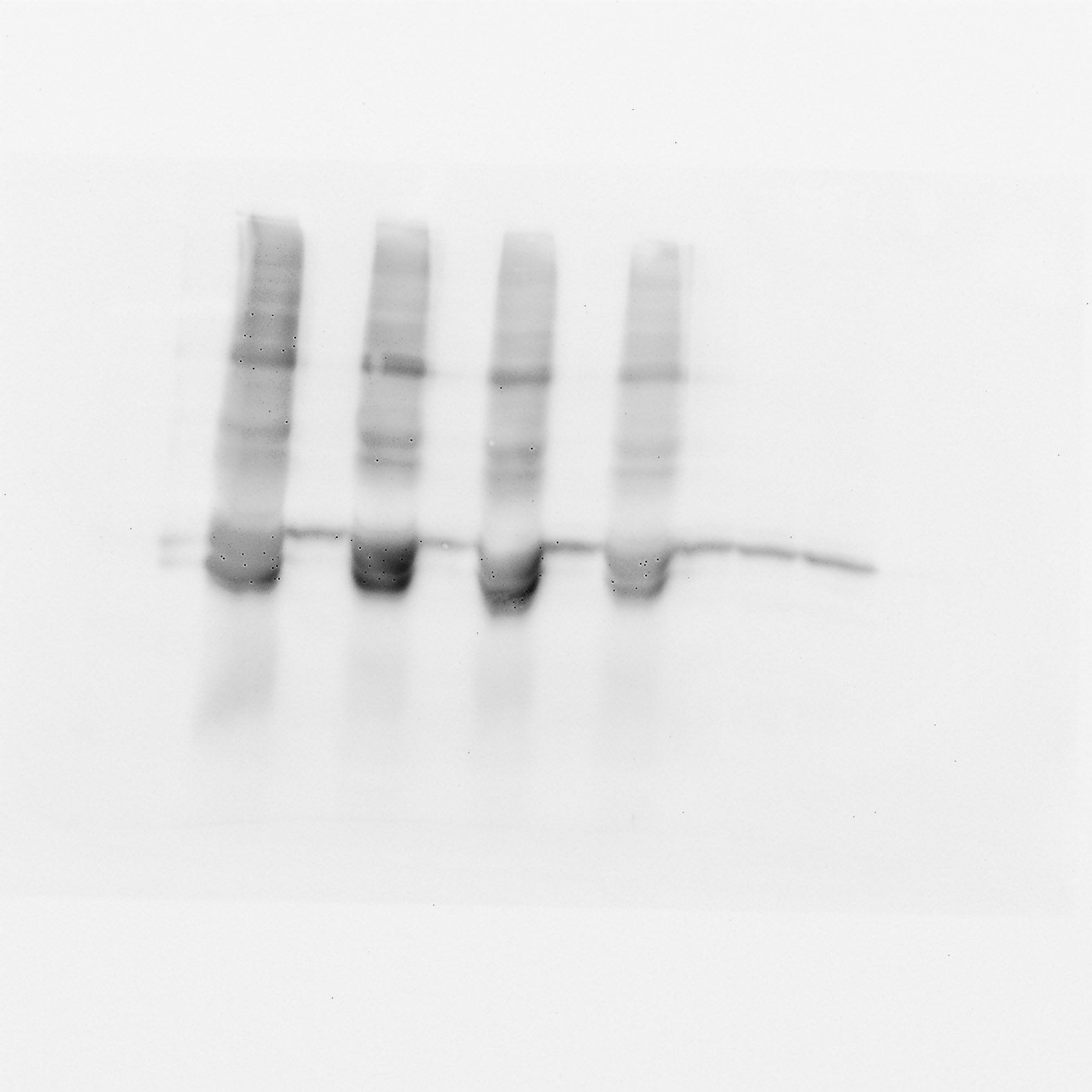

Supplement: Figure 7—figure supplement 1—source data 1. [file elife-86920-fig7-figsupp1-data1.zip › Figure 7-Figure Supplement 1 - Source Data 1/B_V757_9_0.6min_aShh.jpg]

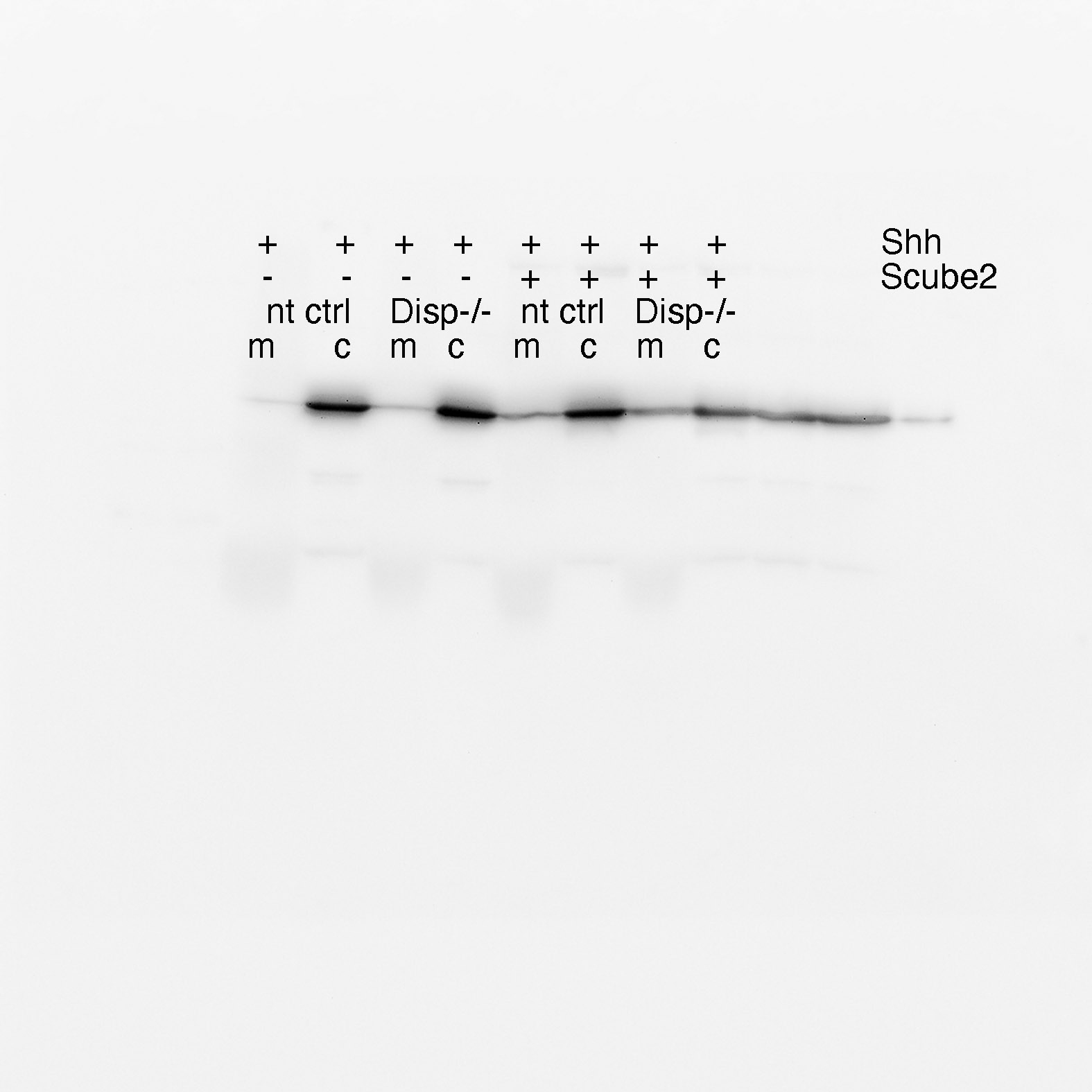

Supplement: Figure 7—figure supplement 1—source data 1. [file elife-86920-fig7-figsupp1-data1.zip › Figure 7-Figure Supplement 1 - Source Data 1/B_V757_9_actin_top7sec labelled.jpg]

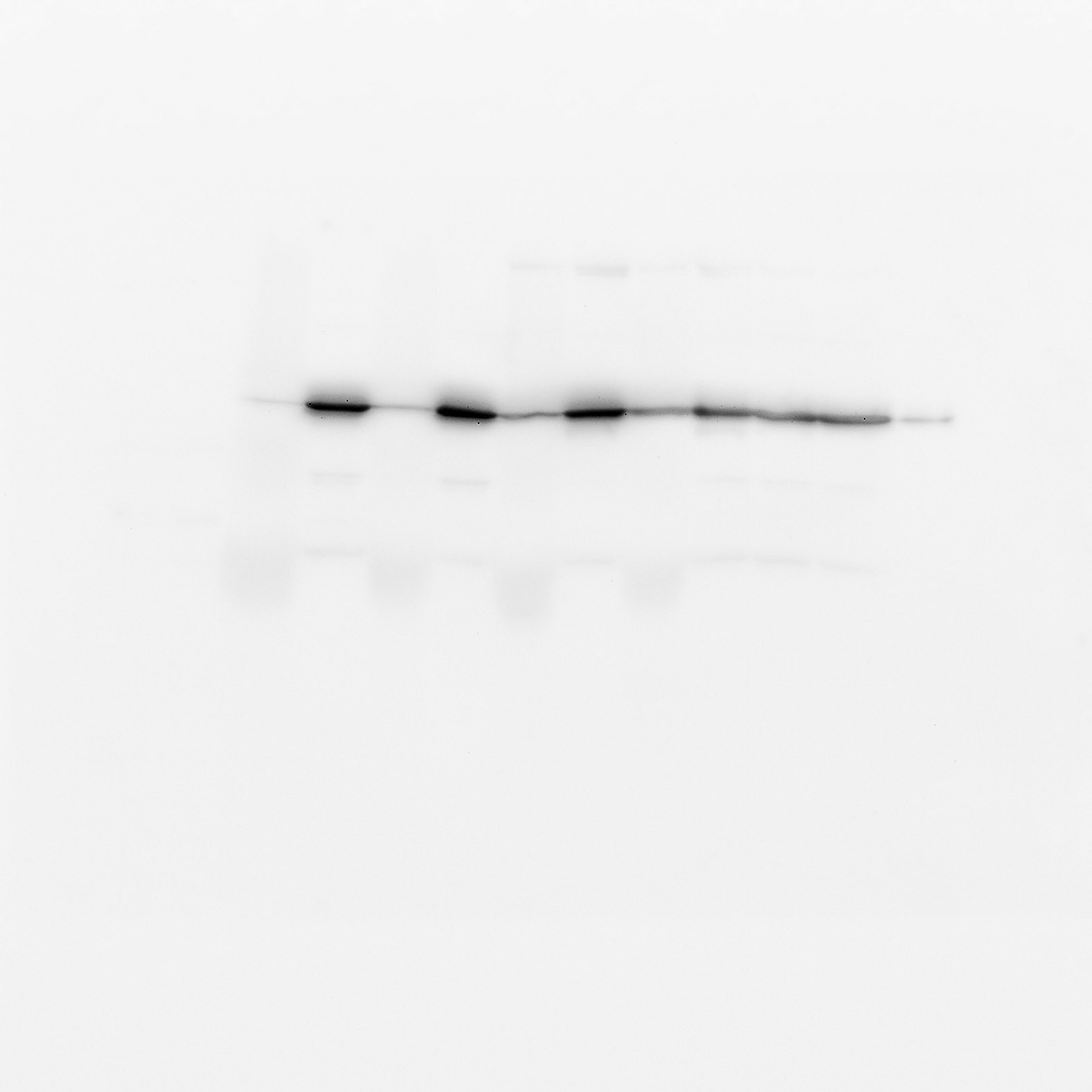

Supplement: Figure 7—figure supplement 1—source data 1. [file elife-86920-fig7-figsupp1-data1.zip › Figure 7-Figure Supplement 1 - Source Data 1/B_V757_9_actin_top7sec.jpg]

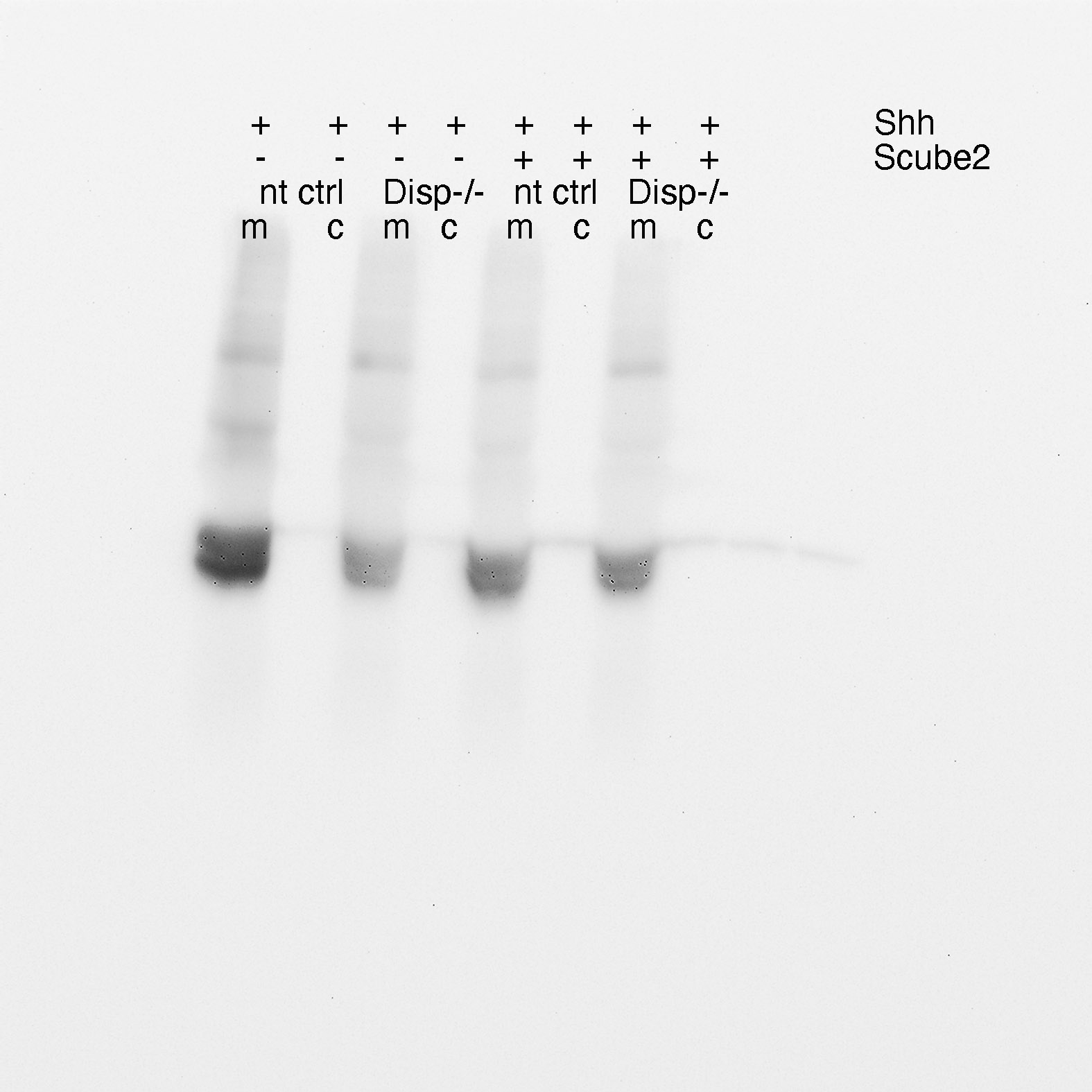

Supplement: Figure 7—figure supplement 1—source data 1. [file elife-86920-fig7-figsupp1-data1.zip › Figure 7-Figure Supplement 1 - Source Data 1/B_V757_9_ApoA1_1min labelled.jpg]

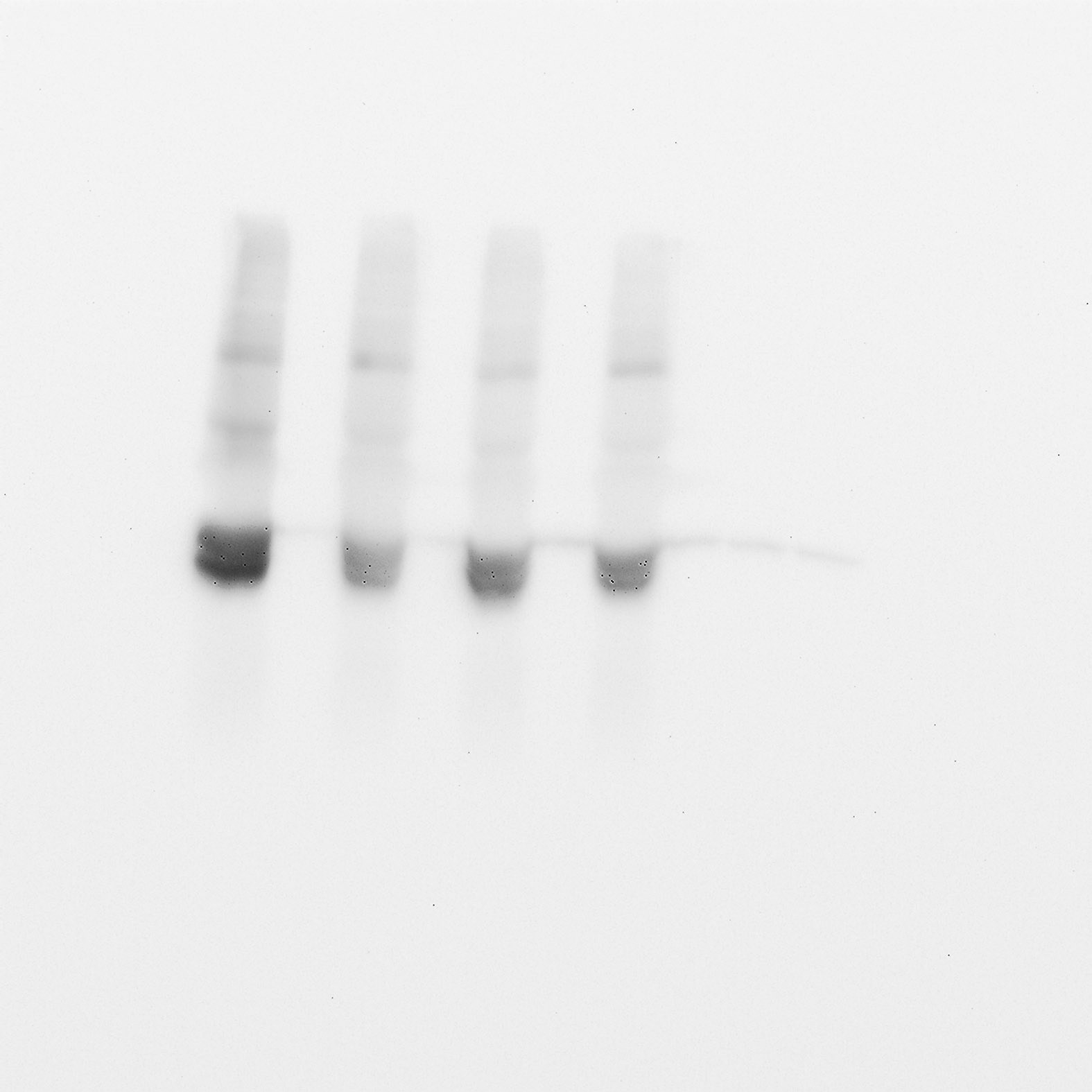

Supplement: Figure 7—figure supplement 1—source data 1. [file elife-86920-fig7-figsupp1-data1.zip › Figure 7-Figure Supplement 1 - Source Data 1/B_V757_9_ApoA1_1min.jpg]

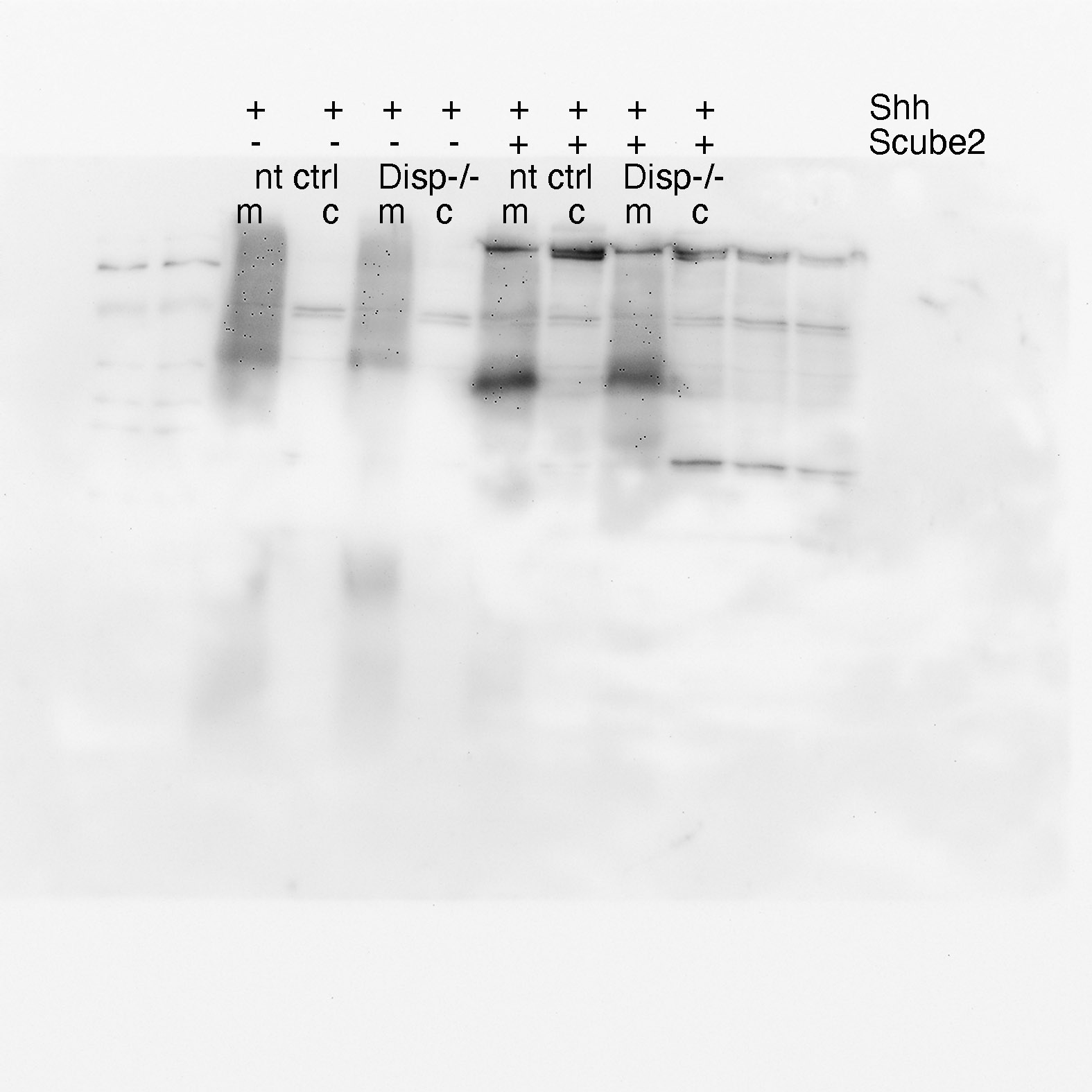

Supplement: Figure 7—figure supplement 1—source data 1. [file elife-86920-fig7-figsupp1-data1.zip › Figure 7-Figure Supplement 1 - Source Data 1/B_V757_Flag_9-1min labelled.jpg]

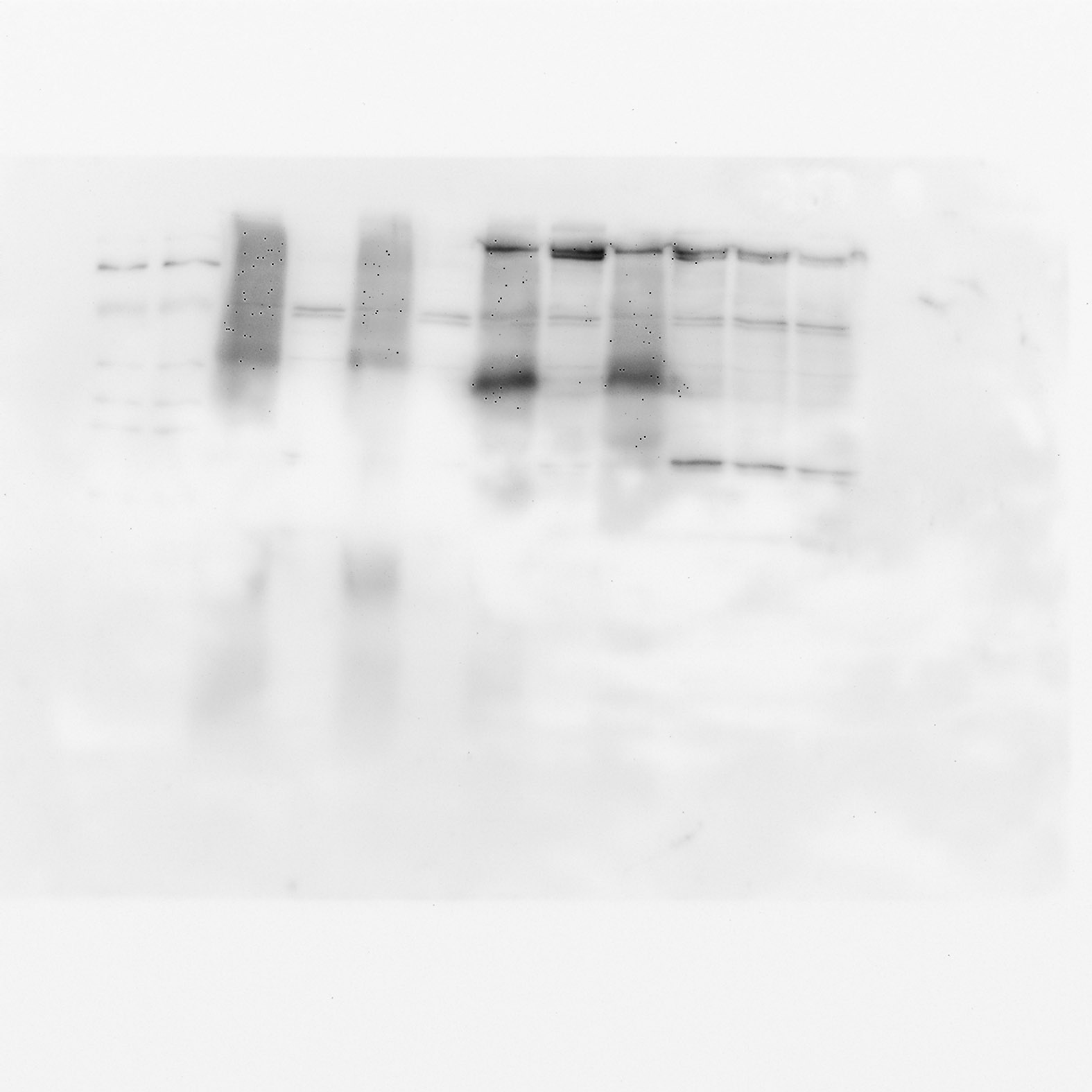

Supplement: Figure 7—figure supplement 1—source data 1. [file elife-86920-fig7-figsupp1-data1.zip › Figure 7-Figure Supplement 1 - Source Data 1/B_V757_Flag_9-1min.jpg]

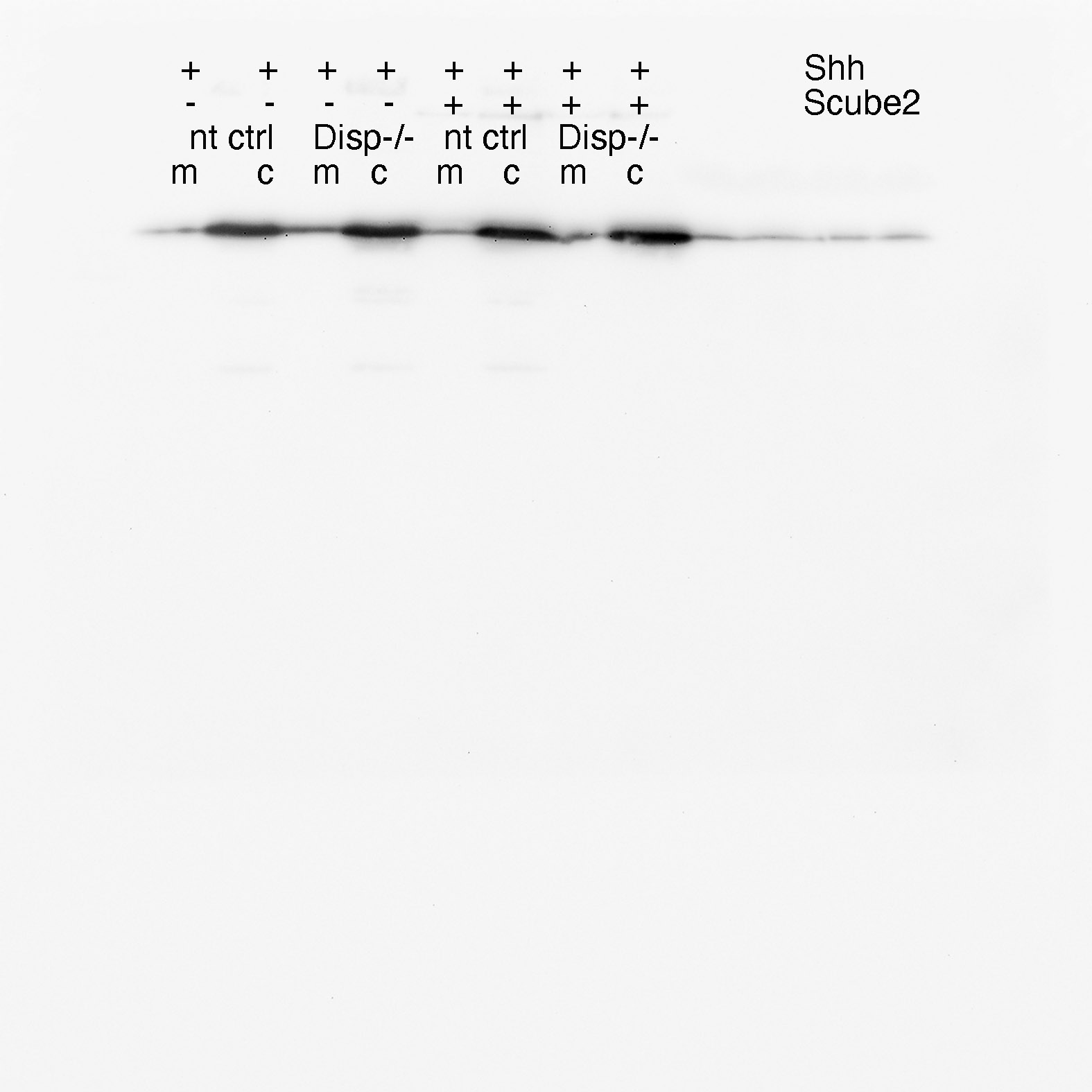

Supplement: Figure 7—figure supplement 1—source data 1. [file elife-86920-fig7-figsupp1-data1.zip › Figure 7-Figure Supplement 1 - Source Data 1/C_21-02-2022_16Bit_V753-4_anti Actin_41sec labelled.jpg]

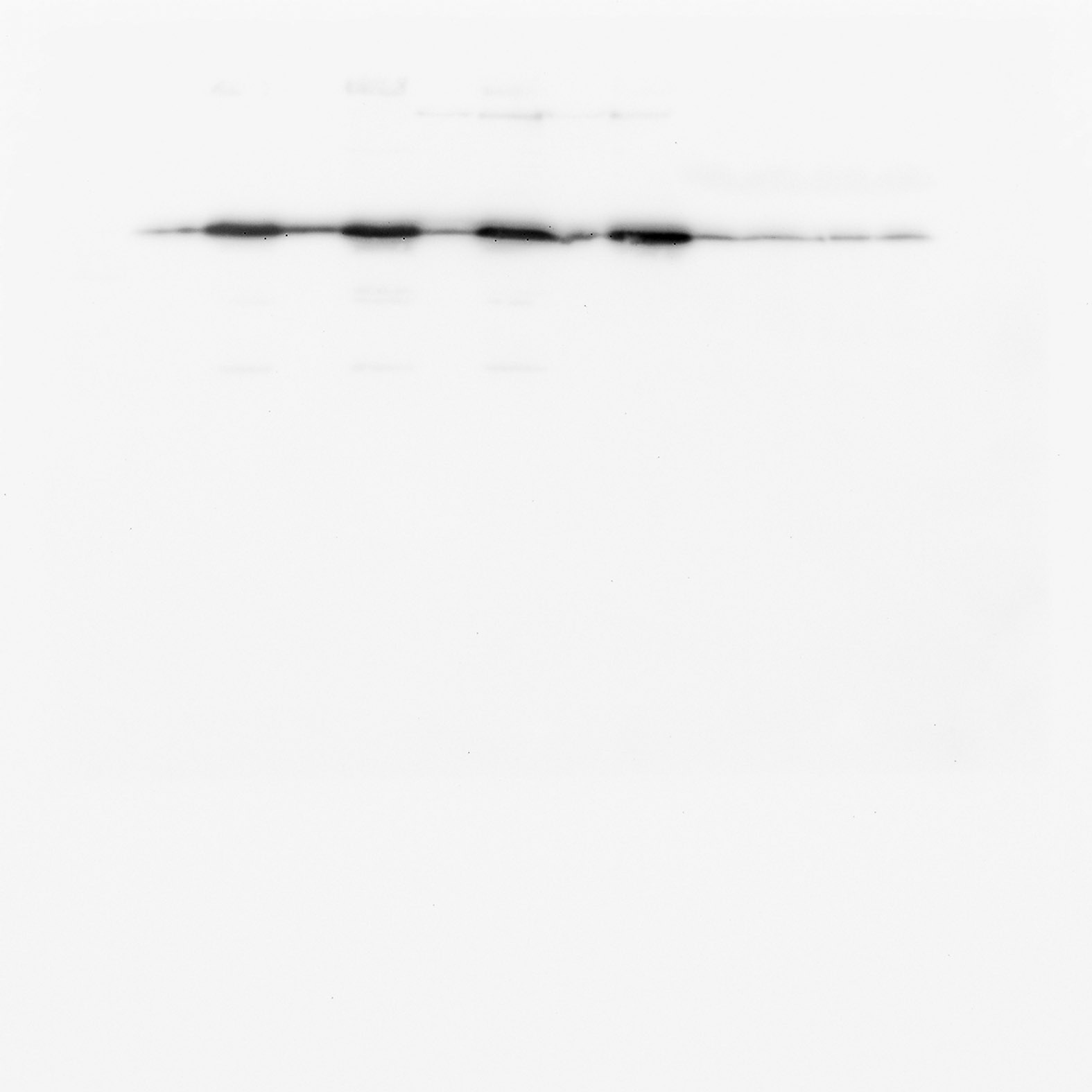

Supplement: Figure 7—figure supplement 1—source data 1. [file elife-86920-fig7-figsupp1-data1.zip › Figure 7-Figure Supplement 1 - Source Data 1/C_21-02-2022_16Bit_V753-4_anti Actin_41sec.jpg]

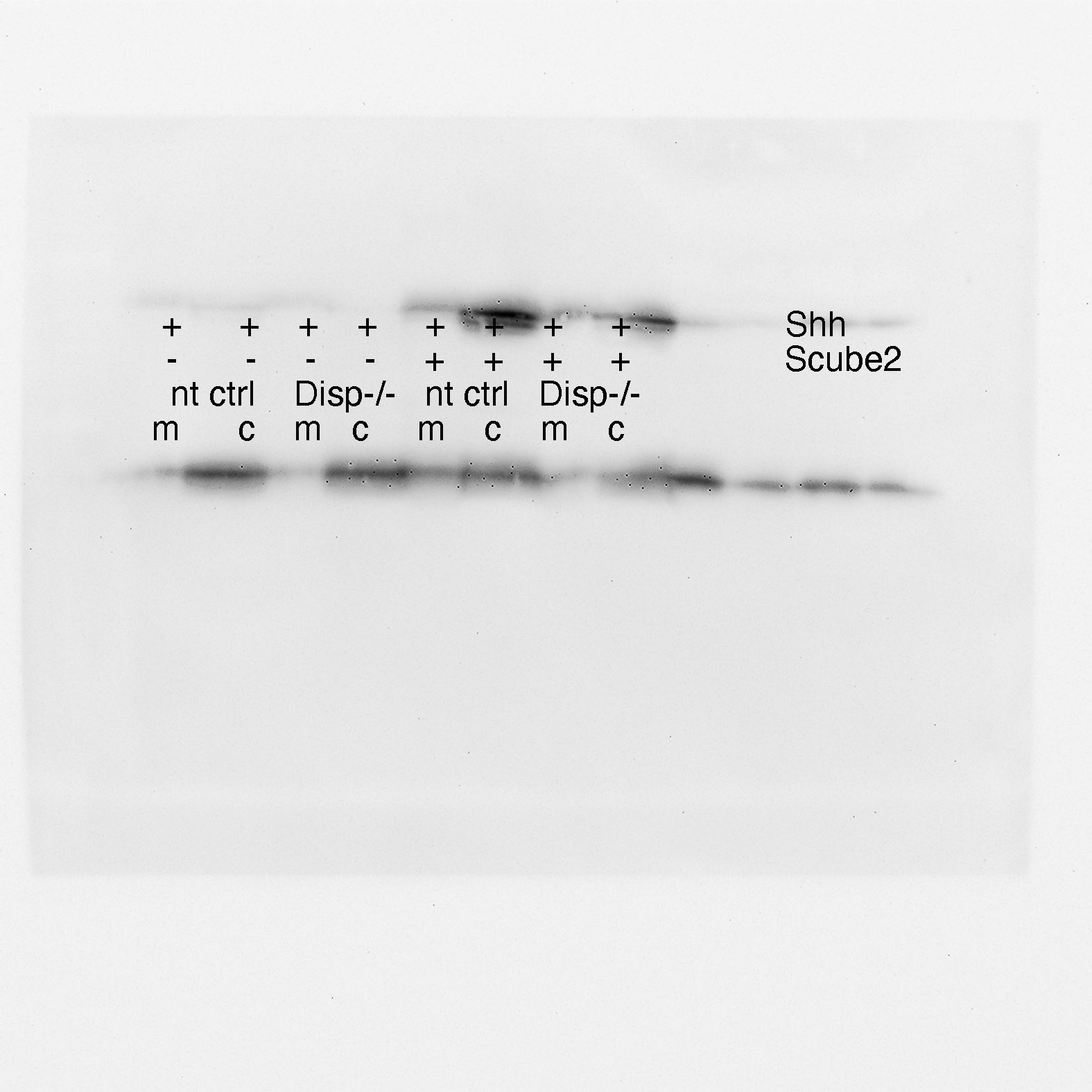

Supplement: Figure 7—figure supplement 1—source data 1. [file elife-86920-fig7-figsupp1-data1.zip › Figure 7-Figure Supplement 1 - Source Data 1/C_V752_4_Shh_2min labelled.jpg]

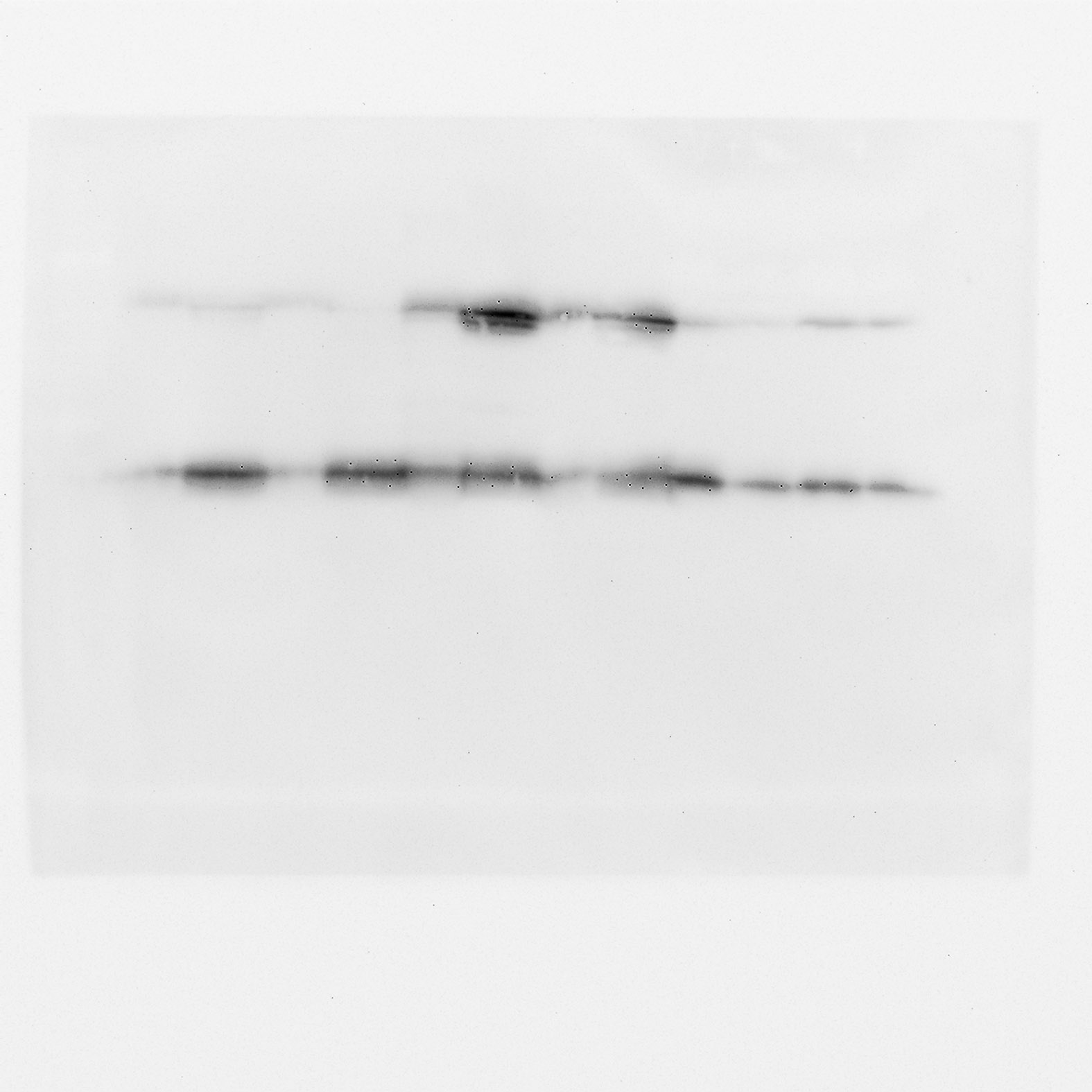

Supplement: Figure 7—figure supplement 1—source data 1. [file elife-86920-fig7-figsupp1-data1.zip › Figure 7-Figure Supplement 1 - Source Data 1/C_V752_4_Shh_2min.jpg]

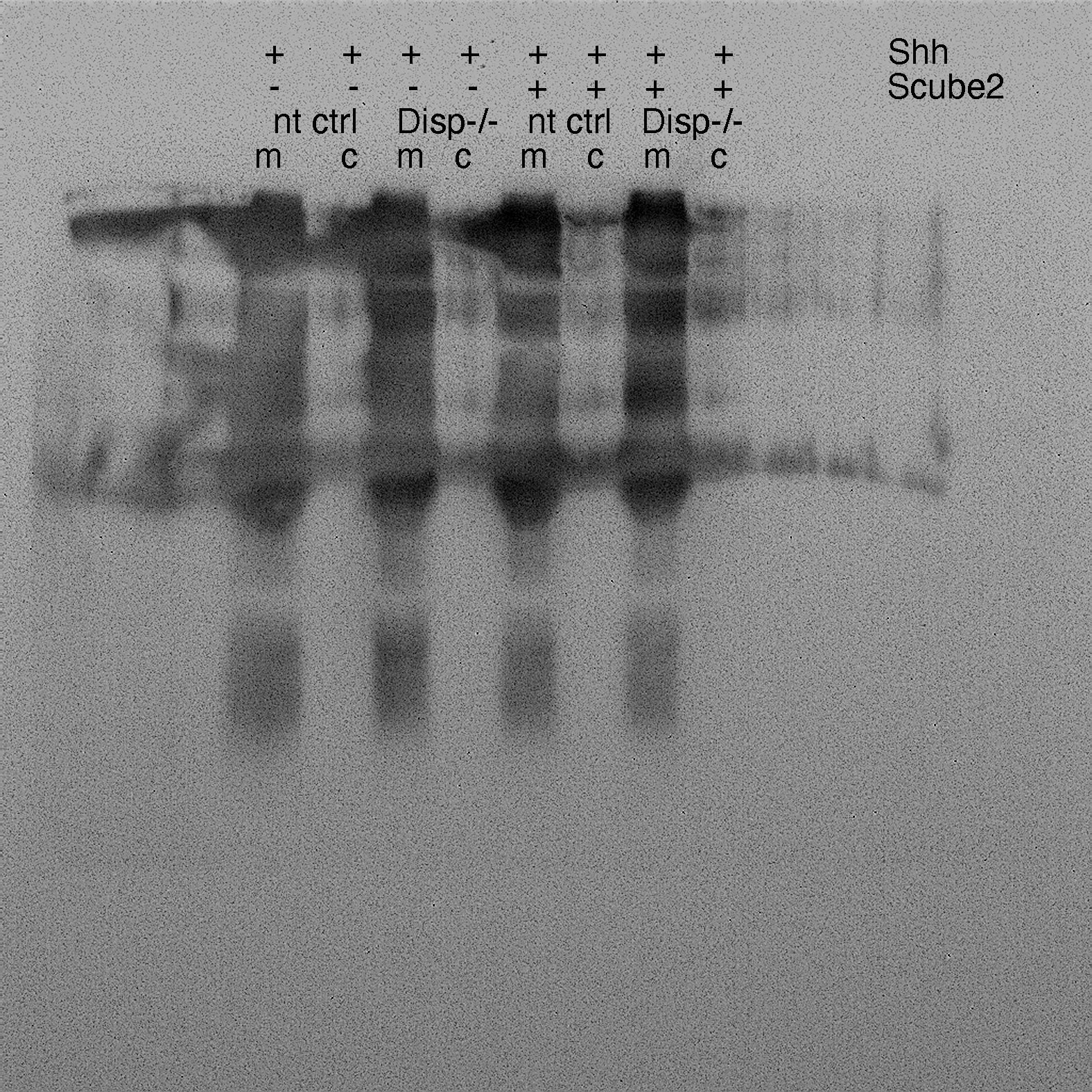

Supplement: Figure 7—figure supplement 1—source data 1. [file elife-86920-fig7-figsupp1-data1.zip › Figure 7-Figure Supplement 1 - Source Data 1/D_V760_2_1min_ApoA1 labelled.jpg]

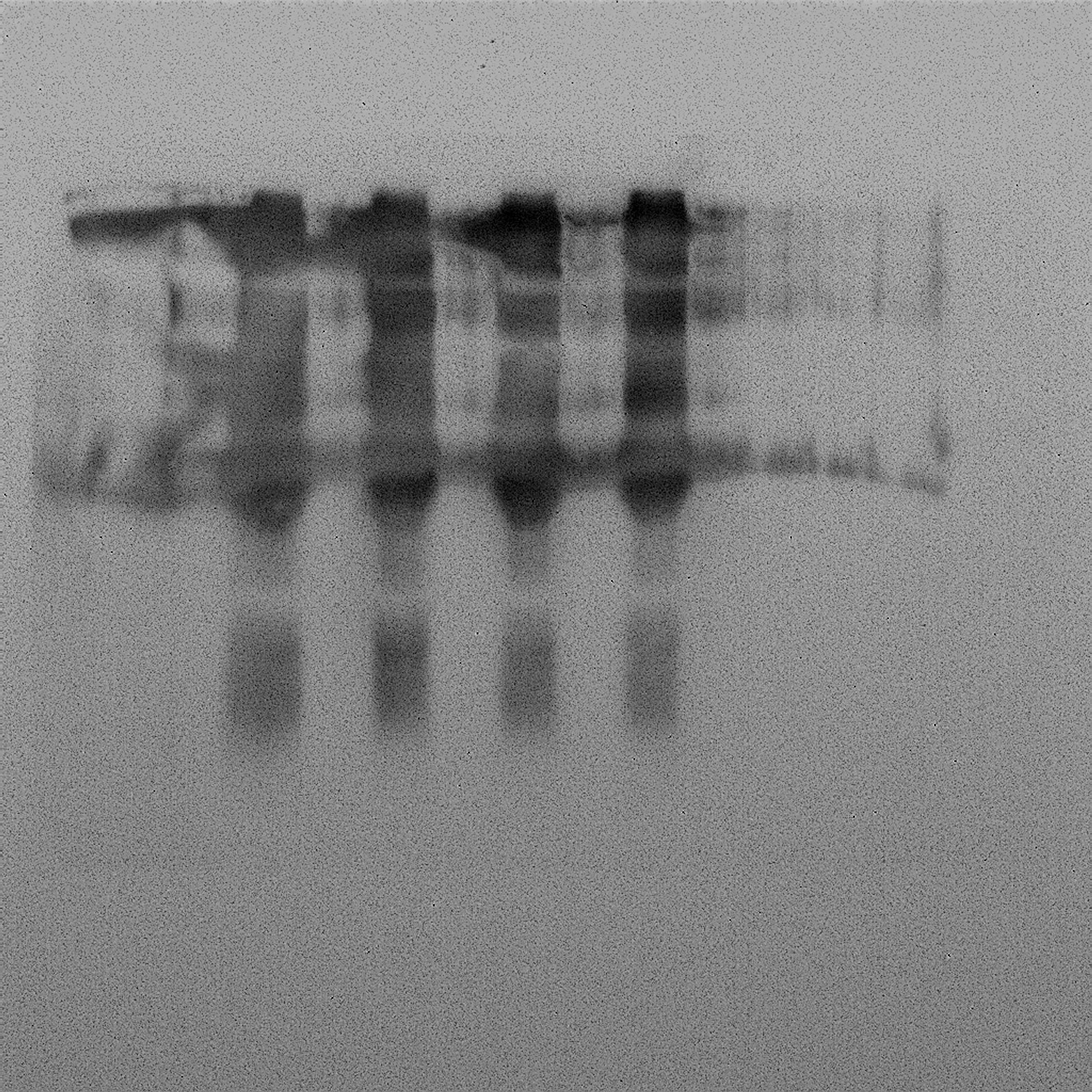

Supplement: Figure 7—figure supplement 1—source data 1. [file elife-86920-fig7-figsupp1-data1.zip › Figure 7-Figure Supplement 1 - Source Data 1/D_V760_2_1min_ApoA1.jpg]

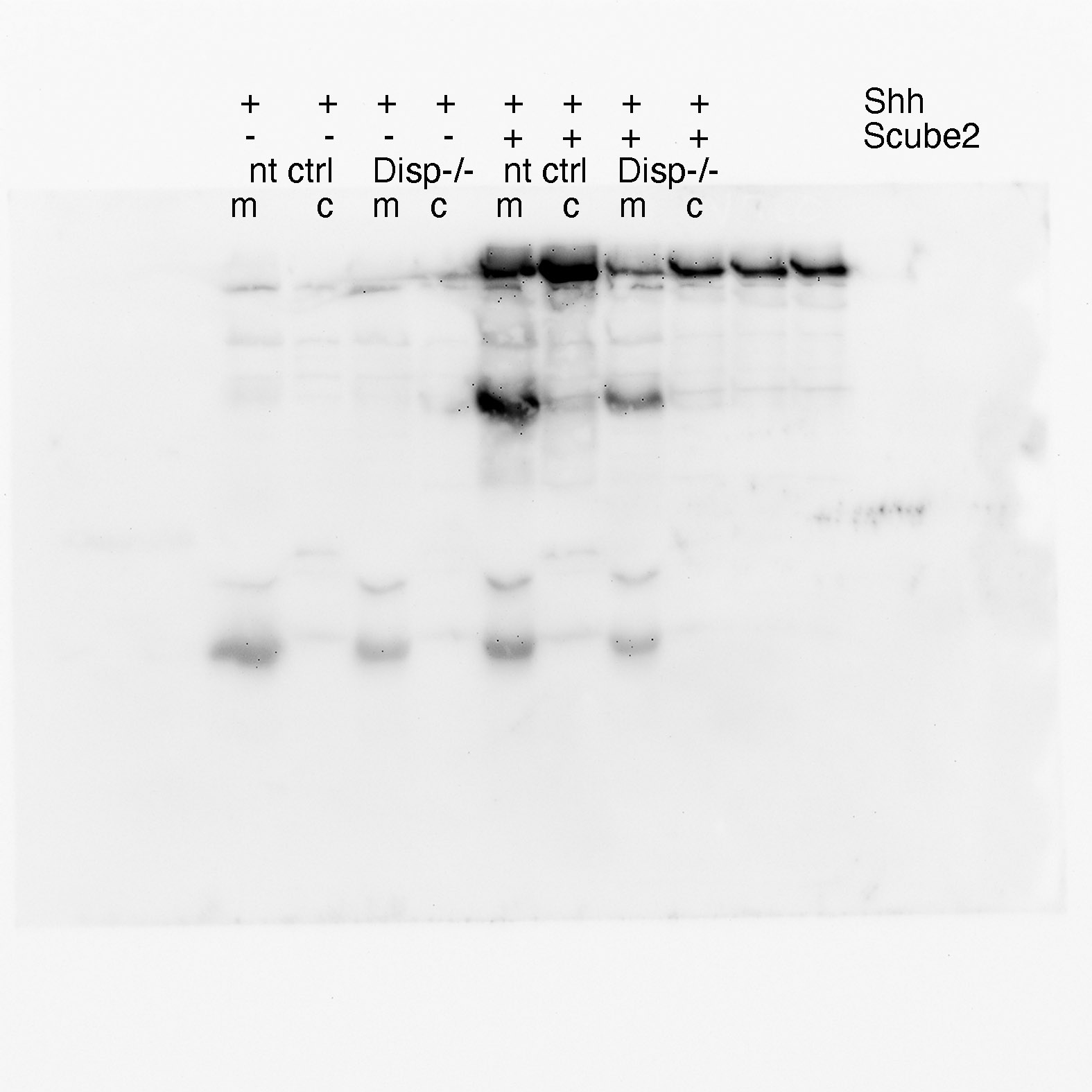

Supplement: Figure 7—figure supplement 1—source data 1. [file elife-86920-fig7-figsupp1-data1.zip › Figure 7-Figure Supplement 1 - Source Data 1/D_V760_2_1min_Scube labelled.jpg]

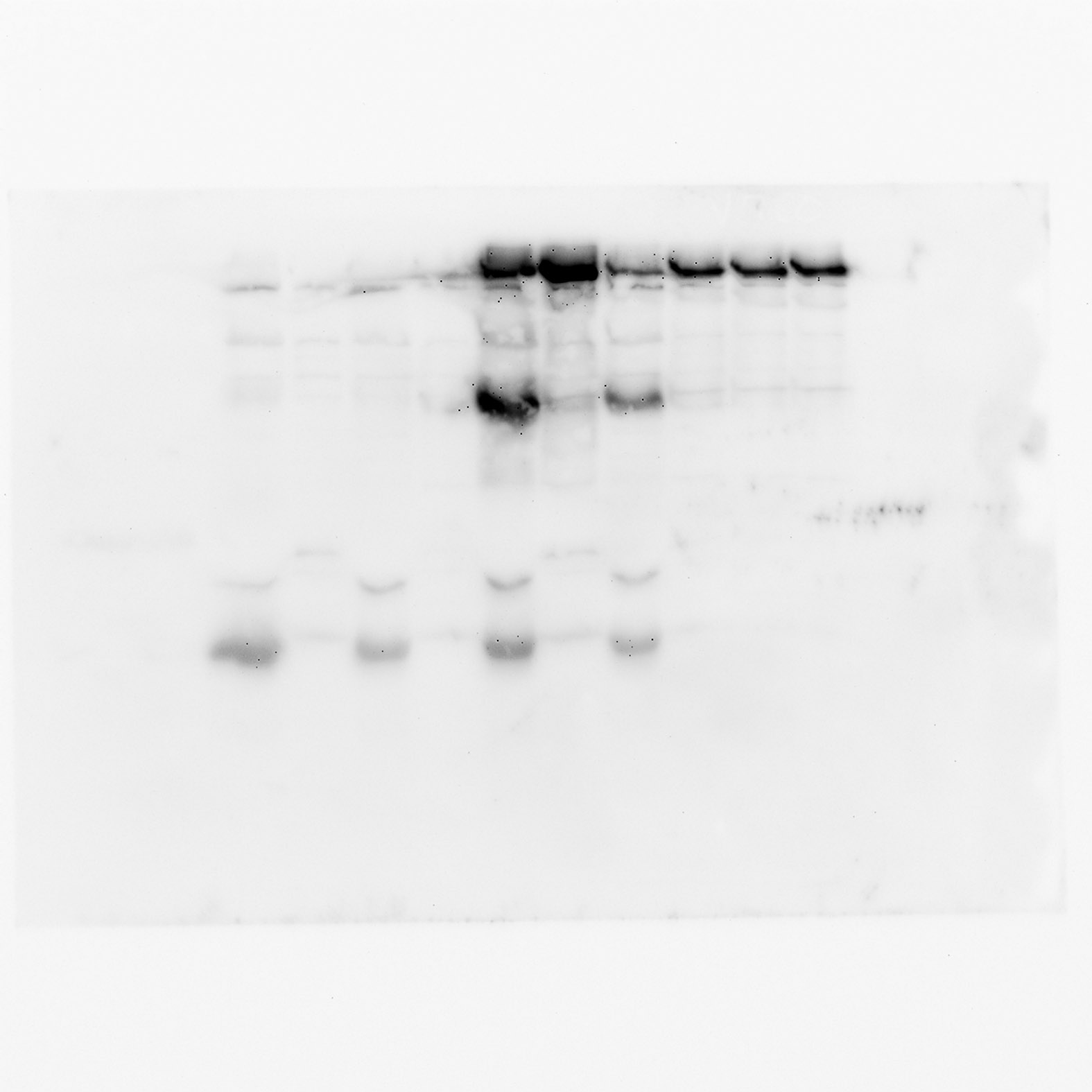

Supplement: Figure 7—figure supplement 1—source data 1. [file elife-86920-fig7-figsupp1-data1.zip › Figure 7-Figure Supplement 1 - Source Data 1/D_V760_2_1min_Scube.jpg]

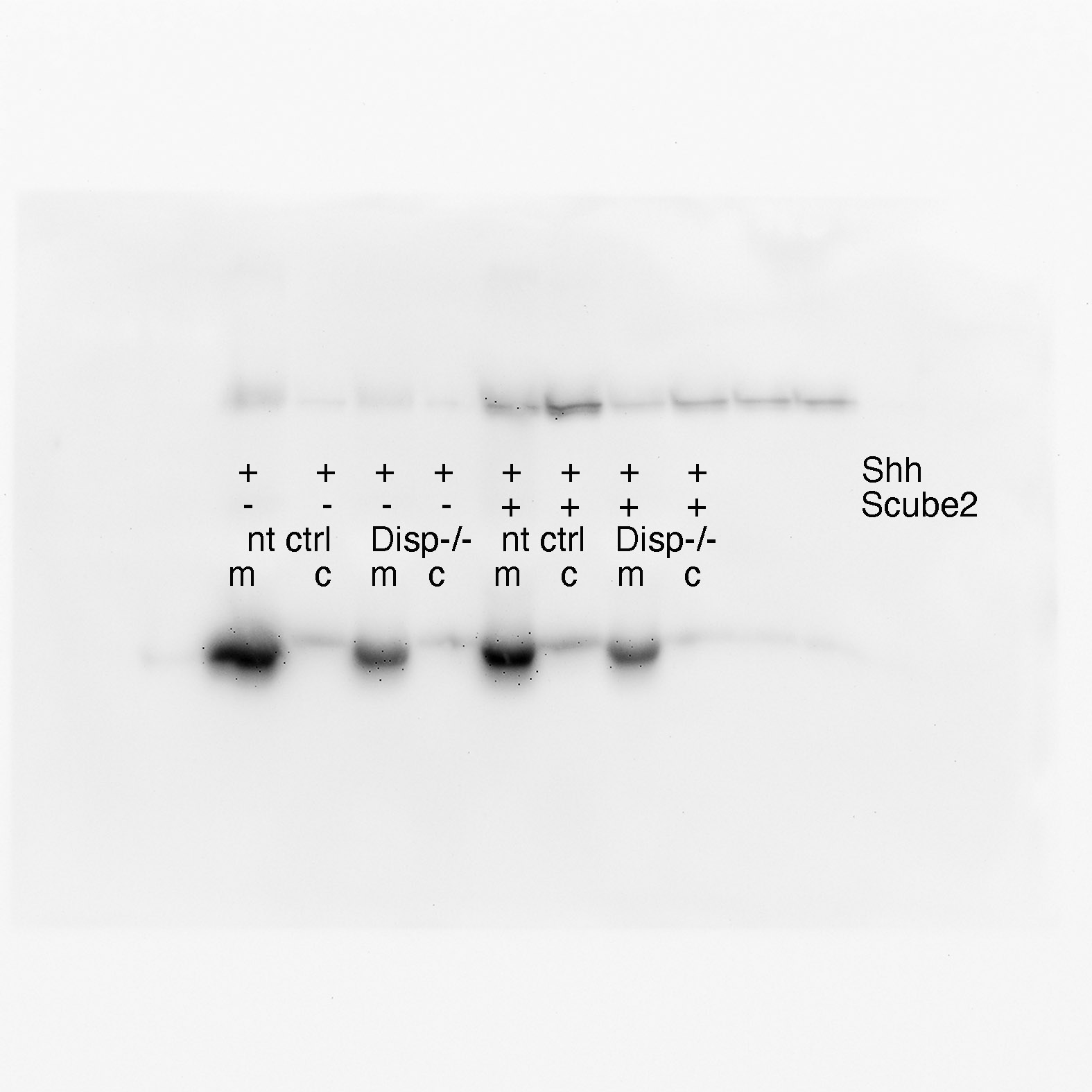

Supplement: Figure 7—figure supplement 1—source data 1. [file elife-86920-fig7-figsupp1-data1.zip › Figure 7-Figure Supplement 1 - Source Data 1/D_V760_2_1min_Shh labelled.jpg]

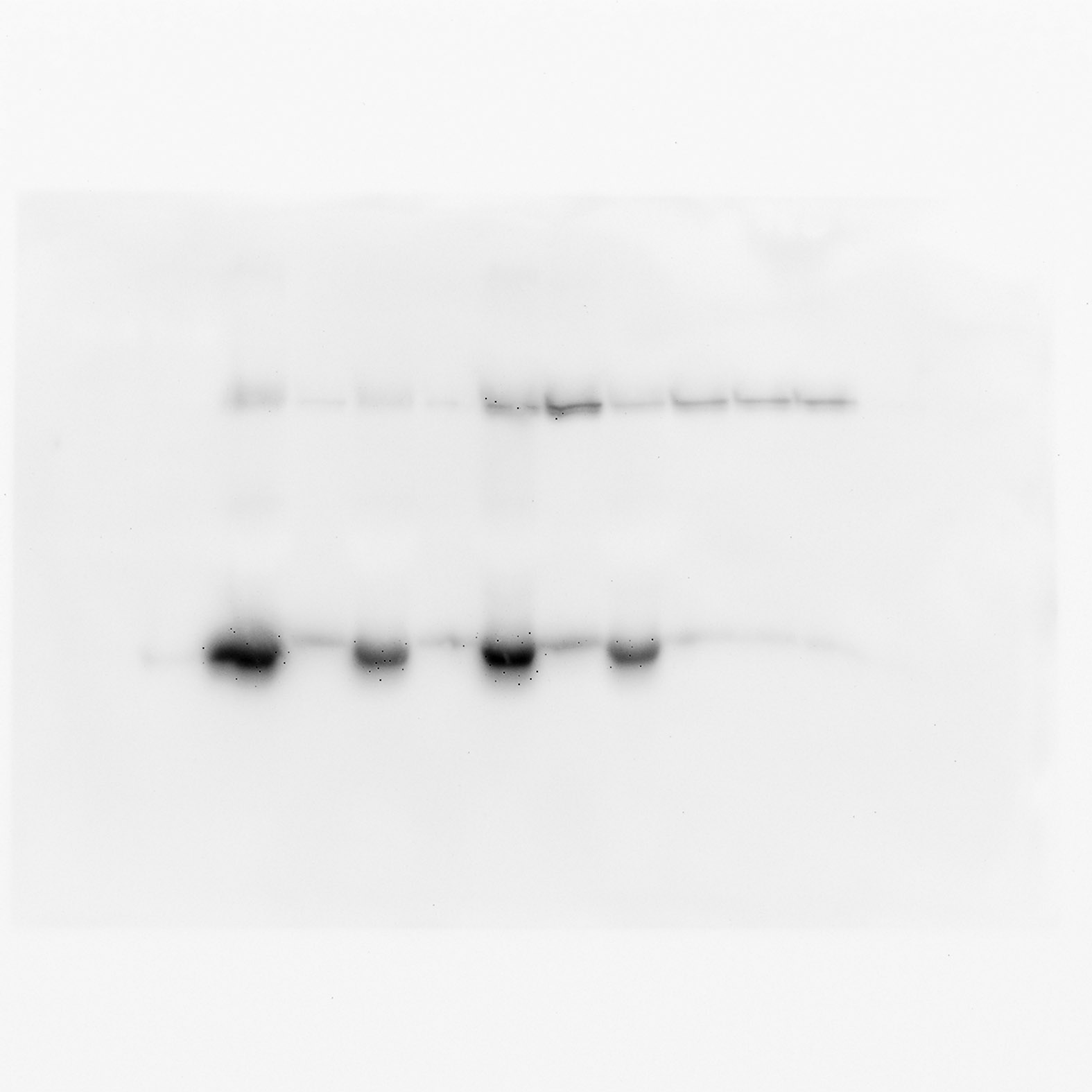

Supplement: Figure 7—figure supplement 1—source data 1. [file elife-86920-fig7-figsupp1-data1.zip › Figure 7-Figure Supplement 1 - Source Data 1/D_V760_2_1min_Shh.jpg]
