# Supplementary material for: Two-way Dispatched function in Sonic hedgehog shedding and transfer to high-density lipoproteins
Source: eLife. 2024 Sep 19;12:RP86920. doi: 10.7554/eLife.86920 (PMC11412720; doi:10.7554/eLife.86920)
Supplement: Supplementary file 1. [file elife-86920-supp1.docx]

**Supplementary File 1. Raw data used for the figures in this study.**

| Figure | Cell type | Constructs used/treatment | Values (Mean)±SD, n | P-values |
| --- | --- | --- | --- | --- |

| 1A` | nt | Shh±Scube2 | 48.7±11% / 325±46%, n=7 | <0.0001 |
| --- | --- | --- | --- | --- |
| 1A` | Disp^-/-^ / nt | Shh±Scube2 | 31.9±17% / 325±46%, n=7 | <0.0001 |
| 1A` | Disp^-/-^ / nt | Shh+Scube2 | 74.9±26% / 325±46%, n=7 | <0.0001 |
| 1B` | nt | ^C25S^Shh±Scube2 | 207±311% / 314±248%, n=3 | 0.8503 |
| 1B` | Disp^-/-^ / nt | ^C25S^Shh±Scube2 | 39.7±28% / 314±248%, n=3 | 0.2863 |
| 1B` | Disp^-/-^ / nt | ^C25S^Shh+Scube2 | 31.7±29% / 314±248%, n=3 | 0.2679 |
| 1C` | nt | ShhN±Scube2 | 810±131% / 607±310%, n=4 | 0.5065 |
| 1C` | Disp^-/-^ / nt | ShhN±Scube2 | 960±300% / 607±310%, n=4 | 0.1366 |
| 1C` | Disp^-/-^ / nt | ShhN+Scube2 | 194±148% / 607±310%, n=4 | 0.0739 |
| 1D` | nt | ^C25S^ShhN±Scube2 | 1048±21% / 1003±411%, n=3 | 0.9913 |
| 1D` | Disp^-/-^ / nt | ^C25S^ShhN±Scube2 | 894±193% / 1003±411%, n=3 | 0.9033 |
| 1D` | Disp^-/-^ / nt | ^C25S^ShhN+Scube2 | 801±177% / 1003±411%, n=3 | 0.6345 |

| 2C | Bosc23 | CMK/DMSO | 77%±38% / 501%±249%, n=6 | 0.0021 |
| --- | --- | --- | --- | --- |
| 2E | nt | Shh±Scube2 | 33.3±18% / 62±21%, n=6 | 0.0216 |
| 2E | Disp^-/-^ / nt | Shh±Scube2 | 27.7±14.5% / 62±21%, n=6 | 0.0059 |
| 2E | Disp^-/-^ / nt | Shh+Scube2 | 51±13% / 62±21%, n=6 | 0.5483 |

| 3B` | nt | Shh±Scube2 .05% FCS | 87.3±64% / 377±91%, n=3 | 0.0013 |
| --- | --- | --- | --- | --- |
| 3B` | Disp^-/-^ / nt | Shh±Scube2 .05% FCS | 62±15% / 377±91%, n=3 | 0.0008 |
| 3B` | Disp^-/-^ / nt | Shh+Scube2 .05% FCS | 92±59% / 377±91%, n=3 | 0.0015 |
| 3C` | nt | Shh±Scube2 5% FCS | 92±50% / 299±112%, n=3 | 0.0158 |
| 3C` | Disp^-/-^ / nt | Shh±Scube2 5% FCS | 78±45% / 299±112%, n=3 | 0.0112 |
| 3C` | Disp^-/-^ / nt | Shh+Scube2 5% FCS | 67±44% / 299±112%, n=3 | 0.0087 |
| 3D` | nt | Shh±Scube2 10% FCS | 52±45% / 338±77%, n=3 | 0.0001 |
| 3D` | Disp^-/-^ / nt | Shh±Scube2 10% FCS | 30±12% / 338±77%, n=3 | <0.0001 |
| 3D` | Disp^-/-^ / nt | Shh+Scube2 10% FCS | 55±12% / 338±77%, n=3 | 0.0002 |
| 3E | nt | Shh±Scube2 10% FCS | 152±12% / 158±59%, n=3 | 0.9916 |
| 3E | Disp^-/-^ / nt | Shh±Scube2 10% FCS | 112±3% / 158±59%, n=3 | 0.2659 |
| 3E | Disp^-/-^ / nt | Shh+Scube2 10% FCS | 105±26% / 158±59%, n=3 | 0.1841 |

| 4A | C3H10T1/2 | mock/Shh (125µl) | 0.13±0.02au / 0.877±0.14 au, n=6 | <0.0001 |
| --- | --- | --- | --- | --- |
| 4A | C3H10T1/2 | Shh/ ^C25S^Shh (125µl) | 0.877±0.14 au / 0.872±0.09au, n=6 | >0.9999 |
| 4A | C3H10T1/2 | Shh/^C25S^Shh (250µl) | 2.16±0.1au / 2.69±0.28au, n=6 | <0.0001 |
| 4B | C3H10T1/2 | Shh/ ^C25S^Shh (1x) Ptch1 | 2.66±0.15 / 2.64±0.08, n=3 | >0.9999 |
| 4B | C3H10T1/2 | Shh/ ^C25S^Shh (2x) Ptch1 | 3.11±0.49 / 3.31±1, n=3 | 0.9938 |
| 4B | C3H10T1/2 | Shh/ ^C25S^Shh (1x) Gli1 | 5.7±0.6 / 5.33±0.26, n=3 | 0.9446 |
| 4B | C3H10T1/2 | Shh/ ^C25S^Shh (2x) Gli1 | 5.5±0.79 / 5.5±1.14, n=3 | >0.9999 |
| 4B | C3H10T1/2 | Shh (1x) / ^C25S^ShhN (2x) Ptch1 | 2.66±0.15 / 0.78±0.48, n=3 | 0.0083 |
| 4B | C3H10T1/2 | ^C25S^Shh (1x) / ^C25S^ShhN (2x) Ptch1 | 2.64±0.08 / 0.78±0.48, n=3 | 0.009 |
| 4B | C3H10T1/2 | Shh (2x) / ^C25S^ShhN (2x) Ptch1 | 3.11±0.49 / 0.78±0.48, n=3 | 0.0019 |
| 4B | C3H10T1/2 | ^C25S^Shh (2x) / ^C25S^ShhN (2x) Ptch1 | 3.31±1 / 0.78±0.48, n=3 | 0.0011 |
| 4B | C3H10T1/2 | Shh (1x) / ^C25S^ShhN (2x) Gli1 | 5.7±0.6 / 1.84±0.68, n=3 | 0.0003 |
| 4B | C3H10T1/2 | ^C25S^Shh (1x) / ^C25S^ShhN (2x) Gli1 | 5.33±0.26 / 1.84±0.68, n=3 | 0.0007 |
| 4B | C3H10T1/2 | Shh (2x) / ^C25S^ShhN (2x) Gli1 | 5.5±0.79 / 1.84±0.68, n=3 | 0.0005 |
| 4B | C3H10T1/2 | ^C25S^Shh (2x) / ^C25S^ShhN (2x) Gli1 | 5.5±1.14 / 1.84±0.68, n=3 | 0.0005 |

| 5C | nt | Shh±Scube2 | 446%±177% / 392%±176%, n=7 | 0.7727 |
| --- | --- | --- | --- | --- |
| 5C | Disp^-/-^ / nt | Shh±Scube2 | 131%±35% / 392%±176%, n=7 | 0.0023 |
| 5C | Disp^-/-^ / nt | Shh+Scube2 | 101%±38% / 392%±176%, n=7 | 0.0008 |

| 6B | C3H10T1/2 | Shh/ ^C25S^Shh (1x) Ptch1 | 1.685±0.31 / 1.44±0.16, n=3 | 0.9632 |
| --- | --- | --- | --- | --- |
| 6B | C3H10T1/2 | Shh/ ^C25S^Shh (2x) Ptch1 | 1.653±0.49 / 1.89±0.33, n=3 | 0.9683 |
| 6B | C3H10T1/2 | Shh/ ^C25S^Shh (1x) Gli1 | 4.24±0.23 / 4.47±0.26, n=3 | 0.9691 |
| 6B | C3H10T1/2 | Shh/ ^C25S^Shh (2x) Gli1 | 4.44±0.74 / 4.47±1.03, n=3 | >0.9999 |
| 6B | C3H10T1/2 | Shh (1x) / ^C25S^ShhN (2x) Ptch1 | 1.685±0.31 / 1.3±0.6, n=3 | 0.0013 |
| 6B | C3H10T1/2 | ^C25S^Shh (1x) / ^C25S^ShhN (2x) Ptch1 | 1.44±0.16 / 1.3±0.6, n=3 | 0.0053 |
| 6B | C3H10T1/2 | Shh (2x) / ^C25S^ShhN (2x) Ptch1 | 1.653±0.49 / 1.3±0.6, n=3 | 0.0015 |
| 6B | C3H10T1/2 | ^C25S^Shh (2x) / ^C25S^ShhN (2x) Ptch1 | 1.89±0.33 / 1.3±0.6, n=3 | 0.0004 |
| 6B | C3H10T1/2 | Shh (1x) / ^C25S^ShhN (2x) Gli1 | 4.24±0.23 / 1.3±0.6, n=3 | 0.0008 |
| 6B | C3H10T1/2 | ^C25S^Shh (1x) / ^C25S^ShhN (2x) Gli1 | 4.47±0.26 / 1.3±0.6, n=3 | 0.0005 |
| 6B | C3H10T1/2 | Shh (2x) / ^C25S^ShhN (2x) Gli1 | 4.44±0.74 / 1.3±0.6, n=3 | 0.0005 |
| 6B | C3H10T1/2 | ^C25S^Shh (2x) / ^C25S^ShhN (2x) Gli1 | 4.47±1.03 / 1.3±0.6, n=3 | 0.0005 |

Figure Supplements

| FS1B | Disp^-/-^ / nt | Shh-NanoLuc | 65%±30% / 2745%±1186%, n=3 | <0.0173 |
| --- | --- | --- | --- | --- |

| F1S1K | nt | Shh±Scube2 | 83.7±38% / 31.5±15%, n=7 | 0.0005 |
| --- | --- | --- | --- | --- |
| F1S1K | Disp^-/-^ / nt | Shh±Scube2 | 56.9±13% / 31.5±15%, n=7 | 0.1034 |
| F1S1K | Disp^-/-^ / nt | Shh+Scube2 | 40±11% / 31.5±15%, n=7 | 0.8103 |
| F1S1L | nt | ^C25S^Shh±Scube2 | 223±102% / 93.3±22%, n=3 | 0.2739 |
| F1S1L | Disp^-/-^ / nt | ^C25S^Shh±Scube2 | 514±126% / 93.3±22%, n=3 | 0.0014 |
| F1S1L | Disp^-/-^ / nt | ^C25S^Shh+Scube2 | 262±87% / 93.3±22%, n=3 | 0.1307 |
| F1S1M | nt | ShhN±Scube2 | 199±137% / 170±66%, n=4 | 0.9819 |
| F1S1M | Disp^-/-^ / nt | ShhN±Scube2 | 268±146% / 170±66%, n=4 | 0.6471 |
| F1S1M | Disp^-/-^ / nt | ShhN+Scube2 | 230±185% / 170±66%, n=4 | 0.8752 |

| FS5F | Bosc23 | Shh (50μl-300μl) | 0.7±0.1, 0.9±0.3, 1.8±0.27, 2.0±0.56, n=4 ea |  |
| --- | --- | --- | --- | --- |
| FS5F | Bosc23 | ^C25S^Shh (50μl-300μl) | 0.6±0.04, 0.77±0.02, 1.7±0.1, 1.7±0.35, n=4 ea | 0.89, 0.78, 0.91, 0.33 |
| FS5F | Bosc23 | ^C25S^ShhN (50μl-300μl) | 0.02±0.01, 0.04±0.01, 0.02±0.015, 0.15±0.12, n=4 ea | <0.0001 for all amounts |
| FS5F | HEK293 | R&D 8908-SH 1ng-6ng) | 0.4±0.04, 0.52±0.1, 0.9±0.08, 0.9±0.09, n=4 ea | 0.13, 0.033, <0.0001, <0.0001 |

| F8S1A | eye disc | w^1118^ control | 712±39, n=20 | 0.0002 |
| --- | --- | --- | --- | --- |
| F8S1A | eye disc |  | 186±26, n=20 | <0.0001 |
| F8S1A | eye disc | Hh | 757±34, n=16 |  |
| F8S1A | eye disc | ^HA^Hh | 266±36, n=20 | <0.0001 |
| F8S1A | eye disc | Hh^HA^ | 769±16, n=16 | 0.6999 |
| F8S1A | eye disc | ^C85S;Δ86-99^Hh | 239±25, n=8 | <0.0001 |
| F8S1B | wing disc | w^1118^ control | 1.147±0.03, n=10 | <0.0001 |
| F8S1B | wing disc | Hh | 1.92±0.17, n=10 |  |
| F8S1B | wing disc | ^HA^Hh | 0.14±0.02, n=11 | <0.0001 |
| F8S1B | wing disc | Hh^HA^ | 1.68±0.15, n=12 | <0.0001 |
| F8S1B | wing disc | ^C85S;Δ86-99^Hh | 0.9±0.03, n=7 | <0.0001 |
