# Supplementary material for: Two-way Dispatched function in Sonic hedgehog shedding and transfer to high-density lipoproteins
Source: eLife. 2024 Sep 19;12:RP86920. doi: 10.7554/eLife.86920 (PMC11412720; doi:10.7554/eLife.86920)
Supplement: Supplementary file 2. [file elife-86920-supp2.docx]

**Supplementary File 2: qPCR target genes analyzed in this work**

| 1 | control, normalization of 2-21 | **Actb** | β-actin | Control, cytoplasmic |
| --- | --- | --- | --- | --- |
| 2 | Shh signaling | **Ptch1** | Patched homolog 1 | Receptor for Shh/Ihh/Dhh |
| 3 | Shh signaling | **Ptch2** | Patched homolog 2 | Possible receptor for Shh, plays a role in control of cellular growth |
| 4 | Shh signaling | **Gli1** | Zinc finger protein GLI1 | Transcriptional activator, binds to DNA consensus 5'-GACCACCCA-3', craniofacial/digit/CNS/gastrointestinal development. Direct target of Hh |
| 5 | Shh signaling | **Gli2** | Zinc finger protein GLI2 | Transcriptional activator (repressor) in Shh pathway, binds to DNA sequence 5'-GAACCACCCA-3' (part of TRE-2S regulatory element) |
| 6 | Shh signaling | **Gli3** | Zinc finger protein GLI3 | Dual function: transcriptional activator (GLI3A full length after phosphorylation and nuclear translocation) and repressor (GLI3R, C-terminally truncated) of Shh pathway, binds to 5'-GGGTGGTC-3' |
| 7 | osteogenic differentiation | **Alpl** | Alkaline phosphatase (pan-expressed) | Key role in skeletal mineralization by regulating levels of diphosphate (PPi) |
| 8 | osteogenic differentiation | **Spp1** | Secreted phosphoprotein 1 (= Osteopontin) | Binds to hydroxyapatite, part of mineralized matrix, important for cell-matrix interactions |
| 9 | osteogenic differentiation | **Bglap** | Bone γ-carboxy-glutamate protein (Osteocalcin) | Hormone, secreted by osteoblasts, functions in bone remodeling and energy metabolism |
| 10 | osteogenic differentiation | **Runx2** | Runt related transcription factor 2 | Essential for osteoblastic differentiation and skeletal morphogenesis |
| 11 | adipogenic differentiation | **Dlk1** | Delta like non-canonical Notch ligand 1 (= Pref1) | Preadipocyte marker. TM protein, inhibits adipocyte differentiation |
| 12 | adipogenic differentiation | **Ppar-**γ | Peroxisome proliferator-activated receptor γ | Proadipogenic. Ligand-activated TF, binds to DNA-specific PPAR response elements, key regulator of adipocyte differentiation and glucose homeostasis |
| 13 | adipogenic differentiation | **Fabp4** | Fatty acid-binding protein (=Ap2) | Differentiated adipocytes (lipid transport protein in adipocytes, binds long chain fatty acids and retinoic acid) |
| 14 | adipogenic differentiation | **Cfd** | Complement factor D (= adipsin) | Differentiated adipocytes |
| 15 | TAG biosynthesis | **Dgat2** | Diacylglycerin-acyltransferase 2 | Acylation of DAG, → triacylgylcerol (TAG) |
| 16 | chondrogenic differentiation | **Sox9** | SRY (sex determining region Y)-bos 9 | Promotes expression of genes in chondrogenesis, including cartilage matrix protein-coding genes COL2A1, COL4A2, COL9A1, COL11A2 and ACAN, SOX5 and SOX6. |
| 17 | chondrogenic differentiation | **Col2**α**1** | Collagen type II α I | Fibril-forming, major component of cartilage |
| 18 | chondrogenic differentiation | **Col10**α**1** | Collagen type X α I | Product of hypertrophic condrocytes, localised to presumptive mineralisation zones of hyaline cartilage |
| 19 | chondrogenic differentiation | **Col1**α**1** | Collagen type I α I | Fibril-forming, most abundant protein of bone/skin/tendon ECM |
| 20 | chondrogenic differentiation | **Mmp3** | Matrix metallopeptidase 3 | Codes for Stromelysin-1, degrades fibronectin, laminin, gelatins of type I, III, IV, and V; collagens III, IV, X, and IX, and cartilage proteoglycans. Activates procollagenase. Promotes cartilage degeneration. |
| 21 | proliferation | **Cdk9** | Cyclin-dependent kinase 9 (CDC2-related kinase) | Involved in regulation of transcription, component of TAK/P-TEFb complex forms complex with/is regulated by CyclinT or CyclinK |
| 21 | proliferation | **Mki67** | Antigen identified by monoclonal antibody Ki67 | Required to maintain individual mitotic chromosomes (associates with surface), dispersed in the cytoplasm following nuclear envelope disassembly |
