# Supplementary material for: Two-way Dispatched function in Sonic hedgehog shedding and transfer to high-density lipoproteins
Source: eLife. 2024 Sep 19;12:RP86920. doi: 10.7554/eLife.86920 (PMC11412720; doi:10.7554/eLife.86920)
Supplement: Supplementary file 3. [file elife-86920-supp3.docx]

**Supplementary File 3: Information regarding amplicons and primers**

| **name** | **isoform** | **accession number** | **FWD primer (5'-3')** | **REV primer (3'-5')** | **Amplicon length** |
| --- | --- | --- | --- | --- | --- |
| **Actin β** | 1 | [NM_007393.5](https://www.ncbi.nlm.nih.gov/nuccore/NM_007393.5) | CTATTGGCAACGAGCGGTTC | CGGATGTCAACGTCACACTTC | 124 |
| **Ptch1** | 2 | [NM_001328514.1, NM_008957.3](https://www.ncbi.nlm.nih.gov/nuccore/NM_001328514.1) | GGGCTACGACTATGTCTCTC | CTTTGATGAACCACCTCCAC | 99 |
| **Ptch2** | 2 | [NM_001312903.1, NM_008958.3](https://www.ncbi.nlm.nih.gov/nuccore/NM_001312903.1) | TCTGTGCCCTGCTTCTACTC | GCCCAGGAATCCCATGATAC | 101 |
| **Gli1** | 1 | [NM_010296.2](https://www.ncbi.nlm.nih.gov/nuccore/NM_010296.2) | CCCTGGTGGCTTTCATCAAC | TGACTCATCTGAGGTGGGAATC | 109 |
| **Gli2** | 1 | [NM_001081125.1](https://www.ncbi.nlm.nih.gov/nuccore/NM_001081125.1) | CAACTCAGCAGCAGTAGCAG | CTCCGCTTATGAATGGTGATGG | 110 |
| **Gli3** | 1 | [NM_008130.3](https://www.ncbi.nlm.nih.gov/nuccore/NM_008130.3) | GTGGTTCCTATGGGCACTTATC | GTCGGCTTAGGATCTGTTGATG | 108 |
| **Dlk1** | 4 | [NM_001190703.1, NM_001190704.1, NM_001190705.1, NM_010052.5](https://www.ncbi.nlm.nih.gov/nuccore/NM_001190703.1) | GGCTGTGTCAATGGAGTCTG | AAGCCCGAACGTCTATTTCG | 91 |
| **Pparg** | 4 | [NM_001127330.2, NM_001308352.1, NM_001308354.1, NM_011146.3](https://www.ncbi.nlm.nih.gov/nuccore/NM_001127330.2) | TCCATTCACAAGAGCTGACC | GGTGGAGATGCAGGTTCTAC | 99 |
| **Fabp4** | 1 | [NM_024406.3](https://www.ncbi.nlm.nih.gov/nuccore/NM_024406.3) | GTGTGATGCCTTTGTGGGAAC | CATGCCTGCCACTTTCCTTG | 106 |
| **Cfd** | 3 | [NM_001291915.2, NM_001329541.1, NM_013459.4](https://www.ncbi.nlm.nih.gov/nuccore/NM_001291915.2) | CCTGAACCCTACAAGCGATG | CAACGAGGCATTCTGGGATAG | 117 |
| **Dgat2** | 1 | [NM_026384.3](https://www.ncbi.nlm.nih.gov/nuccore/NM_026384.3) | GGCTGATAGCTGTGCTCTAC | GATGGGAAAGTAGTCTCGGAAG | 128 |
| **AlpI** | 5 | [NM_001287172.1, NM_007431.3](https://www.ncbi.nlm.nih.gov/nuccore/NM_001287172.1) | CTGCAAGGACATCGCATATCAG | CCACATCAGTTCTGTTCTTCGG | 104 |
| **Spp1** | 5 | [NM_001204201.1, NM_001204202.1, NM_001204203.1, NM_001204233.1, NM_009263.3](https://www.ncbi.nlm.nih.gov/nuccore/NM_001204201.1) | ACAGAATGCTGTGTCCTCTG | GGTCTCCATCGTCATCATCATC | 122 |
| **Bglap** | 2 | [NM_001032298.3 (Bglap2), NM_007541.3 (Bglap)](https://www.ncbi.nlm.nih.gov/nuccore/NM_001032298.3) | CCAAGCAGGAGGGCAATAAG | CTCGTCACAAGCAGGGTTAAG | 122 |
| **Runx2** | 6 | [NM_001145920.2, NM_001146038.2, NM_001271627.1, NM_001271630.1, NM_001271631.1, NM_009820.5](https://www.ncbi.nlm.nih.gov/nuccore/NM_001145920.2) | ACACTGCCACCTCTGACTTC | GGGATGAAATGCTTGGGAACTG | 117 |
| **Sox9** | 1 | [NM_011448.4](https://www.ncbi.nlm.nih.gov/nuccore/NM_011448.4) | CGGAACAGACTCACATCTCTCC | GACCCTGAGATTGCCCAGAG | 123 |
| **Col2a1** | 2 | [NM_001113515.2, NM_031163.3](https://www.ncbi.nlm.nih.gov/nuccore/NM_001113515.2) | CTGAAGGTGCTCAAGGTTCTC | GATCCTTTGGCTCCAGGAATAC | 109 |
| **Col10a1** | 1 | [NM_009925.4](https://www.ncbi.nlm.nih.gov/nuccore/NM_009925.4) | TCTCCCAGCACCAGAATCTATC | CCATGAACCAGGGTCAAGAAC | 82 |
| **Col1a1** | 1 | [NM_007742.4](https://www.ncbi.nlm.nih.gov/nuccore/NM_007742.4) | TGGTCCACAAGGTTTCCAAG | CATCTCCATTCTTGCCAGGAG | 107 |
| **Mmp3** | 1 | [NM_010809.2](https://www.ncbi.nlm.nih.gov/nuccore/NM_010809.2) | ACTTGTCCCGTTTCCATCTC | GGTTCCAGAGAGTTAGACTTGG | 118 |
| **Cdk9** | 1 | [NM_130860.3](https://www.ncbi.nlm.nih.gov/nuccore/NM_130860.3) | CAGCTCTGTGGCTCCATCAC | GTCCTTCACCTTCCGCTTCTG | 105 |
| **Mki67** | 1 | [NM_001081117.2](https://www.ncbi.nlm.nih.gov/nuccore/NM_001081117.2) | TGAGGCTGAGACATGGAGAC | GGTTCCTTTCCAAGGGACTTTC | 121 |
